# Photocatalytic Denitrative Homo- and Cross-Coupling of Nitroalkanes toward the Synthesis of Alkenes

**DOI:** 10.1021/jacs.6c06459

**Published:** 2026-07-16

**Authors:** Max Dietel, Mangish Ghosh, Hendrik Paps, Zen Shimizu, Jonas Scholl, Michael Andreas Gruber, Oliver Reiser

**Affiliations:** † Institut für Organische Chemie, 9147 Universität Regensburg, Regensburg 93053, Germany; ‡ Klinik für Anästhesiologie, 39070 Universitätsklinikum Regensburg, Regensburg 93053, Germany

## Abstract

Alkenes serve as fundamental motifs across natural products and synthetic molecules, yet efficient, mild, and versatile olefination strategies remain in demand. Nitroalkanes, renowned for their broad reactivity as nucleophiles or corresponding radicals, still offer untapped potential in photocatalysis. Here, we report a unified, photocatalyzed generation of α-nitroalkyl radicals via oxidation of simple, nonprefunctionalized nitronates, followed by addition to another nitronate (formal homo- or cross-coupling) and subsequent double denitration to furnish alkenes. This transformation leverages both the pro-nucleophilic and pro-electrophilic nature of nitroalkanes, challenging the classic *McMurry*-type coupling protocol. Moreover, the synthetic manifold is scalable and demonstrates its applicability by synthesizing medicinally relevant Combretastatin and lignan-type cores.

## Graphical abstract

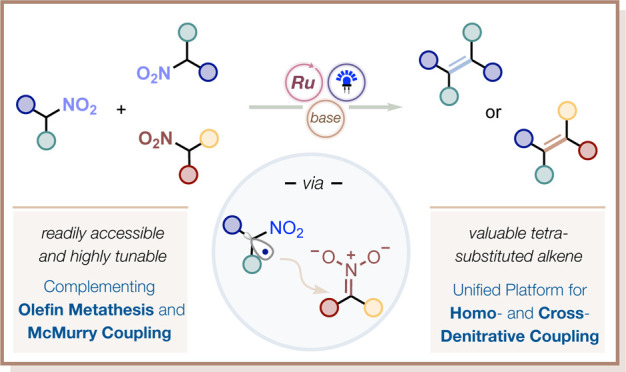

## Introduction

The ubiquity of alkenes in natural products and synthetic molecules underscores their essential role in modern organic chemistry. Considering the universal applicability and diverse reactivity of olefins, efficient methods for their synthesis are highly desirable. Classical approaches toward the formation of substituted alkenes, such as *Aldol-, McMurry-, Wittig*-, *Horner-Wadsworth-Emmons* reactions or *Julia*- and *Peterson*-olefinations commonly involve a carbonyl group (Figure 1A, top). Other olefination strategies involve base- or acid-mediated eliminations, alkene metathesis, or transition-metal-catalyzed cross-couplings.^1^ While the yields, scope, selectivity, and synthetic utility of these reactions are impressive, most strategies require a second preactivated reaction partner or stoichiometric reagents. The titanium-mediated *McMurry* coupling stands out because it enables the coupling of two carbonyl compounds, yielding substituted alkenes that are otherwise difficult to obtain.^2,3^ However, handling the harsh and strongly reducing reaction conditions and limited functional group compatibility still poses a notable challenge. Additionally, the active Ti(0) species is generally employed stoichiometrically, with few exceptions that use specially designed catalytic systems.^4^ We report here a complementary approach to the *McMurry* coupling that allows the direct photochemical coupling of unactivated nitroalkanes to alkenes under mild conditions.

**Figure 1:**
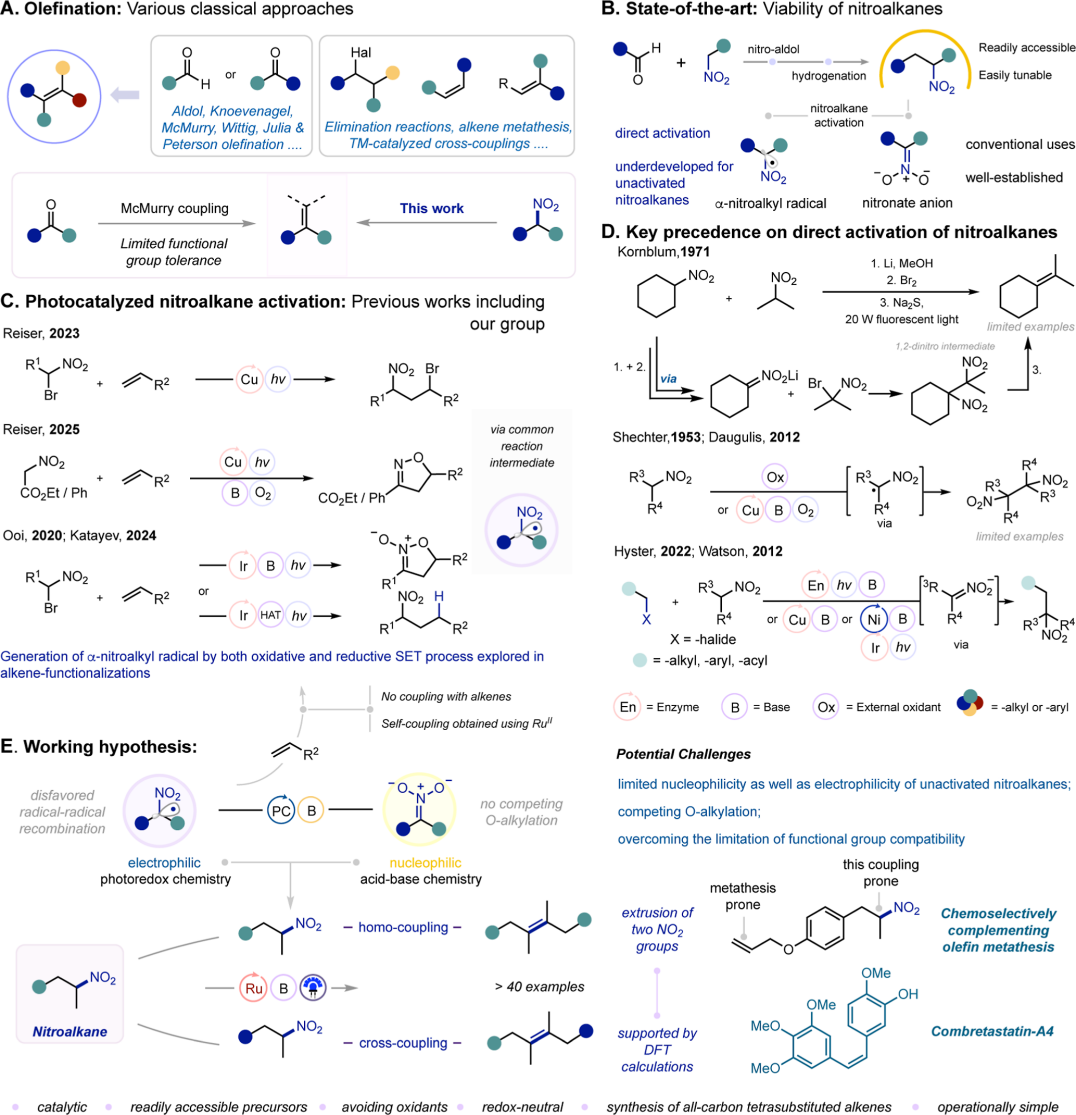
Background on olefin synthesis. (A) Various classical approaches for olefination (B) viability of nitroalkanes (C) photocatalyzed nitroalkane activation (D) key precedence on direct activation of nitroalkanes (E) our working hypothesis.

Nitroalkanes are readily available from commercial sources and find use as versatile building blocks in organic synthesis (Figure 1B).^5,6^ For instance, classic strategies such as the *Henry* reaction or *Michael* additions benefit from the nitronate ion’s strong nucleophilicity.^7^ Recent advances in the photochemical and photocatalytic activation of nitroarenes and iron nitrate complexes have enabled diverse bond-forming reactions through radical intermediates, highlighting the broad synthetic utility of nitro-derived reactive species.^8–11^

On the other hand, the formation of α-nitroalkyl radicals by the direct activation of unfunctionalized nitroalkanes and their subsequent use in streamlined synthesis remains underexplored due to the intrinsically low electrophilicity and nucleophilicity of the parent nitroalkanes. As part of the resurgence of photoredox catalysis as a robust and sustainable tool for synthesizing complex molecules from readily available feedstocks, open-shell α-nitroalkyl radical species are finding increasing attention: For example, our group previously demonstrated that α-halo nitroalkanes could be activated, producing α-nitroalkyl radicals with the use of [Cu^I^(dap)_2_]Cl as a photocatalyst, and very recently, we found that the same intermediates could also be generated from nitroesters or phenylnitromethane using catalytic [Cu^II^(dap)]­Cl_2_ under visible light and an aerobic atmosphere (Figure 1C).^12,13^ Similarly, the Ooi and the Katayev groups independently demonstrated the formation of these radical intermediates from α-halo nitroalkanes through iridium-photocatalysis.^14–16^ In these approaches, the resulting radicals underwent addition to alkenes, affording different products depending on the reaction conditions (Figure 1C). On the contrary, in our present work we find that unfunctionalized nitroalkanes in the presence of catalytic Ru^II^(bpy)_3_Cl_2_ under visible light irradiation do not engage in alkene addition but instead undergo formal dimerization with double denitration, forging C=C bonds (Figure 1E).

Relevant to our current study, Shechter et al. investigated the radical–radical recombination of α-nitroalkyl radicals starting from nitroalkanes using superstoichiometric oxidants, which formed vicinal dinitro compounds.^17^ More recently, Daugulis et al. achieved the same products, but under aerobic copper-catalyzed conditions (Figure 1D).^18^ In the mid-1900s, *Kornblum* pioneered several transformations involving nitroalkanes, especially by developing substitutions of a nitro group with different functionalities.^19,20^ Strikingly, a S_N_-type cross-coupling of nitronates with bromonitroalkanes was developed toward vicinal dinitro compounds, which were subsequently transformed into unsymmetric tetra-substituted olefins (Figure 1D).^21^ In the past few years, multiple strategies were published by Watson et al. and Hyster et al. using the nitronate’s C–N double bond as a radical acceptor (Figure 1D).^22–25^

Based on these previous synthetic campaigns, we show here that upon visible-light irradiation of simple nitroalkanes in the presence of Ru­(bpy)_3_Cl_2_ as a photocatalyst and a base, α-nitroalkyl radicals are generated. Their strong electrophilicity disfavors the radical–radical recombination, paving the way for coupling with another nitronate counterpart, which facilitates the formation of C–C bonds with no competing O-alkylation being observed (Figure 1E). Supported by a mechanistic study, the formal extrusion of two NO_2_ groups leads to the formation of di- or tetra-substituted olefins, mimicking *McMurry*-type products. Besides the coupling of two identical nitroalkanes, two structurally distinct ones can also be coupled, yielding unsymmetric alkenes. The title transformation is operationally simple, proceeds under mild reaction conditions, avoids stoichiometric oxidants or reductants, and exhibits broad functional group compatibility. The resulting tetra-substituted olefins bear close structural resemblance to their hydrogenated derivatives, dibenzylbutanes–a subclass of lignans. The synthesis of Combretastatin-A4, a clinically validated anticancer pharmacophore, underscores the method’s applicability to medicinally relevant compounds. Altogether, the developed strategy offers a concise platform for constructing architecturally complex alkenes from simple, readily available nitroalkane precursors, thereby expanding the synthetic utility of α-nitroalkyl radicals in modern organic synthesis.

## Results and Discussion

### Reaction Development

To initiate our investigation, we sought model substrates that were readily accessible and structurally amenable to postreaction analysis. In this context, 2-nitropropylarenes emerged as an ideal choice, as they can be conveniently synthesized via nitro-aldol condensation followed by borohydride-mediated hydrogenation. Moreover, their structural features enable efficient tracking of the transformation through standard analytical techniques. The study began by using 2-nitropropylbenzene (**1a**), styrene (2 equiv), and K_3_PO_4_ (2 equiv) as a base in a solution of acetonitrile under 455 nm LED irradiation in the presence of a photoredox catalyst. Neither [Cu^I^(dap)_2_]Cl nor [Cu^II^(dap)­Cl_2_] showed the conversion of the starting substrates to the corresponding ATRA- or cycloadducts (Table 1, entry 3 and 4), in sharp contrast to the previously developed couplings with bromonitroalkanes or β-nitroesters (*cf* Figure 1C). Instead, a self-coupling of **1a** had occurred when [Cu^II^(dap)­Cl_2_] was employed, leading to (2,3-dimethylbut-2-ene-1,4-diyl)­dibenzene (**2a**) in 32% yield as a 1.8:1 mixture of *E/Z*-isomers (Table 1, entry 3) based on ^1^H nuclear magnetic resonance (NMR) integration analysis. Moving from Cu-based photocatalysts to Ru^II^(bpy)_3_Cl_2_ and omitting styrene significantly increased the yield of **2a** (Table 1, entry 5 and 6). Recognizing that deprotonation of the nitroalkane is likely the initial step in the reaction mechanism, screening various bases (for full details see Supporting Information) revealed that 1,8-diazabicyclo[5.4.0]­undec-7-ene (DBU) drastically enhanced the reaction efficiency, increasing the yield of **2a** from 68% to 93% (Table 1, entry 2).

**Table 1:**
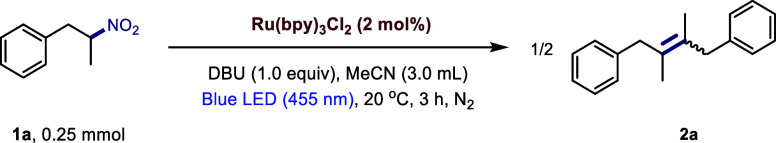
Optimization of the Homo-Coupling Reaction^a^

entry	deviations from above	^1^H NMR yield of 2a [%]
1	none	93
2	DBU (2 equiv), 18 h	93
3	[Cu^II^dap]Cl_2_ (2 mol %), styrene (2 equiv), K_3_PO_4_ (2 equiv), 18 h	32
4	[Cu^I^dap_2_]Cl (2 mol %), styrene (2 equiv), K_3_PO_4_ (2 equiv), 18 h	0
5	K_3_PO_4_ (2 equiv), styrene (2 equiv), 18 h	42
6	K_3_PO_4_ (2 equiv), 18 h	68
7	no photocatalyst, 18 h	<10
8	dark, 18 h	0
9	no base, 18 h	0
10	*fac*-Ir(ppy)_3_, 18 h	59
11	4CzIPN, 18 h	53
12	3DPA2FBN, 18 h	73

a Reaction conditions unless otherwise stated: Nitroalkane (0.25 mmol), DBU (1 equiv) and photocatalyst (2 mol %) are dissolved in dry acetonitrile (MeCN, 3 mL) under nitrogen atmosphere. The mixture was irradiated with 455 nm light-emitting diodes (LEDs) at room temperature.

Control experiments underscored the necessity of each reaction component. Omission of the photocatalyst, light, and base independently led to little or no formation of **2a** (<10, 0, and 0% yield; Table 1, entries 7 to 9, for control experiments with **1i** and **1zc** in the absence of catalyst, see Table S3, Supporting Information), which confirmed the photocatalytic nature as well as light and base sensitivity of this process. Further screening revealed that Ru­(bpy)_3_Cl_2_ proved to be the most efficient photocatalyst, although organic dyes (e.g., 4-CzIPN or 3DPA2FBN) or other transition metal complexes were also viable for this transformation, albeit with lower yields (Table 1, entries 10 to 12). The amount of base could be reduced to one equivalent, and the irradiation time shortened to 3 h without compromising the yield (Table 1, entry 1). These optimized parameters not only improved the operational simplicity but also established their practicality for further exploration and application (see Supporting Information for more details).

### Substrate Scope

With the optimized conditions in hand, we explored the substrate scope of the homocoupling reaction. Readily available, secondary 2-nitropropylbenzene derivatives were efficiently converted to the corresponding alkenes **2a**–**2q** in high yields (80%–99%) as mixtures of *E*- and *Z*-isomers (*E/Z* = 1.1–1.8:1). The robustness of the transformation was tested by introducing various functional groups present in the aromatic moieties. Benzylic alkyl groups that could have been prone to HAT (Figure 2b–d), additional aryl groups (**2e**), aryl halides (**2f**–**2h**), medicinally relevant trifluoromethyl groups (**2i**), ethers (**2j**–**2l**), pyridine moieties (**2m**), and easily oxidizable dimethylamino groups (**2n**) were tolerated well, giving rise to the desired alkenes. Importantly, reducible functional groups such as nitrile (**2o**) and ester (**2p**) were amenable substrates, in contrast to the *McMurry* coupling, which is problematic with such groups due to the strongly reducing reaction conditions required (TiCl_4_/LiAlH_4_). Benzylic nitroalkanes also underwent efficient dimerization to deliver tetraphenylethene (**2r**) and several stilbene derivatives (**2s**–**2v**). Remarkably, the stereoselectivity in these cases was significantly enhanced, favoring the *Z*-isomer with up to >20:1 *Z/E* selectivity, which can be attributed to a photoinduced isomerization under the reaction conditions (Figure 3A).^26^ A control experiment showed that for the reaction with (nitromethyl)­benzene derivates (e.g., **1s**), the catalyst can be omitted to majorly obtain *trans*-**2s** (4.8:1 = *E/Z*), caused by a direct absorption of the corresponding activated nitronates at 455 nm (*cf*. the study by Parasram et al. submitted in parallel to this journal).^27^ When our standard conditions are subsequently applied to *trans*-stilbene, photoisomerization occurs toward the *cis*-alkene (1:19 = *E/Z*). The isomerization kinetics were monitored by ^1^H NMR in which the ratio steadily increased toward the *Z*-product (Figure 3B). Upon functionalization of the aryl moiety (**1u** and **1v**), the yield for the resulting olefin dropped (68% for **2u**, 52% for **2v)** compared to simple stilbene.

**Figure 2:**
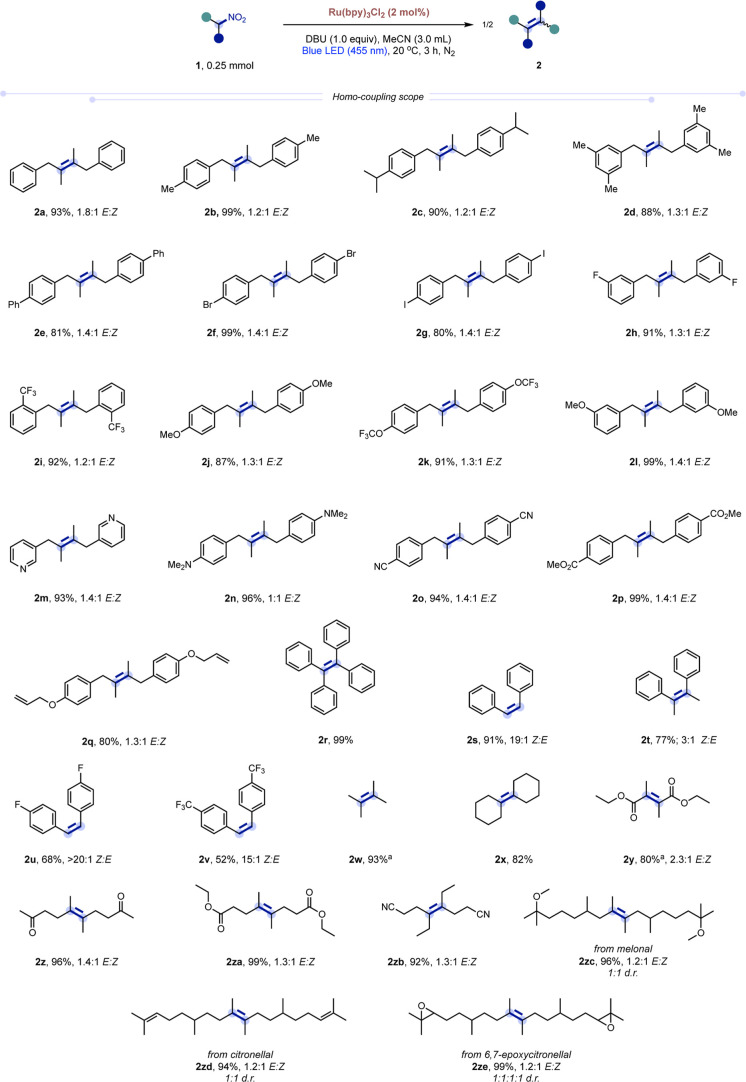
Substrate scope evaluation for the homocoupling reaction. Reaction conditions unless otherwise noted: nitroalkane (0.25 mmol), DBU (1 equiv) and Ru­(bpy)_3_Cl_2_ (2 mol %) are dissolved in dry acetonitrile (MeCN, 3 mL) under a nitrogen atmosphere. The mixture was irradiated with 455 nm light-emitting diodes (LEDs) for 3h at room temperature. Isolated yields are reported. [a] = crude ^1^H NMR yield. (*E*)-**2y** was isolated in 56% yield after workup.

**Figure 3:**
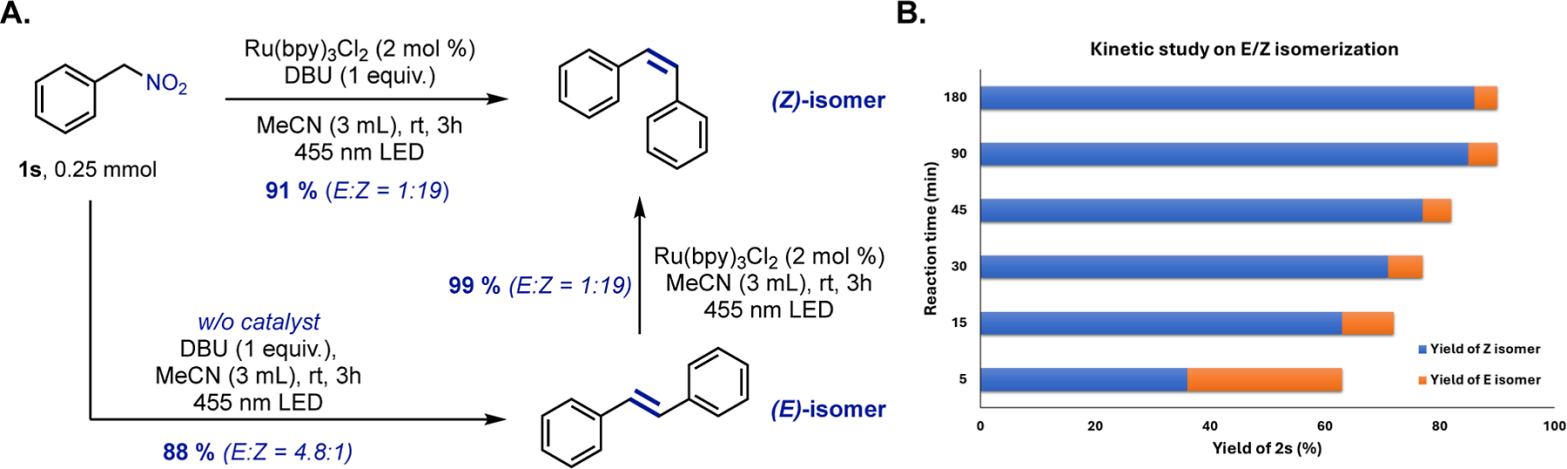
Divergent synthesis of cis- or*trans*-stilbene. (A): photodimerization of **1s** in the absence and presence of Ru­(bpy)_3_Cl_2_ (2 mol %); isolated yields. (B): kinetic study performed with substrate **1s**. Yields were determined with ^1^H NMR analysis of the crude reaction using 1,3,5-trimethylbenzene as an internal standard.

Other acyclic and cyclic aliphatic nitroalkanes as well as those containing different functional groups such as methoxy, ketone, ester, nitrile, vinyl groups, and epoxide, did not obstruct the title reaction (**2w-2ze**), highlighting the versatility of the methodology. Again, it should be noted that some of these functional groups are generally incompatible with the *McMurry* coupling due to their susceptibility to reductive conditions and reactions with titanium.^4^

Additionally, reactions using substrates **1c** or **1g** were performed on a larger scale (5.0 mmol) with an acceptable decrease in yield (90% to 65% and 80% to 66%, respectively), indicating the feasibility of this approach for preparative applications.

The homocoupling reaction was further examined using nitromethane, the smallest nitroalkane (see Supporting Information for details). Due to operational limitations, product detection was limited to qualitative analysis by gas chromatography, which indicated the formation of ethylene gas in an unknown amount. Given that nitromethane is industrially produced either by direct nitration of methane or by chlorination of the latter followed by nitration, our methodology appears feasible for the overall conversion of methane to ethylene.^28,29^

We next investigated a possible cross-coupling reaction between two structurally distinct nitroalkanes. Selected, previously employed substrates (Figure 2) were irradiated in the presence of 2-nitropropane (6 equiv), obtaining the desired cross-coupled olefins (Figure 4) in moderate to excellent yields. Functional group compatibility is again high, and with a view to distinguishing this approach from the *McMurry* coupling, aryl halides (**3c**), methoxide (**3d**) **3e**, and nitrile (**3f**) are tolerated. Homocoupling is observed only for the excess component 2-nitropropane, yielding tetramethylethylene (**2w**), which is lost during workup due to its volatility. More complex combinations of two nitroalkanes were tested, for which the use of an excess of one component was applied (1.4 equiv, 0.35 mmol), achieving the synthesis of diverse unsymmetric olefins, tolerating moieties, such as –CF_3_ (**3g**), –OMe (**3h**), –NMe_2_ (**3i**), –CN (**3m**) and –CO_2_Me (**3k**). Our method also allows for the efficient synthesis of an unsymmetric stilbene derivative (**3p**) again with high *Z*-selectivity (50:1 *Z/E*) as observed for the homocoupling cases.

**Figure 4:**
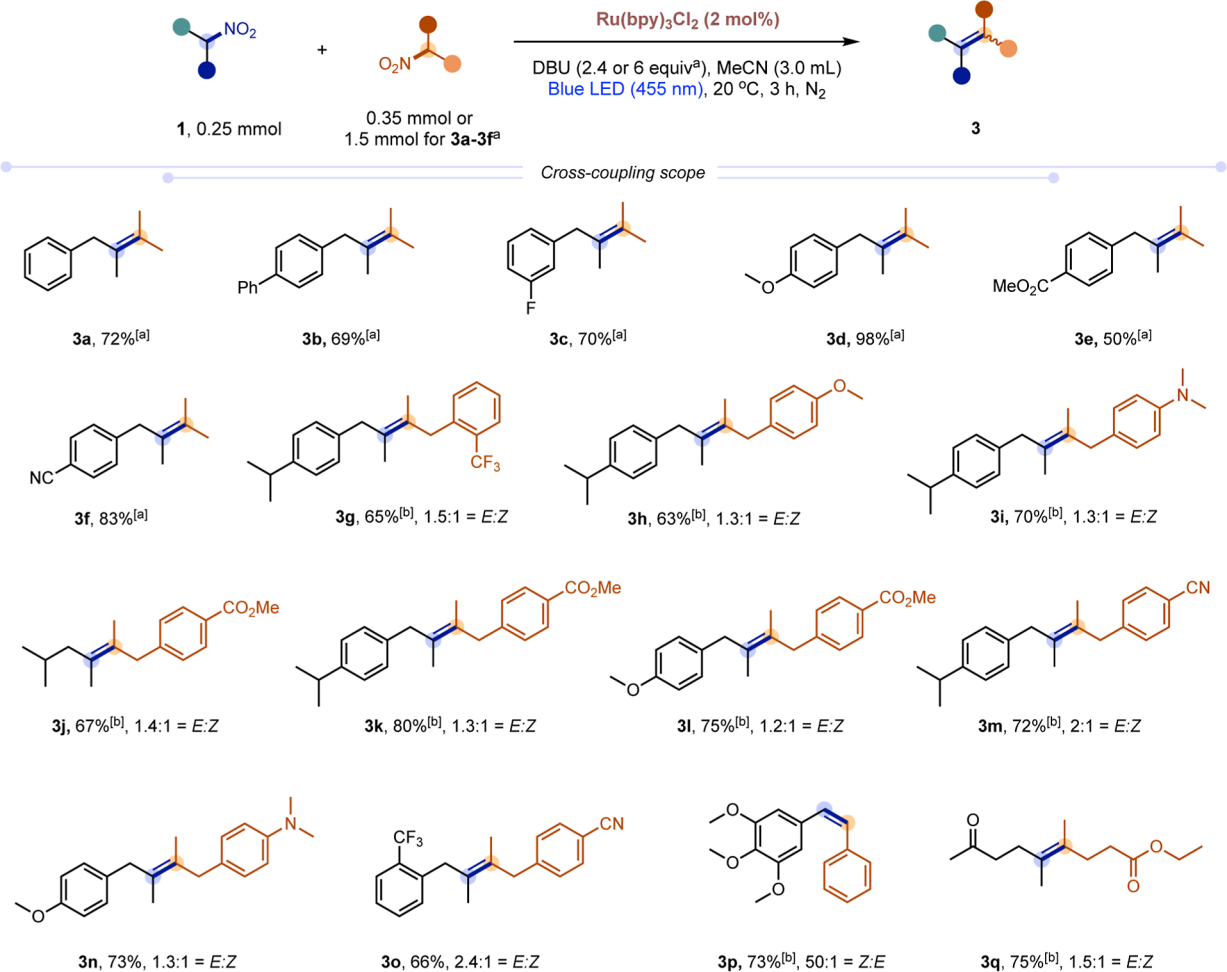
Substrate scope evaluation for the cross-coupling reaction. Nitroalkane **1** (0.25 mmol), the second nitroalkane **1′** (0.35 mmol), DBU (2.4 equiv), and Ru­(bpy)_3_Cl_2_ (2 mol %) are dissolved in anhydrous acetonitrile (MeCN, 3 mL) under a nitrogen atmosphere. The mixture was irradiated with 455 nm light-emitting diodes (LEDs) for 3h at room temperature. Isolated yields are reported with respect to the limiting nitroalkane. [a] The second nitroalkane was employed in 1.5 mmol (6 equiv). [b] The reactions were performed 2–3× with isolated yields varying ±3% and the average yield is reported (see Supporting Information for more details).

Some substrates moderately exceed yields expected from a statistical distribution, when nitroalkanes with electron-donating groups on the aryl moiety were employed as the limiting reagent (1 equiv. for ^i^Pr (**3k**, **3m**), –OMe (**3l**, **3p**)) and nitroalkanes with electron-withdrawing groups on the aryl moiety as the excess component (1.4 equiv for –CO_2_Me (**3k**, **3l** The, **3j**), –CN (**3m**). This is likely due to differences in reactivity of the nitroalkanes. For example, kinetic measurements in the homocoupling of **1z** and **1za** showed a significantly faster consumption of the former (see Figure S16 in the Supporting Information for more details). Related observations were also made in the parallel study of Parasram et al.,^27^ which will be the subject of a joint future study of our groups.

## Application

The versatility and applicability of this protocol were demonstrated through the streamlined synthesis of *Z*-dehydrobrittonin A (**5**) and a silyl-protected precursor of Combretastatin-A4 (**6**). Starting from trimethoxylated bromobenzene, a Pd-catalyzed cross-coupling with nitromethane produced the nitromethylbenzene intermediate **1zf**,^30^ which underwent homocoupling under our optimized conditions to afford dehydrobrittonin A (**5**) in 80% overall yield over two steps with excellent *cis*-selectivity (95:5). This method compares well to traditional synthetic routes, which require multiple steps and deliver a mixture of *cis*/*trans*-isomers.^31^ Brittonin A, the hydrogenated derivative of **5**, has been studied for its inhibitory activity in human myelogenous leukemia cells.^32^

Furthermore, TBS-protected Combretastatin-A4 (**6**), a potent tubulin polymerization inhibitor with strong antitumor activity,^33^ was obtained in 81% (*cis/trans* 94:6) overall yield over three steps from intermediates **1zf** and **1zg** (Figure 5A). Thereby, an efficient alternative is provided to the reported pathway via Horner–Wadsworth-Emmons reaction. Notably, only the *cis*-isomer (**6**) exhibits high binding affinity for the colchicine-binding site of tubulin, underscoring the importance of this protocol, as the classical synthesis yields isomeric mixtures.^34^ Access to these precursors suggests the potential of this method for the generalized synthesis of medicinally relevant stilbenoids.

**Figure 5:**
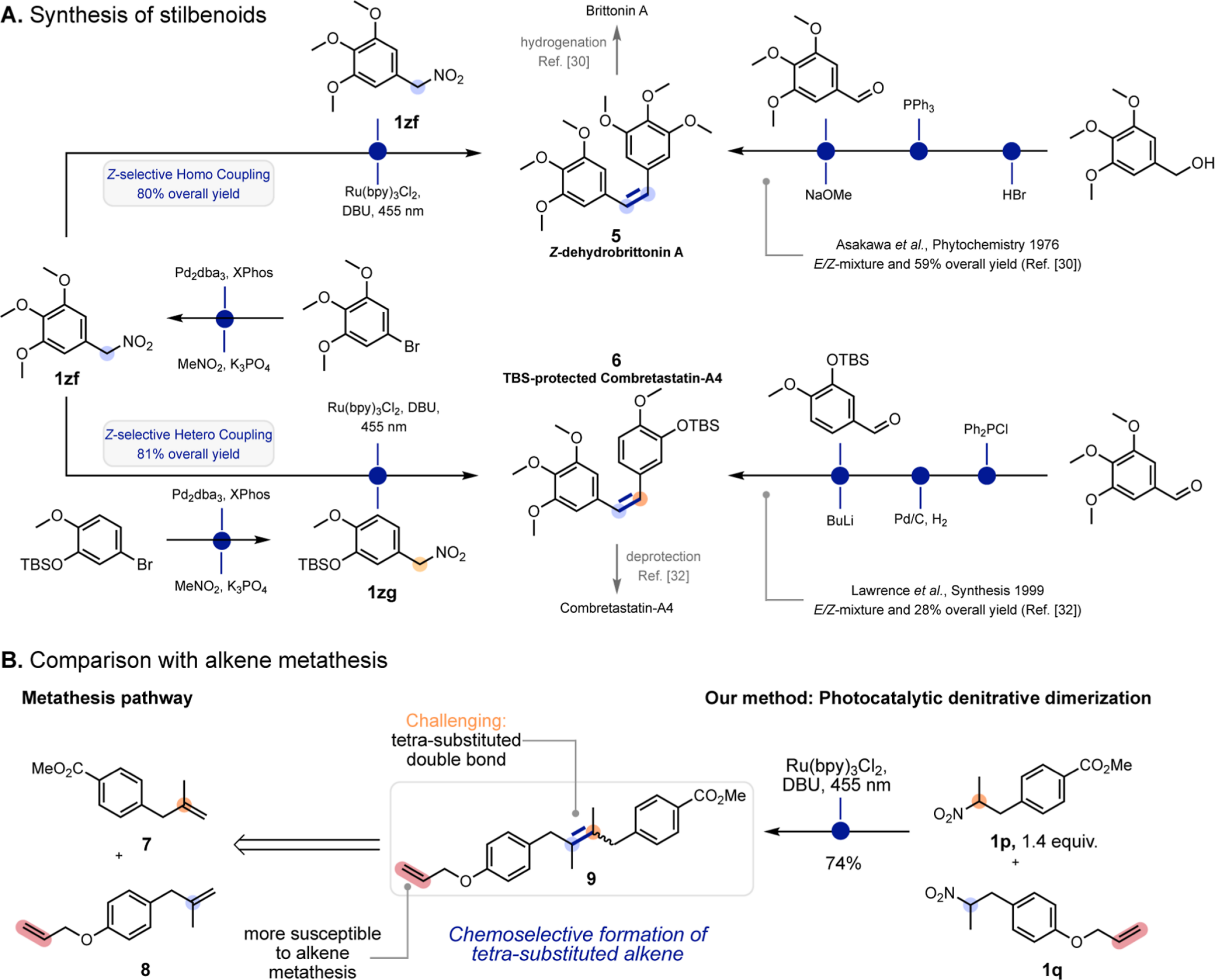
Application toward the synthesis of Combretastatin-A4 (A) and Comparison with Alkene Metathesis (B). (A): homocoupling of **1zf** and cross-coupling with **1zg** were performed with the standard protocols (vide infra). Upper: comparison between reported synthesis of Dehydrobrittonin A and our homocoupling of nitroalkane **1zf**.^31^ Lower: comparison between the first reported synthesis of a Combretastatin-A4 precursor **6** and our cross-coupling procedure between nitroalkanes **1zf** and **1zg**.^33^ (B) The cross-coupling reaction between **1p** and **1q** was performed with the standard protocol (vide infra), where **1p** was used in excess (1.4 equiv).

The current method also complements alkene metathesis (Figure 5B), exemplified with the synthesis of **9**, which would call for the cross-olefinic coupling between **7** and **8**. The latter contains an allyl ether moiety, a highly reactive functionality in classical alkene metathesis reactions,^35^ and thereby restricts the formation of tetrasubstituted alkenes.^36^ Under our developed protocol, the denitrative cross-coupling reaction between the analogous nitroalkanes **1p** and **1q** furnishes **9** with good yield (74%), showing no cross-reaction with the terminal alkene functionality.

### Mechanistic Studies and DFT Calculations

To elucidate the pathway of this transformation, several mechanistic experiments and density-functional-theory (DFT) calculations were conducted. The proposed pathway consists of six main steps: (**i**) deprotonation of a nitroalkane, (**ii**) Ru­(II)-photocatalyzed formation of an α-nitroalkyl radical via oxidation of a nitronate, (**iii**) dimerization through radical addition of an α-nitroalkyl radical onto another nitronate, (**iv**) NO_2_^–^ extrusion, (**v**) NO_2_^•^ extrusion and (**vi**) regeneration of the Ru­(II)-photocatalyst (Figure 6F).

**Figure 6:**
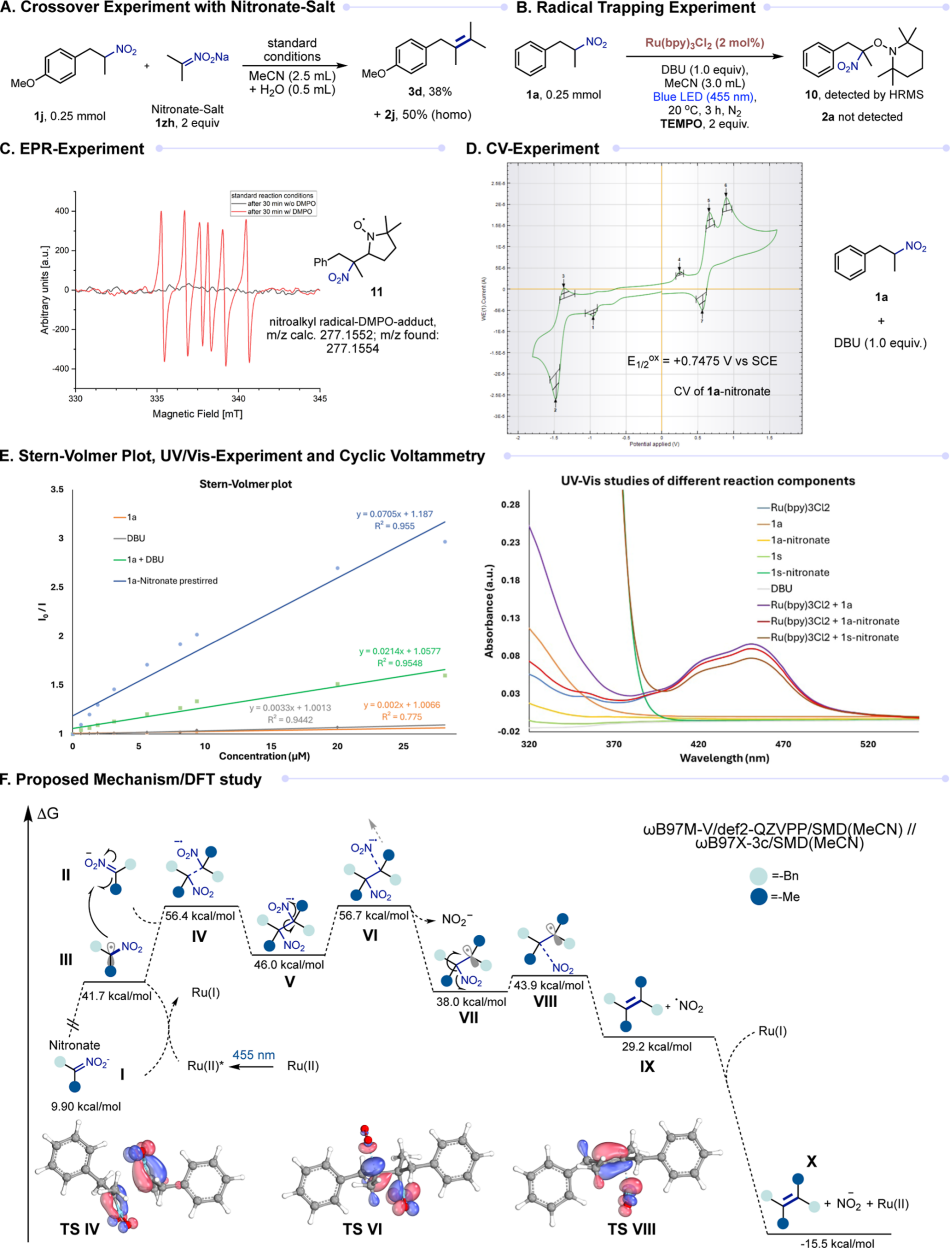
Mechanistic proposal with underlining experiments. (A). Crossover experiment. (B). Radical trapping experiment. (C). EPR experiment. (D). Cyclic Voltammetry Experiment for **1a**. (E). Stern–Volmer plot, UV/vis-Experiment. (F). Proposed mechanism and DFT-study, bottom: highest occupied molecular orbitals for transition states **IV**, **VI** and **VIII**.

In a control experiment, the nitronate salt (**1zh**) was employed as the cross-coupling partner instead of 2-nitropropane. Cross-product formation was still observed, indicating that the nitronate acts as a reaction partner, although the yield was diminished due to the salt’s poor solubility (Figure 6A). Employing 2,2,6,6-tetramethylpiperdinyl-oxyl (TEMPO) as a radical scavenger resulted in complete suppression of the reaction and adduct **10** was confirmed by HRMS (Figure 6B). Furthermore, addition of 0.8 equiv of 5,5-dimethyl-1-pyrroline *N*-oxide (DMPO) prior to irradiation for 30 min led to **11**, being characterized by its sextet EPR-signal and HRMS (Figure 6C). A cyclic voltammetry study (Figure 6D) suggests the formation of the α-nitro alkyl radical species via single-electron oxidation of nitronate **I** (*E*_1/2_^ox^ = 0.75 V vs SCE), concomitant with reduction of the excited Ru­(II)* to Ru­(I) (*E*_1/2_ = 0.77 V vs SCE).

UV/vis-absorption experiments did not reveal any interaction between the catalyst and the deprotonated substrate **1a**, suggesting purely outer-sphere electron- or energy-transfer processes. As stated above, when employing the activated nitronate of **1s**, the reaction proceeds without catalyst due to a trailing absorption up to 425 nm (Figure 6E), indicating that this species might be excited directly by light. Fluorescence quenching experiments and corresponding Stern–Volmer plots revealed that the nitronate intermediate–especially after prestirring **1a** and DBU for 60 min–efficiently quenches Ru­(bpy)_3_Cl_2_, while the neutral nitroalkane did not engage with the catalyst (Figure 6E, see Supporting Information for more details).

After radical formation, the C–C bond forming step occurs via α-nitroalkyl radical addition onto the nitronate’s C–N double bond, supported by DFT calculations (Figure 6F), which was also proposed by Hyster et al. for the addition of α-ketyl radicals to the same moiety.^24,25^ Toward the formation of a nitro radical anion dimer (Figure 6F, Intermediate **V**), the energetic level rises relative to the ground-state starting materials by Δ*G* = 46.0 kcal/mol. An analogous intermediate was likely observed by Kornblum et al., when 2 equiv of Na_2_S were added under irradiation to a neutral dinitro compound.^21^ Similarly, Evans et al. performed an electrochemical reduction of vicinal dinitro compounds to intermediate **V**, obtaining the alkene products.^37^ Next, the transient intermediate β-nitroalkyl radical **VII** is formed in a mesolytic cleavage of one carbon–nitrogen bond. Hyster et al. proposed a similar pathway after forming intermediate nitro-radical anions via ketyl-radical addition to a nitronate.^24^ Finally, product **X** is obtained through a second denitrative process, involving NO_2_^•^ extrusion. The overall energy decreases to Δ*G* = −15.5 kcal/mol relative to the starting materials, while the catalyst Ru­(II) is reformed by SET from NO_2_^•^, concurrently forming nitrite ions.

## Conclusion

The presented development of a homo- and cross-coupling strategy of secondary and primary nitroalkanes provides access to diverse alkyl- and aryl-substituted alkenes with broad functional group tolerance. In contrast to traditional *McMurry* couplings, various reducible functionalities such as carbonyl compounds and epoxides can be employed, giving direct access to alkenes that were previously inaccessible. Notably, this procedure enabled the synthesis of a valuable stilbenoid drug precursor, TBS-protected Combretastatin-A4, with high *cis*-selectivity. Mechanistic experiments supported the unique reaction pathway, revealing the generation of α-nitroalkyl radicals from unmodified nitroalkanes under mild conditions. Thus, we anticipate that this advancement in alkene synthesis will find broad applications in synthetic chemistry, enabling efficient access to complex molecular scaffolds for pharmaceutical research.

## Supplementary Material



## Data Availability

The primary research data of this study are openly available in Radar4Chem at DOI: 10.22000/tkzauw5dgt4fzs0y.

## Notes

Additional experimental details, materials, and methods, including photographs of experimental setups (PDF)
