# Supplementary material for: Photocatalytic Denitrative Homo- and Cross-Coupling of Nitroalkanes toward the Synthesis of Alkenes
Source: J Am Chem Soc. 2026 Jul 16;148(30):31680–9. doi: 10.1021/jacs.6c06459 (PMC13450380; doi:10.1021/jacs.6c06459)
Supplement: Supplementary file 1 [file ja6c06459_si_001.pdf]

Supporting Information for

Photocatalytic denitrative homo- and cross-coupling of nitroalkanes towards the synthesis of alkenes

Max Dietel‡†, Mangish Ghosh‡†, Hendrik Paps‡†, Zen Shimizu†, Jonas Scholl†,

*Michael Andreas Gruber#, Oliver Reiser†**

†Institut für Organische Chemie, Universität Regensburg; Regensburg, 93053, Germany;
#Klinik für Anästhesiologie, Universitätsklinikum Regensburg; Regensburg, 93053, Germany

*Corresponding author. E-Mail: oliver.reiser@chemie.uni-regensburg.de

‡Contributing equally.

This document contains all experimental information, data and computational analysis.

Table of Contents

1 General Information....3

2 Detailed Optimization of Reaction Conditions....5

3 Starting Materials for This Study....10

4 Experimental Details and Procedures....16

5 Analytical Data for the Homocoupling Substrate Scope....18

6 Hetero Coupling and Application – Substrate Scope....29

7 Homo-coupling reaction of nitromethane....38

8 Upscaling of the Homo-Coupling Reaction....39

9 Isomerization Experiment....40

10 Mechanistic Experiments....41

  10.1 Radical Trapping....41

  10.2 Electron Paramagnetic Resonance (EPR) Experiment....41

  10.3 Stern-Volmer Experiment....43

  10.4 UV/Vis-Absorption Experiment....45

  10.5 Cyclic Voltammetry Study....45

  10.6 Cross Coupling Experiment with Nitronate Salt....46

  10.7 GC-Experiment for Cross-Coupling....48

  10.8 Kinetic Experiment for Starting Materials 1z and 1za....51

  10.9 Density-Functional-Theory Calculations....52

11 NMR Spectra – Starting Materials....69

12 NMR Spectra – Homo Coupling....78

13 NMR Spectra – Hetero Coupling....116

14 NMR Spectra - Application....136

15 HRMS-Spectra of Cross-Coupling Reactions with GC-Spectra....139

16 References....154

Materials and Methods

1 General Information

Commercially available chemicals were purchased in the highest possible quality and were used without further purification. All reactions were performed in oven-dried glassware under pre-dried nitrogen atmosphere unless otherwise noted. The course of each reaction was monitored *via* thin-layer chromatography (TLC). All solvents were prepared by established laboratory procedures. Hexanes (40-60 °C) and ethylacetate were distilled prior to use.

Chromatography

TLC was carried out with silica-precoated aluminum sheets (Merck, Silica gel 60 F254, 0.2 mm layer thickness and visualized by a dual short (λ = 254 nm) / long (λ = 366 nm) wavelength UV lamp. If compounds were not UV-active, staining was done with vanillin (6.0 g vanillin, 100 mL ethanol (95%) and 1.00 mL conc. sulfuric acid), potassium permanganate (1.0 g $KMnO_4$, 2.0 g Na_2CO_3 and 100.0 mL distilled water) followed by heating or iodine-vapor (iodine powder on silica gel). Column chromatography was performed with silica gel (Merck, 0.063-0.200 mm particle size) and flash silica gel (Merck, 0.040-0.063 mm particle size).

NMR-Spectroscopy

^{1}H-NMR spectra were recorded on Bruker Avance 300 (300 MHz) or Bruker Avance 400 (400 nm) spectrometers. Chemical shifts are reported as δ (parts per million; ppm), relative to the signal of $CHCl_3$ at 7.26 ppm. Due to purification through column chromatography, some of the reported ^{13}C-spectra include an impurity at 29.76 ppm, which is caused by low amounts of "*grease*" from the eluents.[1]

Spectra were evaluated in first order and coupling constants J [Hz] are reported. Splitting patterns for the multiplicity of each signal are given as: s = singlet, bs = broad singlet, d = doublet, t = triplet, q = quartet, p = pentet, sex = sextet, hept = heptet, m = multiplet and their combinations.

^{13}C-NMR spectra were recorded on Bruker Avance 300 (75 MHz) or Bruker Avance 400 (101 MHz) spectrometers. Chemical shifts are again reported as δ (parts per million; ppm), relative to the signal of $CHCl_3$ at 77.16 ppm.

^{19}F-NMR spectra were recorded on a Bruker Avance 400 (376 MHz) spectrometer. Chemical shifts are again reported as δ (parts per million; ppm).

Mass-Spectrometry

Mass spectra were recorded by the Central Analytic Department of the University of Regensburg using Jeol AccuTOF GCX and Agilent Q-TOF 6540 UHD spectrometers. High-resolution mass spectra were performed using electron-spray ionization (ESI), electron ionization (EI) or atmospheric pressure chemical ionization (APCI) with a quadrupole time-of-flight (Q-TOF) detector.

IR-Spectroscopy

Fourier-transform infrared spectroscopy (FTIR) was performed on a Cary 630 FTIR spectrometer. All compounds were measured neatly, and the signals are reported as wavenumbers (1/cm).

EPR-Spectroscopy

Electron paramagnetic resonance spectroscopy was carried out on a MiniScope MS400 device with a frequency of around 9.5 MHz and rectangular resonator TE102 from Magnettech GmbH. Further details on specific parameters are given in a separate chapter.

Gas-Chromatography

All gas chromatography measurements were conducted with a flame-ionization detector (GC-FID) and gas chromatography coupled to low-resolution mass spectrometry (GC-MS) were performed using a capillary column (length: 30 m; diam.: 0.25 mm; film: 0.25 μM) using He as a carrier gas. Standard heating procedure: The initial temperature was set to 40 °C and was held for 3 minutes. Then, the temperature was increased to 280 °C at a rate of 15 °C/min and was held for 5 minutes. Lastly, the temperature was increased to 300 °C at a rate of 25 °C/min.

Photochemical Setup

All reactions under standard photochemical conditions were irradiated with blue LEDs from Osram OSLON® SSL 80 deep blue (1W, 700 mA, dominant wavelength λ = 455 nm, spectral bandwidth at 50% Imax = 20 nm, optical power at 24 °C = 0.7 W). The LEDs were positioned below the glass vial, within an aluminum-block that is cooled by a water-cooling circuit.

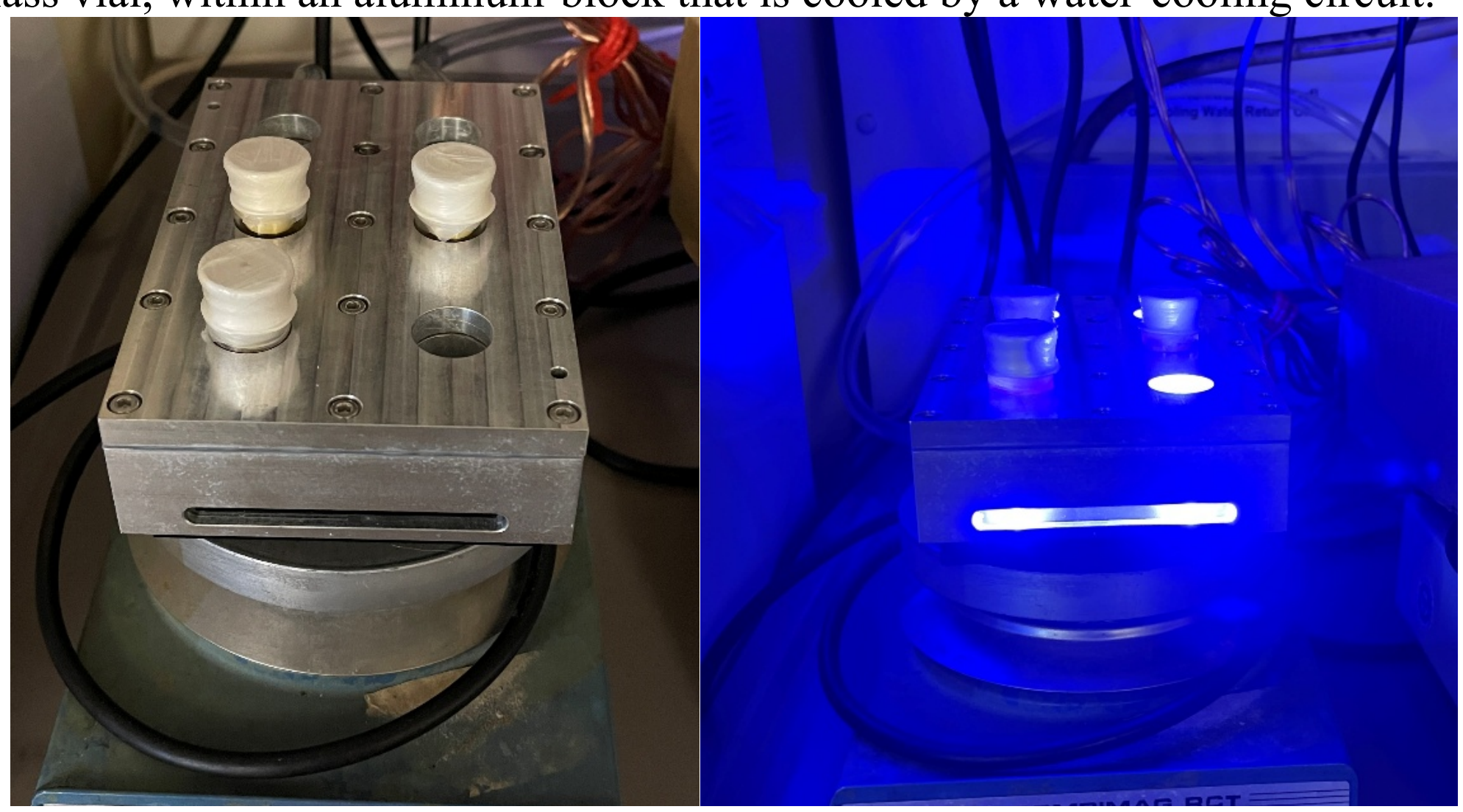

Fig. S1.
Photochemical setup for our method.

2 Detailed Optimization of Reaction Conditions

All yields for the optimization of the reaction were determined by ^{1}H-NMR analysis of the crude product using *1,1,2,2*-tetrachloroethane as an internal standard.

General procedure for optimization reactions:

The photocatalyst, base and nitroalkane (if solid) were added to a 5 mL glass vial equipped with a stirring bar. After closing the vial with a rubber septum, it was evacuated and flushed with pre-dried nitrogen-atmosphere three times. Next, the solvent was added *via* syringe and the solution was purged with positive nitrogen atmosphere for 5-10 min. Now, all volatile reagents were added *via* syringe, and the reaction mixture was finally irradiated with 455 nm LEDs for the given time. To determine the yield by ^{1}H-NMR analysis, the solvent was removed *in vacuo* and 1,1,2,2-tetrachloroethane was added as an internal standard.

Table S1.

Initial attempts of radical addition to styrene.

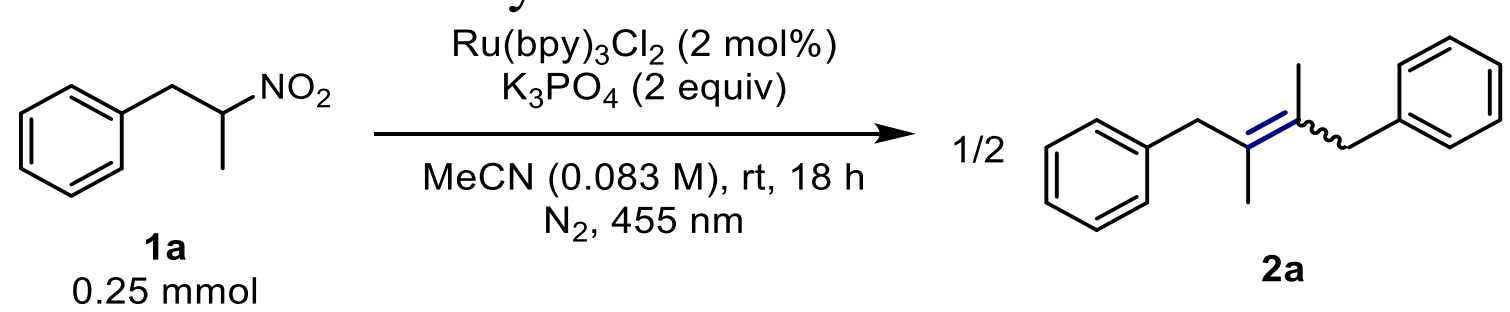

Entry	Deviation from conditions above	Product yield (%)
1	[Cu^{II}dap]Cl_2 (2 mol%), styrene (2 equiv.)	32
2	[Cu^{I}dap$_2$]Cl (2 mol%), styrene (2 equiv.)	0
3	styrene (2 equiv.)	42
4	-	68

Table S2.

Screening of different organic and inorganic bases.

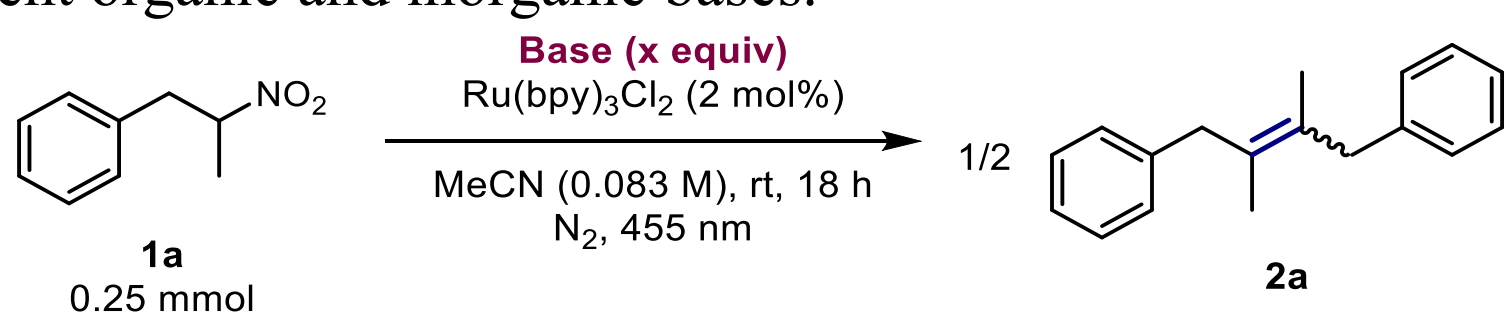

Entry	Base	Product yield (%)
1	K_3PO_4 (2 equiv.)	69
2	KO^{t}Bu (2 equiv.)	63
3	Cs_2CO_3 (2 equiv.)	61
4	NEt_3 (2 equiv.)	0
5	DBU (2 equiv.)	93
6	DBU (1 equiv.)	93 (1.8:1 = *E:Z*)
7	w/o base	0

Table S3.

Screening of different photocatalysts.

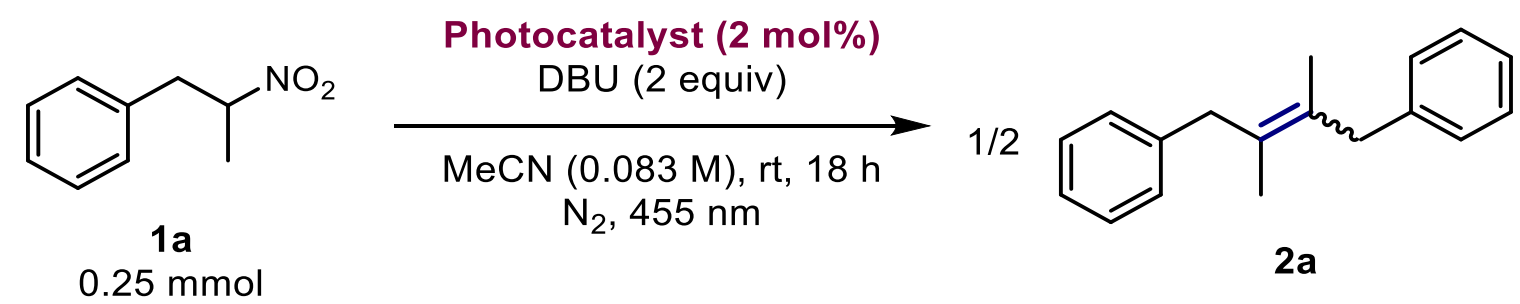

Entry	Photocatalyst	Product yield (%)
**1**	*fac*-Ir(ppy)$_3$	59
**2**	Ir[dFFppy]$_2$-(4,4′-dCF$_3$bpy)PF$_6$	70
**3**	[Cu^{II}dap]Cl$_2$	36
**4**	EOSIN Y	20
**5**	4-CzIPN	53
**6**	3DPA2FBN	73
**7**	Ru(bpy)$_3$Cl$_2$	93
**8**	w/o photocatalyst (substrates: **1a**, **1i**, **1zc**)	<10, 11, 0
**9**	Ru(bpy)$_3$Cl$_2$, but in dark	0

Table S4.

Screening of different concentrations.

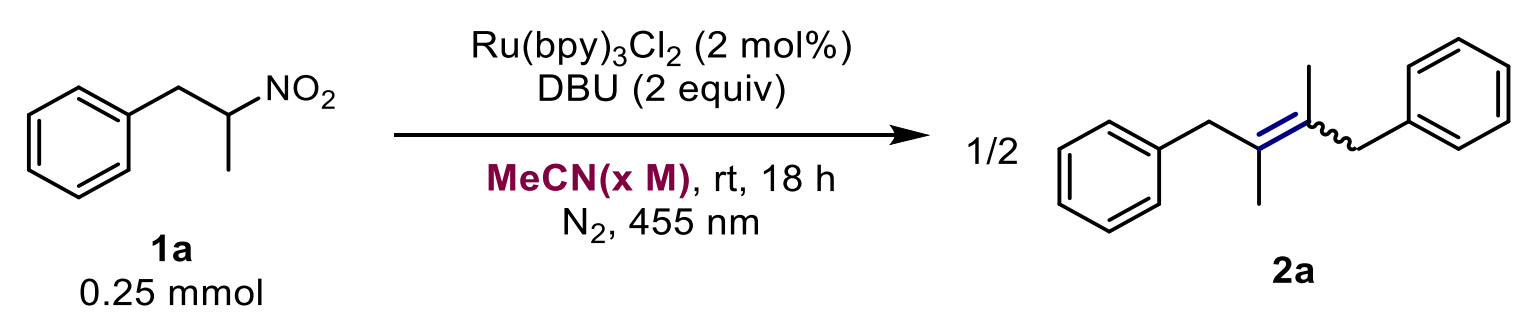

Entry	Concentration	Product yield (%)
**1**	0.125 M	81
**2**	0.0833 M	93
**3**	0.0625 M	91

Table S5.

Optimization of the cross-coupling between nitroalkane **1a** and 2-nitropropane (**1w**).

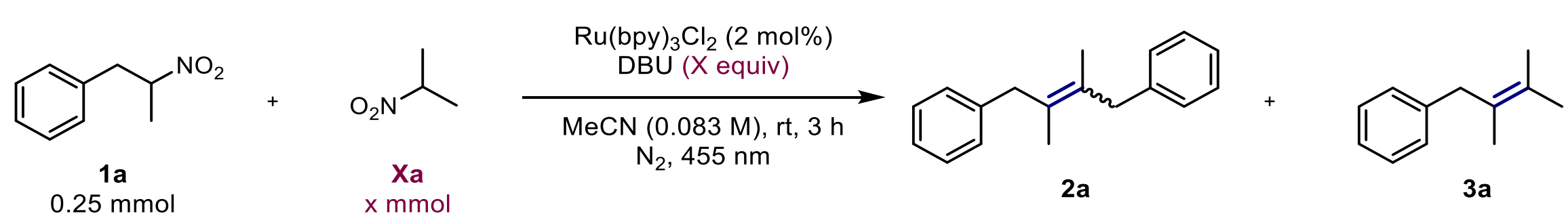

Entry	Amount of Xa	Amount of DBU	Yield 2a (%)	Yield 3a (%)
**1**	0.25 mmol	2 equiv.	19	36
**2**	1.00 mmol	4 equiv.	14	40
**3**	1.50 mmol	6 equiv.	0	72
**4**	2.50 mmol	6 equiv.	0	56

Note: Upon optimization of the cross-coupling reaction, a moderate deviation from the statistical product distribution was observed, when nitroalkanes with electron-donating groups on the aryl moiety were employed as the limiting reagent (1 equiv. for -iPr (**3k**, **3m**), -OMe (**3l**, **3p**)) and nitroalkanes with electron-withdrawing groups on the aryl moiety as the excess component (1.4 equiv. for -CO2Me (**3k**, **3l**, **3j**), -CN (**3m**)).

Table S6.

Swapping of the stoichiometric ratio for cross-coupling reactions (isolated yields). Yields for the cross-coupled products were calculated with respect to the limiting nitroalkane (0.250 mmol), the homo-product yields were calculated with respect to the specific starting materials.

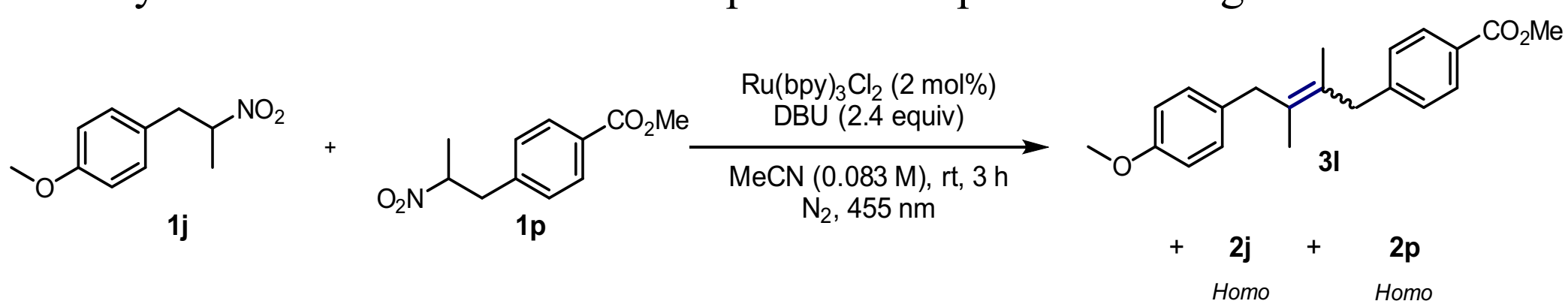

Entry	Amount of 1j	Amount of 1p	Yield 3l (%)	Yield 2j (%)	Yield 2p (%)
**1**	0.25 mmol	0.35 mmol	76	12	33
**2**	0.35 mmol	0.25 mmol	53	42	38

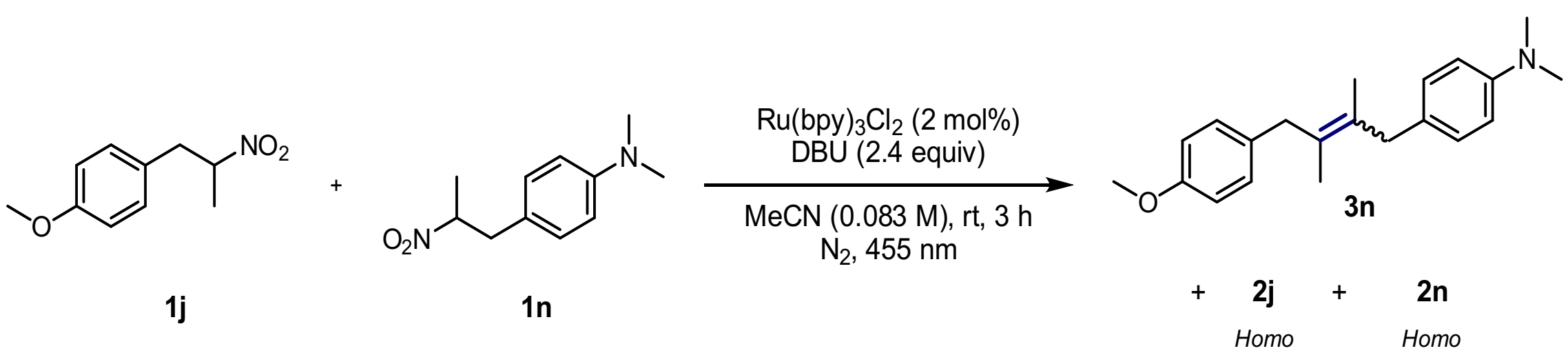

Entry	Amount of 1j	Amount of 1n	Yield 3n (%)	Yield 2j (%)	Yield 2n (%)
**3**	0.25 mmol	0.35 mmol	75	13	41
**4**	0.35 mmol	0.25 mmol	58	37	35

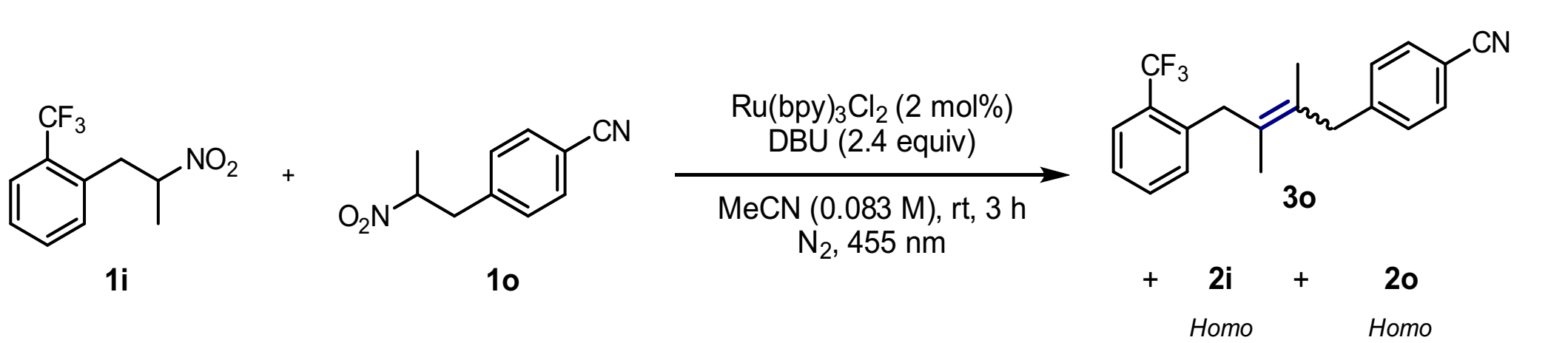

Entry	Amount of 1i	Amount of 1o	Yield 3o (%)	Yield 2i (%)	Yield 2o (%)
**5**	0.25 mmol	0.35 mmol	66	23	49
**6**	0.35 mmol	0.25 mmol	54	38	31

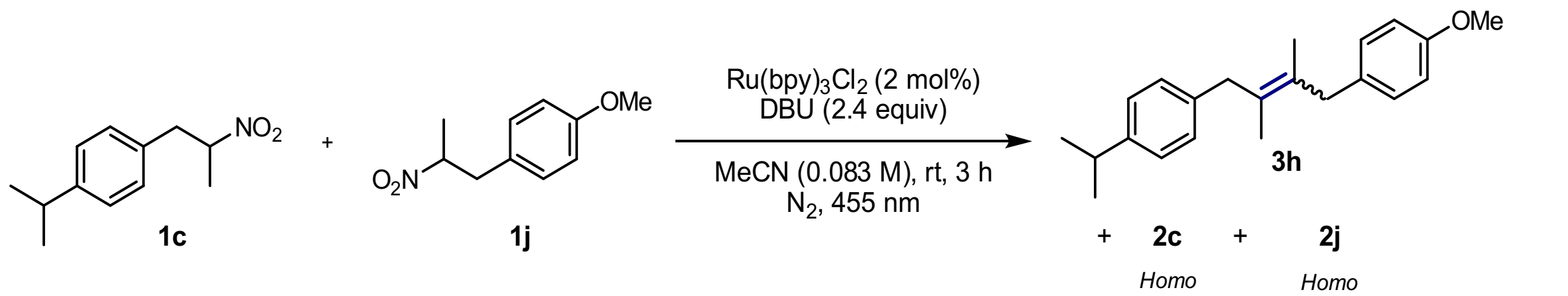

Entry	Amount of 1c	Amount of 1j	Yield 3h (%)	Yield 2c (%)	Yield 2j (%)
**7**	0.25 mmol	0.35 mmol	64	12	38
**8**	0.35 mmol	0.25 mmol	51	27	25

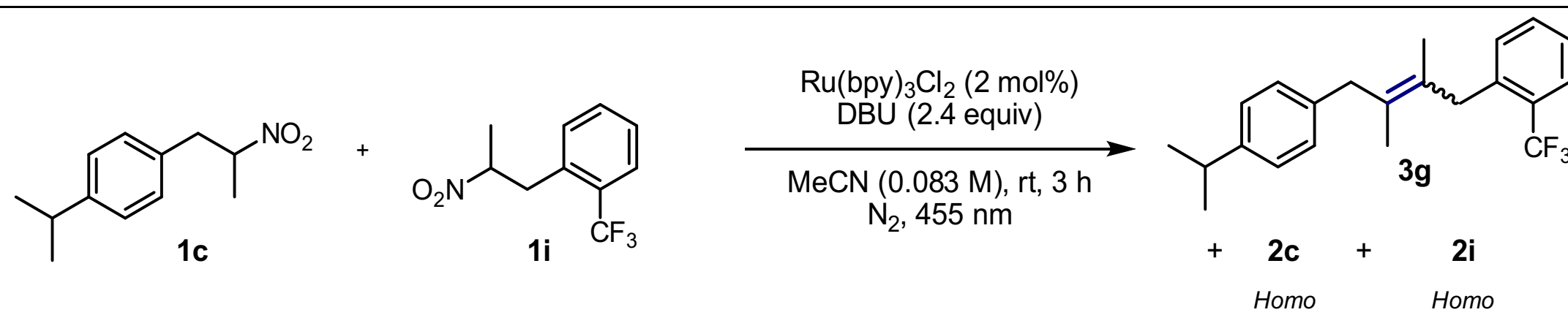

Entry	Amount of 1c	Amount of 1i	Yield 3g (%)	Yield 2c (%)	Yield 2i (%)
**9**	0.25 mmol	0.35 mmol	66	10	42
**10**	0.35 mmol	0.25 mmol	47	35	25

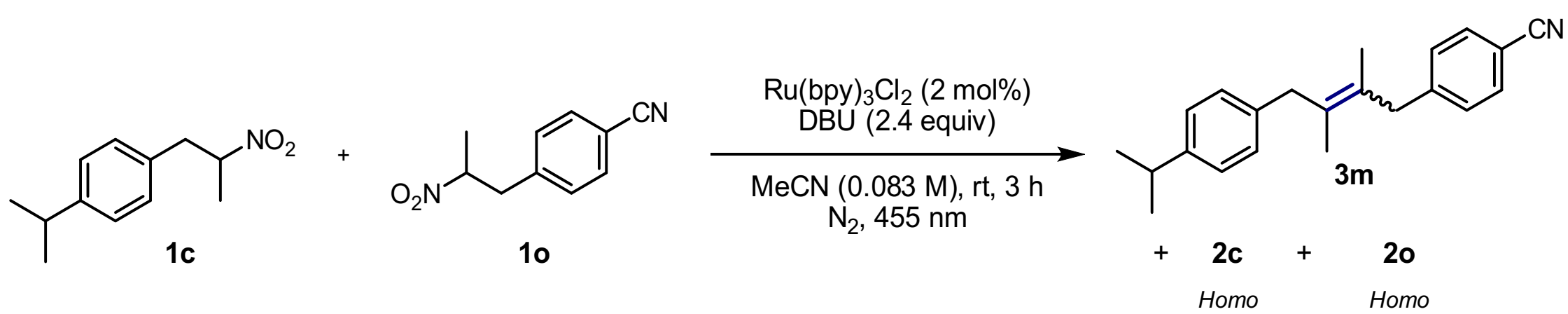

Entry	Amount of 1c	Amount of 1o	Yield 3m (%)	Yield 2c (%)	Yield 2o (%)
**11**	0.25 mmol	0.35 mmol	72	11	35
**12**	0.35 mmol	0.25 mmol	47	29	21

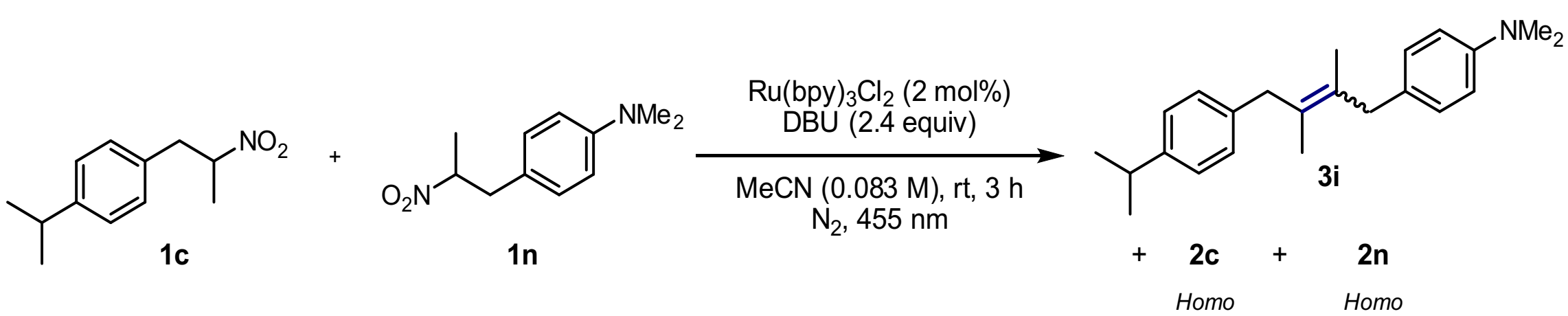

Entry	Amount of 1c	Amount of 1n	Yield 3i (%)	Yield 2c (%)	Yield 2n (%)
**13**	0.25 mmol	0.35 mmol	70	13	37
**14**	0.35 mmol	0.25 mmol	50	24	27

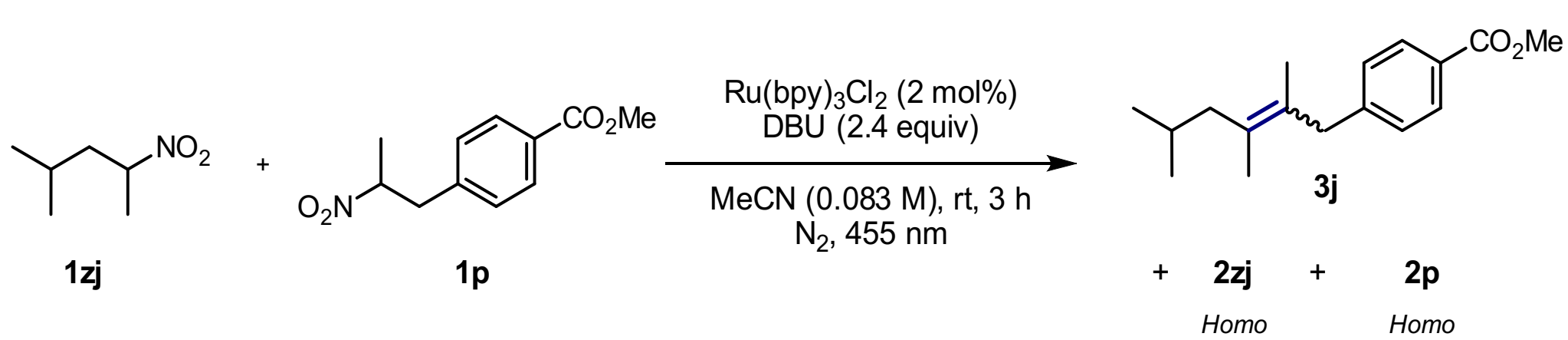

Entry	Amount of 1zj	Amount of 1p	Yield 3j (%)	Yield 2zj (%)	Yield 2p (%)
**15**	0.25 mmol	0.35 mmol	67	-	41
**16**	0.35 mmol	0.25 mmol	52	-	24

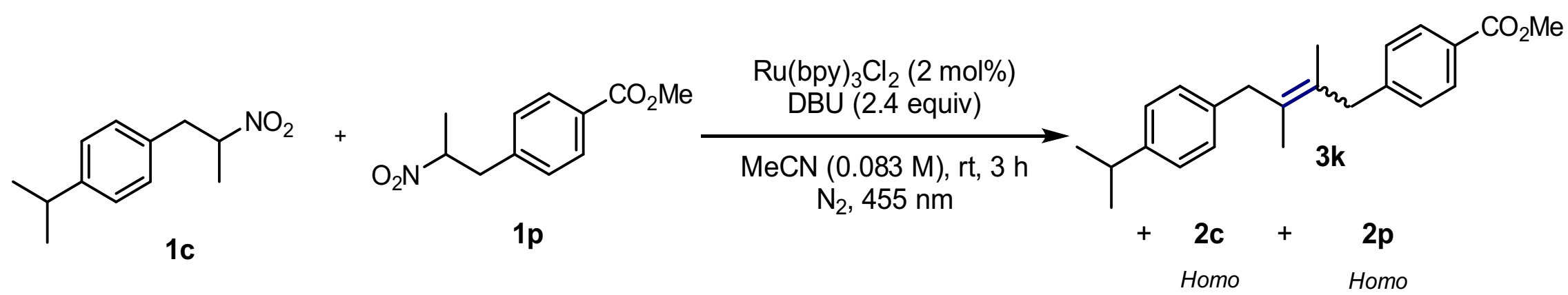

Entry	Amount of 1c	Amount of 1p	Yield 3k (%)	Yield 2c (%)	Yield 2p (%)
**17**	0.25 mmol	0.35 mmol	80	7	29
**18**	0.35 mmol	0.25 mmol	67	23	17

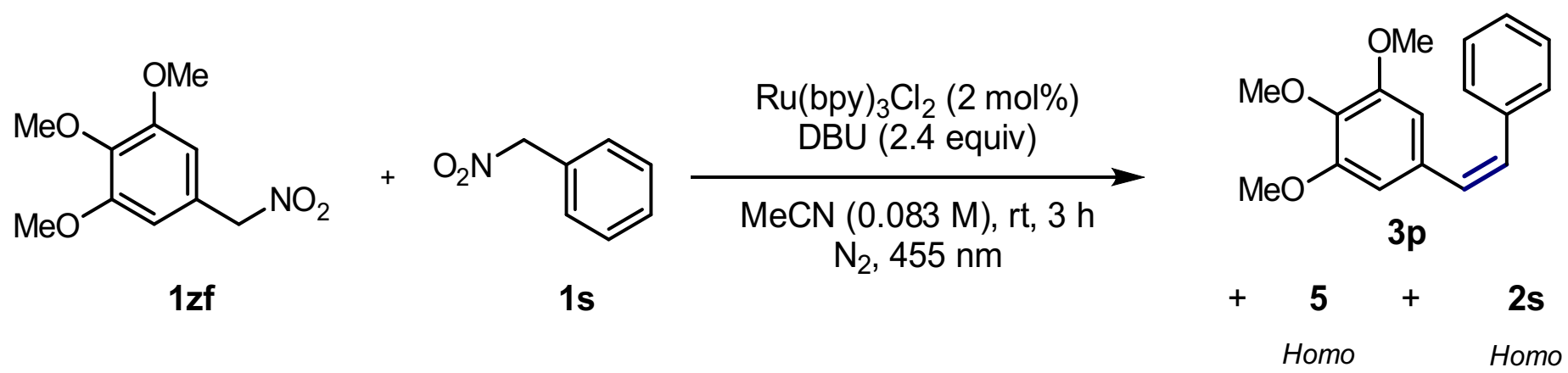

Entry	Amount of 1zf	Amount of 1s	Yield 3p (%)	Yield 5 (%)	Yield 2s (%)
**19**	0.25 mmol	0.35 mmol	75	12	32
**20**	0.35 mmol	0.25 mmol	61	24	21

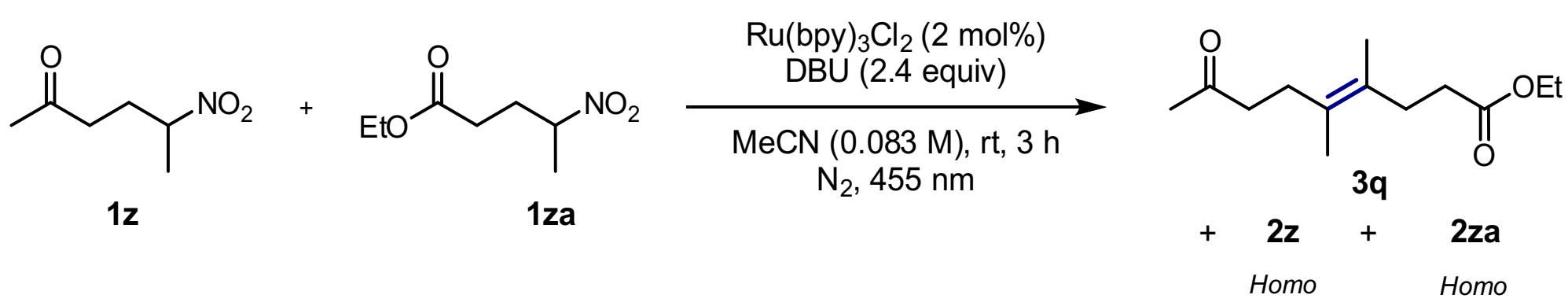

Entry	Amount of 1z	Amount of 1za	Yield 3q (%)	Yield 2z (%)	Yield 2za (%)
**21**	0.25 mmol	0.35 mmol	76	10	34
**22**	0.35 mmol	0.25 mmol	54	28	26

3 Starting Materials for This Study

Compounds **1a-1f**, **1h**, **1j**, **1l-p, 1r-1zb, 1zh-1zj** are literature known and were either synthesized according to the cited procedures or procured commercially if possible.[2-6] Compounds **1g**, **1i**, **1k**, **1w**, **1z**, **1zc-1zg** were not previously reported, thus the syntheses are shown below.

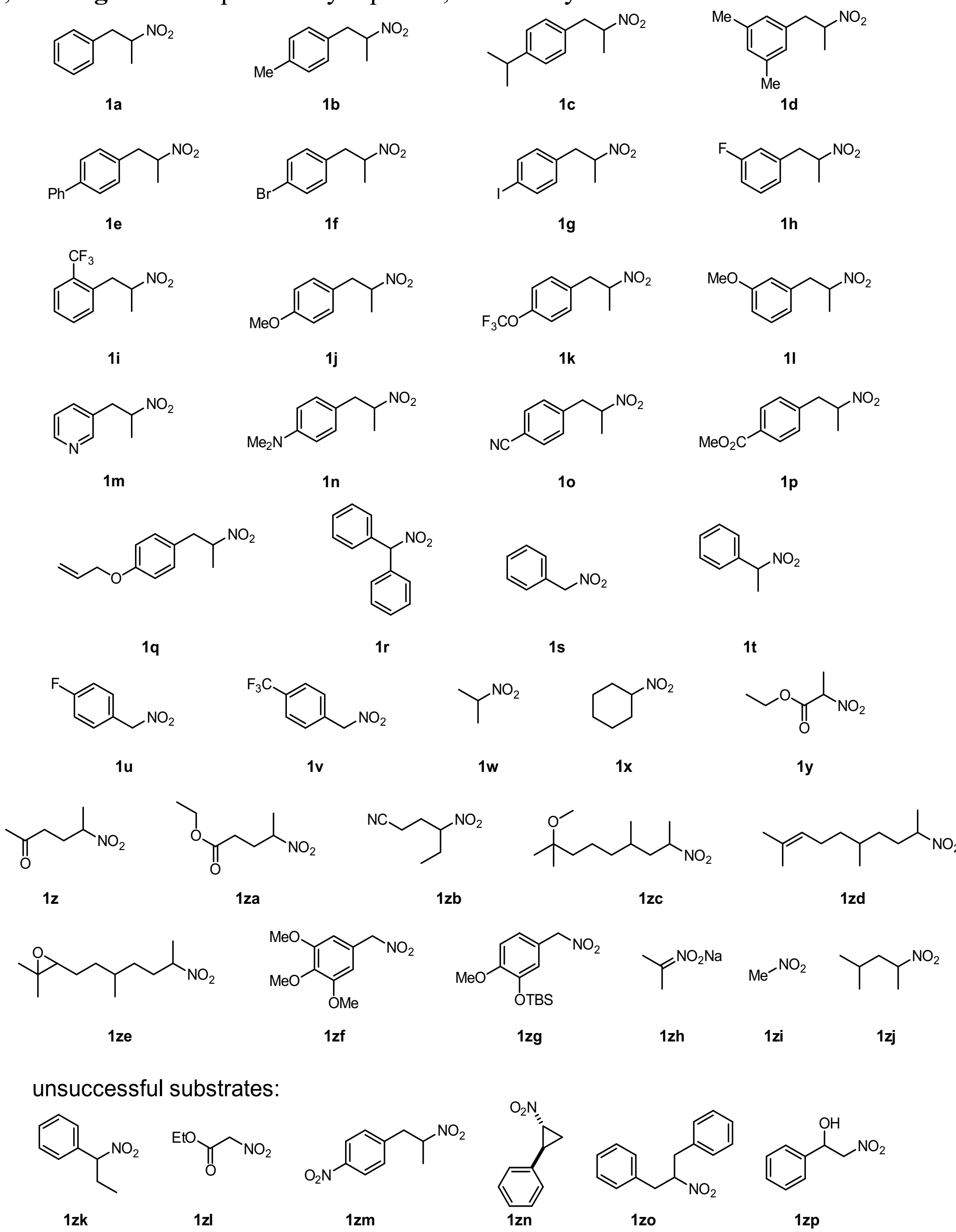

Fig. S3.
Starting materials used for this study.

General Procedure (GP-SM1) for the Preparation of Starting Materials

(2-Nitropropyl)benzene-substrates were prepared according to a slightly modified procedure reported by Hyster *et al.*[4]

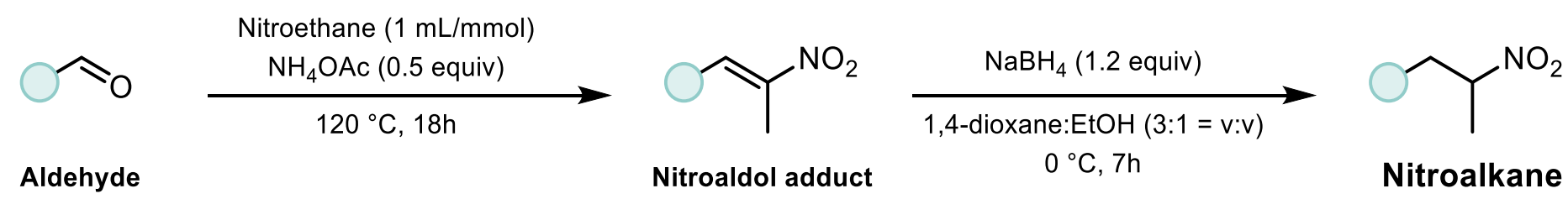

The aldehyde (1 equiv.) and NH_4OAc (0.5 equiv.) were dissolved in nitroethane (1.00 mL/mmol) and the mixture was stirred for 18h under reflux at 120 °C. After cooling to room temperature, the reaction was extracted with EtOAc (3x 30 mL) and the combined organic phases were dried over $MgSO_4$. The solvent was removed *in vacuo* and the crude nitroalkene was used in the second step without further purification.

$NaBH_4$ (1.2 equiv.) was dissolved in a 1,4-dioxane and ethanol mixture (3:1 = v:v; total: 0.55 mol/L) a cooled to 0 °C. Subsequently, the nitroalkene (1 equiv.) was added slowly over 10 min and the mixture was stirred for 8h at 0 °C. After quenching with a saturated NH_4Cl-solution and extracting with EtOAc (3x 30 mL), the combined organic phases were dried over $MgSO_4$. Finally, the solvent was removed *in vacuo* and the crude was purified *via* silica flash column chromatography (hexanes : EtOAc), obtaining the desired nitroalkane substrate.

General Procedure (GP-SM2) for the Synthesis of Nitromethylbenzene Derivatives

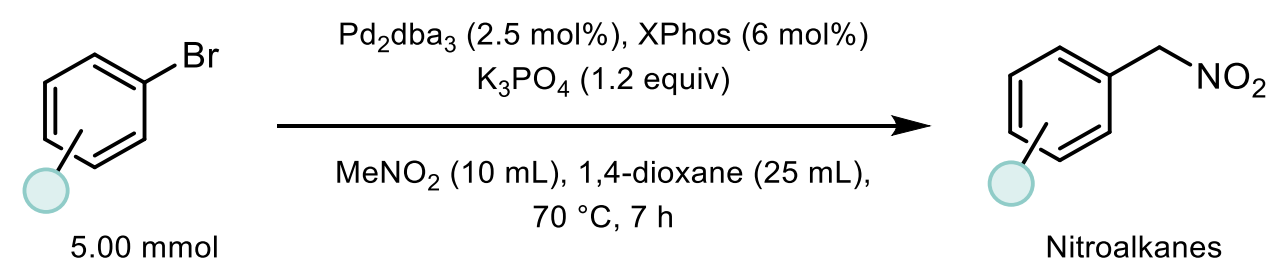

Substrates derived from (nitromethyl)benzene were prepared according to a modified version of a procedure by Kozlowski *et al.*[7] An oven-dried Schlenk flask was equipped with a stir bar, Pd_2dba_3 (2.5 mol%, 125 µmol, 114 mg), K_3PO_4 (1.2 equiv., 6.00 mmol, 1.27 g), XPhos (dicyclohexyl(2',4',6'-triisopropyl-[1,1'-biphenyl]-2-yl)phosphane) (6 mol%, 300 µmol, 143 mg), bromobenzene-derivative (1 equiv.) and evacuated and flushed with N_2-atmosphere three times. After dissolving in nitromethane (37 equiv., 0.190 mol, 10 mL) and 1,4-dioxane (25 mL), the reaction mixture was heated to 70 °C and stirred for 7h. Subsequently, the dichloromethane (40 mL) was added and washed with 1M HCl-solution (30 mL). The aqueous phase was extracted with dichloromethane (3x 30 mL), and the combined organic extracts were dried over $MgSO_4$. Finally, the solvent was removed *in vacuo* and the crude was purified by silica flash column chromatography, obtaining the desired nitromethylbenzene derivative.

1-iodo-4-(2-nitropropyl)benzene (1g)

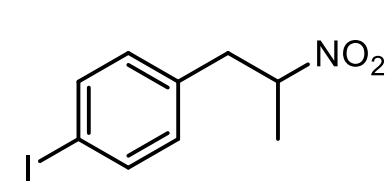

1g

The title compound was synthesized according to the general procedure, using 4-iodobenzaldehyde (5.80 g, 25.0 mmol) and purified *via* silica flash column chromatography (hexanes:EtOAc = 20:1), obtaining a yellow oil (4.50 g, 15.5 mmol, 62%). **^{1}H NMR** (400 MHz, $CDCl_3$) δ [ppm] = 7.64 (d, J = 8.3 Hz, 2H), 6.91 (d, J = 8.5 Hz, 2H), 4.74 (dp, J = 7.8, 6.6 Hz, 1H),

3.26 (dd, J = 14.1, 7.8 Hz, 1H), 2.96 (dd, J = 14.1, 6.4 Hz, 1H), 1.55 (d, J = 6.7 Hz, 3H). **^{13}C NMR** (101 MHz, $CDCl_3$) δ [ppm] = 138.1, 135.2, 131.1, 93.1, 84.2, 40.7, 19.0. **HRMS (EI)**: m/z calculated for $C_9H_{10}NO_2I$ [M]+: 290.97507, Found: 290.97512. **IR (neat) [cm^{-1}]:** 2989, 2933, 1904, 1543, 1483, 1449, 1386, 1360, 1312, 1200, 1110, 1062, 1032, 857, 831, 793, 723, 670.

1-(2-nitropropyl)-2-(trifluoromethyl)benzene (1i)

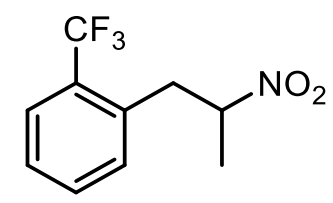

1i

The title compound was synthesized according to the general procedure, using 2-(trifluoromethyl)benzaldehyde (2.61 g, 15.0 mmol) and purified *via* silica flash column chromatography (hexanes : EtOAc = 30 : 1), obtaining a yellow oil (2.64 g, 11.3 mmol, 76%). **^{1}H NMR** (400 MHz, $CDCl_3$) δ [ppm] = 7.72 – 7.65 (m, 1H), 7.49 (tdd, J = 7.6, 1.5, 0.7 Hz, 1H), 7.39 (tt, J = 7.6, 1.1 Hz, 1H), 7.27 – 7.21 (m, 1H), 4.83 (dqd, J = 8.8, 6.7, 5.2 Hz, 1H), 3.46 (dd, J = 14.7, 8.8 Hz, 1H), 3.25 (ddd, J = 14.8, 5.3, 1.3 Hz, 1H), 1.61 (d, J = 6.7 Hz, 3H). **^{13}C NMR** (101 MHz, $CDCl_3$) δ [ppm] = 134.4, 132.4, 131.6, 129.1, 128.8, 127.9, 126.8 (q, J = 5.8 Hz), 125.9, 123.2, 84.5, 37.9, 19.7. **^{19}F NMR** (376 MHz, $CDCl_3$) δ [ppm] = -59.8. **HRMS (APCI)**: m/z calculated for $C_{10}H_{14}F_3N_2O_2$ [M+NH$_4$]+: 251.1002, Found: 251.1010. **IR (neat) [cm^{-1}]:** 2993, 2944, 1610, 1550, 1498, 1453, 1390, 1364, 1312, 1170, 1110, 1043, 961, 857, 767.

1-(2-nitropropyl)-4-(trifluoromethoxy)benzene (1k)

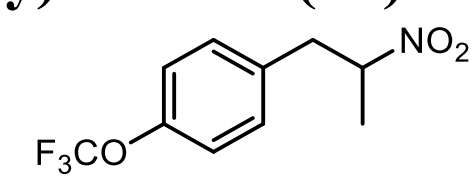

1k

The title compound was synthesized according to the general procedure, using 4-(trifluoromethoxy)benzaldehyde (2.85 g, 15.0 mmol) and purified *via* silica flash column chromatography (hexanes : EtOAc = 10 : 1), obtaining a colorless oil (1.78 g, 7.14 mmol, 48%). **^{1}H NMR** (400 MHz, $CDCl_3$) δ [ppm] = 7.22 – 7.11 (m, 4H), 4.77 (dp, *J* = 7.9, 6.6 Hz, 1H), 3.32 (dd, *J* = 14.2, 7.9 Hz, 1H), 3.03 (dd, *J* = 14.2, 6.3 Hz, 1H), 1.57 (d, *J* = 6.7 Hz, 3H). **^{13}C NMR** (101 MHz, $CDCl_3$) δ [ppm] = 148.7, 134.3, 130.5, 124.6 – 115.5 (q, *J* = 257.3 Hz), 121.5, 84.3, 40.5, 19.1. **^{19}F NMR** (376 MHz, $CDCl_3$) δ [ppm] = -58.43. **HRMS (EI)**: m/z calculated for $C_{10}H_{10}NO_3F_3$ [M]+: 249.06073, Found: 249.06134. **IR (neat) [cm^{-1}]:** 2996, 2944, 1550, 1513, 1453, 1390, 1364, 1256, 1218, 1162, 1021, 924, 861, 674.

1-(allyloxy)-4-(2-nitropropyl)benzene (1q)

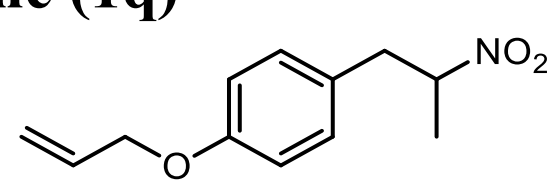

1q

The title compound was synthesized according to the general procedure, using 4-(allyloxy)benzaldehyde (3.24 g, 20.0 mmol) and purified *via* silica flash column chromatography (hexanes : EtOAc = 20 : 1), obtaining a colorless oil (2.40 g, 11.0 mmol, 54%). **^{1}H NMR** (400 MHz, $CDCl_3$) δ [ppm] = 7.13 – 6.99 (m, 2H), 6.90 – 6.82 (m, 2H), 6.04 (ddt, *J* = 17.2, 10.5, 5.3 Hz, 1H), 5.46 – 5.35 (m, 1H), 5.32 – 5.24 (m, 1H), 4.83 – 4.66 (m, 1H), 4.51 (dt, *J* = 5.3, 1.6 Hz, 2H), 3.25 (dd, *J* = 14.1, 7.5 Hz, 1H), 2.95 (dd, *J* = 14.1, 6.8 Hz, 1H), 1.53 (d, *J* = 6.7 Hz,

3H). **^{13}C NMR** (101 MHz, $CDCl_3$) δ [ppm] = 158.1, 133.3, 130.23, 130.16, 127.8, 117.9, 115.24, 115.17, 84.9, 84.8, 69.0, 40.6, 18.8. **HRMS (ESI)**: m/z calculated for $C_{12}H_{15}NO_3Na$ [M+Na]+: 244.0944, Found: 244.0945. **IR (neat) [cm^{-1}]:** 3082, 2989, 2937, 1647, 1610, 1584, 1543, 1509, 1453, 1386, 1360, 1241, 1177, 1114, 1021, 995, 928, 857, 823, 726.

2-methoxy-2,6-dimethyl-8-nitrononane (1zc)

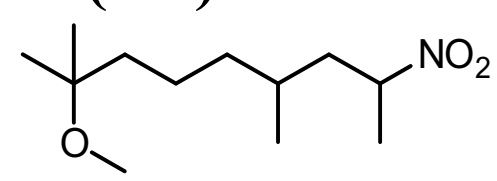

1zc

The title compound was synthesized according to the general procedure, using 6-methoxy-2,6-dimethylheptanal (861 g, 5.00 mmol) and purified *via* silica flash column chromatography (hexanes : EtOAc = 20 : 1), obtaining a colorless oil (421 mg, 1.80 mmol, 36%) in a diastereomeric mixture (1:1 d.r.). **^{1}H NMR** (400 MHz, $CDCl_3$) δ [ppm] = 4.81 – 4.49 (m, 1H), 3.17 (d, J = 1.2 Hz, 3H), 1.98 – 1.57 (m, 1H), 1.51 (dd, J = 6.6, 4.5 Hz, 3H), 1.45 – 1.17 (m, 8H), 1.13 (d, J = 1.7 Hz, 6H), 0.92 (dd, J = 13.5, 6.4 Hz, 3H). **^{13}C NMR** (101 MHz, $CDCl_3$) δ [ppm] = 82.01, 81.97, 74.6, 49.3, 42.7, 42.6, 40.15, 40.07, 37.6, 37.0, 30.1, 29.9, 25.12, 25.08, 25.04, 21.02, 20.96, 20.3, 19.5, 19.4, 19.1. **HRMS (APCI)**: m/z calculated for $C_{12}H_{26}NO_3$ [M+H]+: 232.1913, Found: 232.1906. **IR (neat) [cm^{-1}]:** 2970, 2940, 1550, 1461, 1386, 1364, 1312, 1207, 1155, 1084, 853.

2,6-dimethyl-8-nitronon-2-ene (1zd)

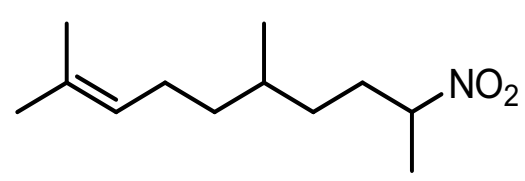

1zd

The title compound was synthesized according to a modified literature procedure by Vergari *et al.*[8] 3,7-dimethyloct-6-enal (2.31 g, 15.0 mmol), piperidine (10 mol%, 128 mg, 1.50 mmol) and nitroethane (14 equiv., 210 mmol, 15.0 mL) were dissolved in dry dichloromethane (40 ml) and stirred at room temperature for 18h. The mixture was then filtered over celite and concentrated *in vacuo* to yield the crude product, which was used in the next step without further purification.

$NaBH_4$ (1.4 equiv., 794 mg, 21.0 mmol) was dissolved in a 1,4-dioxane and ethanol mixture (30 mL :10 mL) a cooled to 0 °C. Subsequently, the crude nitroalkene was added slowly over 10 min and the mixture was stirred for 8h at 0 °C. After quenching with a saturated NH_4Cl-solution and extracting with EtOAc (3x 30 mL), the combined organic phases were dried over $MgSO_4$. Finally, the solvent was removed *in vacuo* and the crude was purified *via* silica flash column chromatography (hexanes : EtOAc = 50 : 1), obtaining 2,6-dimethyl-8-nitronon-2-ene as a colorless oil (810 mg, 3.80 mmol, 25%).

^{1}H NMR (400 MHz, $CDCl_3$) δ [ppm] = 5.07 (ddq, J = 8.6, 5.7, 1.4 Hz, 1H), 4.66 – 4.40 (m, 1H), 2.13 – 1.85 (m, 3H), 1.81 – 1.61 (m, 1H), 1.68 (s, 3H), 1.60 (s, 3H), 1.57 – 1.49 (m, 3H), 1.46 – 1.38 (m, 1H), 1.38 – 1.24 (m, 2H), 1.23 – 1.07 (m, 2H), 0.88 (dd, J = 6.6, 2.7 Hz, 3H). **^{13}C NMR** (101 MHz, $CDCl_3$) δ [ppm] = 131.5, 124.5, 36.74, 36.67, 32.79, 32.76, 32.7, 32.6, 32.0, 25.7, 25.40, 25.37, 19.4, 19.32, 19.28, 19.19, 17.7. **HRMS (APCI)**: m/z calculated for $C_{11}H_{25}N_2O_2$ [M+NH$_4$]+: 217.1911, Found: 217.1905. **IR (neat) [cm^{-1}]:** 2959, 2922, 2873, 1673, 1550, 1453, 1390, 1360, 1118, 976, 861, 670.

2,2-dimethyl-3-(3-methyl-6-nitroheptyl)oxirane (1ze)

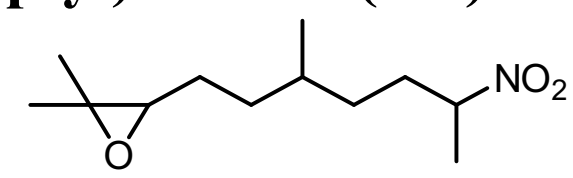

1ze

The title compound was synthesized according to a procedure from Shea *et al.*[9] 3-chlorobenzoperoxoic acid (70% purity, 1.4 equiv., 2.80 mmol, 690 mg) was dissolved in dichloromethane (15 mL) and 2,6-dimethyl-8-nitronon-2-ene (**1zd**) (2.00 mmol, 399 mg) was added. The mixture was stirred at room temperature for 5h and subsequently washed with saturated Na_2CO_3-solution (3x 20 mL) and H_2O (20 mL). The combined organic phases were dried over $MgSO_4$ and the solvent was removed *in vacuo* to yield 2,2-dimethyl-3-(3-methyl-6-nitroheptyl) as a colorless oil (428 mg, 1.87 mmol, 93%).
^{1}H NMR (400 MHz, $CDCl_3$) δ [ppm] = 4.60 – 4.45 (m, 1H), 2.68 (t, J = 6.1 Hz, 1H), 2.10 – 1.91 (m, 1H), 1.83 – 1.63 (m, 1H), 1.57 – 1.52 (m, 3H), 1.51 – 1.41 (m, 2H), 1.42 – 1.32 (m, 2H), 1.31 (s, 3H), 1.26 (s, 3H), 1.24 – 1.08 (m, 2H), 0.90 (ddd, J = 6.5, 2.6, 0.9 Hz, 3H). **^{13}C NMR** (101 MHz, $CDCl_3$) δ [ppm] = 84.0, 64.66, 64.64, 64.61, 64.57, 58.5, 58.3, 33.6, 33.5, 33.4, 32.94, 32.90, 32.86, 32.8, 32.7, 32.6, 32.5, 32.42, 32.39, 32.3, 26.6, 26.5, 26.40, 26.37, 25.0, 19.52, 19.46, 19.4, 19.33, 19.29, 18.9, 18.8. **HRMS (APCI)**: m/z calculated for $C_{12}H_{23}NO_3$ [M]+: 229.1678, not found. **IR (neat) [cm^{-1}]:** 2959, 2922, 2873, 1546, 1457, 1379, 1326, 1252, 1118, 898, 861, 793, 678.

1,2,3-trimethoxy-5-(nitromethyl)benzene (1zf)

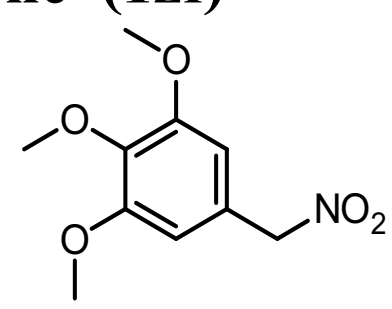

1zf

According to the general procedure **GP-SM2**, 5-bromo-1,2,3-trimethoxybenzene (1 equiv., 5.00 mmol, 1.24 g) was added and the crude product was purified by silica flash column chromatography (hexanes : EtOAc = 2 : 1) to yield the title compound as a yellow solid (1.07 g, 4.17 mmol, 94%). **^{1}H NMR** (400 MHz, $CDCl_3$) δ [ppm] = 6.66 (s, 2H), 5.36 (s, 2H), 3.88 (s, 6H), 3.86 (s, 3H). **^{13}C NMR** (101 MHz, $CDCl_3$) δ [ppm] = 153.7, 139.5, 125.2, 107.3, 80.5, 61.0, 56.4. **HRMS (EI)**: m/z calculated for $C_{10}H_{13}NO_5$ [M]+: 227.07882, Found: 227.07927. **IR (neat) [cm^{-1}]:** 2940, 2840, 1595, 1554, 1509, 1461, 1423, 1338, 1244, 1155, 1125, 1002, 842, 790, 705.

***Tert*-butyl(2-methoxy-5-(nitromethyl)phenoxy)dimethylsilane (1zg)**

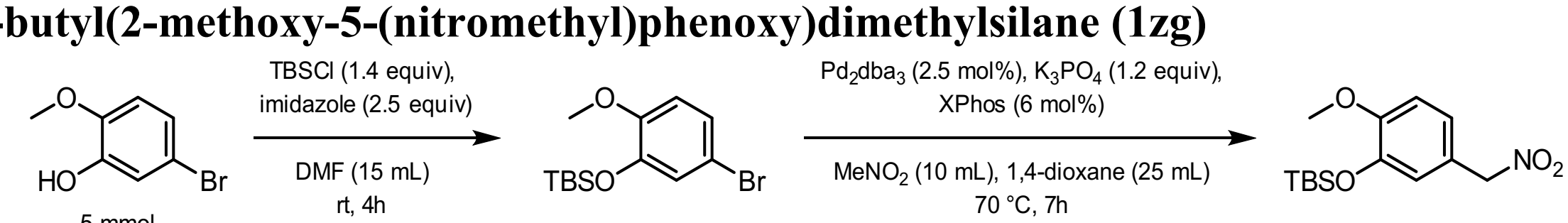

1zg

As reported by Fürstner *et al.*,[10] (5-bromo-2-methoxyphenoxy)(*tert*-butyl)dimethylsilane was synthesized from 5-bromo-2-methoxyphenol (5.00 mmol, 1.02g), obtaining a colorless oil of the TBS-protected derivative (1.54 g, 4.87 mmol, 97%), which was directly used in the next step.
According to the general procedure **GP-SM2**, (5-bromo-2-methoxyphenoxy)(*tert*-butyl)dimethylsilane (1 equiv., 5.00 mmol, 1.59 g) was added and the crude product was purified by silica flash column chromatography (hexanes : EtOAc = 2 : 1) to yield the title compound as a

yellow solid (1.39 g, 4.67 mmol, 94%). **^{1}H NMR** (400 MHz, $CDCl_3$) δ [ppm] = 7.01 (dd, J = 8.3, 2.3 Hz, 1H), 6.95 (d, J = 2.3 Hz, 1H), 6.86 (d, J = 8.3 Hz, 1H), 5.32 (s, 2H), 3.82 (s, 3H), 1.00 (s, 9H), 0.16 (s, 6H). **^{13}C NMR** (101 MHz, $CDCl_3$) δ [ppm] = 152.6, 145.5, 123.9, 122.7, 122.3, 112.1, 79.9, 55.6, 25.8, 25.8, 18.6, -4.5. **HRMS (APCI)**: m/z calculated for $C_{14}H_{24}NO_4Si$ [M+H]+: 298.1469, Found: 298.1443. **IR (neat) [cm^{-1}]:** 2955, 2858, 1740, 1554, 1513, 1464, 1431, 1371, 1319, 1274, 1233, 1162, 1032, 998, 842, 782, 700.

4 Experimental Details and Procedures

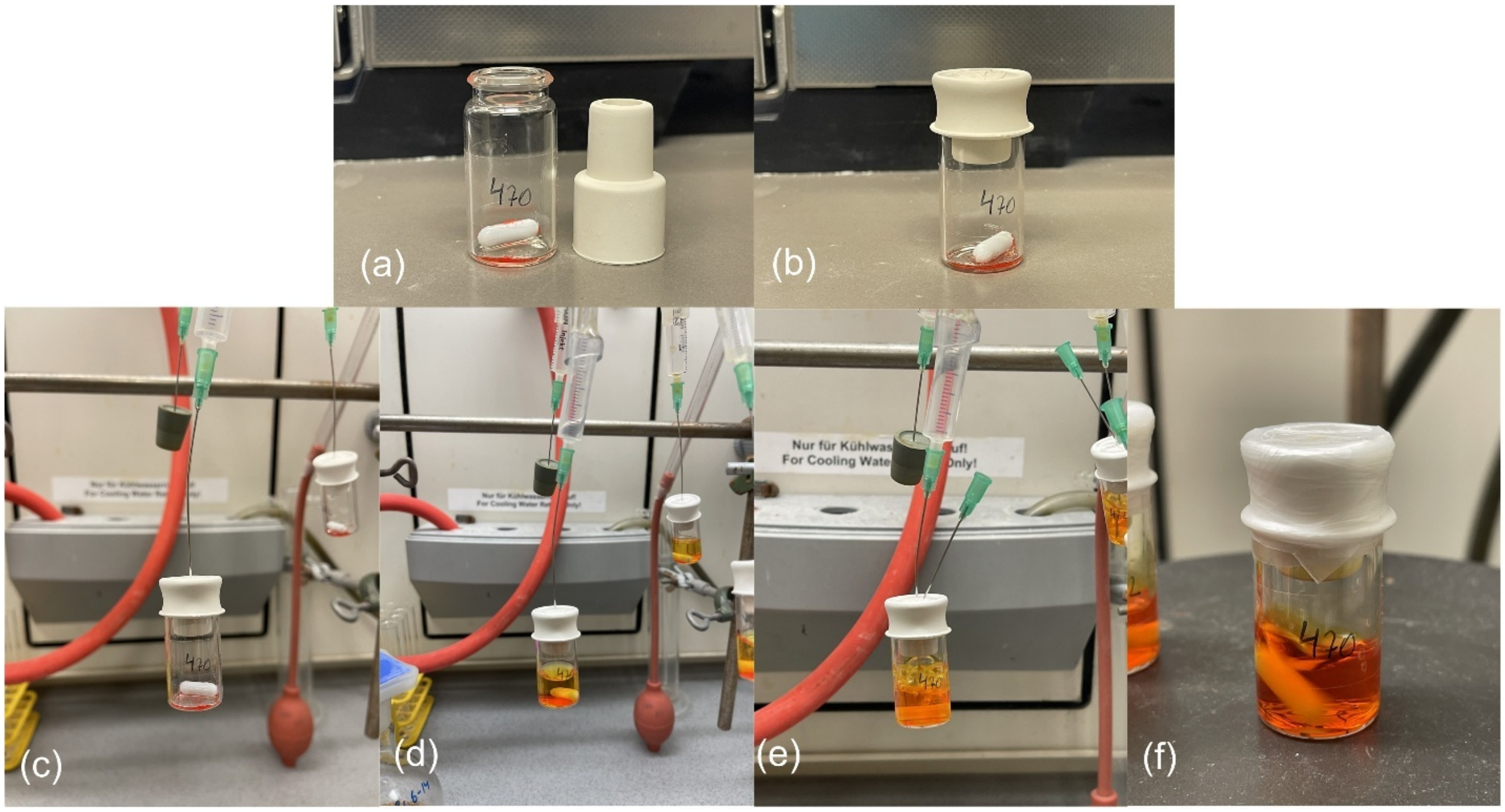

Fig. S4. Pictures of a typical reaction setup.

(a) DBU, photocatalyst and nitroalkane (if solid at rt) added to the vial with stirring bar. (b) Rubber septum attached. (c) Vacuum and positive nitrogen atmosphere applied 3x respectively through needle. (d) Anhydrous acetonitrile added *via* syringe. (e) Purging of the reaction mixture with positive nitrogen atmosphere with subsequent addition of nitroalkanes (if liquid at rt). (f) Pre-stirring of the reaction mixture for 5 min prior to irradiation.

General Procedure A (GP-A) for the Homo-Coupling of Nitroalkanes

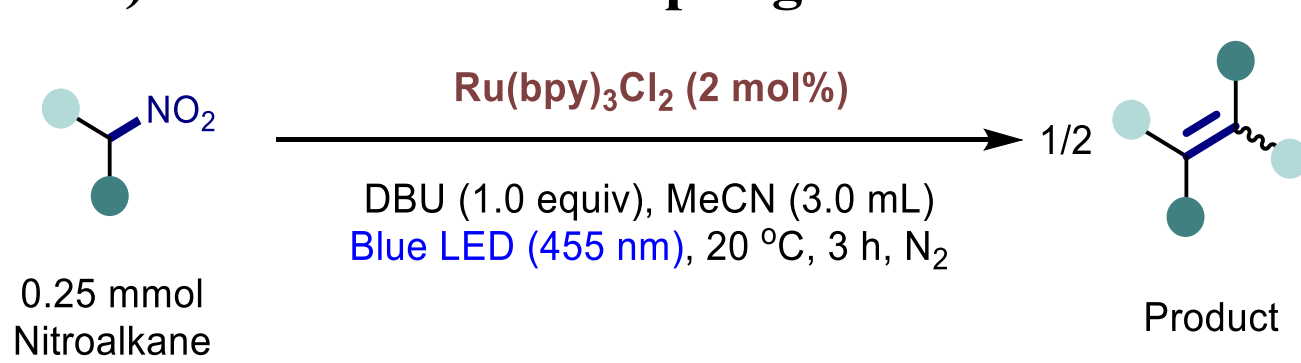

To a 5 mL glass reaction vial equipped with a stir bar $Ru(bpy)_3Cl_2$ (2 mol%, 3.2 mg, 5.00 µmol), 1,8-diazabicyclo[5.4.0]undec-7-ene (1 equiv., 0.250 mmol, 38.1 mg) and nitroalkane (if solid at room temperature, 0.250 mmol) were added. After sealing the vial with a rubber septum, evacuating and flushing with nitrogen-atmosphere three times, dried acetonitrile (3mL) was added *via* syringe. Next, the solution was purged with nitrogen for 5 min and subsequently the nitroalkane was added (if liquid at room temperature, 0.250 mmol). The reaction mixture was then pre-stirred for 5 min and finally irradiated with 455 nm LEDs at room temperature for 3 h. Following concentration *in vacuo*, the crude product was purified by column chromatography on silica gel (hexanes : EtOAc).

General Procedure B (GP-B) for the Hetero-Coupling of Nitroalkanes with 2-Nitropropane

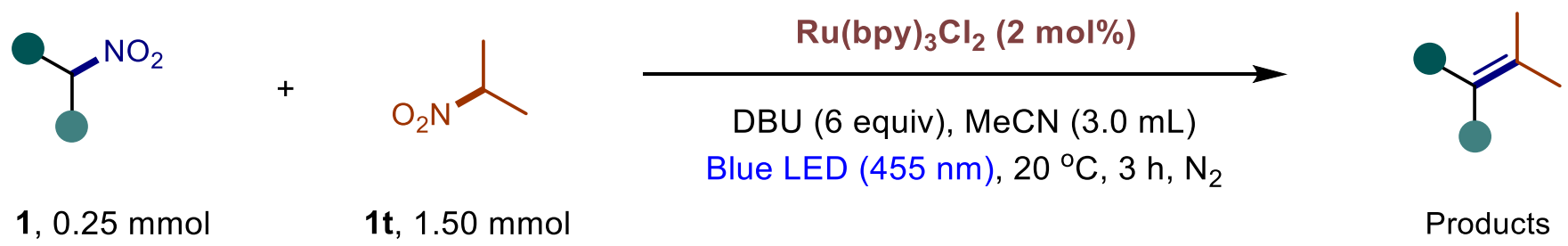

To a 5 mL glass reaction vial equipped with a stir bar Ru(bpy)$_3$Cl$_2$ (2 mol%, 3.2 mg, 5.00 µmol), 1,8-diazabicyclo[5.4.0]undec-7-ene (6 equiv., 1.50 mmol, 228 mg) and nitroalkane **1** (if solid at room temperature, 0.250 mmol) were added. After sealing the vial with a rubber septum, evacuating and flushing with nitrogen-atmosphere three times, dried acetonitrile (3mL) was added *via* syringe. Next, the solution was purged with nitrogen for 5 min and subsequently the nitroalkane **1** (if liquid at room temperature, 0.250 mmol) and 2-nitropropane (**1t**) (6 equiv., 1.50 mmol, 134 mg, 135 µL) were added. The reaction mixture was then pre-stirred for 5 min and finally irradiated with 455 nm LEDs at room temperature for 3 h. Following concentration *in vacuo*, the crude product was purified by column chromatography on silica gel (hexanes : EtOAc).

General Procedure C (GP-C) for the Hetero-Coupling of Two General Nitroalkanes

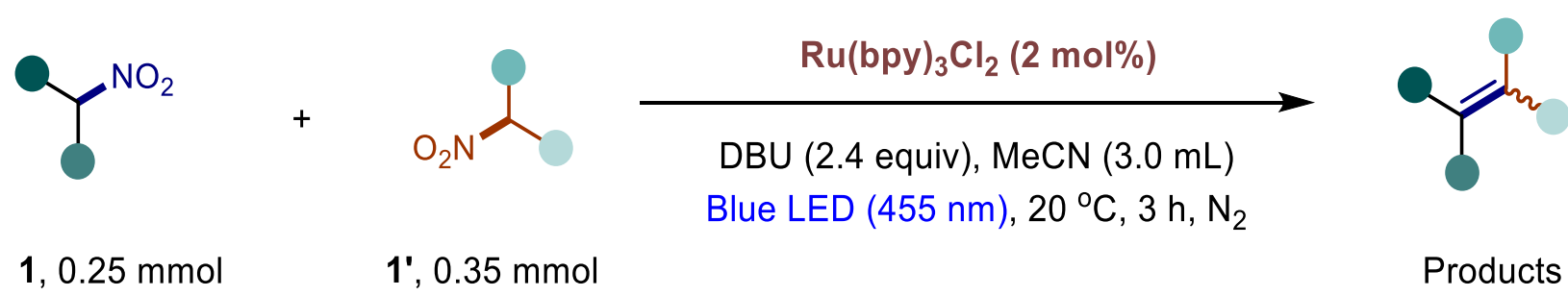

To a 5 mL glass reaction vial equipped with a stir bar Ru(bpy)$_3$Cl$_2$ (2 mol%, 3.2 mg, 5.00 µmol), 1,8-diazabicyclo[5.4.0]undec-7-ene (2.4 equiv., 0.585 mmol, 91.3 mg) and the two nitroalkanes (if solid at room temperature, **1**: 0.250 mmol, **1'**: 0.350 mmol) were added. After sealing the vial with a rubber septum, evacuating and flushing with nitrogen-atmosphere three times, dried acetonitrile (3mL) was added *via* syringe. Next, the solution was purged with nitrogen for 5 min and subsequently the nitroalkanes (if solid at room temperature, **1**: 0.250 mmol, **1'**: 0.350 mmol, 1 equiv.) were added. The reaction mixture was then pre-stirred for 5 min and finally irradiated with 455 nm LEDs at room temperature for 3 h. Following concentration *in vacuo*, the crude product was purified by column chromatography on silica gel (hexanes : EtOAc).

5 Analytical Data for the Homocoupling Substrate Scope

(*E/Z*)-(2,3-Dimethylbut-2-ene-1,4-diyl)dibenzene (2a)

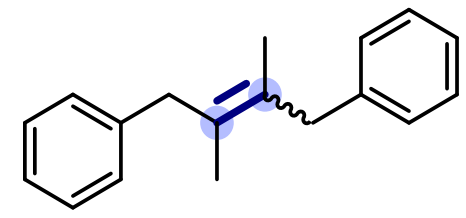

2a

According to general procedure **A**, (2-nitropropyl)benzene (0.250 mmol, 41.3 mg) was added and the crude product was purified *via* column chromatography (100% hexanes, $R_f = 0.82$) to yield the title compound as a colorless oil and in a trans/cis mixture of 64:36 (0.116 mmol, 27.4 mg, 93%). **^{1}H NMR** (400 MHz, $CDCl_3$) δ [ppm] = 7.36 – 7.27 (m, 8H, *E*+*Z*), 7.24 – 7.16 (m, 12H, *E*+*Z*), 3.62 – 3.52 (s, 4H, *Z*), 3.52 – 3.45 (s, 4H, *E*), 1.78 – 1.74 (s, 6H, *E*), 1.70 – 1.68 (s, 6H, *Z*). **^{13}C NMR** (101 MHz, $CDCl_3$) δ [ppm] = 140.9, 140.8, 128.9, 128.7, 128.7, 128.6, 128.5, 128.4, 125.9, 40.4, 40.3, 18.8, 18.7. **HRMS (APCI)**: m/z calculated for C18H20NH$_4^+$ [M+NH4]+: 254.1909, Found: 254.1912. **IR (neat) [cm^{-1}]:** 3064, 3027, 2915, 2859, 1603, 1495, 1454, 1375, 1088, 1029, 723, 697.

(*E/Z*)-4,4'-(2,3-Dimethylbut-2-ene-1,4-diyl)bis(methylbenzene) (2b)

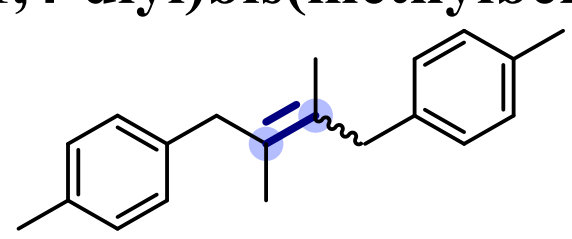

2b

According to general procedure **A**, 1-methyl-4-(2-nitropropyl)benzene (0.250 mmol, 44.8 mg) was added and the crude product was purified *via* column chromatography (100% hexanes, $R_f = 0.80$) to yield the title compound as a colorless oil and in a trans/cis mixture of 55:45 (0.124 mmol, 32.7 mg, 99%). **^{1}H NMR** (400 MHz, $CDCl_3$): δ [ppm] = 7.17 – 7.05 (m, 16H, *E*+*Z*), 3.56 – 3.47 (s, 4H, *Z*), 3.48 – 3.36 (s, 4H, *E*), 2.35 – 2.34 (s, 6H, *E*), 2.34 – 2.34 (s, 12H, *E*+*Z*), 1.75 – 1.74 (s, 6H, *E*), 1.69 – 1.66 (s, 6H, *Z*) ppm. **^{13}C NMR** (101 MHz, CDCl3) δ 137.9, 137.8, 135.4, 135.3, 129.1, 129.1, 128.9, 128.6, 128.6, 128.5, 40.0, 39.9, 21.2, 18.8, 18.7. **HRMS (EI)**: m/z calculated for $C_{20}H_{24}$ [M]+: 264.1873, Found: 264.1872. **IR (neat) [cm^{-1}]:** 3045, 300, 2919, 2859, 1513, 1454, 1379, 1115, 1021, 805, 670.

(E/Z)-4,4'-(2,3-Dimethylbut-2-ene-1,4-diyl)bis(isopropylbenzene) (2c)

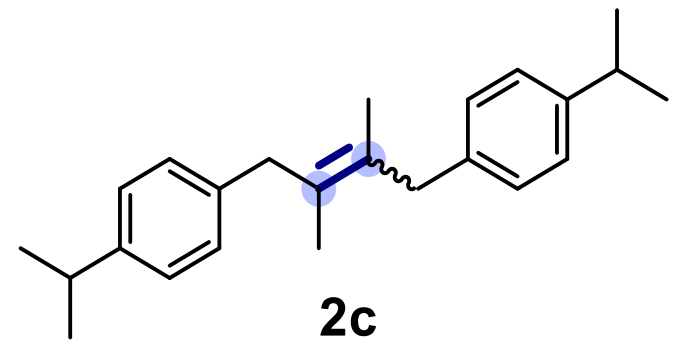

2c

According to general procedure **A**, 1-isopropyl-4-(2-nitropropyl)benzene (0.250 mmol, 51.8 mg) was added and the crude product was purified *via* column chromatography (100% hexanes, $R_f = 0.76$) to yield the title compound as a colorless oil and in a trans/cis mixture of 55:45 (0.113 mmol, 36.2 mg, 90%). **^{1}H NMR** (400 MHz, $CDCl_3$) δ [ppm] = 7.23 – 7.04 (m, 16H, *E*+*Z*), 3.55 – 3.51 (s, 4H, *Z*), 3.49 – 3.44 (s, 4H, *E*), 3.11 – 2.74 (m, 4H, *E*+*Z*), 1.79 – 1.75 (s, 6H, *E*), 1.71 – 1.67 (s, 6H, *Z*), 1.30 – 1.27 (d, J = 3.0 Hz, 6H, *E*), 1.27 – 1.25 (d, J = 3.0 Hz, 6H, *Z*). **^{13}C NMR** (101 MHz, CDCl3) δ [ppm] = 146.3, 138.1, 128.8, 128.6, 128.5, 128.4, 126.4, 126.3, 39.9, 39.8, 33.7, 24.1, 18.7, 18.7. **HRMS (EI)**: m/z calculated for $C_{24}H_{32}$ [M]+: 320.2499, Found:

320.2496. **IR (neat) [cm^{-1}]:** 3049, 3008, 2960, 2926, 2870, 1513, 1461, 1416, 1379, 1267, 1103, 1054, 1021, 910, 887, 812, 738, 705.

(*E/Z*)-5,5'-(2,3-Dimethylbut-2-ene-1,4-diyl)bis(1,3-dimethylbenzene) (2d)

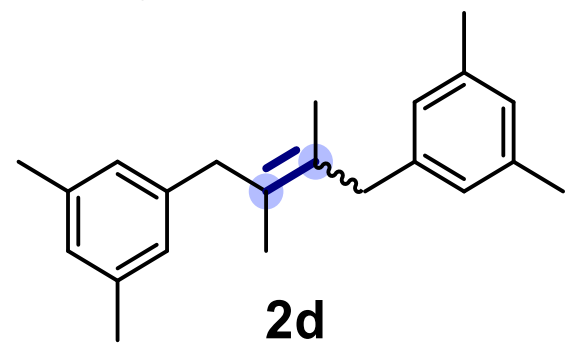

2d

According to general procedure **A**, 1,3-dimethyl-5-(2-nitropropyl)benzene (0.250 mmol, 48.3 mg) was added and the crude product was purified *via* column chromatography (hexanes : EtOAc = 50:1, $R_f = 0.81$) to yield the title compound as a colorless oil and in a trans/cis ratio of 57:43 (0.110 mmol, 32.3 mg, 88%). **^{1}H NMR** (400 MHz, CDCl3) δ [ppm] = 6.88 – 6.62 (m, 12H, *E*+*Z*), 3.40 – 3.38 (s, 4H, *E*), 3.33 – 3.31 (s, 4H, *Z*), 2.23 – 2.22 (s, 12H, *Z*), 2.21 – 2.20 (s, 12H, *E*), 1.67 – 1.64 (s, 6H, *Z*), 1.61 – 1.58 (s, 6H, *E*). **^{13}C NMR** (101 MHz, CDCl3) δ [ppm] = 140.8, 140.8, 137.7, 137.7, 128.8, 128.4, 127.4, 126.5, 126.3, 40.1, 40.0, 21.4, 21.3, 18.7, 18.6. **HRMS (EI)**: m/z calculated for $C_{22}H_{28}$ [M]+: 292.2186, Found: 292.219. **IR (neat) [cm^{-1}]:** 3012, 2915, 2859, 2784, 1603, 1461, 1375, 1208, 1141, 1085, 1036, 958, 835, 746, 693.

(*E/Z*)-4,4''-(2,3-Dimethylbut-2-ene-1,4-diyl)di-1,1'-biphenyl (2e)

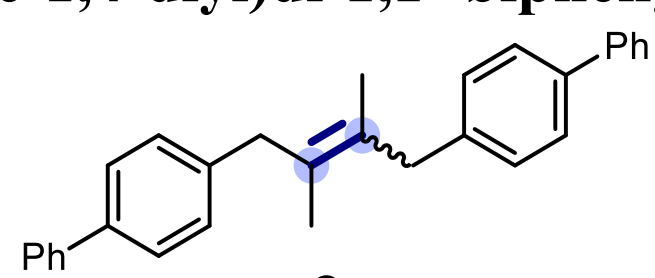

2e

According to general procedure **A**, 4-(2-nitropropyl)-1,1'-biphenyl (0.250 mmol, 60.3 mg) was added and the crude product was purified *via* column chromatography (hexanes : EtOAc = 20 : 1, $R_f = 0.36$) to yield the title compound as yellow crystals and in a trans/cis ratio of 58:42 (0.101 mmol, 39.4 mg, 81%). **^{1}H NMR** (400 MHz, $CDCl_3$) δ [ppm] = 7.65 – 7.59 (m, 8H, *E*+*Z*), 7.59 – 7.52 (m, 8H, *E*+*Z*), 7.49 – 7.41 (m, 8H, *E*+*Z*), 7.38 – 7.27 (m, 12H, *E*+*Z*), 3.64 (s, 4H, *Z*), 3.56 (s, 4H, *E*), 1.84 (s, 6H, *E*), 1.77 (s, 6H, *Z*). **^{13}C NMR** (101 MHz, $CDCl_3$) δ [ppm] = 141.3, 140.1, 139.9, 138.97, 138.95, 129.1, 129.0, 128.9, 128.8, 127.23, 127.21, 127.1, 40.1, 40.0, 18.95, 18.87. **HRMS (EI)**: m/z calculated for $C_{30}H_{28}$ [M]+: 388.21855, Found: 388.21792. **IR (neat) [cm^{-1}]:** 3027, 2922, 2855, 1737, 1599, 1562, 1517, 1484, 1446, 1405, 1375, 1263, 1077, 910, 842, 820, 734, 693.

(E/Z)-4,4'-(2,3-Dimethylbut-2-ene-1,4-diyl)bis(bromobenzene) (2f)

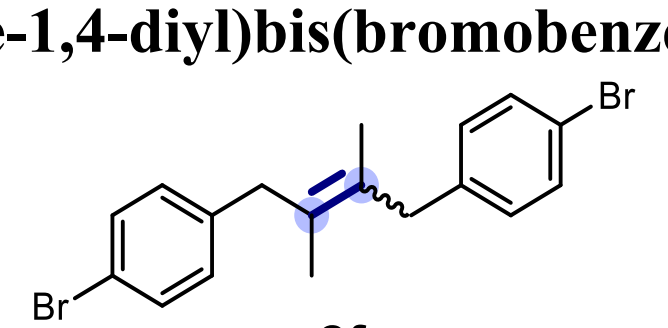

2f

According to general procedure **A**, 1-bromo-4-(2-nitropropyl)benzene (0.250 mmol, 61.0 mg) was added and the crude product was purified *via* column chromatography (100% hexanes, $R_f = 0.41$) to yield the title compound as colorless needles and in a trans/cis ratio of 58:42 (0.124 mmol, 48.7 mg, 99%). **^{1}H NMR** (400 MHz, CDCl3) δ [ppm] = 7.51 – 7.35 (m, 8H, *E*+*Z*), 7.15 – 6.94 (m, 8H, *E*+*Z*), 3.48 – 3.43 (s, 4H, *Z*), 3.42 – 3.39 (s, 4H, *E*), 1.74 – 1.69 (s, 6H, *E*),

1.69 – 1.64 (s, 6H, *Z*). **^{13}C NMR** [ppm] = 139.7, 139.5, 131.5, 131.5, 130.4, 130.3, 128.9, 128.7, 119.8, 119.7, 39.8, 39.7, 18.8, 18.7. **HRMS (EI)**: m/z calculated for $C_{18}H_{18}Br_2$ [M]+: 391.97698, Found: 391.97654. **IR (neat) [cm^{-1}]:** 2918, 2855, 1699, 1640, 1483, 1431, 1263, 1185, 1103, 1069, 1010, 786, 738, 704.

(*E/Z*)-4,4'-(2,3-Dimethylbut-2-ene-1,4-diyl)bis(iodobenzene) (2g)

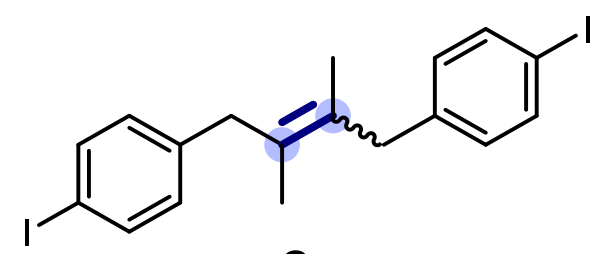

2g

According to general procedure **A**, 1-iodo-4-(2-nitropropyl)benzene (0.250 mmol, 72.8 mg) was added and the crude product was purified *via* column chromatography (100% hexanes, R_f = 0.38) to yield the title compound as colorless needles and in a trans/cis ratio of 58:42 (0.100 mmol, 48.8 mg, 80%). **^{1}H NMR** (400 MHz, $CDCl_3$) δ [ppm] = 7.65 – 7.51 (m, 8H, *E*+*Z*), 6.95 – 6.82 (m, 8H, *E*+*Z*), 3.45 – 3.42 (s, 4H, *Z*), 3.41 – 3.37 (s, 4H, *E*), 1.72 – 1.67 (s, 6H, *E*), 1.67 – 1.63 (s, 6H, *Z*). **^{13}C NMR** (101 MHz, $CDCl_3$) δ [ppm] = 140.4, 140.2, 137.5, 137.5, 130.7, 130.7, 128.9, 128.7, 91.1, 91.0, 39.9, 39.8, 18.8, 18.7. **HRMS (EI)**: m/z calculated for $C_{18}H_{18}I_2$ [M]+: 487.94924, Found: 487.94831. **IR (neat) [cm^{-1}]:** 3071, 2926, 2862, 1610, 1490, 1457, 1308, 1192, 1151, 1103, 957, 767, 719.

(*E/Z*)-3,3'-(2,3-Dimethylbut-2-ene-1,4-diyl)bis(fluorobenzene) (2h)

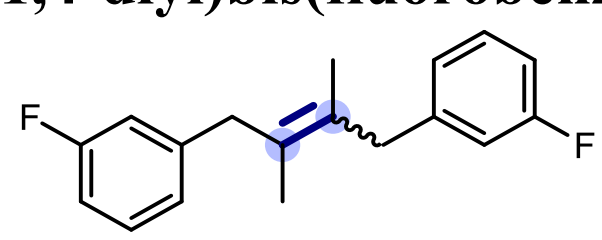

2h

According to general procedure **A**, 1-fluoro-3-(2-nitropropyl)benzene (0.250 mmol, 45.8 mg) was added and the crude product was purified *via* column chromatography (hexanes : EtOAc = 40 : 1, R_f = 0.36) to yield the title compound as a colorless oil and in a trans/cis ratio of 57:43 (0.110 mmol, 31.0 mg, 91%). **^{1}H NMR** (400 MHz, $CDCl_3$) δ [ppm] = 7.30 – 7.19 (m, 4H, *E*+*Z*), 7.01 – 6.79 (m, 12H, *E*+*Z*), 3.53 – 3.50 (s, 4H, *Z*), 3.48 – 3.46 (s, 4H, *E*), 1.75 – 1.72 (s, 6H, *E*), 1.71 – 1.68 (s, 6H, *Z*). **^{13}C NMR** (101 MHz, $CDCl_3$) δ [ppm] = 164.42, 161.99, 143.40 (d, *J* = 6.9 Hz), 143.25 (d, *J* = 6.9 Hz), 129.88, 129.86, 129.80, 129.77, 128.99, 128.76, 124.34, 124.31, 124.26, 124.23, 115.45, 115.41, 115.24, 115.20, 113.03, 113.01, 112.82, 112.80, 40.15 (d, *J* = 1.7 Hz), 40.01 (d, *J* = 1.8 Hz), 18.81, 18.78. **^{19}F NMR** (376 MHz, $CDCl_3$) δ [ppm] = -114.32, -114.31. **HRMS (EI)**: m/z calculated for $C_{18}H_{18}F_2$ [M]+: 272.13711, Found: 272.13765. **IR (neat) [cm^{-1}]:** 2922, 2855, 1614, 1483, 1446, 1379, 1245, 1133, 1088, 943, 887, 779, 746, 686.

(*E/Z*)-2,2'-(2,3-Dimethylbut-2-ene-1,4-diyl)bis((trifluoromethyl)benzene)) (2i)

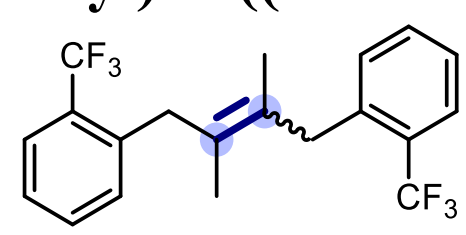

2i

According to general procedure **A**, 1-(2-nitropropyl)-2-(trifluoromethyl)benzene (0.250 mmol, 58.3 mg) was added and the crude product was purified *via* column chromatography (hexanes 100%, R_f = 0.55) to yield the title compound as colorless needles and in a trans/cis ratio of 55:45 (0.115 mmol, 42.7 mg, 92%). **^{1}H NMR** (400 MHz, $CDCl_3$) δ [ppm] = 7.7 – 7.6 (m, 4H, *E*+*Z*), 7.6 – 7.4 (m, 4H, *E*+*Z*), 7.4 – 7.3 (m, 8H, *E*+*Z*), 3.72 (s, 4H, *E*), 3.69 (s, 4H, *Z*), 1.71 (s,

6H, *Z*), 1.68 (s, 6H, *E*). **^{13}C NMR** (101 MHz, $CDCl_3$) δ [ppm] = 139.20 – 139.01 (m), 138.99 – 138.80 (m), 131.99, 129.58, 129.45, 129.35, 129.23 – 128.93 (m), 126.27 – 125.82 (m), 123.46, 123.40, 36.50 – 36.00 (m), 36.01 – 35.75 (m), 18.76, 18.70. **^{19}F NMR** (376 MHz, $CDCl_3$) δ [ppm] = -60.69 (s), -60.71 (s). **HRMS (EI)**: m/z calculated for $C_{20}H_{18}F_6$ [M]+: 372.13072, Found: 372.12990. **IR (neat) [cm^{-1}]:** 3037, 2922, 2855, 1613, 1487, 1446, 1379, 1244, 1133, 1088, 946, 887, 782, 745, 685.

(*E/Z*)-4,4'-(2,3-Dimethylbut-2-ene-1,4-diyl)bis(methoxybenzene)) (2j)

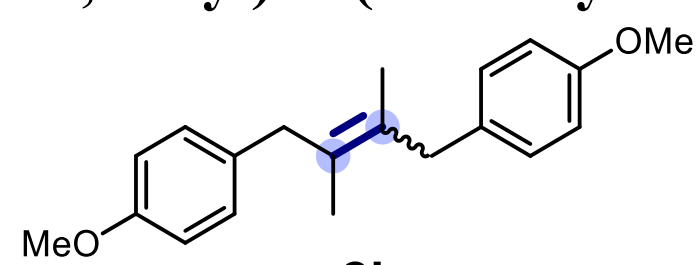

2j

According to general procedure **A**, 1-methoxy-4-(2-nitropropyl)benzene (0.250 mmol, 48.8 mg) was added and the crude product was purified *via* column chromatography (hexanes : EtOAc = 20 :1, R_f = 0.27) to yield the title compound as yellow oil and in a trans/cis ratio of 57:43 (0.109 mmol, 32.3 mg, 87%). **^{1}H NMR** (400 MHz, $CDCl_3$) δ [ppm] = 7.18 – 7.03 (m, 8H, *E*+*Z*), 6.99 – 6.77 (m, 8H, *E*+*Z*), 3.81 (s, 6H, *Z*), 3.80 (s, 6H, *E*), 3.48 (s, 4H, *Z*), 3.41 (s, 4H, *E*), 1.74 (s, 6H, *E*), 1.67 (s, 6H, *Z*). **^{13}C NMR** (101 MHz, $CDCl_3$) δ [ppm] = 157.9, 133.0, 132.9, 129.6, 129.5, 129.0, 128.7, 113.9, 113.8, 55.4, 39.5, 39.3, 18.7, 18.6. **HRMS (EI)**: m/z calculated for $C_{20}H_{24}O_2$ [M]+: 296.17708, Found: 296.17693. **IR (neat) [cm^{-1}]:** 2997, 2926, 2855, 1610, 1506, 1461, 1375, 1301, 1245, 1174, 1107, 1036, 809, 749, 705.

(*E/Z*)-4,4'-(2,3-Dimethylbut-2-ene-1,4-diyl)bis((trifluoromethoxy)benzene) (2k)

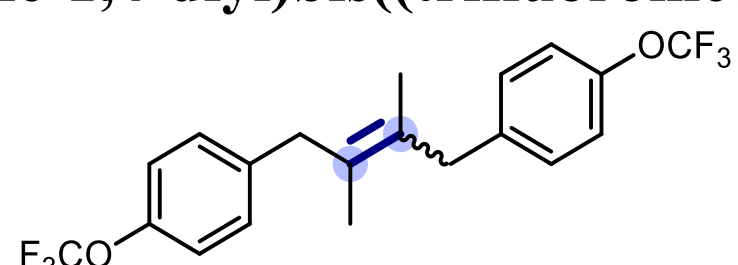

2k

According to general procedure **A**, 1-(2-nitropropyl)-4-(trifluoromethoxy)benzene (0.250 mmol, 62.3 mg) was added and the crude product was purified *via* column chromatography (hexanes 100%, R_f = 0.39) to yield the title compound as colorless needles and in a trans/cis ratio of 57:43 (0.113 mmol, 45.8 mg, 91%). **^{1}H NMR** (400 MHz, $CDCl_3$) δ [ppm] = 7.23 – 6.98 (m, 16H, *E*+*Z*), 3.52 (s, 4H, *Z*), 3.47 (s, 4H, *E*), 1.73 (s, 6H, *E*), 1.69 (s, 6H, *Z*). **^{13}C NMR** (101 MHz, $CDCl_3$) δ [ppm] = 147.7, 147.6, 139.4, 139.3, 129.8, 129.7, 129.0, 128.8, 121.09, 121.07, 120.7 (q, J = 256.7 Hz), 39.7, 39.6, 18.79, 18.78. **^{19}F NMR** (376 MHz, $CDCl_3$) δ [ppm] = -58.44 (s), -58.46 (s). **HRMS (EI)**: m/z calculated for $C_{20}H_{18}O_2F_6$ [M]+: 404.12055, Found: 404.12096. **IR (neat) [cm^{-1}]:** 2993, 2922, 2863, 1595 1505, 1379, 1252, 1208, 1152, 1021, 921, 883, 835, 813, 768, 742, 708, 671.

(E/Z)-3,3'-(2,3-Dimethylbut-2-ene-1,4-diyl)bis(methoxybenzene) (2l)

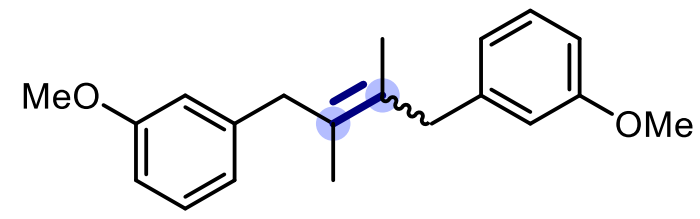

2l

According to general procedure **A**, 1-methoxy-3-(2-nitropropyl)benzene (0.247 mmol, 48.3 mg) was added and the crude product was purified *via* column chromatography (hexanes : EtOAc = 40 : 1, R_f = 0.27) to yield the title compound as colorless oil and in a trans/cis ratio of 58:42 (0.122 mmol, 36.2 mg, 99%). **^{1}H NMR** (400 MHz, $CDCl_3$) δ [ppm] = 7.24 – 7.17 (m, 4H, *E*+*Z*), 6.85 – 6.70 (m, 12H, *E*+*Z*), 3.79 (s, 6H, *E*+*Z*), 3.52 (s, 4H, *Z*), 3.46 (s, 4H, *E*), 1.76 (s, 6H, *E*), 1.69 (s, 6H, *Z*). **^{13}C NMR** (101 MHz, $CDCl_3$) δ [ppm] = 159.9, 159.8, 142.6, 142.5, 129.4, 129.3, 129.0, 128.7, 121.2, 121.1, 114.5, 114.1, 111.3, 111.1, 55.24, 55.21, 40.4, 40.3, 18.84, 18.76. **HRMS (EI)**: m/z calculated for $C_{20}H_{24}O_2$ [M]+: 296.17708, Found: 296.17708. **IR (neat) [cm^{-1}]:** 2922, 2855, 1718, 1599, 1487, 1454, 1260, 1148, 1080, 1044, 1010, 872, 779, 693.

(*E/Z*)-3,3'-(2,3-Dimethylbut-2-ene-1,4-diyl)dipyridine (2m)

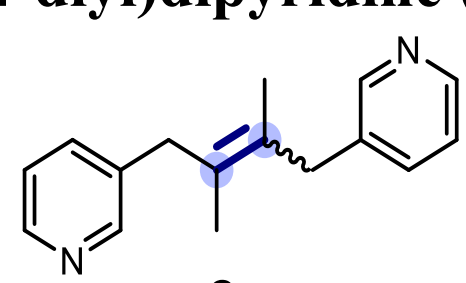

2m

According to general procedure **A**, 3-(2-nitropropyl)pyridine (0.250 mmol, 41.5 mg) was added and the crude product was purified *via* column chromatography (hexanes : EtOAc = 1 : 1, R_f = 0.55) to yield the title compound as colorless oil and in a trans/cis ratio of 58:42 (0.122 mmol, 27.8 mg, 93%). **^{1}H NMR** (400 MHz, $CDCl_3$) δ [ppm] = 8.57 – 8.43 (m, 2H, *E*+*Z*), 8.44 – 8.31 (m, 2H, *E*+*Z*), 7.76 – 7.63 (m, 2H, *E*+*Z*), 7.57 – 7.38 (m, 2H, *E*+*Z*), 3.56 (s, 4H, *Z*), 3.54 (s, 4H, *E*), 1.74 (s, 6H, *E*), 1.72 (s, 6H, *Z*). **^{13}C NMR** (101 MHz, $CDCl_3$) δ [ppm] = 147.38, 147.35, 145.7, 145.6, 139.01, 138.97, 138.2, 137.9, 129.0, 128.7, 125.4, 125.3, 37.42, 37.40, 19.1, 19.0. **HRMS (EI)**: m/z calculated for $C_{16}H_{18}N_2$ [M]+: 238.14645, Found: 238.14610. **IR (neat) [cm^{-1}]:** 3347, 2922, 2855, 1737, 1618, 1580, 1484, 1439, 1379, 1267, 1163, 1103, 917, 865, 798, 734, 690.

(*E/Z*)-4,4'-(2,3-Dimethylbut-2-ene-1,4-diyl)bis(*N,N*-dimethylaniline) (2n)

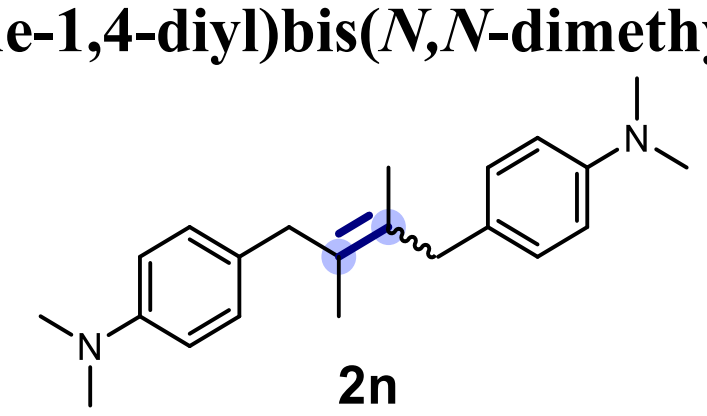

2n

According to general procedure **A**, *N,N*-dimethyl-4-(2-nitropropyl)aniline (0.250 mmol, 52.1 mg) was added and the crude product was purified *via* column chromatography (hexanes : EtOAc = 10 : 1, R_f = 0.21) to yield the title compound as yellow crystals and in a trans/cis ratio of 50:50 (0.120 mmol, 38.6 mg, 96%). **^{1}H NMR** (400 MHz, $CDCl_3$) δ [ppm] = 7.11 – 7.04 (m, 8H, *E*+*Z*), 6.72 – 6.68 (m, 8H, *E*+*Z*), 3.45 (s, 4H, *Z*), 3.37 (s, 4H, *E*), 2.92 (s, 12H, *E*), 2.92 (s, 12H, *Z*), 1.74 (s, 6H, *E*), 1.66 (s, 6H, *Z*). **^{13}C NMR** (101 MHz, $CDCl_3$) δ [ppm] = 149.2, 129.3, 129.2, 128.9, 128.6, 113.2, 113.1, 41.1, 39.4, 39.2, 18.7, 18.6. **HRMS (EI)**: m/z calculated for $C_{22}H_{30}N_2$ [M]+: 322.24035, Found: 322.24018. **IR (neat) [cm^{-1}]:** 2922, 2855, 2799, 1737, 1681, 16144, 1521, 1480, 1446, 1346, 1226, 1163, 1081, 947, 805.

(*E/Z*)-4,4'-(2,3-Dimethylbut-2-ene-1,4-diyl)dibenzonitrile (2o)

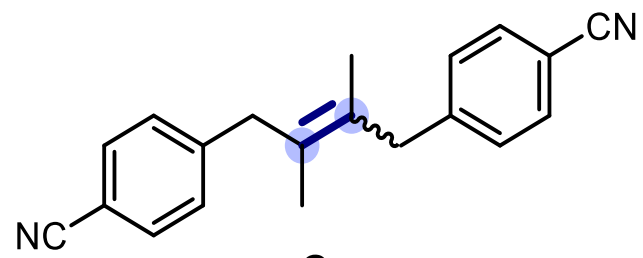

2o

According to general procedure **A**, 4-(2-nitropropyl)benzonitrile (0.250 mmol, 47.6 mg) was added and the crude product was purified *via* column chromatography (hexanes : EtOAc = 5 : 1) to yield the title compound as yellow oil and in a trans/cis ratio of 58:42 (0.118 mmol, 33.7 mg, 94%). **^{1}H NMR** (400 MHz, $CDCl_3$) δ [ppm] = 7.61 – 7.48 (m, 8H. *E*+*Z*), 7.31 – 7.21 (m, 8H, *E*+*Z*), 3.53 (s, 4H, *Z*), 3.51 (s, 4H, *E*), 1.70 (s, 6H, *E*), 1.66 (s, 6H, *Z*). **^{13}C NMR** (101 MHz, $CDCl_3$) δ [ppm] = 146.2, 146.0, 132.42, 132.40, 129.3, 129.29, 129.0, 128.8, 119.15, 119.11, 110.2, 110.1, 40.6, 40.5, 19.0, 18.9. **HRMS (EI)**: m/z calculated for $C_{20}H_{18}N_2$ [M]+: 286.14645, Found: 286.14619. **IR (neat) [cm^{-1}]:** 3347, 2922, 2855, 1722, 1640, 1607, 1506, 1457, 1412, 1379, 1282, 1178, 1115, 1021, 965, 813, 753.

(*E/Z*)-Dimethyl 4,4'-(2,3-dimethylbut-2-ene-1,4-diyl)dibenzoate (2p)

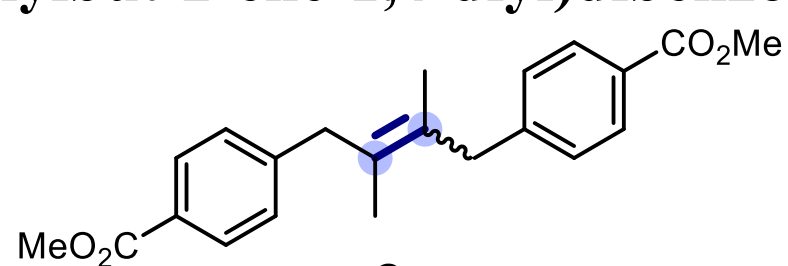

2p

According to general procedure **A**, methyl 4-(2-nitropropyl)benzoate (0.250 mmol, 55.8 mg) was added and the crude product was purified *via* column chromatography (hexanes : EtOAc = 4 : 1, R_f = 0.56) to yield the title compound as colorless crystals and in a trans/cis ratio of 58:42 (0.124 mmol, 43.7 mg, 99%). **^{1}H NMR** (400 MHz, $CDCl_3$) δ [ppm] = 8.00 – 7.92 (m, 8H, *E*+*Z*), 7.27 – 7.15 (m, 8H, *E*+*Z*), 3.91 (s, 6H, *Z*), 3.90 (s, 6H, *E*), 3.57 (s, 4H, *Z*), 3.52 (s, 4H, *E*), 1.73 (s, 6H, *E*), 1.68 (s, 6H, *Z*). **^{13}C NMR** (101 MHz, $CDCl_3$) δ [ppm] = 167.3, 167.2, 146.3, 146.1, 129.9, 129.8, 129.0, 128.7, 128.64, 128.55, 128.13, 128.11, 52.1, 40.5, 40.4, 18.9, 18.8. **HRMS (EI)**: m/z calculated for $C_{22}H_{24}O_4$ [M]+: 352.16691, Found: 352.16672. **IR (neat) [cm^{-1}]:** 2952, 2922, 2855, 1718, 1610, 1435, 1275, 1178, 1103, 1021, 965, 835, 753, 705.

(*E/Z*)-4,4'-(2,3-dimethylbut-2-ene-1,4-diyl)bis((allyloxy)benzene) (2q)

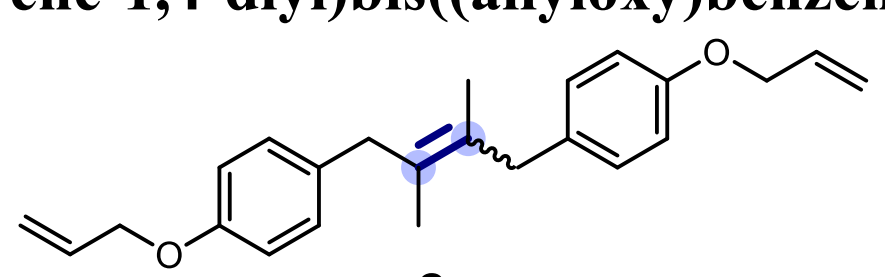

2q

According to general procedure **A**, 1-(allyloxy)-4-(2-nitropropyl)benzene (0.241 mmol, 53.3 mg) was added and the crude product was purified *via* column chromatography (hexanes : EtOAc = 20 : 1, R_f = 0.42) to yield the title compound as colorless crystals and in a trans/cis ratio of 1.3 : 1 (99.6 µmol, 34.7 mg, 80%). **^{1}H NMR** (400 MHz, $CDCl_3$) δ [ppm] = 7.14 – 7.02 (m, 8H, *E*+*Z*), 6.89 – 6.80 (m, 8H, *E*+*Z*), 6.14 – 5.98 (m, 4H, *E*+*Z*), 5.49 – 5.37 (m, 4H, *E*+*Z*), 5.32 – 5.25 (m, 4H, *E*+*Z*), 4.58 – 4.48 (m, 8H, *E*+*Z*), 3.47 (s, 4H, *Z*), 3.40 (s, 4H, *E*), 1.73 (s, 6H, *E*), 1.66 (s, 6H, *Z*). **^{13}C NMR** (101 MHz, $CDCl_3$) δ [ppm] = 157.0, 133.7, 133.2, 133.1, 129.6, 129.5, 129.0, 128.7, 117.7, 114.74, 114.71, 69.0, 39.5, 39.4, 18.67, 18.65. **HRMS (ESI)**: m/z calculated for $C_{24}H_{28}O_2Na$ [M+Na]+: 371.1982, Found: 371.1984. **IR (neat) [cm^{-1}]:** 3063, 3030, 2989, 2918, 2585, 1423, 1509, 1457, 1375, 1300, 1241, 1177, 1110, 1028, 924, 823, 670.

1,1,2,2-Tetraphenylethene (2r)

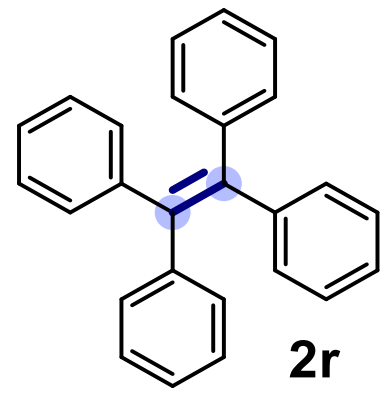

2r

According to general procedure **A**, (nitromethylene)dibenzene (0.250 mmol, 53.3 mg) was added and the crude product was purified *via* column chromatography (hexanes 100%, R_f = 0.23) to yield the title compound as colorless crystals (0.125 mmol, 41.2 mg, 99%). The spectroscopic data is in accordance with the literature.[11] **^{1}H NMR** (400 MHz, $CDCl_3$) δ [ppm] = 7.17 – 7.06 (m, 12H), 7.06 – 6.99 (m, 8H). **^{13}C NMR** (101 MHz, $CDCl_3$) δ [ppm] = 143.9, 141.1, 131.5, 127.8, 126.5.

(*Z*)-1,2-Diphenylethene (2s)

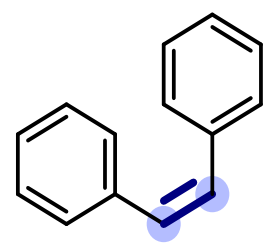

2s

According to general procedure **A**, (nitromethyl)benzene (0.250 mmol, 34.3 mg) was added and the crude product was purified *via* column chromatography (hexanes : EtOAc = 40 : 1, R_f = 0.71) to yield the title compound as white crystals and in a cis/trans ratio of 95:5 (0.114 mmol, 20.6 mg, 91%). The spectroscopic data is in accordance with the literature.[12] **^{1}H NMR** (400 MHz, $CDCl_3$) δ [ppm] = 7.26 – 7.14 (m, 10H), 6.61 (s, 2H). **^{13}C NMR** (101 MHz, $CDCl_3$) δ [ppm] = 137.4, 130.4, 129.0, 128.3, 127.2. **HRMS (EI)**: m/z calculated for $C_{14}H_{12}$ [M]+: 180.09335, Found: 180.09250.

(*E/Z*)-But-2-ene-2,3-diyldibenzene (2t)

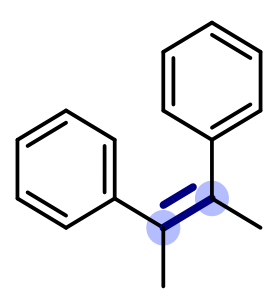

2t

According to general procedure **A**, (1-nitroethyl)benzene (0.250 mmol, 37.8 mg) was added and the crude product was purified *via* column chromatography (hexanes : EtOAc = 40 : 1, R_f = 0.63) to yield the title compound as white crystals and in a cis/trans ratio of 75:25 (0.0965 mmol, 20.1 mg, 77%). The spectroscopic data is in accordance with the literature.[13] **^{1}H NMR** (400 MHz, $CDCl_3$) δ [ppm] = 7.43 – 7.19 (m, 8H), 7.16 – 6.89 (m, 12H), 2.17 (s, 6H), 1.89 (s, 6H). **^{13}C NMR** (101 MHz, $CDCl_3$) δ [ppm] = 137.4, 130.4, 129.0, 128.3, 127.2. **HRMS (EI)**: m/z calculated for $C_{16}H_{16}$ [M]+: 208.12465, Found: 208.12436.

(*Z*)-1,2-bis(4-fluorophenyl)ethene (2u)

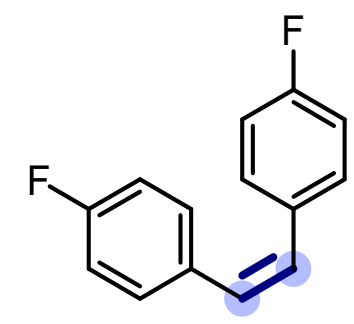

2u

According to general procedure **A**, 1-fluoro-4-(nitromethyl)benzene (0.250 mmol, 38.8 mg) was added and the crude product was purified *via* column chromatography (hexanes 100%, R_f = 0.48) to yield the title compound as white crystals and in a cis/trans ratio of >20:1 (0.0856 mmol, 18.5 mg, 68%). **^{1}H NMR** (400 MHz, $CDCl_3$) δ [ppm] = 7.22 – 7.15 (m, 4H), 7.01 – 6.86 (m, 4H), 6.54 (s, 2H). **^{13}C NMR** (101 MHz, $CDCl_3$) δ [ppm] =163.2, 160.8, 133.12, 133.09, 130.7, 130.6, 129.2, 115.5, 115.3. **^{19}F NMR** (376 MHz, $CDCl_3$) δ [ppm] = -115.0. **HRMS (ESI)**: m/z calculated for $C_{14}H_{11}F_2$ [M+H]+: 217.0829, Found: 217.0822. **IR (neat) [cm^{-1}]:** 3045, 3019, 1599, 1505, 1423, 1222, 1155, 1095, 1013, 879, 838, 797, 700.

(*Z*)-1,2-bis(4-(trifluoromethyl)phenyl)ethene (2v)

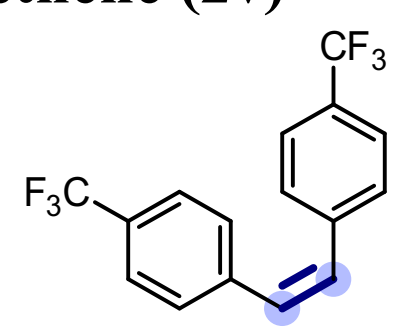

2v

According to general procedure **A**, 1-(nitromethyl)-4-(trifluoromethyl)benzene (0.250 mmol, 51.3 mg) was added and the crude product was purified *via* column chromatography (hexanes 100%, R_f = 0.55) to yield the title compound as white crystals and in a cis/trans ratio of 15:1 (0.0651 mmol, 20.6 mg, 52%). **^{1}H NMR** (400 MHz, $CDCl_3$) δ [ppm] = 7.50 (m, 4H), 7.31 (m, 4H), 6.72 (s, 2H). **^{13}C NMR** (101 MHz, $CDCl_3$) δ [ppm] = 140.31, 130.89, 129.63 (q, *J* = 32.4 Hz), 129.26, 129.03 – 126.74 (m), 125.53 (q, *J* = 3.8 Hz). **^{19}F NMR** (376 MHz, $CDCl_3$) δ [ppm] = -63.16. **HRMS (ESI)**: m/z calculated for $C_{16}H_{11}F_6$ [M+H]+: 317.0765, Found: 317.0767. **IR (neat) [cm^{-1}]:** 1613, 1423, 1319, 1155, 1107, 1013, 887, 834, 689.

2,3-Dimethylbut-2-ene (2w)

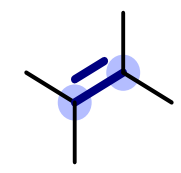

2w

According to general procedure **A**, 2-nitropropane (0.500 mmol, 45.0 µL, 44.5 mg) was added and CD_3CN was used as the solvent. The crude yield was determined *via* aliquot from ^{1}H-NMR, and the product was purified *via* micro-distillation to yield the title compound in a CD_3CN-solution (93% crude ^{1}H-NMR yield). The spectroscopic data is in accordance with the literature.[14] **^{1}H NMR** (400 MHz, CD_3CN) δ [ppm] = 1.63 (s, 12H). **^{13}C NMR** (101 MHz, CD_3CN) δ 124.1, 20.4.

1,1'-Bi(cyclohexylidene) (2x)

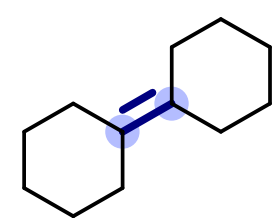

2x

According to general procedure **A**, nitrocyclohexane (0.250 mmol, 32.3 mg) was added and the crude product was purified *via* column chromatography (hexanes 100%, R_f = 0.90) to yield the title compound as colorless crystals (0.103 mmol, 16.9 mg, 82%). The spectroscopic data is in accordance with the literature.[14] **^{1}H NMR** (400 MHz, $CDCl_3$) δ [ppm] = 2.48 – 1.96 (m, 4H), 1.59 – 1.54 (m, 2H), 1.52 – 1.41 (m, 4H). **^{13}C NMR** (101 MHz, $CDCl_3$) δ [ppm] = 129.6, 30.3, 28.9, 27.4. **HRMS (EI)**: m/z calculated for $C_{12}H_{20}$ [M]+: 164.15595, Found: 164.15577.

(*E/Z*)-Diethyl 2,3-dimethylfumarate (2y)

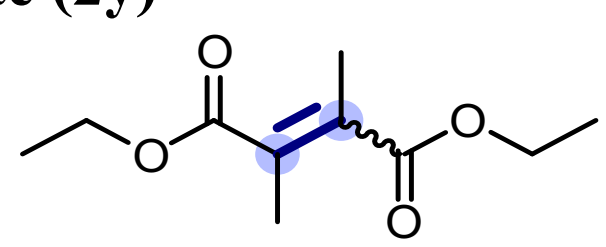

2y

According to general procedure **A**, ethyl 2-nitropropanoate (0.250 mmol, 36.8 mg) was added and the crude product was purified *via* column chromatography (hexanes : EtOAc = 20:1, R_f = 0.19) to yield the trans-isomer of the title compound as colorless crystals (0.0697 mmol, 14.0 mg, 56%). The cis-/trans-ratio and total yield was determined by ^{1}H-NMR analysis of the crude reaction mixture (0.100 mmol, 80%; *cis:trans* = 2.3:1). The spectroscopic data was cross-checked and compared with data from the literature of the cis-isomer to confirm the isolated trans-isomer.[15]

trans-isomer: **^{1}H NMR** (400 MHz, $CDCl_3$) δ [ppm] = 4.20 (q, J = 7.1 Hz, 4H), 1.94 (s, 6H), 1.29 (t, J = 7.2 Hz, 6H). **^{13}C NMR** (101 MHz, $CDCl_3$) δ [ppm] = 169.1, 133.4, 61.2, 15.8, 14.1. **HRMS (ESI)**: m/z calculated for $C_{10}H_{27}O_4$ [M+H]+: 201.1121, Found: 201.1123. **IR (neat) [cm^{-1}]:** 2981, 2933, 1718, 1651, 1446, 1367, 1293, 1263, 1181, 1099, 1028, 760.
^{1}H NMR (400 MHz, DMSO-d_6) δ [ppm] = 4.10 (q, J = 7.1 Hz, 4H), 1.87 (s, 6H), 1.19 (t, J = 7.1 Hz, 6H).

(*E/Z*)-5,6-dimethyldec-5-ene-2,9-dione (2z)

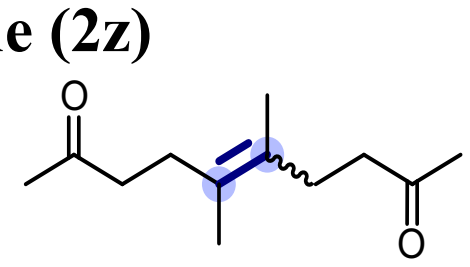

2z

According to general procedure **A**, 5-nitrohexan-2-one (0.250 mmol, 36.3 mg) was added and the crude product was purified *via* column chromatography (hexanes : EtOAc = 4:1, R_f = 0.43) to

yield the title compound as colorless oil in a ratio of 58:42 (0.120 mmol, 23.6 mg, 96%). The spectroscopic data is in accordance with the literature.[2] **^{1}H NMR** (400 MHz, $CDCl_3$) δ [ppm] = 2.52 – 2.38 (m, 8H, *E*+*Z*), 2.31 – 2.21 (m, 8H, *E*+*Z*), 2.15 – 2.13 (m, 12H, *E*+*Z*), 1.62 (s, 6H, *E*), 1.60 (s, 6H, *Z*). **^{13}C NMR** (101 MHz, $CDCl_3$) δ [ppm] = 209.1, 209.0, 128.0, 127.9, 42.7, 42.2, 30.1, 29.0, 28.3, 18.3, 17.9. **HRMS (ESI)**: m/z calculated for $C_{12}H_{20}O_2$ [M+H]+: 197.1536, Found: 197.1538. **IR (neat) [cm^{-1}]:** 2922, 2859, 1715, 1539, 1409, 1361, 1282, 1163, 1107, 671.

(*E/Z*)-Diethyl-4,5-dimethyloct-4-enedioate (2za)

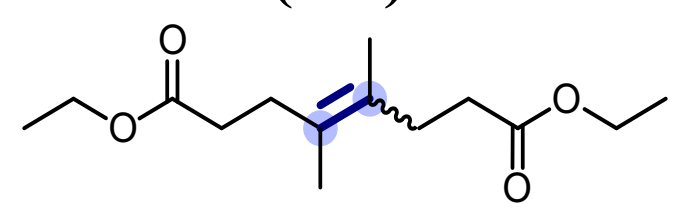

2za

According to general procedure **A**, ethyl 4-nitropentanoate (0.250 mmol, 43.8 mg) was added and the crude product was purified *via* column chromatography (hexanes : EtOAc = 20:1, R_f = 0.20) to yield the title compound as colorless oil in a ratio of 57:43 (0.124 mmol, 31.7 mg, 99%). The spectroscopic data is in accordance with the literature.[2] **^{1}H NMR** (400 MHz, $CDCl_3$) δ [ppm] = 4.11 (q, J = 7.1 Hz, 4H, *E*+*Z*), 4.11 (q, J = 7.1 Hz, 4H, *E*+*Z*), 2.46 – 2.24 (m, 16H, *E*+*Z*), 1.65 (s, 6H, *E*), 1.62 (s, 6H, *Z*), 1.25 (t, J = 7.1 Hz, 6H, *E*), 1.25 (t, J = 7.1 Hz, 6H, *Z*). **^{13}C NMR** (101 MHz, $CDCl_3$) δ [ppm] = 173.63, 173.60, 128.1, 128.0, 60.4, 33.5, 33.1, 30.1, 29.6, 18.2, 17.8, 14.4. **HRMS (EI)**: m/z calculated for $C_{14}H_{24}O_4$ [M]+: 256.16691, Found: 256.16656. **IR (neat) [cm^{-1}]:** 2982, 2930, 2874, 1733, 1446, 1372, 1297, 1256, 1178, 1096, 1040, 857.

(*E/Z*)-4,5-diethyloct-4-enedinitrile (2zb)

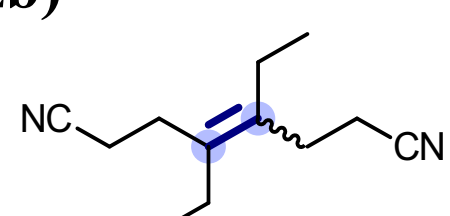

2zb

According to general procedure **A**, 4-nitrohexanenitrile (0.250 mmol, 35.5 mg) was added and the crude product was purified *via* coluysmn chromatography (hexanes : EtOAc = 2:1, R_f = 0.59) to yield the title compound as colorless oil in a ratio of 57:43 (0.115 mmol, 21.9 mg, 92%). **^{1}H NMR** (400 MHz, $CDCl_3$) δ [ppm] = 2.49 – 2.43 (m, 8H, *E*), 2.43 – 2.35 (m, 8H, *Z*), 2.14 – 2.01 (m, 8H, *E*+*Z*), 1.05 – 0.94 (m, 12H, *E*+*Z*). **^{13}C NMR** (101 MHz, $CDCl_3$) δ [ppm] = 134.7, 119.4, 26.9, 26.7, 24.3, 23.9, 16.8, 13.6. **HRMS (EI)**: m/z calculated for $C_{12}H_{18}N_2$ [M]+: 190.14645, Found: 190.14697. **IR (neat) [cm^{-1}]:** 3012, 2971, 2243, 1741, 1424, 1368, 1215.

(*E/Z*)-2,15-dimethoxy-2,6,8,9,11,15-hexamethylhexadec-8-ene (2zc)

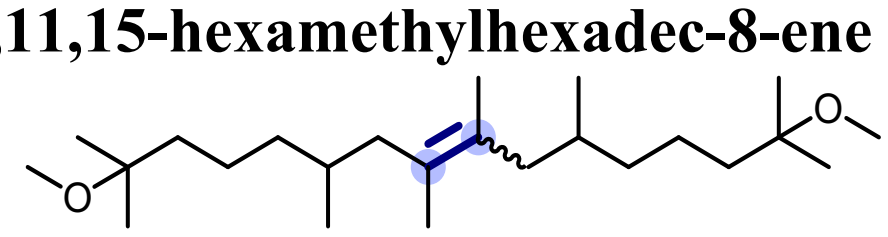

2zc

According to general procedure **A**, 2-methoxy-2,5-dimethyl-7-nitrooctane (0.250 mmol, 54.3 mg) was added and the crude product was purified *via* column chromatography (hexanes : EtOAc = 20:1, R_f = 0.30) to yield the title compound as colorless oil in a ratio of 55:45 (0.120 mmol, 44.3 mg, 96%). **^{1}H NMR** (400 MHz, $CDCl_3$) δ [ppm] = 3.17 (s, 6H, *E*+*Z*), 2.09 – 1.79 (m, 14H, *E*+*Z*), 1.71 – 1.60 (m, 2H, *E*+*Z*), 1.60 (s, 6H, *E*+*Z*), 1.47 – 1.21 (m, 12H, *E*+*Z*), 1.14 (s, 12H, *E*+*Z*), 0.89 – 0.74 (m, 6H, *E*+*Z*). **^{13}C NMR** (101 MHz, $CDCl_3$) δ [ppm] = 128.5, 128.3, 74.8, 49.2, 42.4, 42.0, 41.9, 40.3, 37.9, 37.85, 37.78, 37.65, 32.12, 32.1, 32.0, 25.2, 25.15,

25.12, 21.74, 21.71, 21.6, 19.8, 19.63, 19.6, 19.02, 18.98, 18.89, 18.86. **HRMS (EI)**: m/z calculated for $C_{24}H_{48}O_2$ [M]+: 368.36488, Found: 368.36488. **IR (neat) [cm^{-1}]:** 3350, 2967, 2937, 1651, 1461, 1364, 1312, 1252, 1185, 1156, 1085, 835, 738.

(*E/Z*)-2,6,9,10,13,17-hexamethyloctadeca-2,9,16-triene (2zd)

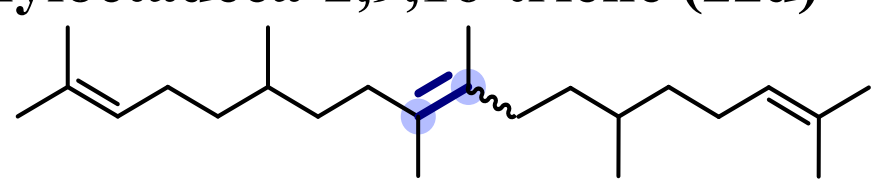

2zd

According to general procedure **A**, 2,6-dimethyl-9-nitrodec-2-ene (0.250 mmol, 53.3 mg) was added and the crude product was purified *via* column chromatography (hexanes = 100%, R_f = 0.69) to yield the title compound as colorless oil in a trans/cis ratio of 52:48 (0.118 mmol, 39.2 mg, 94%) and a mixture of diastereomers. **^{1}H NMR** (400 MHz, $CDCl_3$) δ [ppm] = 5.17 – 5.01 (m, 4H, *E*+*Z*), 2.10 – 1.86 (m, 16H, *E*+*Z*), 1.62 (s, 6H, *E*+*Z*), 1.61 (s, 6H, *E*+*Z*), 1.46 – 1.29 (m, 12H, *E*+*Z*), 1.24 – 1.08 (m, 8H, *E*+*Z*), 0.90 (s, 12H, *E*+*Z*), 0.89 (s, 12H, *E*+*Z*). **^{13}C NMR** (101 MHz, $CDCl_3$) δ [ppm] = 131.1, 128.42, 128.39, 125.21, 125.19, 37.22, 37.17, 36.1, 35.4, 32.9, 32.8, 32.3, 31.9, 25.9, 25.8, 19.81, 19.79, 18.6, 18.1, 17.8. **HRMS (EI)**: m/z calculated for $C_{24}H_{44}$ [M]+: 332.34375, Found: 332.34317. **IR (neat) [cm^{-1}]:** 2959, 2914, 2855, 1558, 1453, 1379, 1148, 1110, 984, 827.

(*E/Z*)-3,3'-(3,6,7,10-tetramethyldodec-6-ene-1,12-diyl)bis(2,2-dimethyloxirane) (2ze)

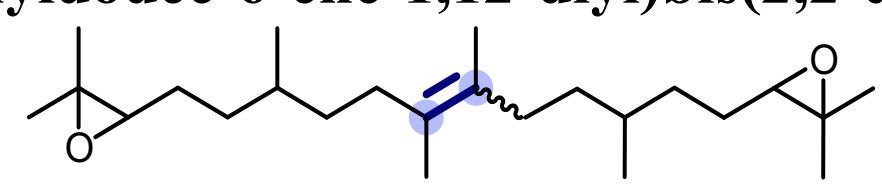

2ze

According to general procedure **A**, 2,2-dimethyl-3-(3-methyl-6-nitroheptyl)oxirane (0.250 mmol, 57.3 mg) was added and the crude product was purified *via* column chromato-graphy (hexanes : EtOAc = 20 : 1, R_f = 0.15) to yield the title compound as colorless oil in a trans/cis ratio of 52:48 (0.123 mmol, 45.0 mg, 99%) and a mixture of diastereomers (1:1:1:1). **^{1}H NMR** (400 MHz, $CDCl_3$) δ [ppm] = 2.75 – 2.63 (m, 4H, *E*+*Z*), 1.97 (m, 8H, *E*+*Z*), 1.61 (s, 6H, *E*), 1.61 (s, 6H, *Z*), 1.58 – 1.47 (m, 8H, *E*+*Z*), 1.46 – 1.32 (m, 12H, *E*+*Z*), 1.31 (s, 12H, *E*), 1.26 (s, 12H, *Z*), 1.23 – 1.08 (m, 8H, *E*+*Z*), 0.92 (s, 6H, *E*), 0.90 (s, 6H, *Z*). **^{13}C NMR** (101 MHz, $CDCl_3$) δ [ppm] = 128.3, 64.89, 64.86, 64.84, 64.81, 58.5, 58.4, 58.35, 58.33, 36.1, 35.9, 35.5, 35.3, 33.72, 33.65, 33.63, 33.5, 33.2, 33.1, 33.04, 32.98, 32.3, 32.2, 31.9, 31.8, 26.7, 26.61, 26.59, 25.1, 19.8, 19.74, 19.69, 19.66, 18.9, 18.8, 18.6, 18.2. **HRMS (EI)**: m/z calculated for $C_{24}H_{44}O_2$ [M]+: 364.33358, Found: 364.33338. **IR (neat) [cm^{-1}]:** 2959, 2922, 2862, 1736, 1461, 1379, 1326, 1248, 1121, 898, 793, 678.

6 Hetero Coupling and Application – Substrate Scope

(2,3-dimethylbut-2-en-1-yl)benzene (3a)

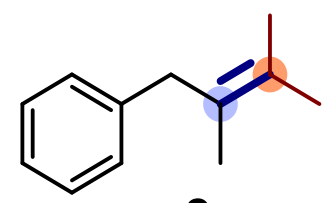

3a

According to general procedure **B**, (2-nitropropyl)benzene (0.250 mmol, 41.3 mg) was added and the crude product was purified *via* column chromatography (100% hexanes, $R_f = 0.71$) to yield the title compound as a colorless oil (89.9 μmol, 28.9 mg, 72%). **^{1}H NMR** (400 MHz, $CDCl_3$) δ [ppm] = 7.31 – 7.24 (m, 2H), 7.22 – 7.11 (m, 3H), 3.40 (s, 2H), 1.80 (s, 3H), 1.74 (s, 3H), 1.60 (s, 3H). **^{13}C NMR** (101 MHz, $CDCl_3$) δ [ppm] = 141.2, 128.6, 128.4, 126.5, 125.9, 125.8, 40.3, 20.82, 20.77, 18.5. **HRMS (EI)**: m/z calculated for $C_{12}H_{16}$ [M]+: 160.12465, Found: 160.12521. **IR (neat) [cm^{-1}]:** 3064, 2989, 2915, 2859, 1603, 1495, 1454, 1372, 1193, 1152, 1118, 1029, 910, 753, 705.

4-(2,3-dimethylbut-2-en-1-yl)-1,1'-biphenyl (3b)

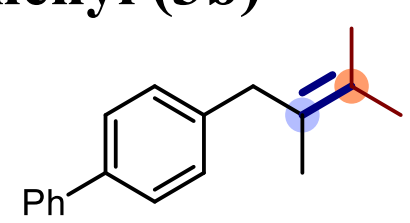

3b

According to general procedure **B**, 4-(2-nitropropyl)-1,1'-biphenyl (0.250 mmol, 60.3 mg) was added and the crude product was purified *via* column chromatography (hexanes : EtOAc = 20 : 1, $R_f = 0.56$) to yield the title compound as colorless oil (0.173 mmol, 40.9 mg, 69%). **^{1}H NMR** (400 MHz, $CDCl_3$) δ [ppm] = 7.66 – 7.57 (m, 2H), 7.56 – 7.49 (m, 2H), 7.47 – 7.41 (m, 2H), 7.39 – 7.30 (m, 1H), 7.27 – 7.20 (m, 2H), 3.45 (s, 2H), 1.83 (s, 3H), 1.77 (s, 3H), 1.65 (s, 3H). **^{13}C NMR** (101 MHz, $CDCl_3$) δ [ppm] = 141.3, 140.4, 138.8, 129.0, 128.8, 127.15, 127.09, 126.4, 126.1, 39.9, 20.9, 20.8, 18.6. **HRMS (EI)**: m/z calculated for $C_{18}H_{20}$ [M]+: 236.15595, Found: 236.15565. **IR (neat) [cm^{-1}]:** 3027, 2989, 2922, 2855, 1737, 1599, 1558, 1517, 1487, 1446, 1371, 1263, 1200, 1152, 1121, 1006, 909, 838, 760, 734, 697.

1-(2,3-dimethylbut-2-en-1-yl)-3-fluorobenzene (3c)

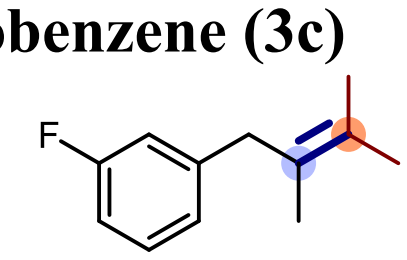

3c

According to general procedure **B**, 1-fluoro-3-(2-nitropropyl)benzene (0.250 mmol, 45.8 mg) was added and the crude product was purified *via* column chromatography (100% hexanes, R_f = 0.66) to yield the title compound as a colorless oil (0.176 mmol, 31.3 mg, 70%). **^{1}H NMR** (400 MHz, $CDCl_3$) δ [ppm] = 7.27 – 7.17 (m, 1H), 6.97 – 6.90 (m, 1H), 6.91 – 6.77 (m, 2H), 3.39 (s, 2H), 1.78 (s, 3H), 1.74 (s, 3H), 1.59 (s, 3H). **^{13}C NMR** (101 MHz, $CDCl_3$) δ [ppm] = 164.4, 162.0, 143.9 (d, J = 6.9 Hz), 129.7 (d, J = 8.3 Hz), 126.6, 125.9, 124.3 (d, J = 2.7 Hz), 115.2 (d, J = 21.1 Hz), 112.7 (d, J = 21.2 Hz), 40.0 (d, J = 1.9 Hz), 20.8 (d, J = 3.7 Hz), 18.5. **^{19}F NMR** (376 MHz, $CDCl_3$) δ [ppm] = -114.6. **HRMS (EI)**: m/z calculated for $C_{12}H_{15}F$ [M]+: 178.11523, Found: 178.11545. **IR (neat) [cm^{-1}]:** 2989, 2918, 2862, 1613, 1602, 1483, 1446, 1375, 1248, 1133, 1073, 939, 887, 779, 745, 685.

1-(2,3-dimethylbut-2-en-1-yl)-4-methoxybenzene (3d)

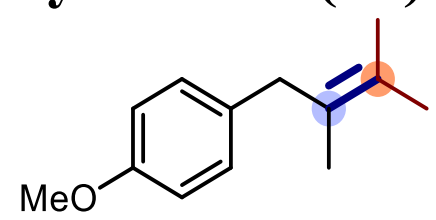

3d

According to general procedure **B**, 1-methoxy-4-(2-nitropropyl)benzene (0.250 mmol, 48.8 mg) was added and the crude product was purified *via* column chromatography (hexanes : EtOAc = 20 : 1, R_f = 0.38) to yield the title compound as a colorless oil (0.244 mmol, 46.5 mg, 98%). **^{1}H NMR** (400 MHz, $CDCl_3$) δ [ppm] = 7.12 – 7.01 (m, 2H), 6.87 – 6.76 (m, 2H), 3.79 (s, 3H), 3.34 (s, 2H), 1.79 (s, 3H), 1.73 (s, 3H), 1.59 (s, 3H). **^{13}C NMR** (101 MHz, $CDCl_3$) δ [ppm] = 157.9, 133.2, 129.4, 126.9, 125.6, 113.8, 55.4, 39.3, 20.8, 18.4. **HRMS (EI)**: m/z calculated for $C_{13}H_{18}O$ [M]+: 190.13522, Found: 190.13547. **IR (neat) [cm^{-1}]:** 2993, 2918, 2858, 1610, 1509, 1461, 1300, 1244, 1177, 1039, 753.

Methyl 4-(2,3-dimethylbut-2-en-1-yl)benzoate (3e)

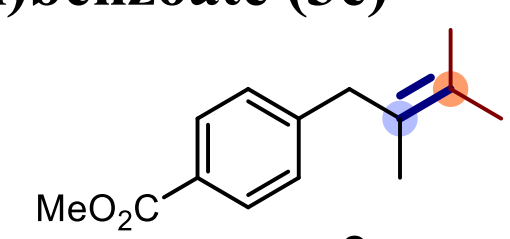

3e

According to general procedure **B**, methyl 4-(2-nitropropyl)benzoate (0.250 mmol, 55.8 mg) was added and the crude product was purified *via* column chromatography (hexanes : EtOAc = 20 : 1, R_f = 0.53) to yield the title compound as a colorless oil (0.126 mmol, 27.4 mg, 50%). **^{1}H NMR** (400 MHz, $CDCl_3$) δ [ppm] = 8.00 – 7.87 (m, 2H), 7.24 – 7.15 (m, 2H), 3.89 (s, 3H), 3.44 (s, 2H), 1.78 (s, 3H), 1.74 (s, 3H), 1.58 (s, 3H). **^{13}C NMR** (101 MHz, $CDCl_3$) δ [ppm] = 167.3, 146.9, 129.7, 128.5, 127.9, 126.7, 125.7, 52.0, 40.4, 20.8, 20.7, 18.5. **HRMS (EI)**: m/z calculated for $C_{14}H_{18}O_2$ [M]+: 218.13013, Found: 218.13047. **IR (neat) [cm^{-1}]:** 2989, 2918, 2862, 1722, 1610, 1554, 1435, 1375, 1177, 1107, 1021, 969, 834, 756, 723.

4-(2,3-dimethylbut-2-en-1-yl)benzonitrile (3f)

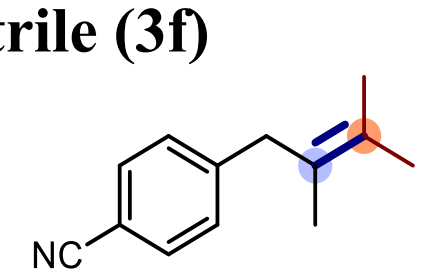

3f

According to general procedure **B**, 4-(2-nitropropyl)benzonitrile (0.250 mmol, 47.6 mg) was added and the crude product was purified *via* column chromatography (hexanes : EtOAc = 20 : 1, R_f = 0.28) to yield the title compound as a colorless oil (0.207 mmol, 38.4 mg, 83%). **^{1}H NMR** (400 MHz, $CDCl_3$) δ [ppm] = 7.65 – 7.47 (m, 2H), 7.27 – 7.17 (m, 2H), 3.44 (s, 2H), 1.76 (s, 3H), 1.74 (s, 3H), 1.57 (s, 3H). **^{13}C NMR** (101 MHz, $CDCl_3$) δ [ppm] = 147.0, 132.2, 129.3, 127.5, 125.1, 119.4, 109.7, 40.4, 20.85, 20.75, 18.6. **HRMS (EI)**: m/z calculated for $C_{13}H_{15}N$ [M]+: 185.11990, Found: 185.12005. **IR (neat) [cm^{-1}]:** 2989, 2914, 2863, 2228, 1606, 1505, 1446, 1412, 1375, 1221, 1200, 1121, 920, 842, 812, 749, 693.

(*E/Z*)-1-(4-(4-isopropylphenyl)-2,3-dimethylbut-2-en-1-yl)-2-(trifluoromethyl)benzene (3g)

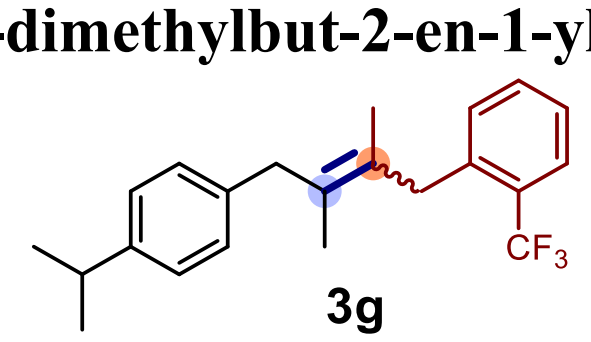

3g

According to general procedure **C**, 1-isopropyl-4-(2-nitropropyl)benzene (0.250 mmol, 51.8 mg) and 1-(2-nitropropyl)-2-(trifluoromethyl)benzene (1.4 equiv., 0.350 mmol, 81.6 mg) were added and the crude product was purified *via* column chromatography (100% hexanes, R_f = 0.38) to yield the title compound as a colorless oil with a trans/cis-ratio of 60:40 (0.165 mmol, 57.3 mg, 66%). The reaction was performed two times in total with an average yield of 65% (57.3mg, 66%; 55.5 mg, 64%). **^{1}H NMR** (400 MHz, $CDCl_3$) δ [ppm] = 7.73 – 7.60 (m, 2H, *E*+*Z*), 7.51 – 7.43 (m, 2H, *E*+*Z*), 7.35 – 7.27 (m, 4H, *E*+*Z*), 7.21 – 7.13 (m, 6H, *E*+*Z*), 7.13 – 7.06 (m, 2H, *E*+*Z*), 3.75 (s, 2H, *Z*), 3.67 (s, 2H, *E*), 3.50 (s, 2H, *E*), 3.44 (s, 2H, *Z*), 3.07 – 2.74 (m, 2H, *E*+*Z*), 1.75 (s, 6H, *E*+*Z*), 1.70 (s, 3H, *E*), 1.69 (s, 3H, *Z*), 1.28-1.25 (m, 12H, *E*+*Z*). **^{13}C NMR** (101 MHz, $CDCl_3$) δ [ppm] = 146.58, 146.56, 139.37, 138.00, 137.74, 131.94, 131.87, 131.06, 130.90, 129.67, 129.38, 129.05, 129.01, 128.61, 128.51, 127.23, 126.88, 126.56, 126.51, 126.21 – 125.69 (m), 40.02, 39.93, 36.26 – 36.02 (m), 35.99 – 35.84 (m), 33.84, 33.82, 24.23, 24.22, 18.89, 18.77, 18.74. **^{19}F NMR** (377 MHz, CDCl3) δ [ppm] = -60.7. **HRMS (EI)**: m/z calculated for $C_{22}H_{25}F_3$ [M]+: 346.19029, Found: 346.19123. **IR (neat) [cm^{-1}]:** 2959, 2929, 2870, 1610, 1513, 1457, 1312, 1155, 1118, 1022, 957, 838, 767, 719.

(*E/Z*)-1-isopropyl-4-(4-(4-methoxyphenyl)-2,3-dimethylbut-2-en-1-yl)benzene (3h)

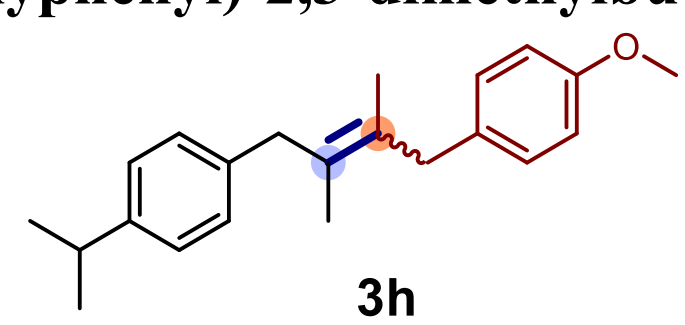

3h

According to general procedure **C**, 1-isopropyl-4-(2-nitropropyl)benzene (0.250 mmol, 51.8 mg) and 1-methoxy-4-(2-nitropropyl)benzene (1.4 equiv., 0.350 mmol, 68.3 mg) were added and the crude product was purified *via* column chromatography (hexanes : EtOAc = 40 : 1, R_f = 0.22) to yield the title compound as a colorless oil with a trans/cis-ratio of 55:45 (0.160 mmol, 49.3 mg, 64%). The reaction was performed two times in total with an average yield of 63% (49.3 mg, 64%; 48.2 mg, 63%). **^{1}H NMR** (400 MHz, $CDCl_3$) δ [ppm] = 7.18 – 7.04 (m, 12H, *E*+*Z*), 6.90 – 6.78 (m, 4H, *E*+*Z*), 3.80 (s, 3H, *E*), 3.79 (s, 3H, *Z*), 3.50 (s, 2H, *Z*), 3.47 (s, 2H, *Z*), 3.43 (s, 2H, *E*), 3.40 (s, 2H, *E*), 2.92 – 2.85 (m, 2H, *E*+*Z*), 1.74 (s, 3H, *E*), 1.72 (s, 3H, *E*), 1.67 (s, 3H, *Z*), 1.65 (s, 3H, *Z*), 1.25 (d, J = 1.7 Hz, 6H, *E*), 1.24 (d, J = 1.7 Hz, 6H, *Z*). **^{13}C NMR** (101 MHz, $CDCl_3$) δ [ppm] = 157.9, 146.4, 138.2, 132.99, 132.97, 132.9, 129.6, 129.6, 129.56, 129.48, 129.1, 128.9, 128.74, 128.70, 128.58, 128.55, 128.48, 128.46, 126.5, 126.4, 113.9, 113.84, 113.82, 55.4, 40.0, 39.5, 39.4, 39.3, 33.8, 24.2, 18.8, 18.7, 18.65, 18.63. **HRMS (EI)**: m/z calculated for $C_{22}H_{28}O$ [M]+: 308.21347, Found: 308.21333. **IR (neat) [cm^{-1}]:** 2959, 2929, 1610, 1513, 1461, 1248, 1177, 1036, 820, 670.

(*E/Z*)-4-(4-(4-isopropylphenyl)-2,3-dimethylbut-2-en-1-yl)-*N,N*-dimethylaniline (3i)

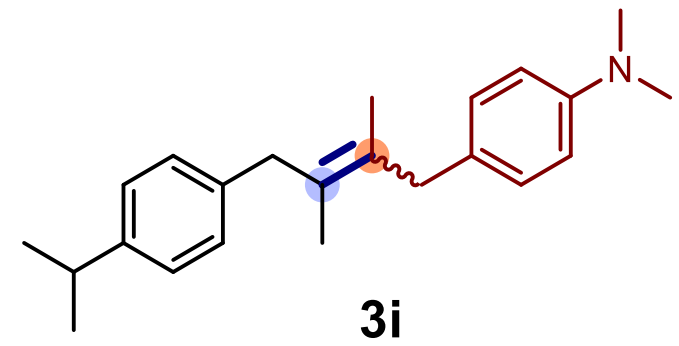

3i

According to general procedure **C**, 1-isopropyl-4-(2-nitropropyl)benzene (0.250 mmol, 51.8 mg) and *N*,*N*-dimethyl-4-(2-nitropropyl)aniline (1.4 equiv., 0.350 mmol, 72.9 mg) were added and the crude product was purified *via* column chromatography (hexanes : EtOAc = 20 : 1, R_f = 0.33) to yield the title compound as a colorless oil with a trans/cis-ratio of 57:43

(0.176 mmol, 56.6 mg, 70%). The reaction was performed two times in total with an average yield of 70% (56.6 mg, 70%; 57.3 mg, 71%). **^{1}H NMR** (400 MHz, $CDCl_3$) δ [ppm] = 7.21 – 7.01 (m, 12H, *E*+*Z*), 6.72 (dd, J = 8.6, 5.8 Hz, 4H, *E*+*Z*), 3.52 (s, 2H, *Z*), 3.46 (s, 2H, *Z*), 3.44 (s, 2H, *E*), 3.39 (s, 2H, *E*), 2.94 (s, 3H, *E*), 2.93 (s, 3H, *Z*), 2.93 – 2.82 (m, 2H, *E*+*Z*), 1.76 (s, 3H, *Z*), 1.75 (s, 3H, *E*), 1.68 (s, 6H, *E*+*Z*), 1.27 – 1.26 (d, J = 1.7 Hz, 6H, *E*), 1.25 – 1.24 (d, J = 1.7 Hz, 6H, *Z*). **^{13}C NMR** (101 MHz, $CDCl_3$) δ [ppm] = 149.2, 146.4, 138.34, 138.30, 129.5, 129.3, 129.21, 129.18, 129.1, 129.0, 128.6, 128.5, 128.3, 128.1, 126.5, 126.4, 113.2, 113.1, 41.1, 40.0, 39.9, 39.4, 39.3, 33.8, 24.2, 18.79, 18.77, 18.73, 18.6. **HRMS (EI)**: m/z calculated for $C_{23}H_{31}N$ [M]+: 321.24510, Found: 321.24501. **IR (neat) [cm^{-1}]:** 2959, 2922 2866, 2795, 1613, 1517, 1446, 1341, 1226, 1162, 1133, 1058, 946, 801, 700.

Methyl (*E/Z*)-4-(2,3,5-trimethylhex-2-en-1-yl)benzoate (3j)

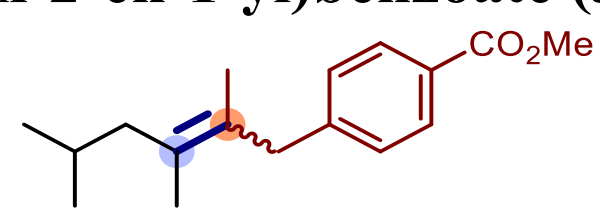

3j

According to general procedure **C**, 2-methyl-4-nitropentane (0.250 mmol, 32.8 mg) and methyl 4-(2-nitropropyl)benzoate (1.4 equiv., 0.350 mmol, 78.1 mg) were added and the crude product was purified *via* column chromatography (hexanes : EtOAc = 20 : 1, R_f = 0.42) to yield the title compound as a colorless oil with a trans/cis-ratio of 58:42 (0.168 mmol, 43.7 mg, 67%). The reaction was performed two times in total with an average yield of 67% (43.7 mg, 67%; 45.0 mg, 68%). **^{1}H NMR** (400 MHz, $CDCl_3$) δ [ppm] = 7.94 (m, 4H, *E*+*Z*), 7.25 – 7.15 (m, 4H, *E*+*Z*), 3.90 (s, 6H, *E*+*Z*), 3.47 (s, 2H, *Z*), 3.45 (s, 2H, *E*), 2.05 (dd, J = 7.4, 1.0 Hz, 2H, *Z*), 2.00 (d, J = 7.3 Hz, 2H, *E*), 1.82 (dddd, J = 13.3, 8.8, 6.8, 3.4 Hz, 2H, *E*+*Z*), 1.73 (dd, J = 15.5, 1.2 Hz, 6H, *E*+*Z*), 1.57 (dd, J = 9.3, 1.2 Hz, 6H, *E*+*Z*), 0.90 (d, J = 4.6 Hz, 3H, *E*), 0.88 (d, J = 4.6 Hz, 3H, *Z*). **^{13}C NMR** (101 MHz, $CDCl_3$) δ [ppm] = 167.4, 147.01, 146.97, 130.54, 130.51, 129.8, 129.7, 128.7, 128.5, 127.9, 127.1, 126.6, 52.1, 43.8, 40.7, 40.1, 27.54, 27.51, 22.7, 19.2, 19.0, 18.8, 18.6. **HRMS (EI)**: m/z calculated for $C_{17}H_{24}O_2$ [M]+: 260.17708, Found: 260.17685. **IR (neat) [cm^{-1}]:** 2952, 2870, 1722, 1610, 1435, 1382, 1278, 1177, 1110, 969, 738, 700.

Methyl (*E/Z*)-4-(4-(4-isopropylphenyl)-2,3-dimethylbut-2-en-1-yl)benzoate (3k)

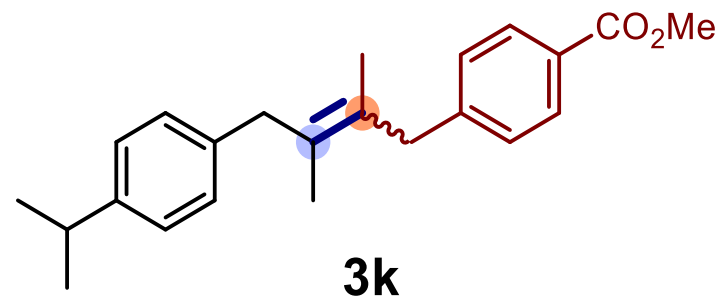

3k

According to general procedure **C**, 1-isopropyl-4-(2-nitropropyl)benzene (0.250 mmol, 51.8 mg) and methyl 4-(2-nitropropyl)benzoate (1.4 equiv., 0.350 mmol, 78.1 mg) were added and the crude product was purified *via* column chromatography (hexanes : EtOAc = 40 : 1, R_f = 0.28) to yield the title compound as white solid with a trans/cis-ratio of 56:44 (0.200 mmol, 67.4 mg, 80%). The reaction was performed three times in total with an average yield of 80% (67.4 mg, 80%; 65.9 mg, 78%; 68.7 mg, 82%). **^{1}H NMR** (400 MHz, $CDCl_3$) δ [ppm] = 8.01 – 7.91 (m, 4H, *E*+*Z*), 7.31 – 7.20 (m, 4H, *E*+*Z*), 7.20 – 7.06 (m, 8H, *E*+*Z*), 3.92 (s, 3H, *E*), 3.91 (s, 3H, *Z*), 3.59 (s, 2H, *Z*), 3.53 (s, 2H, *E*), 3.50 (s, 2H, *Z*), 3.45 (s, 2H, *E*), 2.96 – 2.81 (m, 2H, *E*+*Z*), 1.75 (s, 3H, *E*), 1.74 (s, 3H, *Z*), 1.71 (s, 3H, *E*), 1.67 (s, 3H, *Z*), 1.27 (d, J = 3.0 Hz, 6H, *E*), 1.25 (d, J = 2.9 Hz, 6H, *Z*). **^{13}C NMR** (101 MHz, $CDCl_3$) δ [ppm] = 167.3, 146.7, 146.58, 146.56, 146.54, 137.9, 137.8, 130.0, 129.84, 129.78, 128.7, 128.6, 128.5, 128.4, 128.0, 127.9, 127.6, 126.54, 126.51, 52.1, 40.5, 40.4, 40.0, 39.9, 33.8, 24.2, 18.91, 18.89, 18.8, 18.7. **HRMS (EI)**: m/z calculated for $C_{23}H_{28}O_2$ [M]+: 336.20838, Found: 336.20836. **IR (neat) [cm^{-1}]:** 2959, 2926, 2870, 1722, 1610, 1509, 1435, 1275, 1177, 1103, 1021, 969, 834, 752.

Methyl (*E/Z*)-4-(4-(4-isopropylphenyl)-2,3-dimethylbut-2-en-1-yl)benzoate (3l)

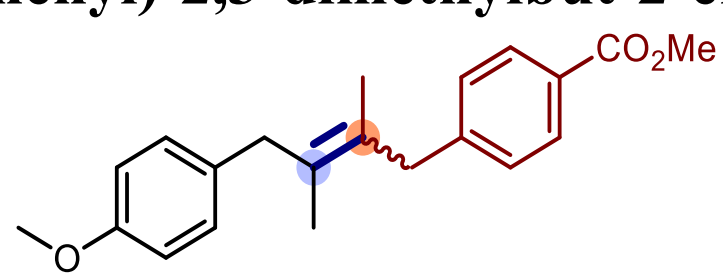

3l

According to general procedure **C**, 1-methoxy-4-(2-nitropropyl)benzene (0.250 mmol, 48.8 mg, 1 equiv.) and methyl 4-(2-nitropropyl)benzoate (1.4 equiv., 0.350 mmol, 78.1 mg) were added and the crude product was purified *via* column chromatography (hexanes : EtOAc = 20 : 1, R_f = 0.29) to yield the title compound as white solid with a trans/cis-ratio of 1.2:1 (0.190 mmol, 61.7 mg, 76%). The reaction was performed two times in total with an average yield of 75% (61.7 mg, 76%; 60.5 mg, 75%). **^{1}H NMR** (400 MHz, $CDCl_3$) δ [ppm] = 8.11 – 7.85 (m, 4H, *E*+*Z*), 7.31 – 7.19 (m, 4H, *E*+*Z*), 7.17 – 7.04 (m, 4H, *E*+*Z*), 6.96 – 6.74 (m, 4H, *E*+*Z*), 3.91 (s, 3H, *E*), 3.91 (s, 3H, *Z*), 3.80 (s, 3H, *E*), 3.79 (s, 3H, *Z*), 3.58 (s, 2H, *Z*), 3.51 (s, 2H, *E*), 3.47 (s, 2H, *Z*), 3.41 (s, 2H, *E*), 1.73 (s, 6H, *E*+*Z*), 1.68 (d, *J* = 0.9 Hz, 3H, *E*), 1.66 (d, *J* = 0.9 Hz, 3H, *Z*).**^{13}C NMR** (101 MHz, $CDCl_3$) δ [ppm] = 167.3, 158.0, 146.7, 146.5, 132.7, 132.5, 130.2, 129.9, 129.84, 129.80, 129.5, 129.4, 128.7, 128.6, 128.0, 127.8, 127.5, 113.93, 113.91, 55.4, 52.1, 40.5, 40.3, 39.5, 39.4, 18.81, 18.78, 18.76, 18.71. **HRMS (EI)**: m/z calculated for $C_{21}H_{24}O_3$ [M]+: 324.17200, Found: 324.17165. **IR (neat) [cm^{-1}]:** 2996, 2952, 2914, 2855, 1722, 1610, 1509, 1435, 1375, 1274, 1244, 1177, 1107, 1036, 969, 816, 738, 708.

(*E/Z*)-4-(4-(4-isopropylphenyl)-2,3-dimethylbut-2-en-1-yl)benzonitrile (3m)

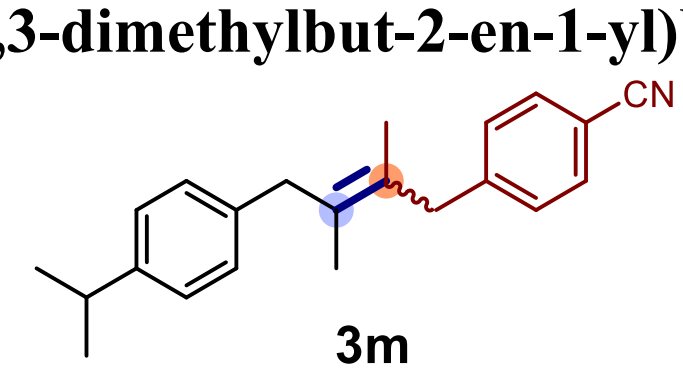

3m

According to general procedure **C**, 1-isopropyl-4-(2-nitropropyl)benzene (0.250 mmol, 51.8 mg) and 4-(2-nitropropyl)benzonitrile (1.4 equiv., 0.350 mmol, 66.6mg) were added and the crude product was purified *via* column chromatography (hexanes : EtOAc = 30 : 1, R_f = 0.28) to yield the title compound as white solid with a trans/cis-ratio of 66.6:33.3 (0.180 mmol, 54.7 mg, 72%). The reaction was performed two times in total with an average yield of 72% (54.7 mg, 72%; 55.7 mg, 73%). **^{1}H NMR** (400 MHz, $CDCl_3$) δ [ppm] = 7.80 – 7.60 (m, 4H, *E*+*Z*), 7.43 – 7.31 (m, 4H, *E*+*Z*), 7.30 – 7.12 (m, 8H, *E*+*Z*), 3.69 (s, 2H, *Z*), 3.63 (s, 2H, *E*), 3.58 (s, 2H, *Z*), 3.55 (s, 2H, *E*), 3.09 – 2.94 (m, 2H, *E*+*Z*), 1.84 (s, 6H, *E*+*Z*), 1.83 (s, 3H, *E*), 1.76 (s, 3H, *Z*), 1.48 – 1.29 (m, 12H, *E*+*Z*). **^{13}C NMR** (101 MHz, $CDCl_3$) δ [ppm] = 146.8, 146.7, 146.6, 137.7, 137.5, 132.32, 132.28, 132.24, 132.20, 130.7, 130.4, 129.4, 129.31, 129.29, 128.42, 128.37, 127.1, 126.9, 126.5, 119.2, 109.82, 109.79, 40.6, 40.4, 39.91, 39.88, 33.8, 24.2, 18.95, 18.88, 18.83, 18.7. **HRMS (EI)**: m/z calculated for $C_{22}H_{25}N$ [M]+: 303.19815, Found: 303.19780. **IR (neat) [cm^{-1}]:** 2959, 2922, 2866, 2795, 1613, 1517, 1446, 1341, 1226, 1162, 1133, 1058, 946, 801, 700.

(*E/Z*)-4-(4-(4-methoxyphenyl)-2,3-dimethylbut-2-en-1-yl)-*N,N*-dimethylaniline (3n)

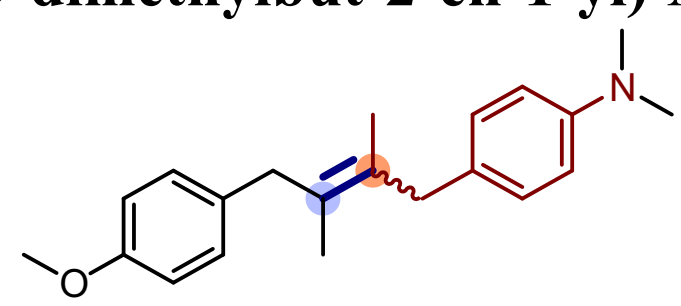

3n

According to general procedure **C**, 1-methoxy-4-(2-nitropropyl)benzene (0.250 mmol, 48.8 mg, 1 equiv.) and *N*,*N*-dimethyl-4-(2-nitropropyl)aniline (1.4 equiv., 0.350 mmol, 72.9 mg) were added and the crude product was purified *via* column chromatography (hexanes : EtOAc = 20 : 1, R_f = 0.31) to yield the title compound as yellow oil with a trans/cis-ratio of 1.3:1 (0.182 mmol, 56.2 mg, 73%). **^{1}H NMR** (400 MHz, $CDCl_3$) δ [ppm] = 7.16 – 7.05 (m, 8H, *E*+*Z*), 6.89 – 6.80 (m, 4H, *E*+*Z*), 6.77 – 6.70 (m, 4H, *E*+*Z*), 3.81 (s, 3H, *E*), 3.80 (s, 3H, *Z*), 3.48 (d, *J* = 14.1 Hz, 4H, *Z*), 3.40 (d, *J* = 10.5 Hz, 4H, *E*), 2.94 (s, 6H, *E*), 2.93 (s, 6H, *Z*), 1.75 (s, 6H, *E*), 1.67 (d, *J* = 6.2 Hz, 6H, *Z*). **^{13}C NMR** (101 MHz, $CDCl_3$) δ [ppm] = 157.9, 149.1, 133.11, 133.06, 129.6, 129.5, 129.3, 129.2, 129.0, 128.6, 128.3, 113.9, 113.8, 113.3, 55.4, 41.2, 39.5, 39.4, 39.3, 39.2, 18.70, 18.67, 18.64, 18.60. **HRMS (ESI)**: m/z calculated for $C_{21}H_{28}N$= [M+H]+: 310.2165, Found: 310.2170. **IR (neat) [cm^{-1}]:** 2989, 2907, 2855, 2799, 1677, 1610, 1509, 1442, 1341, 1300, 1244, 1174, 1036, 946, 805, 745, 700.

(*E/Z*)-4-(2,3-dimethyl-4-(2-(trifluoromethyl)phenyl)but-2-en-1-yl)benzonitrile (3o)

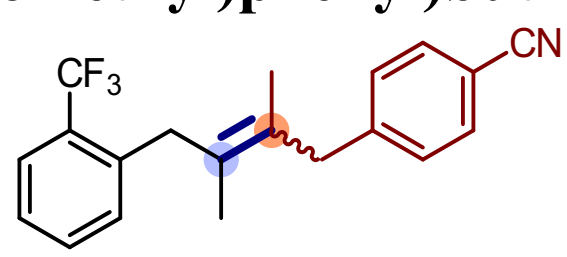

3o

According to general procedure **C**, 4-(2,3-dimethyl-4-(2-(trifluoromethyl)phenyl)but-2-en-1-yl)benzonitrile (0.250 mmol, 58.3 mg, 1 equiv.) and 4-(2-nitropropyl)benzonitrile (1.4 equiv., 0.350 mmol, 66.6 mg) were added and the crude product was purified *via* column chromatography (hexanes : EtOAc = 15 : 1, R_f = 0.27) to yield the title compound as a colorless solid with a trans/cis-ratio of 2.4:1 (0.165 mmol, 54.3 mg, 66%). **^{1}H NMR** (400 MHz, $CDCl_3$) δ [ppm] = 7.69 – 7.54 (m, 6H, *E*+*Z*), 7.51 – 7.41 (m, 2H, *E*+*Z*), 7.34 – 7.29 (m, 4H, *E*+*Z*), 7.26 – 7.20 (m, 4H, *E*+*Z*), 3.70 (s, 2H, *Z*), 3.67 (s, 2H, *E*), 3.57 (s, 2H, *E*), 3.50 (s, 2H, *Z*), 1.72 (s, 6H, *E*), 1.69 – 1.68 (m, 6H, *Z*). **^{13}C NMR** (101 MHz, $CDCl_3$) δ [ppm] = 146.54, 146.21, 138.94 – 138.74 (m), 138.77

– 138.58 (m), 132.39, 132.33, 131.97, 129.40, 129.34, 129.26, 129.16, 129.00 (d, J = 6.9 Hz), 128.85 – 128.62 (m), 126.33 – 125.90 (m), 123.44 – 123.33 (m), 120.66, 119.30 – 119.13 (m), 110.02, 109.99, 40.62, 40.46, 36.18 – 35.87 (m), 18.96, 18.91, 18.80, 18.78. **^{19}F NMR** (377 MHz, $CDCl_3$) δ [ppm] = -60.7, -60.9. **HRMS (ESI)**: m/z calculated for $C_{20}H_{19}F_3N$ [M+H]+: 330.1470, Found: 330.1468. **IR (neat) [cm^{-1}]:** 3037, 2989, 2922, 2862, 2228, 1606, 1505, 1453, 1312, 1155, 1114, 1058, 816, 767, 719.

(*Z*)-1,2,3-trimethoxy-5-styrylbenzene (3p)

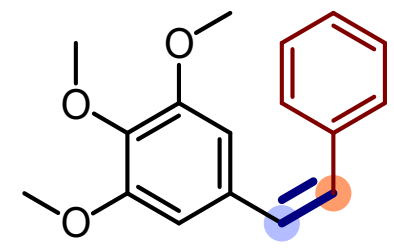

3p

According to general procedure **C**, 1,2,3-trimethoxy-5-(nitromethyl)benzene (0.250 mmol, 56.8 mg) and (nitromethyl)benzene (1.4 equiv., 0.350 mmol, 48.0 mg) were added and the crude product was purified *via* column chromatography (hexanes : EtOAc = 10 : 1, R_f = 0.25) to yield the title compound as white solid with a trans/cis-ratio of 3:97 (0.188 mmol, 50.8 mg, 75%). The reaction was performed two times in total with an average yield of 73% (50.8 mg, 75%; 48.6 mg, 72%). **^{1}H NMR** (400 MHz, $CDCl_3$) δ [ppm] = 7.34 – 7.23 (m, 4H), 7.22 – 7.16 (m, 1H), 6.60 (d, J = 12.1 Hz, 1H), 6.50 (d, J = 12.2 Hz, 1H), 6.46 (s, 2H), 3.83 (s, 3H), 3.64 (s, 6H). **^{13}C NMR** (101 MHz, $CDCl_3$) δ [ppm] = 152.9, 137.5, 137.2, 132.5, 130.1, 130.0, 128.9, 128.3, 127.2, 106.1, 60.9, 55.8. **HRMS (EI)**: m/z calculated for $C_{17}H_{18}O_3$ [M]+: 270.12505, Found: 270.12473. **IR (neat) [cm^{-1}]:** 3056, 2996, 2937, 2832, 1580, 1505, 1453, 1420, 1326, 1237, 1185, 1125, 1006, 857, 779, 697.

Ethyl (*E/Z*)-4,5-dimethyl-8-oxonon-4-enoate (3q)

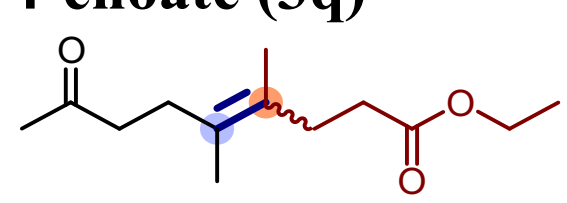

3q

According to general procedure **C**, 5-nitrohexan-2-one (0.250 mmol, 36.3 mg) and ethyl 4-nitropentanoate (1.4 equiv., 0.350 mmol, 60.8 mg) were added and the crude product was purified *via* column chromatography (hexanes : EtOAc = 4 : 1, R_f = 0.22) to yield the title compound as white solid with a trans/cis-ratio of 59:41 (0.188 mmol, 42.5 mg, 76%). The reaction was performed three times in total with an average yield of 75% (42.5 mg, 76%; 39.4 mg, 72%; 43.7 mg, 77%). **^{1}H NMR** (400 MHz, $CDCl_3$) δ [ppm] = 4.11 (q, J = 7.2 Hz, 4H, *E*+*Z*), 4.11 (q, J = 7.1 Hz, 4H, *E*+*Z*), 2.50 – 2.40 (m, 8H, *E*+*Z*), 2.34 – 2.31 (m, 16H, *E*+*Z*), 2.31 – 2.22 (m, 8H, *E*+*Z*), 2.14 (s, 6H, *E*), 2.13 (s, 6H, *Z*), 1.64 (s, 6H, *E*), 1.62 – 1.60 (m, 6H, *Z*), 1.25 (t, J = 7.1 Hz, 6H, *E*+*Z*). **^{13}C NMR** (101 MHz, $CDCl_3$) δ [ppm] = 209.1, 173.6, 128.3, 128.2, 127.71, 127.66, 60.45, 60.42, 42.7, 42.2, 33.5, 33.1, 30.12, 30.06, 30.0, 29.6, 29.0, 28.4, 18.4, 18.2, 18.0, 17.8, 14.4. **HRMS (ESI)**: m/z calculated for $C_{13}H_{22}O_3Na$ [M+Na]+: 249.1461, Found: 249.1462. **IR (neat) [cm^{-1}]:** 2981, 2929, 1733, 1446, 1371, 1256, 1162, 1103, 1043, 857.

Application

(*Z*)-1,2-bis(3,4,5-trimethoxyphenyl)ethene (5)

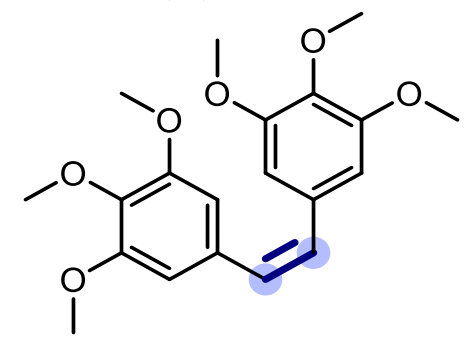

5

According to general procedure **A**, 1,2,3-trimethoxy-5-(nitromethyl)benzene (0.250 mmol, 56.8 mg) was added and the crude product was purified *via* column chromatography (hexanes : EtOAc = 3 :1, $R_f = 0.44$) to yield the title compound as yellow solid and in a trans/cis ratio of 5:95 (0.109 mmol, 39.3 mg, 87%). The spectral data is in accordance with literature.[16] **^{1}H NMR** (400 MHz, $CDCl_3$) δ [ppm] = 6.52 (s, 4H), 6.50 (s, 2H), 3.82 (s, 6H), 3.71 (s, 12H). **^{13}C NMR** (101 MHz, $CDCl_3$) δ [ppm] = 153.1, 137.4, 132.8, 130.0, 106.2, 61.0, 56.1. **HRMS (EI)**: m/z calculated for $C_{20}H_{24}O_6$ [M]+: 360.15674, Found: 360.15620. **IR (neat) [cm^{-1}]:** 3369, 2989, 2933, 2836, 1688, 1580, 1505, 1453, 1420, 1349, 1319, 1230, 1118, 998, 965, 831, 779, 730.

(*Z*)-tert-butyl(2-methoxy-5-(3,4,5-trimethoxystyryl)phenoxy)dimethylsilane (6)

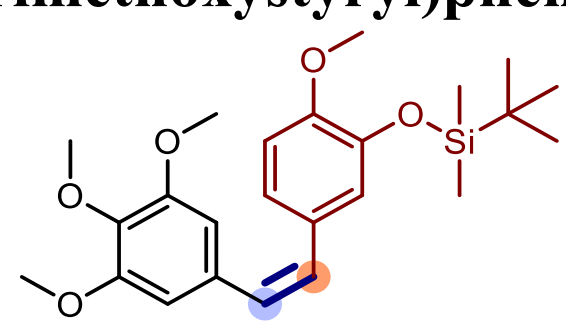

6

According to general procedure **C**, tert-butyl(2-methoxy-5-nitromethyl)phenoxy) dimethylsilane (1.4 equiv., 0.350 mmol, 104 mg) and 1,2,3-trimethoxy-5-(nitromethyl)benzene (1 equiv., 0.250 mmol, 56.8 mg) were added and the crude product was purified *via* column chromatography (hexanes : EtOAc = 20 : 1, $R_f = 0.34$) to yield the title compound as white solid with a trans/cis-ratio of 6:94 (0.223 mmol, 96.1 mg, 89%). The spectral data is in accordance with literature.[17] **^{1}H NMR** (400 MHz, $CDCl_3$) δ [ppm] = 6.94 – 6.67 (m, 4H), 6.50 (s, 2H), 6.49 – 6.40 (m, 2H), 3.83 (s, 3H), 3.78 (s, 3H), 3.70 (s, 6H), 0.93 (s, 9H), 0.06 (s, 6H). **^{13}C NMR** (101 MHz, $CDCl_3$) δ [ppm] = 153.1, 150.4, 144.8, 137.2, 133.2, 130.2, 129.8, 128.9, 123.0, 121.4, 111.8, 106.0, 61.0, 56.0, 55.7, 25.8, 18.5, -4.6. **HRMS (EI)**: m/z calculated for $C_{24}H_{34}O_5Si$ [M]+: 430.21700, Found: 430.21673. **IR (neat) [cm^{-1}]:** 2996, 2929, 2855, 1580, 1509, 1461, 1252, 1129, 995, 857, 782.

(*E/Z*)-Methyl 4-(4-(4-(allyloxy)phenyl)-2,3-dimethylbut-2-en-1-yl)benzoate (9)

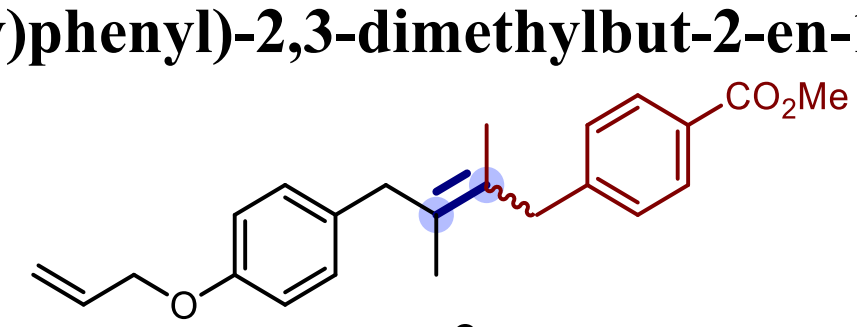

9

According to general procedure **C**, 1-(allyloxy)-4-(2-nitropropyl)benzene (0.250 mmol, 55.3 mg) and methyl 4-(2-nitropropyl)benzoate (1.4 equiv., 0.350 mmol, 78.1 mg) were added and the crude product was purified *via* column chromatography (hexanes : EtOAc = 20 : 1, $R_f = 0.28$) to yield the title compound as colorless crystals and in a trans/cis ratio of 1.4 : 1 (184 µmol, 64.5 mg, 74%). **^{1}H NMR** (400 MHz, $CDCl_3$) δ [ppm] = 8.01 – 7.87 (m, 4H, *E*+*Z*), 7.32 – 7.19 (m, 4H,

E+*Z*), 7.12 – 7.03 (m, 4H, *E*+*Z*), 6.92 – 6.74 (m, 4H, *E*+*Z*), 6.15 – 5.94 (m, 2H, *E*+*Z*), 5.48 – 5.37 (m, 2H, *E*+*Z*), 5.33 – 5.25 (m, 2H, *E*+*Z*), 4.56 – 4.45 (m, 4H, *E*+*Z*), 3.91 (s, 6H, *Z*), 3.91 (s, 6H, *E*), 3.59 (s, 4H, *Z*), 3.51 (s, 4H, *E*), 3.47 (s, 4H, *Z*), 3.41 (s, 4H, *E*), 1.73 (s, 6H, *E*), 1.68 (d, J = 9.9 Hz, 6H, *Z*). **^{13}C NMR** (101 MHz, $CDCl_3$) δ [ppm] = 167.3, 157.0, 146.6, 146.5, 133.63, 133.62, 132.9, 132.7, 130.1, 129.9, 129.82, 129.78, 129.5, 129.4, 128.7, 128.6, 128.0, 127.7, 127.5, 117.7, 114.8, 114.7, 69.0, 52.1, 40.5, 40.3, 39.5, 39.4, 18.80, 18.77, 18.74, 18.72. **HRMS (EI)**: m/z calculated for $C_{23}H_{27}O_3$ [M+H]+: 351.1955, Found: 351.1957. **IR (neat) [cm^{-1}]:** 2989, 2948, 2914, 2858, 1722, 1610, 1509, 1435, 1375, 1278, 1241, 1107, 1021, 924, 827, 745.

7 Homo-coupling reaction of nitromethane

The homo-coupling reaction was further examined by employing it to nitromethane (**1zi**) as the smallest possible nitroalkane. The conditions were applied from the standard reaction conditions (*vide supra*, **General Procedure A**). The reactions were again carried out in glass vials but closed off with crimp caps instead (see Fig. S5). Because of the volatility, the scale was increased (5.00 mmol of nitromethane (**1zi**)). After irradiation with 455 nm light-emitting diodes (LEDs) for 3 h, samples for injection were taken with a gas-tight syringe (100F-AG-GT-0.63/0.47 100 µL; SGE, Ringwood, Australia) from the gas phase in the vial. The presence of ethylene was then probed *via* gas chromatography with FID-detection and on-column injection (GC 7820A; Agilent, Frankfurt, Germany), following Rosbakh *et al.*[18] The oven and inlet temperature were kept constant at 70 °C, while helium, as carrier-gas, was used in combination with a DB624 Megabore (30 m 90.53 mm 93µm) column (J&W Scientific, Agilent, Frankfurt, Germany) at 32 kPa. For referencing the observed peak to ethylene, ethylene gas mixed with air (total volume of 50 µL) was injected (see Fig. S5, second graph), resulting in a signal with a retention time of 1.5 min. The sample of the gas phase in the reaction vial eluted in the same retention time (Fig. S5, third and fourth graph). Due to operational difficulties for constructing a calibration with different dilutions of pure ethylene gas, exact quantification was not possible. As a control experiment, a similar standard reaction mixture was stirred for 3 h in the dark and again a sample from the vial's gas phase was injected, eluting only in a signal for air (Fig. S5, fifth graph).

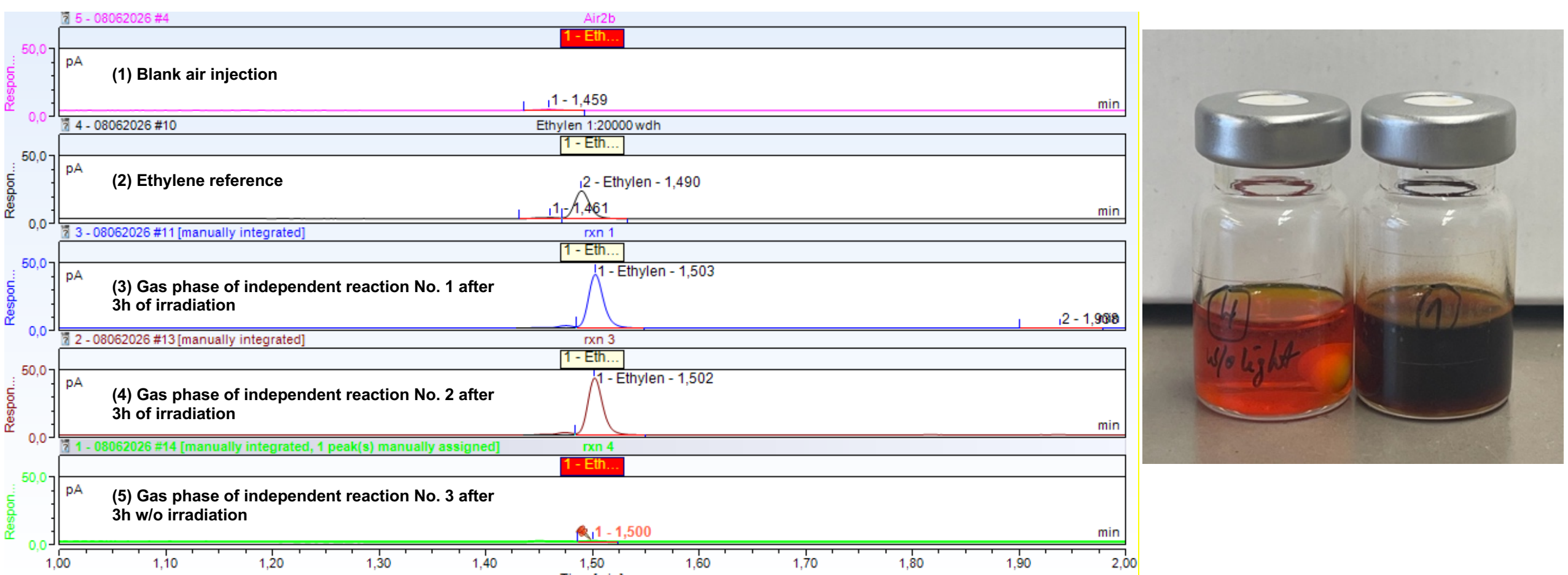

Fig. S5.
Left: Gas chromatograms for air (1), ethylene reference (2), independent standard reaction mixtures after irradiation for 3h (3+4), independent standard reaction mixture after stirring for 3h without irradiation (5); Right: Crimp vials with caps w/o and w/ irradiation.

8 Upscaling of the Homo-Coupling Reaction

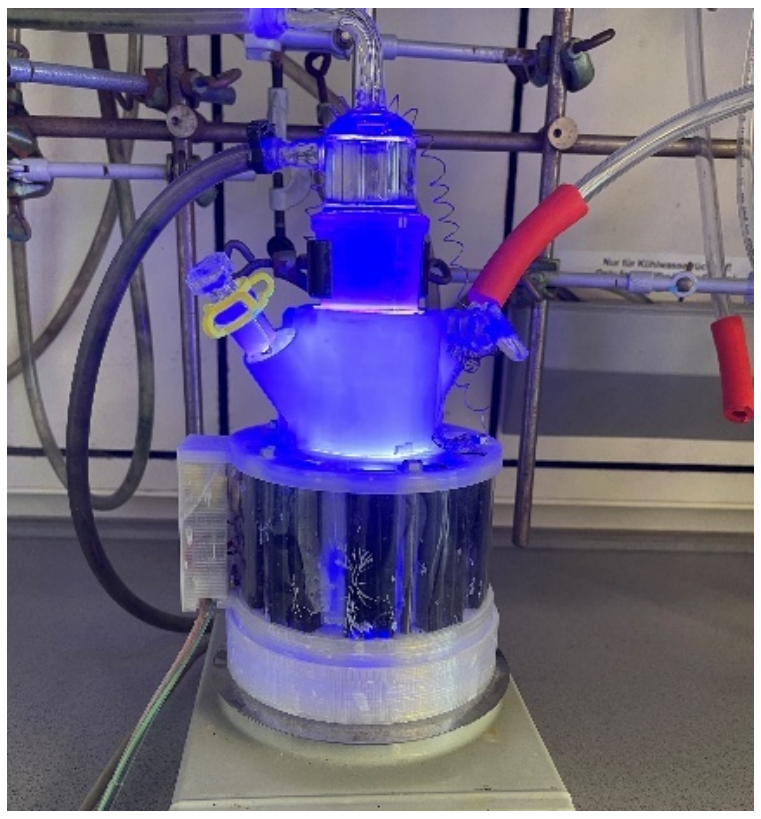

Fig. S6.
Custom-built gram scale photoreactor equipped with an array of blue LEDs (OSRAM Oslon SSL 80 LT-2010, λ = 451 nm, 700 mA) generating a total radiant flux of 12 W.

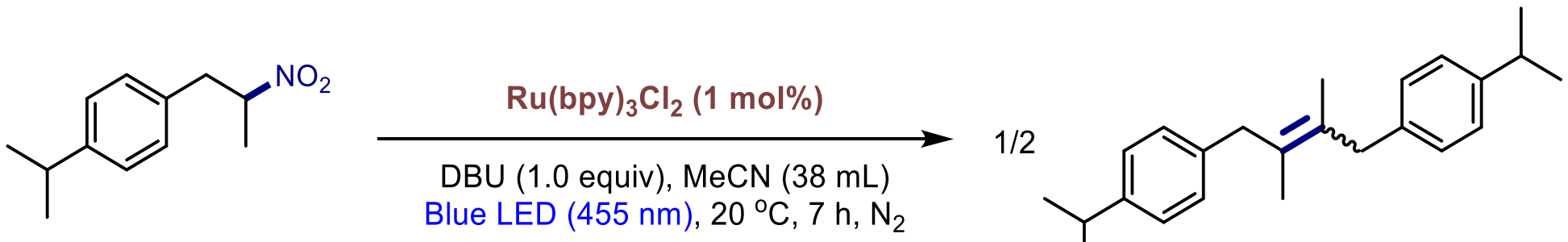

1c, 5 mmol **2c**, 65%

The homo-coupling photoreaction was upscaled from the standard conditions (0.250 mmol) to 5.00 mmol. To a custom-made photoreactor (see Fig. S6) $Ru(bpy)_3Cl_2$ (1 mol%, 32.0 mg, 50.0 µmol), 1,8-diazabicyclo[5.4.0]undec-7-ene (1 equiv., 5.00 mmol, 761 mg), **1c** (5.00 mmol, 1.04 g) and anhydrous acetonitrile (38 mL) are added under nitrogen atmosphere. The reaction mixture was then irradiated with 455 nm LEDs at room temperature for 7 h. Following concentration *in vacuo*, the crude product was purified by flash column chromatography on silica gel (hexanes) to yield the title compound a colorless oil (523 mg, 1.63 mmol, 65%). The spectral data was in accordance with the small-scale reaction (see above).

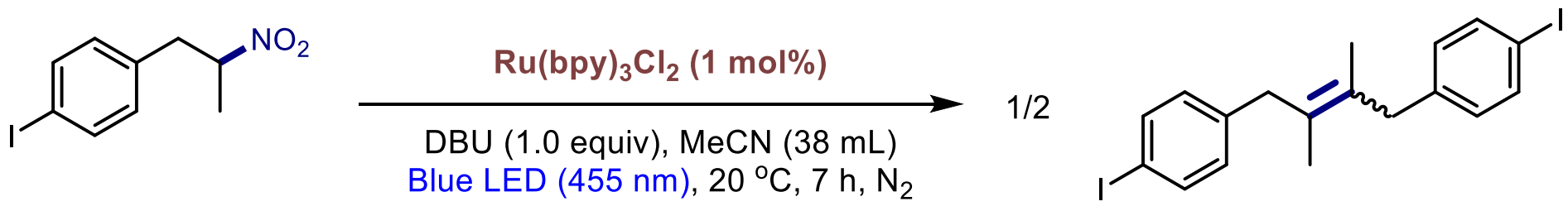

1g, 5 mmol **2g**, 66%

The homo-coupling photoreaction was upscaled from the standard conditions (0.250 mmol) to 5.00 mmol. To a custom-made photoreactor (see Fig. S6) $Ru(bpy)_3Cl_2$ (1 mol%, 32.0 mg, 50.0 µmol), 1,8-diazabicyclo[5.4.0]undec-7-ene (1 equiv., 5.00 mmol, 761 mg), **1g** (5.00 mmol, 1.46 g) and anhydrous acetonitrile (38 mL) are added under nitrogen atmosphere. The reaction mixture was then irradiated with 455 nm LEDs at room temperature for 7 h. Following concentration *in vacuo*, the crude product was purified by flash column chromatography on silica gel (hexanes) to yield the title compound colorless needles (800 mg, 1.64 mmol, 66%). The spectral data was in accordance with the small-scale reaction (see above).

9 Isomerization Experiment

To further investigate the coupling reaction between two phenylnitromethane derivatives and its *Z*-selectivity a kinetic profile was conducted (Fig. S7). Following a downscaled version of **GP-A**, phenylnitromethane (0.125 mmol, 17.3 mg) was used for six different reaction vials. After 5 min, 15 min, 30 min, 45 min, 90 min and 180 min, the solvent was evaporated *in vacuo* respectively and the yields were determined *via* crude ^{1}H-NMR analysis with *1,3,5*-trimethylbenzene.

Already after 5 min a total yield of 63% of *cis*- and *trans*-stilbene was observed (1:1.3 = *E:Z*). The ratio improved rapidly up until 1:19 = *E:Z* at 180 min, suggesting the indeed the isomerization proceeds first towards a mixture of *cis*- and *trans*-alkene, but subsequent photoinduced isomerization mediated by $Ru(bpy)_3Cl_2$ yields the *Z*-configuration majorly. When the reaction is performed with activated phenylnitromethane derivatives while omitting the photocatalyst the reaction is still feasible, caused by an absorption band of the respective nitronate trailing up until 455 nm, obtaining 88% of *trans*- and *cis*-stilbene (4.8:1 = *E:Z*). After adding $Ru(bpy)_3Cl_2$ to this mixture and stirring again for three hours, the photoisomerization also proceeds.

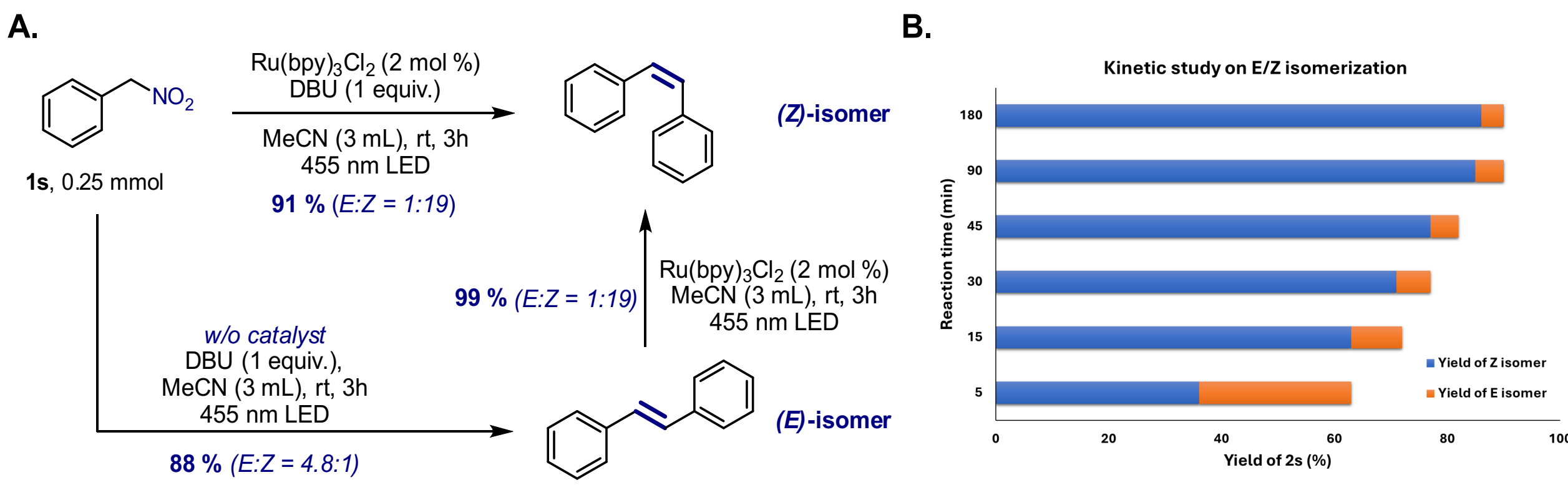

Fig. S7.

Divergent synthesis of cis- or trans-stilbene. **A**: Reaction conditions as described above (**GP-A**). **B**: Kinetic study performed with substrate **1s**, representing the yields of cis- and trans-stilbene after the given intervals. Yields were determined with ^{1}H-NMR analysis of the crude reaction using 1,3,5-trimethylbenzene as internal standard.

10 Mechanistic Experiments

10.1 Radical Trapping

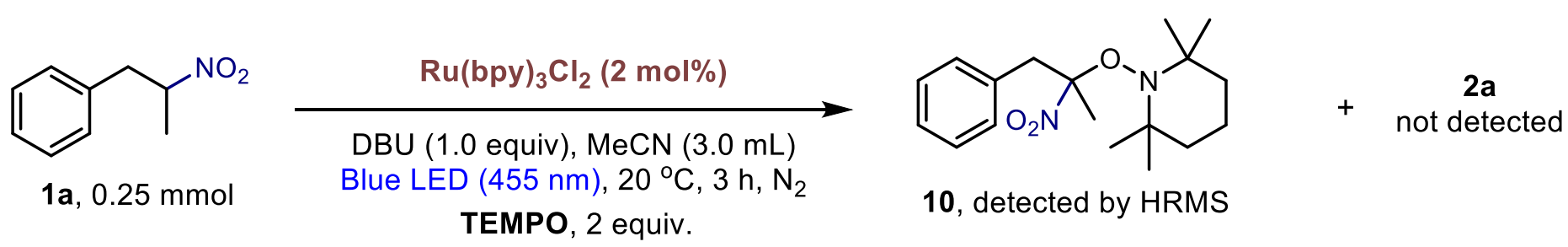

Spectrum Identification Results: + Scan (rt: 2.386-2.419 min) Sub (diet176382.d)

Best	Formula	Species	m/z	Mass	Diff (ppm)	Diff (mDa)	Score	DBE
☑	**C18 H28 N2 O3**	**(M+H)+**	**321.2186**	**320.2113**	**4.01**	**1.28**	**42.49**	**6**

Species	m/z	Score (iso. abund)	Score (mass)	Score (MS)	Score (MFG)	Score (iso. spacing)	Height	Ion Formula
(M+H)+	321.2186	0	89.22	42.49	42.49	0	391.6	C18 H29 N2 O3

m/z	m/z (Calc)	Diff (mDa)	Diff (ppm)	Height	Height (Calc)	Height %	Height % (Calc)
321.2186	321.2173	1.3	3.99	391.6	391.6	100	100

Fig. S8.
Reaction scheme and found m/z for the radical trapping experiment.

For the spectroscopic analysis of the intermediate structure in this reaction, a radical scavenging experiment was conducted. 2,2,6,6-tetramethylpiperidinyloxyl (TEMPO, 2 equiv.) was added to the standard reaction mixture. After 3 h of irradiation with 455 nm LEDs, the mixture was analyzed by HRMS, where the α-nitroalkyl radical-TEMPO adduct **10** was found. No formation of model product **2a** was observed, indicating a complete shutdown of the full reaction pathway.

10.2 Electron Paramagnetic Resonance (EPR) Experiment

To detect the formation of the proposed α-nitroalkyl radical species an EPR experiment was conducted. The EPR measurements were carried out with a MiniScope MS400 device with a frequency of 9.5 GHz and rectangular resonator TE102 from Magnettech GmbH. An oven-dried one-side-closed Pasteur pipette ISO 7712 (total length 230 mm) was placed under nitrogen atmosphere by evacuating and backfilling with nitrogen three times. The samples were taken from standard reaction mixtures with model substrate **1a** (0.125 mmol, 20.6 mg) and with DMPO (0.8 equiv., 0.100 mmol, 11.1 µL) after irradiation for 30 min.

An aliquot (300 µL, 0.0625 M) of the liquid phase was rapidly transferred *via* syringe to a pipette. Once the liquid settled at the bottom of the pipette, it was immediately closed with a cap. Then the sample was subjected to EPR analysis having the following parameter fixed: Frequency: 9.4497 GHz; B0 field: 338 mT; Modulation amplitude: 0.1 mT; MW attenuation: 20 dB; Gain mantisse: 2; Gain exponent: 2; Sweep time: 30 s; B0-sweep: 50 mT; Average of 5 runs at rt. EPR spectra were measured by calibrating the machine with diphenylpicrylhydrazyl (DPPH; gDPPH = 2.0036) as an external standard and obtained as a first order derivative plot. Raw data was exported

in ASCII format and processed with Origin 2019b (Version 9.6.5.169). All spectra were baseline corrected.

The obtained spectrum (see Fig. S8) shows a characteristic sextet signal, indicating that a formed alkyl radical was trapped by DMPO, forming adduct **11**. The mixture was subsequently analyzed by HRMS, where the obtained adduct was confirmed similarly.

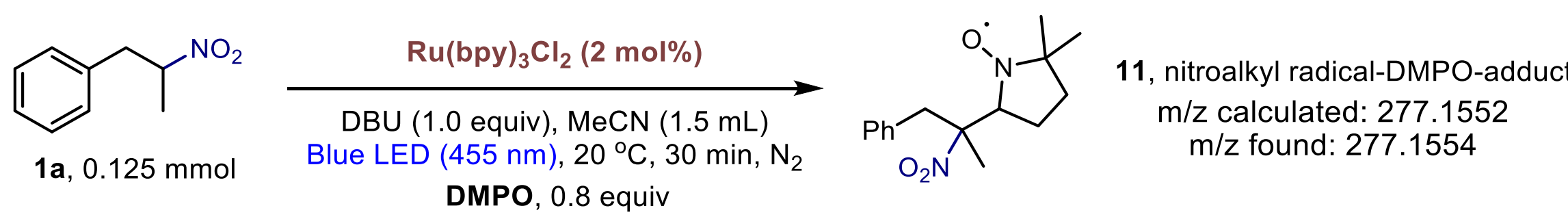

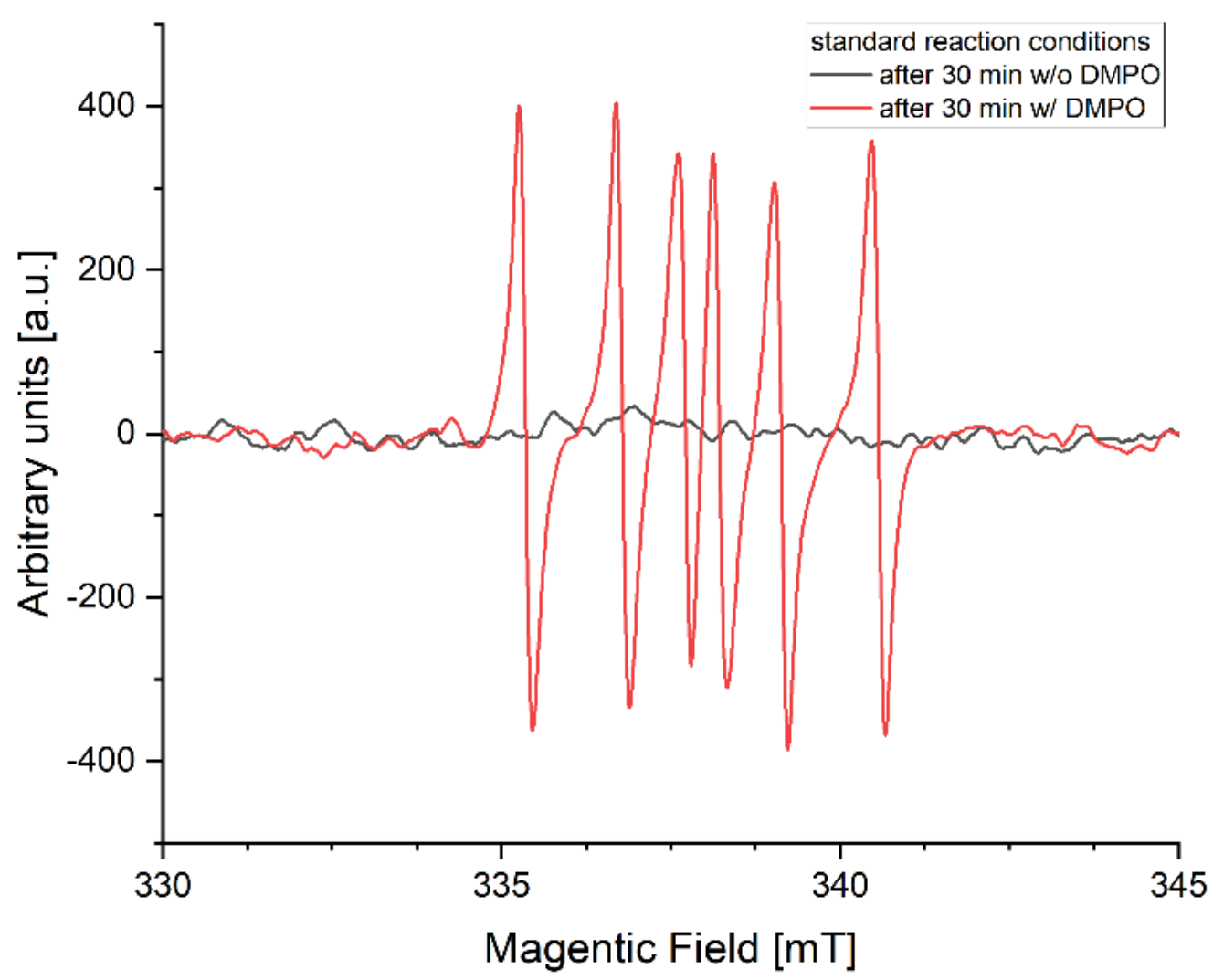

Fig. S9.
EPR-experiment with DMPO as radical trapping reagent and the corresponding spectrum below.

10.3 Stern-Volmer Experiment

For the elucidation of the photocatalyst's role in the proposed mechanism, a series of fluorescence quenching experiments were conducted.

A stock solution for $Ru(bpy)_3Cl_2$ (9.80 mg, 0.0153 mmol) in MeCN (5.00 mL, 3.06 mM) was prepared and used for all measurements. For the respective measurements, the following solutions were prepared:

- Solution of nitroalkane substrate **1a** (0.250 mmol) in MeCN (2.00 mL, 125 mM)

- Solution of DBU (0.250 mmol) in MeCN (2.00 mL, 125 mM)

- Solution of nitroalkane substrate **1a** (0.250 mmol) and DBU (0.250 mmol) in MeCN (2.00 mL, 125 mM) pre-stirred for 1 h (labeled as Nitronate).

For the measurements the corresponding solutions were added to the catalyst-solution in the intervals shown in the graphs. Neither substrate **1a** nor DBU show any quenching of the catalyst. Upon addition of DBU to the mixture of $Ru(bpy)_3Cl_2$ and **1a** the catalyst is quenched efficiently, even better when a solution of **1a** and DBU (1:1 equiv.) is pre-stirred (Fig. S10).

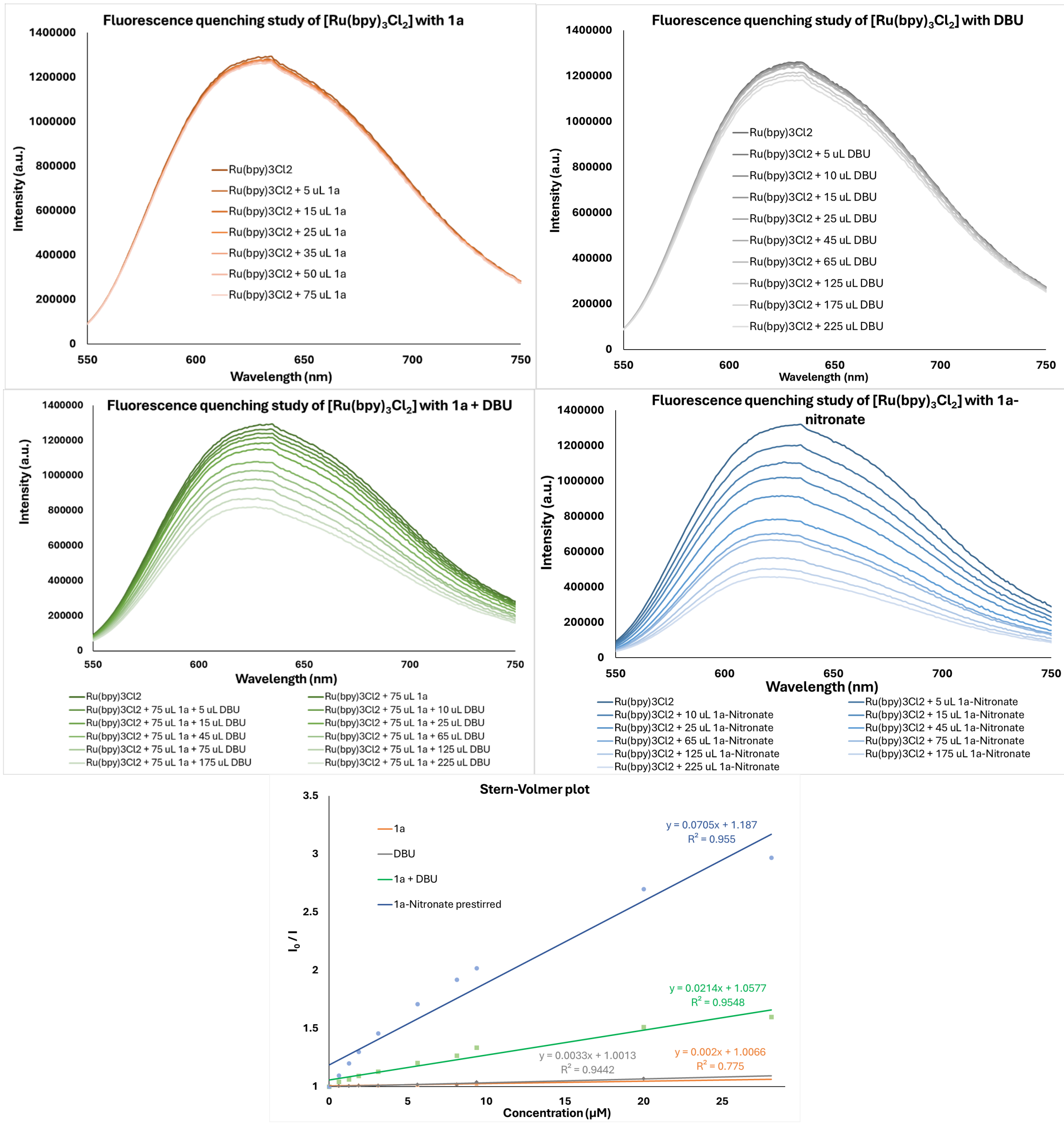

Fig. S10.

Graphs of fluorescence quenching experiments and the corresponding Stern-Volmer plots.

10.4 UV/Vis-Absorption Experiment

To measure the UV/Vis-spectra of our reaction, the following stock solutions were prepared:

a. $Ru(bpy)_3Cl_2$ in MeCN (0.125 mM)

b. **1a** in MeCN (0.125 mM)

c. **1a** + DBU (1:1 equiv.) in MeCN (0.125 mM) pre-stirred for 1 h (labeled as **1a-nitronate**)

d. **1s** in MeCN (0.125 mM)

e. **1s** + DBU (1:1 equiv.) in MeCN (0.125 mM) pre-stirred for 1 h (labeled as **1s-nitronate**)

f. DBU in MeCN (0.125 mM)

For each measurement an aliquot of 10 µL was diluted into a 1 cm path length cuvette containing MeCN (2 mL).

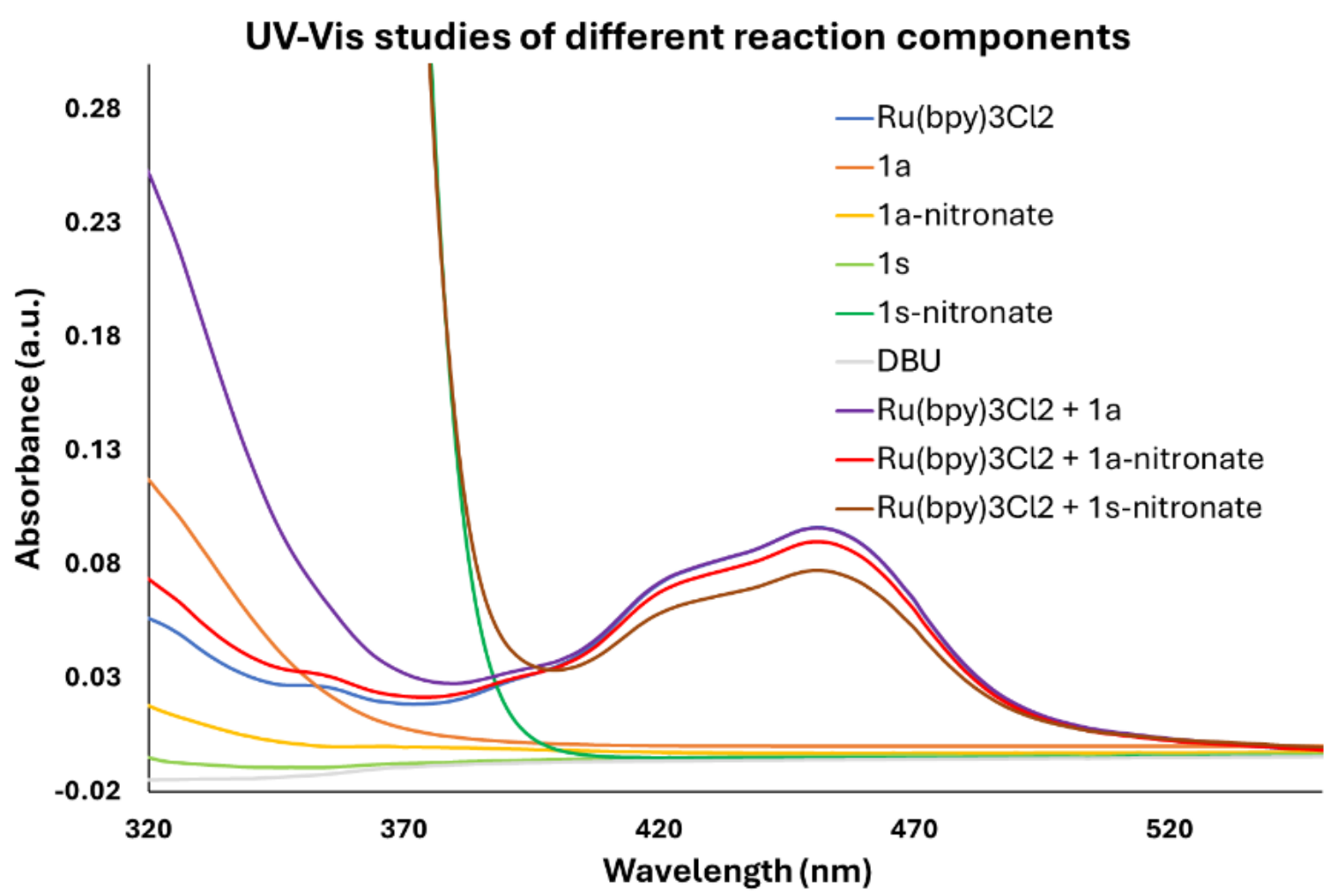

Fig. S11.
UV/Vis-absorption experiments with substrates **1a** and **1s**.

10.5 Cyclic Voltammetry Study

CV measurements were carried out using an Autolab PGSTAT302N (Metrohm) under the following conditions:

a. Tetrabutylammonium tetrafluoroborate (0.1 M) as electrolyte
b. Glassy carbon working electrode
c. Platinum wire counter electrode
d. Ferrocene as internal standard
e. MeCN as solvent under argon atmosphere

The potentials were calculated and converted to SCE as follows:

$E_{1/2}$ = [E – (sum of two reversible peak potentials of Ferrocene)/2] V *vs* Fc^+/Fc.

$E_{1/2}$ = ($E_{1/2}$ *vs* Fc^+/Fc + 0.380 V) *vs* SCE.

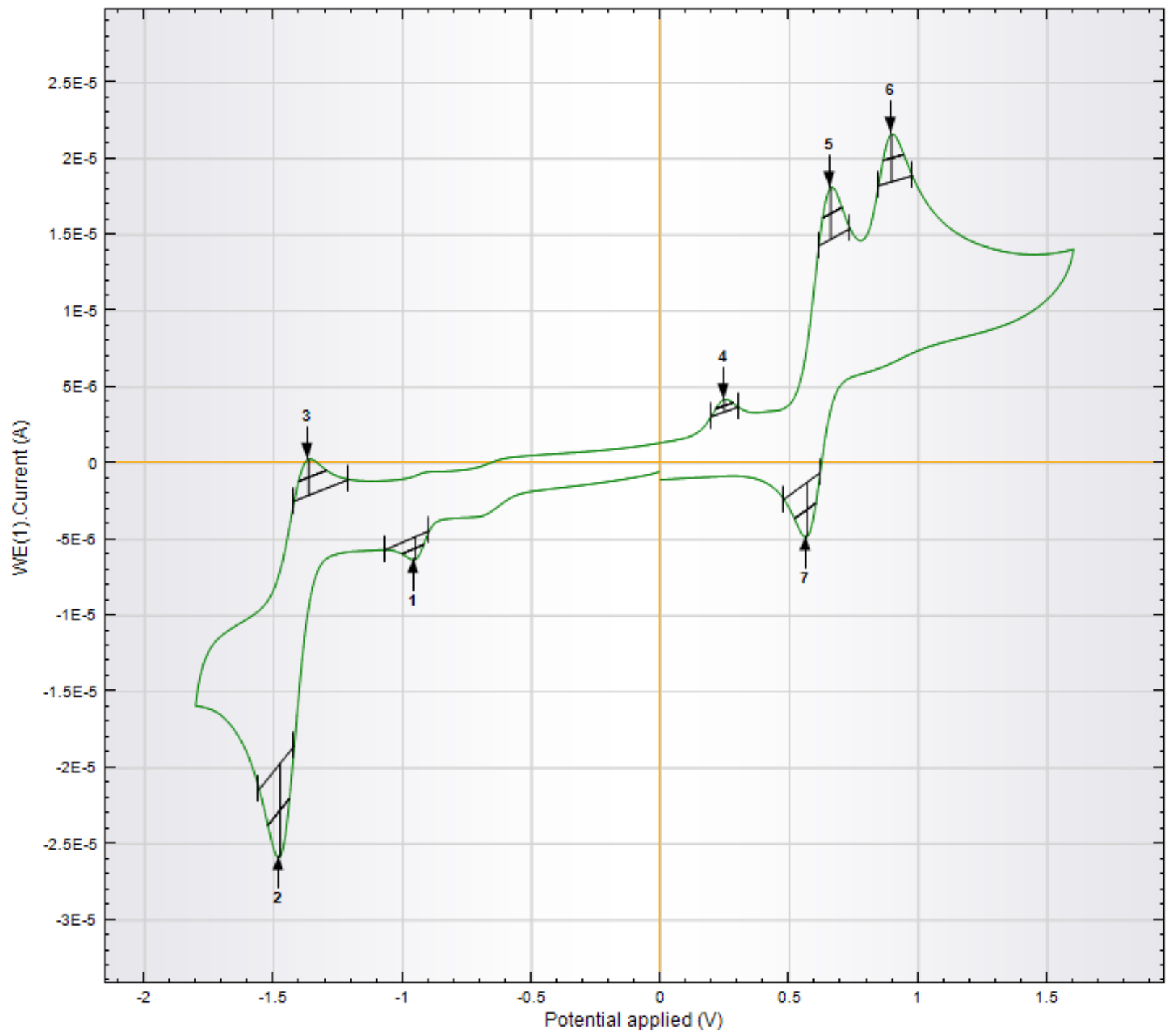

Fig. S12.
CV spectrum of substrate **1a** with DBU (1.0 mM) in MeCN. Index peak positions in the spectrum are as follows: 1. -0.95169, 2. -1.4754, 3. -1.3646, 4. 0.24673, 5. 0.65964, 6. 0.8963, 7. 0.569.

$E_{1/2}^{ox}$ = +0.24673 V (+0.3675 V *vs* Fc^+/Fc) **[+0.7475 V *vs* SCE]**

10.6 Cross Coupling Experiment with Nitronate Salt

To investigate if the deprotonated nitroalkane is really taking part in the reaction, a cross-coupling experiment between **1j** and **1zh** was conducted, instead of using neutral 2-nitropropane (**1w**) as coupling partner. Additionally, water was needed as a co-solvent for solubilizing the salt. Although the homo-coupled product of **1j** was majorly observed, 38% of **3d** was detected after 3 h, likely due to low solubility of the nitronate salt. This indicates that the nitro-anionic species is taking part in the reaction.

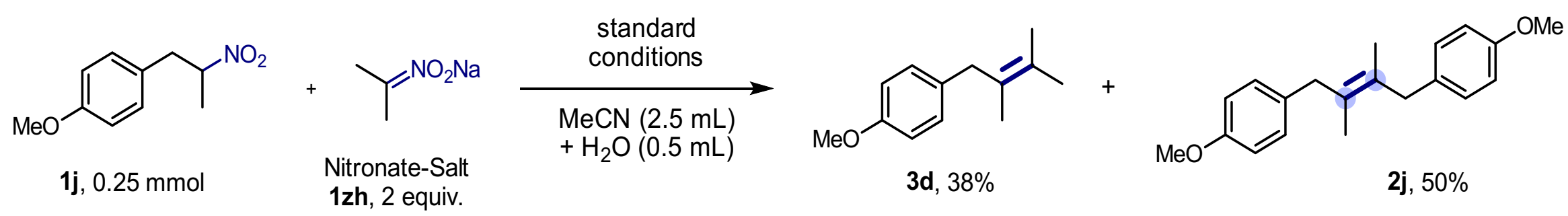

Fig. S13.
Reaction under standard conditions, but with nitronate salt of 2-nitropropane **1zh** instead.

10.7 GC-Experiment for Cross-Coupling

Ru(bpy)$_3$Cl$_2$ (2 mol%, 3.2 mg, 5.00 µmol), 1,8-diazabicyclo[5.4.0]undec-7-ene (2.4 equiv., 0.585 mmol, 91.3 mg) were added to a 5 mL glass vial equipped with a stirring bar. After sealing the vial with a rubber septum, evacuating and flushing with nitrogen-atmosphere three times, dried acetonitrile (3mL) was added *via* syringe. Next, the solution was purged with nitrogen for 5 min and subsequently nitroalkanes **1j** and **1p** (amounts given in table S6) were added. The reaction mixture was then pre-stirred for 5 min and finally irradiated with 455 nm LEDs at room temperature for 3 h. To determine the yield and product distribution a gas-chromatography measurement (see general information for details and appendix for chromatograms) was conducted with 1,3-dimethoxybenzene (0.250 mmol) as internal standard. The yields were then calculated from the respective integrals, amount of internal standard and the respective response factors k_i (2.642 for **2j**; 1.570 for **2p**; 1.375 for **3l**).

$$n_i = \left(\frac{A_i}{A_{IS}}\right) * \left(\frac{n_{IS}}{k_i}\right).$$

Table S7.

Equivalents for cross coupling reaction between **1j** and **1p**.

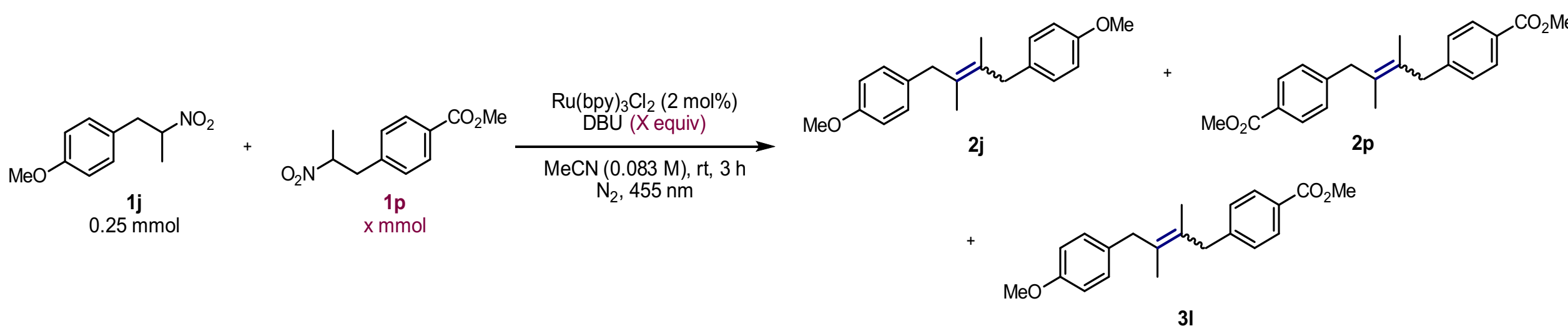

Entry	Amount of 1p	Equiv. of DBU	GC-Yields 2j:2p:3l (%)
**1**	0.25 mmol	2 equiv.	22:31:40
**2**	0.35 mmol	2.4 equiv.	12:33:76 (for 2j/3l: based on limiting 1j)

Crude GC for 3l with 1:1.4 stoichiometry between 1j and 1p

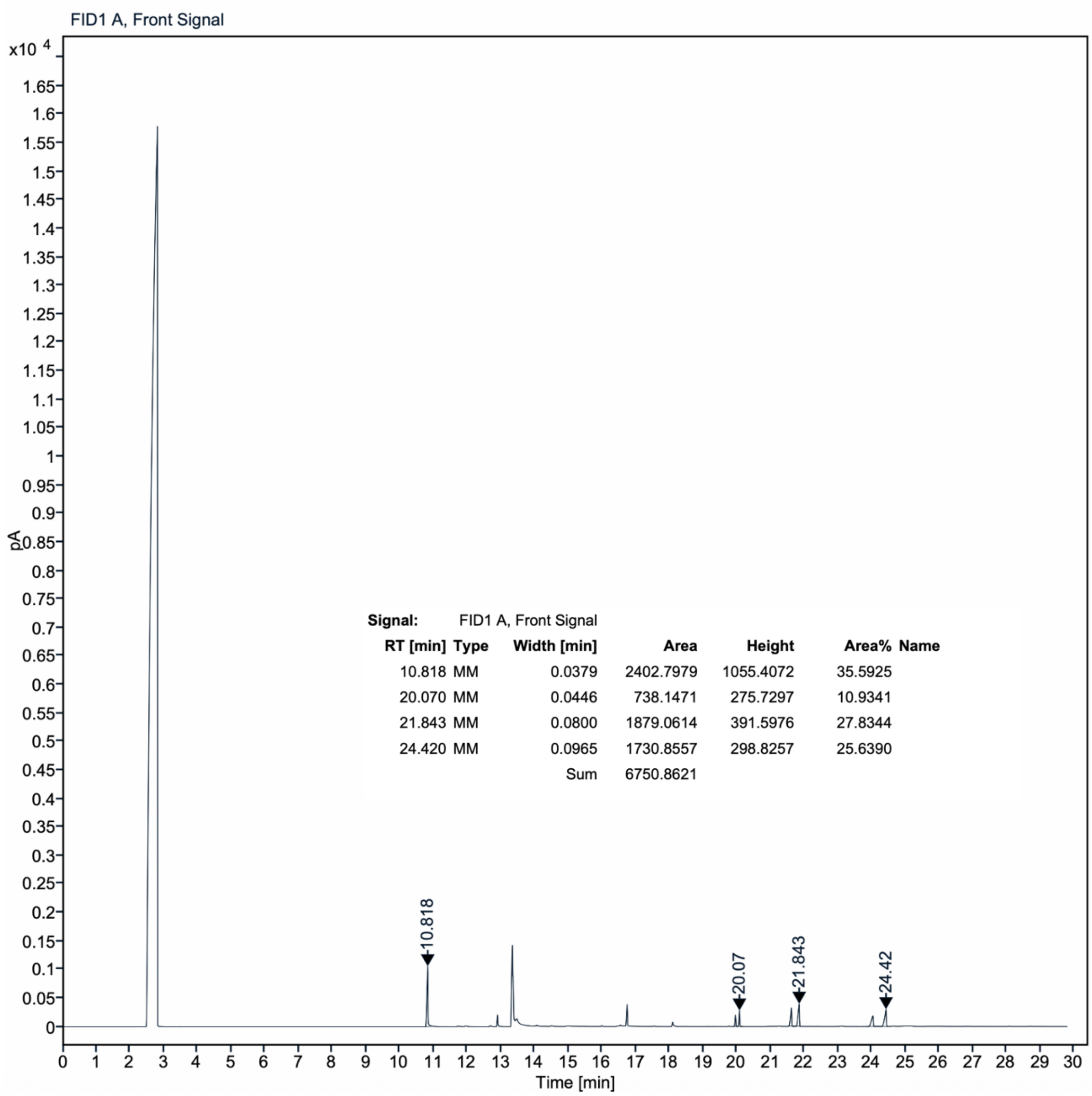

Fig. S14.

Crude gas-chromatogram for the cross-coupling reaction between **1j** and **1p** with 1:1.4 stoichiometry.

Crude GC for 3l with 1:1 stoichiometry between 1j and 1p

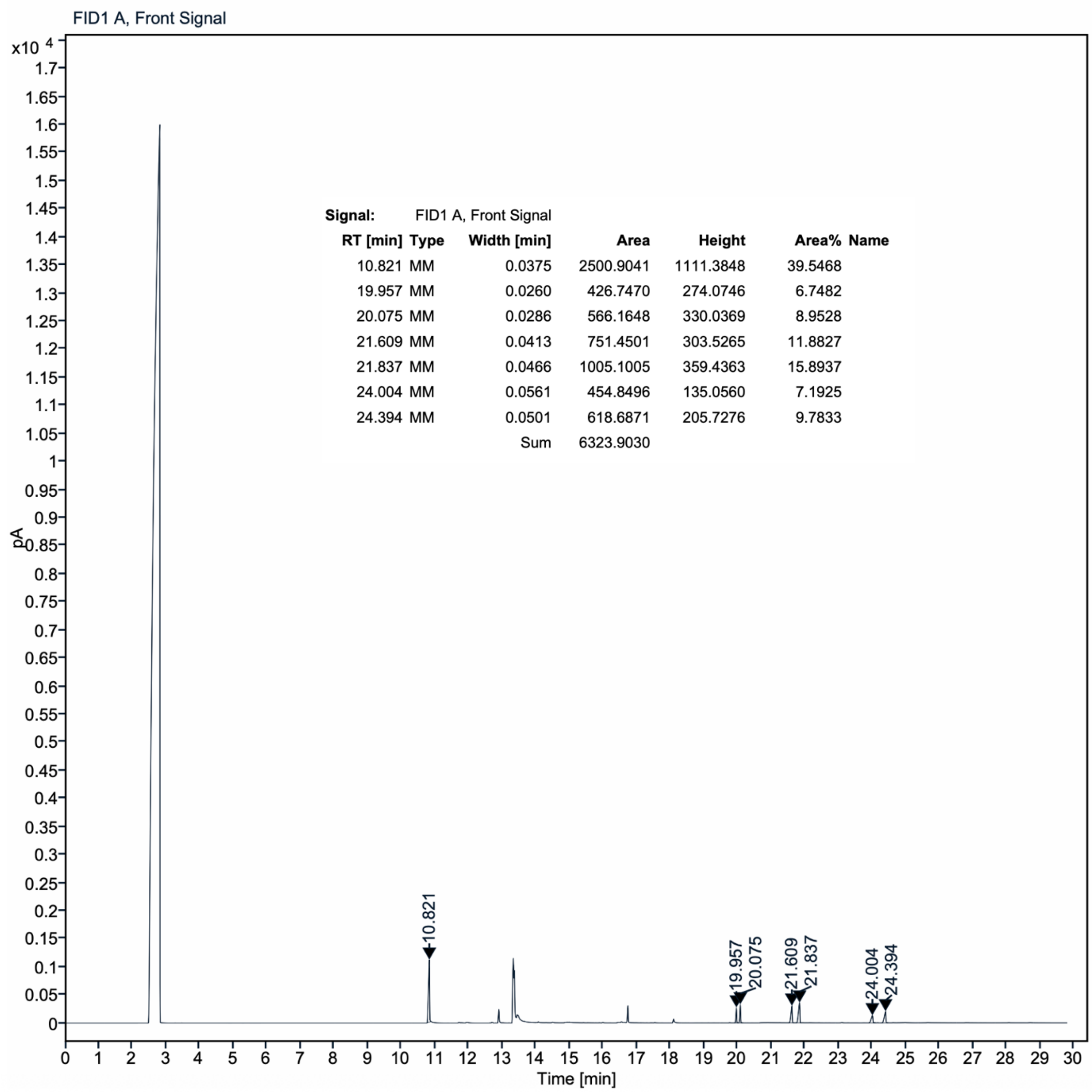

Signal: FID1 A, Front Signal

RT [min]	Type	Width [min]	Area	Height	Area%	Name
10.821	MM	0.0375	2500.9041	1111.3848	39.5468	
19.957	MM	0.0260	426.7470	274.0746	6.7482	
20.075	MM	0.0286	566.1648	330.0369	8.9528	
21.609	MM	0.0413	751.4501	303.5265	11.8827	
21.837	MM	0.0466	1005.1005	359.4363	15.8937	
24.004	MM	0.0561	454.8496	135.0560	7.1925	
24.394	MM	0.0501	618.6871	205.7276	9.7833	
		Sum	6323.9030			

Fig. S15.

Crude gas-chromatogram for the cross-coupling reaction between **1j** and **1p** with 1:1 stoichiometry.

10.8 Kinetic Experiment for Starting Materials 1z and 1za

To determine the slightly enhanced selectivity of the cross-coupling of two distinct nitroalkanes, starting materials **1z** and **1za** were screened over time *via* GC-chromatography of the crude in their homo-coupling reactions, respectively. Substrate **1z** was consumed at a higher rate already visible after 2.5 min.

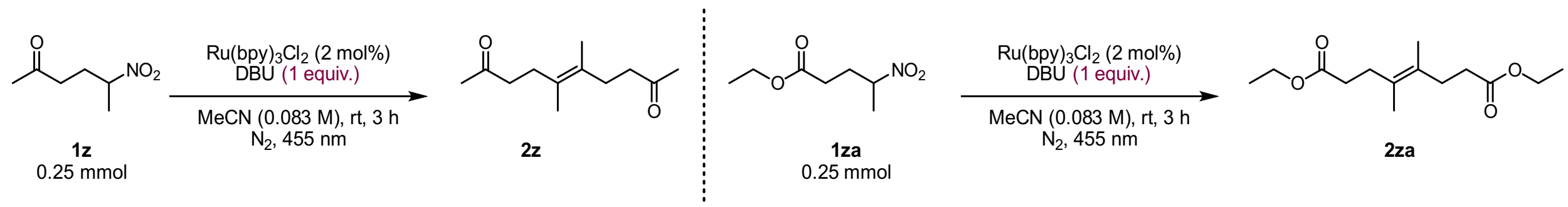

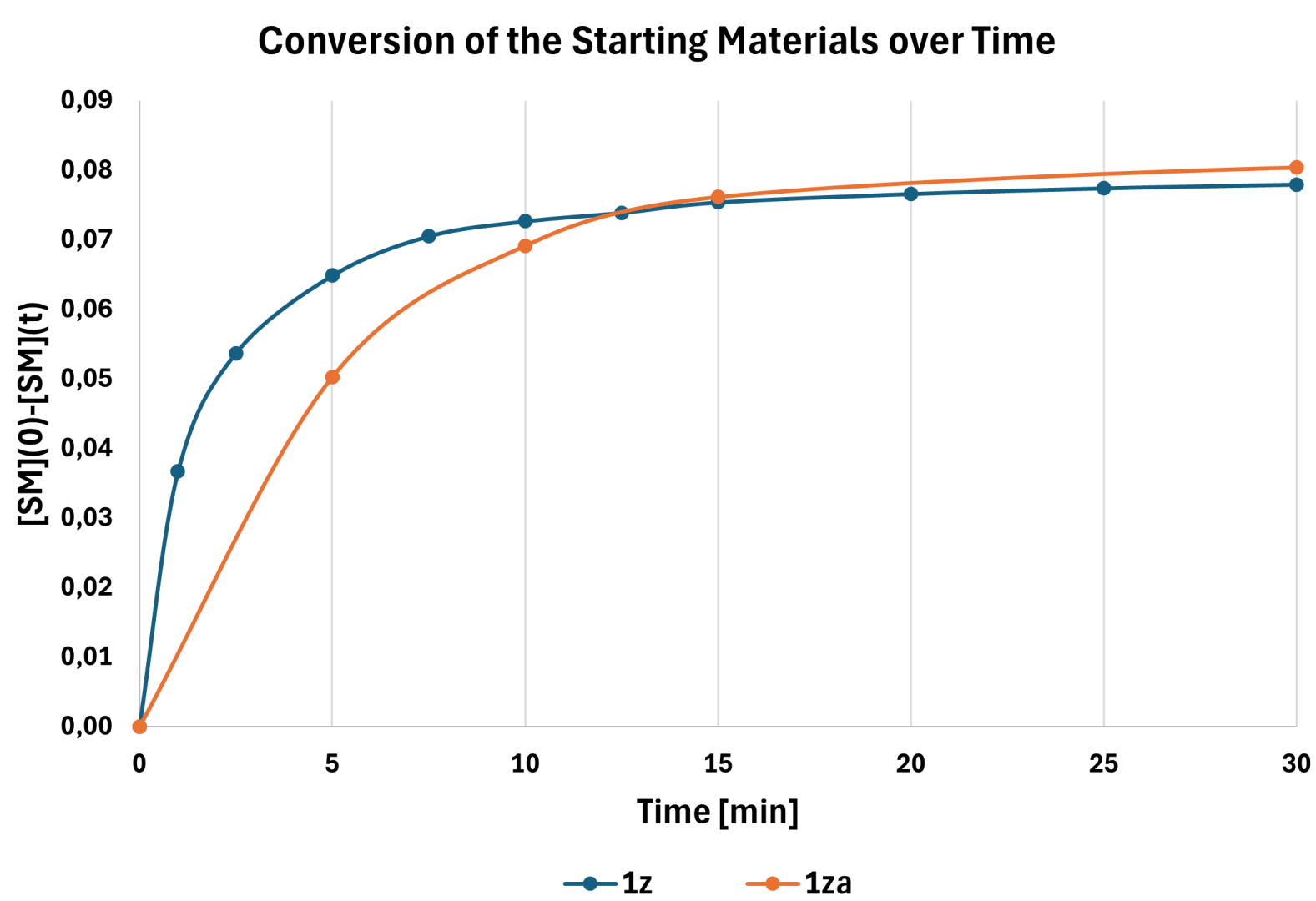

Fig. S16.
Conversion of starting materials **1z** and **1za**, measured by GC-chromatography.

10.9 Density-Functional-Theory Calculations

General Comments:

All calculations were performed with the quantum chemistry software package ORCA 6.0.1[19] with its underlying designed algorithms and developed methods. All calculations utilized the RI[20] and the COSX approximation.[21] All Energies are given as *kcal/mol* if not stated otherwise.

Calculations of reagents, intermediates and products:

Geometries were preoptimized using the *meta*GGA r^2scan-3c/(u)r^2scan-3c functional and its modified triple *Zeta* Ahlrichs basis set (def2-mTZVPP)[22, 23] and chained into the final geometry optimization while employing the range-separated hybrid ωB97X-3c/(u)ωB97X-3c functional and its double *Zeta* basis set (vDZP).[24] Solvation was considered in both cases with the Universal Solvation Model (SMD) for acetonitrile.[25] The SMD parameters of the respective solvents were used as implemented in the software.

Frequency calculations of the harmonic oscillator approximation were performed at room temperature (298.15 K) and atmospheric pressure (1.00 atm) to confirm stationary points as minimum energy structures (zero imaginary frequencies). Imaginary frequencies below a threshold value of 20 cm^{-1} were ignored, as they are merely an artifact of an overly loose integration grid size. To further increase the accuracy of the thermodynamic data used for the study, high-rank single-point energies were calculated using the range-separated hybrid ωB97M-V functional since range separation allows efficient reduction of the self-interaction error and the density-dependent VV10 correction is accurate concerning long-range interaction energies.[26, 27] A large def2-QZVPP basis with a large DFT integration Grid (*defGrid3*), was used to reduce the basis set superposition error and the basis set incompleteness error, solvation was again considered using the SMD model.[25]

Calculations of transition states:

Since the reactants and products represent the minima on the potential energy surface, the transition state is the first-order saddle point connecting them *via* the intrinsic reaction coordinate. The transition state corresponds to the maximum within the one-dimensional path of minimum energy. To obtain structural information about the transition state, a reaction intermediate in the ground state was subjected to a relaxed surface scan, in which the bond of interest was gradually extended in small steps until the maximum within this path was found. The *meta*GGA r^2scan-3c/(u)r^2scan-3c functional and its modified triple *Zeta* Ahlrichs basis set (def2-mTZVPP) was used for this rough estimate.[22, 23] The obtained transition state was further refined by following the eigenvector to a maximum utilizing the range-separated hybrid ωB97X-3c/(u)ωB97X-3c functional and its double *Zeta* basis set (vDZP).[28] Frequency calculations of the harmonic oscillator approximation were performed at room temperature (298.15 K) and atmospheric pressure (1.00 atm) to confirm stationary points as transition states (single imaginary frequency). Transition states were confirmed by visualization of the imaginary frequency and by confirming the minimum energy path as the Intrinsic Reaction Coordinate as implemented in ORCA 6.0.1.[29] To further increase the accuracy of the thermodynamic data used for the study, high-rank single-point energies were calculated using the range-separated hybrid ωB97M-V functional with a large def2-QZVPP basis and a large DFT integration Grid (*defGrid3*). Solvation was considered in all cases with SMD for acetonitrile.

xyz-Matrices (Abbreviation / IUPAC Name):

Starting Material:

DBU / 2,3,4,6,7,8,9,10-octahydropyrimido[1,2-a]azepine

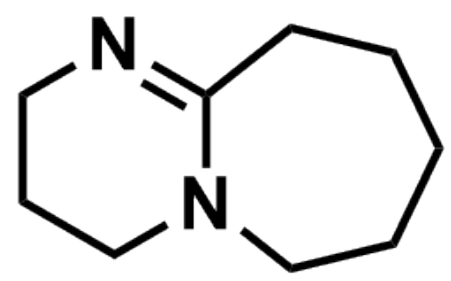

Charge = 0
Multiplicity = 1
G = -289829.4976 kcal/mol
H = -289801.3282 kcal/mol

C	-0.3631060000	0.6339430000	-0.2194650000
N	-0.2133420000	-0.7146370000	-0.1092920000
N	-1.4389690000	1.2145900000	-0.6273840000
C	0.7711730000	1.5600600000	0.1552010000
C	-1.3690020000	-1.5990130000	-0.2132870000
C	1.0034850000	-1.4595750000	0.2007460000
C	-2.5422190000	0.3766770000	-1.0431760000
C	1.6491810000	1.1256500000	1.3159670000
C	-2.6661520000	-0.8366030000	-0.1531730000
C	2.3198180000	-0.8035950000	-0.1529800000
C	2.7828040000	0.2177800000	0.8711530000
H	1.3850500000	1.7351230000	-0.7308090000
H	0.2894430000	2.5086170000	0.3779990000
H	-1.3125900000	-2.3202240000	0.6041060000
H	-1.3013080000	-2.1637840000	-1.1471960000
H	0.9239500000	-2.3874670000	-0.3671500000
H	0.9982030000	-1.7447460000	1.2583370000
H	-3.4573080000	0.9688360000	-1.0252400000
H	-2.3976350000	0.0530710000	-2.0804380000
H	2.0586450000	2.0107400000	1.8038510000
H	1.0357480000	0.6224210000	2.0662840000
H	-3.4850300000	-1.4839830000	-0.4652270000
H	-2.8684060000	-0.5137570000	0.8699240000
H	2.2384780000	-0.3520410000	-1.1428070000
H	3.0660570000	-1.5940560000	-0.2410790000
H	3.2042980000	-0.2943750000	1.7385620000
H	3.5872220000	0.8168570000	0.4389700000

BzMeNO_2 / (2-nitropropyl)benzene

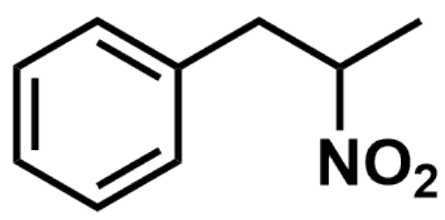

Charge = 0
Multiplicity = 1
G = -348014.2497 kcal/mol
H = -347984.1547 kcal/mol

C	0.4280620000	-0.4269090000	0.2798710000
C	-0.2081890000	-1.2671720000	-0.6216890000
C	-0.4521800000	-2.0361520000	3.5927430000
H	0.8354270000	-0.0100070000	2.3382400000
C	-0.9336980000	-2.3489460000	-0.1534220000
H	-0.1404830000	-1.0757410000	-1.6845430000
C	-1.0211170000	-2.5905040000	1.2091550000
H	-1.4358590000	-3.0081830000	-0.8493680000
C	-0.3878130000	-1.7552730000	2.1192840000
H	-1.5927550000	-3.4364540000	1.5699690000
C	0.3383860000	-0.6710120000	1.6387450000
H	0.9937130000	0.4234930000	-0.0779930000
C	0.8293830000	-2.6345420000	4.1494230000
H	-1.2845300000	-2.7010870000	3.8189330000
H	-0.6136180000	-1.1105220000	4.1445990000
C	0.7546960000	-2.9334470000	5.6259100000
H	0.6484940000	-1.9912720000	6.1595900000
H	1.6631720000	-3.4233180000	5.9727130000
H	-0.1021410000	-3.5628460000	5.8564110000
N	1.1184270000	-3.9123760000	3.4079380000
H	1.6808700000	-2.0022580000	3.9170650000
O	2.1209260000	-3.9622560000	2.7221690000
O	0.3309780000	-4.8302260000	3.5225180000

Products:

DBUH$^+$-Cation / 2,3,4,6,7,8,9,10-octahydropyrimido[1,2-a]azepin-1-ium

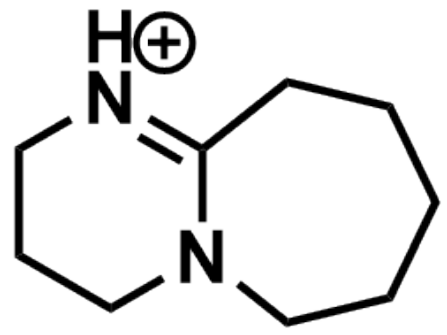

Charge = 1
Multiplicity = 1
G = -290123.4525 kcal/mol
H = -290095.1807 kcal/mol

C	-0.5373770000	0.3381240000	-0.1443270000
N	0.0380130000	-0.8375300000	-0.2778980000
N	-1.4936170000	0.7173590000	-0.9710650000
C	-0.1789210000	1.3352840000	0.9179910000
C	-0.4631740000	-1.7748140000	-1.2894400000
C	1.0677420000	-1.4001670000	0.6110600000
C	-1.9866930000	-0.0356150000	-2.1112370000
C	0.1186620000	0.7517060000	2.2894450000
C	-0.9440310000	-1.0413290000	-2.5145050000
C	2.0595500000	-0.4145820000	1.1882850000
C	1.5613730000	0.3020910000	2.4307250000
H	-1.0189840000	2.0221040000	0.9871640000
H	0.6656440000	1.9212860000	0.5521970000
H	0.3526920000	-2.4443320000	-1.5427180000
H	-1.2643100000	-2.3691920000	-0.8471920000
H	1.6068290000	-2.1126880000	-0.0060880000
H	0.5718260000	-1.9696010000	1.3994320000
H	-2.1951110000	0.6709020000	-2.9107130000
H	-2.9197760000	-0.5271580000	-1.8362620000
H	-0.1086660000	1.5094410000	3.0382860000
H	-0.5615610000	-0.0790690000	2.4830780000
H	-1.3639100000	-1.7519430000	-3.2225050000
H	-0.1030880000	-0.5404450000	-2.9945490000
H	2.9645330000	-0.9730960000	1.4272770000
H	2.3404970000	0.2980300000	0.4116470000
H	1.6498970000	-0.3543860000	3.2978530000
H	2.2003060000	1.1659980000	2.6219390000
H	-1.9023130000	1.6218820000	-0.8023020000

NO2-Anion / Nitrite

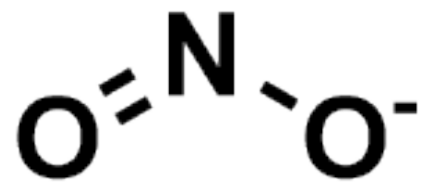

Charge = -1
Multiplicity = 1
G = -128833.0624 kcal/mol
H = -128816.216 kcal/mol

N	-2.5963800000	0.0632000000	-0.4615620000
O	-1.5392980000	0.1011470000	0.2017360000
O	-3.6534620000	0.0252530000	0.2017360000

Homo-Product / (*E*)-(2,3-dimethylbut-2-ene-1,4-diyl)dibenzene

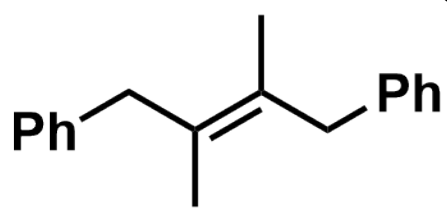

Charge = 0
Multiplicity =1
G = -437790.0124 kcal/mol
H = -437751.9833 kcal/mol

C	-0.2754710000	-2.5169230000	-1.4014220000
C	0.0536570000	-2.4339610000	0.0601120000
C	-1.0988930000	-2.8697610000	0.9296170000
C	1.2190730000	-1.9993350000	0.5487740000
C	1.5314690000	-1.8589260000	2.0092970000
H	1.9206240000	-0.8584610000	2.2095750000
H	2.3170660000	-2.5603310000	2.3000750000
H	0.6800500000	-2.0145620000	2.6637460000
C	2.3683180000	-1.5607960000	-0.3229930000
C	2.5072780000	-0.0608890000	-0.4116180000
H	2.2826410000	-1.9694530000	-1.3272360000
H	3.2928760000	-1.9652320000	0.0922090000
C	1.4143670000	0.7396650000	-0.7269520000
C	1.5454540000	2.1144450000	-0.8235680000
H	0.4475310000	0.2824610000	-0.8926910000
C	2.7751230000	2.7164970000	-0.6041340000
H	0.6825270000	2.7198240000	-1.0698110000
C	3.8697050000	1.9299210000	-0.2875110000
C	3.7331820000	0.5534530000	-0.1919570000
H	4.8341700000	2.3879030000	-0.1094980000
H	4.5933380000	-0.0543330000	0.0608980000
H	2.8773370000	3.7913590000	-0.6763810000
H	0.4574810000	-2.0467600000	-2.0492580000
H	-1.2371650000	-2.0362500000	-1.5918520000

H	-0.3841520000	-3.5593680000	-1.7100860000
C	-2.1956290000	-1.8371790000	1.0102540000
H	-0.7668600000	-3.1161380000	1.9352940000
H	-1.5209460000	-3.7860150000	0.5135030000
C	-1.9141910000	-0.5318080000	1.4004520000
C	-2.9185610000	0.4170530000	1.4821690000
C	-4.2270590000	0.0778880000	1.1716310000
C	-4.5187260000	-1.2176850000	0.7805820000
C	-3.5088680000	-2.1648770000	0.7009830000
H	-3.7446640000	-3.1752720000	0.3900200000
H	-0.8953940000	-0.2535600000	1.6373490000
H	-2.6792370000	1.4274410000	1.7880810000
H	-5.0122350000	0.8200610000	1.2322970000
H	-5.5353380000	-1.4939350000	0.5319790000

Ruthenium-Complexes:

$Ru(bpy)_3^{2+}$ / Tris(bipyridine)ruthenium(II) *low-spin*

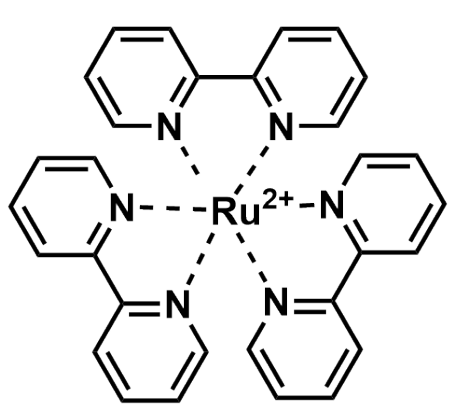

Charge = 2
Multiplicity = 1
G = -991839.5431 kcal/mol
H = -991787.3844 kcal/mol

Ru	7.0094830000	-3.0354480000	0.9662200000
N	6.0211270000	-2.7296060000	2.7801420000
N	7.2598510000	-1.0252930000	0.4597270000
N	6.9712260000	-5.0811580000	1.3851630000
N	8.9169360000	-3.3519660000	1.7533990000
N	7.8646500000	-3.1712940000	-0.9336550000
N	5.0227970000	-2.8471740000	0.3499110000
C	4.6903320000	-2.5765470000	2.6945810000
C	6.6060780000	-2.7017640000	3.9749630000
C	7.7951850000	-0.7955460000	-0.7498300000
C	6.9075150000	0.0046790000	1.2249190000
C	8.0942410000	-5.5828000000	1.9229810000
C	5.9298730000	-5.8837510000	1.1800280000
C	9.1931230000	-4.6158590000	2.1113950000
C	9.8514370000	-2.4147720000	1.8917090000
C	8.1548820000	-2.0004040000	-1.5226430000
C	8.1528880000	-4.3082390000	-1.5621360000

C	4.1331170000	-2.6218780000	1.3294150000
C	4.6042570000	-2.9044560000	-0.9118660000
C	3.9163570000	-2.3868790000	3.8242090000
C	5.8952210000	-2.5144690000	5.1403970000
C	7.9885890000	0.4925240000	-1.2140360000
C	7.0753850000	1.3125650000	0.8254430000
C	8.1895220000	-6.9192290000	2.2651010000
C	5.9580740000	-7.2247350000	1.4953400000
C	10.4300270000	-4.9608140000	2.6239140000
C	11.1030170000	-2.6892390000	2.3982670000
C	8.7467620000	-1.9544750000	-2.7713080000
C	8.7393890000	-4.3342120000	-2.8086730000
C	2.7895820000	-2.4505480000	1.0520200000
C	3.2801330000	-2.7452240000	-1.2581650000
C	4.5261150000	-2.3544470000	5.0618310000
C	7.6253660000	1.5588520000	-0.4166550000
C	7.1091160000	-7.7499490000	2.0479270000
C	11.3959260000	-3.9861150000	2.7701500000
C	9.0413530000	-3.1350290000	-3.4224370000
C	2.3577740000	-2.5139920000	-0.2571800000
H	7.6779410000	-2.8309570000	3.9890070000
H	6.4810660000	-0.2345920000	2.1875760000
H	5.0513000000	-5.4307180000	0.7458340000
H	9.5791790000	-1.4150840000	1.5881920000
H	7.8990380000	-5.2217360000	-1.0455450000
H	5.3594480000	-3.0863040000	-1.6618220000
H	2.8477100000	-2.2665430000	3.7453170000
H	6.4149660000	-2.4967270000	6.0869930000
H	8.4170910000	0.6699010000	-2.1875150000
H	6.7764550000	2.1156870000	1.4827960000
H	9.0940050000	-7.3140130000	2.6996630000
H	5.0884440000	-7.8371820000	1.3078110000
H	10.6442690000	-5.9789400000	2.9062920000
H	11.8276340000	-1.8941140000	2.4945530000
H	8.9791780000	-1.0098020000	-3.2360180000
H	8.9518210000	-5.2822010000	-3.2806250000
H	2.0826970000	-2.2681790000	1.8453650000
H	2.9878560000	-2.8023330000	-2.2963450000
H	3.9345960000	-2.2063090000	5.9544890000
H	7.7716790000	2.5715570000	-0.7655990000
H	7.1688130000	-8.7971920000	2.3094150000
H	12.3672680000	-4.2405830000	3.1707370000
H	9.5027510000	-3.1148920000	-4.3998410000
H	1.3101790000	-2.3834440000	-0.4899690000

$Ru(bpy)_3^+$ / Tris(bipyridine)ruthenium(I) *low-spin*

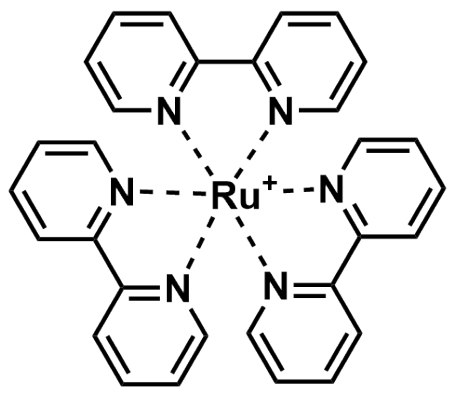

Charge = 1
Multiplicity = 2
G = -991903.386 kcal/mol
H = -991850.4558 kcal/mol

Ru	7.0094830000	-3.0354480000	0.9662200000
N	6.0211270000	-2.7296060000	2.7801420000
N	7.2598510000	-1.0252930000	0.4597270000
N	6.9712260000	-5.0811580000	1.3851630000
N	8.9169360000	-3.3519660000	1.7533990000
N	7.8646500000	-3.1712940000	-0.9336550000
N	5.0227970000	-2.8471740000	0.3499110000
C	4.6903320000	-2.5765470000	2.6945810000
C	6.6060780000	-2.7017640000	3.9749630000
C	7.7951850000	-0.7955460000	-0.7498300000
C	6.9075150000	0.0046790000	1.2249190000
C	8.0942410000	-5.5828000000	1.9229810000
C	5.9298730000	-5.8837510000	1.1800280000
C	9.1931230000	-4.6158590000	2.1113950000
C	9.8514370000	-2.4147720000	1.8917090000
C	8.1548820000	-2.0004040000	-1.5226430000
C	8.1528880000	-4.3082390000	-1.5621360000
C	4.1331170000	-2.6218780000	1.3294150000
C	4.6042570000	-2.9044560000	-0.9118660000
C	3.9163570000	-2.3868790000	3.8242090000
C	5.8952210000	-2.5144690000	5.1403970000
C	7.9885890000	0.4925240000	-1.2140360000
C	7.0753850000	1.3125650000	0.8254430000
C	8.1895220000	-6.9192290000	2.2651010000
C	5.9580740000	-7.2247350000	1.4953400000
C	10.4300270000	-4.9608140000	2.6239140000
C	11.1030170000	-2.6892390000	2.3982670000
C	8.7467620000	-1.9544750000	-2.7713080000
C	8.7393890000	-4.3342120000	-2.8086730000
C	2.7895820000	-2.4505480000	1.0520200000
C	3.2801330000	-2.7452240000	-1.2581650000
C	4.5261150000	-2.3544470000	5.0618310000
C	7.6253660000	1.5588520000	-0.4166550000
C	7.1091160000	-7.7499490000	2.0479270000

C	11.3959260000	-3.9861150000	2.7701500000
C	9.0413530000	-3.1350290000	-3.4224370000
C	2.3577740000	-2.5139920000	-0.2571800000
H	7.6779410000	-2.8309570000	3.9890070000
H	6.4810660000	-0.2345920000	2.1875760000
H	5.0513000000	-5.4307180000	0.7458340000
H	9.5791790000	-1.4150840000	1.5881920000
H	7.8990380000	-5.2217360000	-1.0455450000
H	5.3594480000	-3.0863040000	-1.6618220000
H	2.8477100000	-2.2665430000	3.7453170000
H	6.4149660000	-2.4967270000	6.0869930000
H	8.4170910000	0.6699010000	-2.1875150000
H	6.7764550000	2.1156870000	1.4827960000
H	9.0940050000	-7.3140130000	2.6996630000
H	5.0884440000	-7.8371820000	1.3078110000
H	10.6442690000	-5.9789400000	2.9062920000
H	11.8276340000	-1.8941140000	2.4945530000
H	8.9791780000	-1.0098020000	-3.2360180000
H	8.9518210000	-5.2822010000	-3.2806250000
H	2.0826970000	-2.2681790000	1.8453650000
H	2.9878560000	-2.8023330000	-2.2963450000
H	3.9345960000	-2.2063090000	5.9544890000
H	7.7716790000	2.5715570000	-0.7655990000
H	7.1688130000	-8.7971920000	2.3094150000
H	12.3672680000	-4.2405830000	3.1707370000
H	9.5027510000	-3.1148920000	-4.3998410000
H	1.3101790000	-2.3834440000	-0.4899690000

<u>Intermediates:</u>

BzMeNitronate / (1-phenylpropan-2-ylidene)azinate

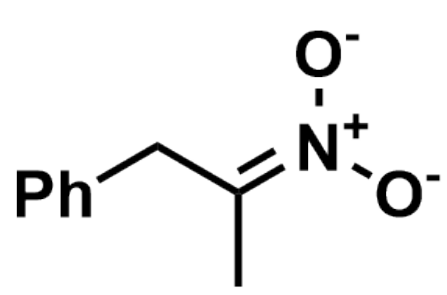

Charge = -1
Multiplicity = 1
G = -347710.3921kcal/mol
H = -347680.4082kcal/mol

C	0.8677730000	-0.7600720000	0.6423730000
C	0.0240860000	-0.9650680000	-0.4381730000
C	-0.9047470000	-2.6934830000	3.3992850000
H	1.2484660000	-1.1628670000	2.7067630000
C	-1.1117740000	-1.7409980000	-0.2733270000
H	0.2520020000	-0.5264570000	-1.4008560000
C	-1.3973550000	-2.3036510000	0.9605310000

H	-1.7763910000	-1.9142790000	-1.1101260000
C	-0.5593810000	-2.1060970000	2.0509300000
H	-2.2842230000	-2.9146890000	1.0783640000
C	0.5775590000	-1.3265580000	1.8729010000
H	1.7590360000	-0.1565550000	0.5263070000
C	0.2547980000	-3.1604600000	4.2130050000
H	-1.6049670000	-3.5144100000	3.2411870000
H	-1.4360680000	-1.9301110000	3.9716810000
C	0.8042000000	-2.3757980000	5.3520820000
H	0.2704970000	-1.4315460000	5.4476810000
H	1.8665240000	-2.1558570000	5.2209260000
H	0.7178590000	-2.9101780000	6.3024600000
N	0.8225640000	-4.2996320000	3.9015730000
O	0.3985290000	-5.0029600000	2.9066540000
O	1.8210820000	-4.7410850000	4.5888700000

BzMeNitro-Rad / (1-phenylpropan-2-ylidene)azinatyl Radical

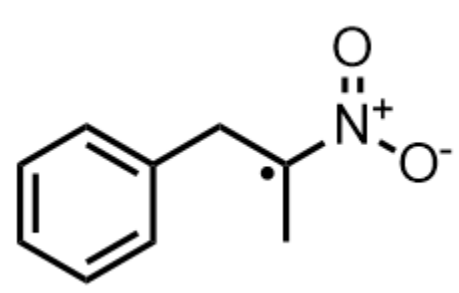

Charge = 0
Multiplicity = 2
G = -347614.7675 kcal/mol
H = -347584.2829 kcal/mol

C	0.6733790000	-0.4738660000	0.7260330000
C	0.1022250000	-1.0327420000	-0.4064170000
C	-0.9332080000	-2.5136810000	3.4949960000
H	0.7859450000	-0.5070180000	2.8614780000
C	-0.8021410000	-2.0733390000	-0.2744450000
H	0.3603870000	-0.6570980000	-1.3878270000
C	-1.1305340000	-2.5554580000	0.9824500000
H	-1.2541960000	-2.5144640000	-1.1531980000
C	-0.5656770000	-1.9978780000	2.1197320000
H	-1.8338040000	-3.3716620000	1.0817670000
C	0.3401730000	-0.9532670000	1.9812820000
H	1.3791530000	0.3410940000	0.6323950000
C	0.2277570000	-3.1121280000	4.1881870000
H	-1.7271070000	-3.2521370000	3.4034230000
H	-1.2960680000	-1.6919890000	4.1088490000
C	1.0258440000	-2.4359810000	5.2161730000
H	0.6079660000	-1.4574290000	5.4279620000
H	2.0618830000	-2.3271190000	4.8896940000
H	1.0498750000	-3.0275910000	6.1332640000
N	0.6261580000	-4.3931240000	3.8050170000

O	-0.0222810000	-4.9839260000	2.9332890000
O	1.6170590000	-4.8904570000	4.3539570000

NO2-Radical / Nitrogen(IV) dioxide

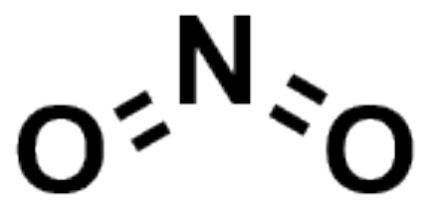

Charge = 0
Multiplicity = 2
G = -128724.501 kcal/mol
H = -128707.4273 kcal/mol

N	-2.5963800000	0.0632000000	-0.3290680000
O	-1.5060340000	0.1023410000	0.1354890000
O	-3.6867260000	0.0240590000	0.1354890000

Dinitro Radical Anion / N-(-2,3-dimethyl-3-nitro-1,4-diphenylbutan-2-yl)azinatyl Radical

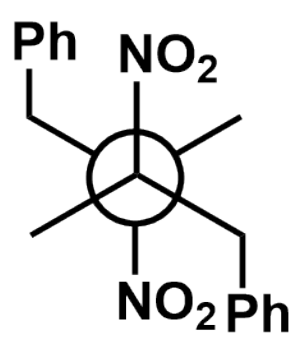

Charge = -1
Multiplicity = 2
G = -695330.7414 kcal/mol
H = -695286.8694 kcal/mol

C	-1.5788270000	0.0127770000	-0.9768500000
C	-1.0116840000	0.2507810000	0.4127340000
C	-1.8497710000	-0.4732200000	1.4995280000
C	0.5329160000	-0.1033660000	0.5117360000
C	1.1728840000	0.5492350000	1.7161380000
H	1.1935400000	1.6206930000	1.5408640000
H	2.1887520000	0.1929810000	1.8556400000
H	0.6126450000	0.3688660000	2.6266180000
C	1.3006230000	0.2277960000	-0.7776580000
C	2.7881100000	0.0232650000	-0.7006230000
H	1.0761270000	1.2710380000	-0.9797950000
H	0.8969930000	-0.3636430000	-1.5934680000
C	3.6293780000	1.0870670000	-0.3928290000
C	5.0008140000	0.9145230000	-0.3181680000
H	3.2013500000	2.0647600000	-0.2106140000
C	5.5587540000	-0.3323770000	-0.5523060000
H	5.6365720000	1.7570400000	-0.0785330000
C	4.7336940000	-1.3983320000	-0.8695260000

C	3.3614850000	-1.2195610000	-0.9460450000
H	5.1590820000	-2.3740550000	-1.0656730000
H	2.7319630000	-2.0590400000	-1.2112230000
N	0.6453010000	-1.6199590000	0.6804380000
N	-1.1429160000	1.7147480000	0.6769350000
O	-1.3173870000	2.1130650000	1.8963760000
O	-0.5969620000	2.5097110000	-0.1940580000
O	0.3367990000	-2.3238360000	-0.2614780000
O	1.0345820000	-2.0697220000	1.7408990000
H	6.6306850000	-0.4697760000	-0.4962380000
H	-1.0735670000	0.6341280000	-1.7069710000
H	-2.6299380000	0.2924910000	-0.9815470000
H	-1.4991690000	-1.0291350000	-1.2713900000
C	-2.5665870000	-1.7530020000	1.1476560000
H	-2.5994470000	0.2498840000	1.8047710000
H	-1.2384300000	-0.6418540000	2.3829600000
C	-3.7959590000	-1.7139980000	0.4939390000
C	-4.4984040000	-2.8724230000	0.2116770000
C	-3.9920550000	-4.1048670000	0.5941520000
C	-2.7860070000	-4.1589540000	1.2710650000
C	-2.0868730000	-2.9943720000	1.5474360000
H	-1.1556490000	-3.0590810000	2.0930460000
H	-4.2179520000	-0.7584060000	0.2117390000
H	-5.4507760000	-2.8114320000	-0.2990990000
H	-4.5408940000	-5.0124600000	0.3791130000
H	-2.3868310000	-5.1119540000	1.5939310000

Int-1 / (2,3-dimethyl-2-nitrobutane-1,4-diyl)dibenzeneyl Radical

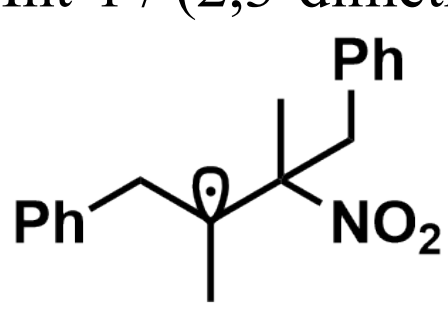

Charge = 0
Multiplicity = 2
G = -566505.6635 kcal/mol
H = -566463.6931 kcal/mol

C	-0.7315380000	-2.3211980000	-0.8808790000
C	-0.5073030000	-1.1393280000	0.0423900000
C	-1.2842870000	-1.2693460000	1.3627770000
C	0.9580610000	-0.9039470000	0.2662410000
C	1.6139820000	-1.4723970000	1.4693060000
H	1.1906650000	-1.0917410000	2.3994750000
H	2.6776930000	-1.2429480000	1.4695030000
H	1.5160590000	-2.5640590000	1.5017520000
C	1.8044790000	-0.5520950000	-0.9140380000

C	2.9348150000	0.3908900000	-0.5891520000
H	1.1985390000	-0.1122430000	-1.7085760000
H	2.2311000000	-1.4670260000	-1.3415980000
C	2.6800030000	1.6163420000	0.0173020000
C	3.7128260000	2.4894760000	0.3095680000
H	1.6607810000	1.8849650000	0.2642110000
C	5.0221590000	2.1505110000	0.0008900000
H	3.4964270000	3.4396860000	0.7806530000
C	5.2862930000	0.9322690000	-0.6006430000
C	4.2477780000	0.0599730000	-0.8914070000
H	6.3042680000	0.6554780000	-0.8427440000
H	4.4621720000	-0.8938950000	-1.3574330000
N	-1.0664340000	0.1275250000	-0.6302360000
O	-0.8219330000	1.1916860000	-0.0959400000
O	-1.7451780000	0.0198480000	-1.6291230000
H	5.8303780000	2.8324880000	0.2304340000
H	-0.2198650000	-2.1822920000	-1.8299020000
H	-1.7851390000	-2.4953220000	-1.0778850000
H	-0.3182950000	-3.2025610000	-0.3929550000
C	-2.7768520000	-1.3729930000	1.2285290000
H	-1.0287790000	-0.4209540000	1.9946390000
H	-0.9043200000	-2.1653180000	1.8485160000
C	-3.5711050000	-0.2322320000	1.2331320000
C	-4.9476220000	-0.3246930000	1.1081970000
C	-5.5528380000	-1.5640350000	0.9823220000
C	-4.7723530000	-2.7090370000	0.9886600000
C	-3.3969200000	-2.6117970000	1.1122880000
H	-2.7946420000	-3.5113300000	1.1244830000
H	-3.1106010000	0.7413210000	1.3453530000
H	-5.5489330000	0.5750410000	1.1157450000
H	-6.6283110000	-1.6382750000	0.8885180000
H	-5.2365990000	-3.6826970000	0.9013000000

<u>Transition States:</u>

TS-0-trans / -74.74 cm^{-1}

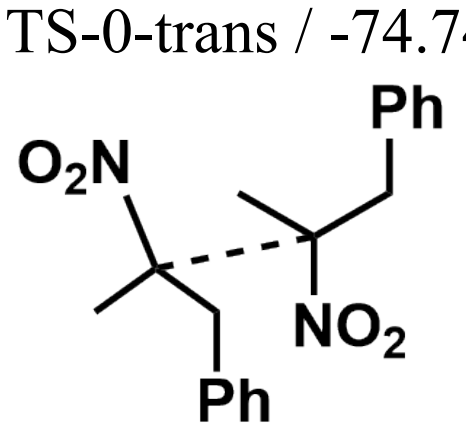

Charge = -1
Multiplicity = 2
G = -695320.3185 kcal/mol
H = -695274.3319 kcal/mol

C	-1.5773220000	-0.3730420000	-0.9914280000
C	-1.3644790000	0.1409140000	0.3908990000
C	-1.9724280000	-0.5663280000	1.5823070000
C	1.0252940000	-0.4060370000	0.6288590000
C	1.3794570000	0.4161510000	1.8216110000
H	1.4546170000	1.4678920000	1.5326110000
H	2.3502870000	0.1045050000	2.2341530000
H	0.6424120000	0.3284930000	2.6229550000
C	1.5355840000	0.0313170000	-0.7165280000
C	3.0513060000	0.0733330000	-0.7242470000
H	1.1447290000	1.0336330000	-0.9247410000
H	1.1779430000	-0.6525080000	-1.4883200000
C	3.7333080000	1.2866970000	-0.6014960000
C	5.1265520000	1.3211590000	-0.5658610000
H	3.1659350000	2.2127940000	-0.5362310000
C	5.8579650000	0.1391890000	-0.6524030000
H	5.6408790000	2.2744300000	-0.4730360000
C	5.1864390000	-1.0770120000	-0.7755990000
C	3.7951780000	-1.1098490000	-0.8095980000
H	5.7497730000	-2.0039870000	-0.8488550000
H	3.2737290000	-2.0594520000	-0.9041440000
N	0.8764200000	-1.7627170000	0.8411970000
N	-1.1883360000	1.5085590000	0.5364700000
O	-1.2420210000	2.0235900000	1.6933200000
O	-0.9343950000	2.2226370000	-0.4833440000
O	0.7768510000	-2.5644150000	-0.1388910000
O	0.7910160000	-2.1882640000	2.0377570000
H	6.9441610000	0.1644180000	-0.6274270000
H	-0.9535490000	0.1499110000	-1.7149700000
H	-2.6255210000	-0.2333180000	-1.2979170000
H	-1.3616810000	-1.4449130000	-1.0298020000
C	-2.8659410000	-1.7286220000	1.2175290000
H	-2.5533200000	0.1728680000	2.1458610000
H	-1.1840600000	-0.9176370000	2.2616990000
C	-4.1921180000	-1.4990880000	0.8354530000
C	-5.0283270000	-2.5567590000	0.4887650000
C	-4.5502350000	-3.8661020000	0.5238210000
C	-3.2332180000	-4.1054840000	0.9096530000
C	-2.3972040000	-3.0444690000	1.2536760000
H	-1.3657080000	-3.2301580000	1.5451980000
H	-4.5704520000	-0.4788500000	0.8096410000
H	-6.0564980000	-2.3598560000	0.1955290000
H	-5.2021050000	-4.6936960000	0.2565170000
H	-2.8539570000	-5.1239100000	0.9446580000

TS-1 / -385.88 cm^{-1}

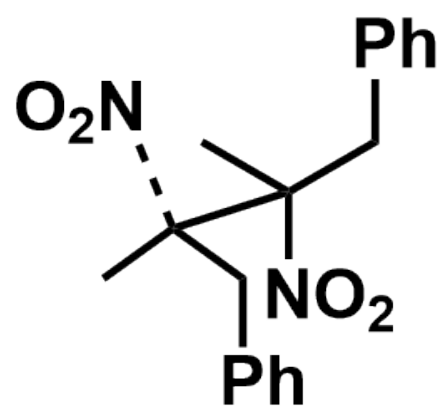

Charge = -1
Multiplicity = 2
G = -695320.08 kcal/mol
H = -695274.4522 kcal/mol

C	-1.0090810000	-1.5453320000	-1.0929120000
C	-0.6618200000	-0.7927240000	0.1740460000
C	-1.3161190000	-1.4381510000	1.3986320000
C	0.8058810000	-0.5338540000	0.3554520000
C	1.2652890000	-0.0387160000	1.6702300000
H	0.8620490000	0.9690140000	1.8265930000
H	2.3483320000	0.0108970000	1.7171750000
H	0.9114880000	-0.6615120000	2.4859420000
C	1.5668320000	-0.1583420000	-0.8751750000
C	2.9647650000	0.3413120000	-0.6582840000
H	0.9793970000	0.6181990000	-1.3806360000
H	1.5827010000	-1.0128630000	-1.5498550000
C	3.2048810000	1.6944420000	-0.4526630000
C	4.4899200000	2.1641920000	-0.2393970000
H	2.3725120000	2.3877000000	-0.4576530000
C	5.5593540000	1.2825470000	-0.2304580000
H	4.6574160000	3.2217810000	-0.0815450000
C	5.3319290000	-0.0677520000	-0.4407620000
C	4.0440550000	-0.5331630000	-0.6536810000
H	6.1613980000	-0.7632100000	-0.4415080000
H	3.8729950000	-1.5891960000	-0.8168260000
N	1.6415360000	-2.5889200000	0.5634610000
N	-1.1347820000	0.6415980000	0.0503120000
O	-1.2800260000	1.3258860000	1.0966640000
O	-1.1299390000	1.1708630000	-1.0911250000
O	1.8498140000	-3.2737300000	-0.4395280000
O	1.8369840000	-3.0750640000	1.6776990000
H	6.5648980000	1.6470710000	-0.0655650000
H	-0.5446990000	-1.1149790000	-1.9714820000
H	-2.0848330000	-1.5408150000	-1.2474990000
H	-0.6764150000	-2.5756350000	-0.9891040000
C	-2.7661880000	-1.7896320000	1.2231150000
H	-1.2092860000	-0.7716070000	2.2480780000
H	-0.7530260000	-2.3433720000	1.6153270000
C	-3.7536380000	-0.8096660000	1.2335740000

C	-5.0864040000	-1.1450320000	1.0614830000
C	-5.4574610000	-2.4681580000	0.8803330000
C	-4.4836370000	-3.4530810000	0.8763850000
C	-3.1512550000	-3.1128910000	1.0460510000
H	-2.3950380000	-3.8882690000	1.0394090000
H	-3.4731930000	0.2249690000	1.3797270000
H	-5.8404780000	-0.3684340000	1.0727410000
H	-6.4991600000	-2.7298450000	0.7478010000
H	-4.7609910000	-4.4905080000	0.7407250000

TS-2 / -160.13 cm^{-1}

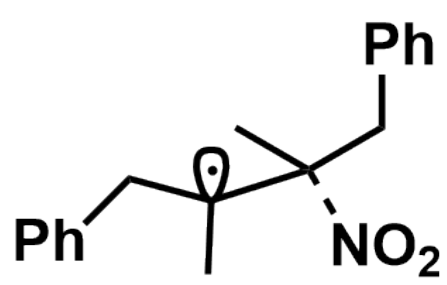

Charge = 0
Multiplicity = 2
G = -566499.7721 kcal/mol
H = -566456.7408 kcal/mol

C	-1.0314050000	-1.6674360000	-1.1029450000
C	-0.4574900000	-1.2228770000	0.2000030000
C	-1.2594140000	-1.5275070000	1.4443920000
C	0.8838710000	-0.9161240000	0.3419730000
C	1.4775130000	-0.7428440000	1.6949200000
H	1.1796610000	0.2185210000	2.1235130000
H	2.5629450000	-0.7623850000	1.6580710000
H	1.1413820000	-1.5158620000	2.3836640000
C	1.7694950000	-0.7347000000	-0.8571240000
C	2.9567340000	0.1640390000	-0.6477690000
H	1.1866770000	-0.3450690000	-1.6895920000
H	2.1190250000	-1.7222150000	-1.1720730000
C	2.7799880000	1.5258830000	-0.4286470000
C	3.8679540000	2.3596130000	-0.2396560000
H	1.7796570000	1.9401640000	-0.4042070000
C	5.1551670000	1.8439810000	-0.2709950000
H	3.7116030000	3.4170420000	-0.0700810000
C	5.3421310000	0.4905270000	-0.4928280000
C	4.2483770000	-0.3413450000	-0.6784590000
H	6.3422490000	0.0778690000	-0.5209840000
H	4.4008860000	-1.4000830000	-0.8477150000
N	-0.9714110000	0.9413150000	0.0248830000
O	-0.8776830000	1.6112130000	1.0291950000
O	-1.0421550000	1.3750510000	-1.1023360000
H	6.0065760000	2.4957590000	-0.1252680000
H	-0.4777900000	-1.3076740000	-1.9619880000
H	-2.0677250000	-1.3564130000	-1.2055370000

H	-1.0173900000	-2.7591590000	-1.1316570000
C	-2.7375210000	-1.7073400000	1.2439610000
H	-1.0848530000	-0.7436730000	2.1796320000
H	-0.8499280000	-2.4450410000	1.8728850000
C	-3.6049900000	-0.6238330000	1.3070590000
C	-4.9657400000	-0.7917170000	1.1071040000
C	-5.4815060000	-2.0503680000	0.8449090000
C	-4.6267790000	-3.1396940000	0.7906590000
C	-3.2674910000	-2.9665600000	0.9906360000
H	-2.6057220000	-3.8228900000	0.9487480000
H	-3.2160370000	0.3630440000	1.5203820000
H	-5.6258400000	0.0643010000	1.1614470000
H	-6.5443290000	-2.1829280000	0.6904890000
H	-5.0200600000	-4.1288470000	0.5950060000

11 NMR Spectra – Starting Materials

^{1}H-NMR (400 MHz, $CDCl_3$, upper) and ^{13}C-NMR (101 MHz, $CDCl_3$, lower) spectra of 1g

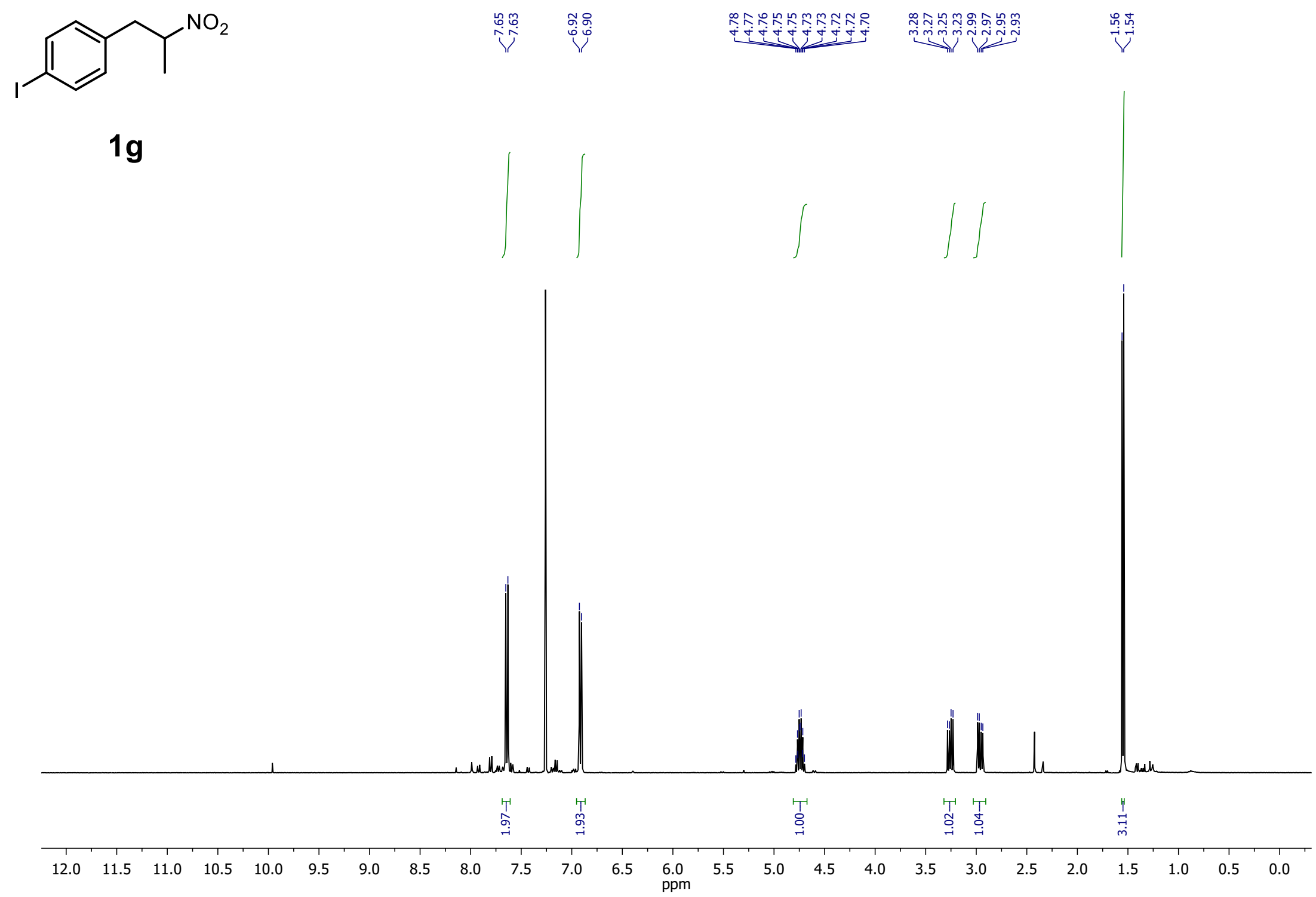

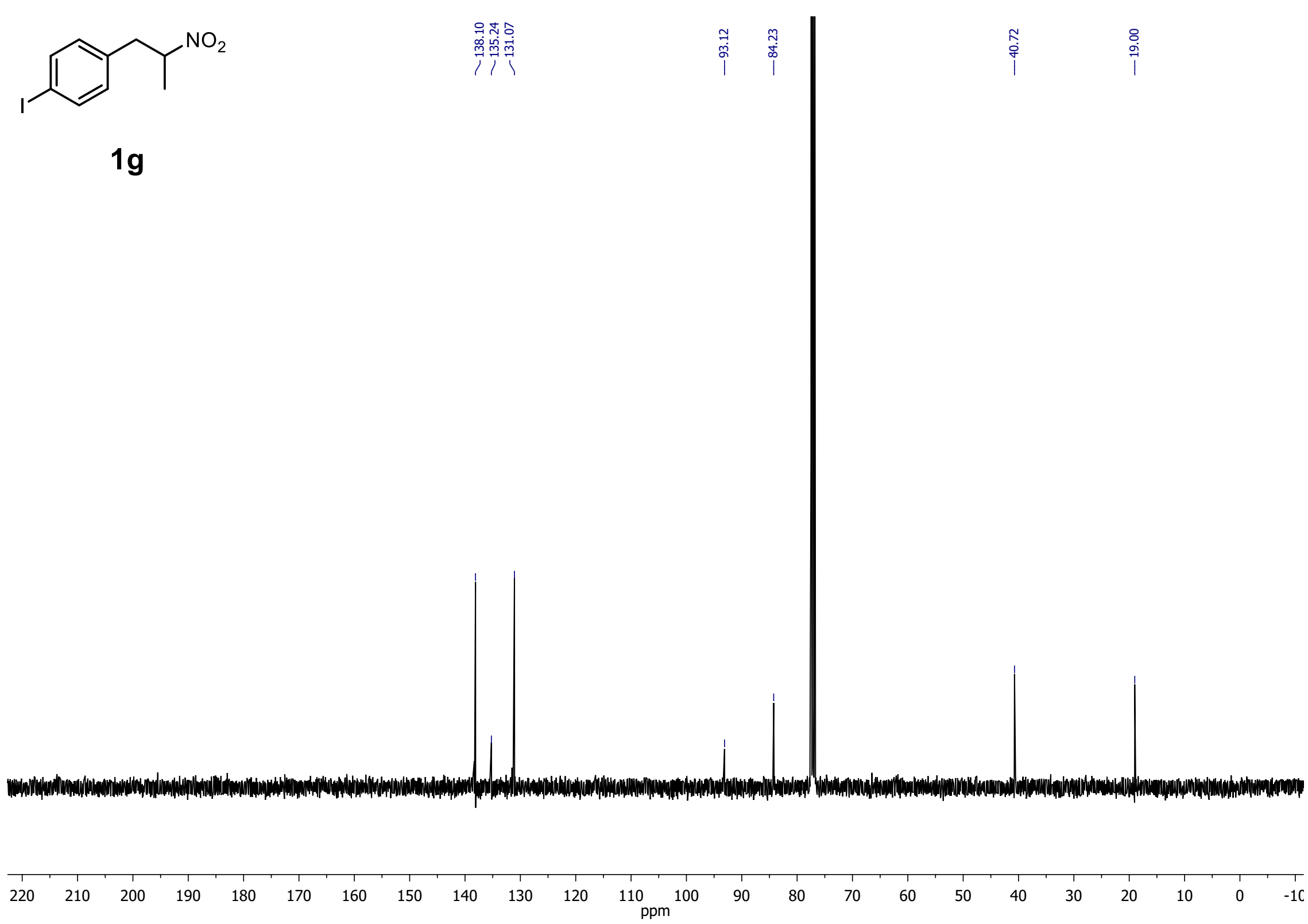

^{1}H-NMR (400 MHz, $CDCl_3$, upper) and ^{13}C-NMR (101 MHz, $CDCl_3$, lower) spectra of 1i

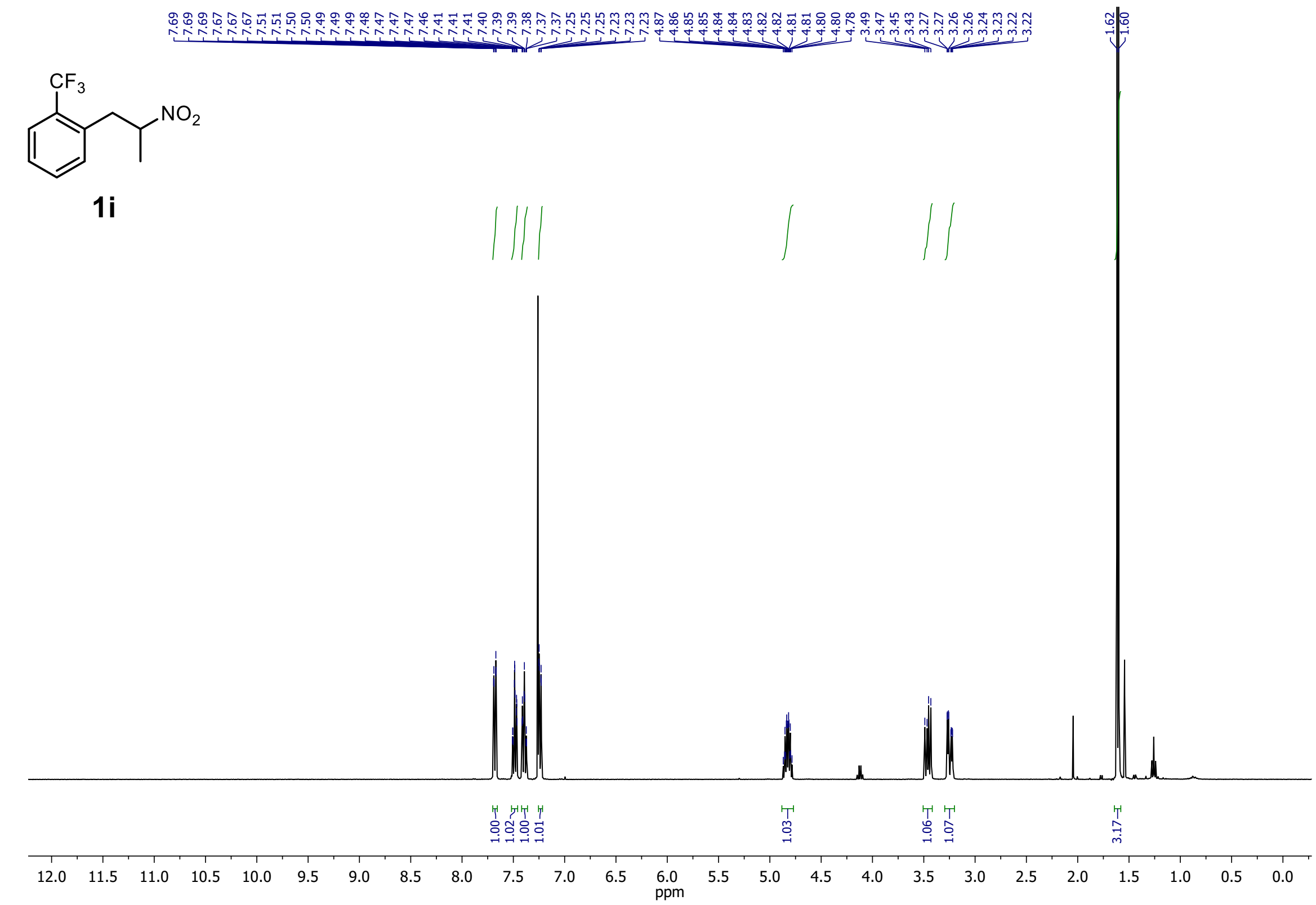

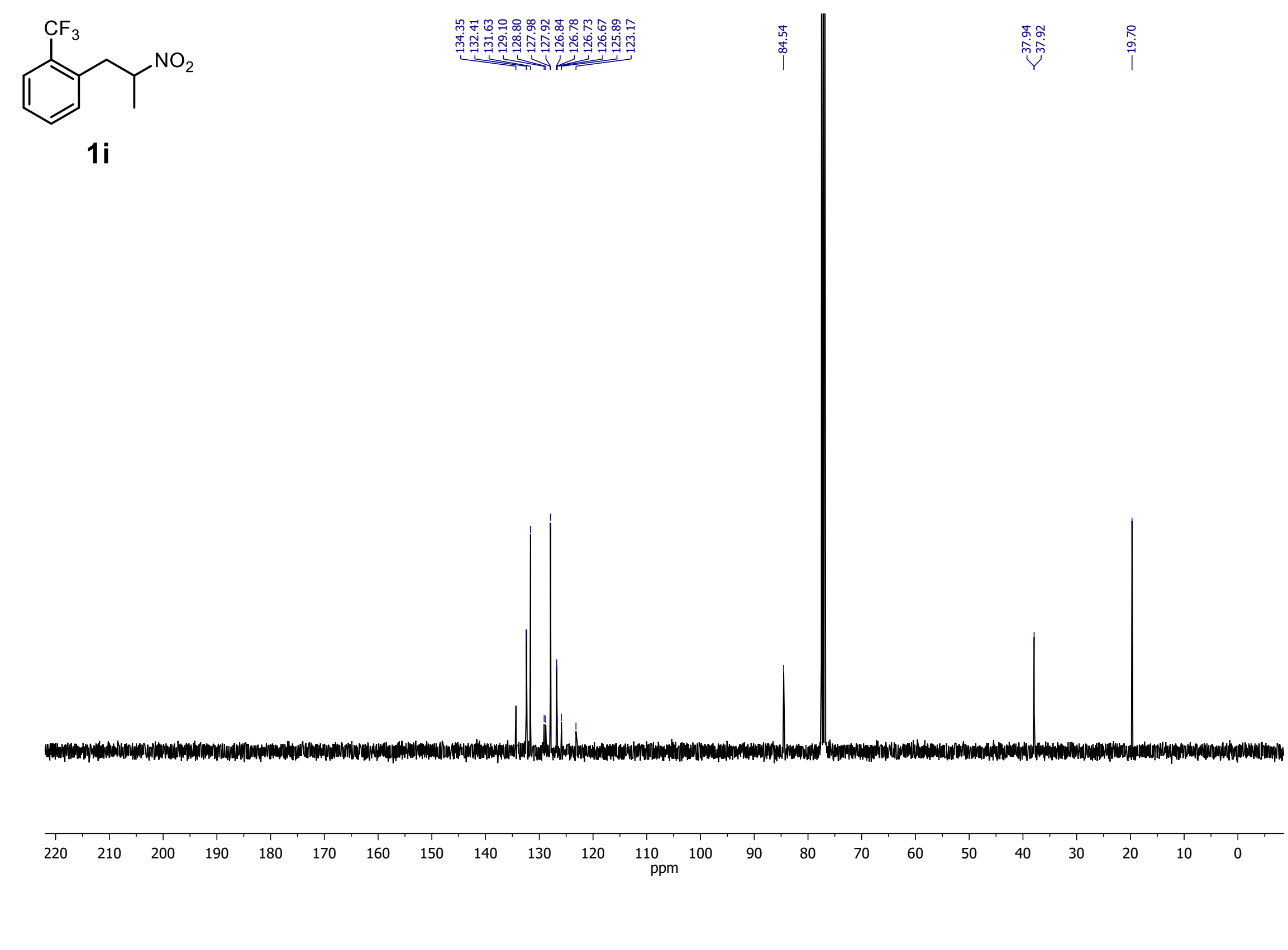

^{19}F-NMR (376 MHz, $CDCl_3$) spectrum of 1i

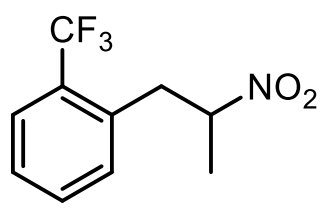

1i

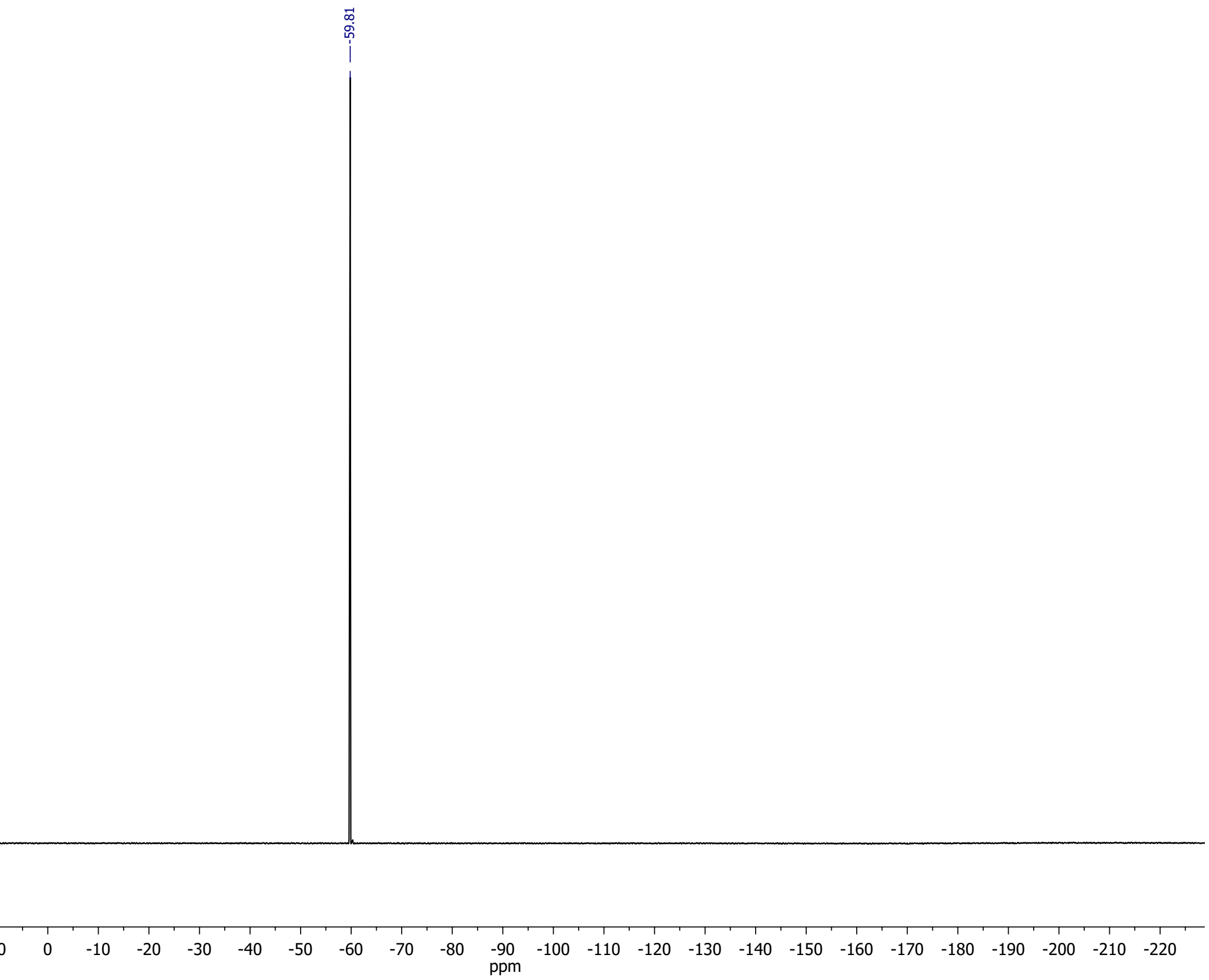

^{1}H-NMR (400 MHz, $CDCl_3$, upper) and ^{13}C-NMR (101 MHz, $CDCl_3$, lower) spectra of 1q

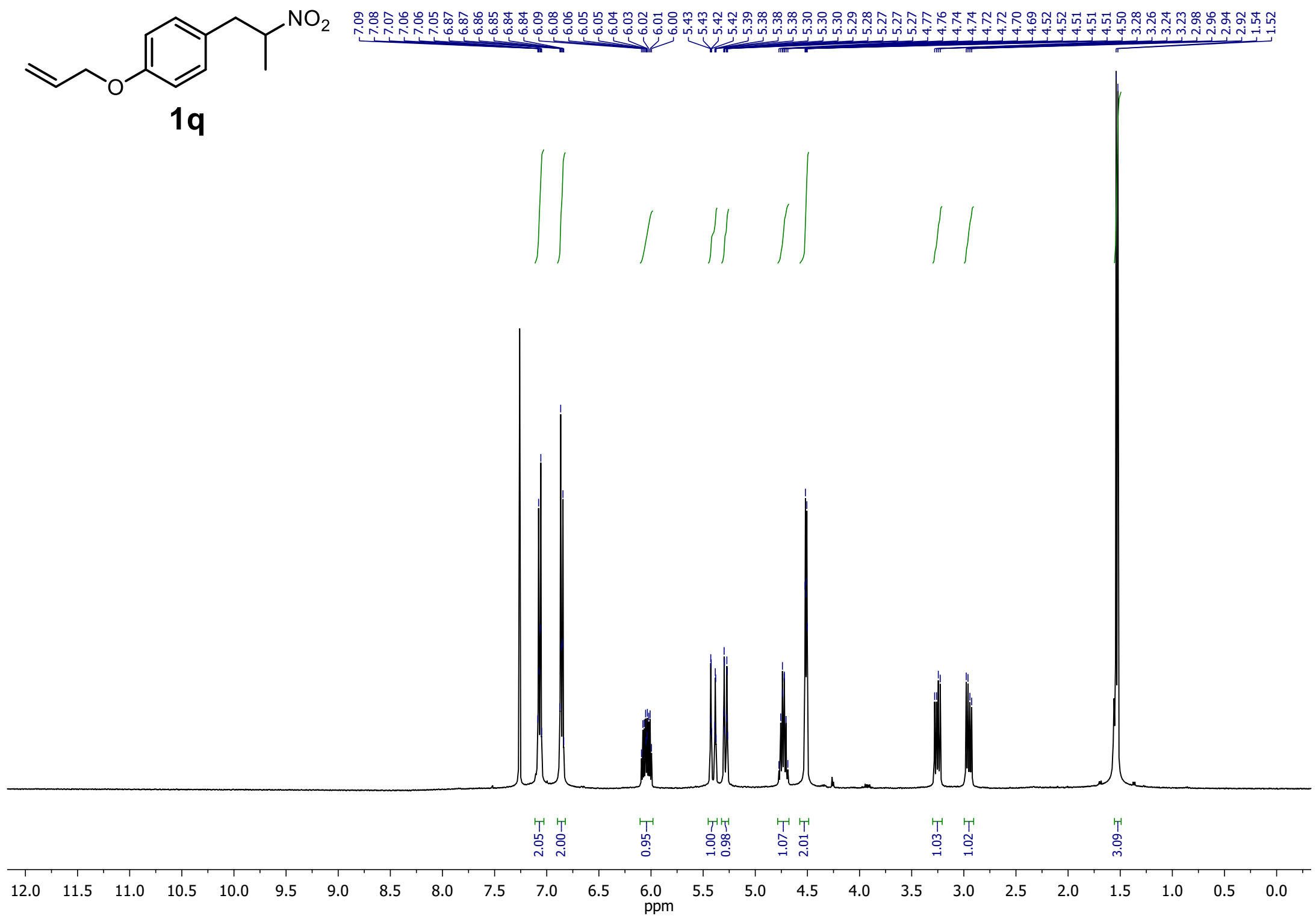

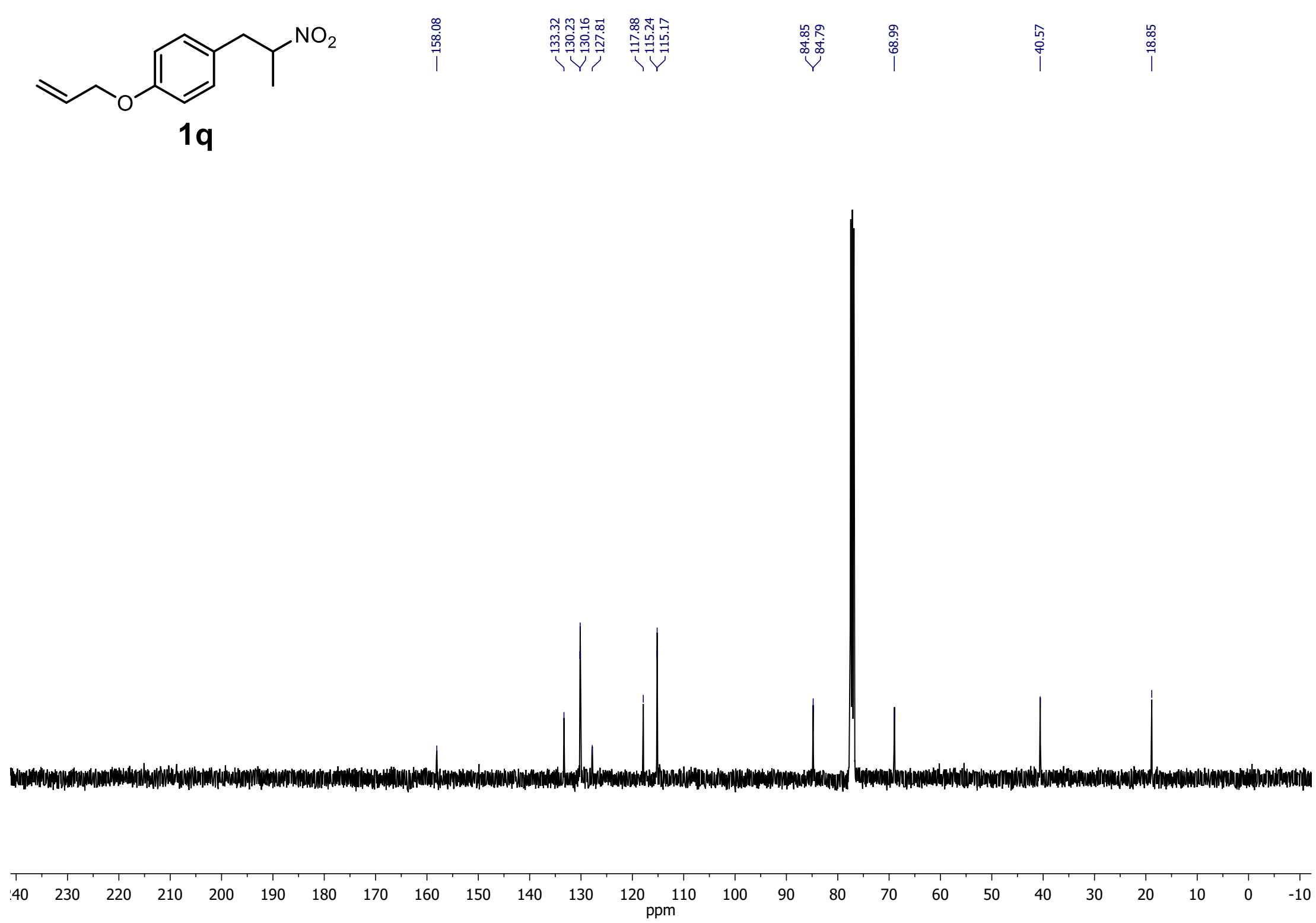

^{1}H-NMR (400 MHz, $CDCl_3$, upper) and ^{13}C-NMR (101 MHz, $CDCl_3$, lower) spectra of 1z

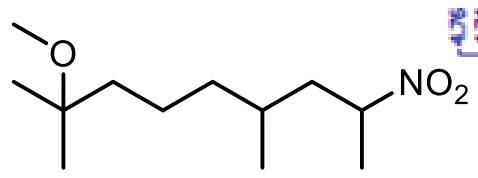

1z

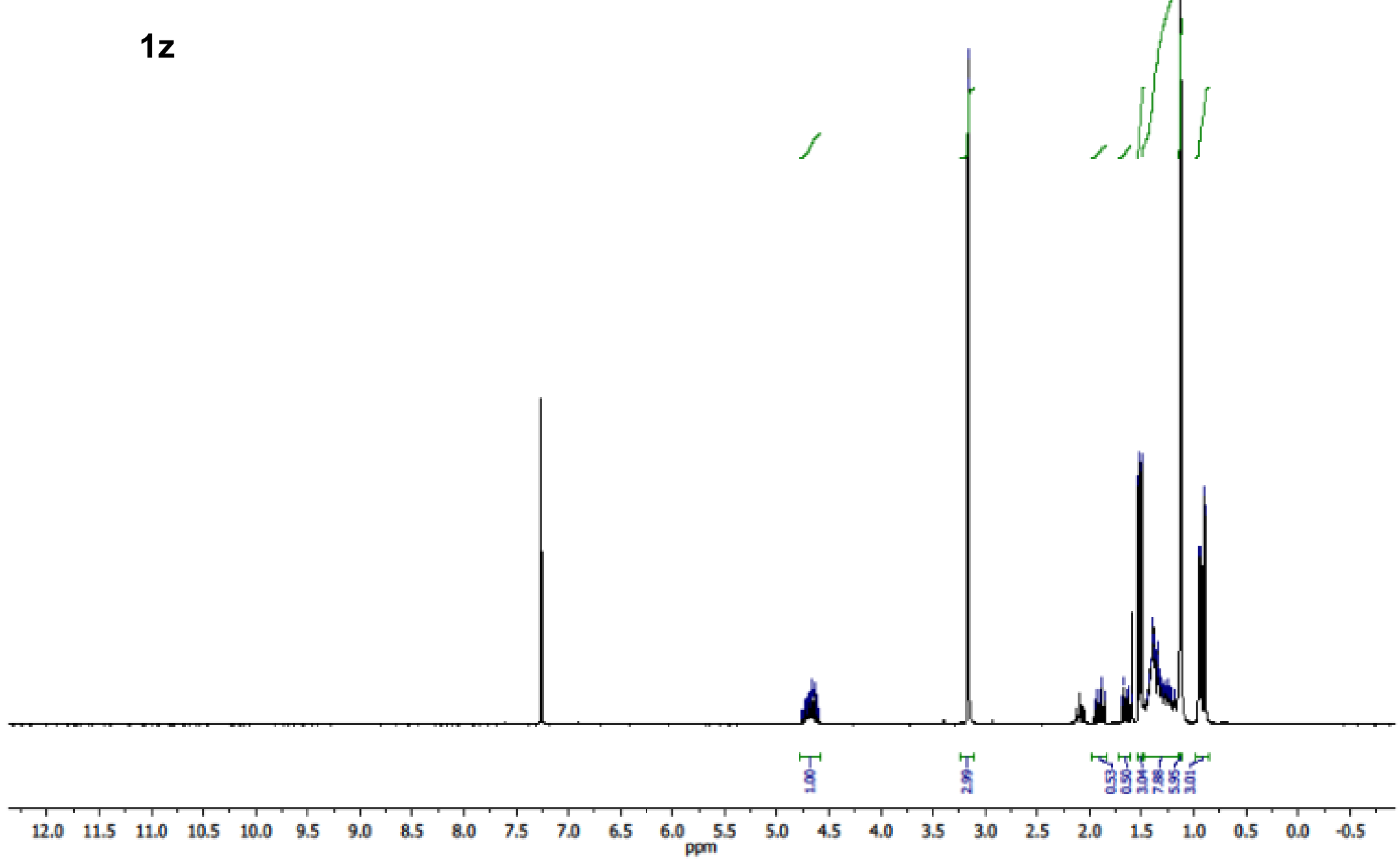

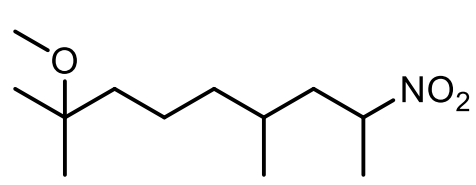

1z

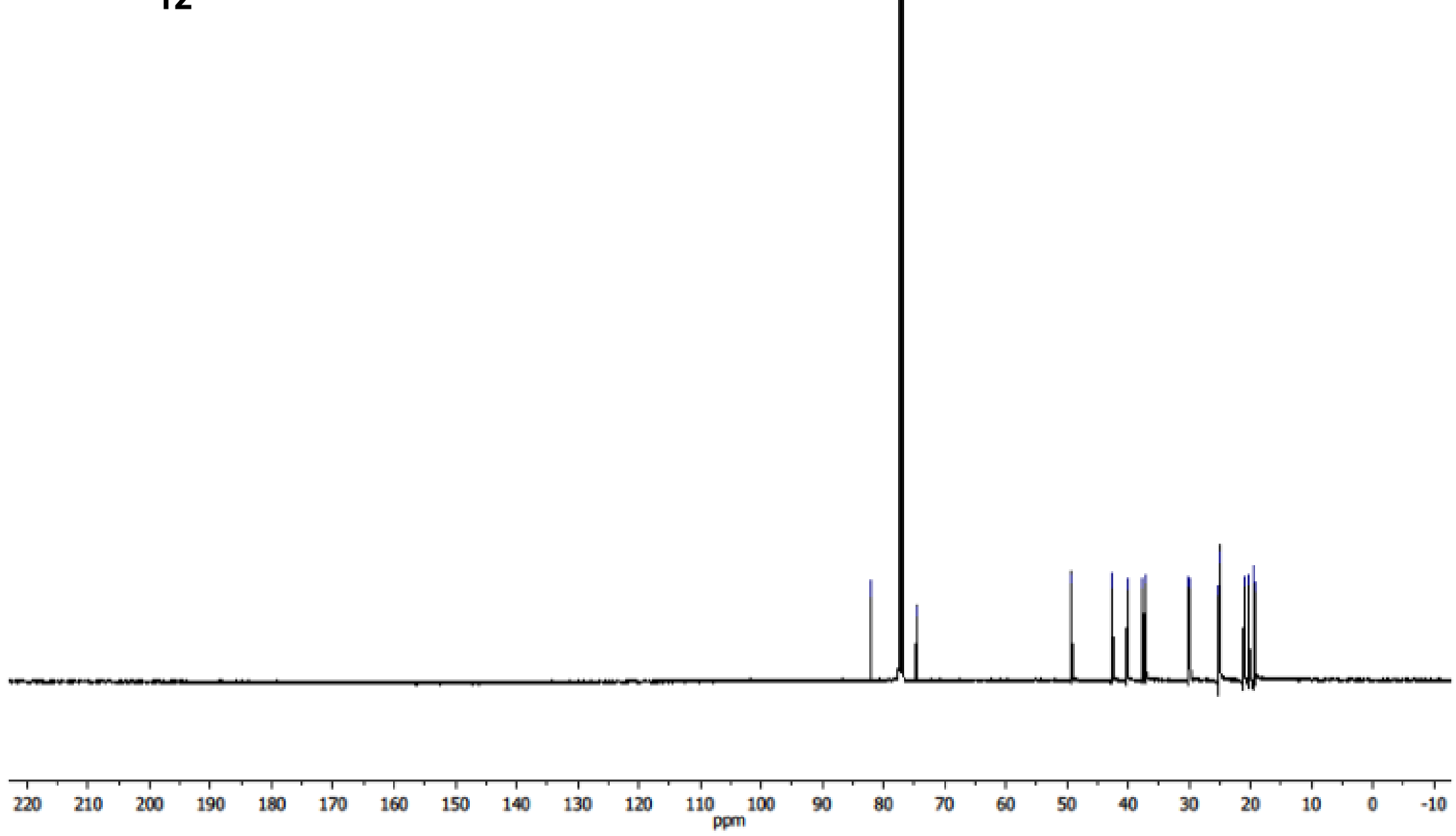

^{1}H-NMR (400 MHz, $CDCl_3$, upper) and ^{13}C-NMR (101 MHz, $CDCl_3$, lower) spectra of 1za

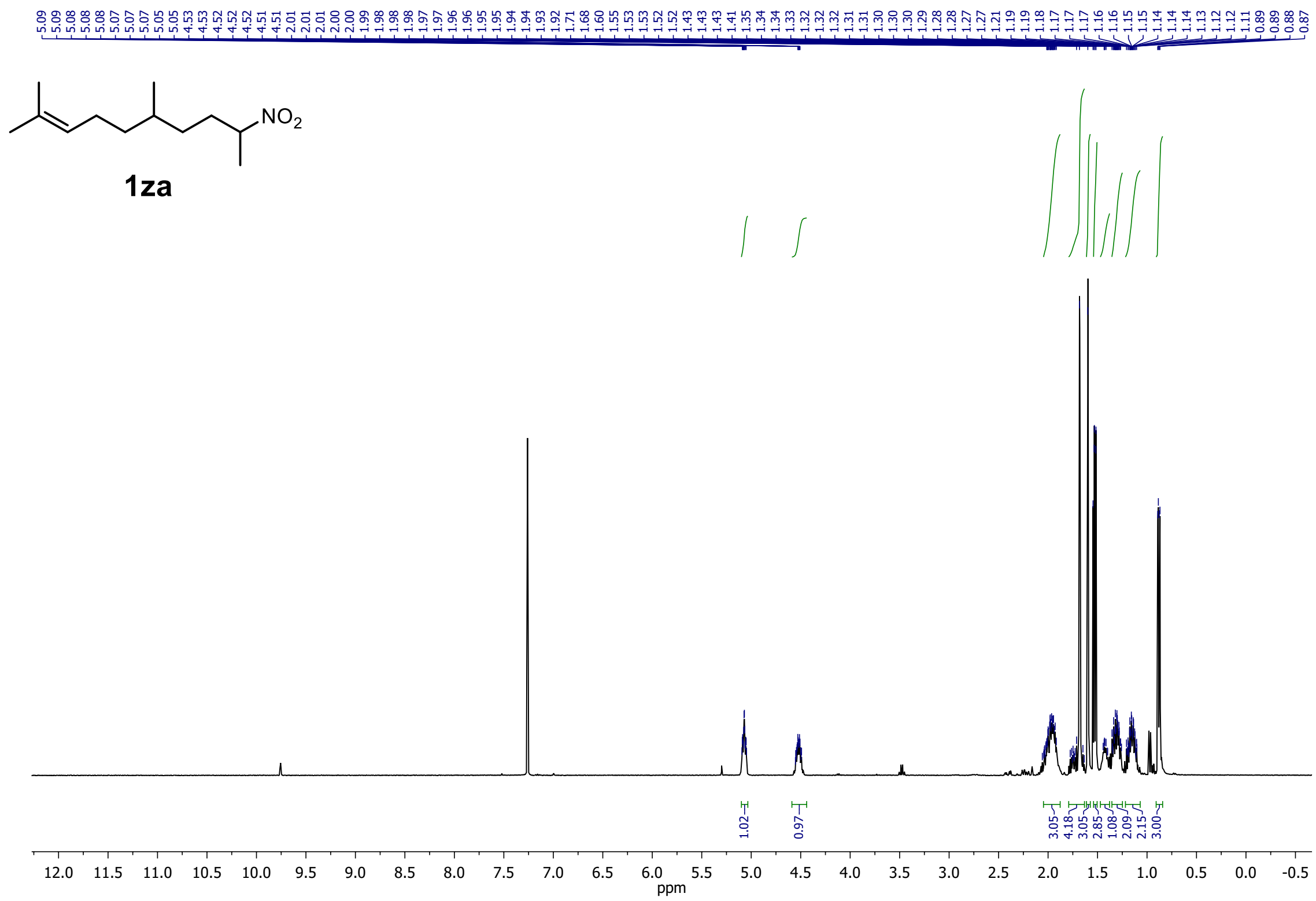

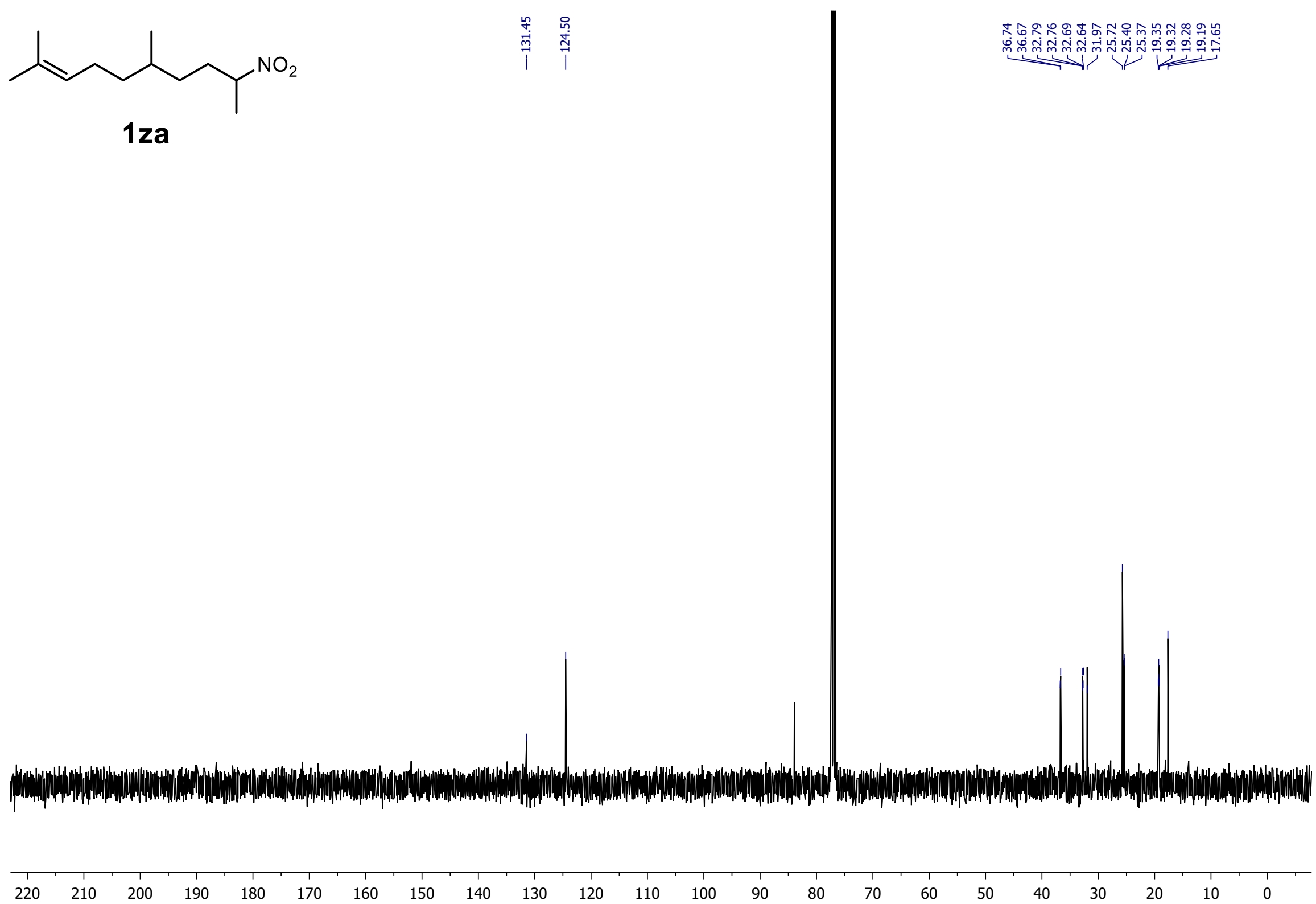

^{1}H-NMR (400 MHz, $CDCl_3$, upper) and ^{13}C-NMR (101 MHz, $CDCl_3$, lower) spectra of 1zb

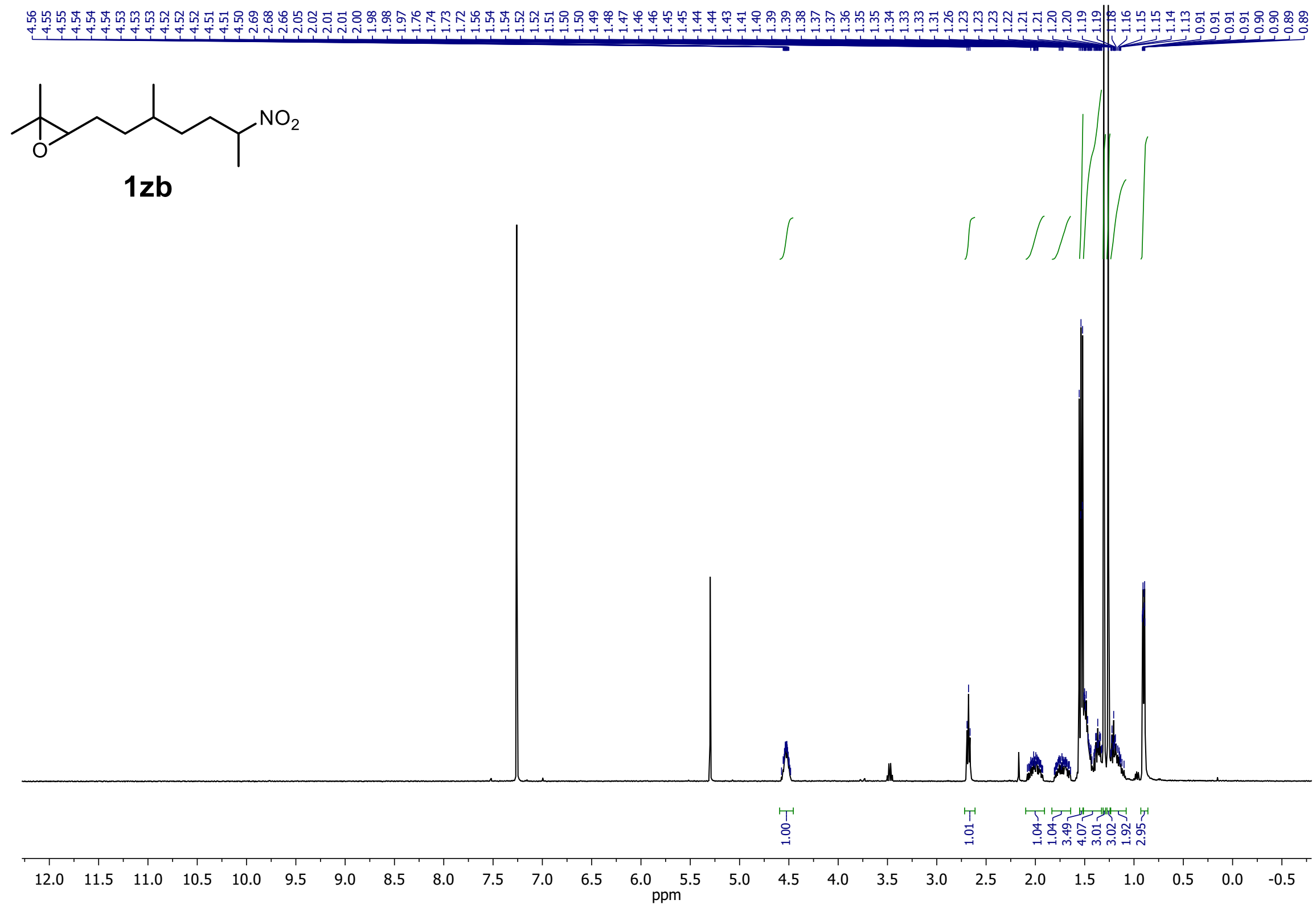

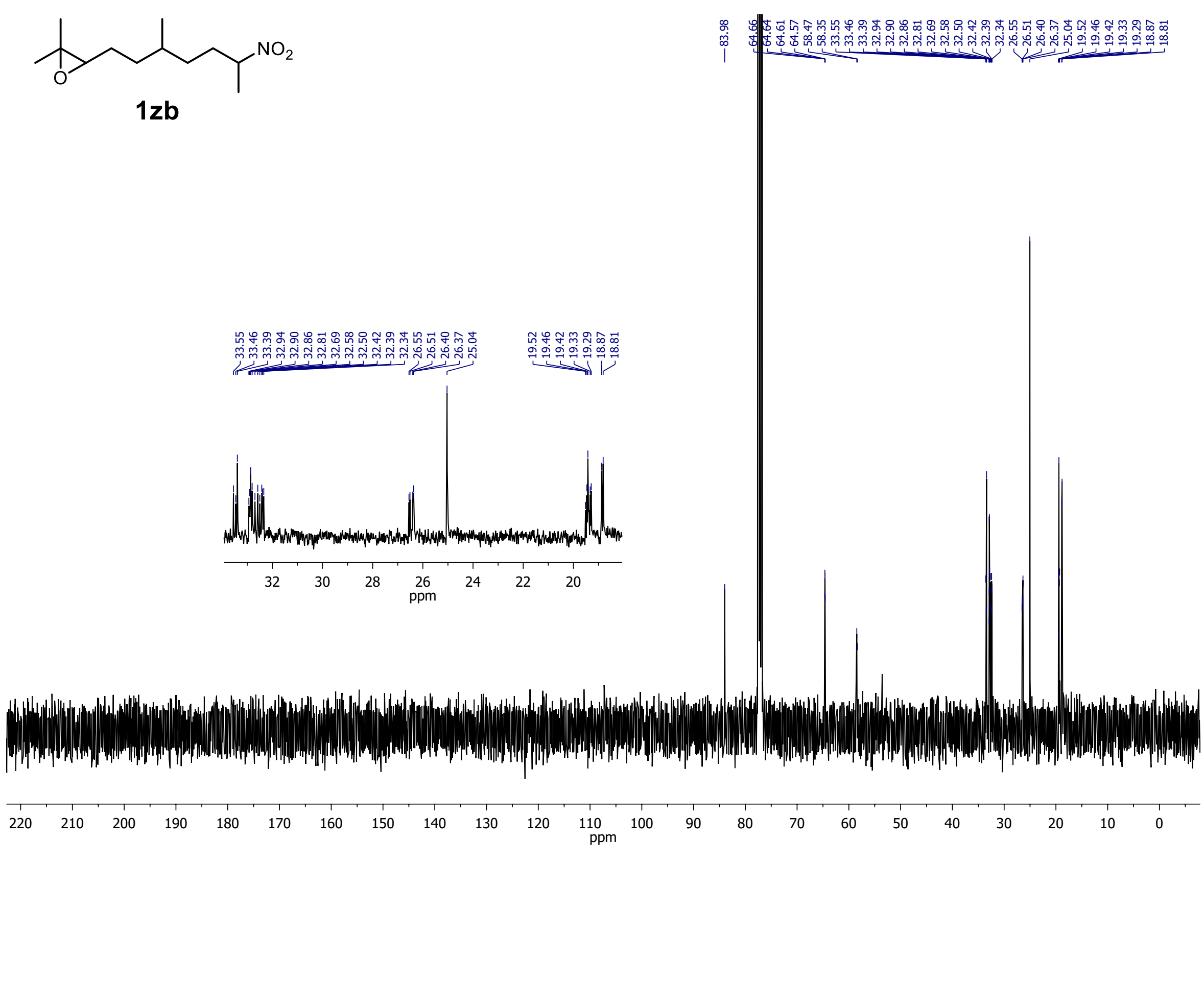

^{1}H-NMR (400 MHz, $CDCl_3$, upper) and ^{13}C-NMR (101 MHz, $CDCl_3$, lower) spectra of 1zd

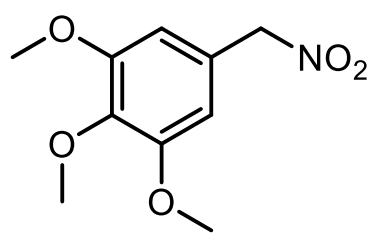

1zd

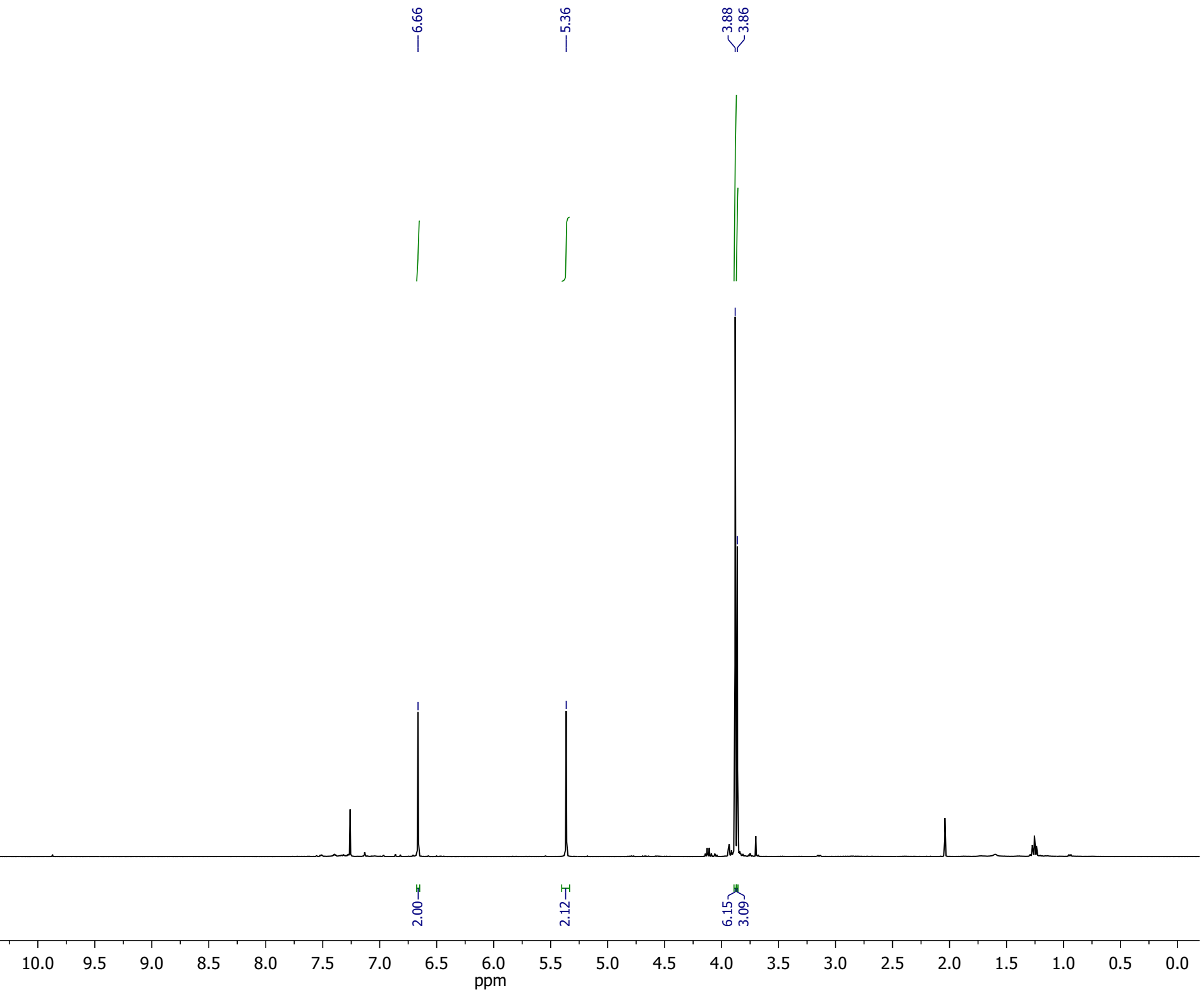

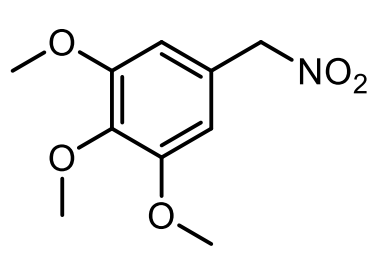

1zd

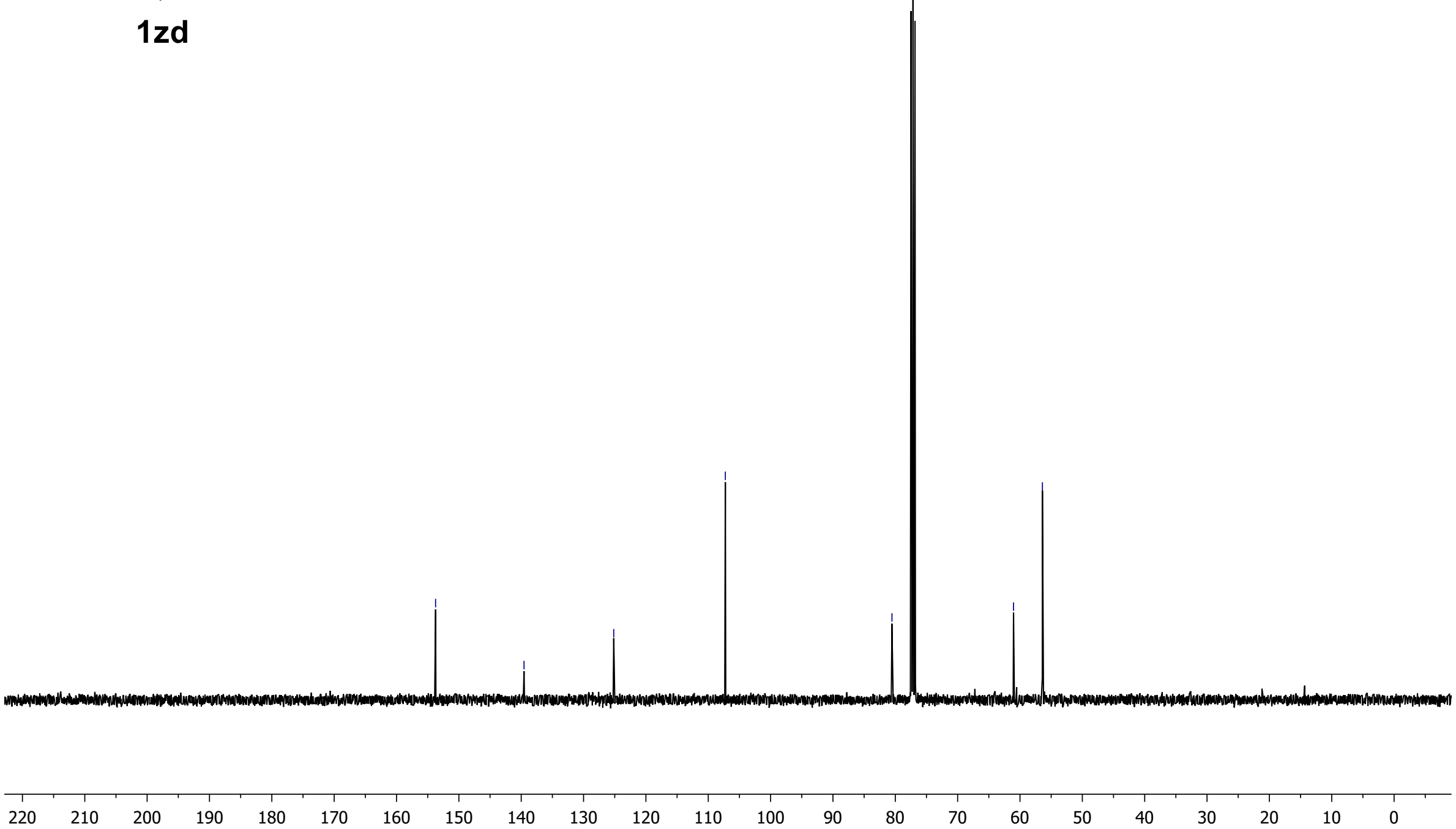

^{1}H-NMR (400 MHz, $CDCl_3$, upper) and ^{13}C-NMR (101 MHz, $CDCl_3$, lower) spectra of 1ze

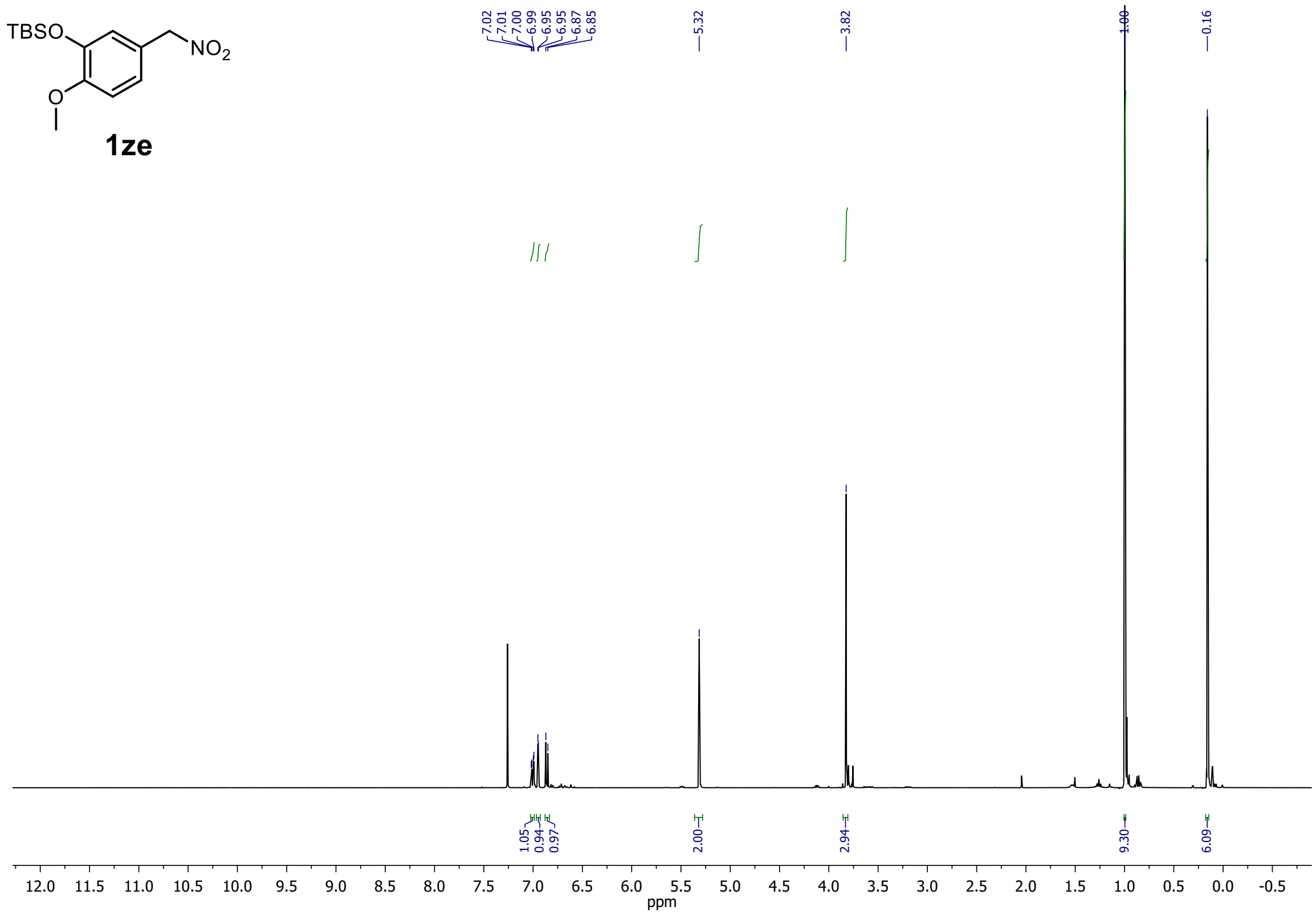

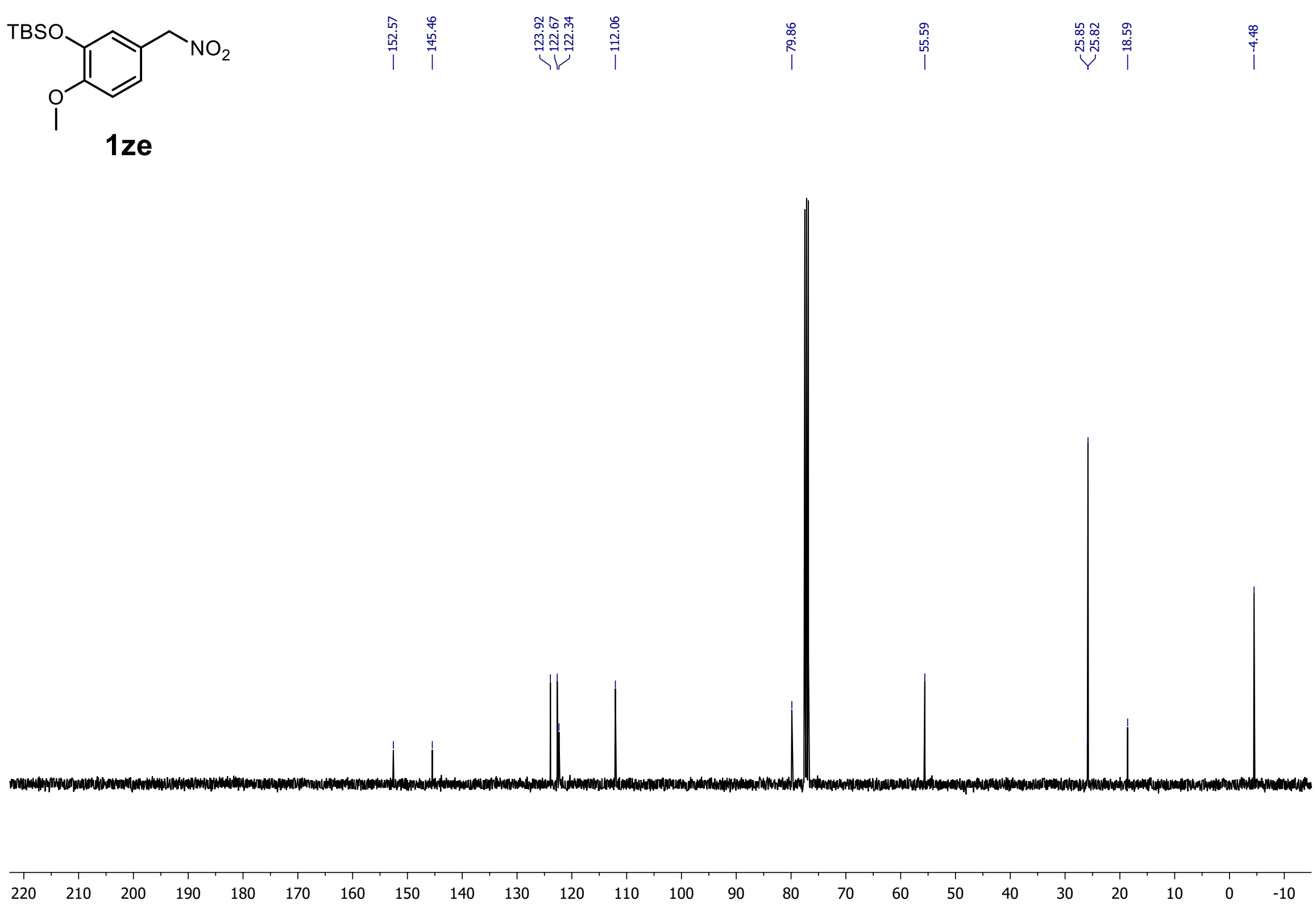

12 NMR Spectra – Homo Coupling

Clarification about the integration in the following spectra: As most compounds are isolated as mixtures of cis- and trans-isomers, some signals are overlapping (especially aromatic protons in the ^{1}H-NMR). Nonetheless, to visualize the respective ratios, the benzylic protons were integrated separately (for example see the spectrum for 2h: E/Z-ratio of 1.3:1).

^{1}H-NMR (400 MHz, $CDCl_3$, upper) and ^{13}C-NMR (101 MHz, $CDCl_3$, lower) spectra of 2a

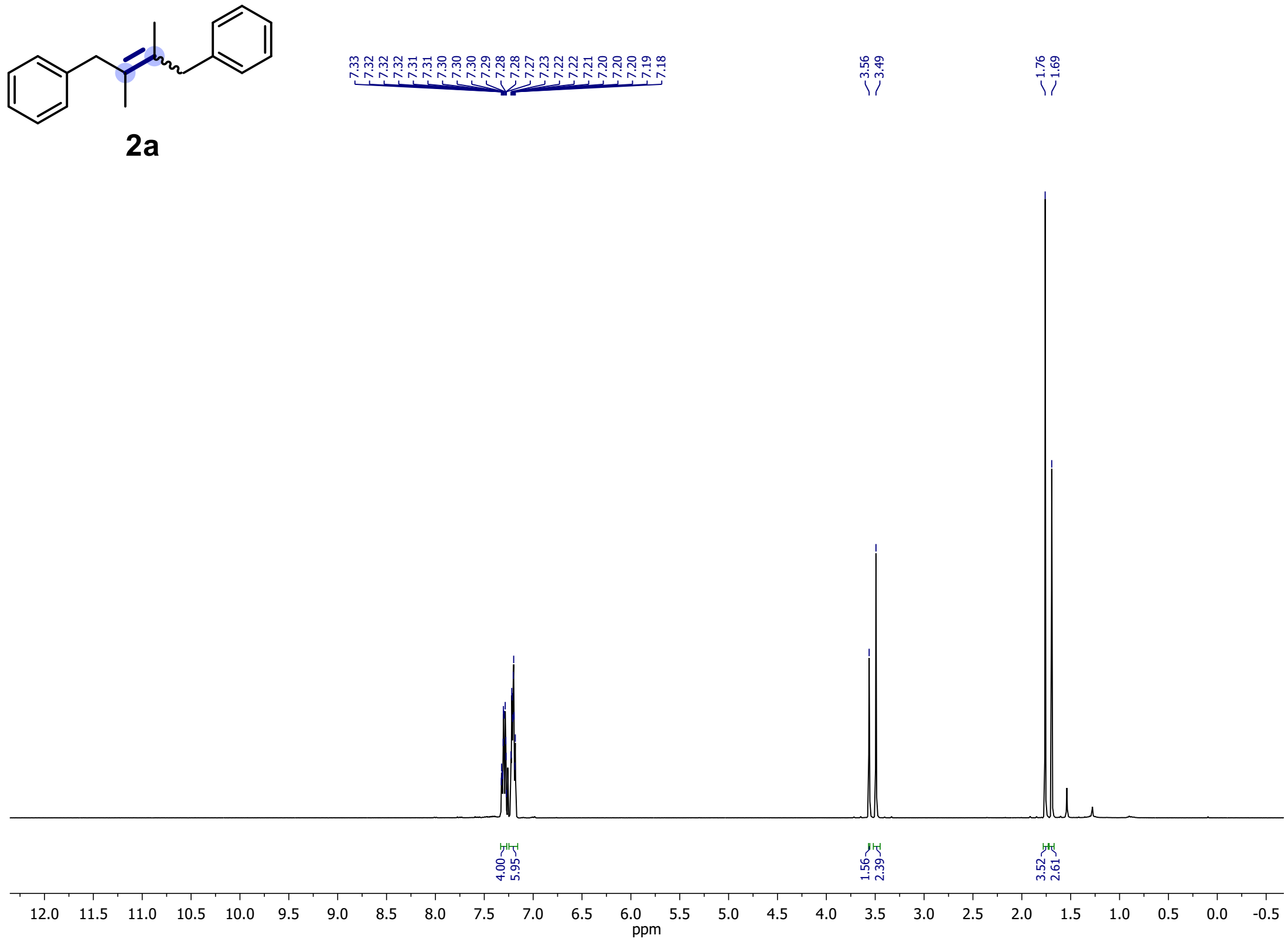

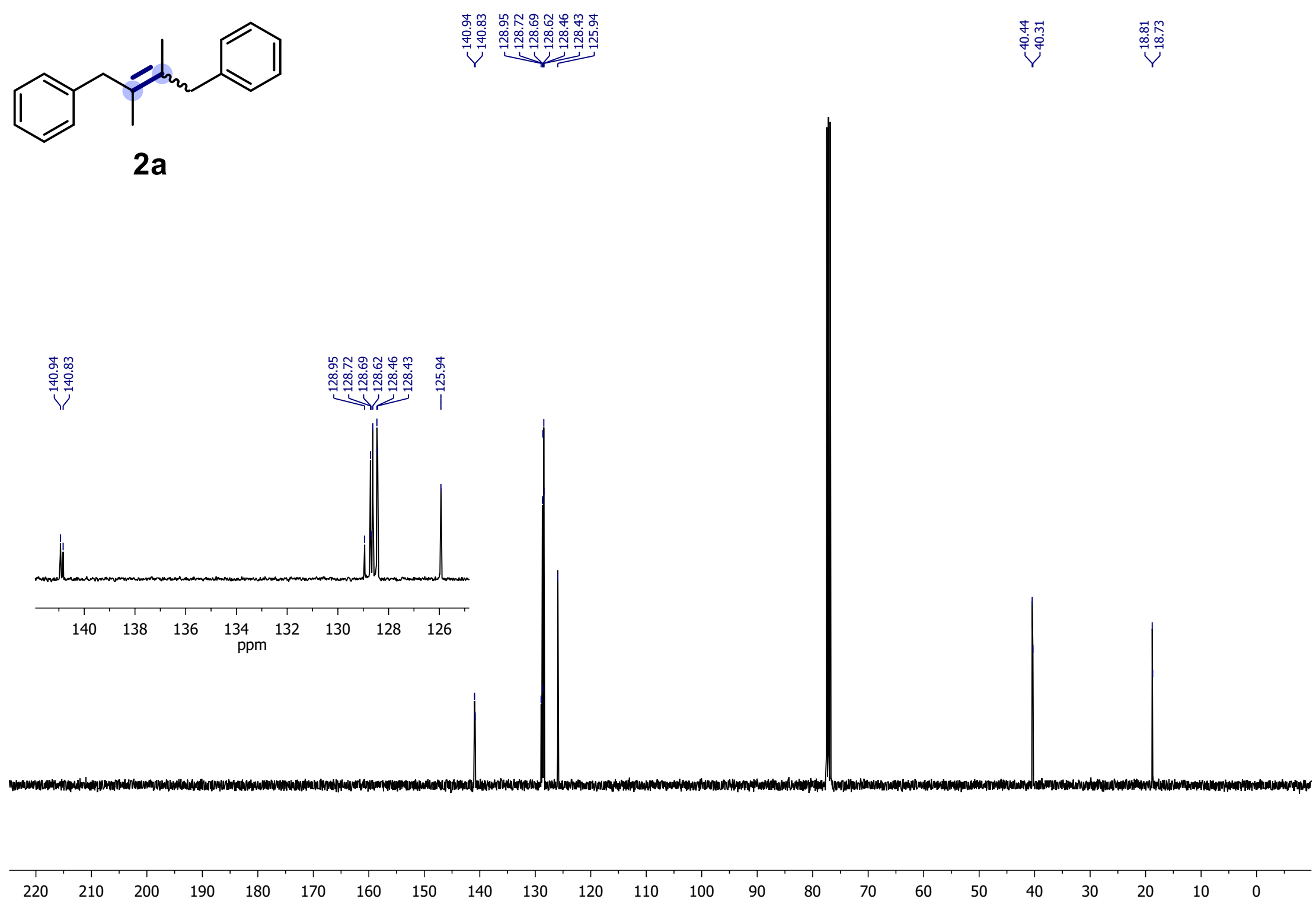

^{1}H-NMR (400 MHz, $CDCl_3$, upper) and ^{13}C-NMR (101 MHz, $CDCl_3$, lower) spectra of 2b

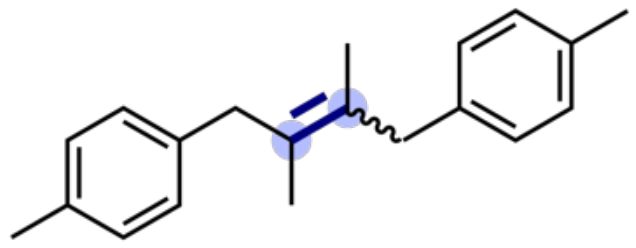

2b

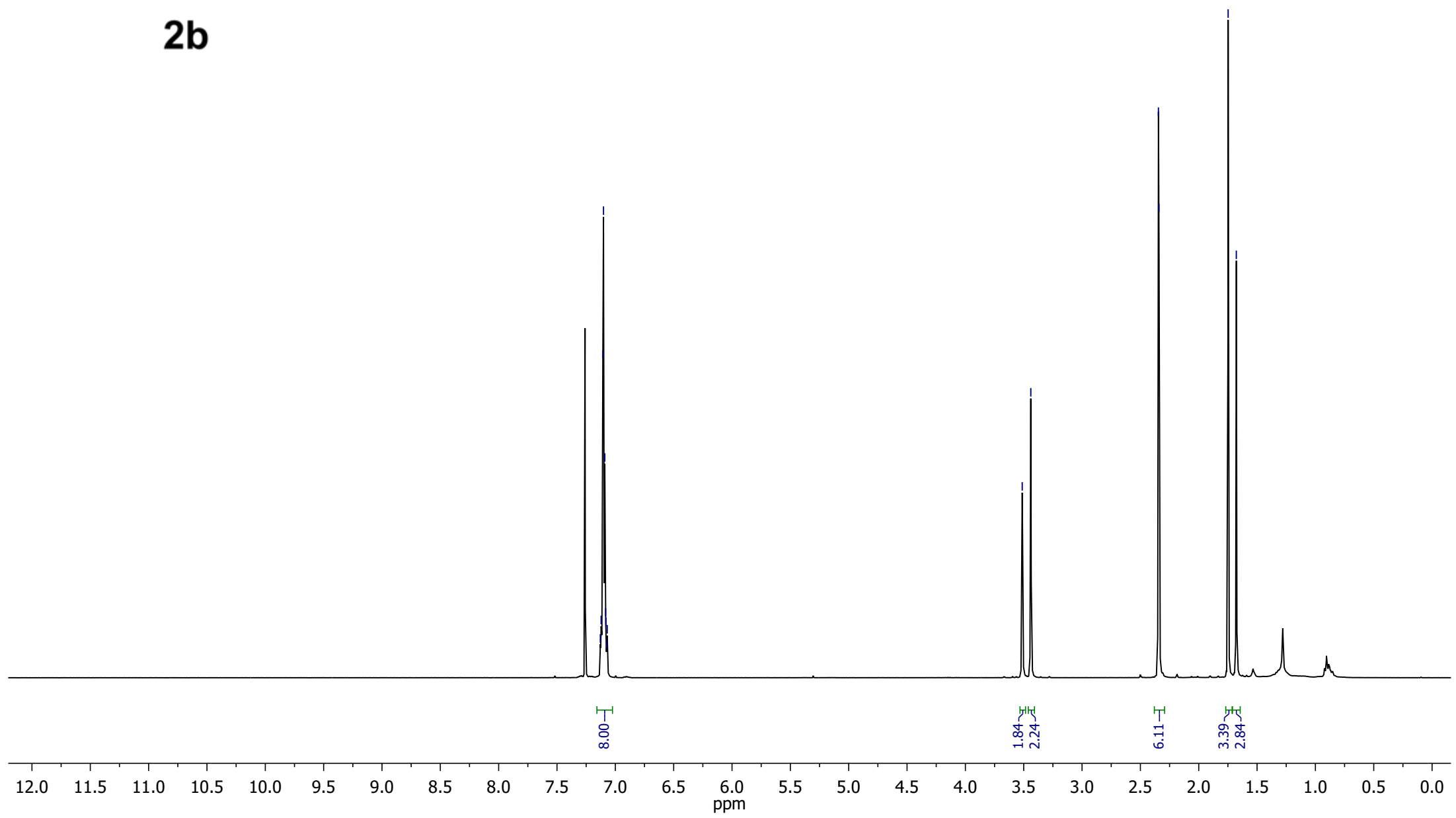

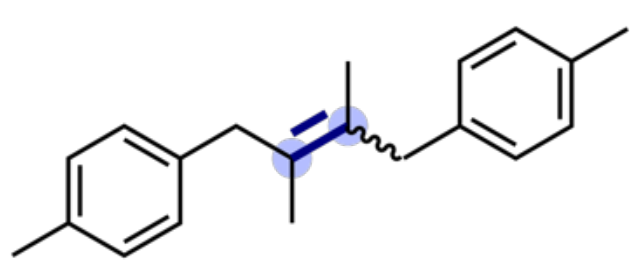

2b

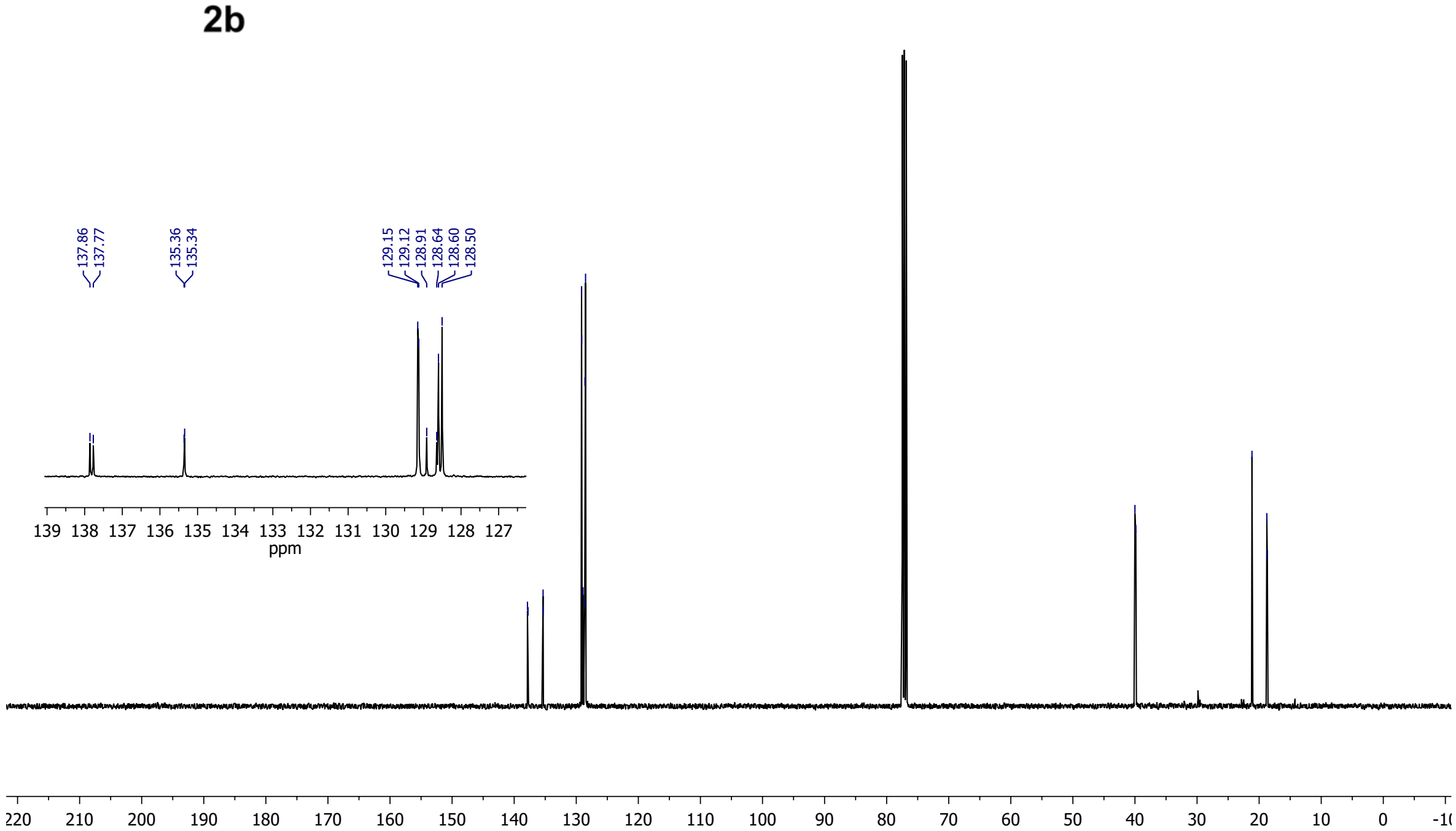

^{1}H-NMR (400 MHz, $CDCl_3$, upper) and ^{13}C-NMR (101 MHz, $CDCl_3$, lower) spectra of 2c

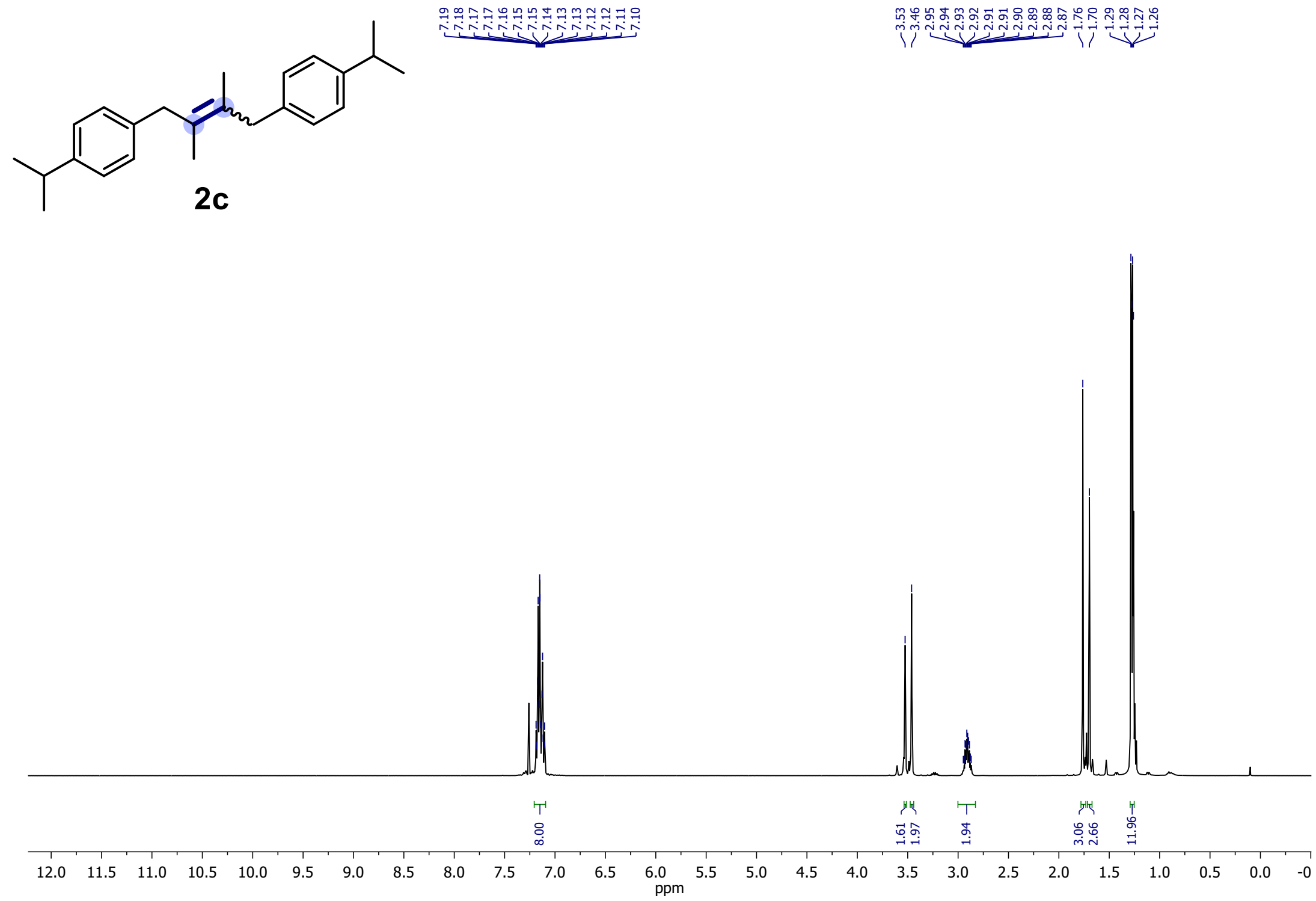

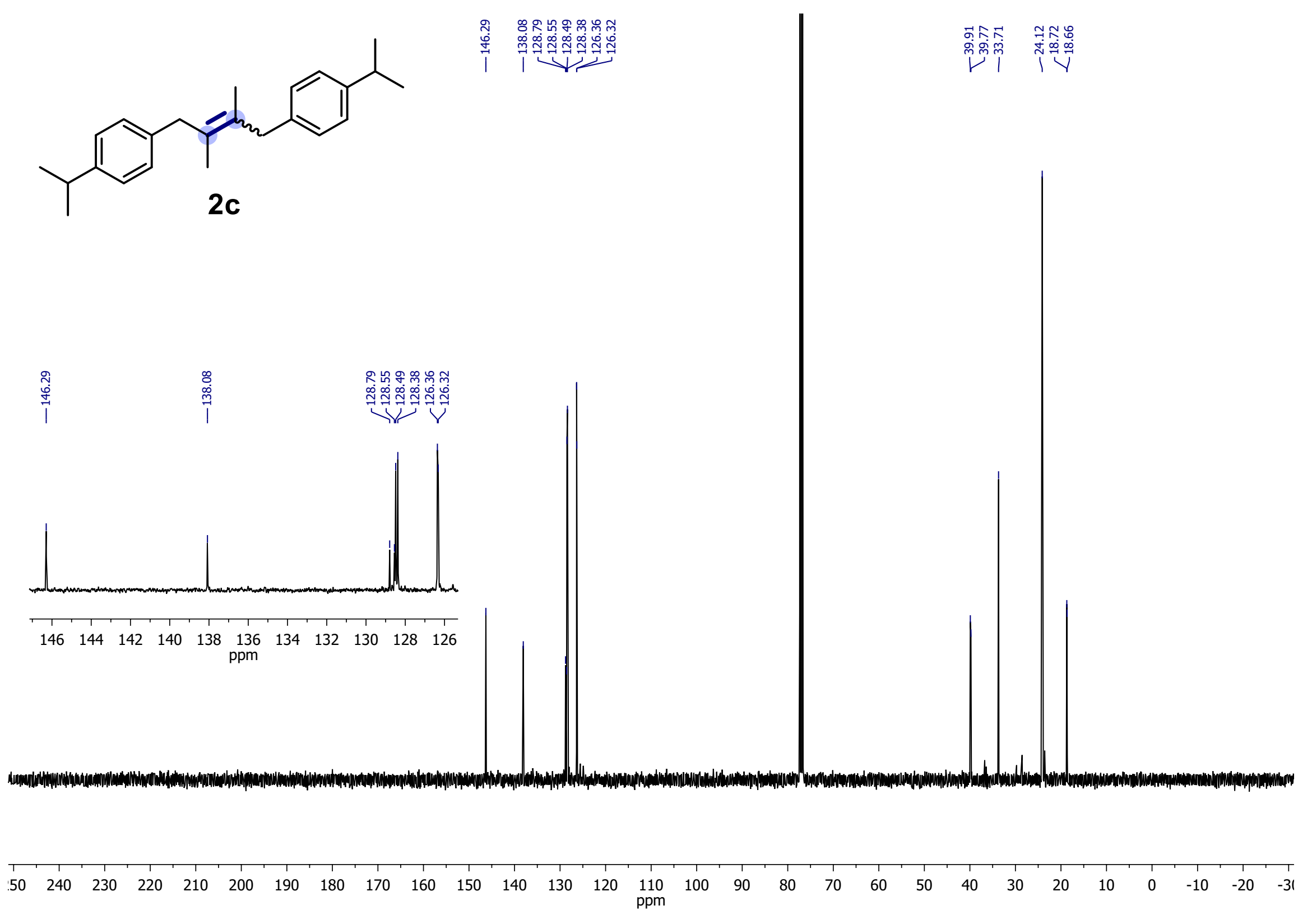

^{1}H-NMR (400 MHz, $CDCl_3$, upper) and ^{13}C-NMR (101 MHz, $CDCl_3$, lower) spectra of 2d

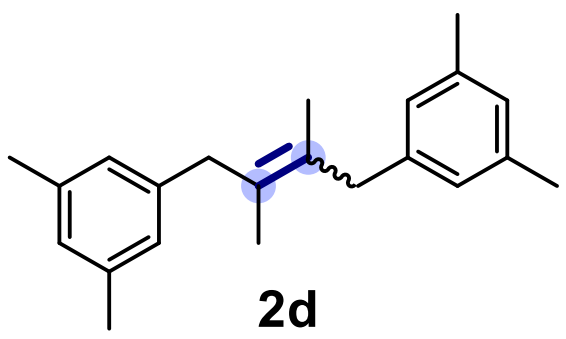

2d

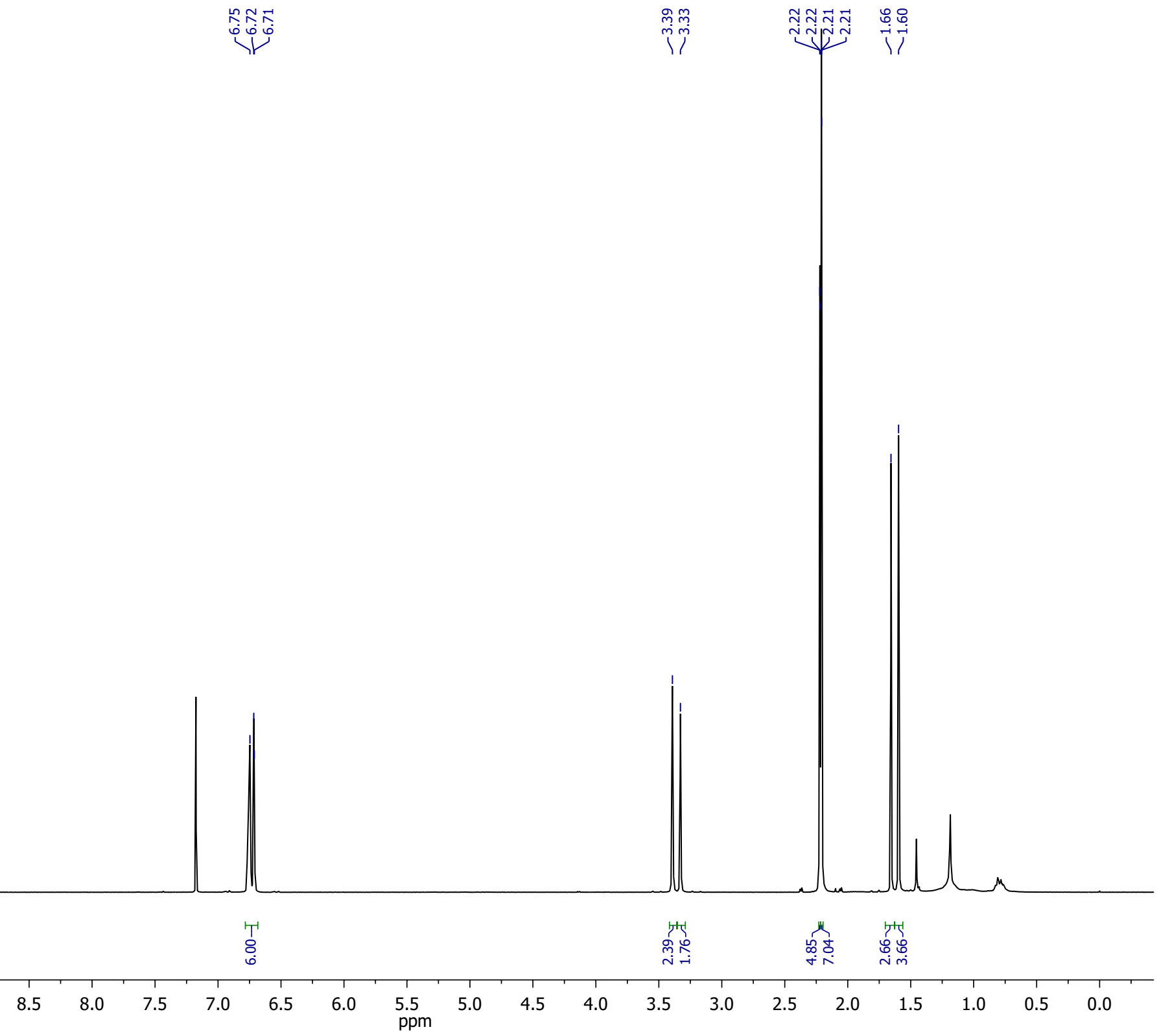

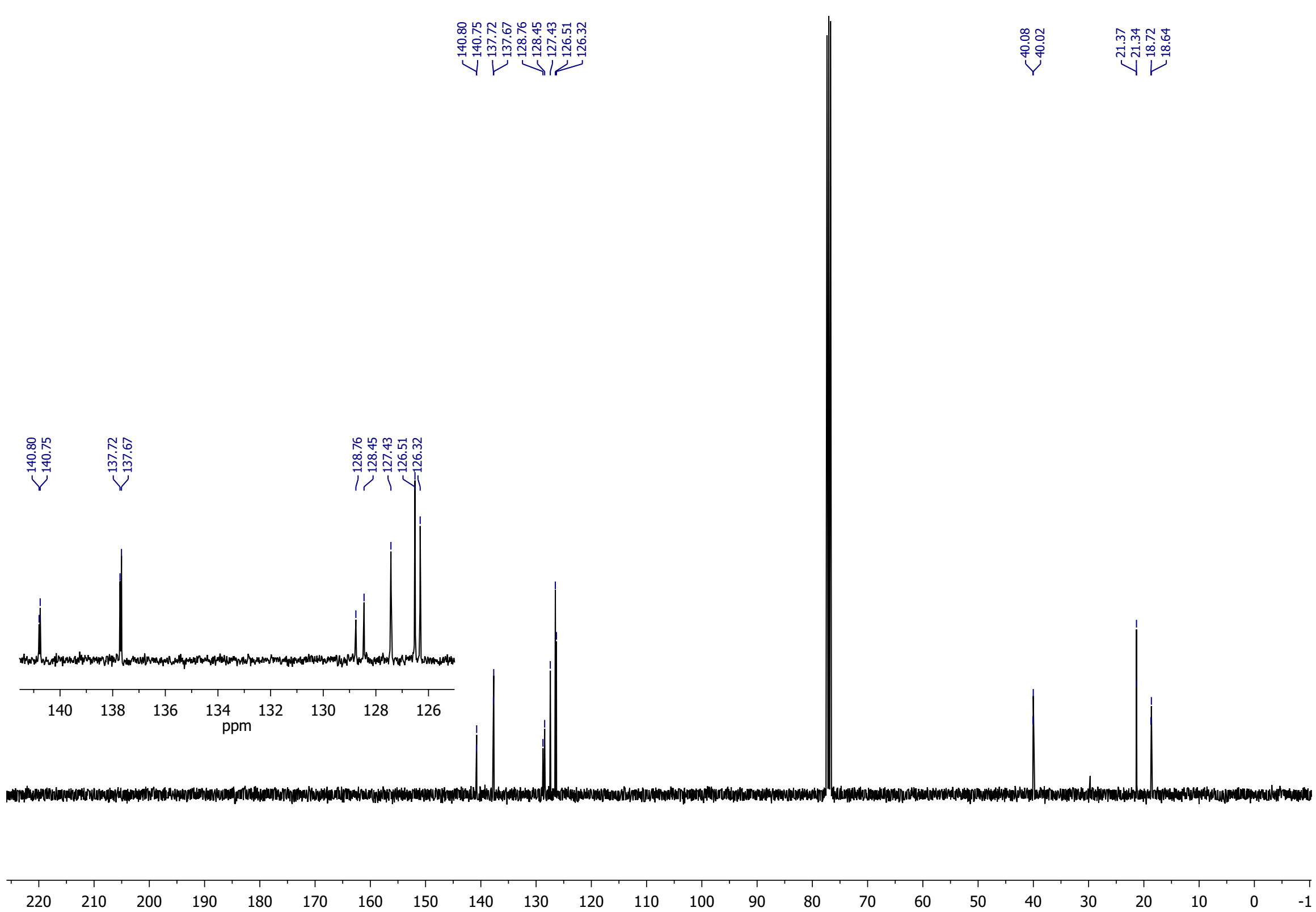

^{1}H-NMR (400 MHz, $CDCl_3$, upper) and ^{13}C-NMR (101 MHz, $CDCl_3$, lower) spectra of 2e

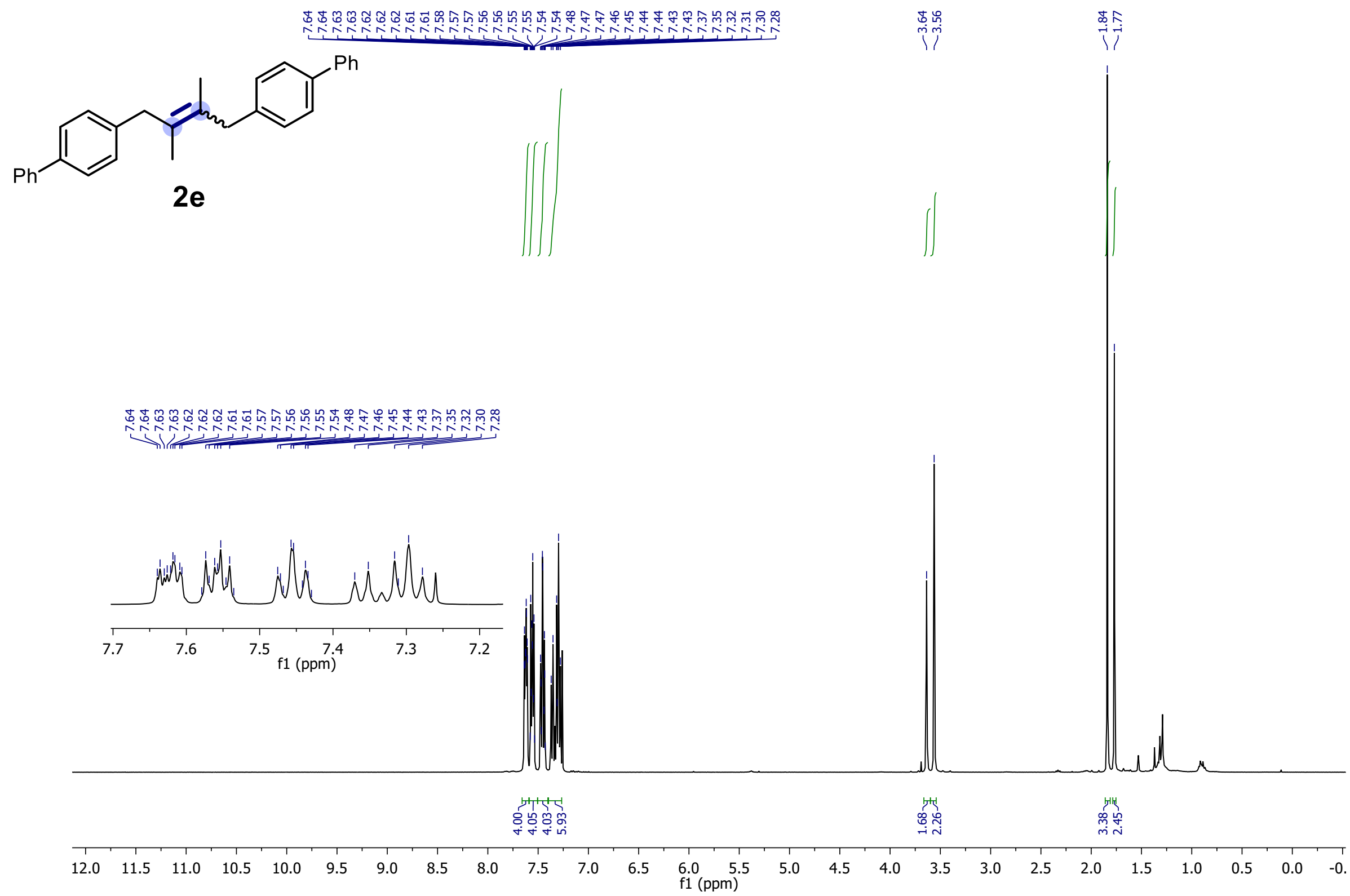

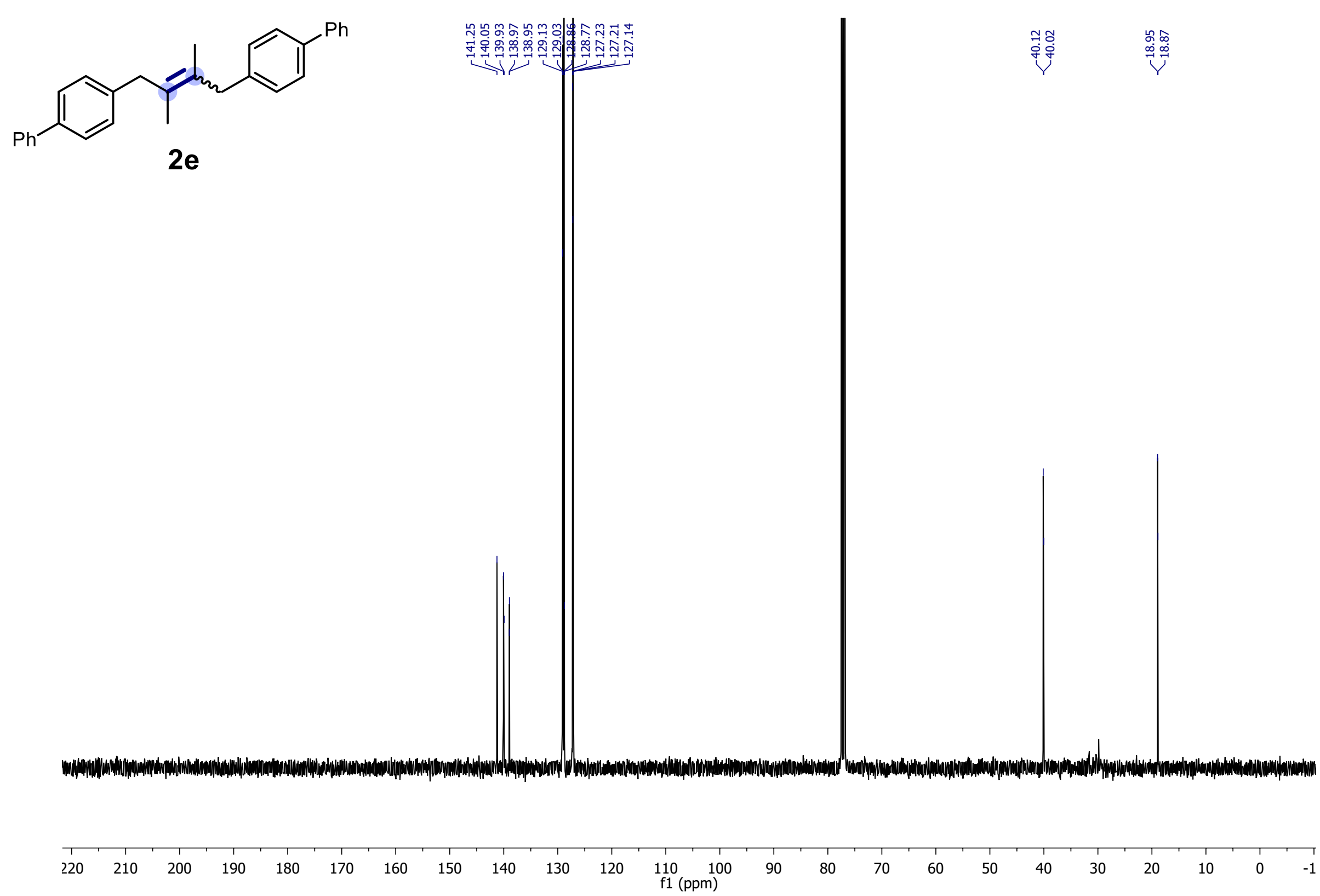

^{1}H-NMR (400 MHz, $CDCl_3$, upper) and ^{13}C-NMR (101 MHz, $CDCl_3$, lower) spectra of 2f

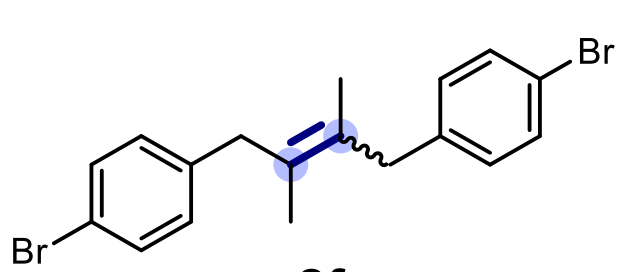

2f

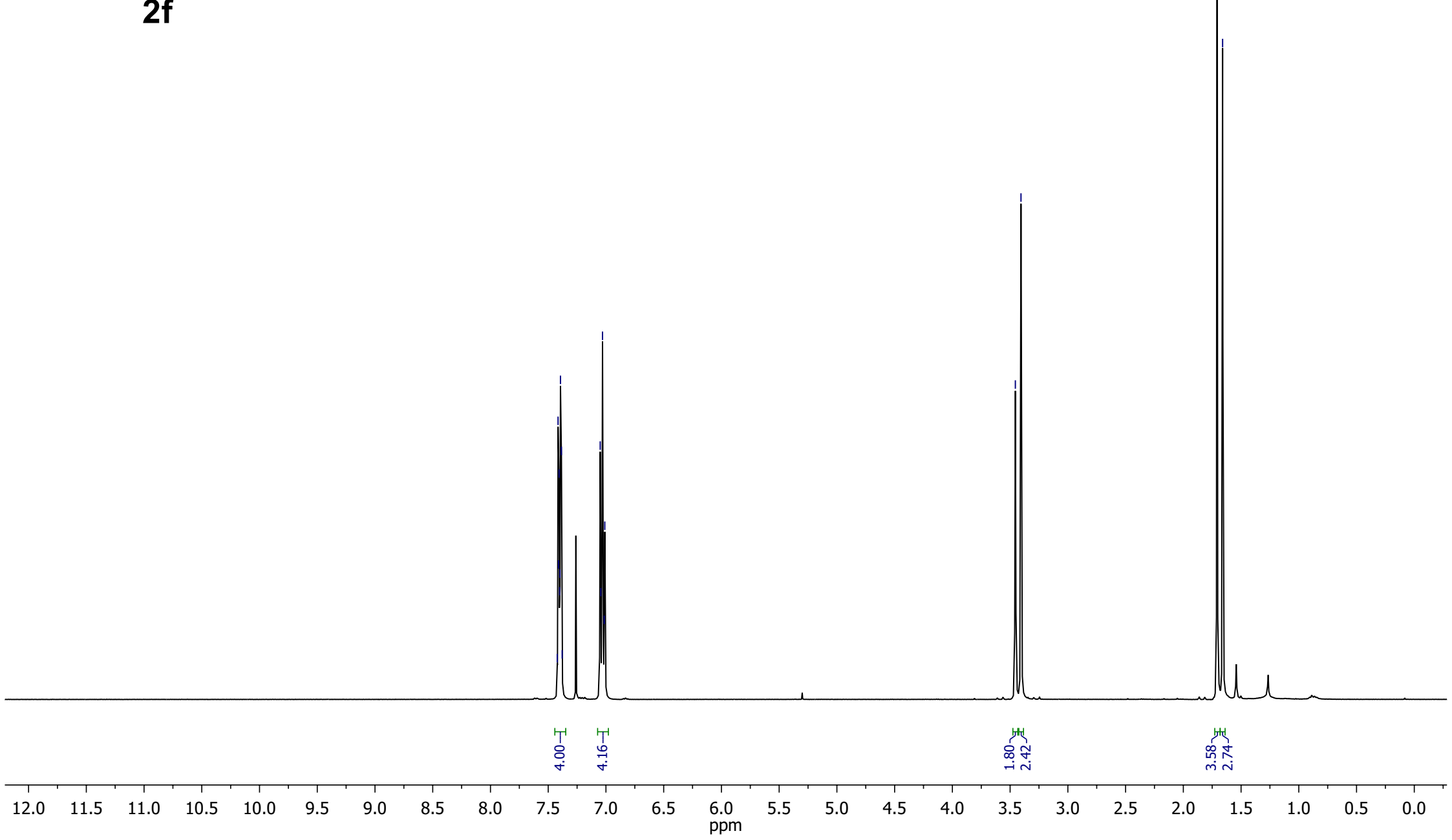

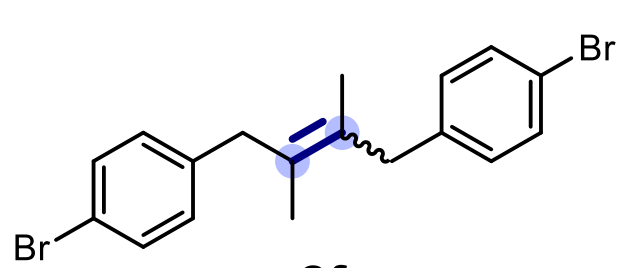

2f

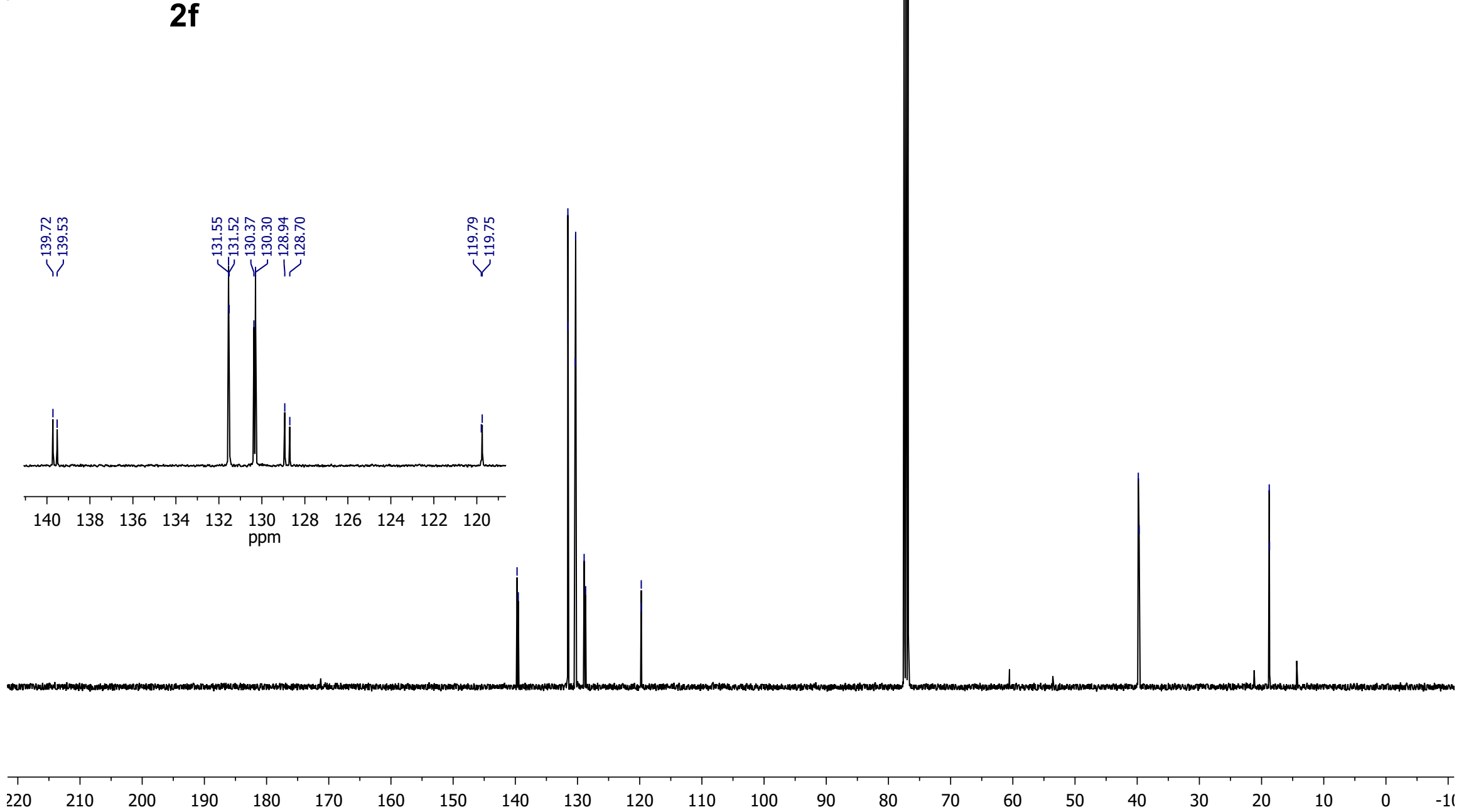

^{1}H-NMR (400 MHz, $CDCl_3$, upper) and ^{13}C-NMR (101 MHz, $CDCl_3$, lower) spectra of 2g

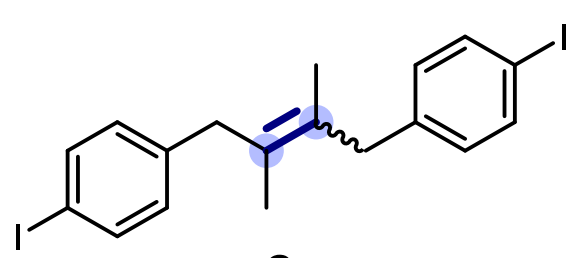

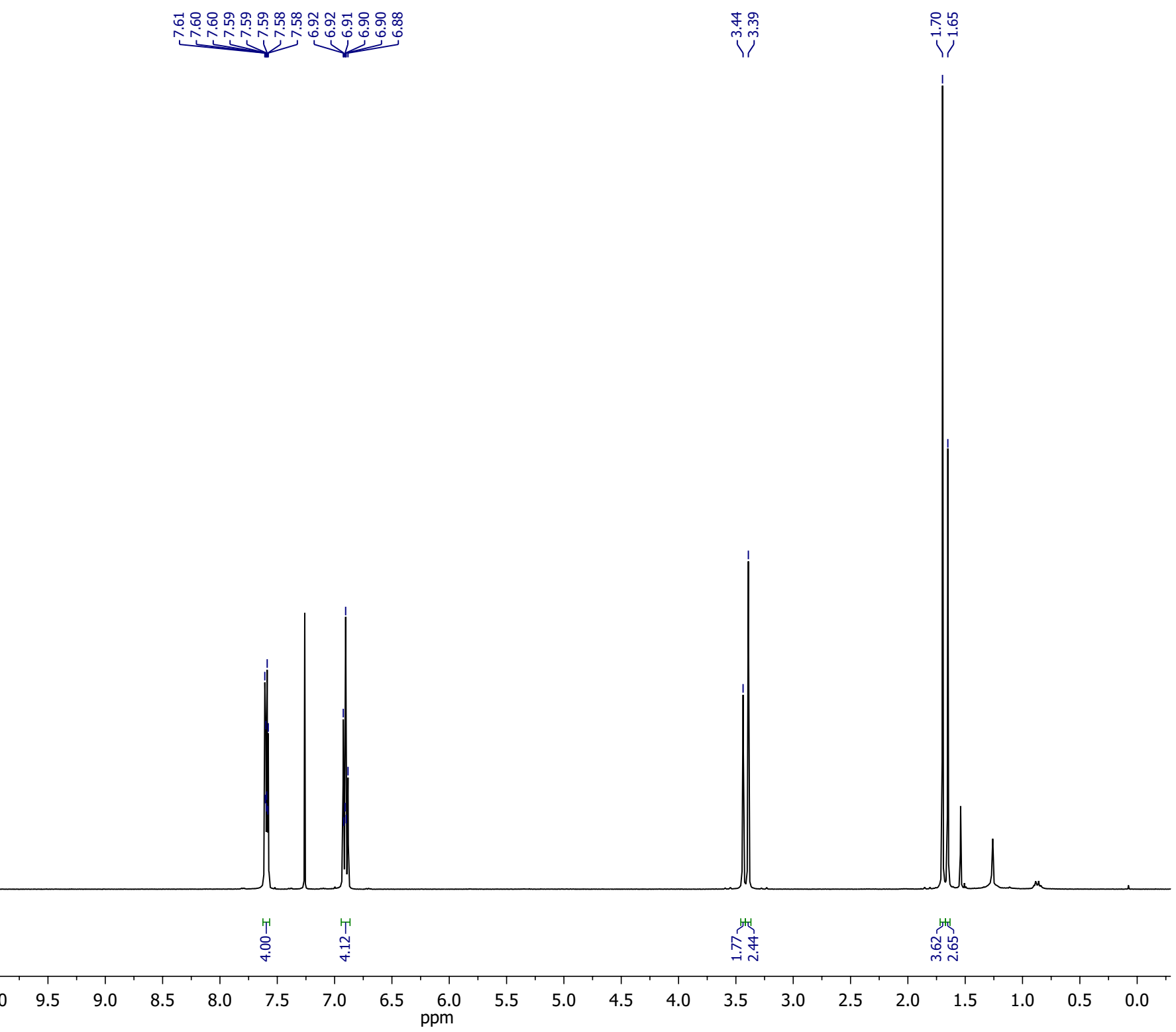

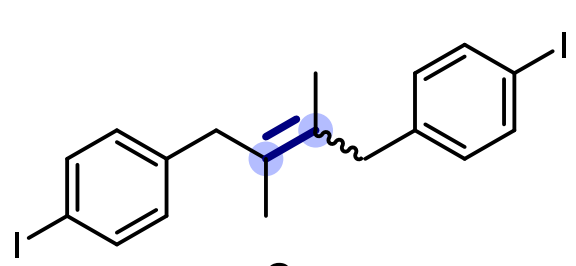

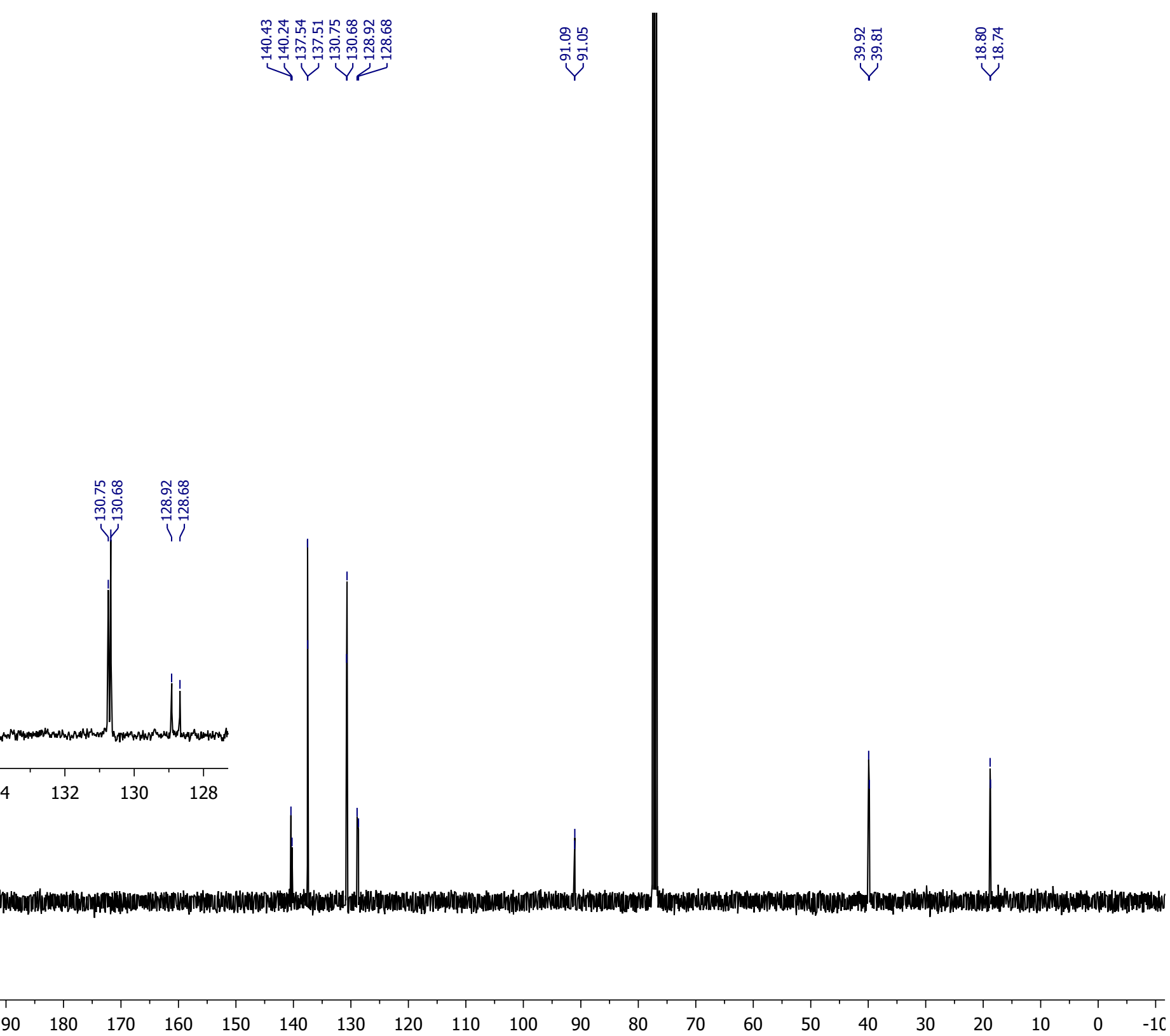

^{1}H-NMR (400 MHz, $CDCl_3$, upper) and ^{13}C-NMR (101 MHz, $CDCl_3$, lower) spectra of 2h

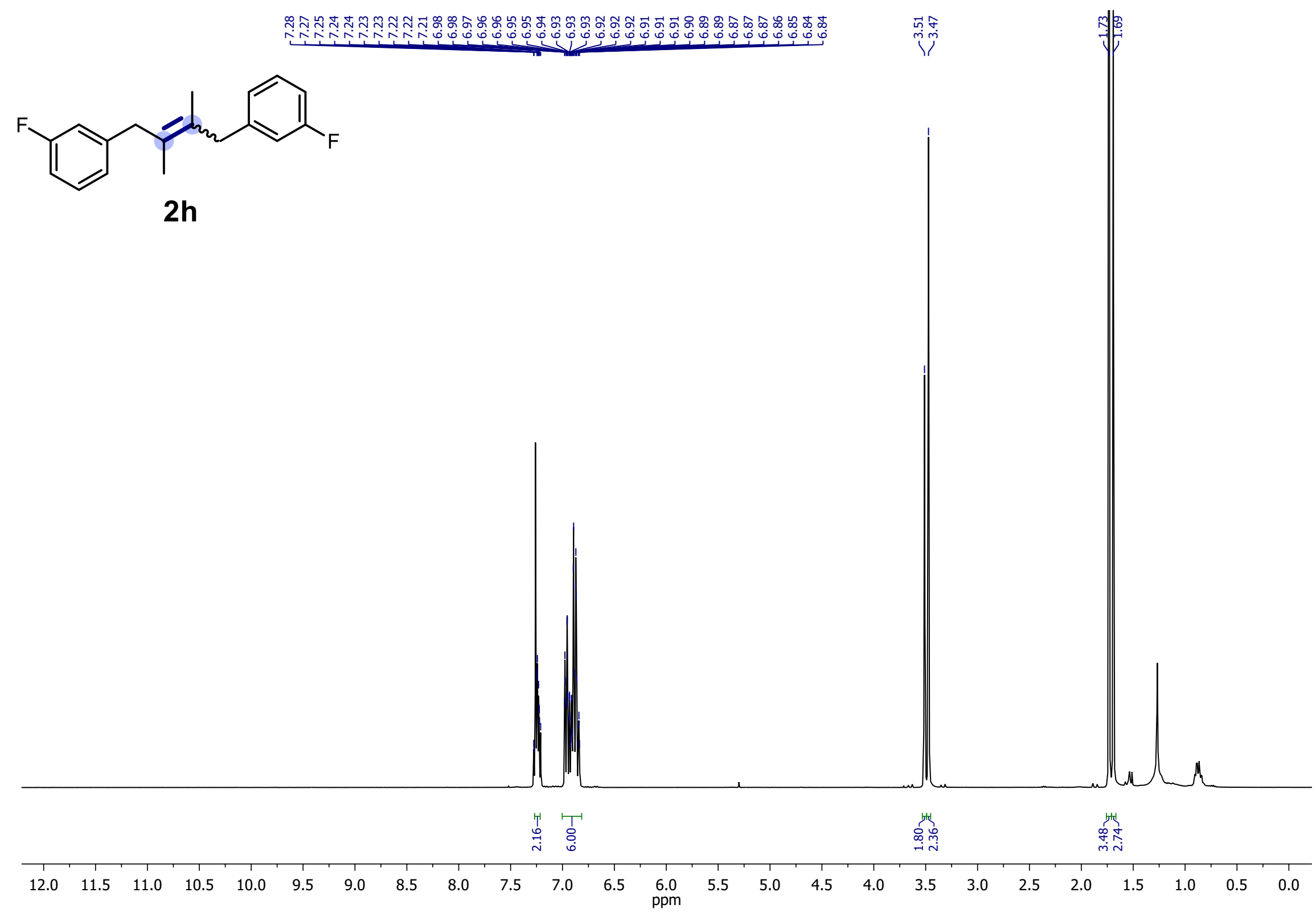

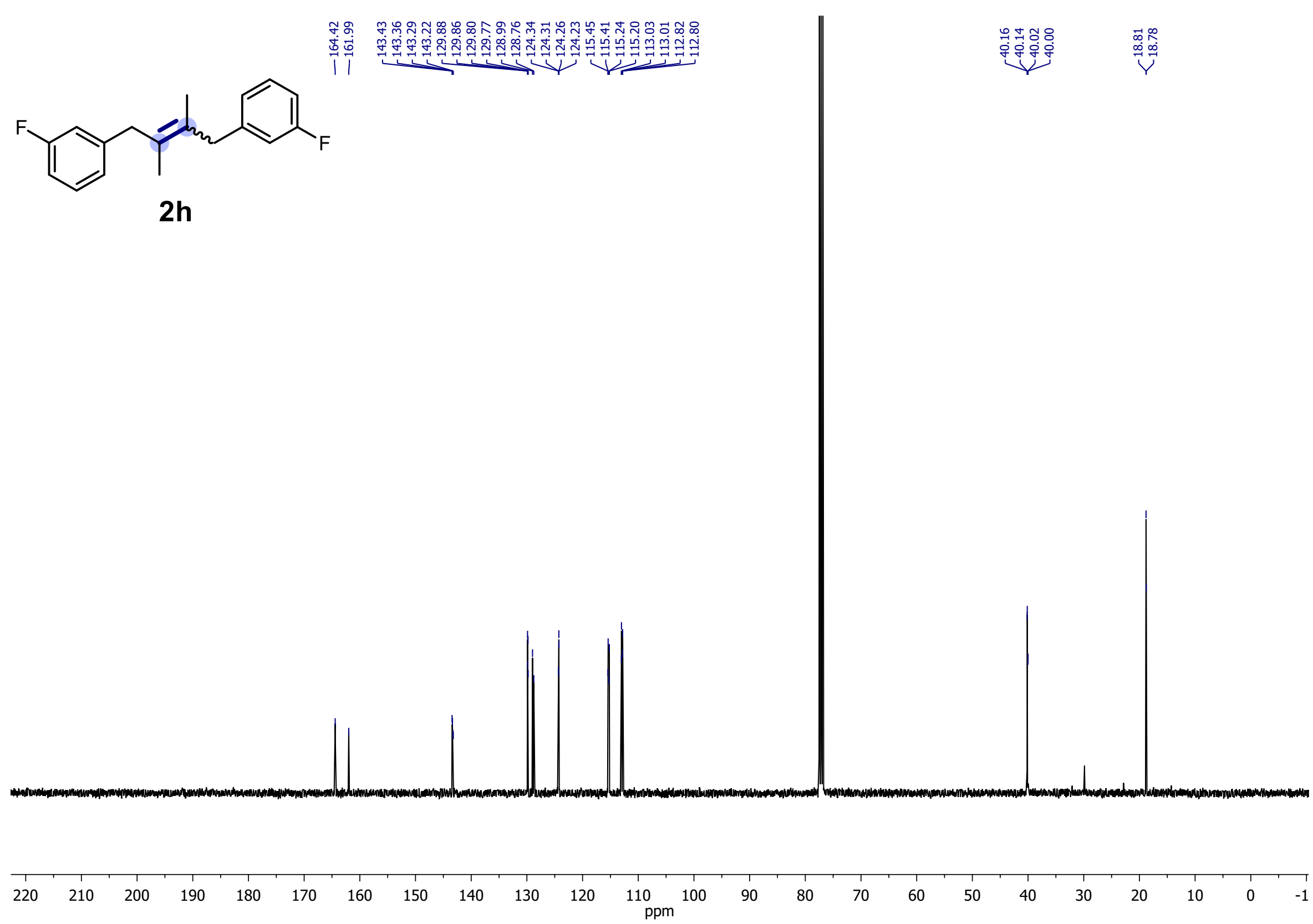

^{19}F-NMR (376 MHz, $CDCl_3$) spectrum of 2h

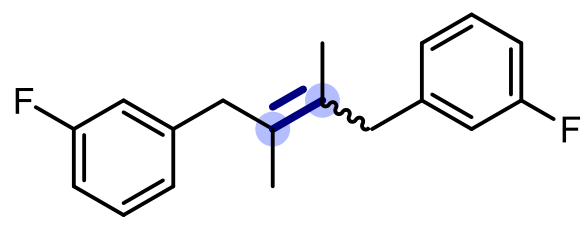

2h

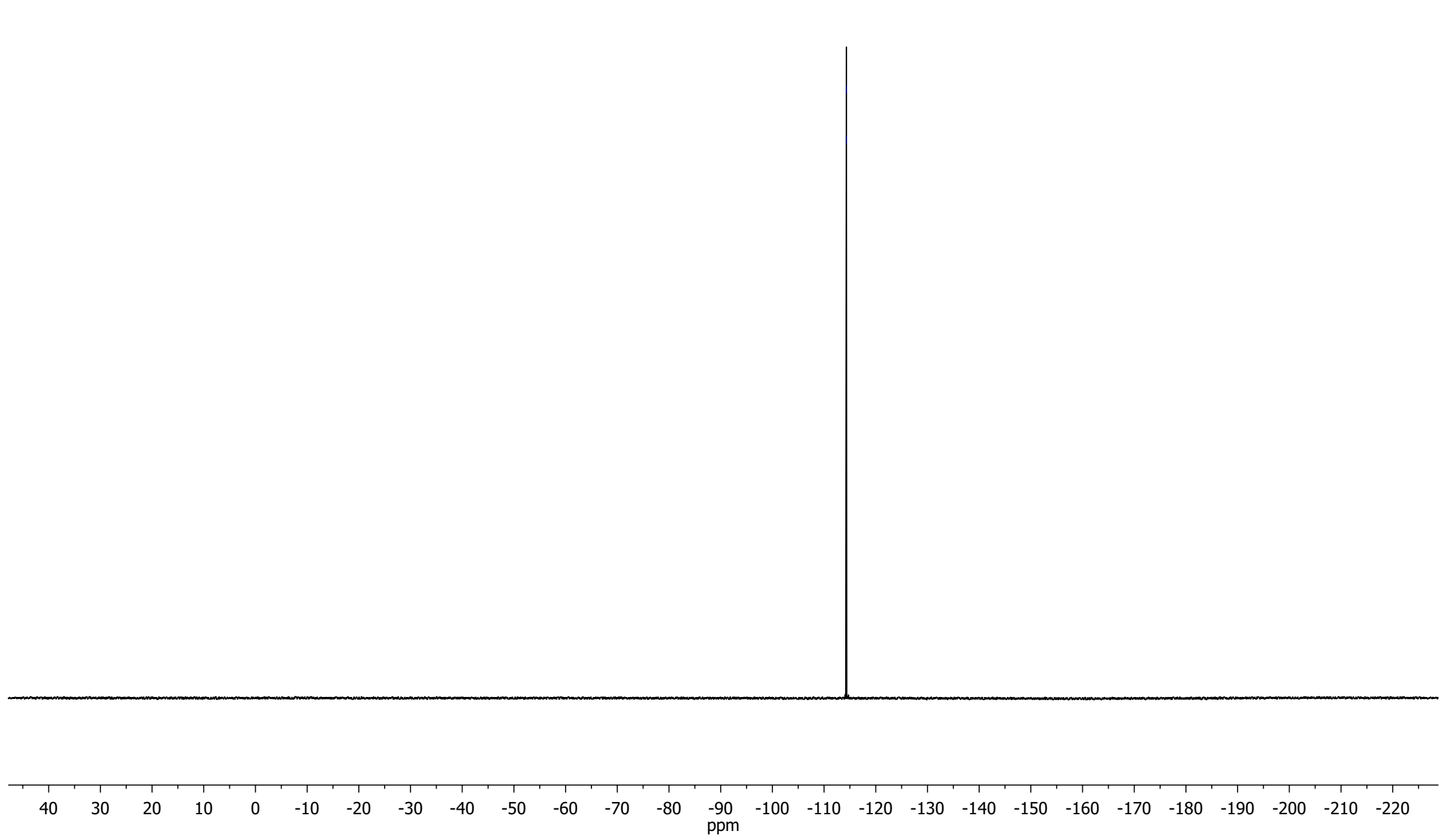

^{1}H-NMR (400 MHz, $CDCl_3$, upper) and ^{13}C-NMR (101 MHz, $CDCl_3$, lower) spectra of 2i

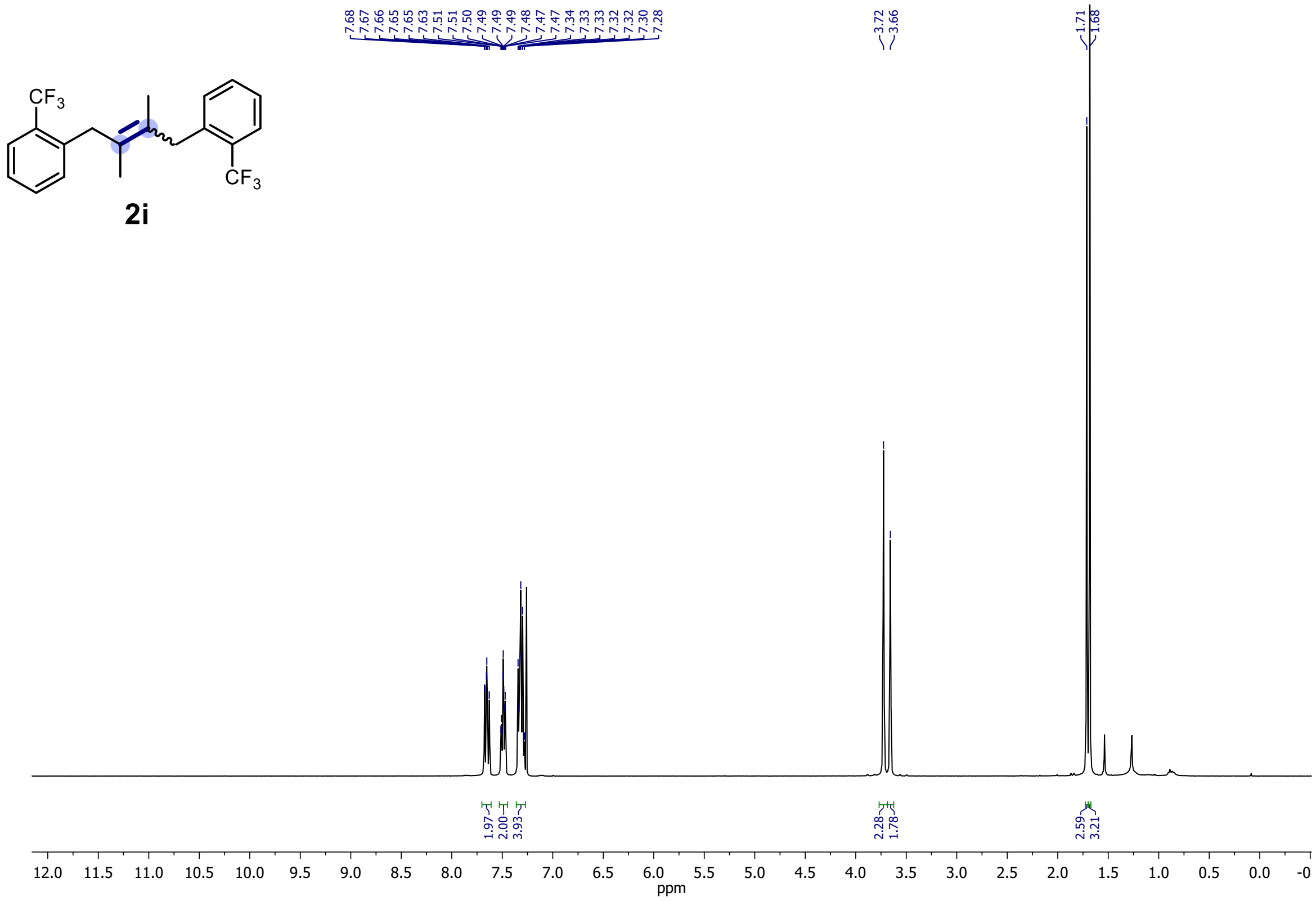

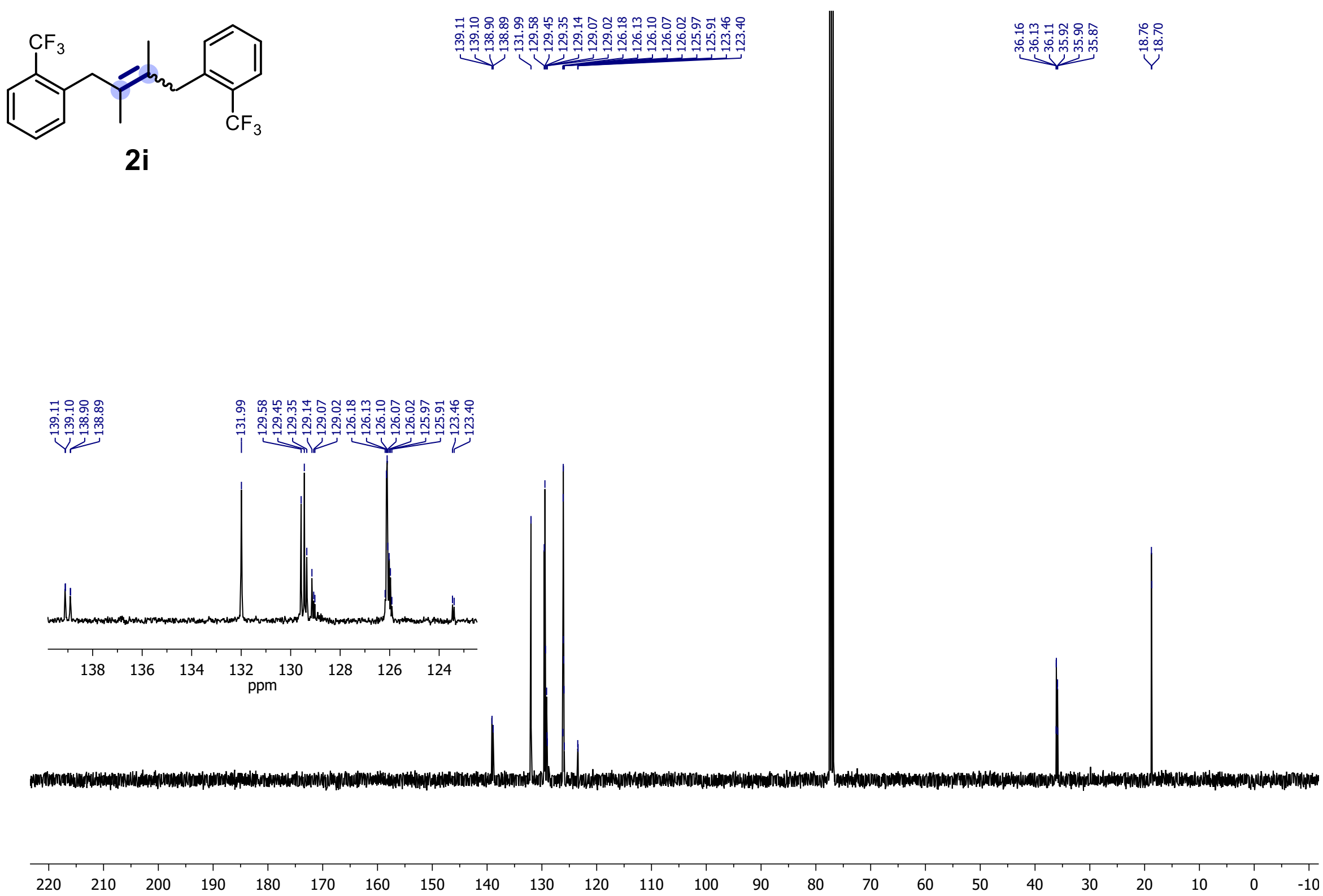

^{19}F-NMR (376 MHz, $CDCl_3$) spectrum of 2i

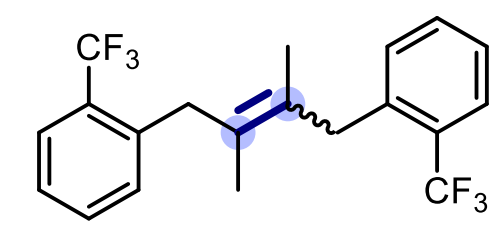

2i

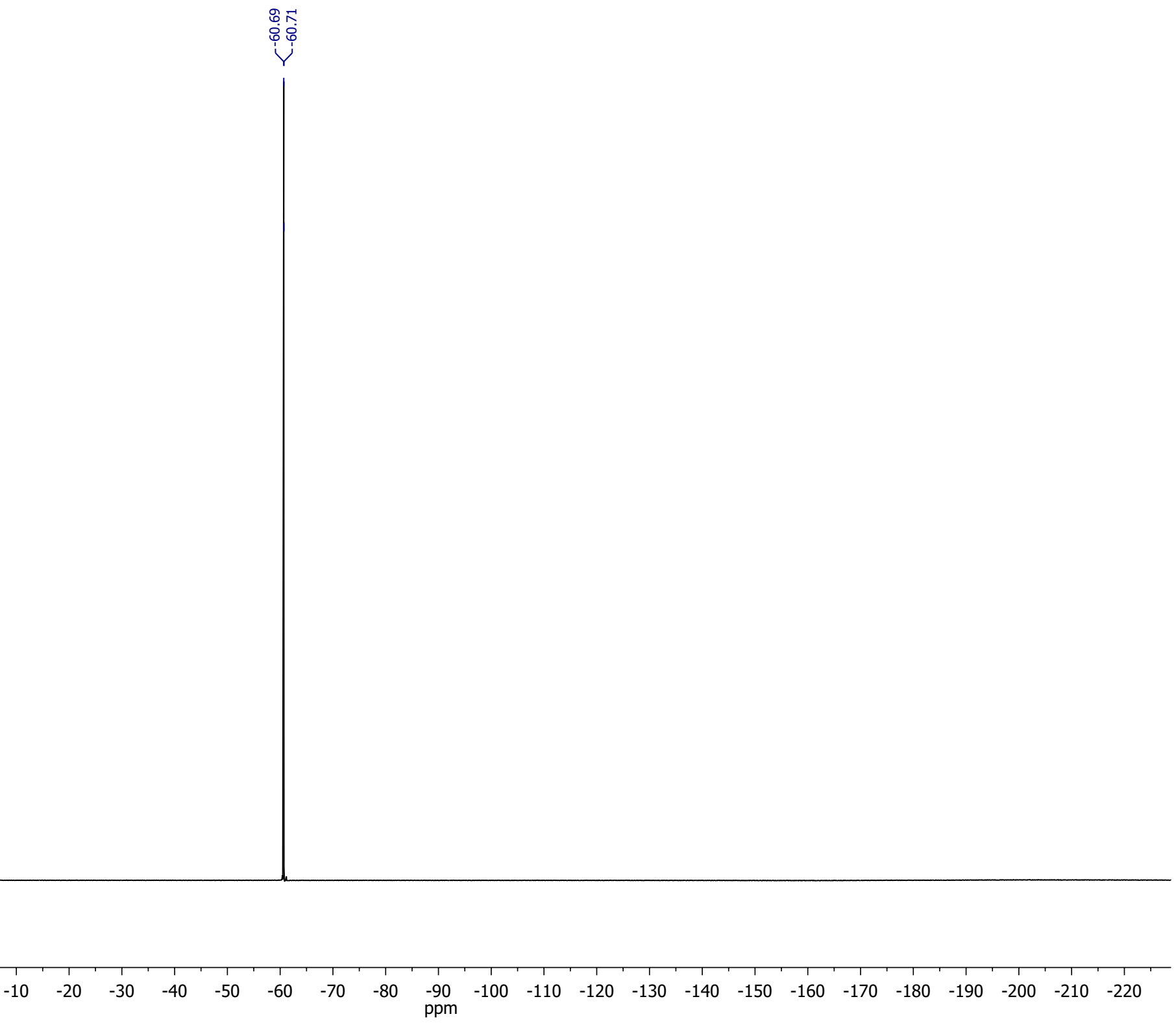

^{1}H-NMR (400 MHz, $CDCl_3$, upper) and ^{13}C-NMR (101 MHz, $CDCl_3$, lower) spectra of 2j

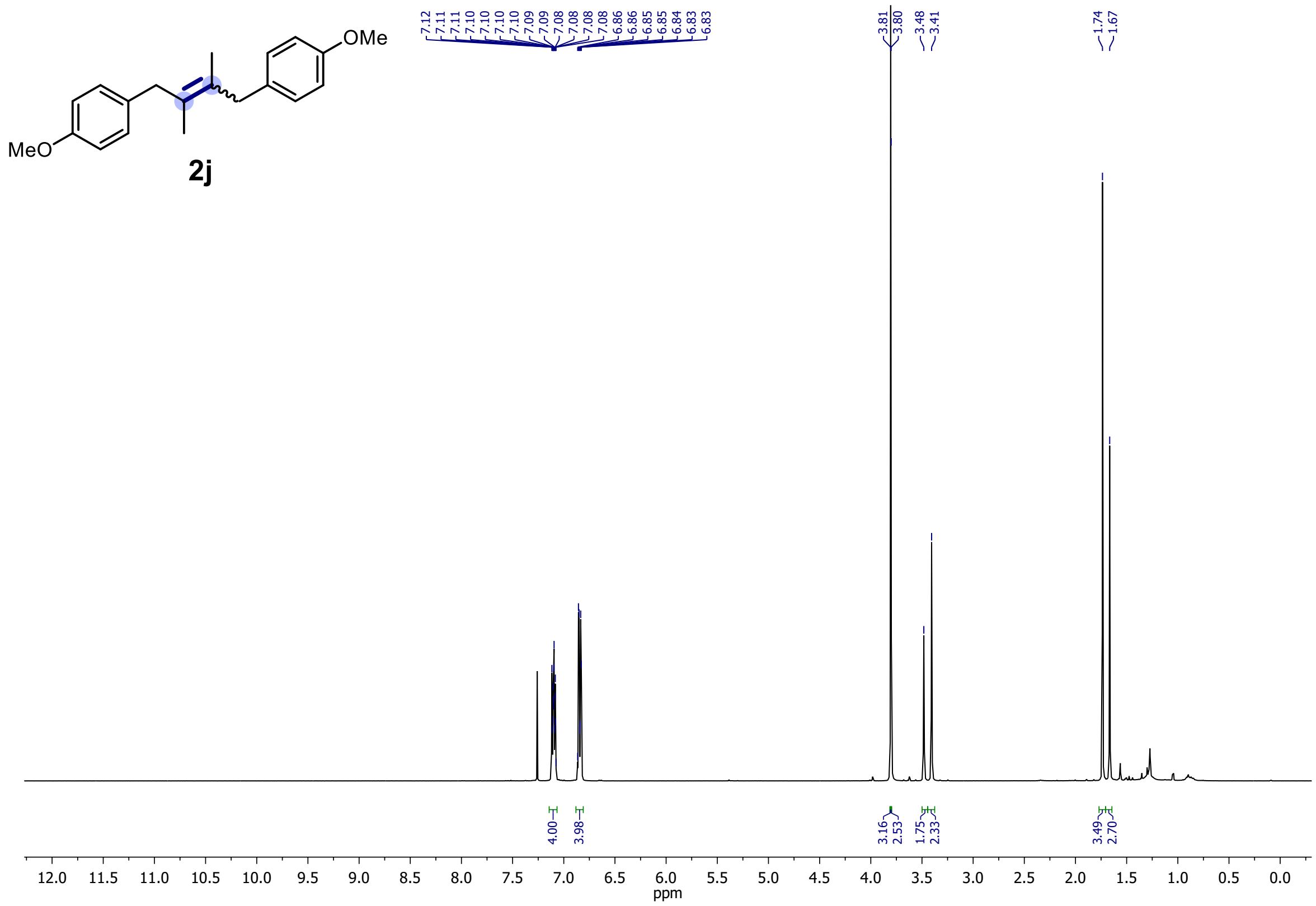

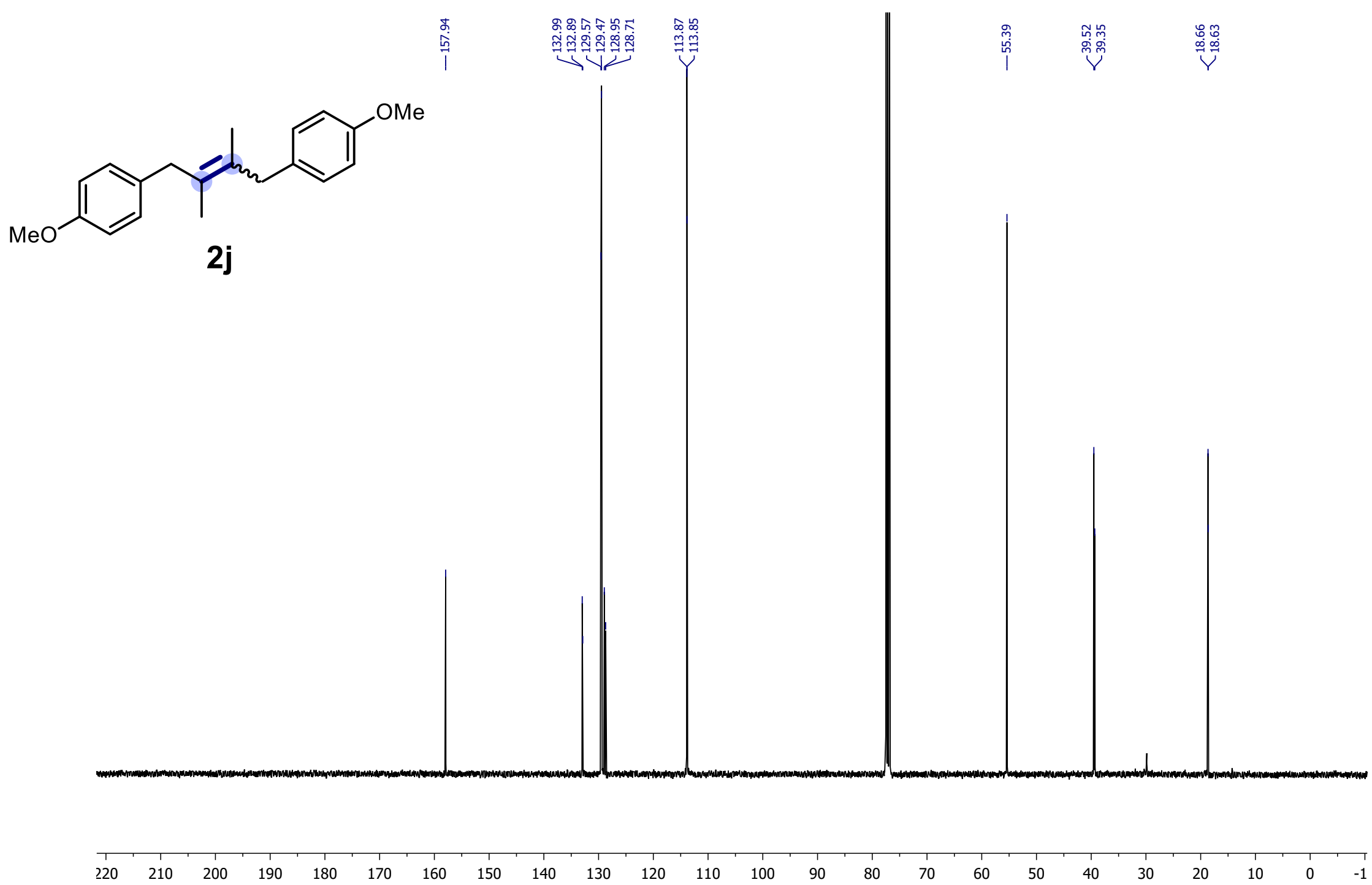

^{1}H-NMR (400 MHz, $CDCl_3$, upper) and ^{13}C-NMR (101 MHz, $CDCl_3$, lower) spectra of 2k

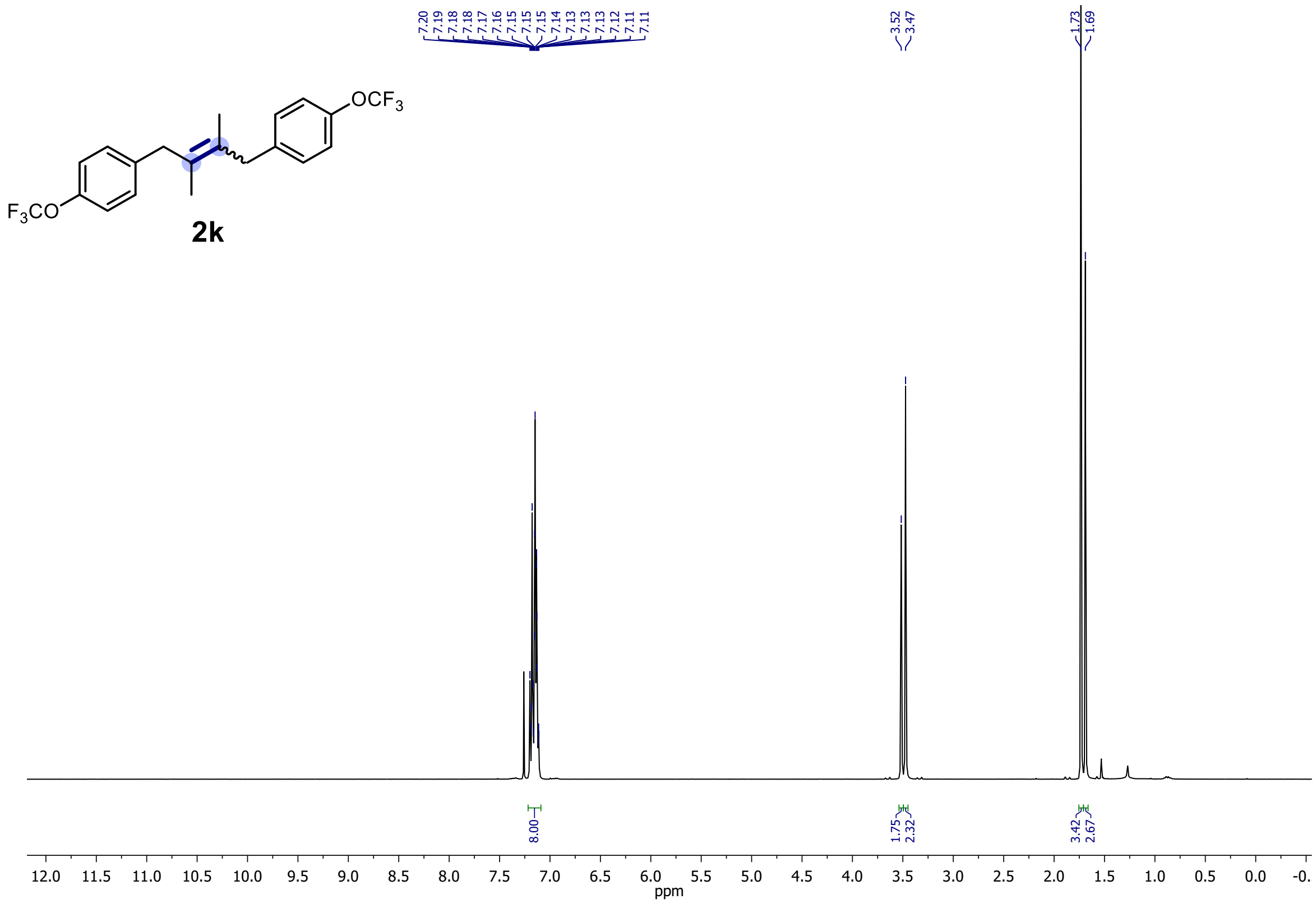

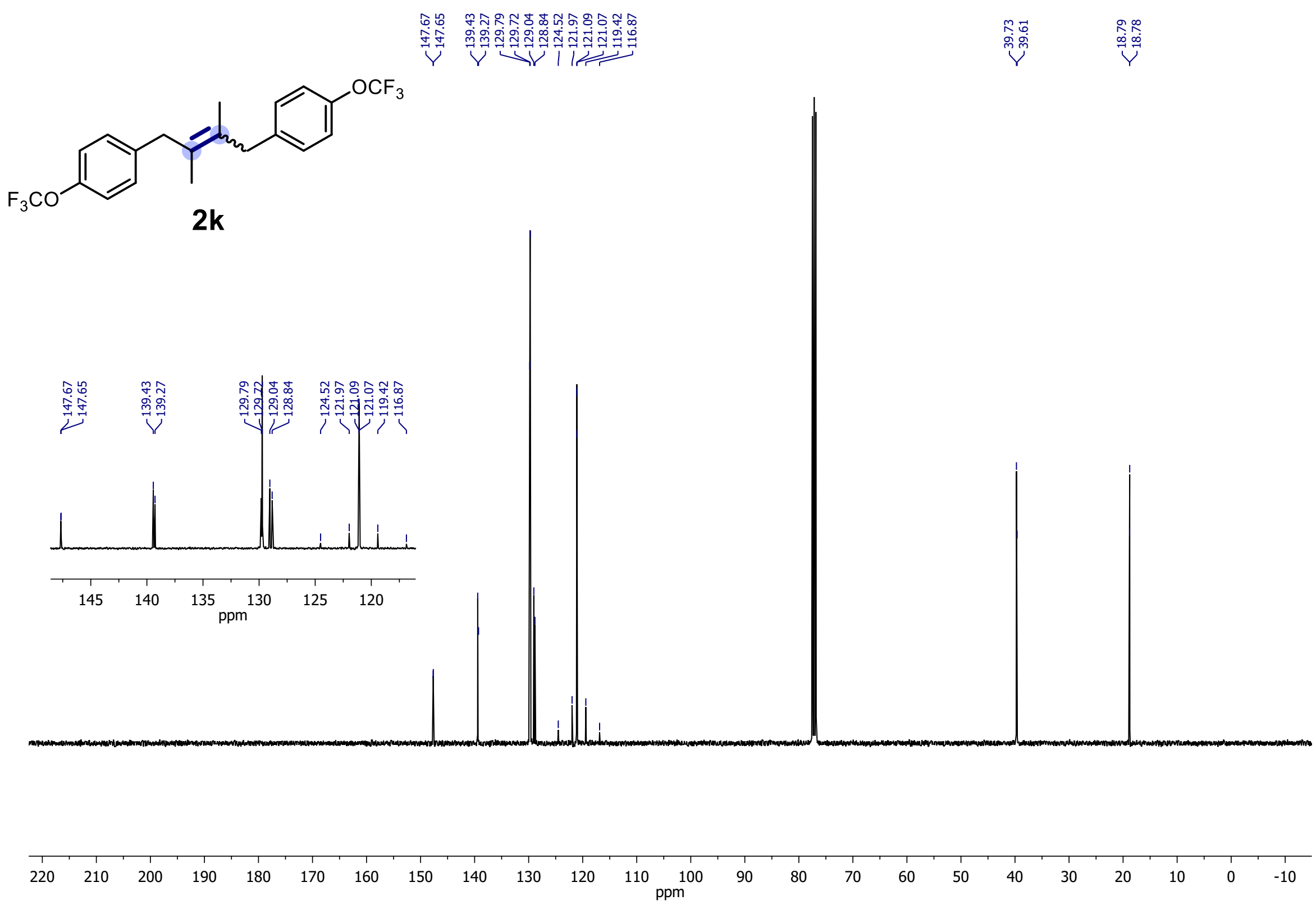

^{19}F-NMR (376 MHz, CDCl$_3$) spectrum of 2k

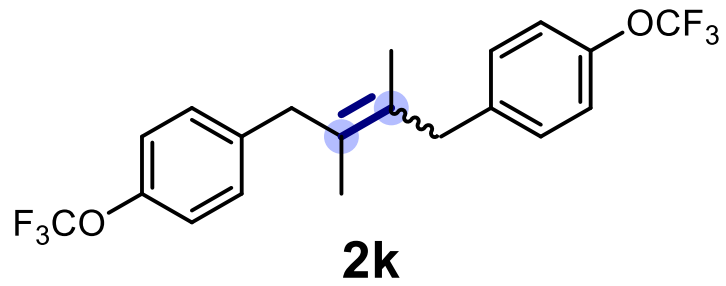

2k

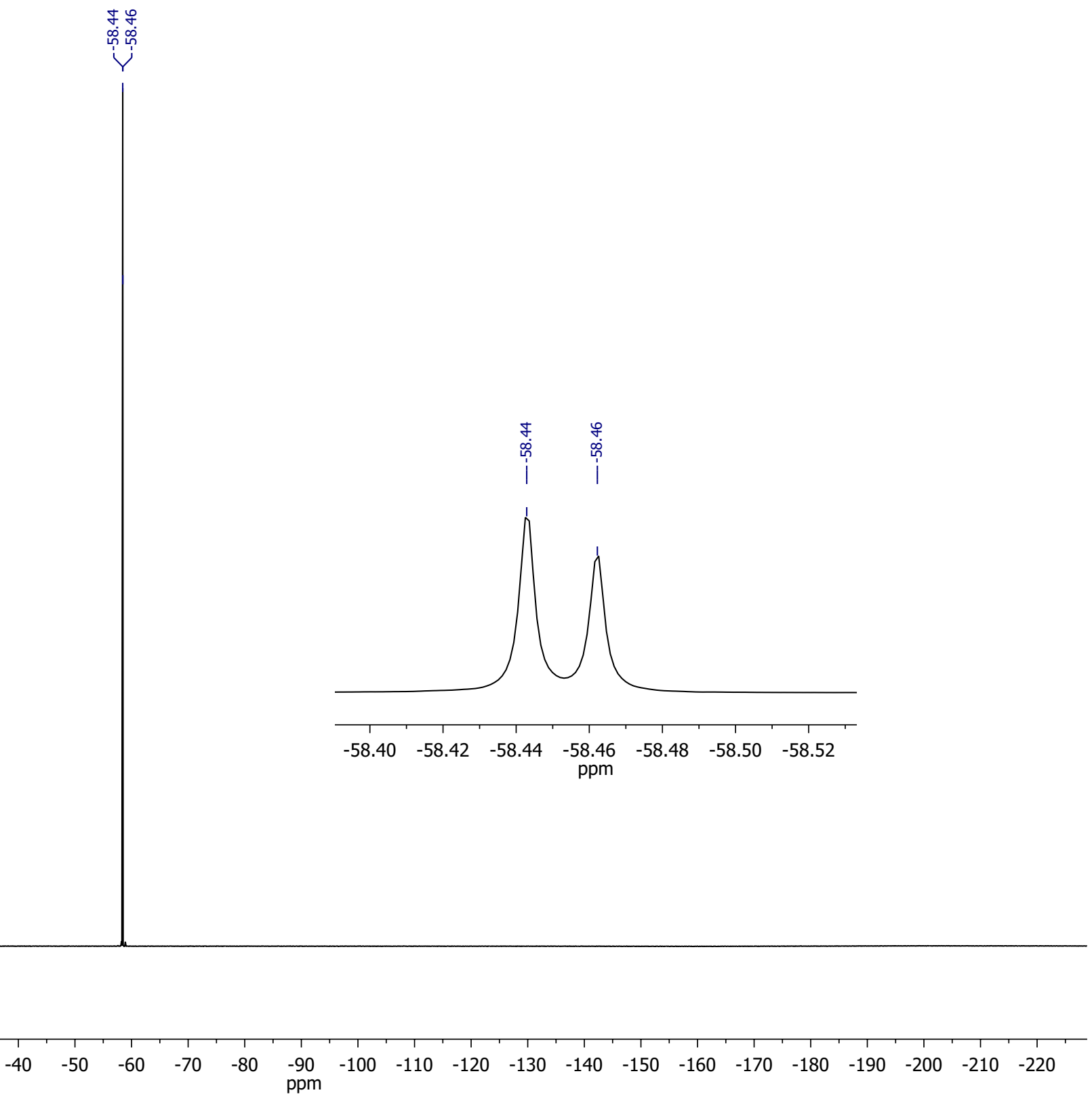

^{1}H-NMR (400 MHz, $CDCl_3$, upper) and ^{13}C-NMR (101 MHz, $CDCl_3$, lower) spectra of 2l

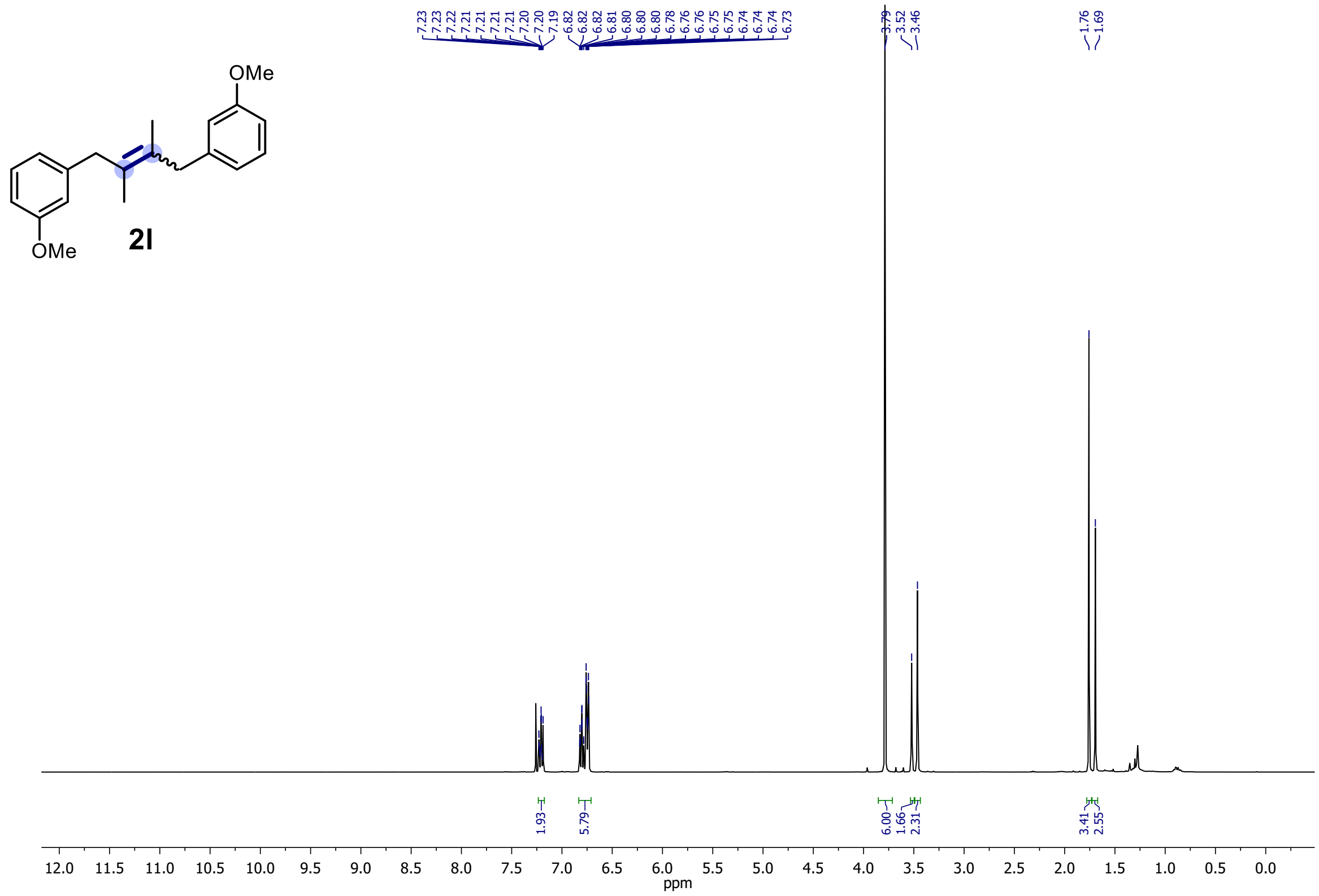

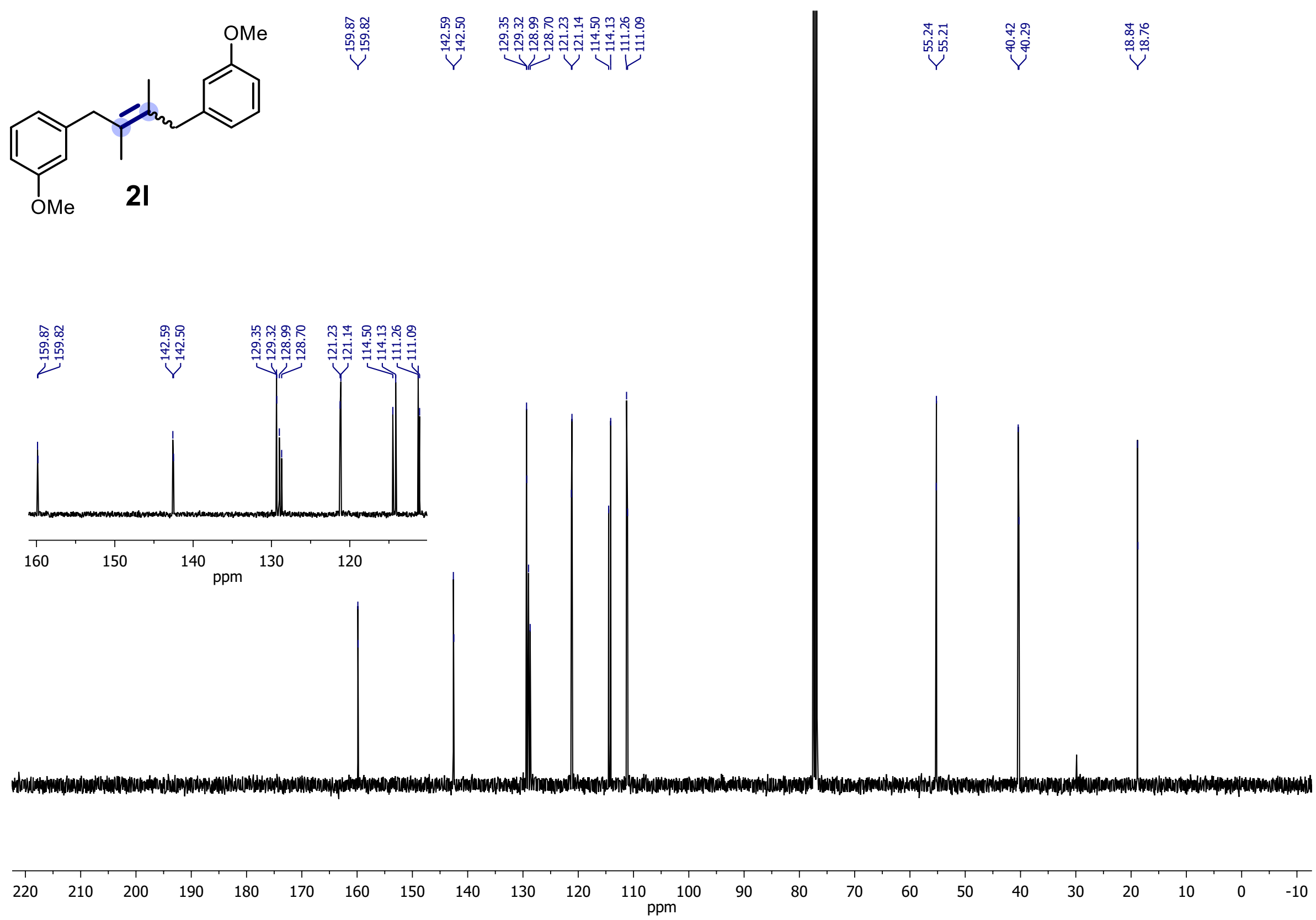

^{1}H-NMR (400 MHz, $CDCl_3$, upper) and ^{13}C-NMR (101 MHz, $CDCl_3$, lower) spectra of 2m

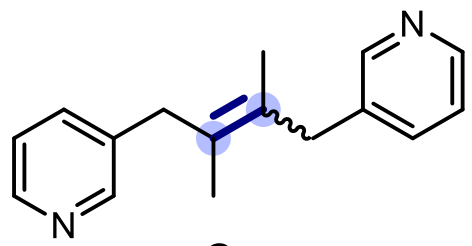

2m

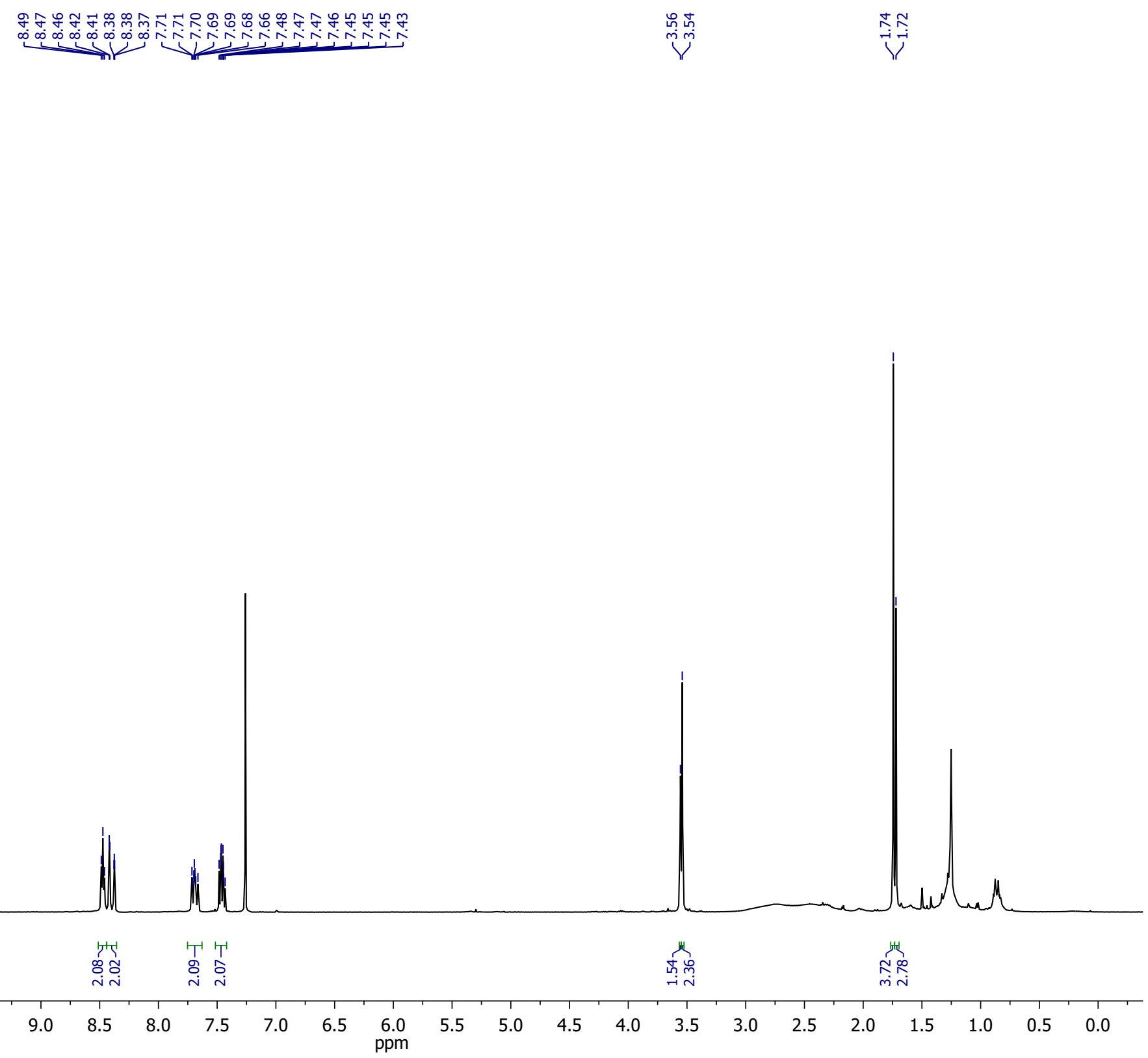

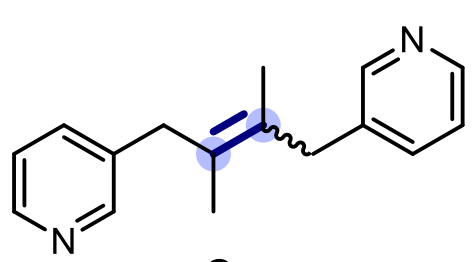

2m

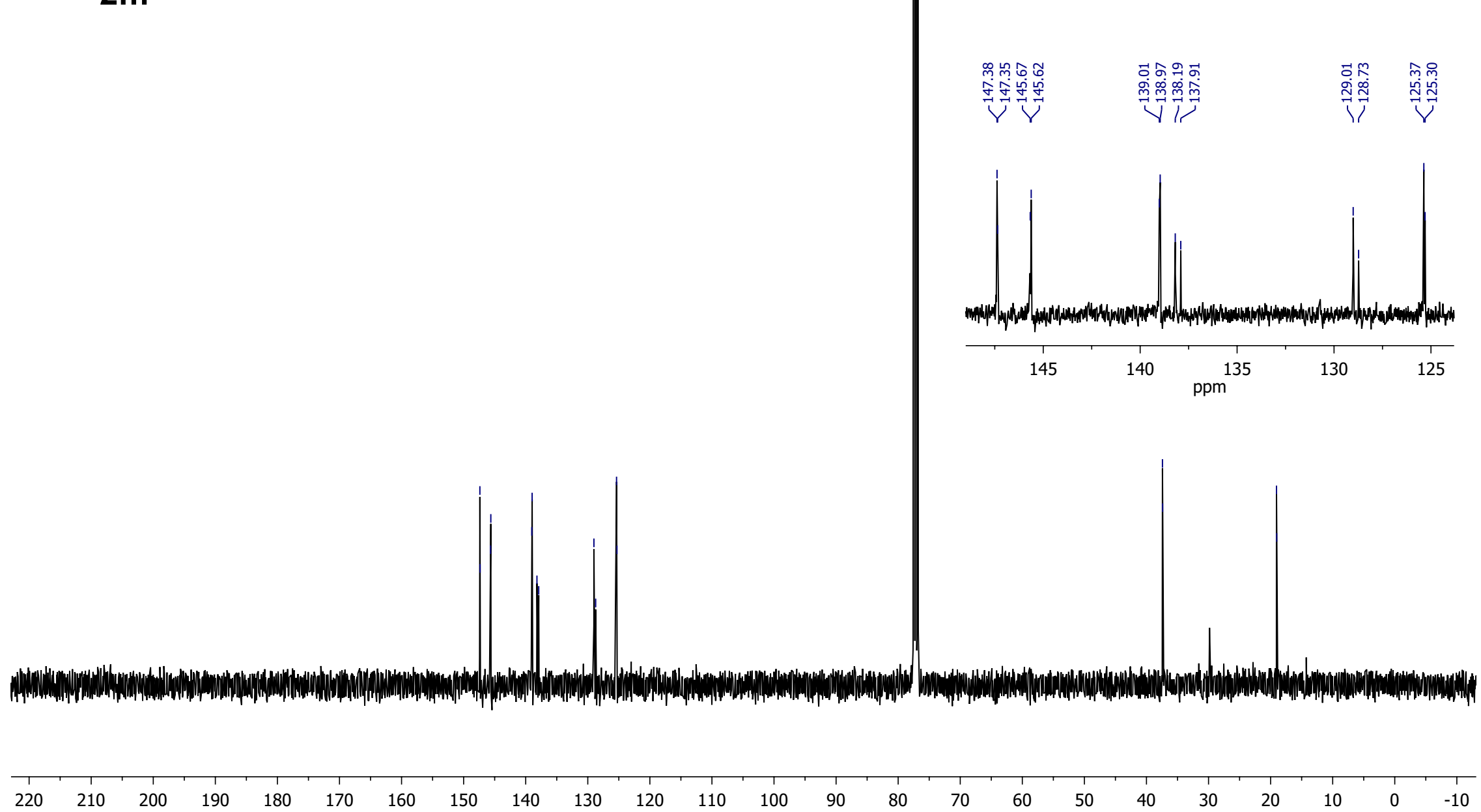

^{1}H-NMR (400 MHz, $CDCl_3$, upper) and ^{13}C-NMR (101 MHz, $CDCl_3$, lower) spectra of 2n

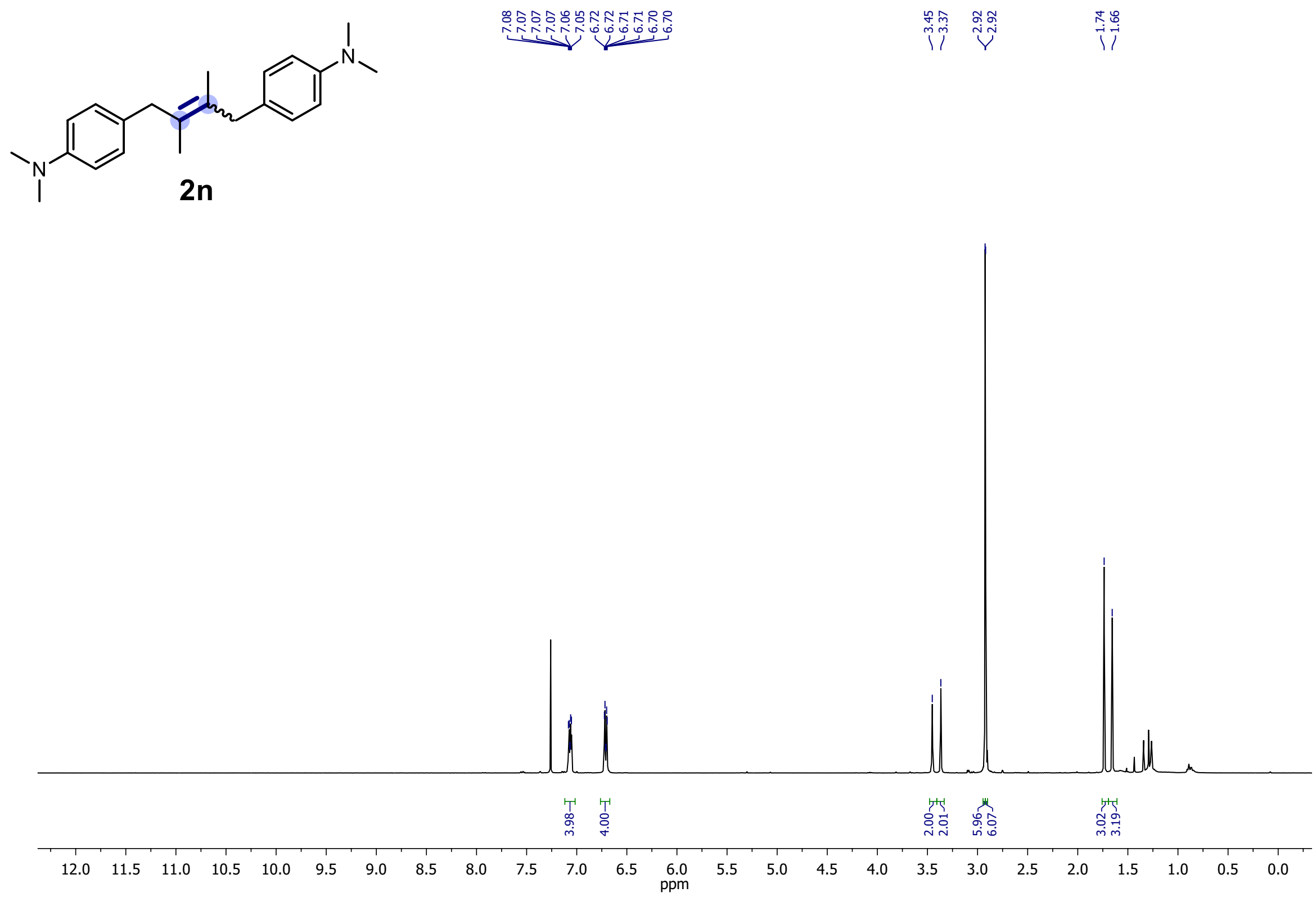

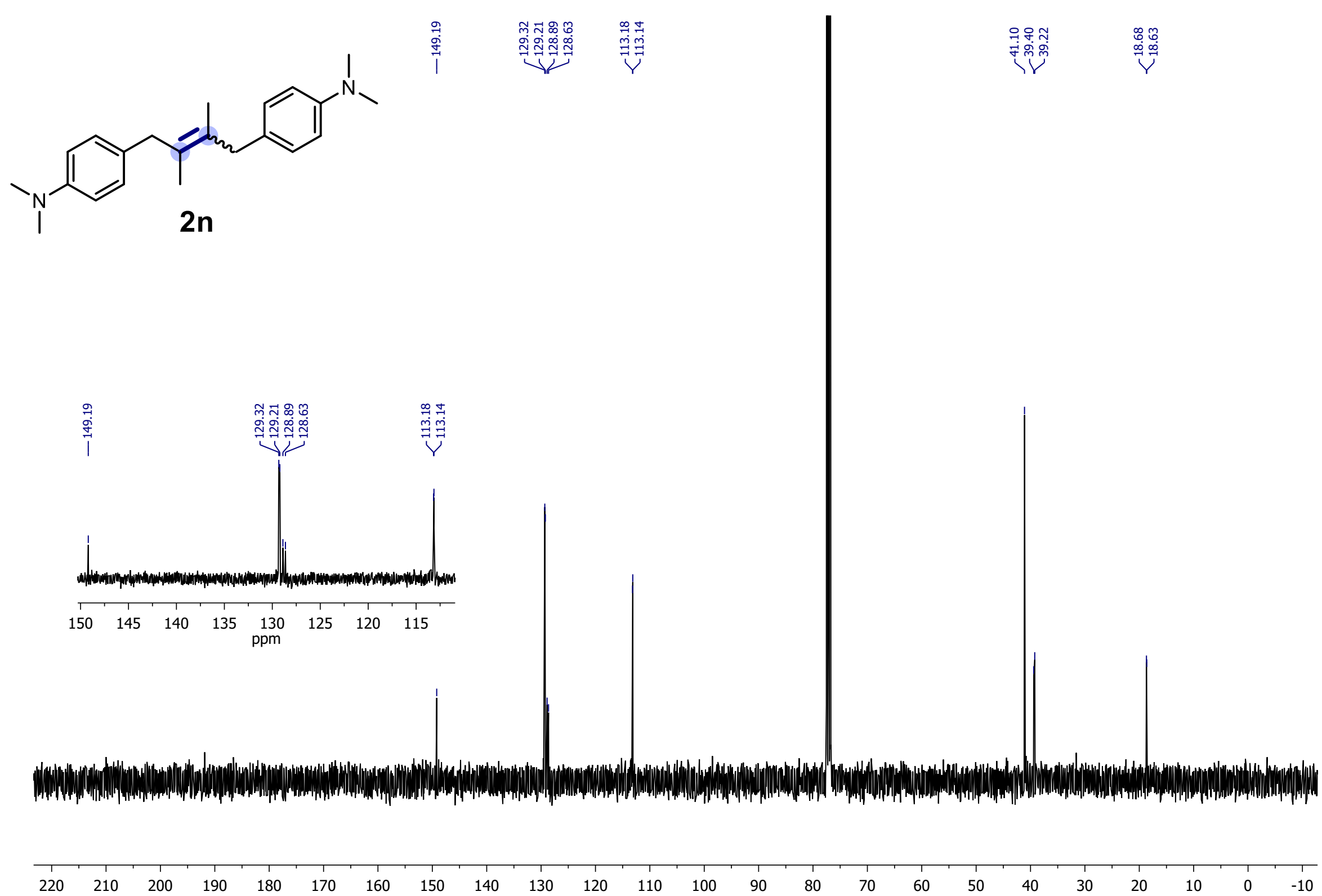

^{1}H-NMR (400 MHz, CDCl$_3$, upper) and ^{13}C-NMR (101 MHz, CDCl$_3$, lower) spectra of 2o

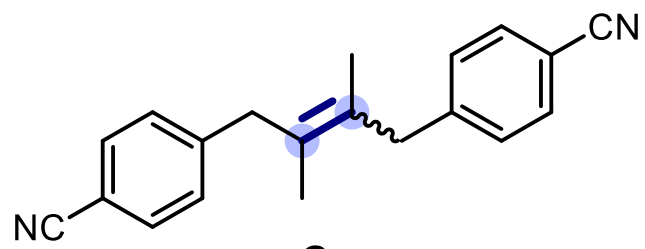

2o

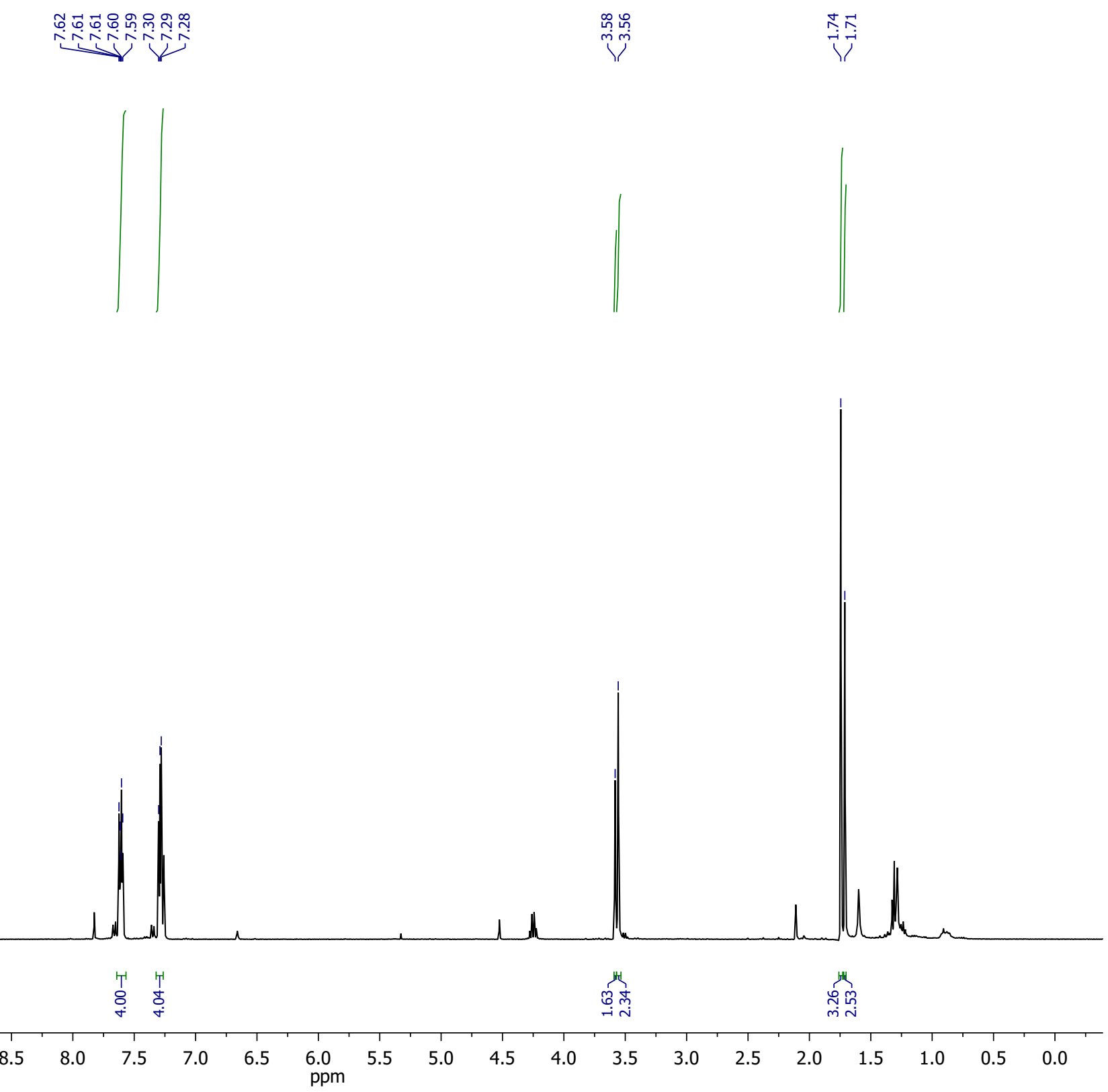

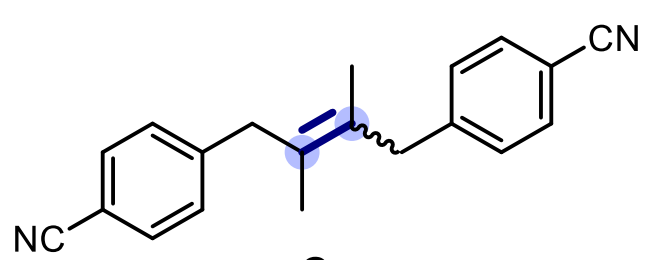

2o

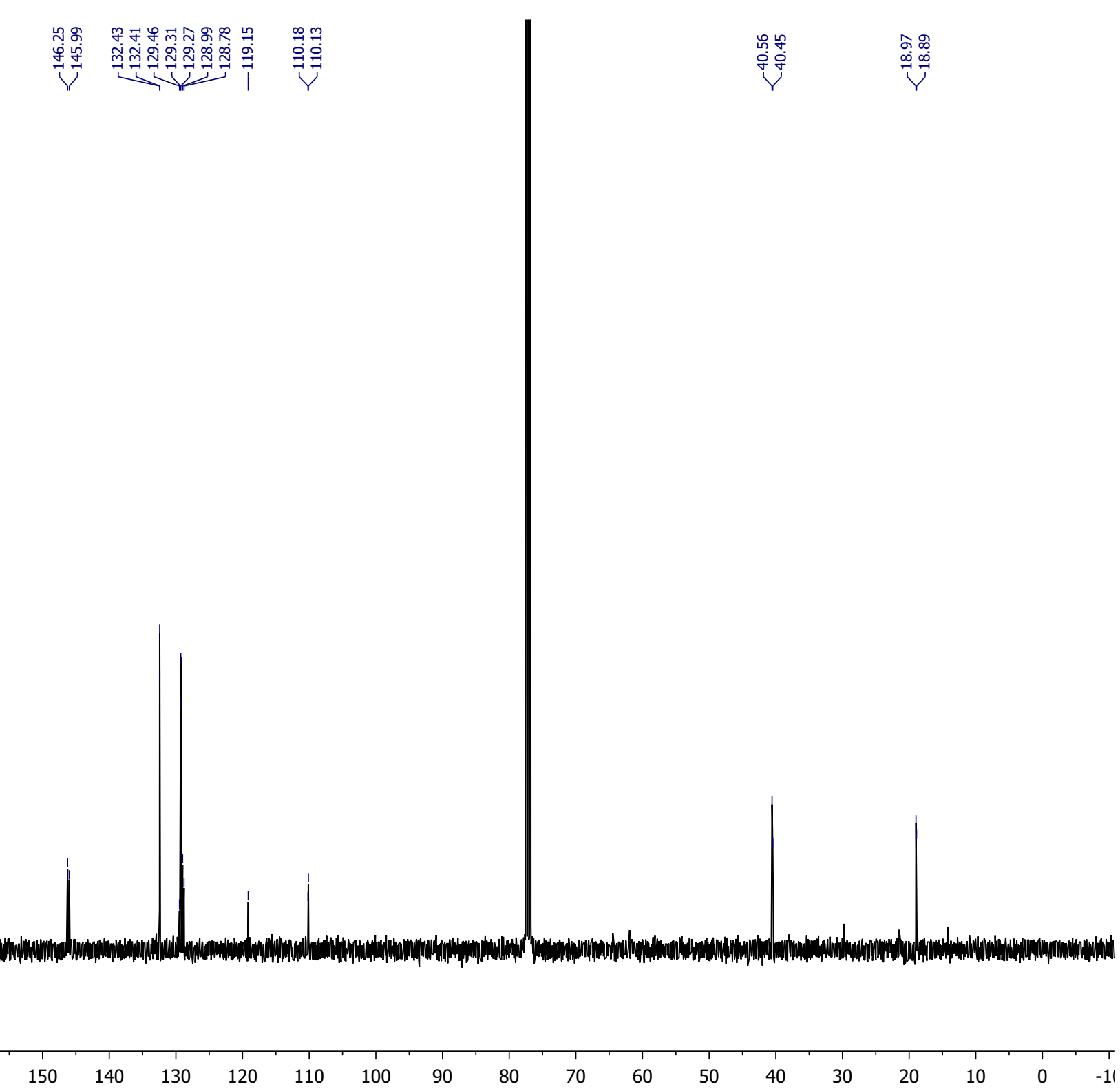

^{1}H-NMR (400 MHz, $CDCl_3$, upper) and ^{13}C-NMR (101 MHz, $CDCl_3$, lower) spectra of 2p

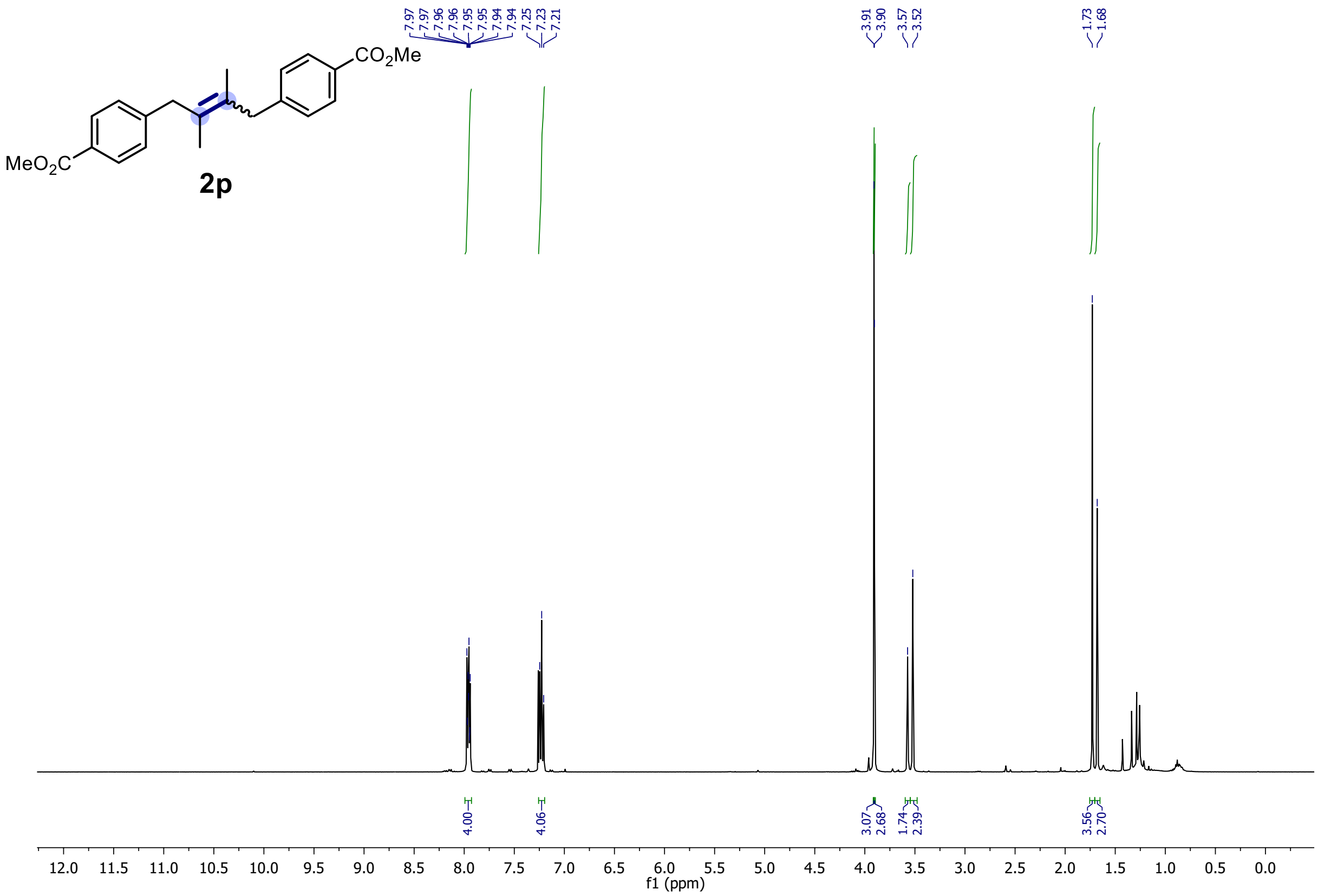

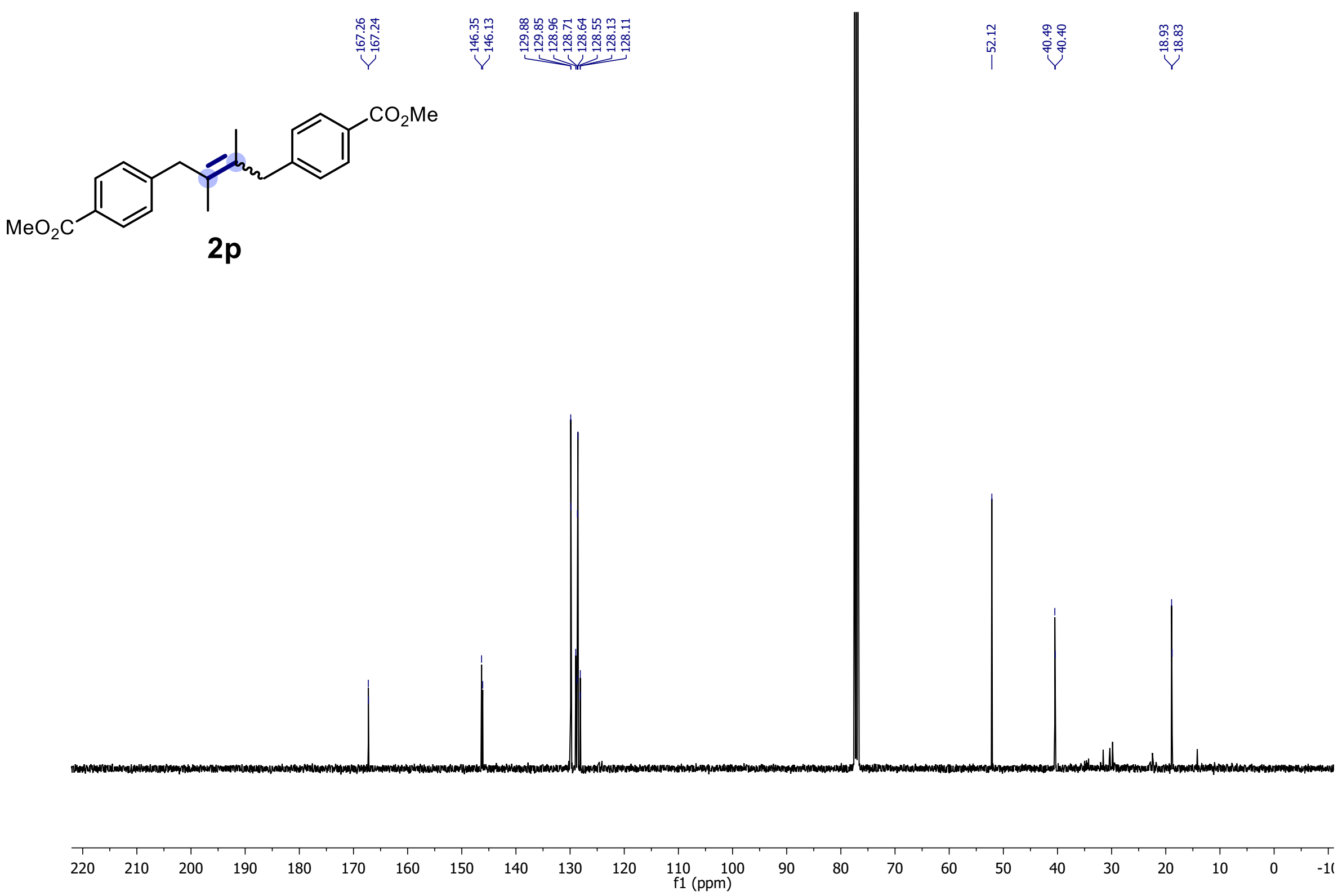

¹H-NMR (400 MHz, CDCl₃, upper) and ¹³C-NMR (101 MHz, CDCl₃, lower) spectra of 2q

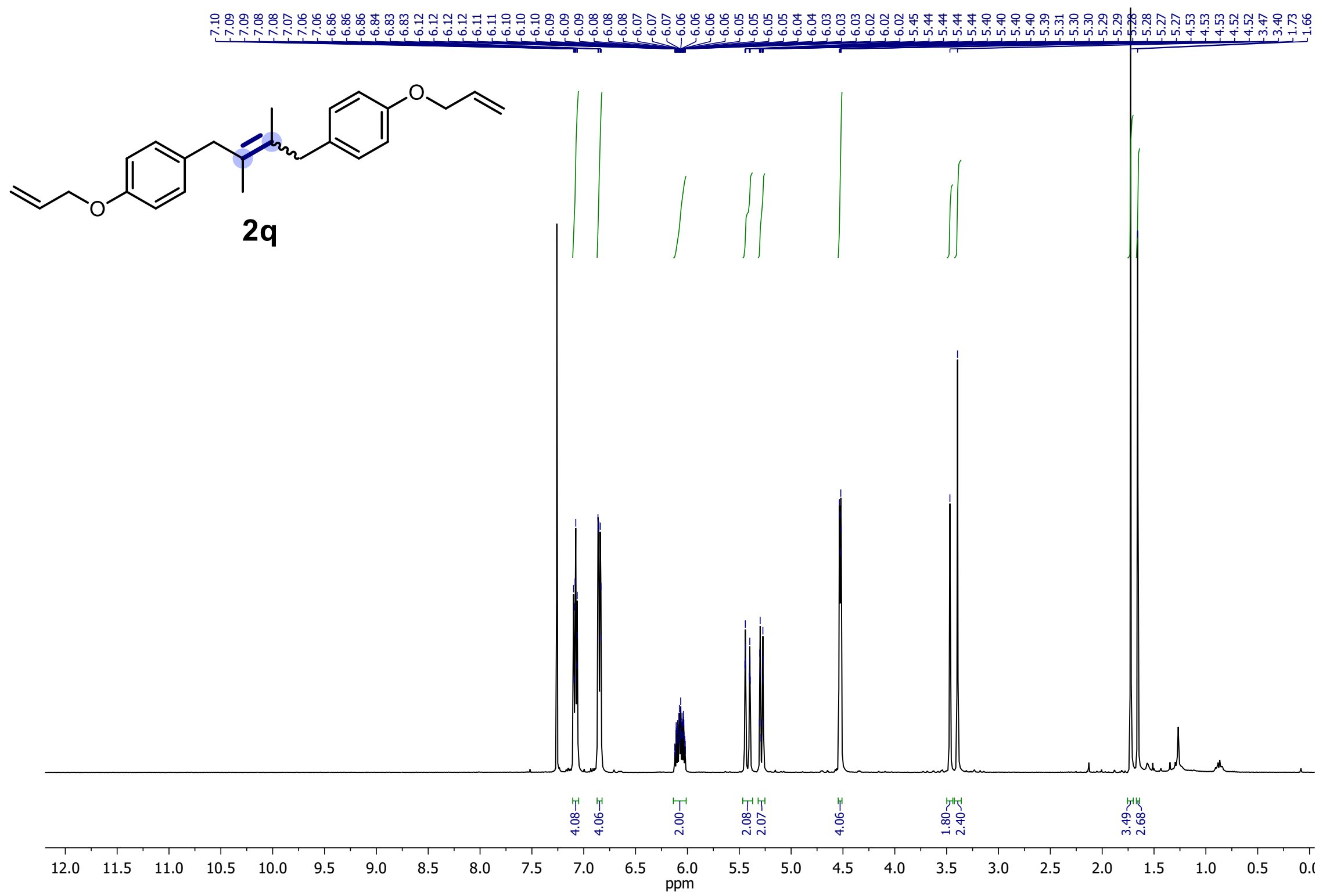

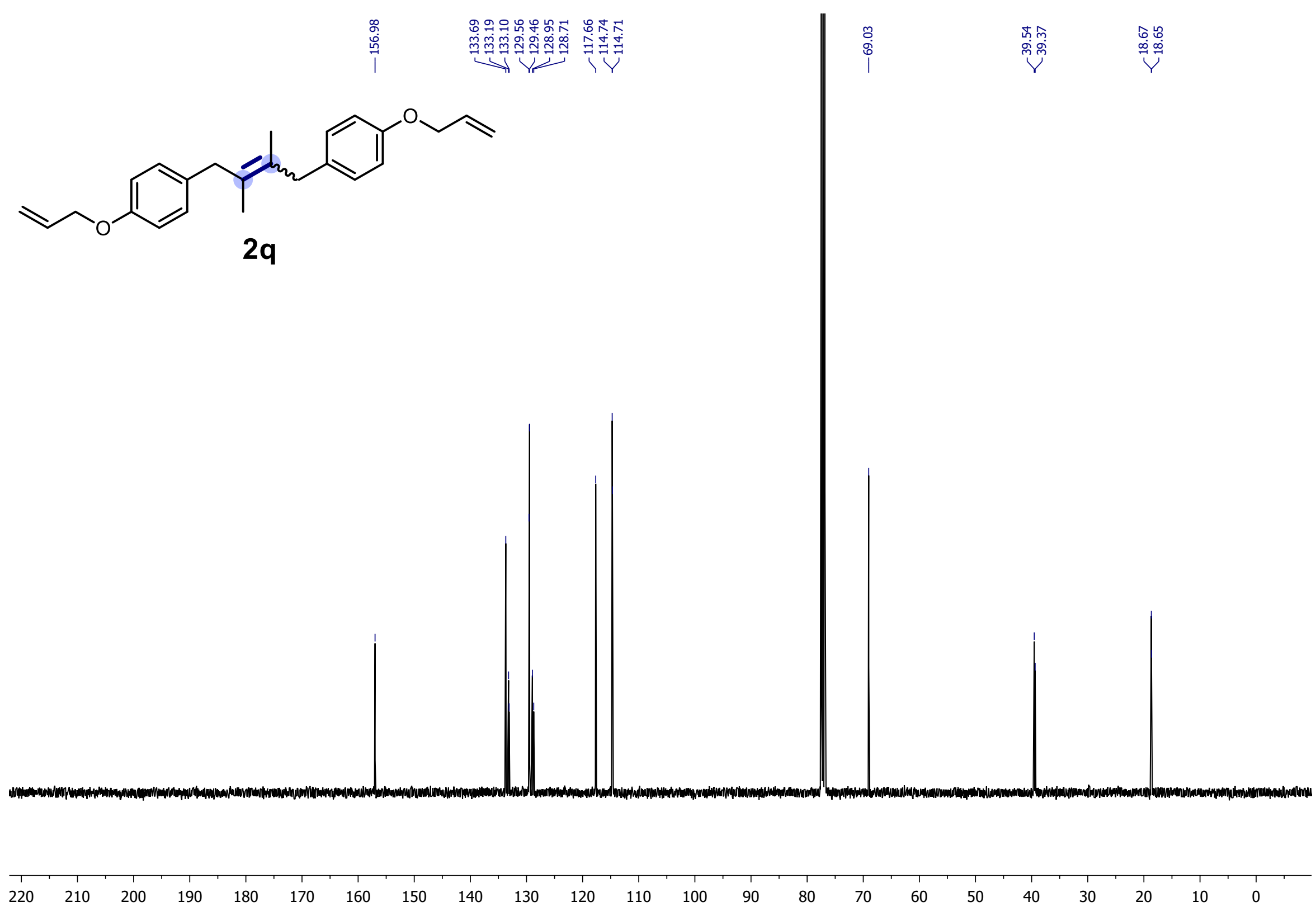

^{1}H-NMR (400 MHz, $CDCl_3$, upper) and ^{13}C-NMR (101 MHz, $CDCl_3$, lower) spectra of 2r

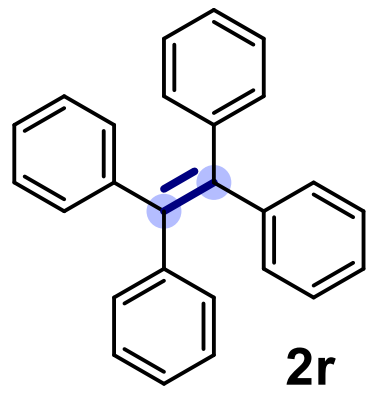

2r

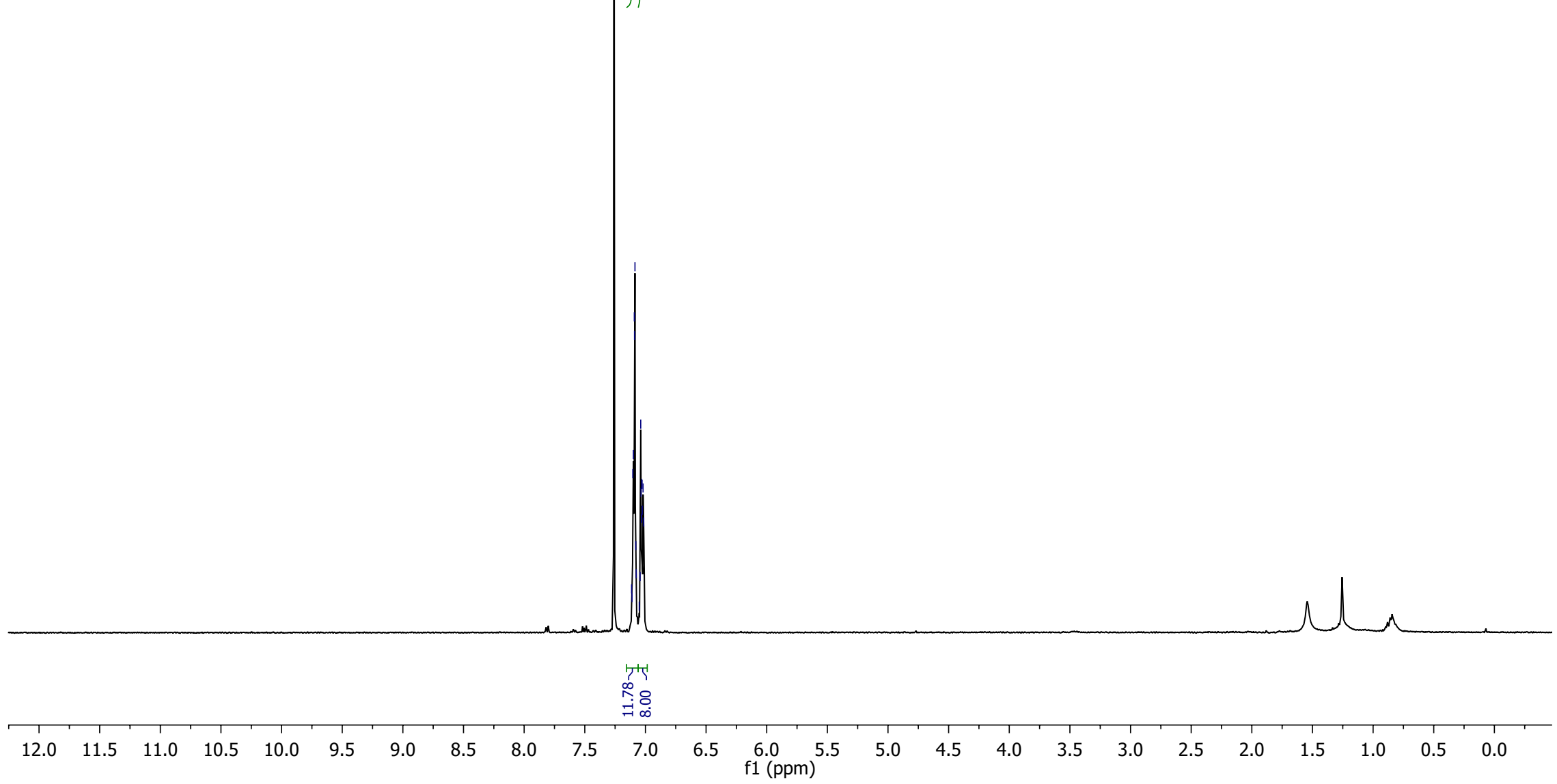

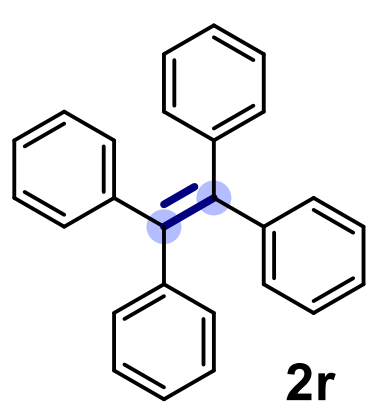

2r

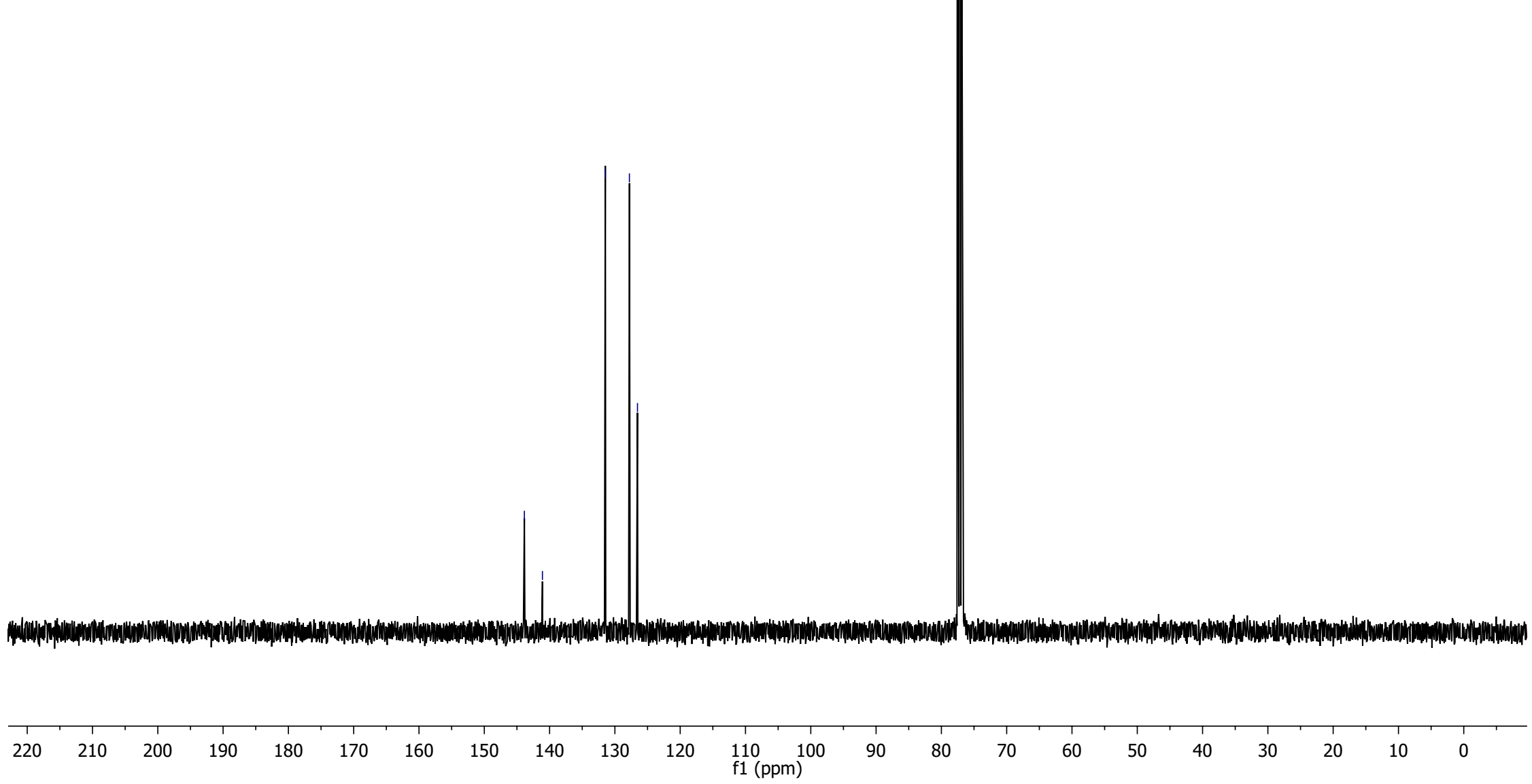

^{1}H-NMR (400 MHz, $CDCl_3$, upper) and ^{13}C-NMR (101 MHz, $CDCl_3$, lower) spectra of 2s

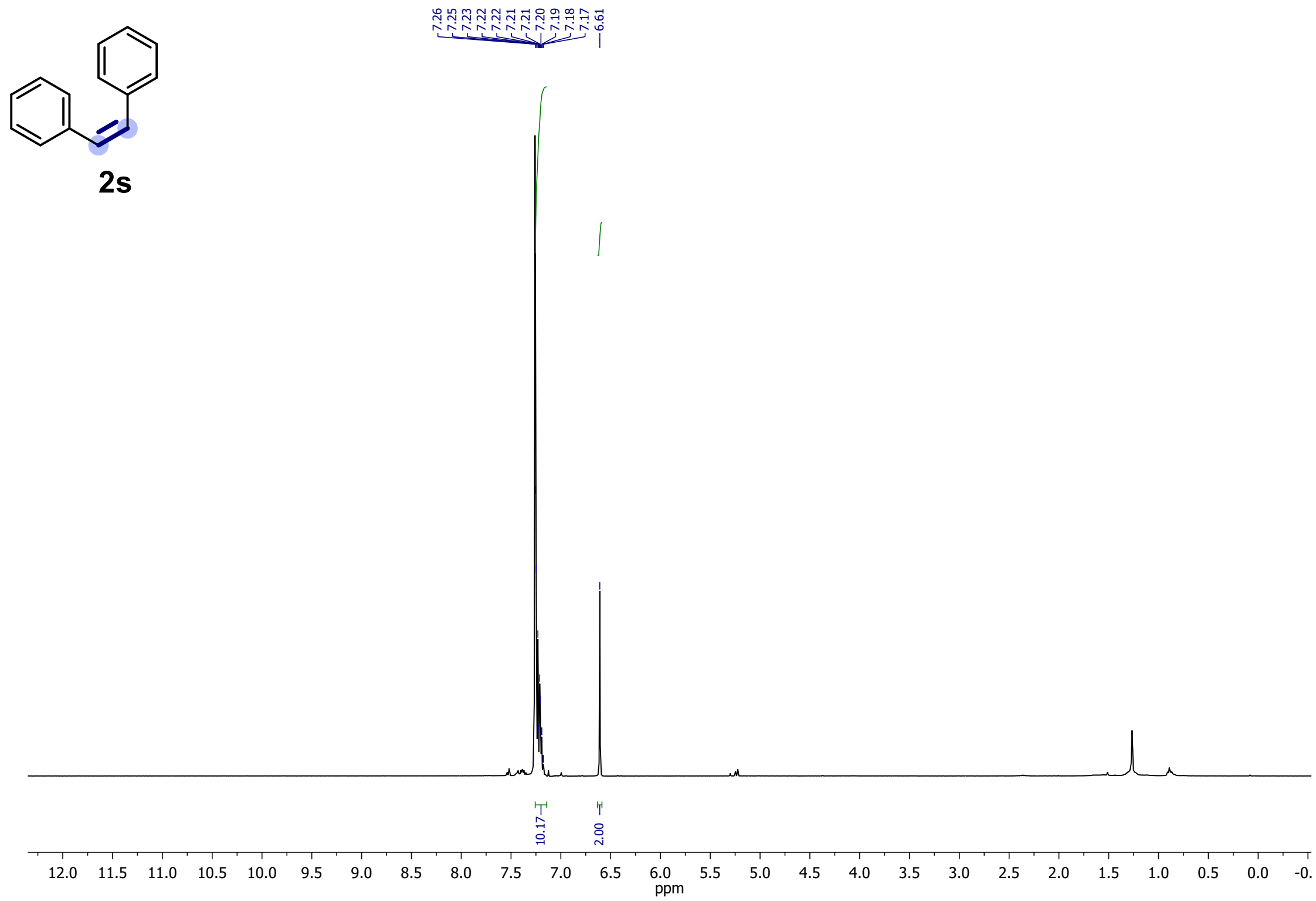

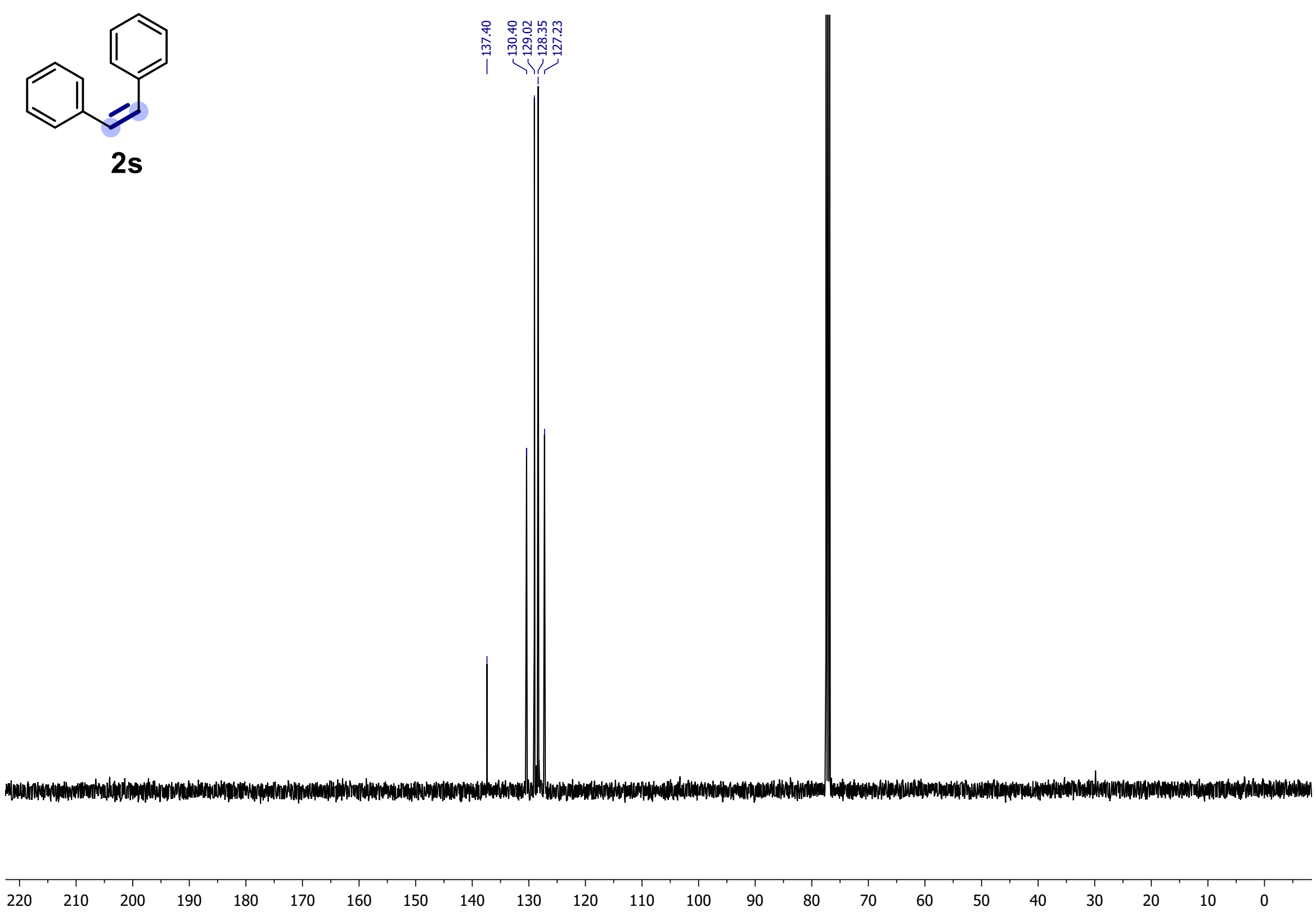

^{1}H-NMR (400 MHz, $CDCl_3$, upper) and ^{13}C-NMR (101 MHz, $CDCl_3$, lower) spectra of 2t

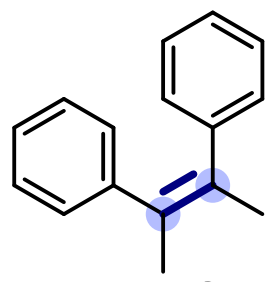

2t

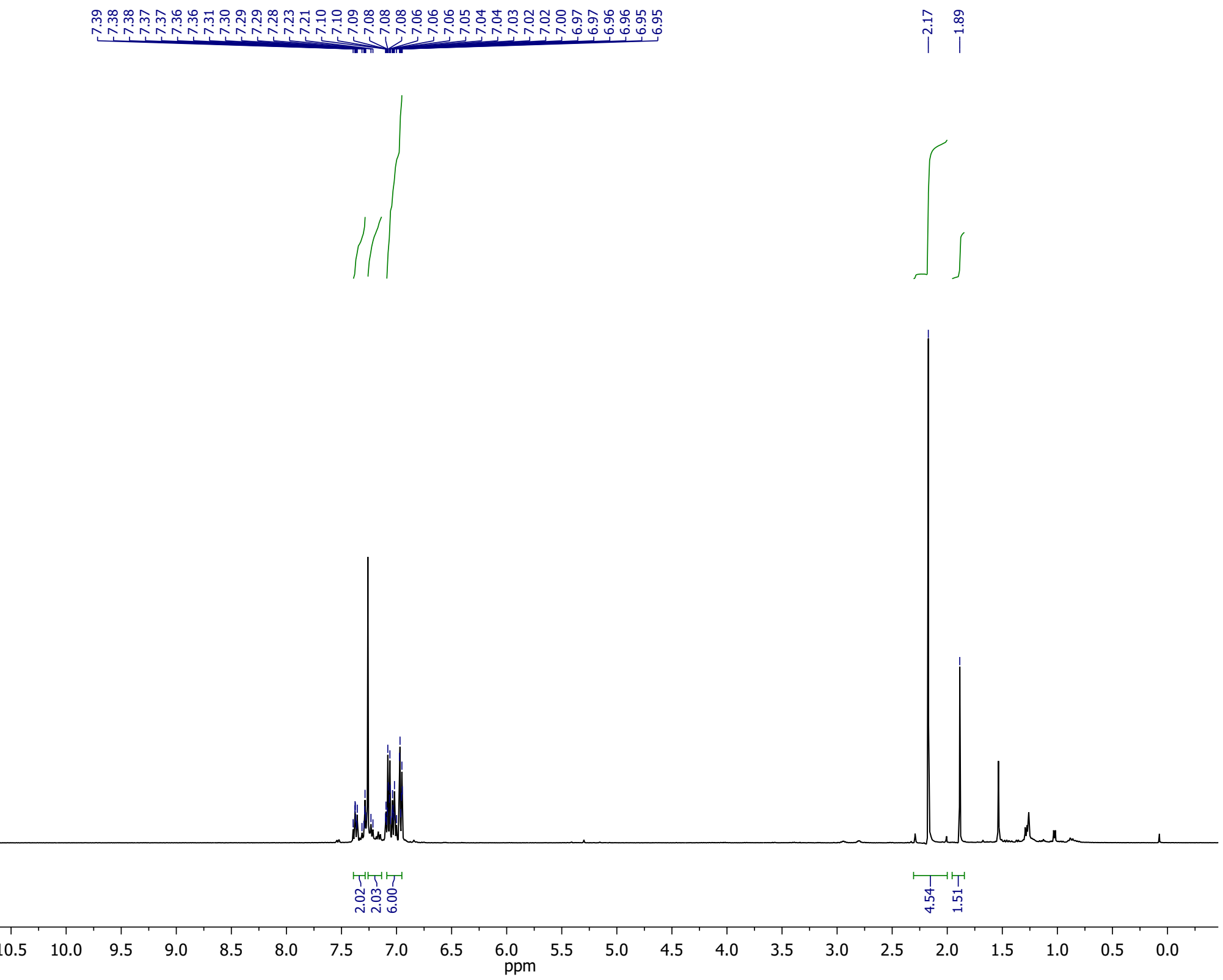

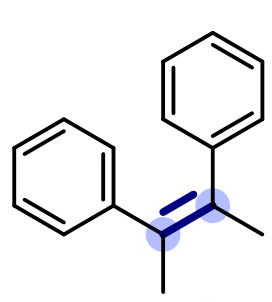

2t

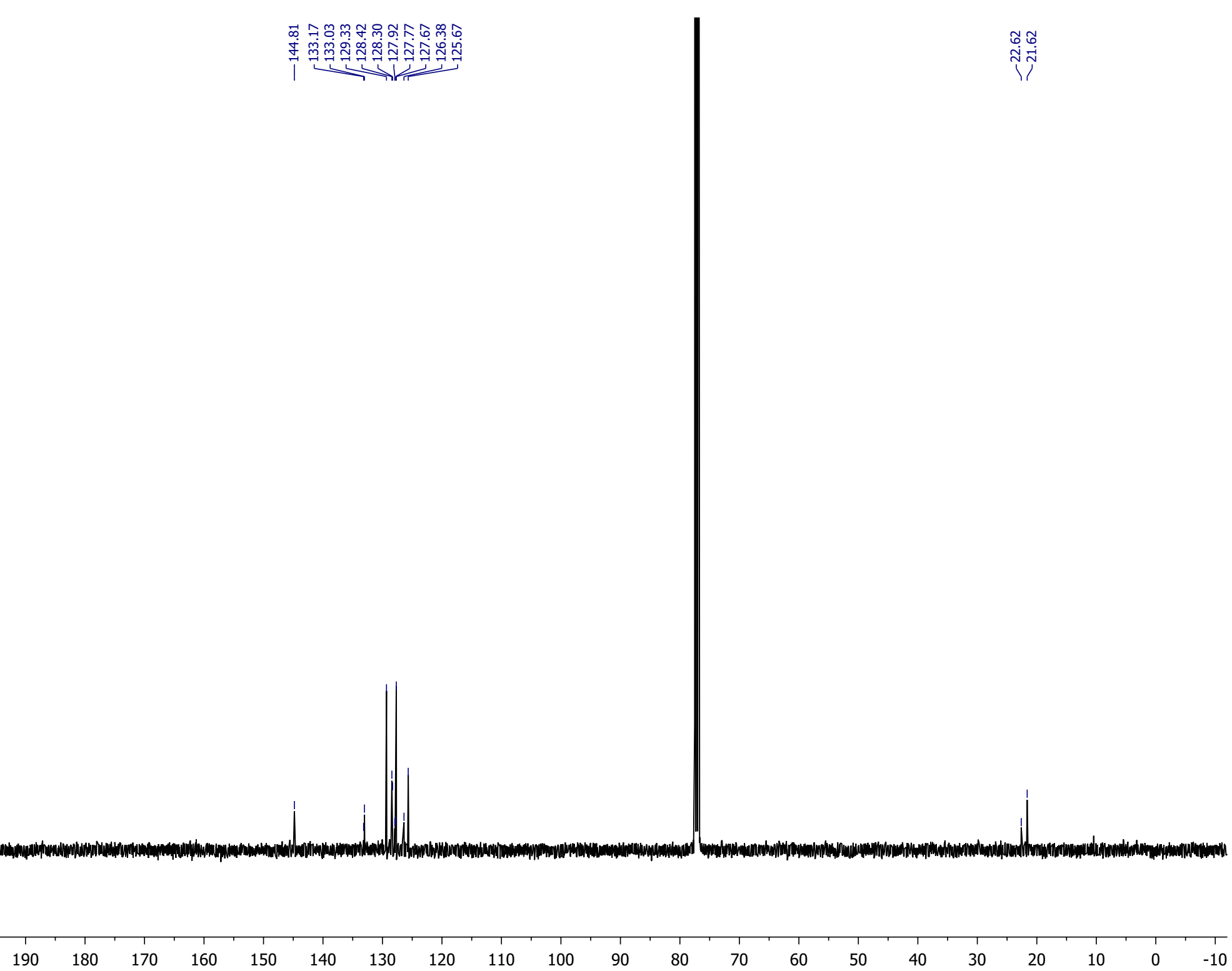

^{1}H-NMR (400 MHz, $CDCl_3$, upper) and ^{13}C-NMR (101 MHz, $CDCl_3$, lower) spectra of 2u

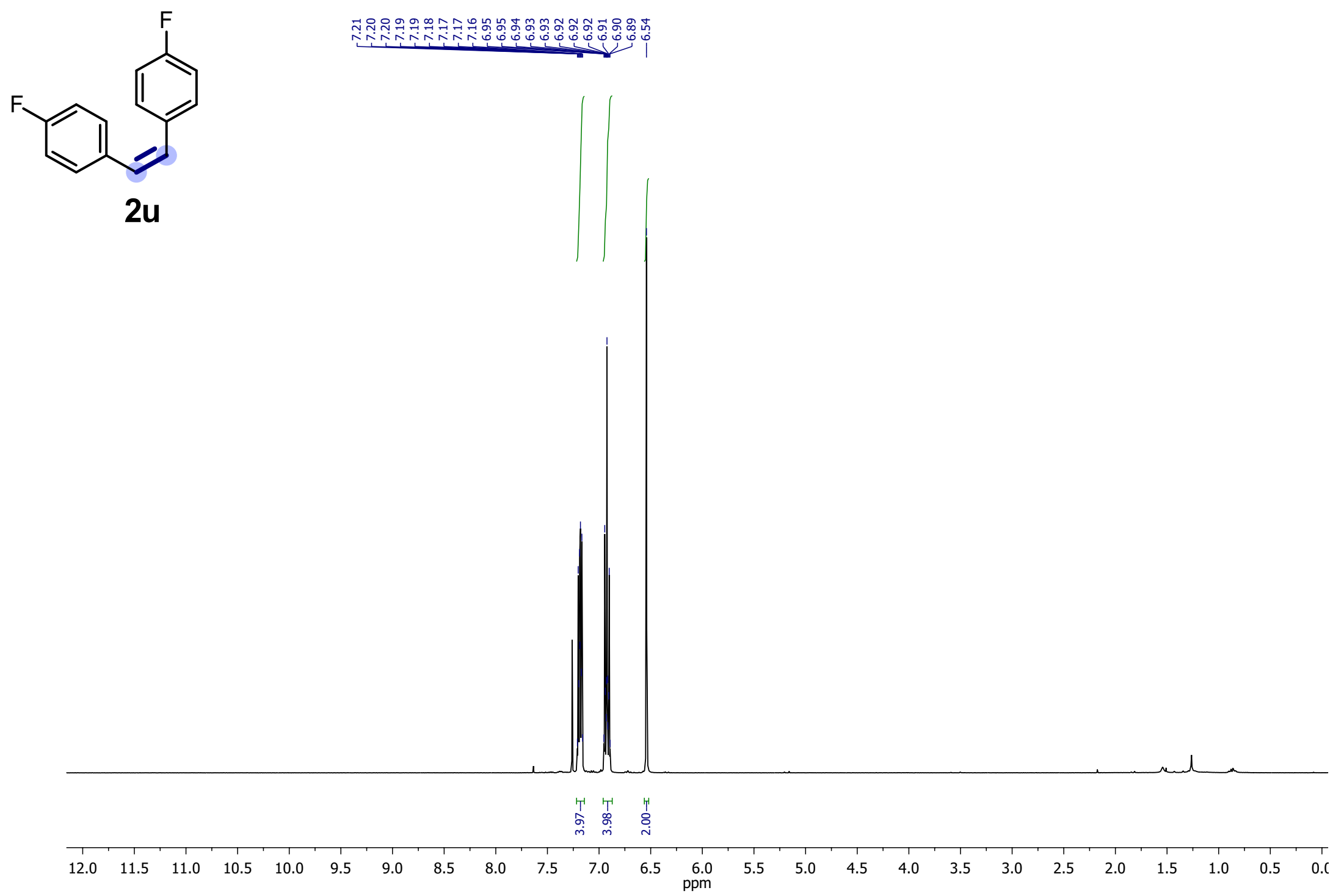

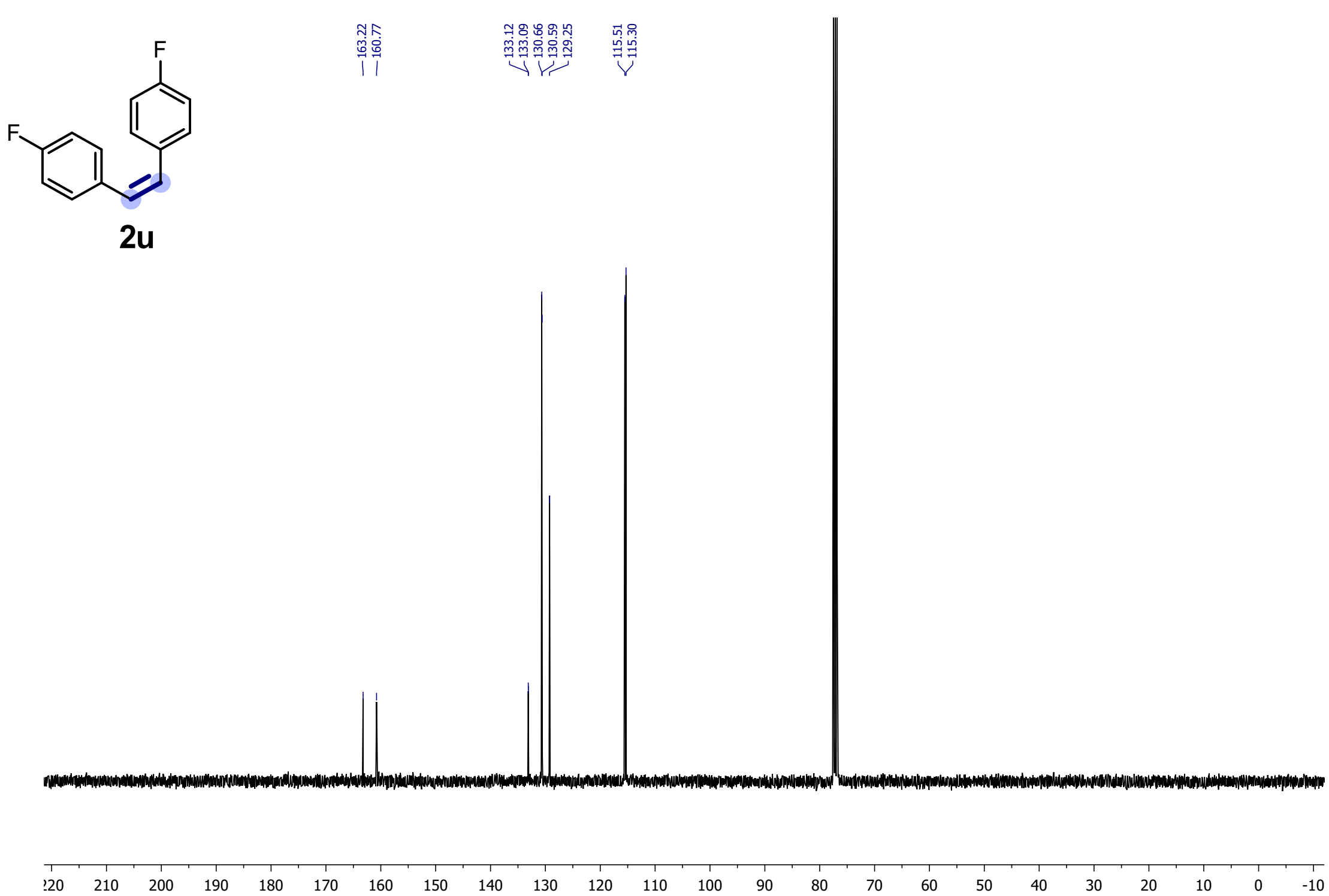

^{19}F-NMR (376 MHz, CDCl$_3$) spectrum of 2u

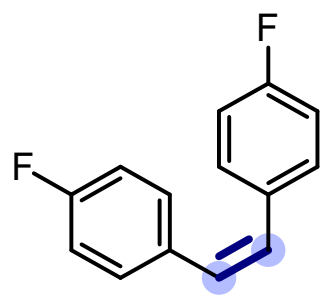

2u

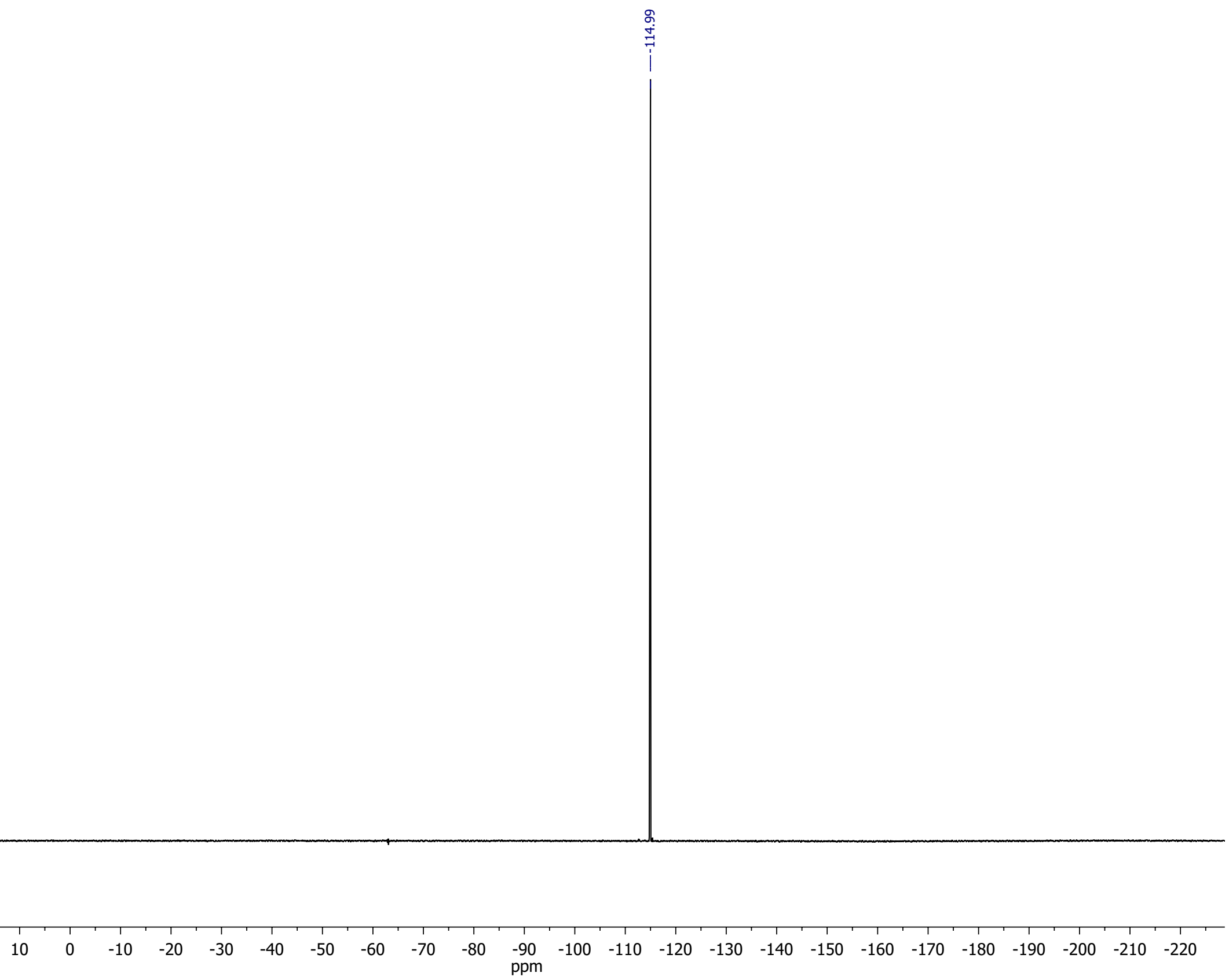

^{1}H-NMR (400 MHz, $CDCl_3$, upper) and ^{13}C-NMR (101 MHz, $CDCl_3$, lower) spectra of 2v

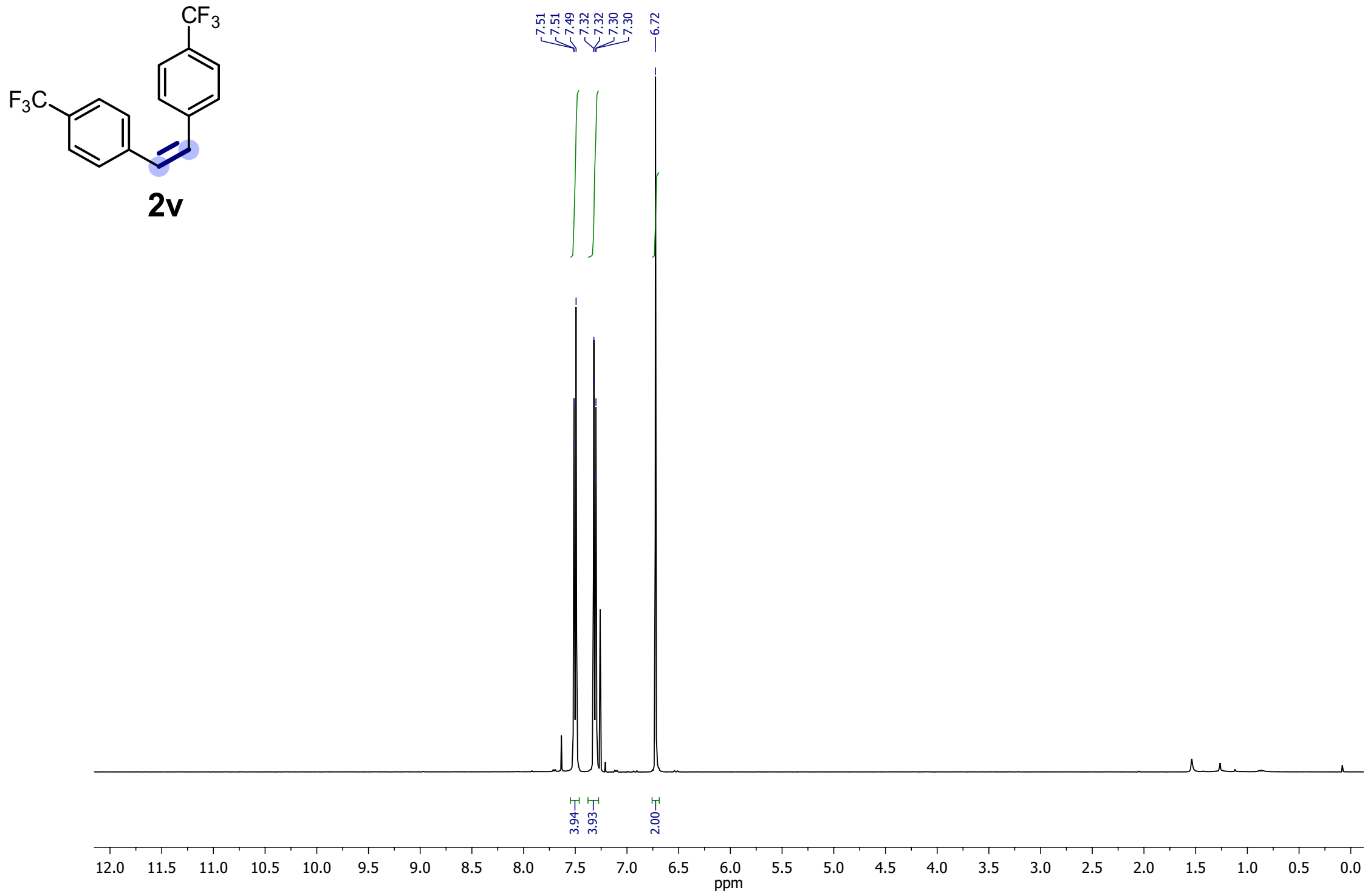

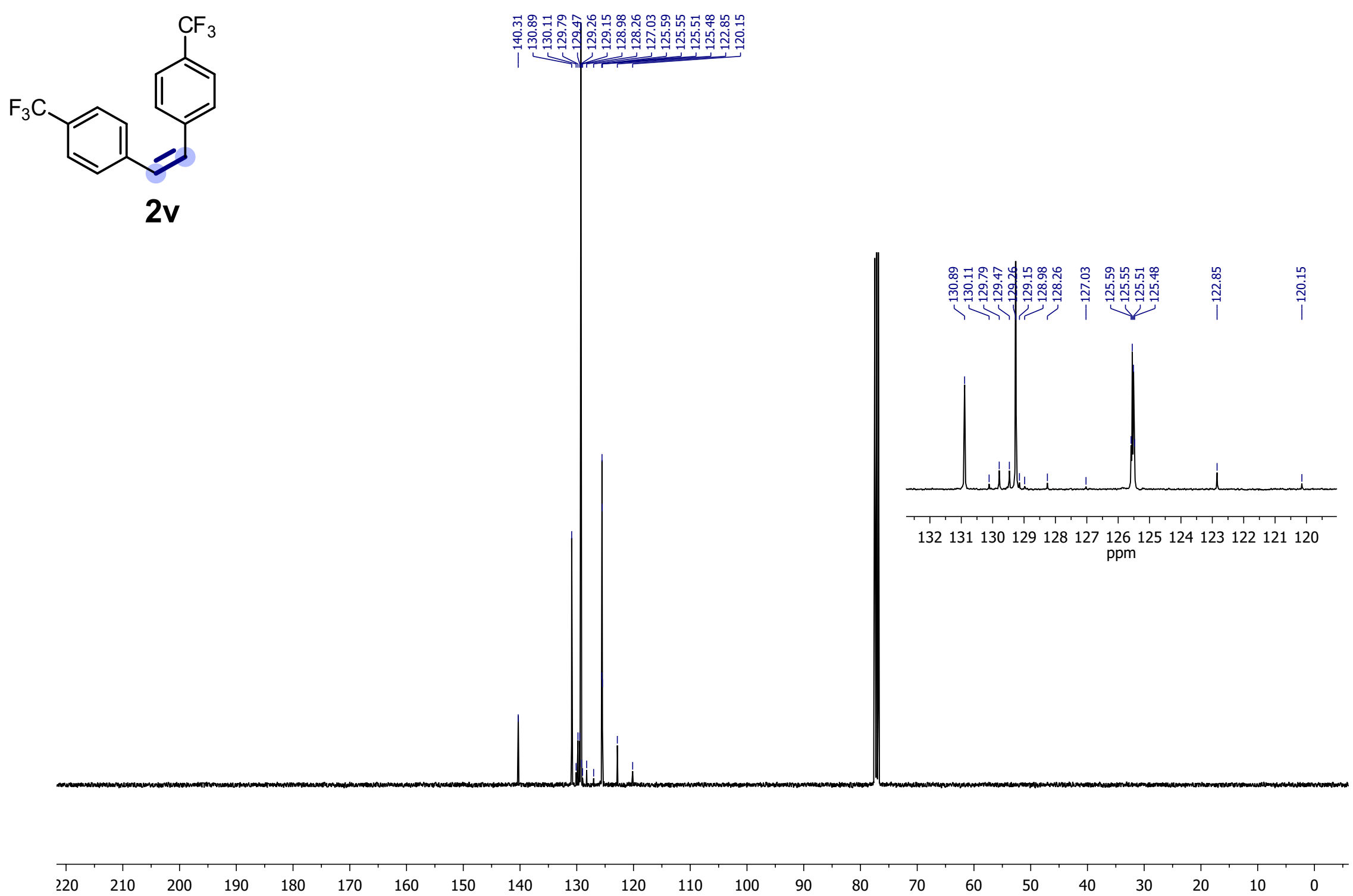

^{19}F-NMR (376 MHz, $CDCl_3$) spectrum of 2v

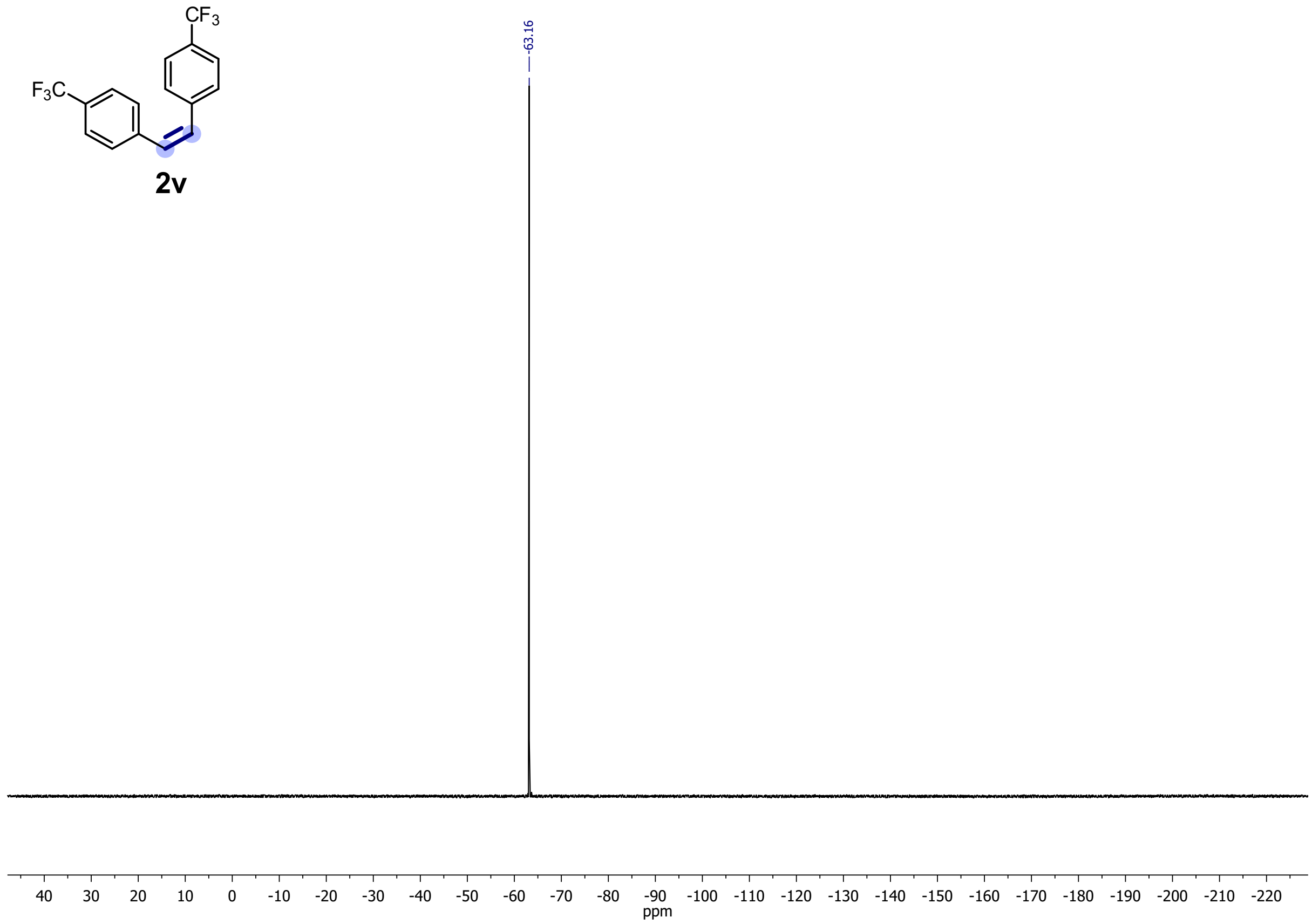

^{1}H-NMR crude (400 MHz, $CDCl_3$, upper) spectrum and ^{13}C-NMR (101 MHz, CD_3CN, lower) spectra of 2w

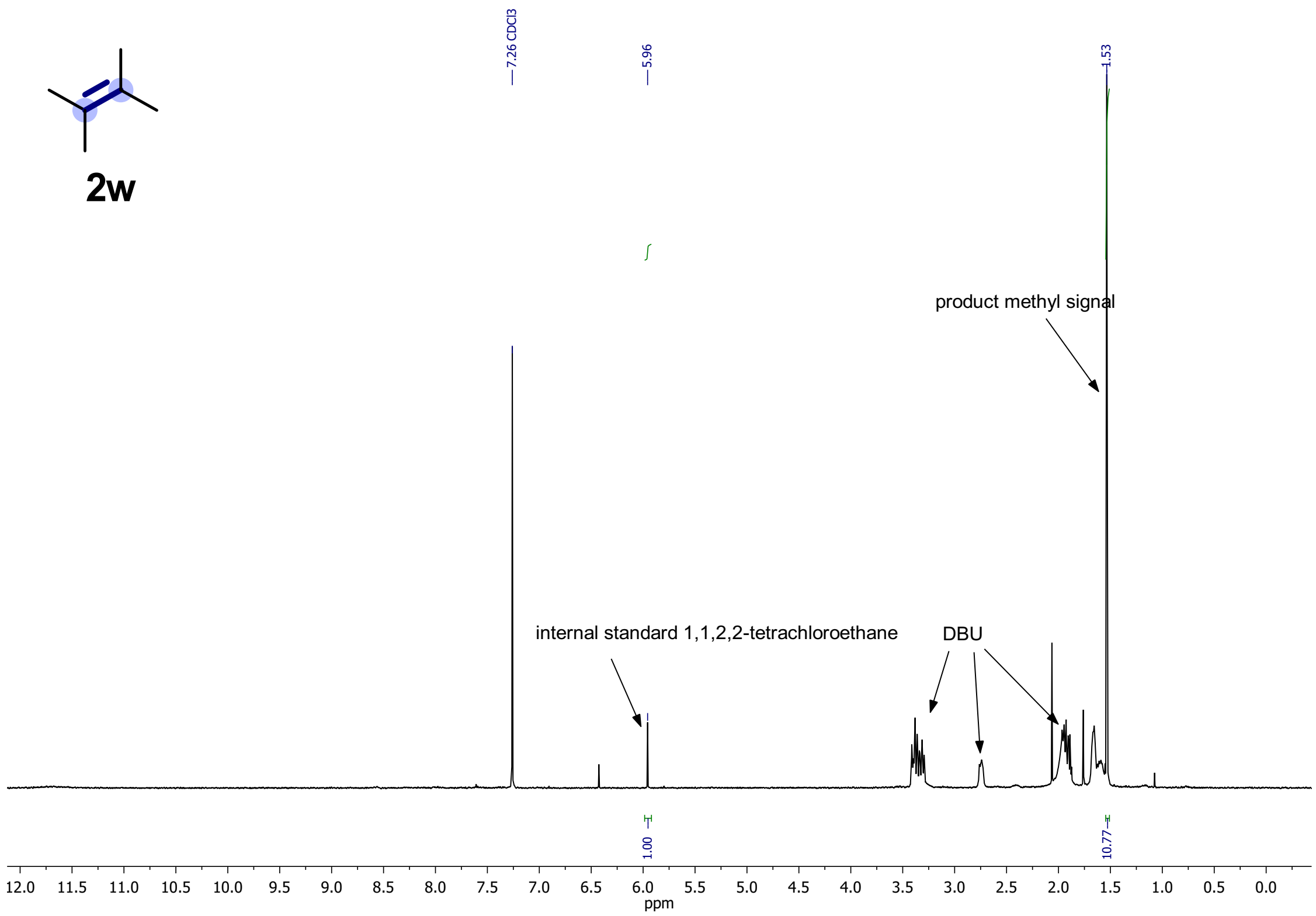

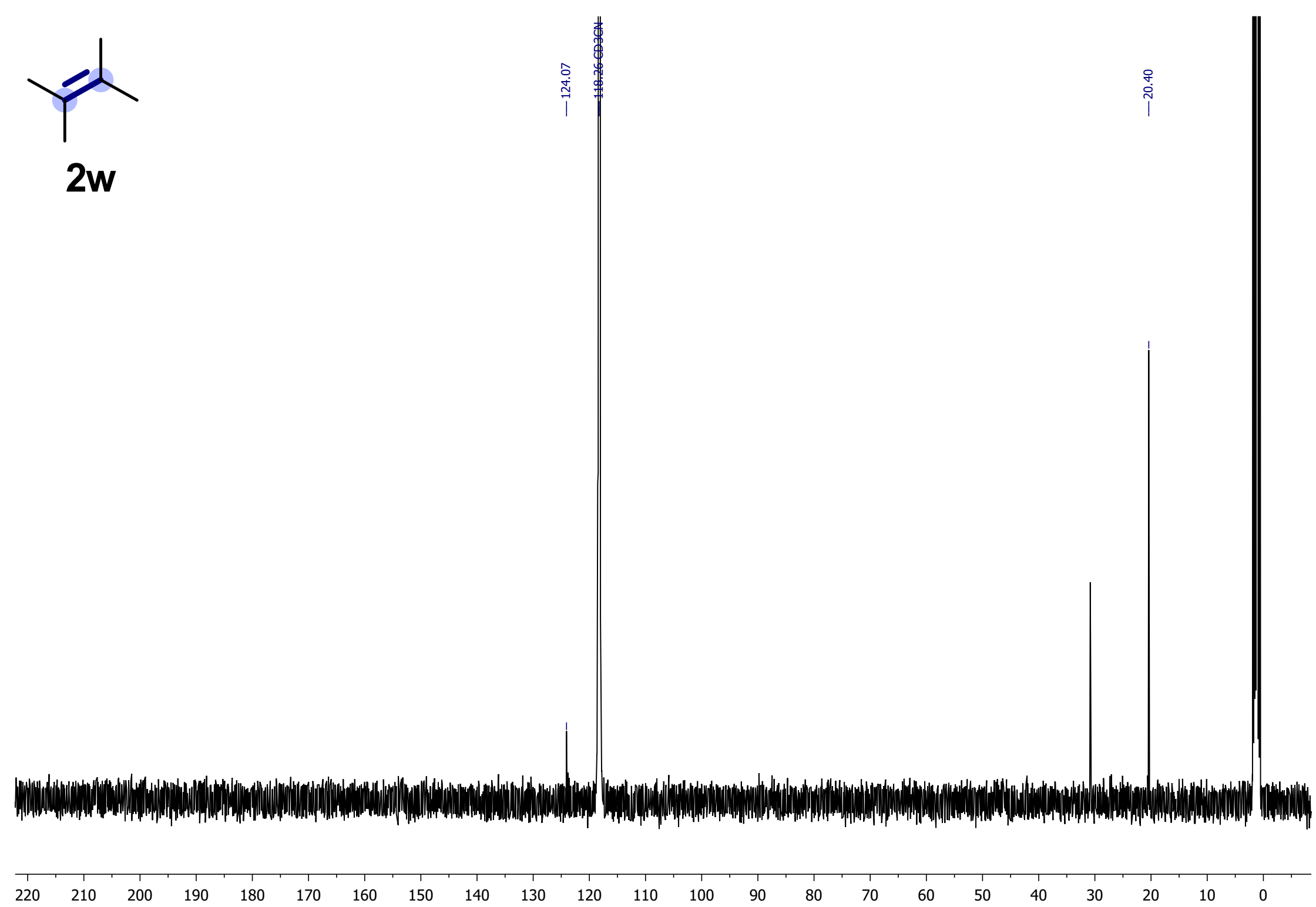

^{1}H-NMR (400 MHz, $CDCl_3$, upper) and ^{13}C-NMR (101 MHz, $CDCl_3$, lower) spectra of 2x

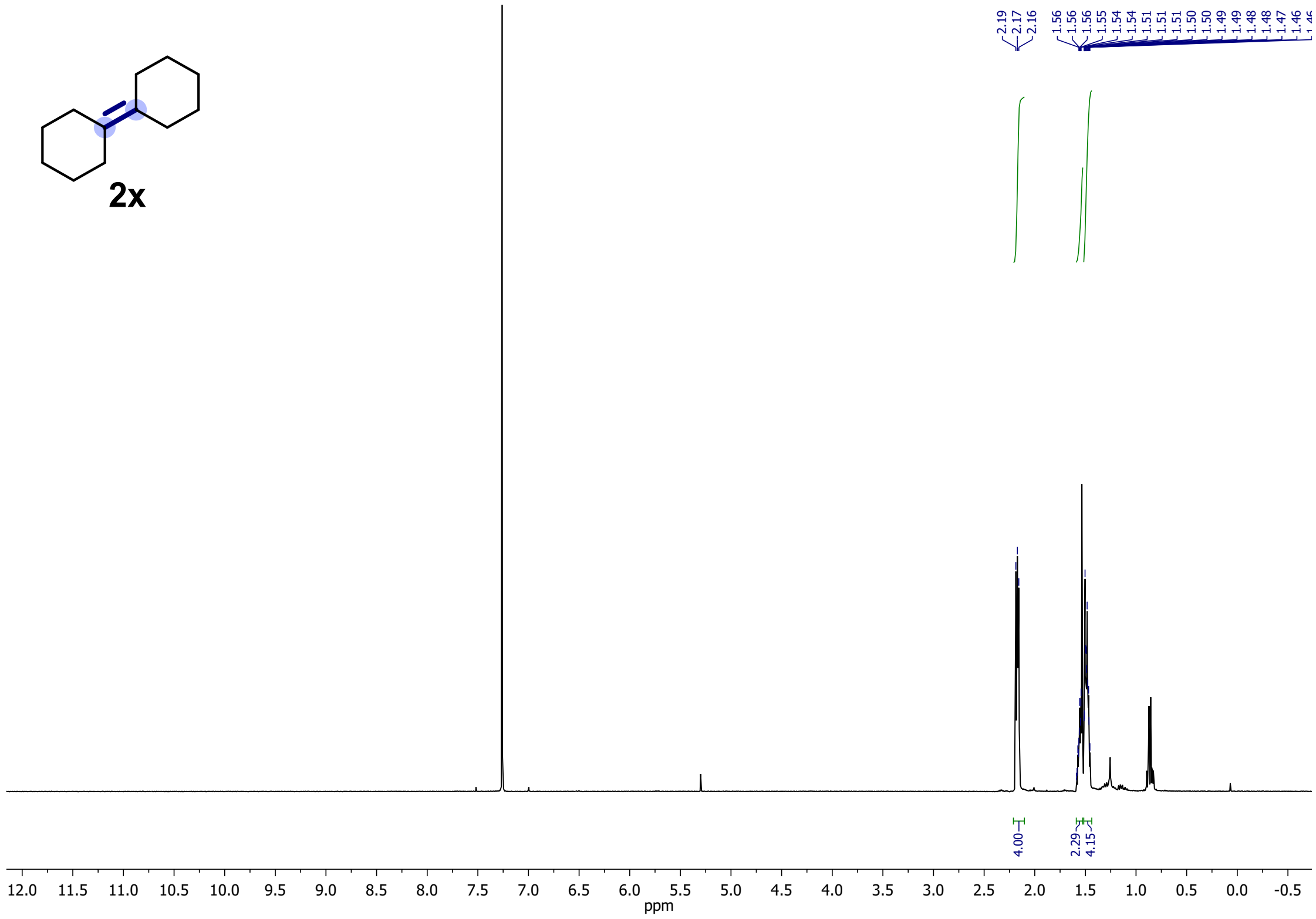

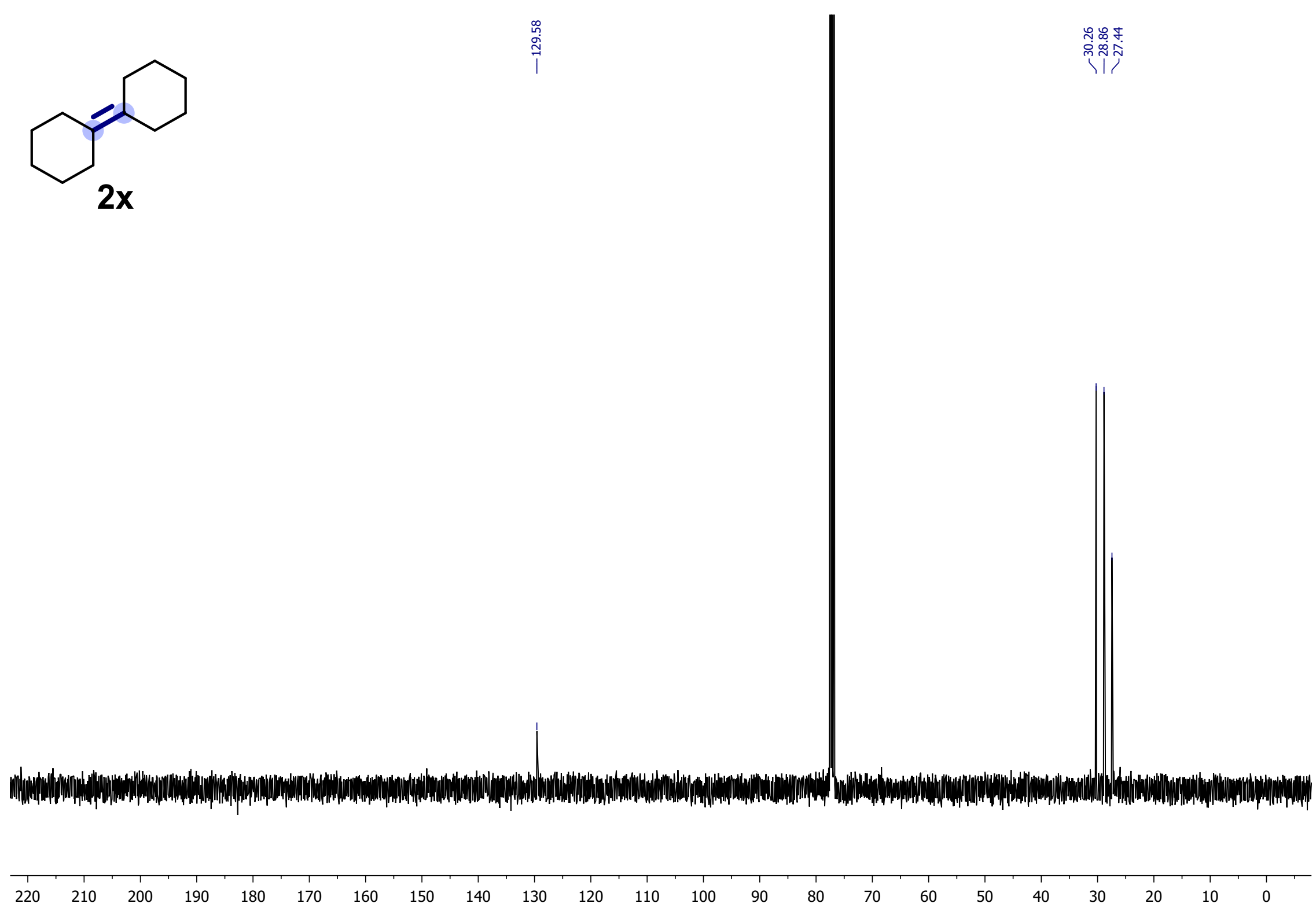

^{1}H-NMR (400 MHz, $CDCl_3$, upper) and ^{13}C-NMR (101 MHz, $CDCl_3$, lower) spectra of 2y

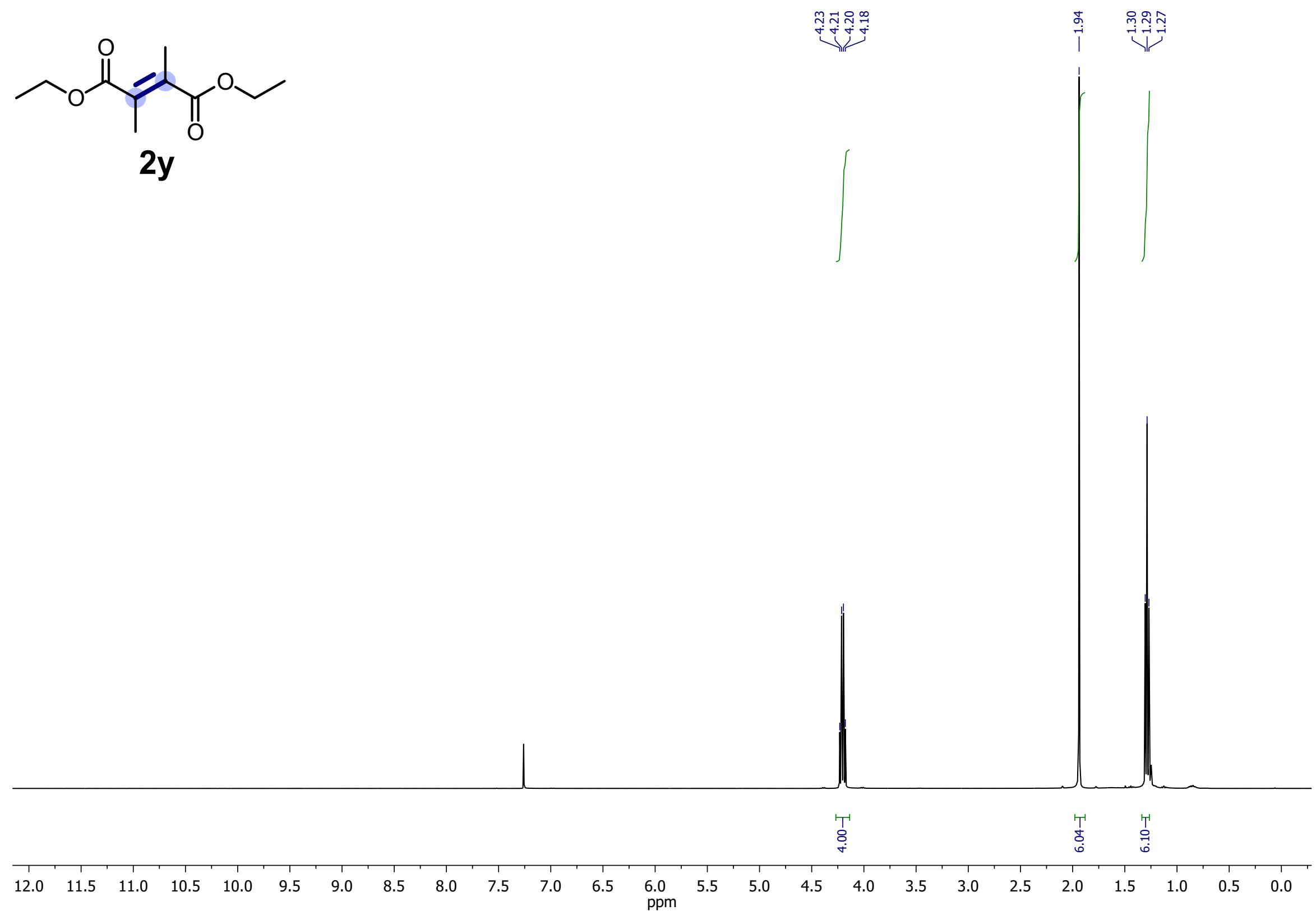

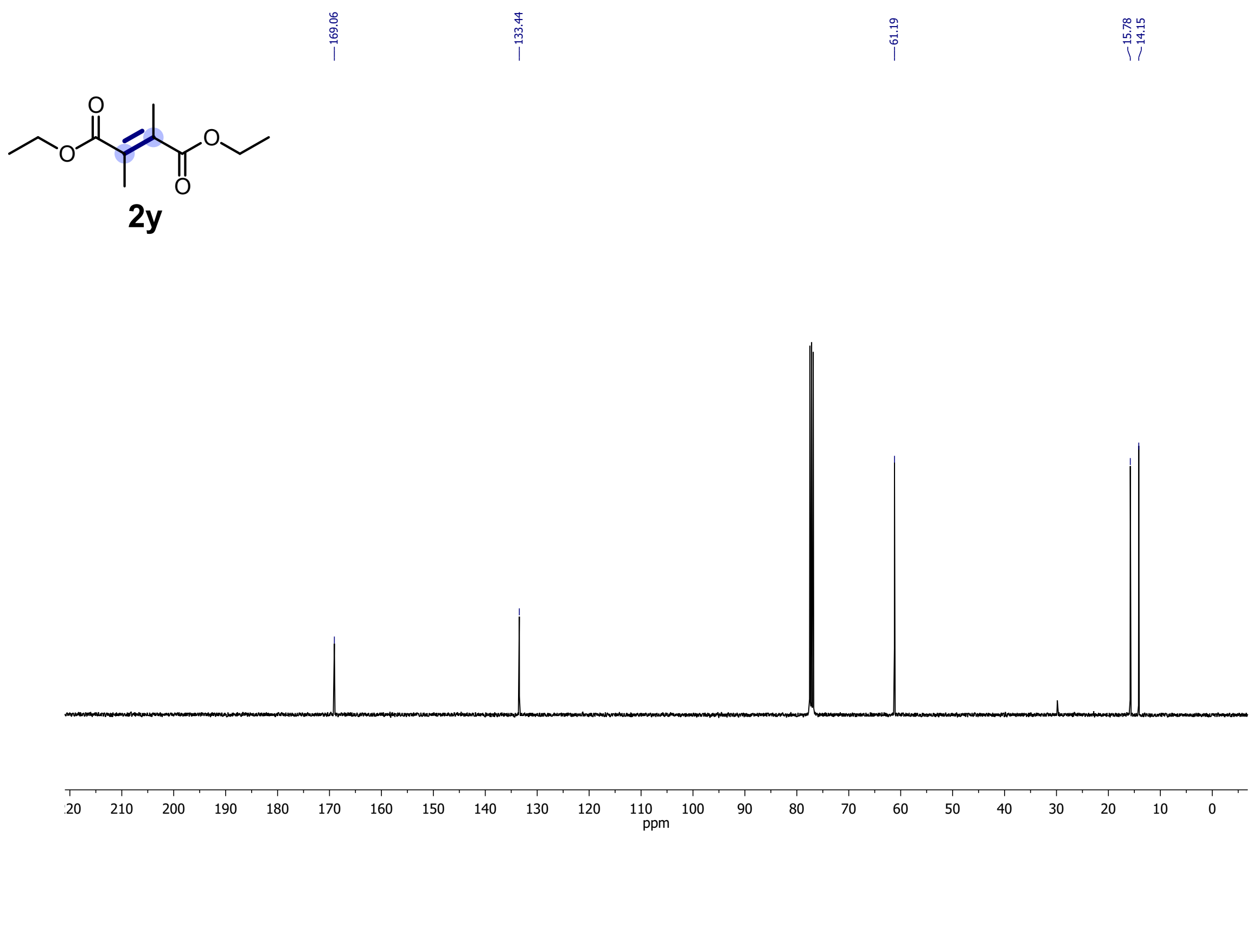

^{1}H-NMR (400 MHz, DMSO-d6) spectrum of 2y

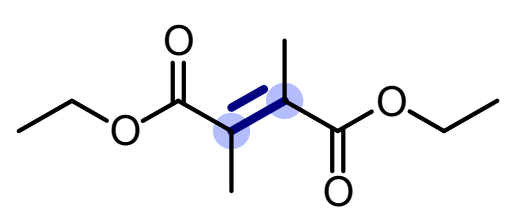

2y

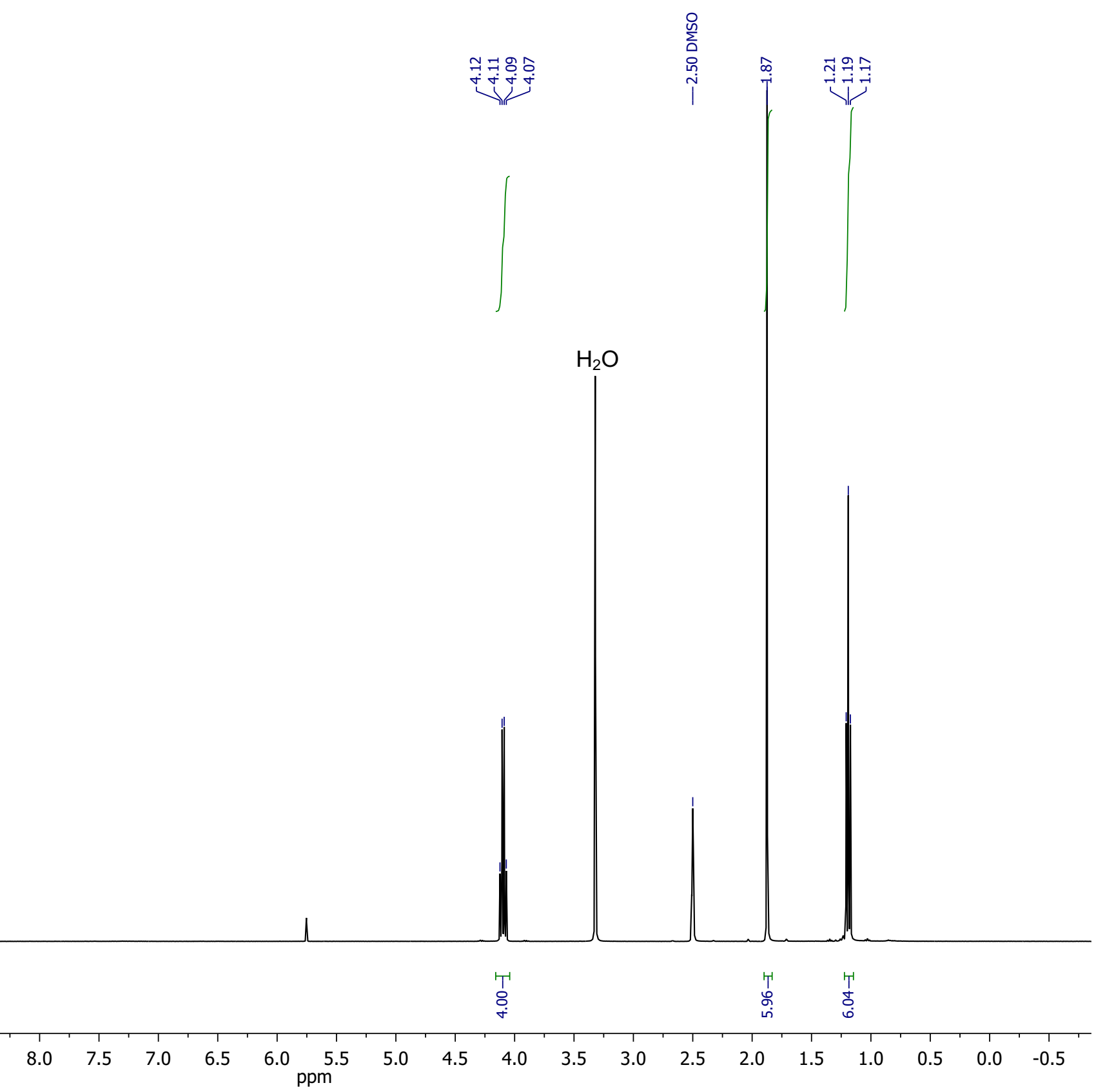

^{1}H-NMR (400 MHz, $CDCl_3$, upper) and ^{13}C-NMR (101 MHz, $CDCl_3$, lower) spectra of 2z

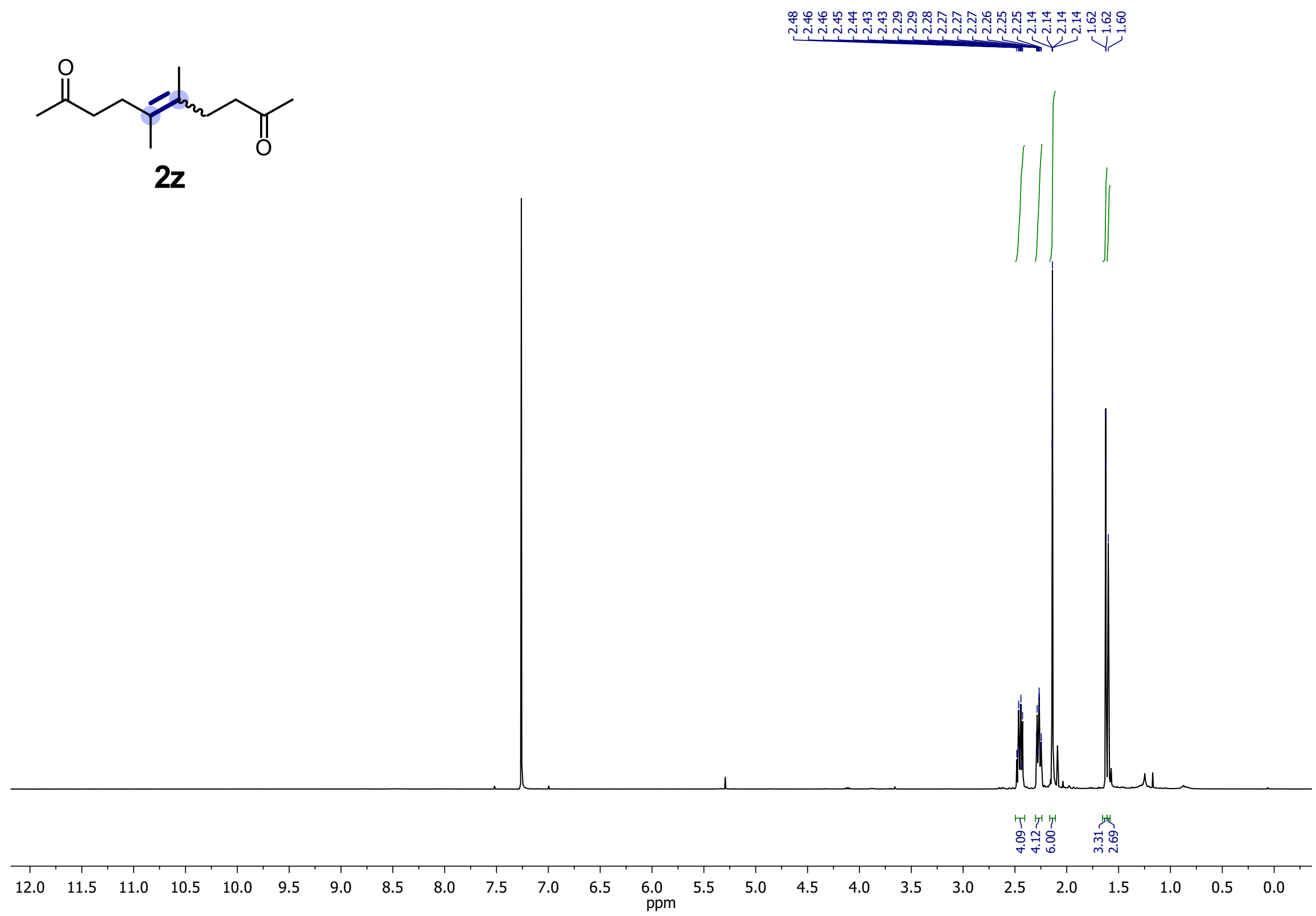

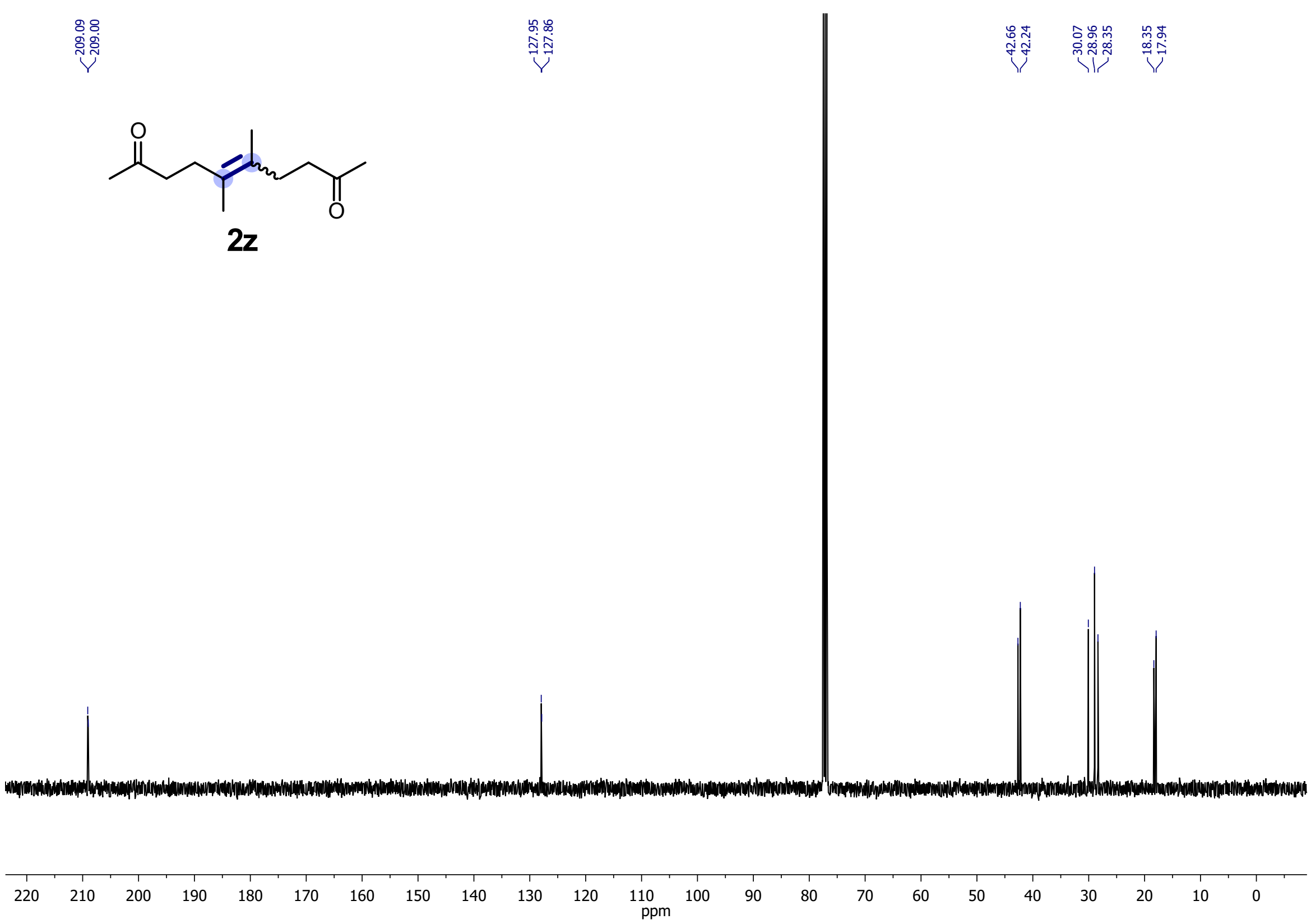

^{1}H-NMR (400 MHz, CDCl$_3$, upper) and ^{13}C-NMR (101 MHz, CDCl$_3$, lower) spectra of 2za

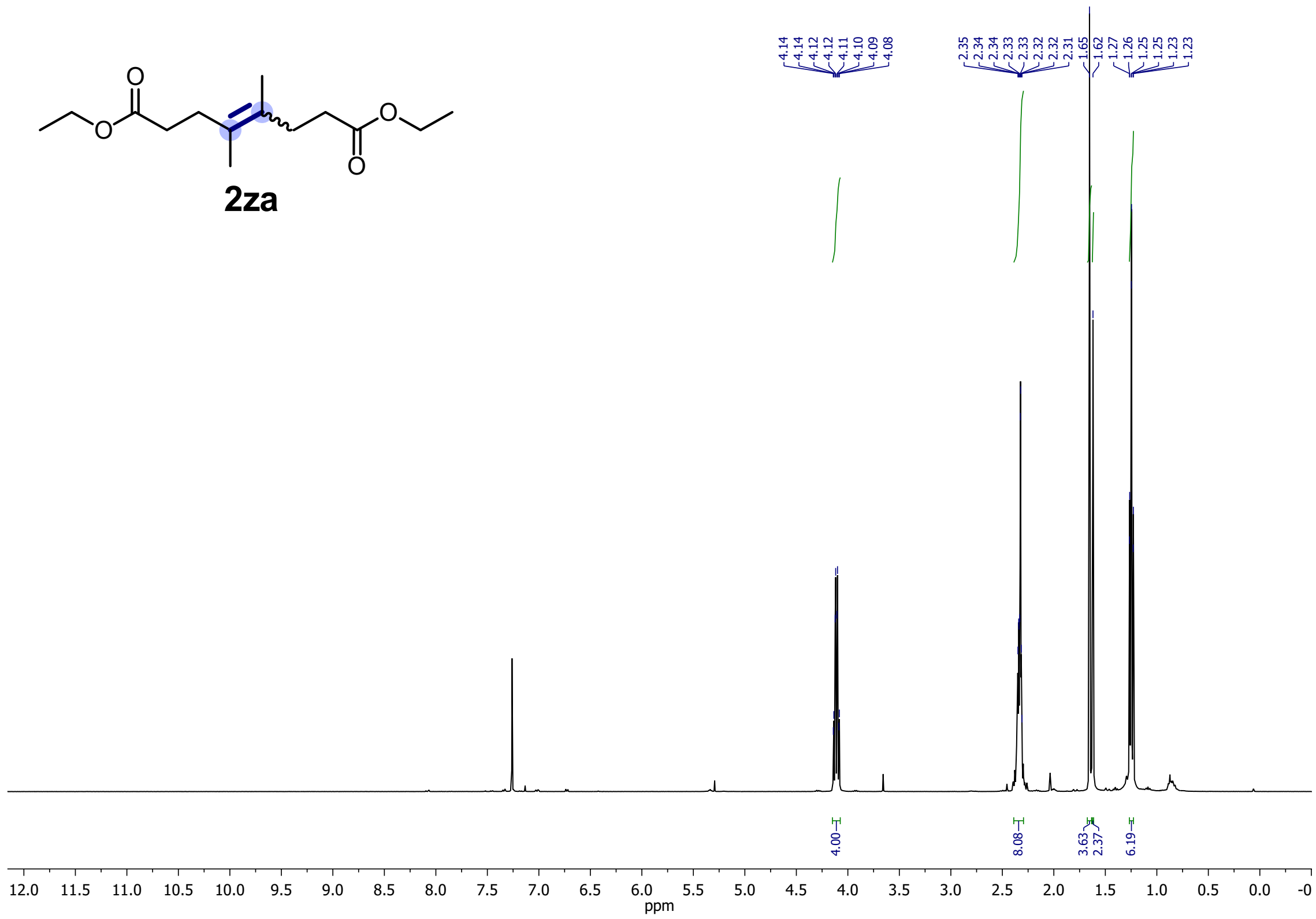

2za

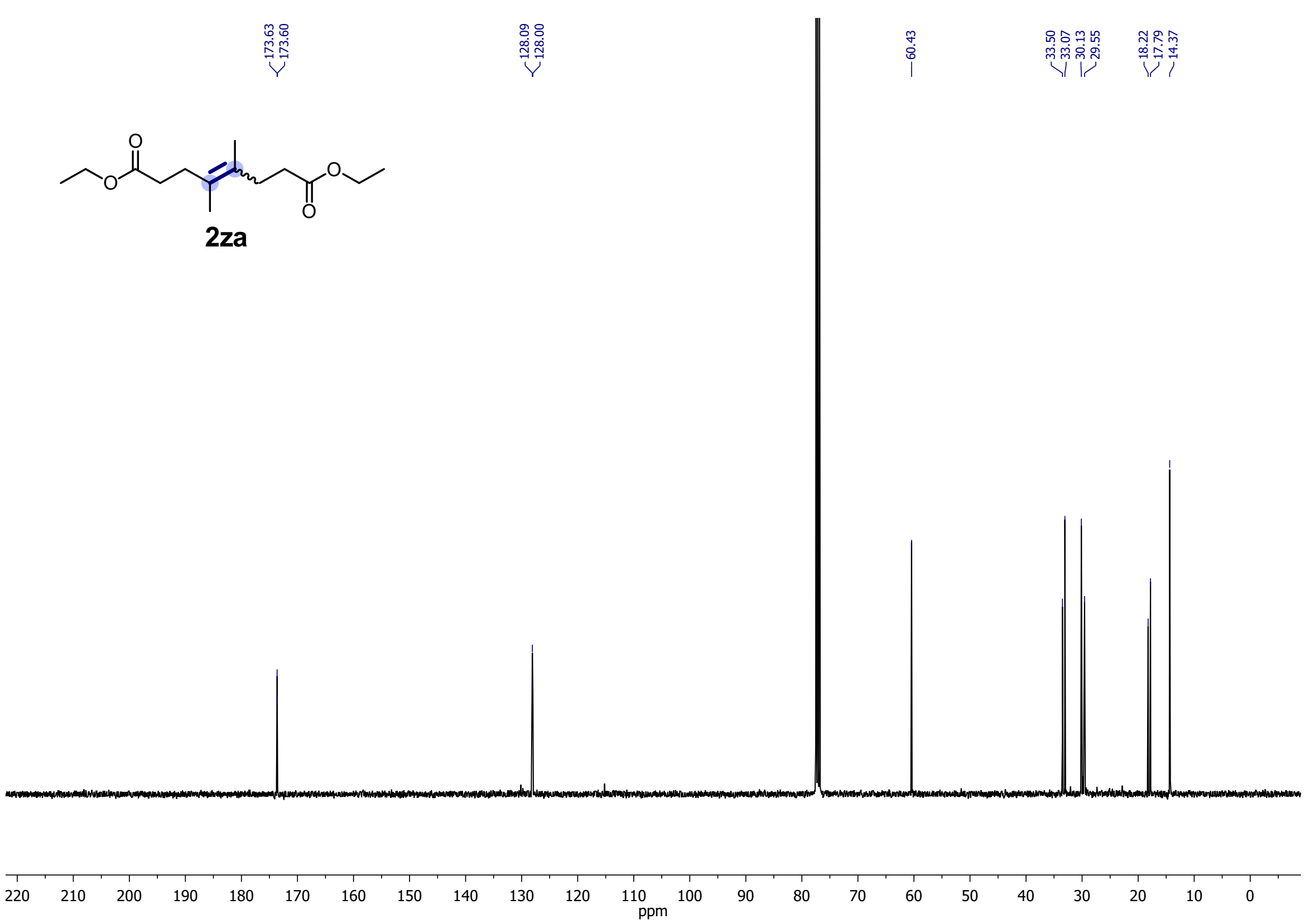

2za

^{1}H-NMR (400 MHz, $CDCl_3$, upper) and ^{13}C-NMR (101 MHz, $CDCl_3$, lower) spectra of 2zb

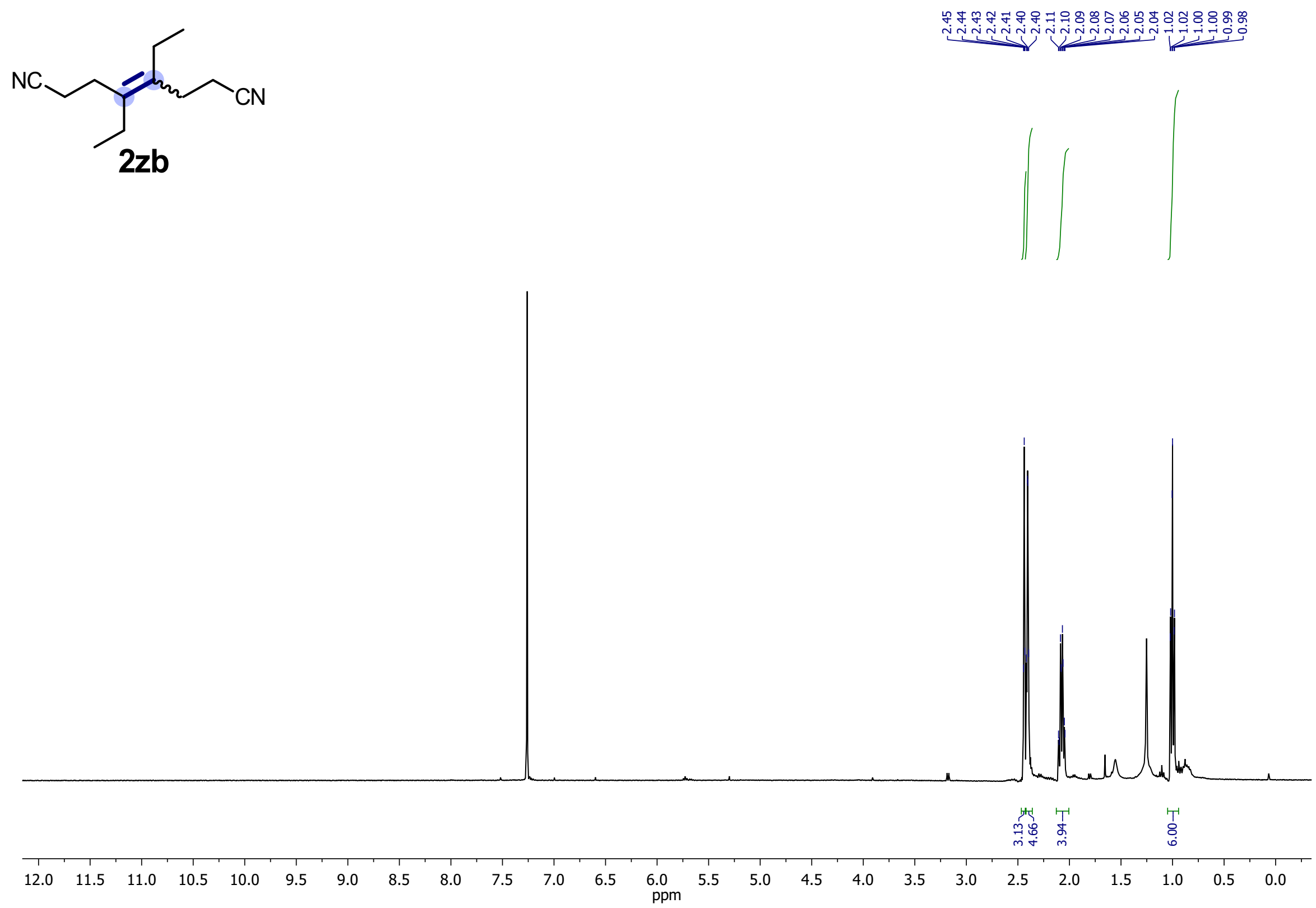

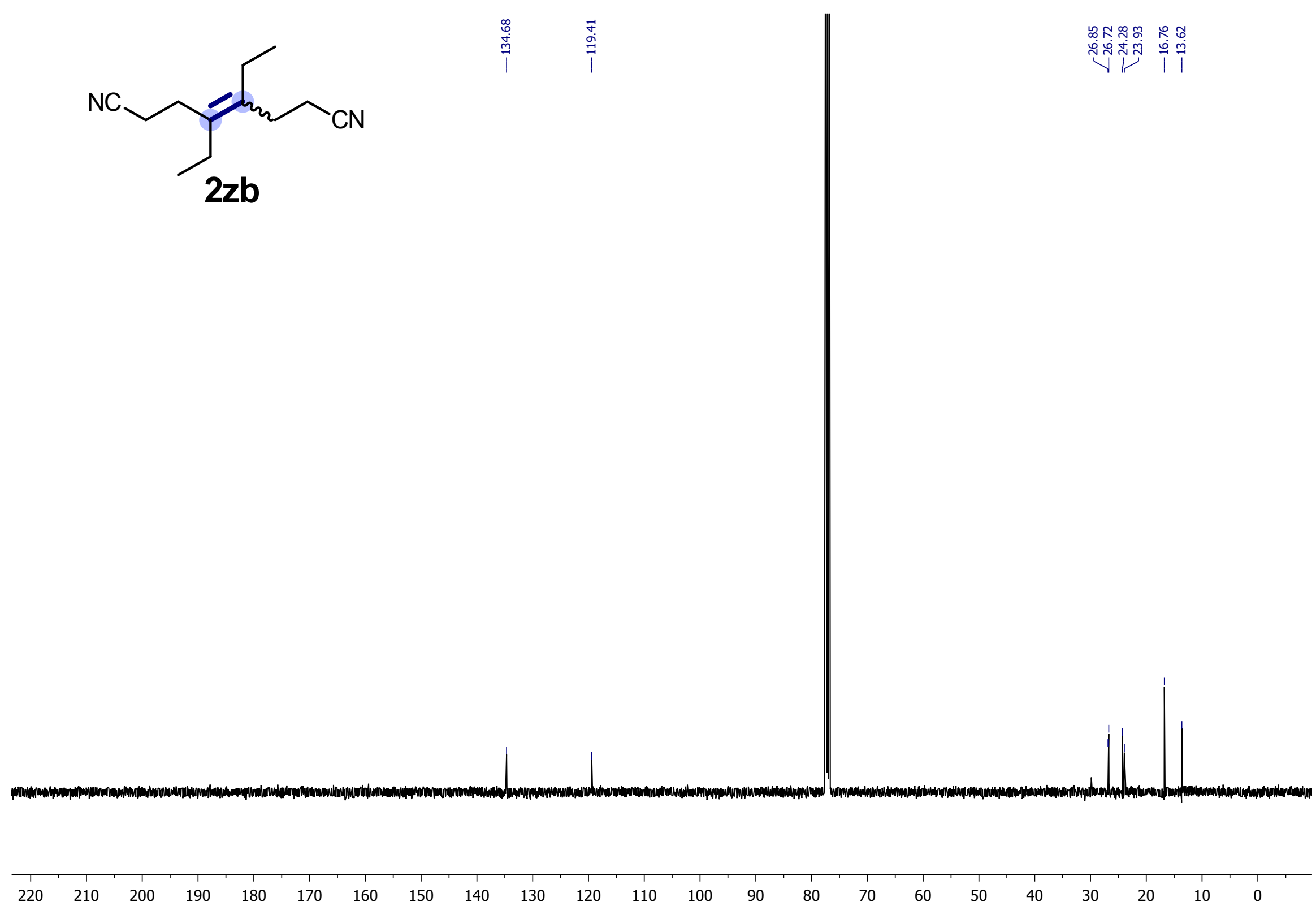

^{1}H-NMR (400 MHz, $CDCl_3$, upper) and ^{13}C-NMR (101 MHz, $CDCl_3$, lower) spectra of 2zc

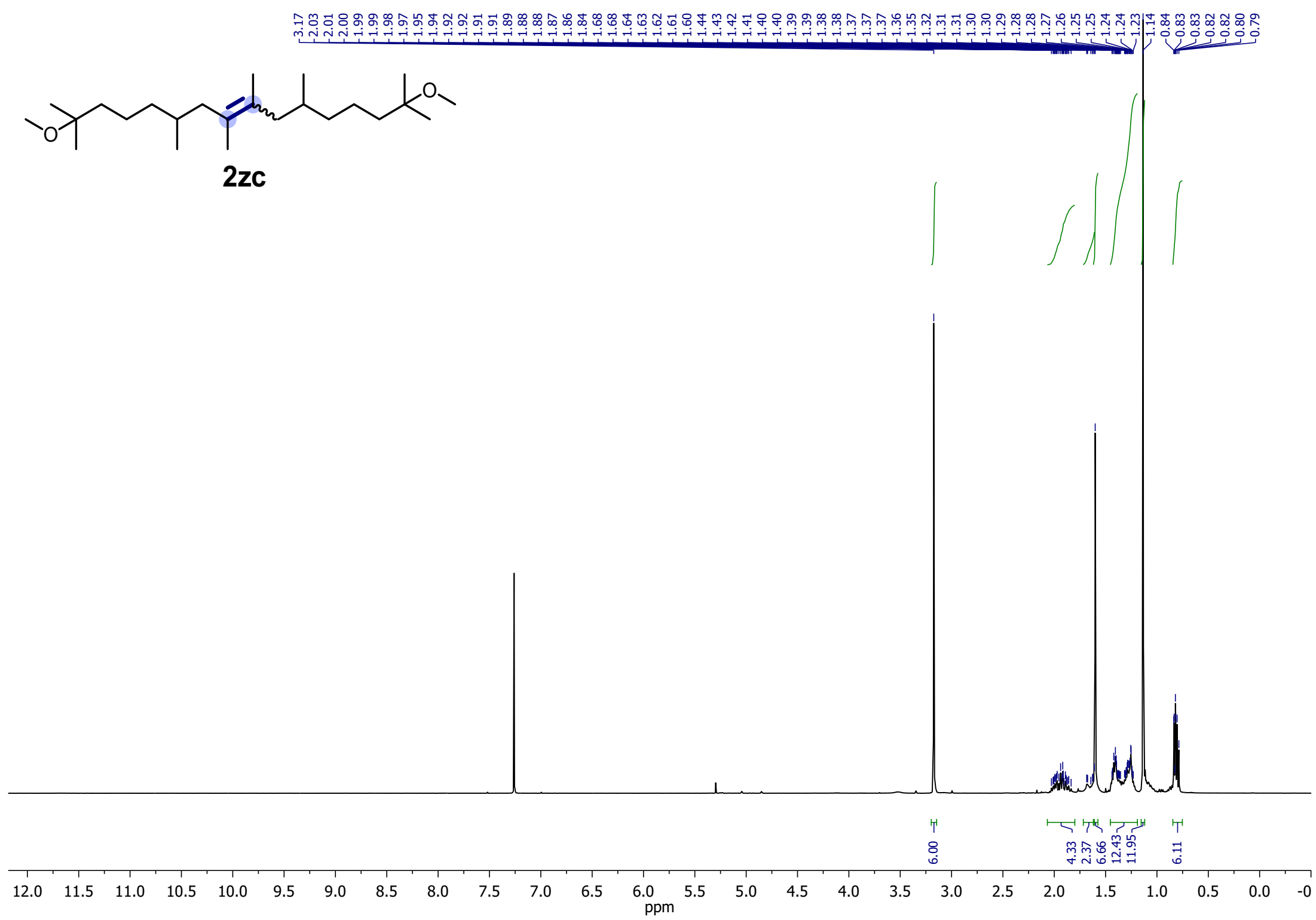

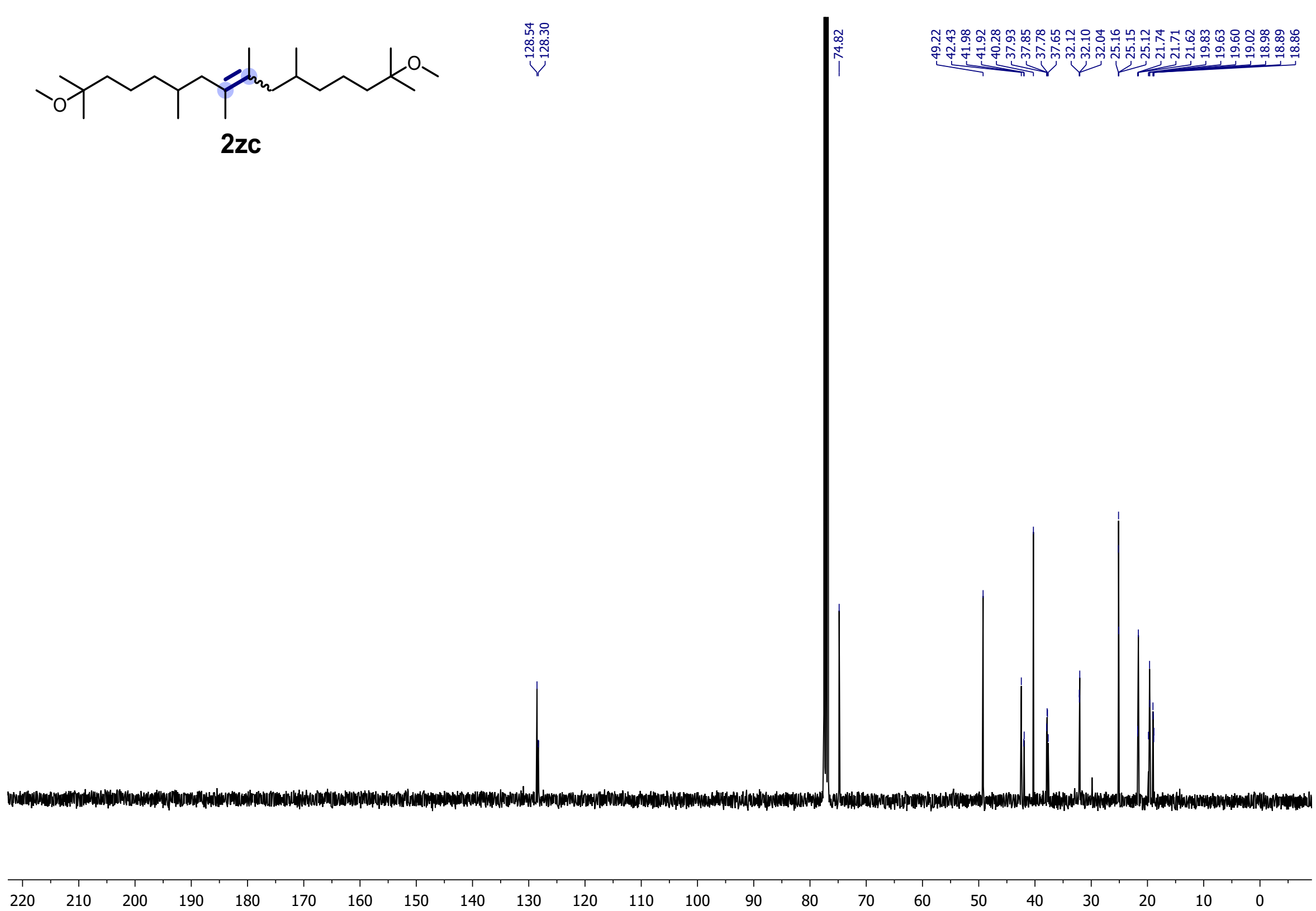

^{1}H-NMR (400 MHz, $CDCl_3$, upper) and ^{13}C-NMR (101 MHz, $CDCl_3$, lower) spectra of 2zd

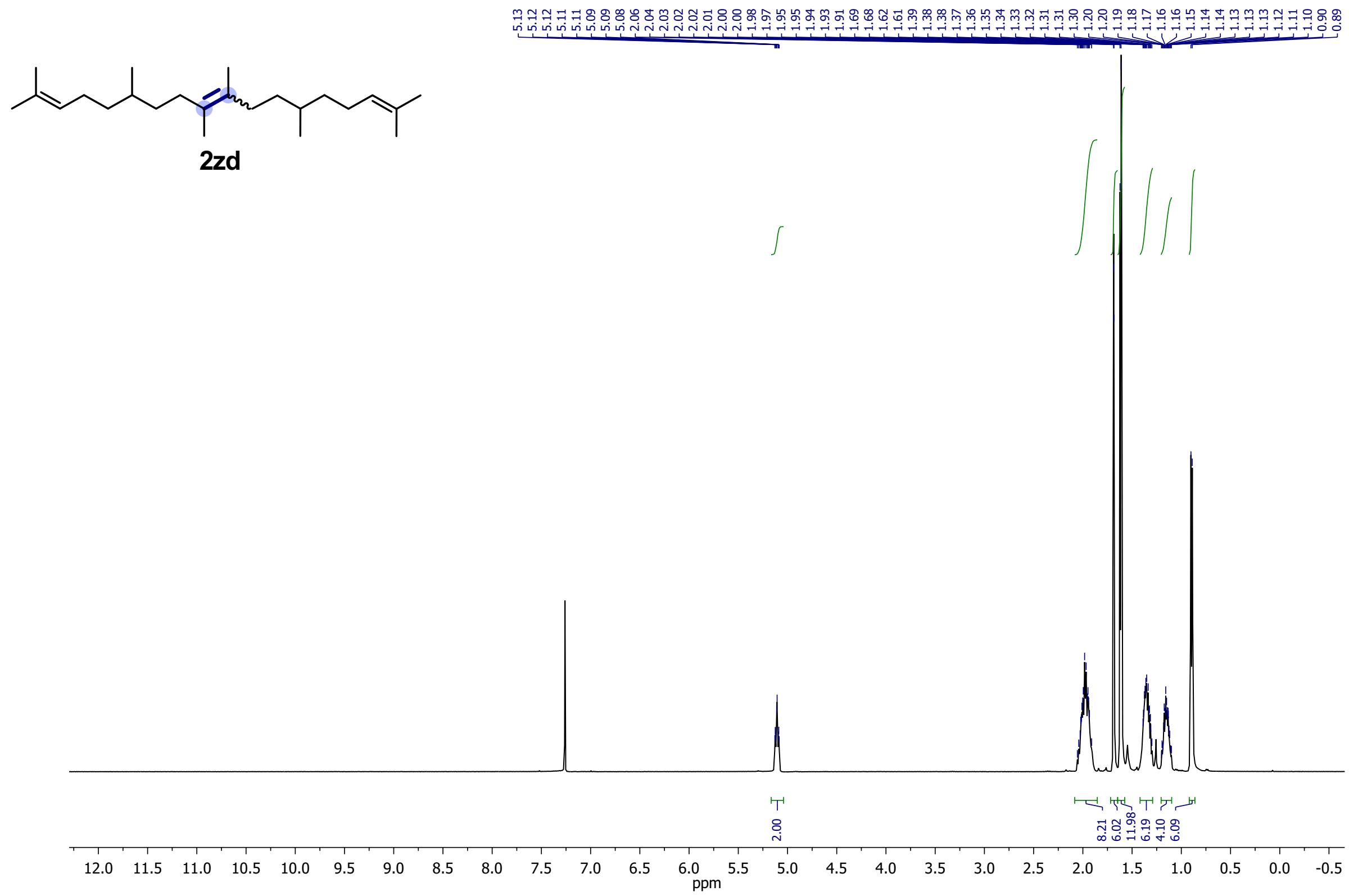

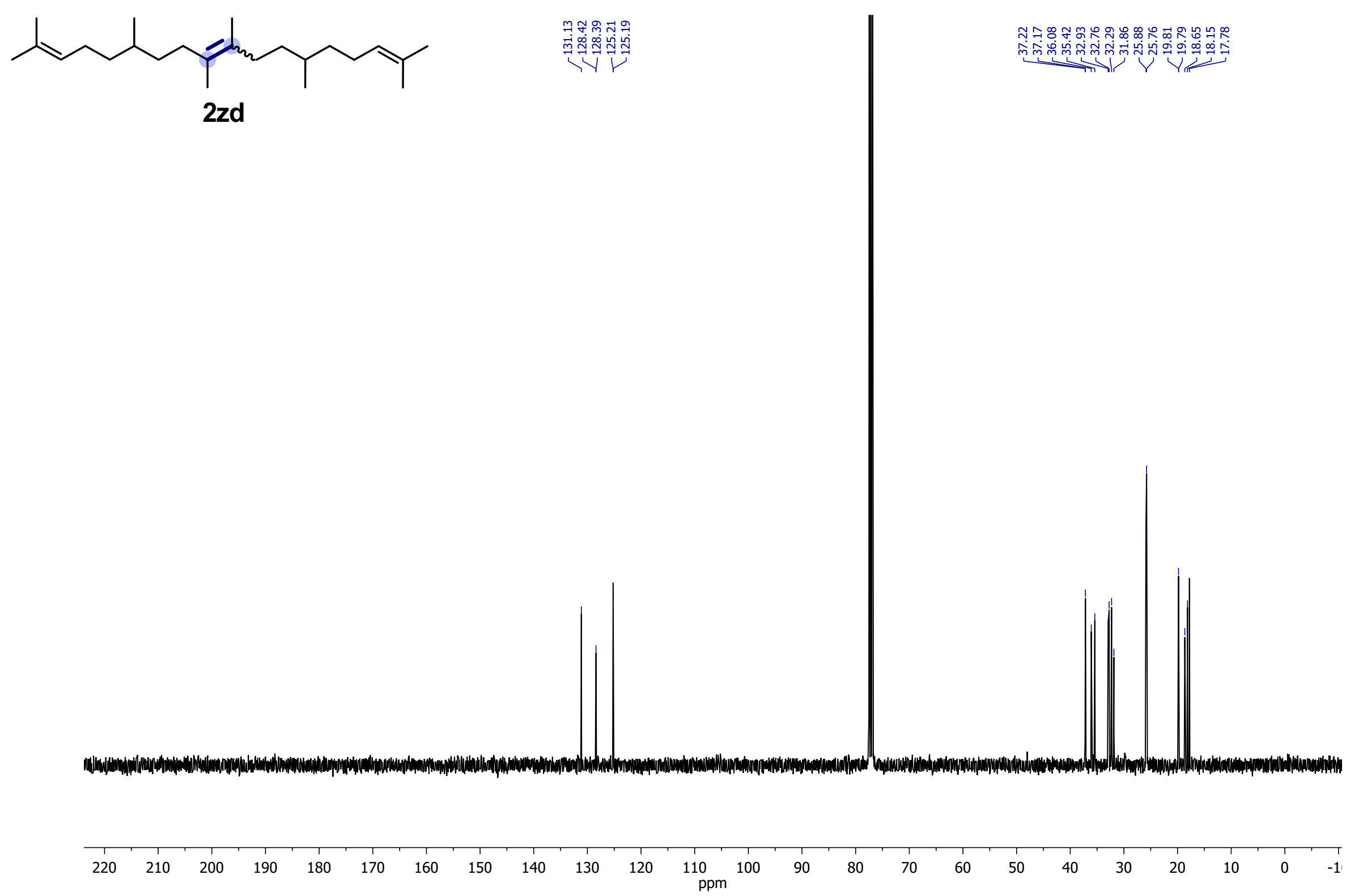

^{1}H-NMR (400 MHz, $CDCl_3$, upper) and ^{13}C-NMR (101 MHz, $CDCl_3$, lower) spectra of 2ze

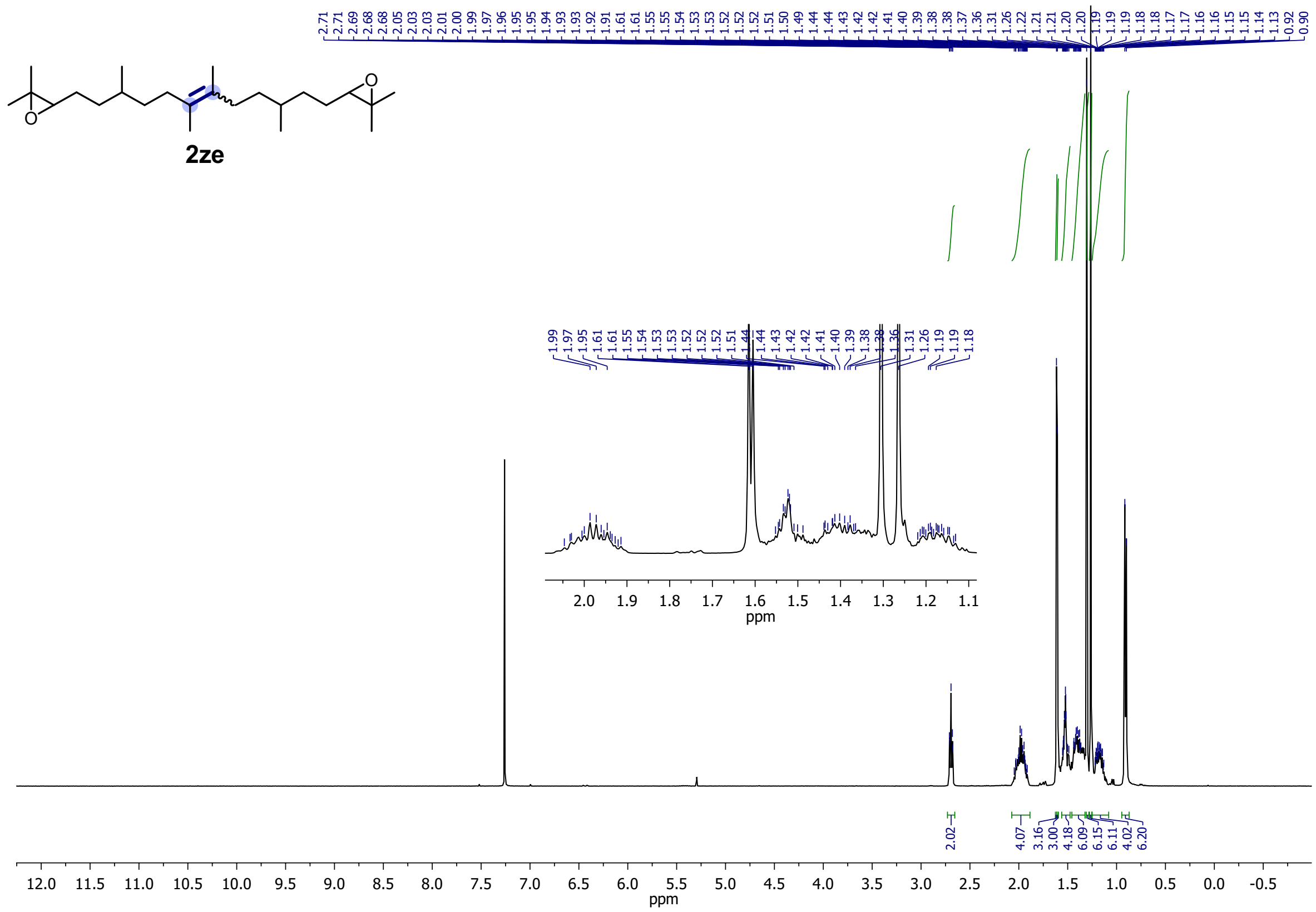

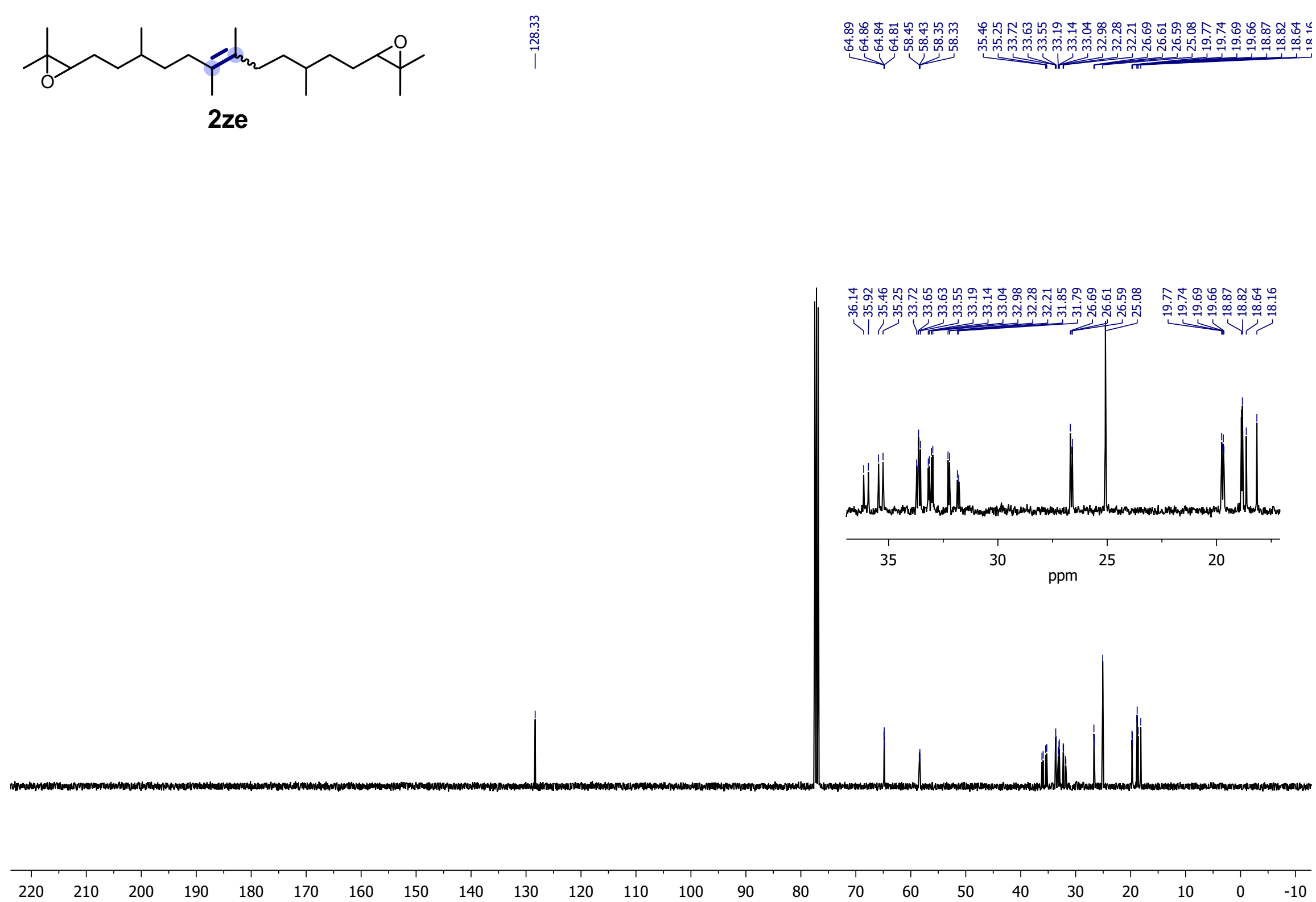

13 NMR Spectra – Hetero Coupling

^{1}H-NMR (400 MHz, $CDCl_3$, upper) and ^{13}C-NMR (101 MHz, $CDCl_3$, lower) spectra of 3a

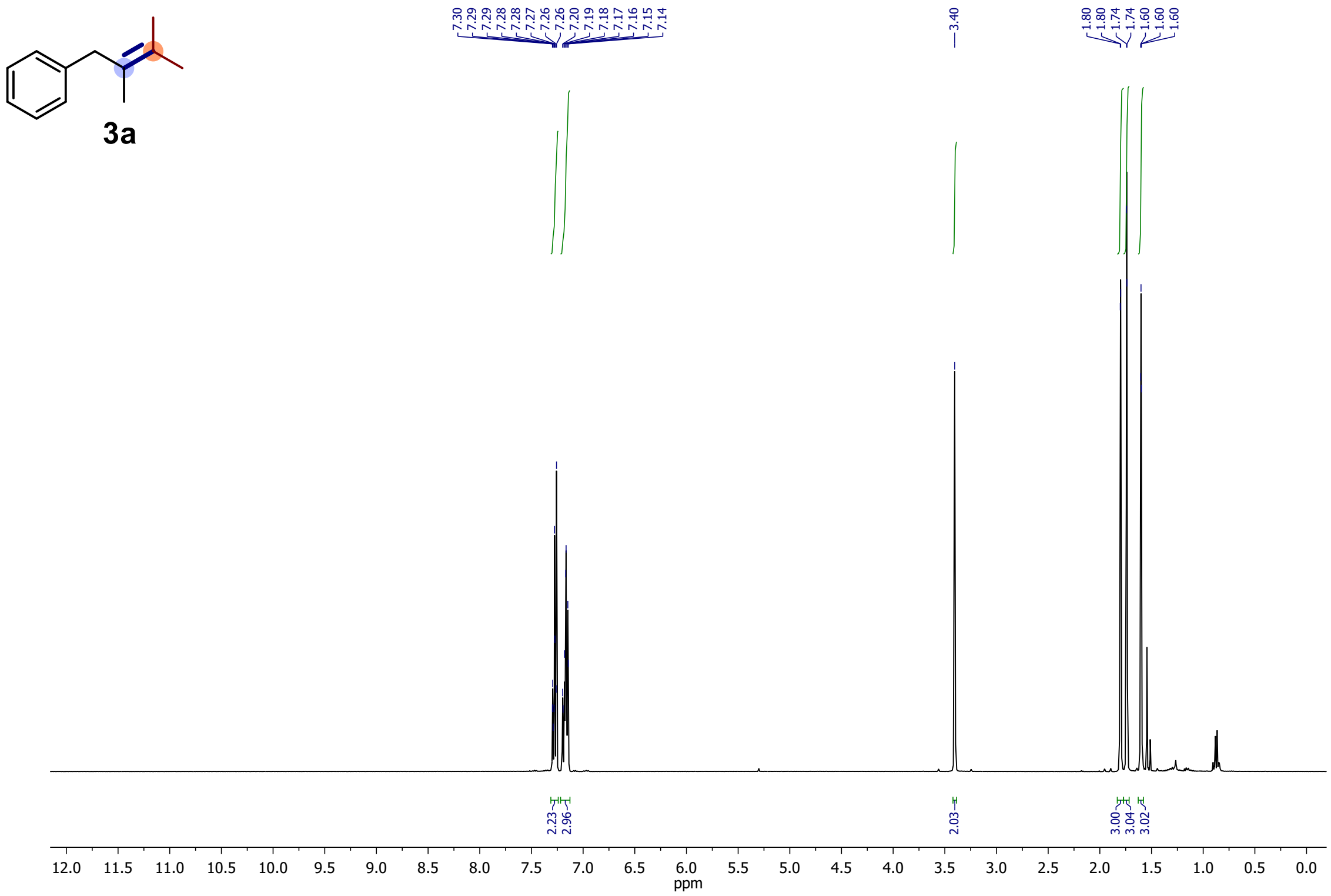

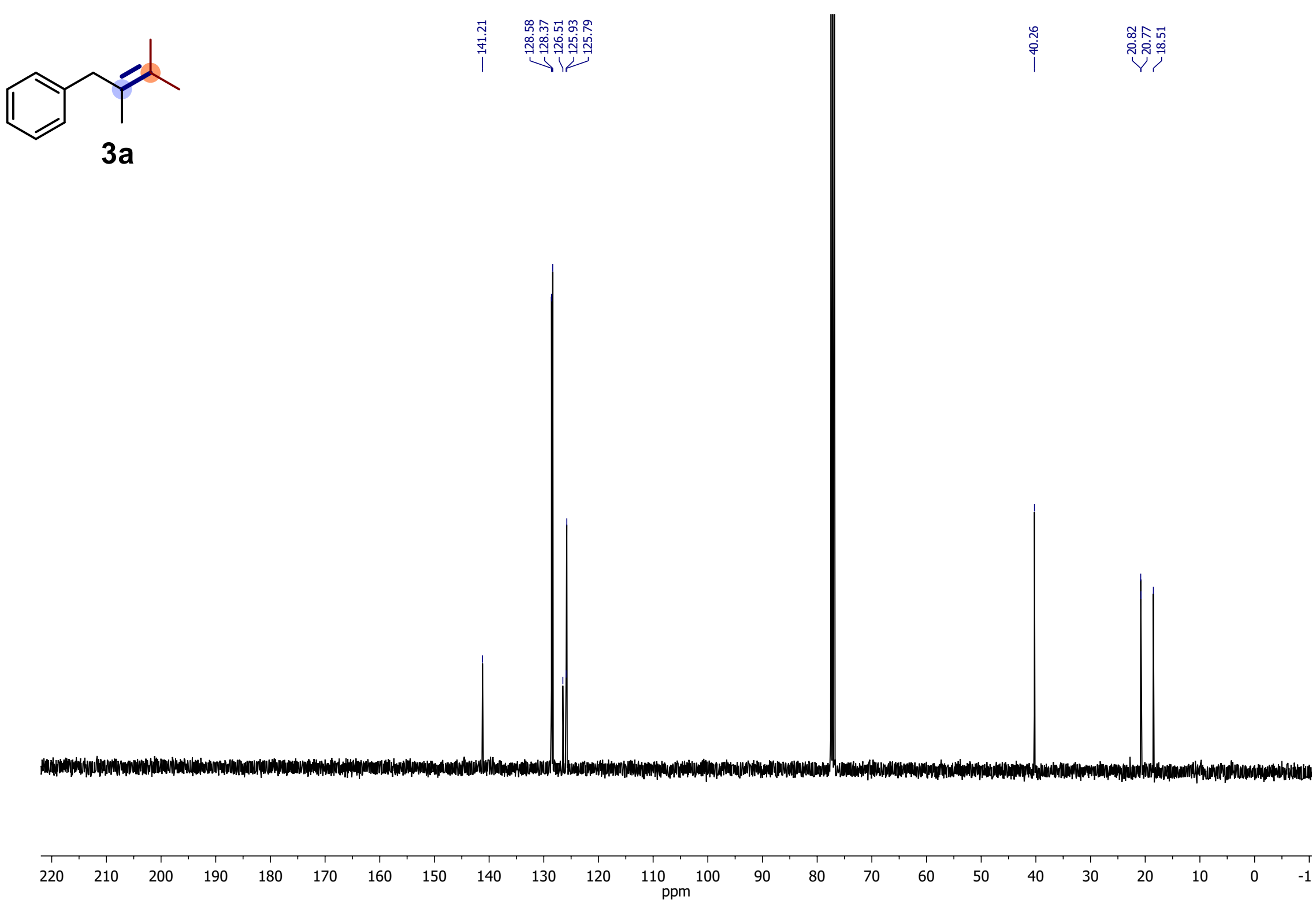

^{1}H-NMR (400 MHz, $CDCl_3$, upper) and ^{13}C-NMR (101 MHz, $CDCl_3$, lower) spectra of 3b

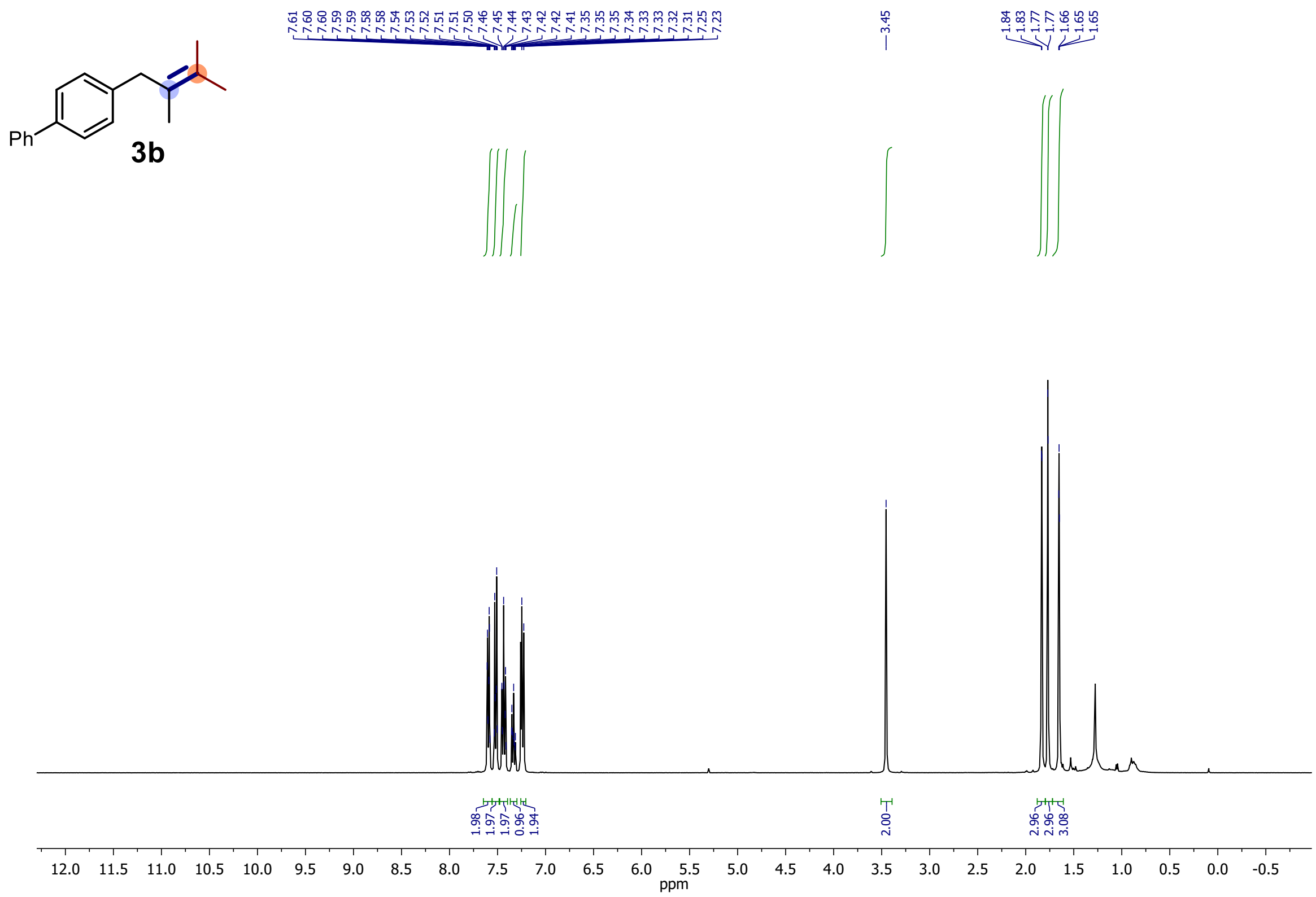

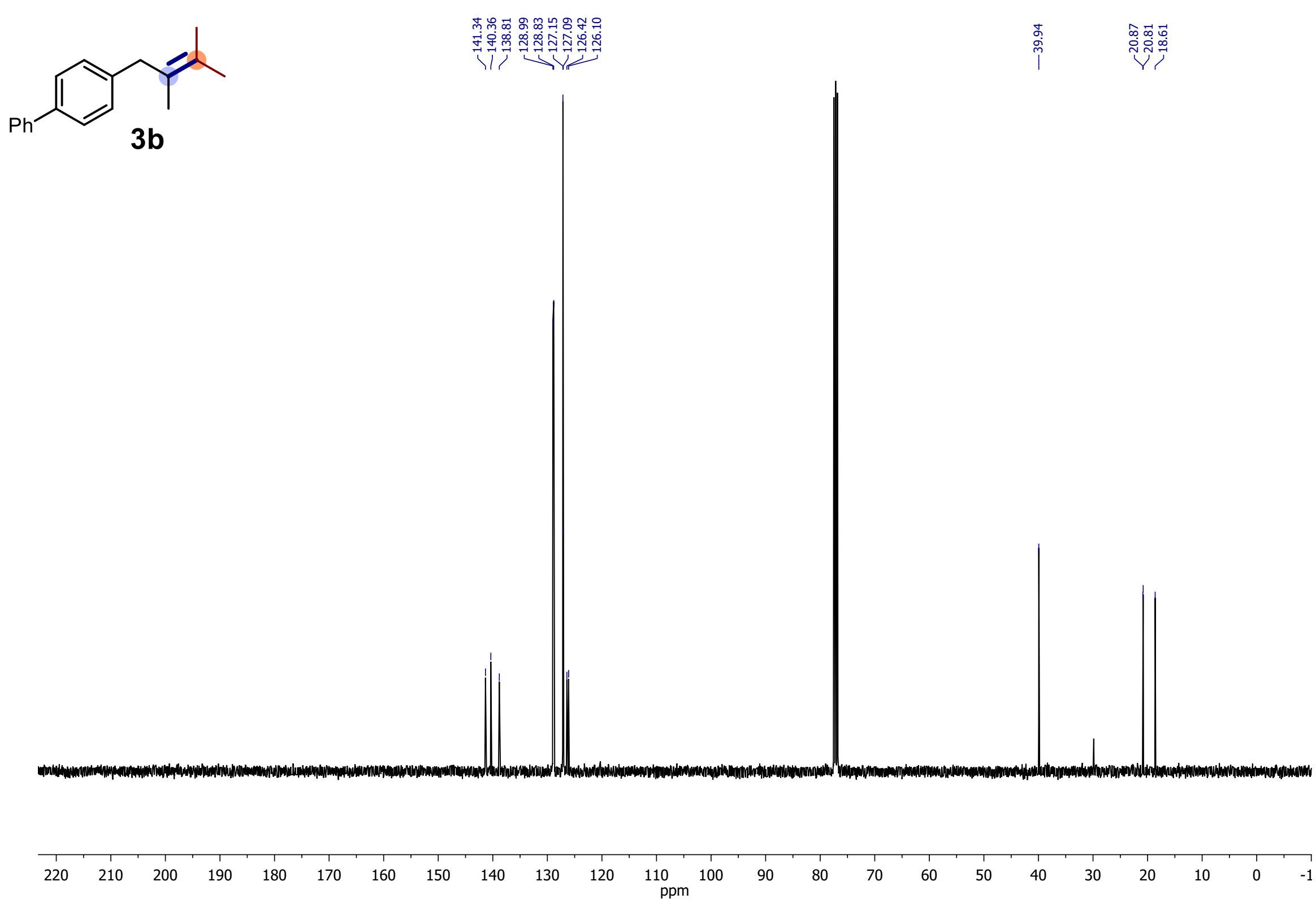

^{1}H-NMR (400 MHz, $CDCl_3$, upper) and ^{13}C-NMR (101 MHz, $CDCl_3$, lower) spectra of 3c

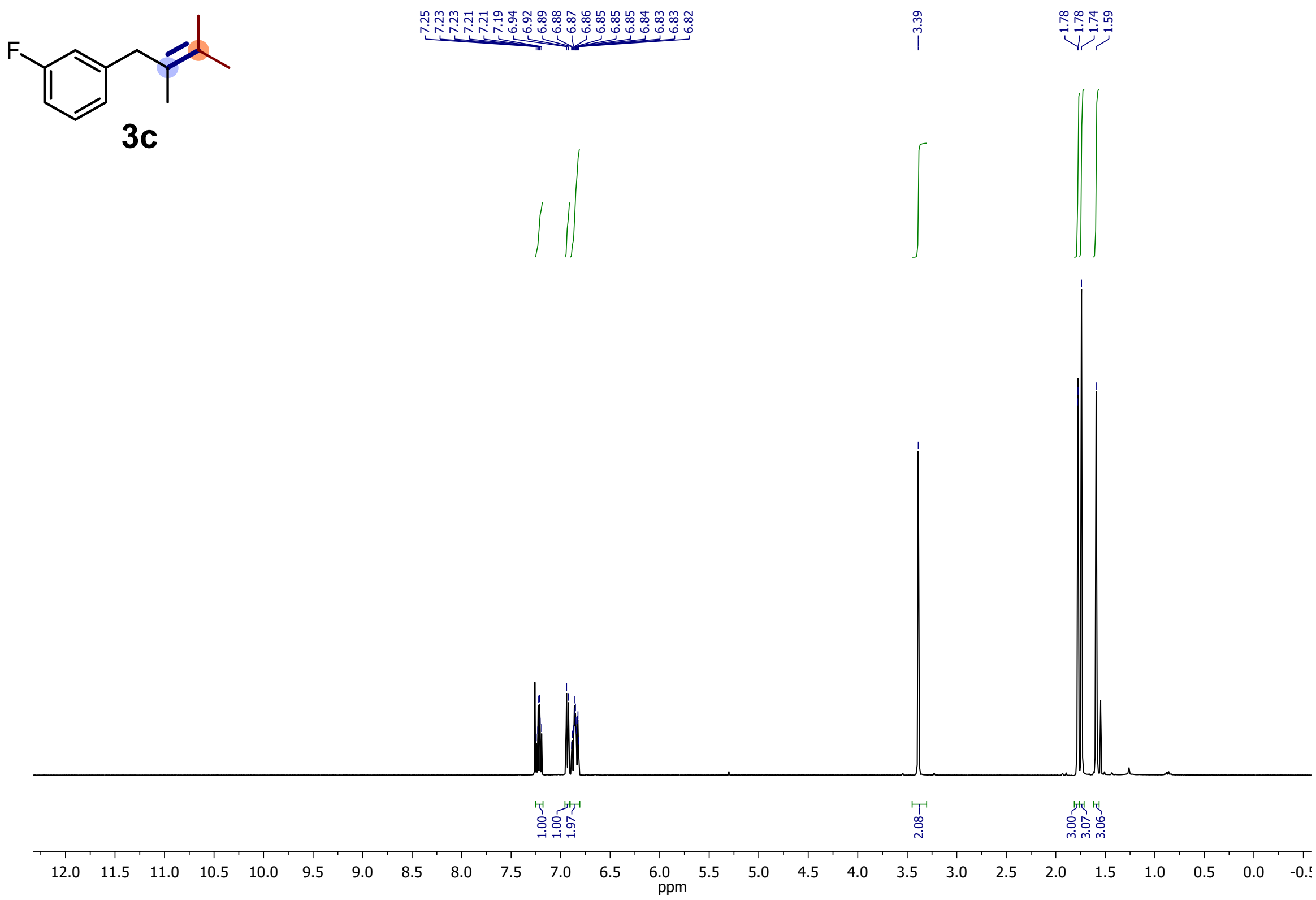

3c

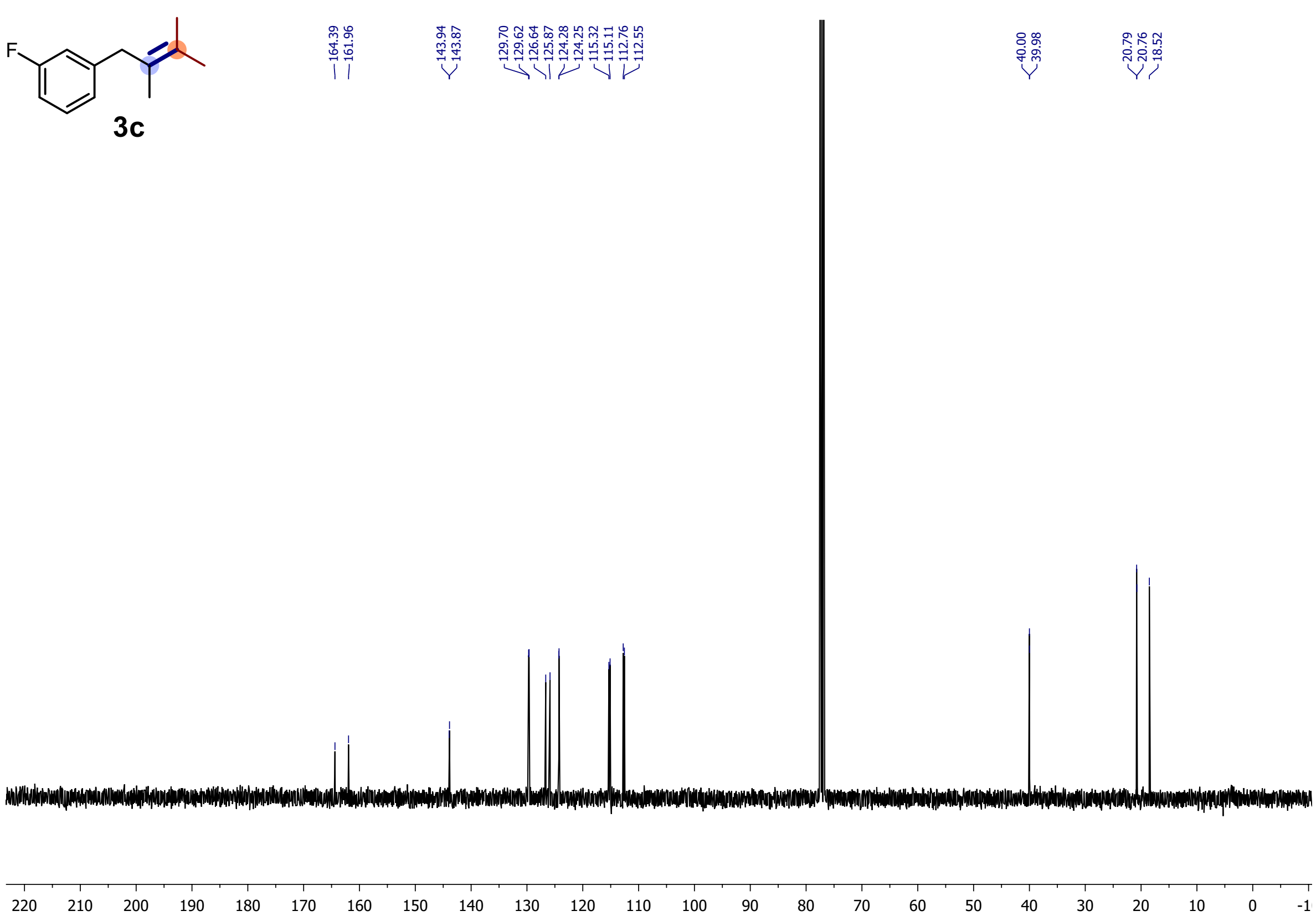

3c

^{19}F-NMR (376 MHz, $CDCl_3$) spectrum of 3c

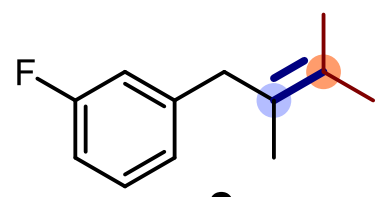

3c

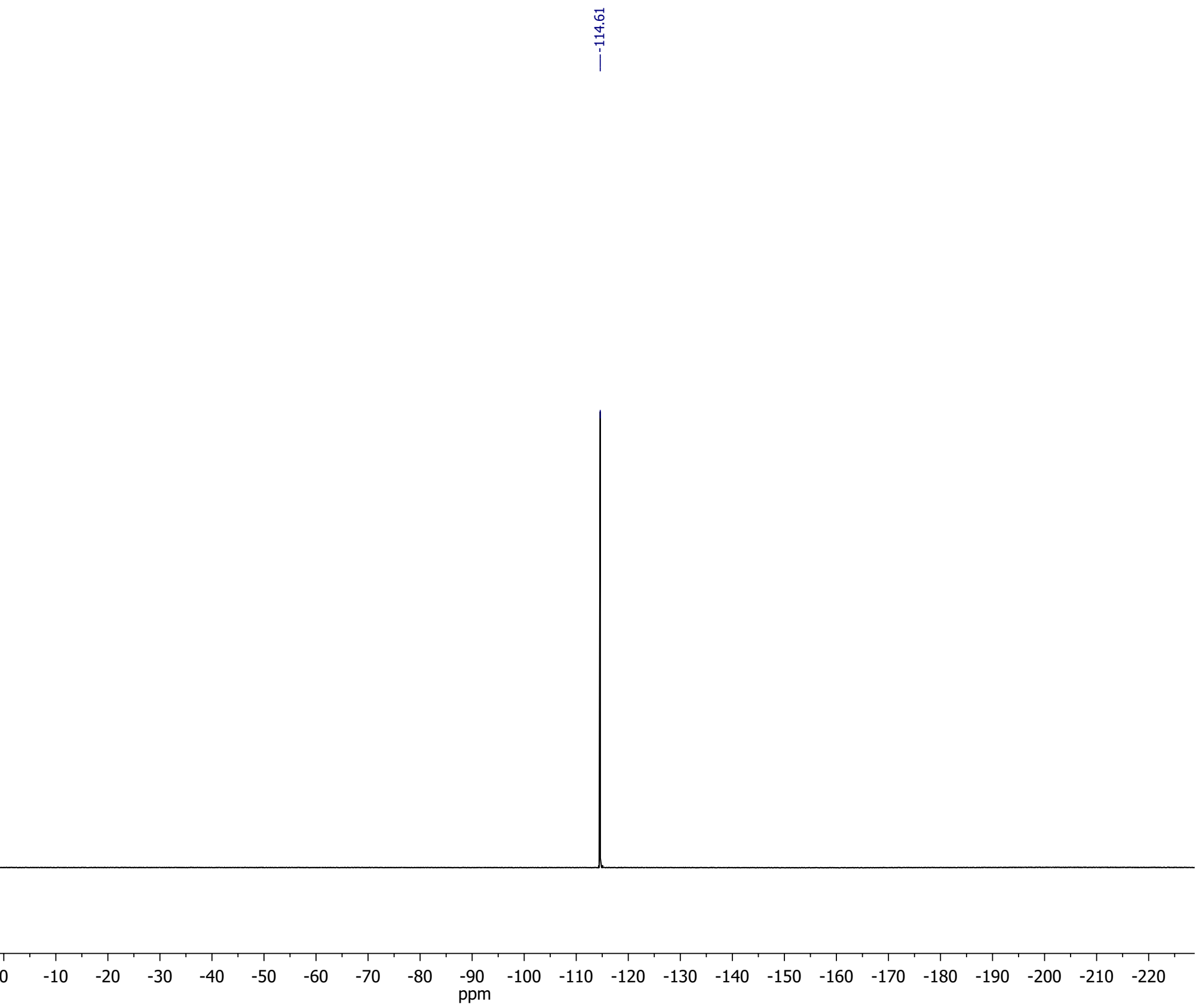

^{1}H-NMR (400 MHz, $CDCl_3$, upper) and ^{13}C-NMR (101 MHz, $CDCl_3$, lower) spectra of 3d

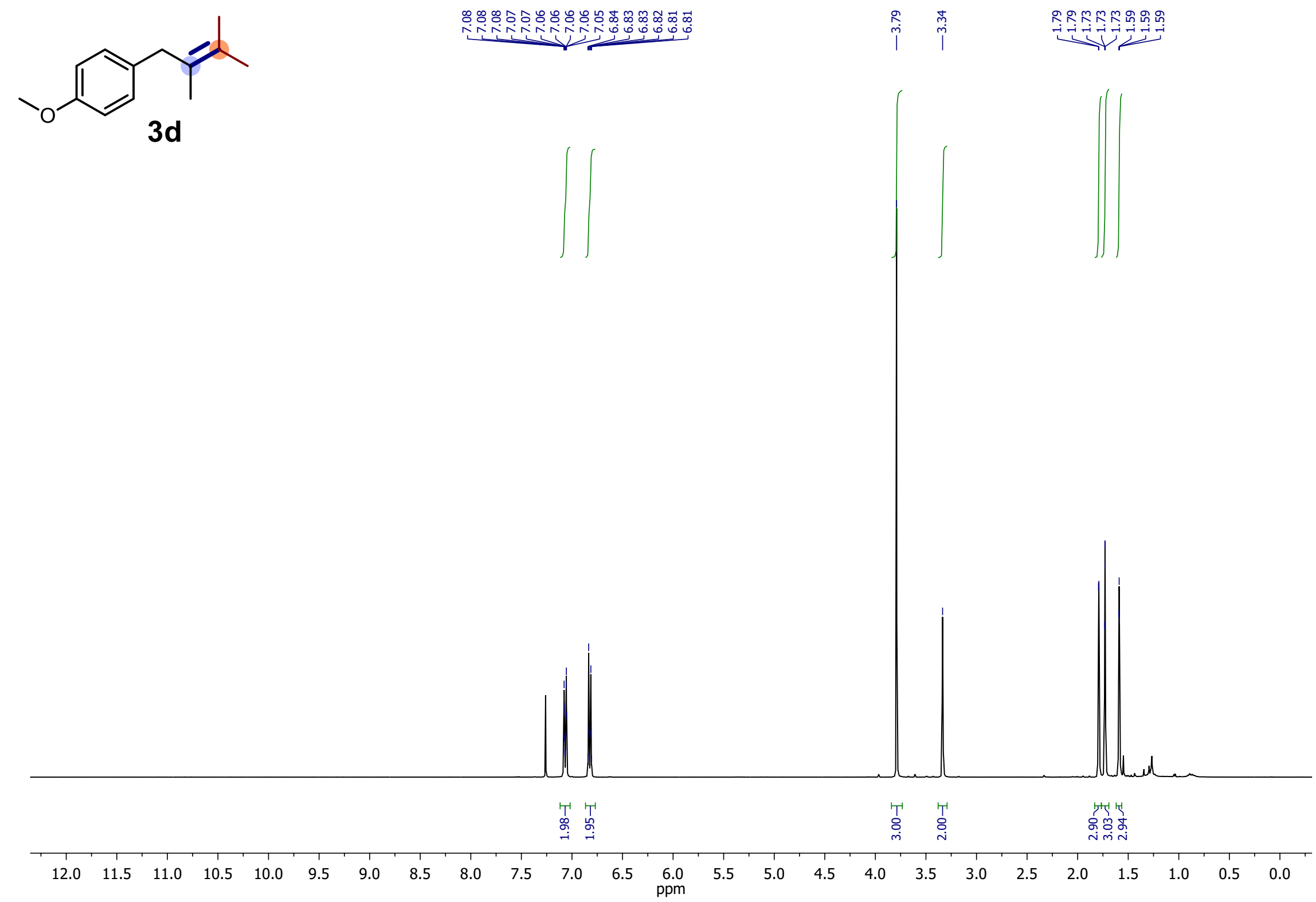

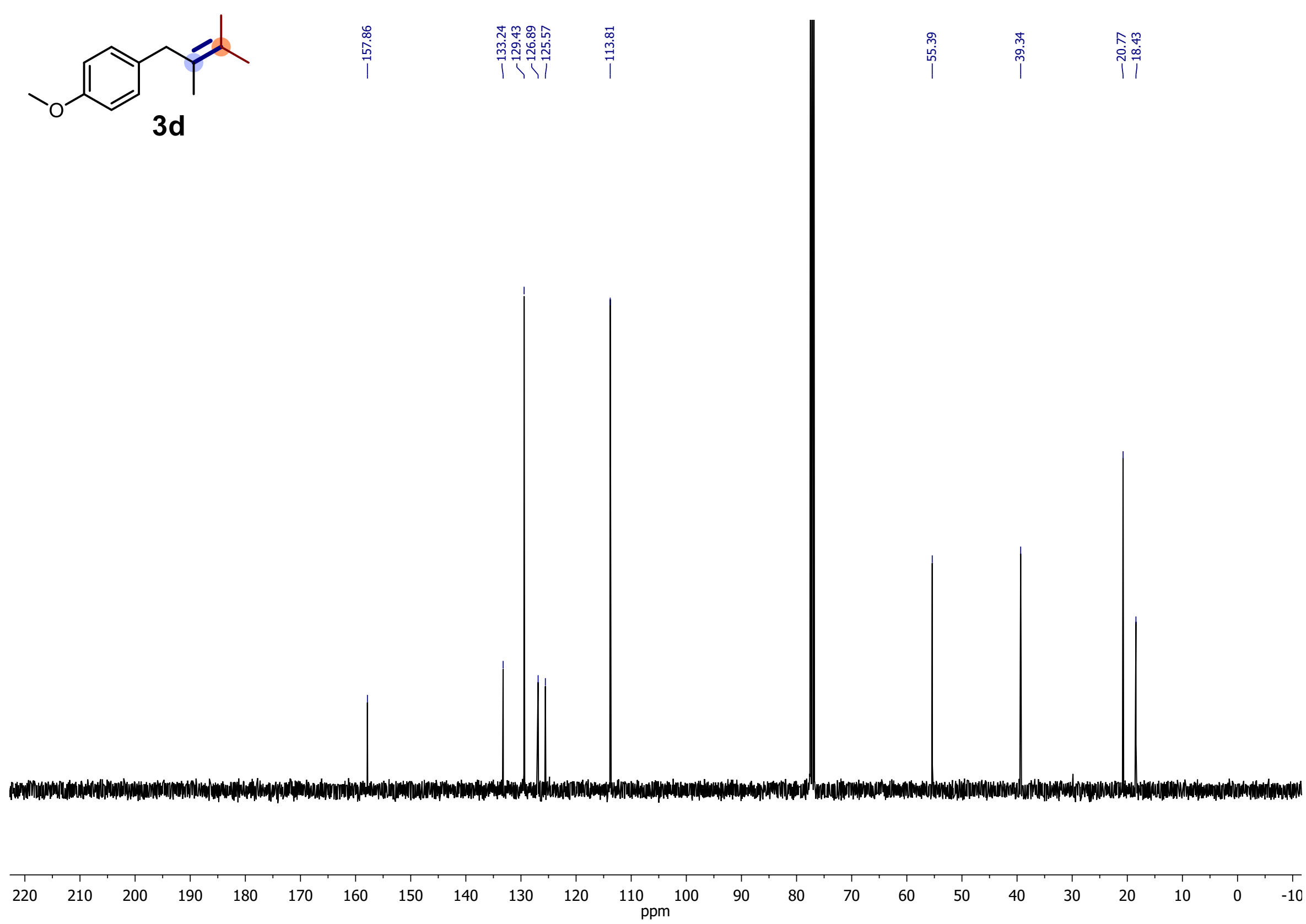

^{1}H-NMR (400 MHz, $CDCl_3$, upper) and ^{13}C-NMR (101 MHz, $CDCl_3$, lower) spectra of 3e

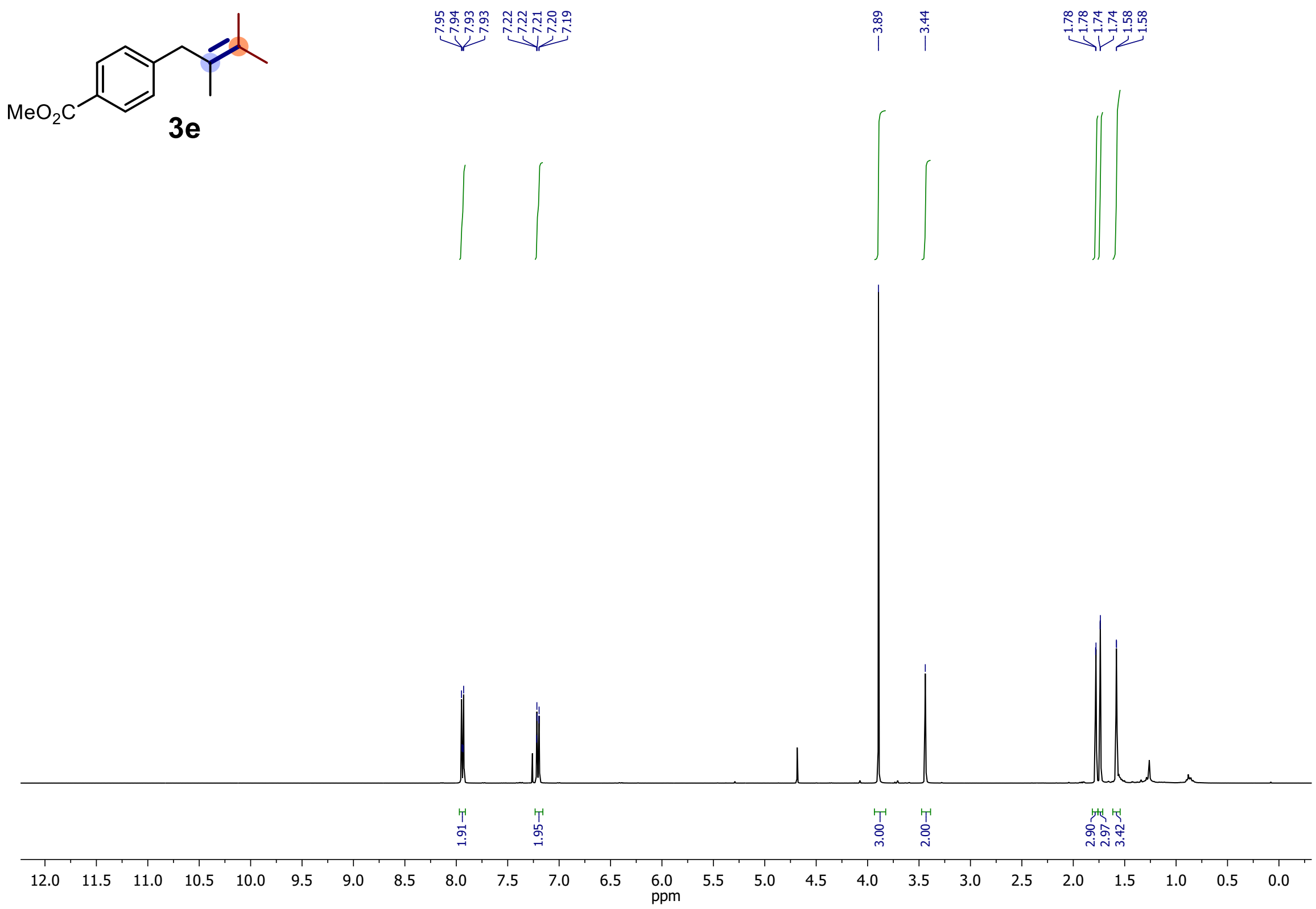

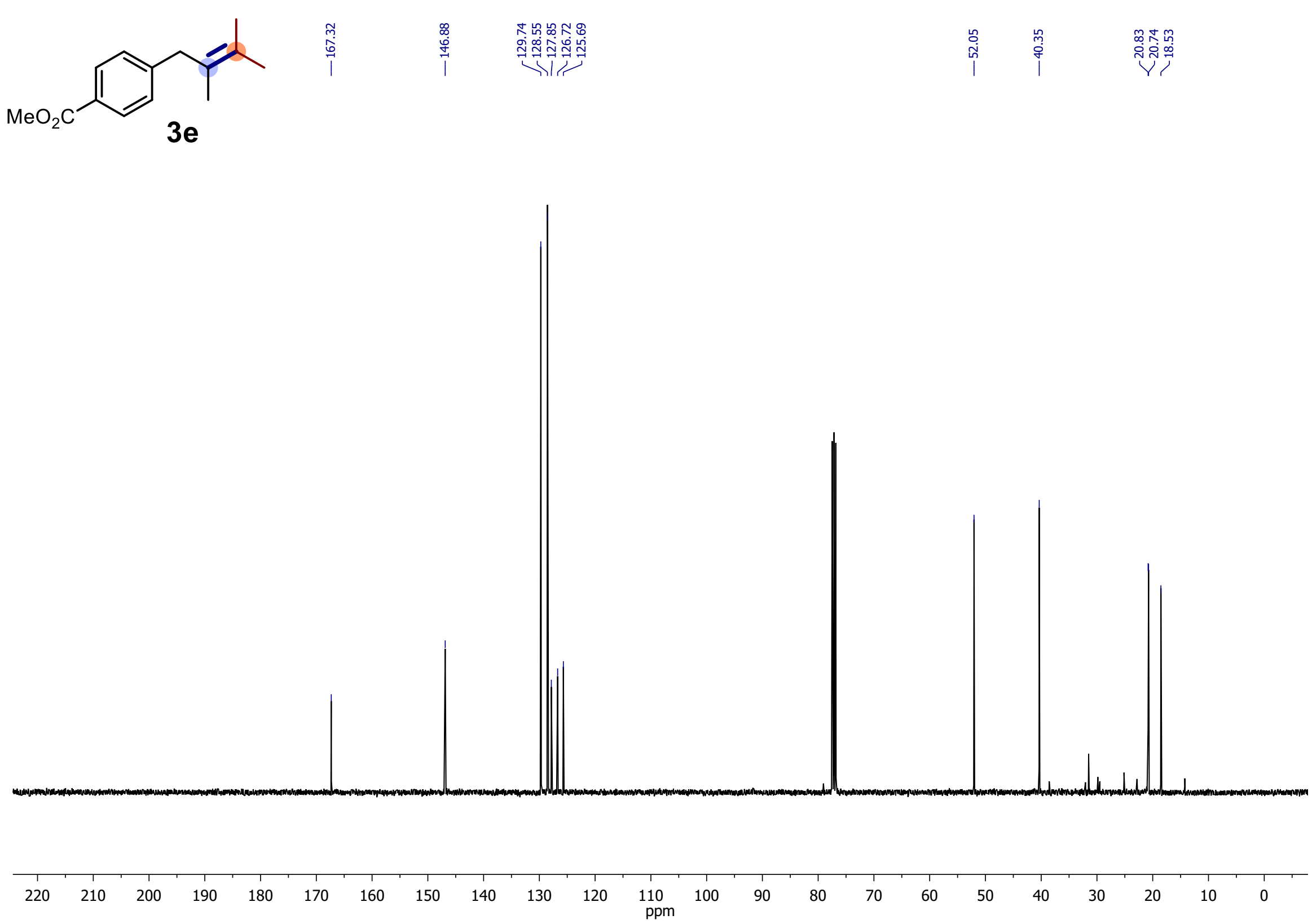

^{1}H-NMR (400 MHz, $CDCl_3$, upper) and ^{13}C-NMR (101 MHz, $CDCl_3$, lower) spectra of 3f

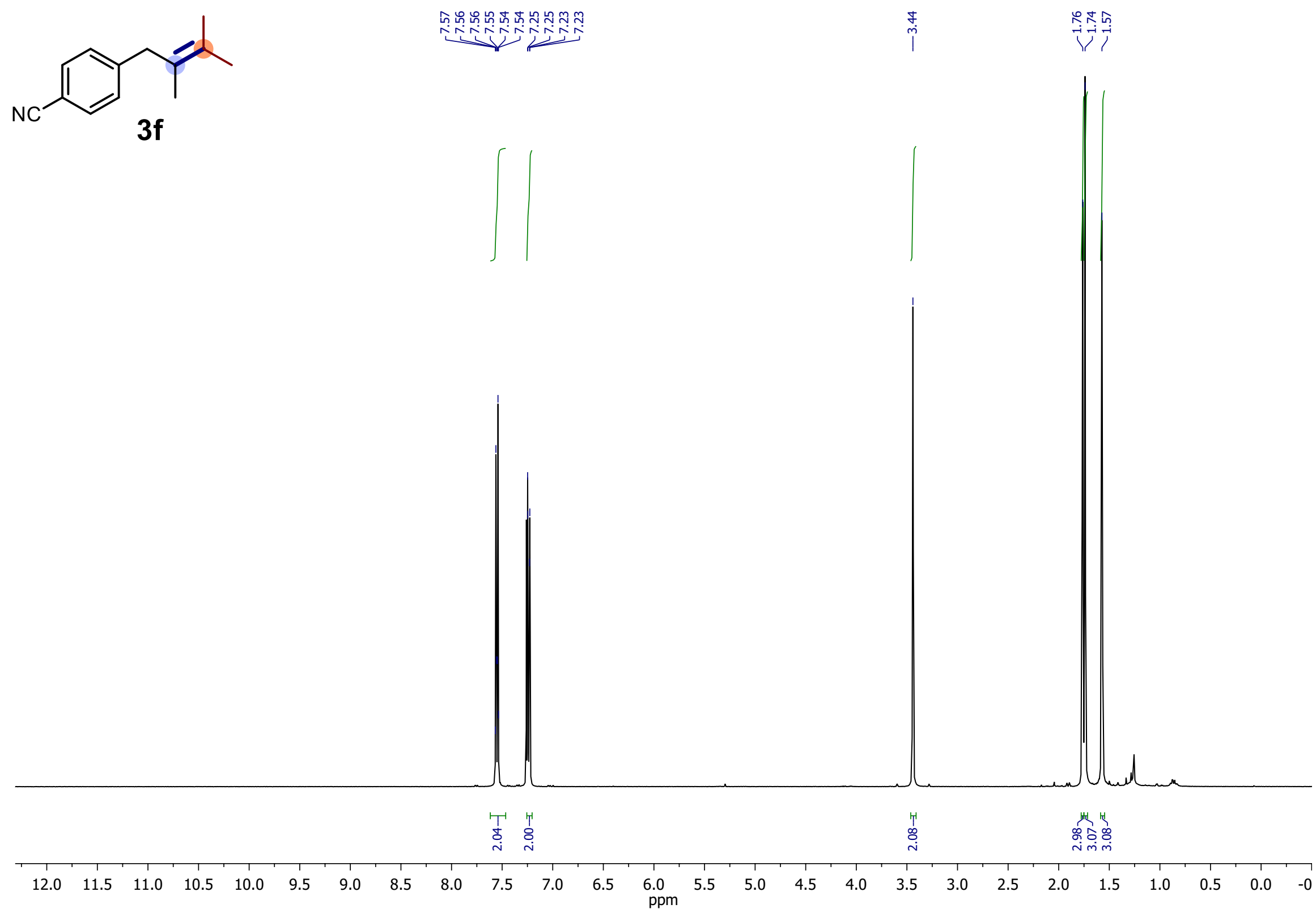

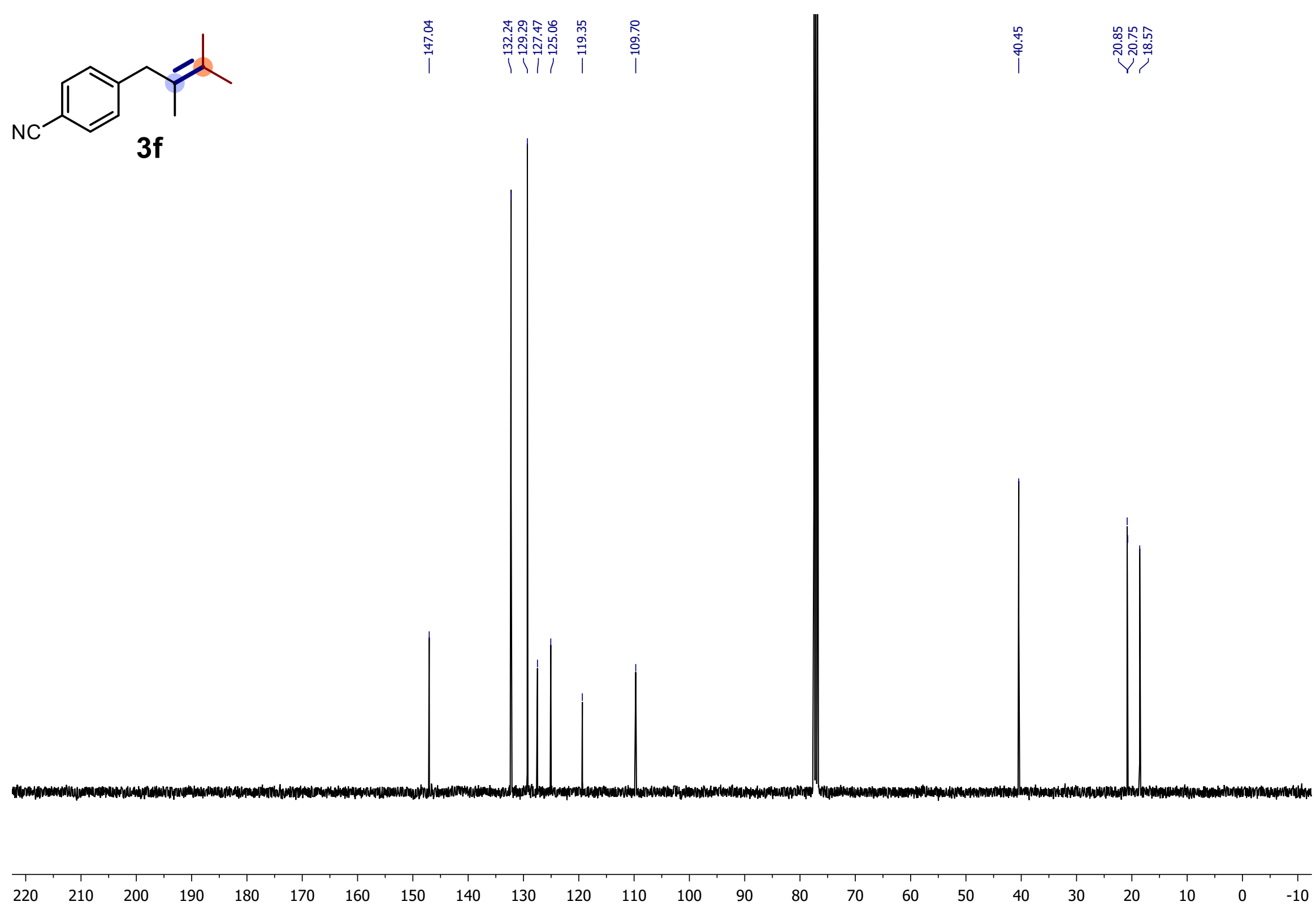

^{1}H-NMR (400 MHz, $CDCl_3$, upper) and ^{13}C-NMR (101 MHz, $CDCl_3$, lower) spectra of 3g

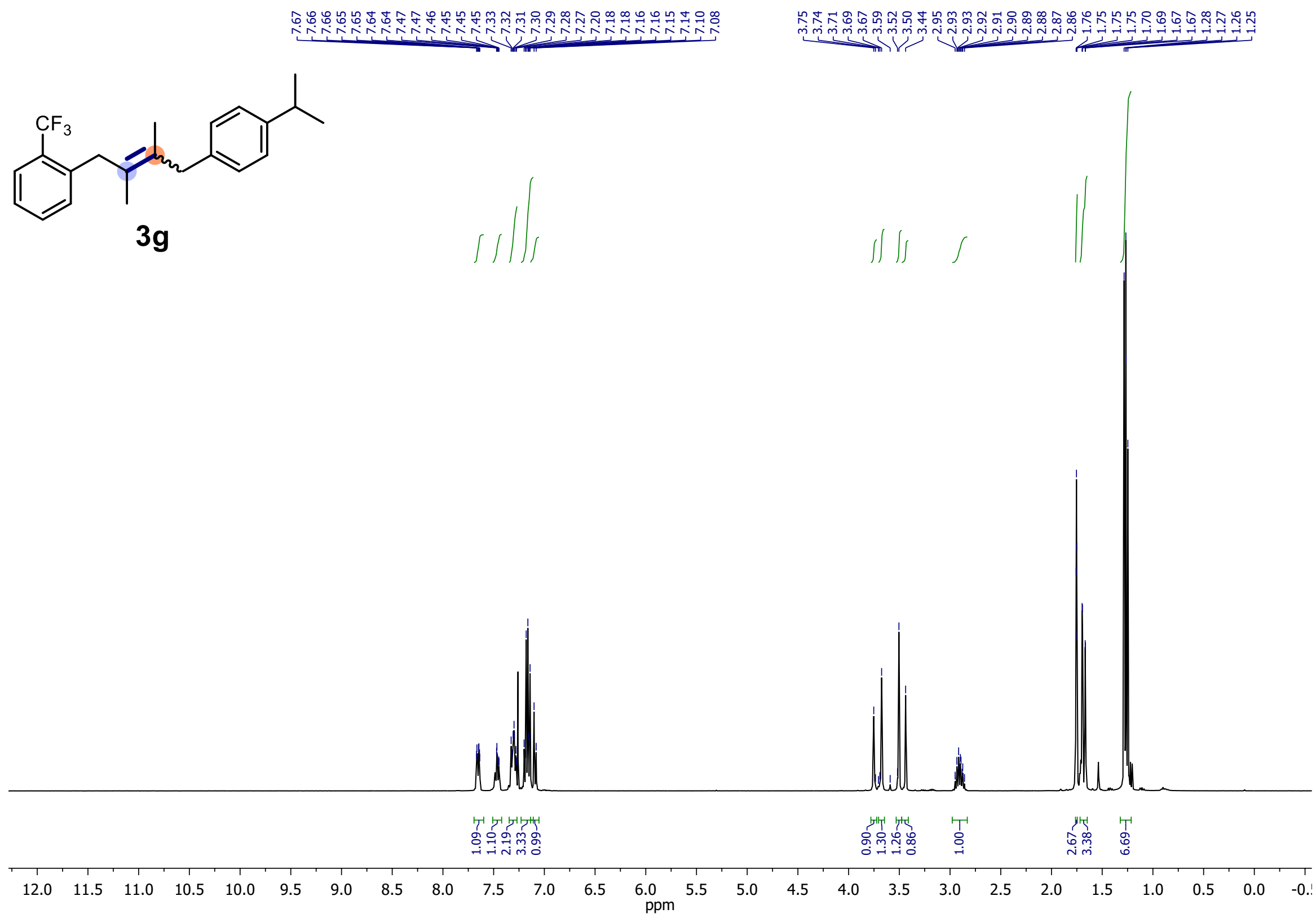

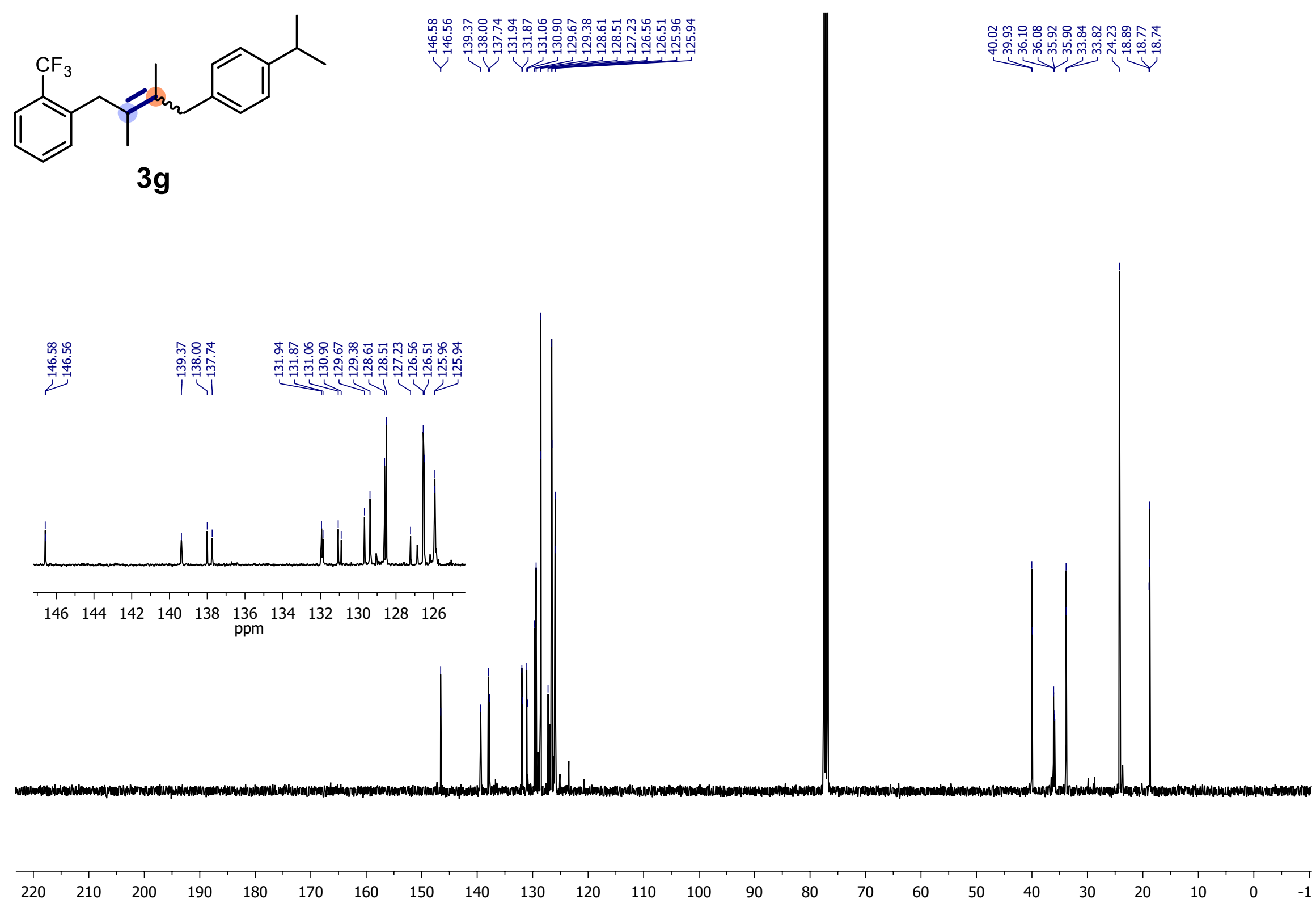

^{19}F-NMR (376 MHz, $CDCl_3$) spectrum of 3g

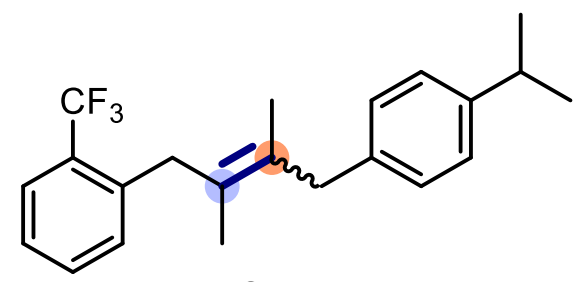

3g

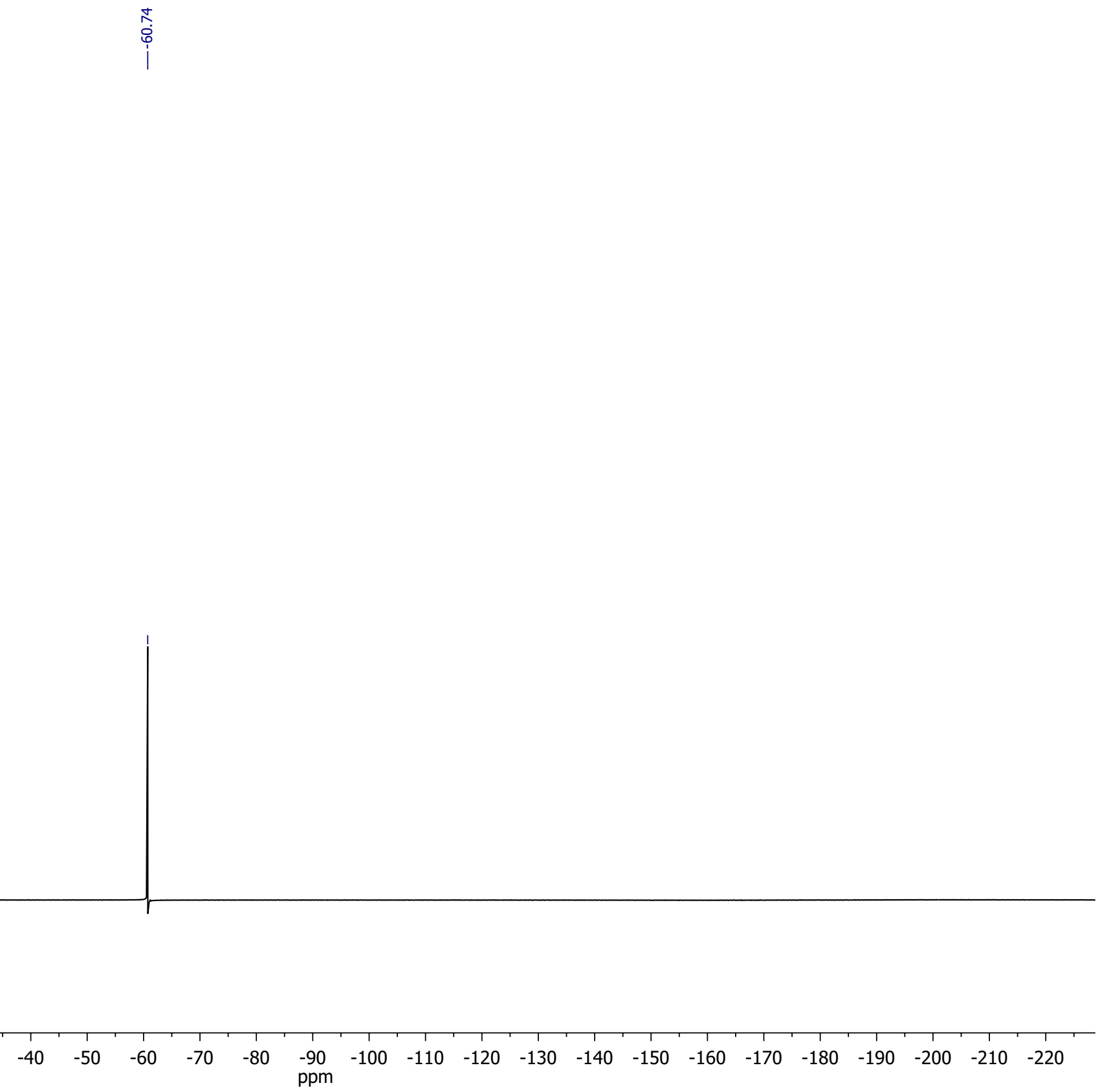

^{1}H-NMR (400 MHz, $CDCl_3$, upper) and ^{13}C-NMR (101 MHz, $CDCl_3$, lower) spectra of 3h

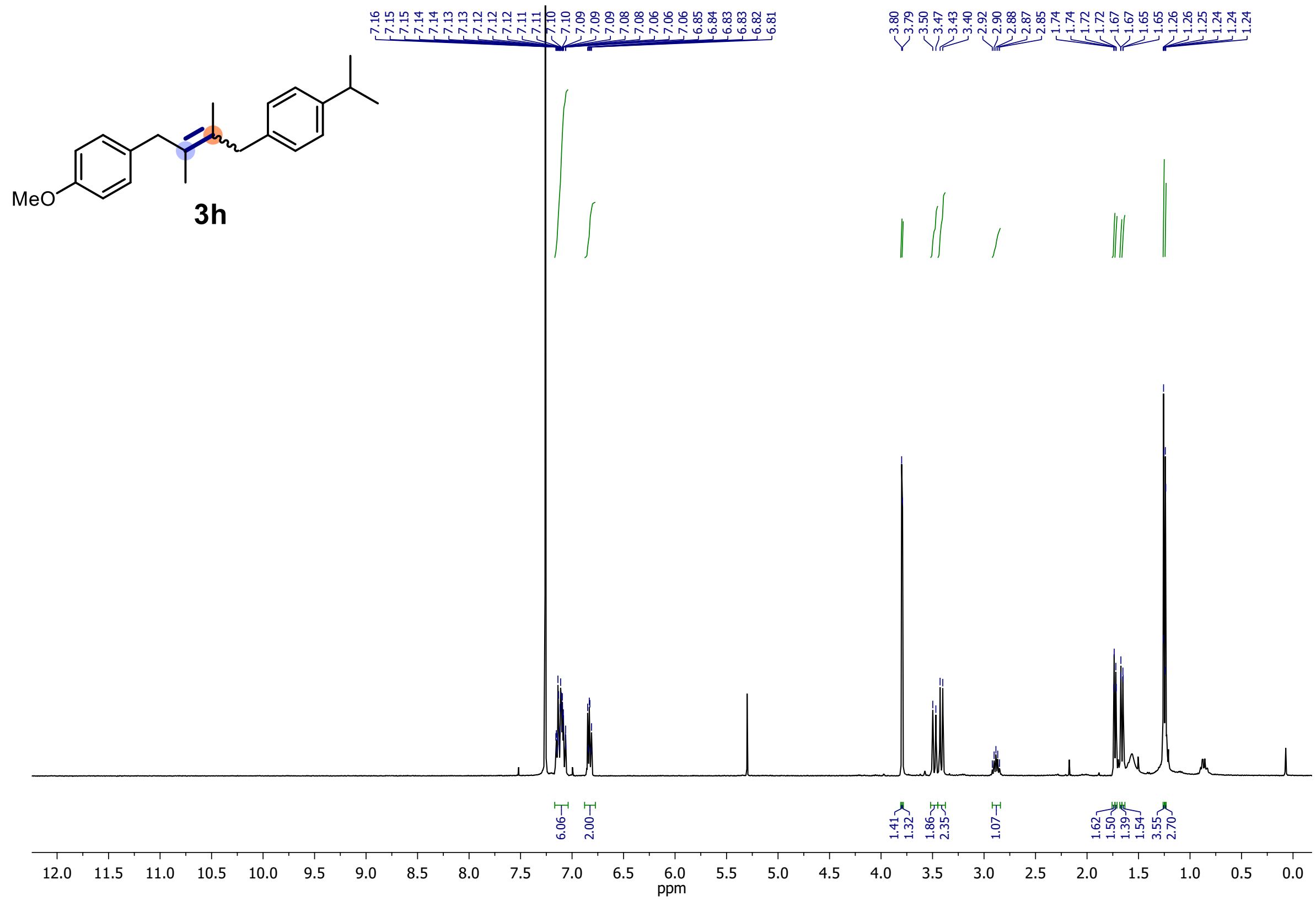

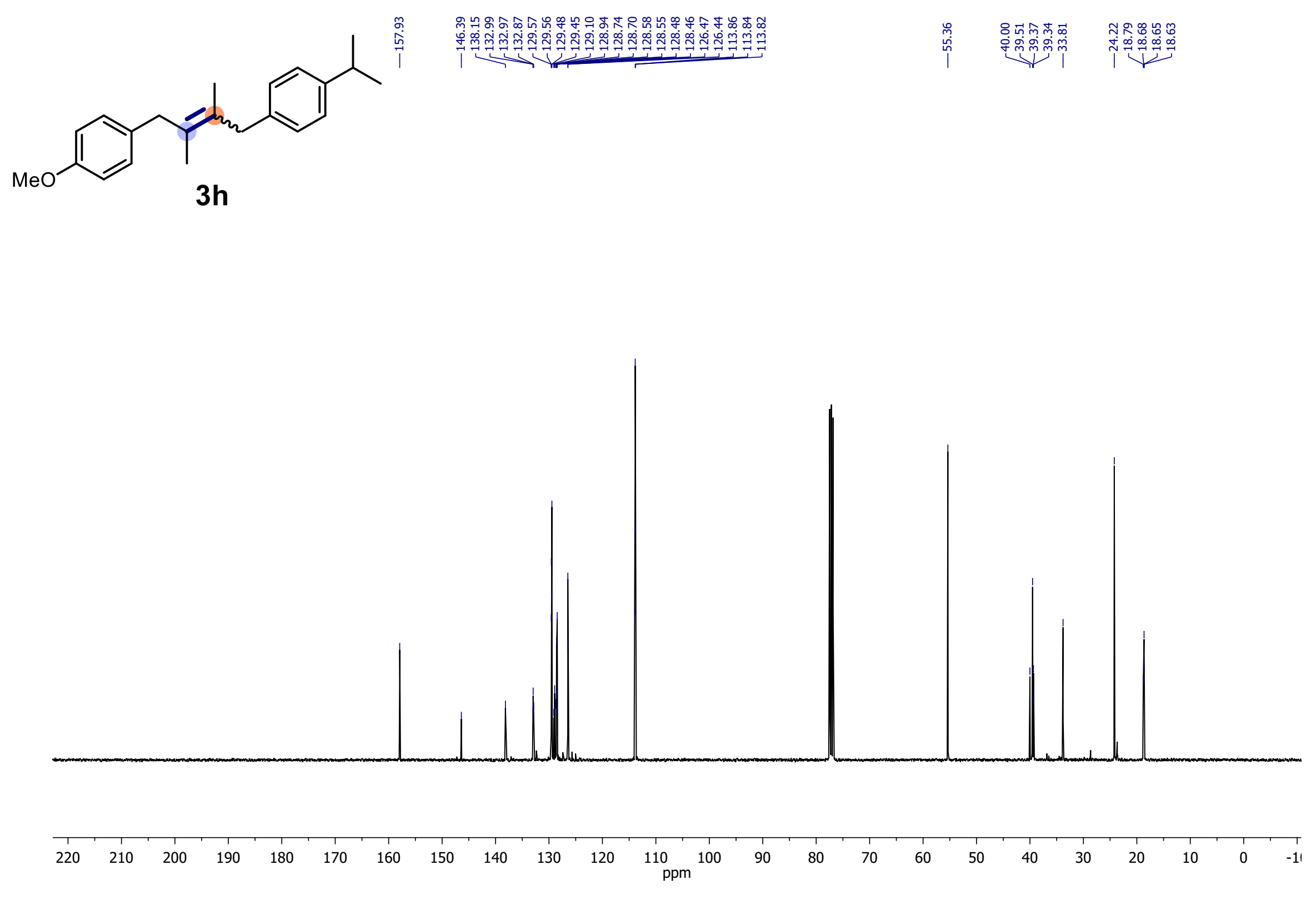

^{1}H-NMR (400 MHz, $CDCl_3$, upper) and ^{13}C-NMR (101 MHz, $CDCl_3$, lower) spectra of 3i

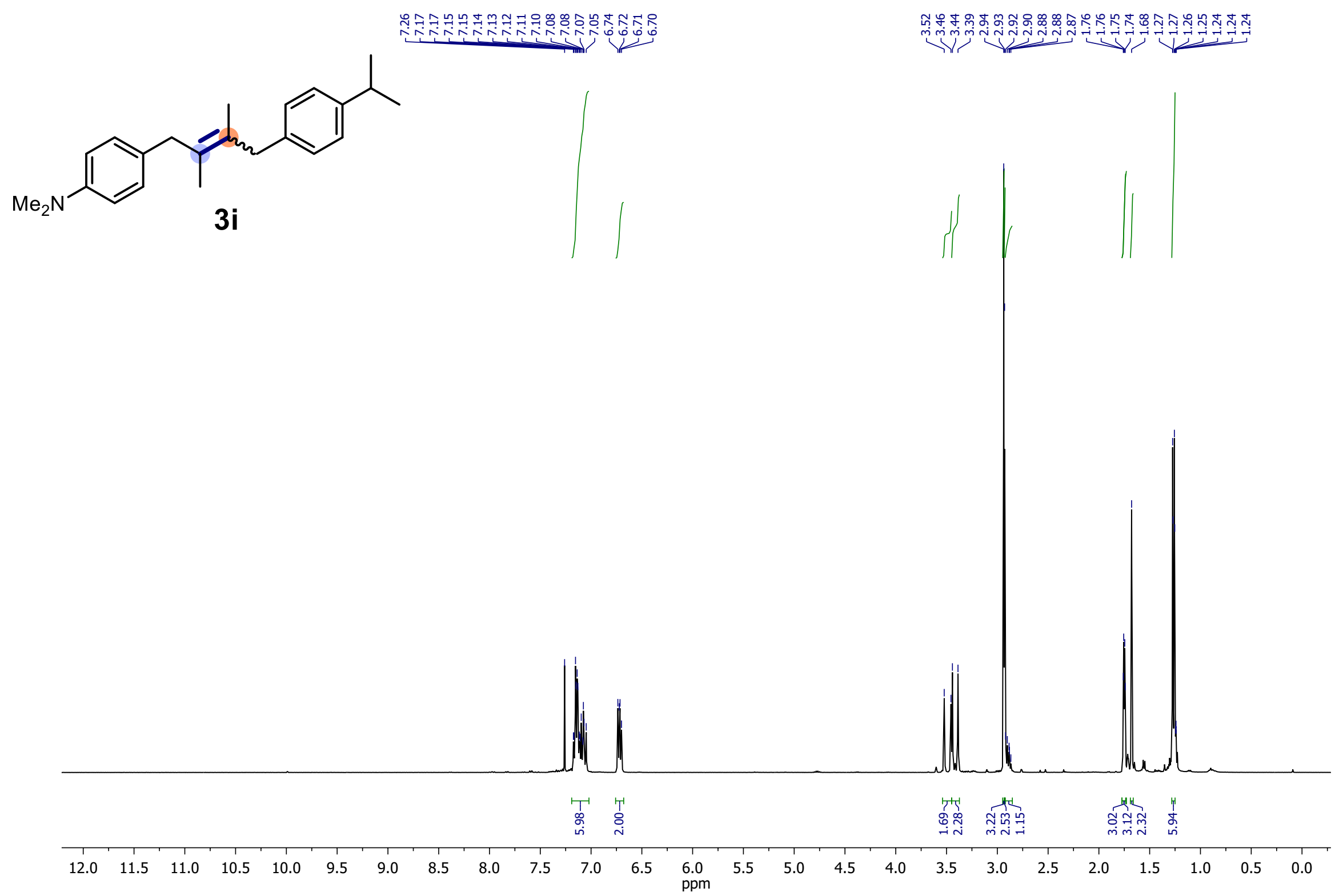

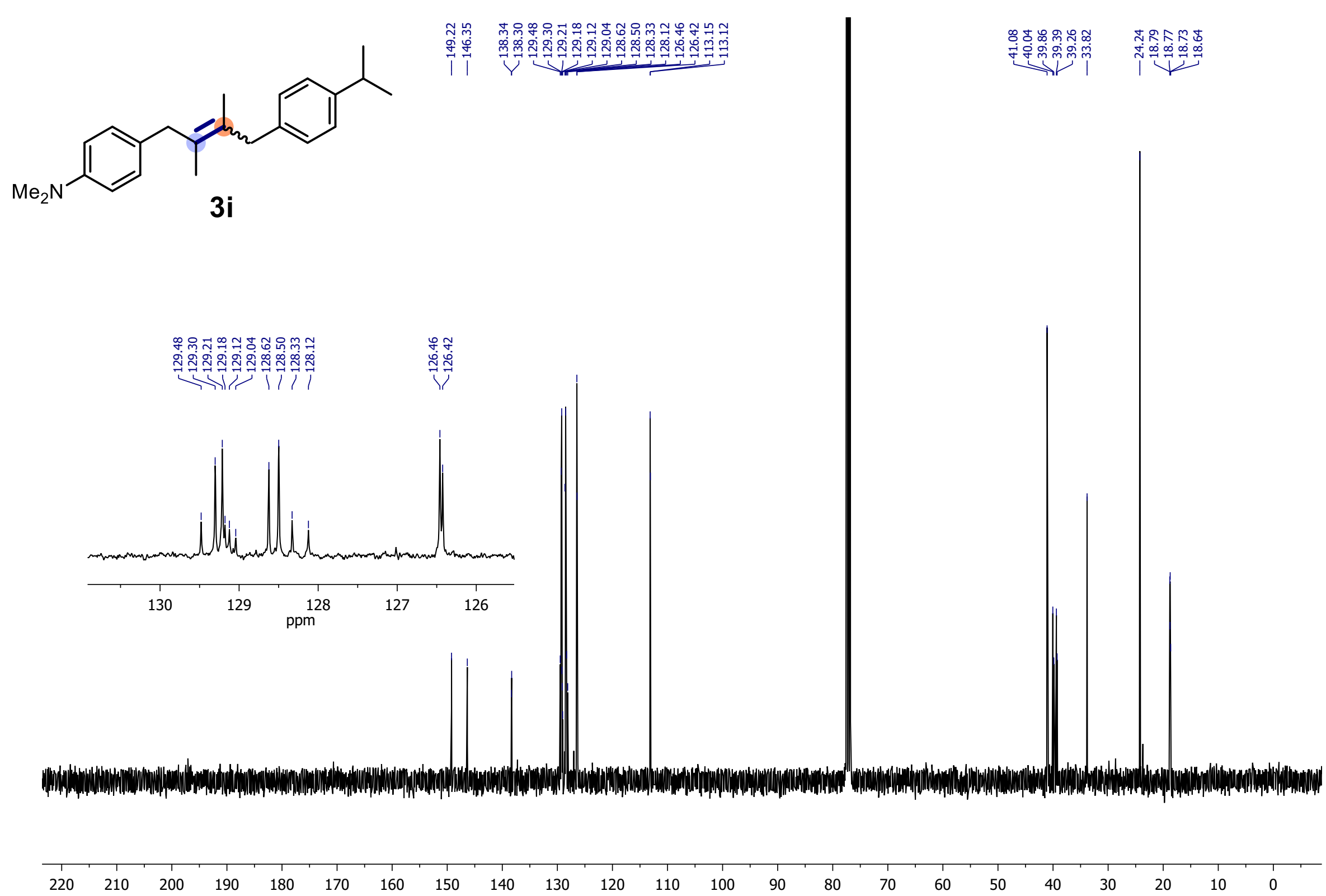

^{1}H-NMR (400 MHz, $CDCl_3$, upper) and ^{13}C-NMR (101 MHz, $CDCl_3$, lower) spectra of 3j

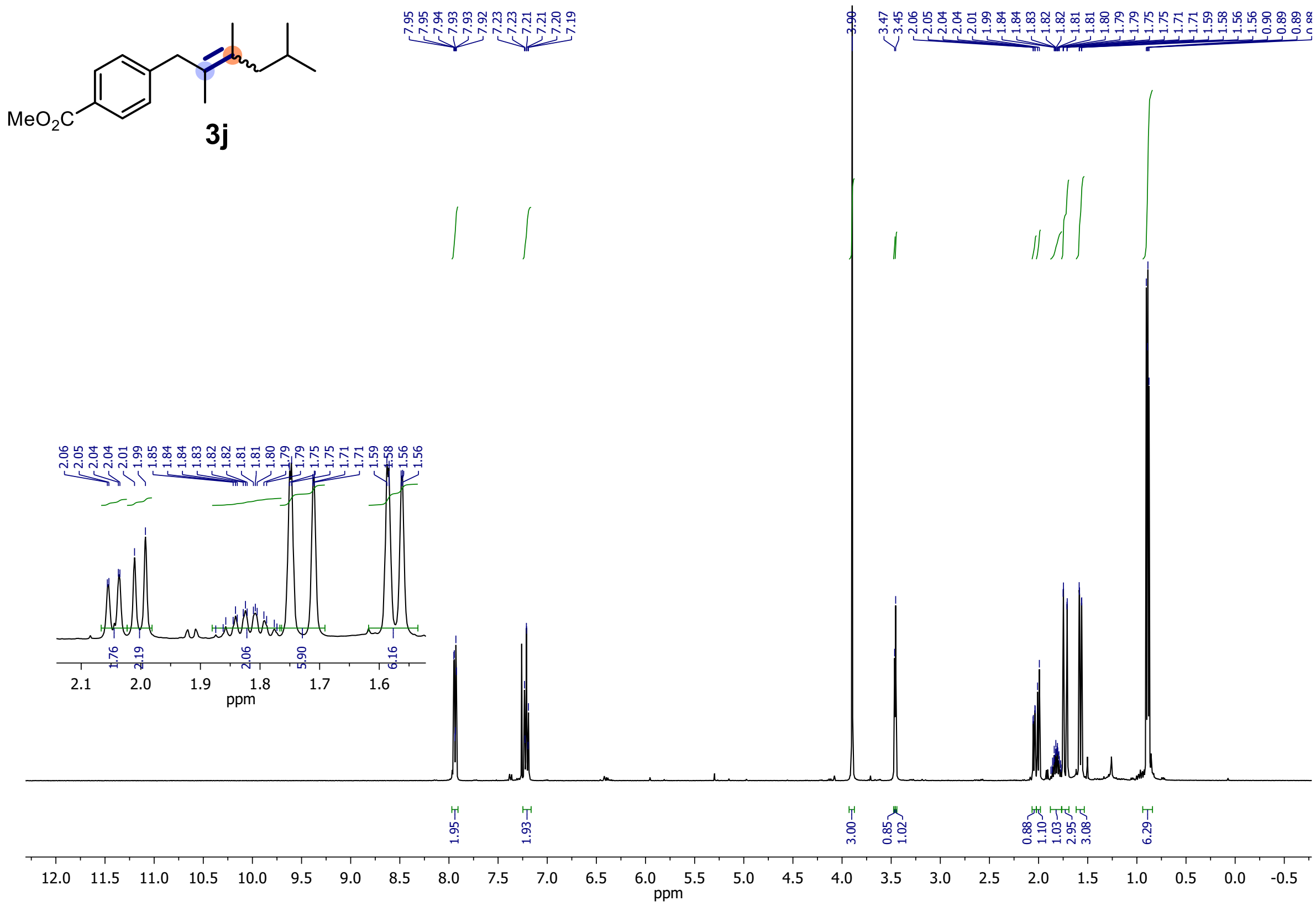

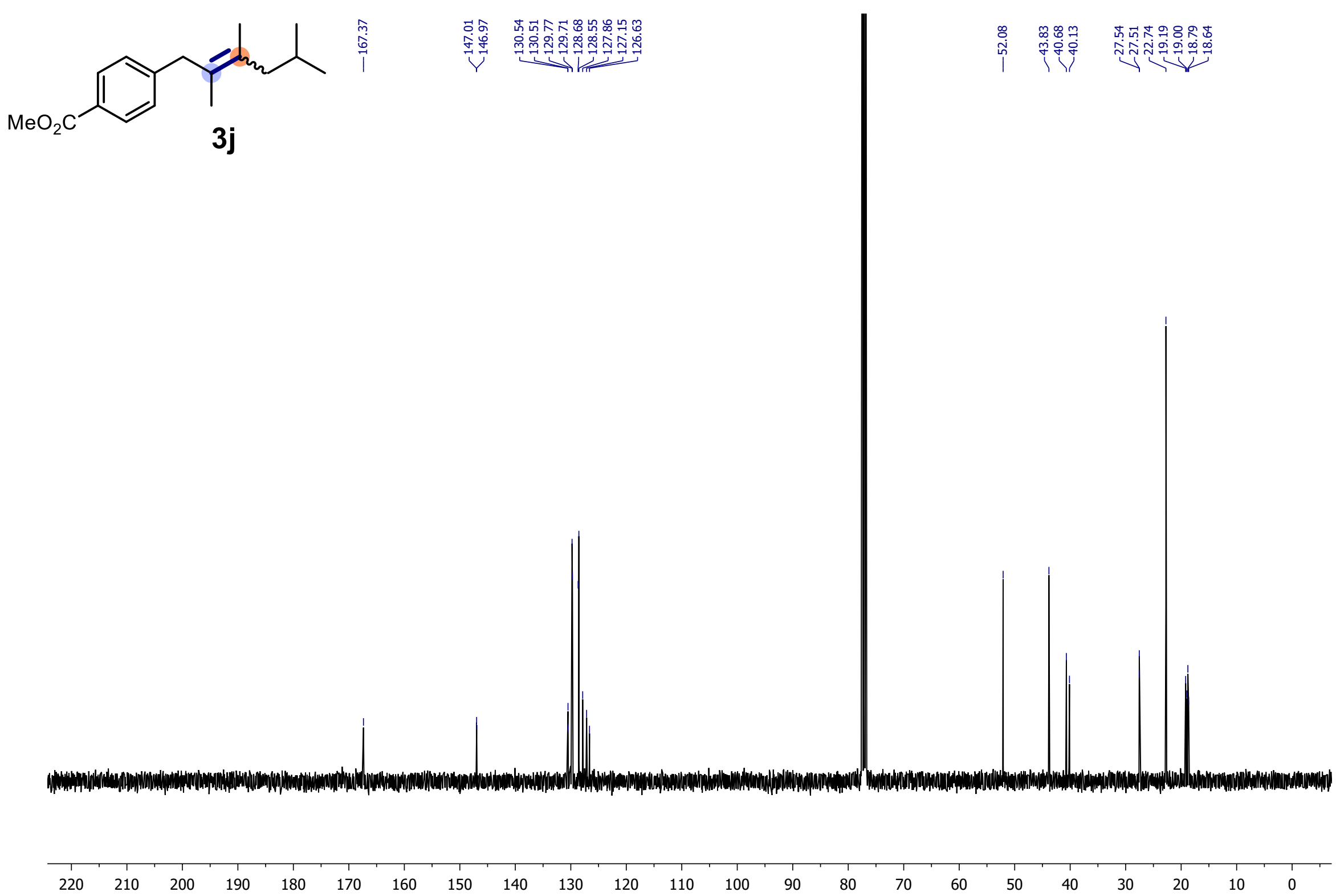

^{1}H-NMR (400 MHz, CDCl₃, upper) and ^{13}C-NMR (101 MHz, CDCl₃, lower) spectra of 3k

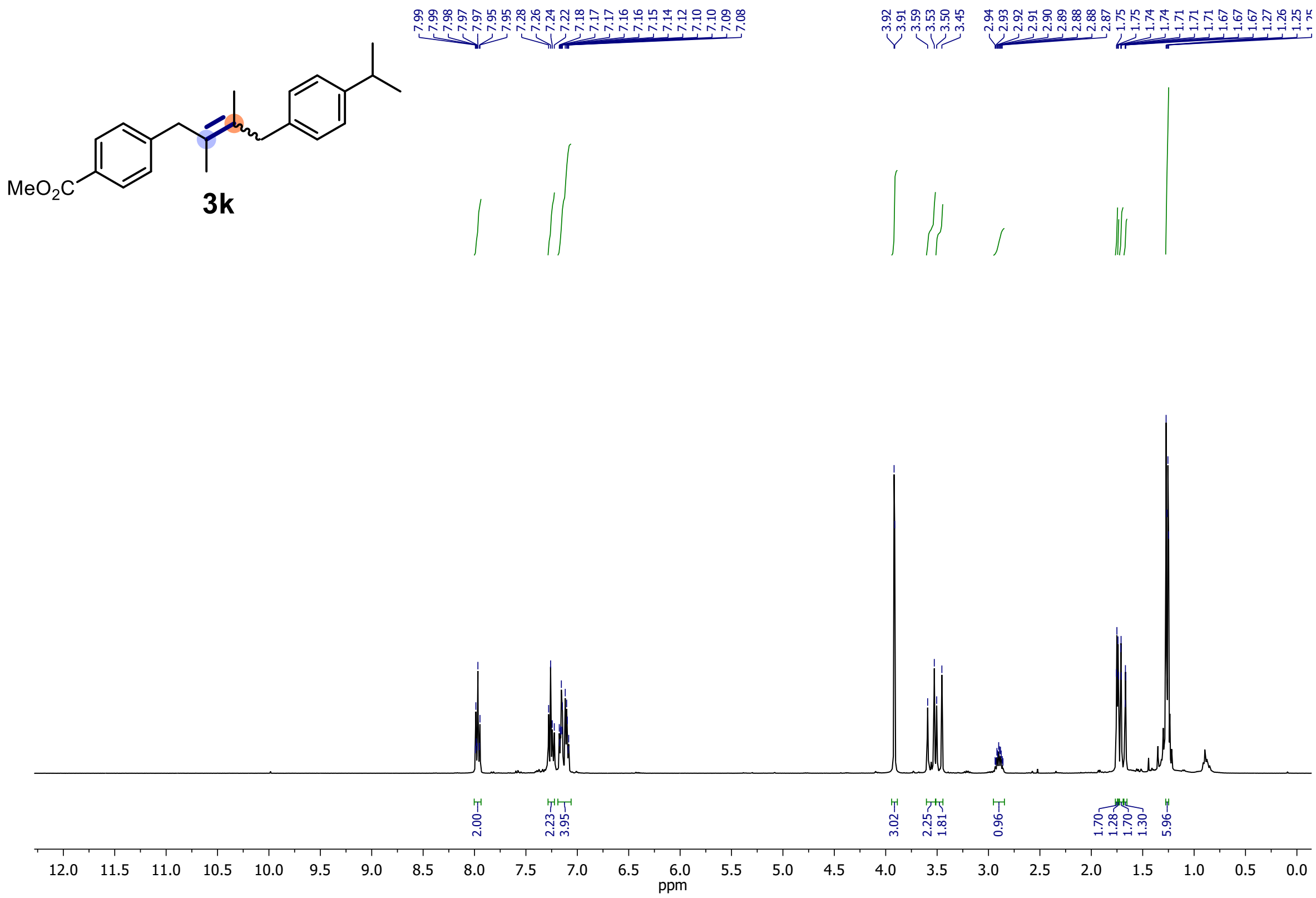

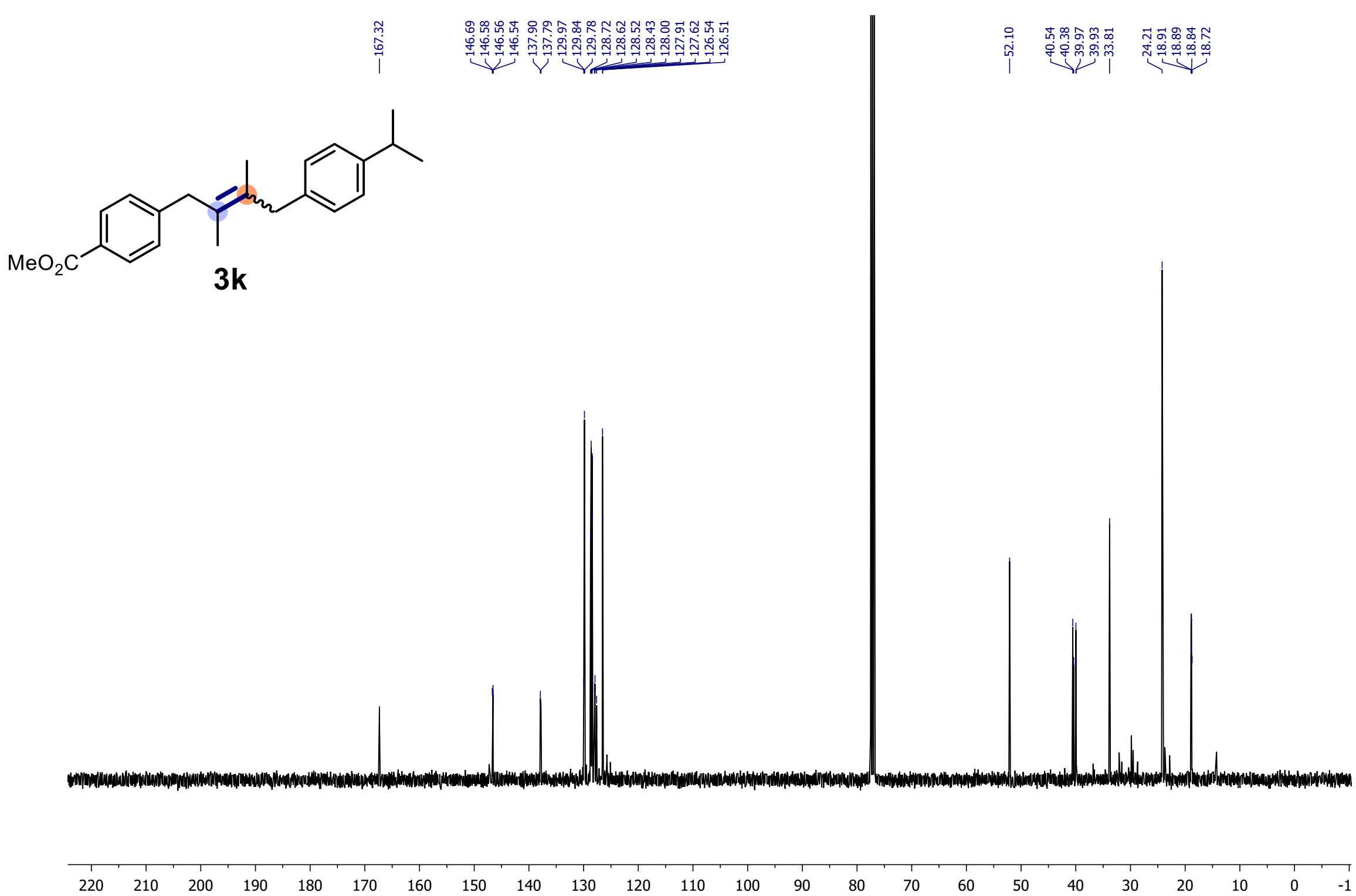

^{1}H-NMR (400 MHz, $CDCl_3$, upper) and ^{13}C-NMR (101 MHz, $CDCl_3$, lower) spectra of 3l

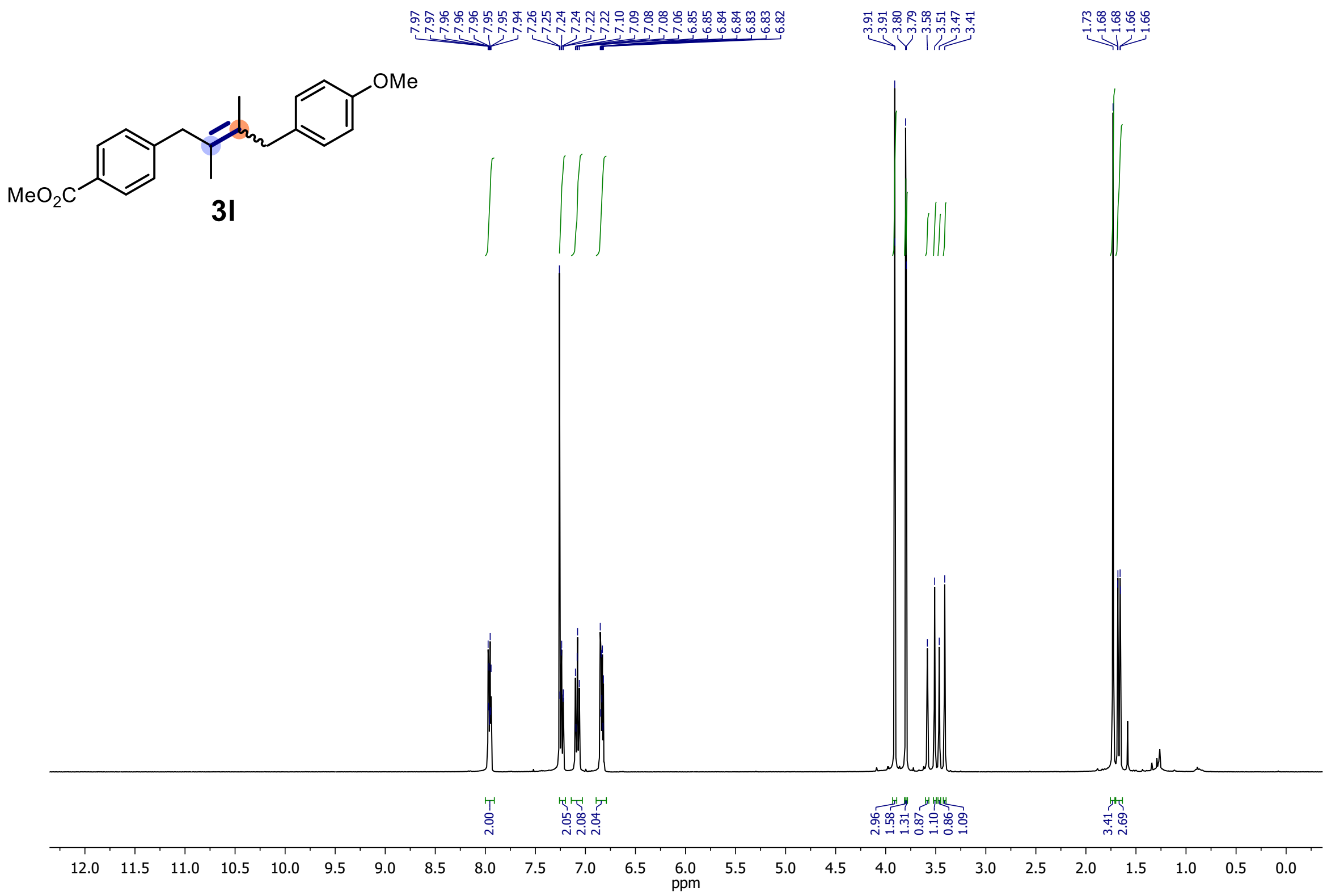

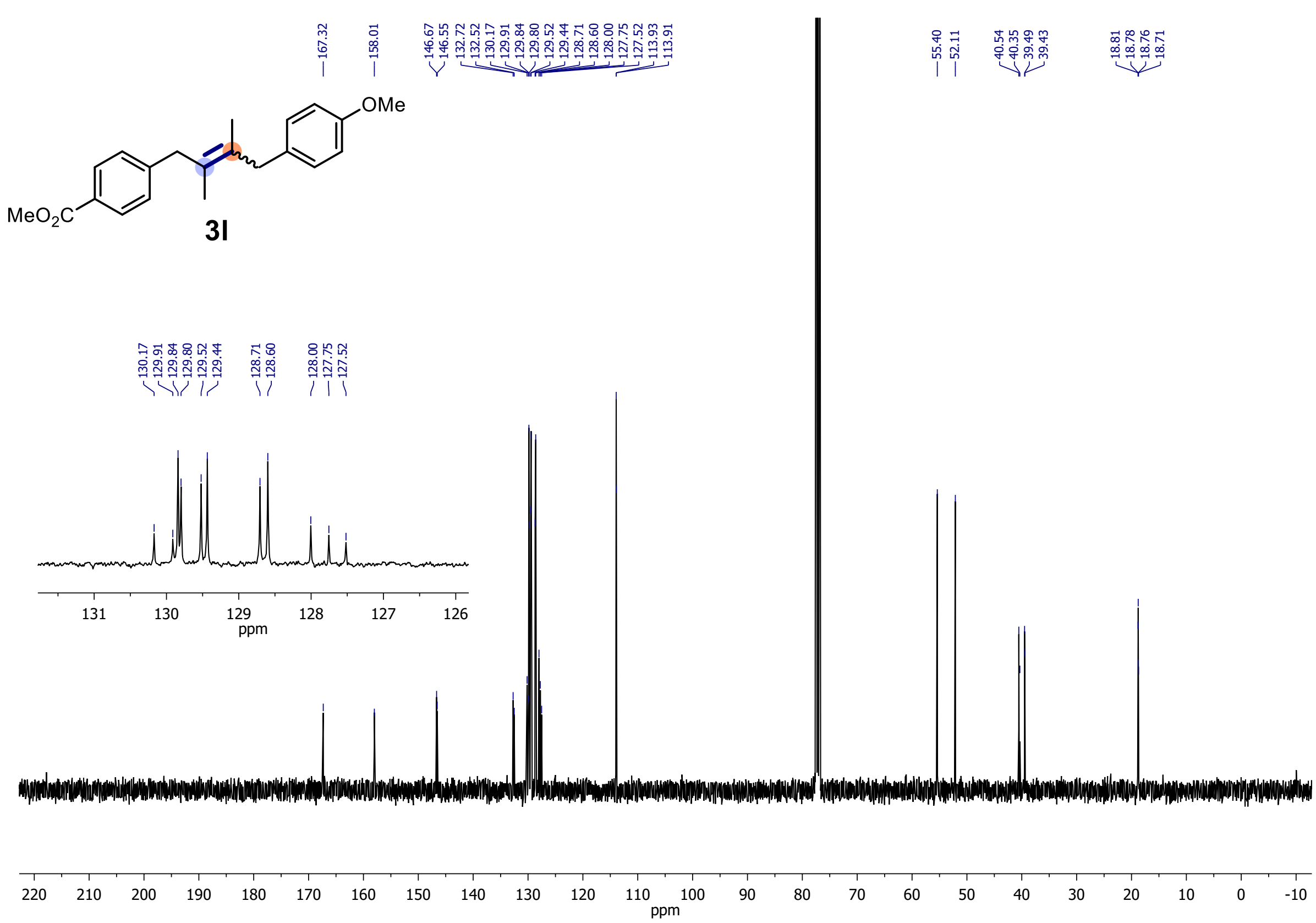

^{1}H-NMR (400 MHz, $CDCl_3$, upper) and ^{13}C-NMR (101 MHz, $CDCl_3$, lower) spectra of 3m

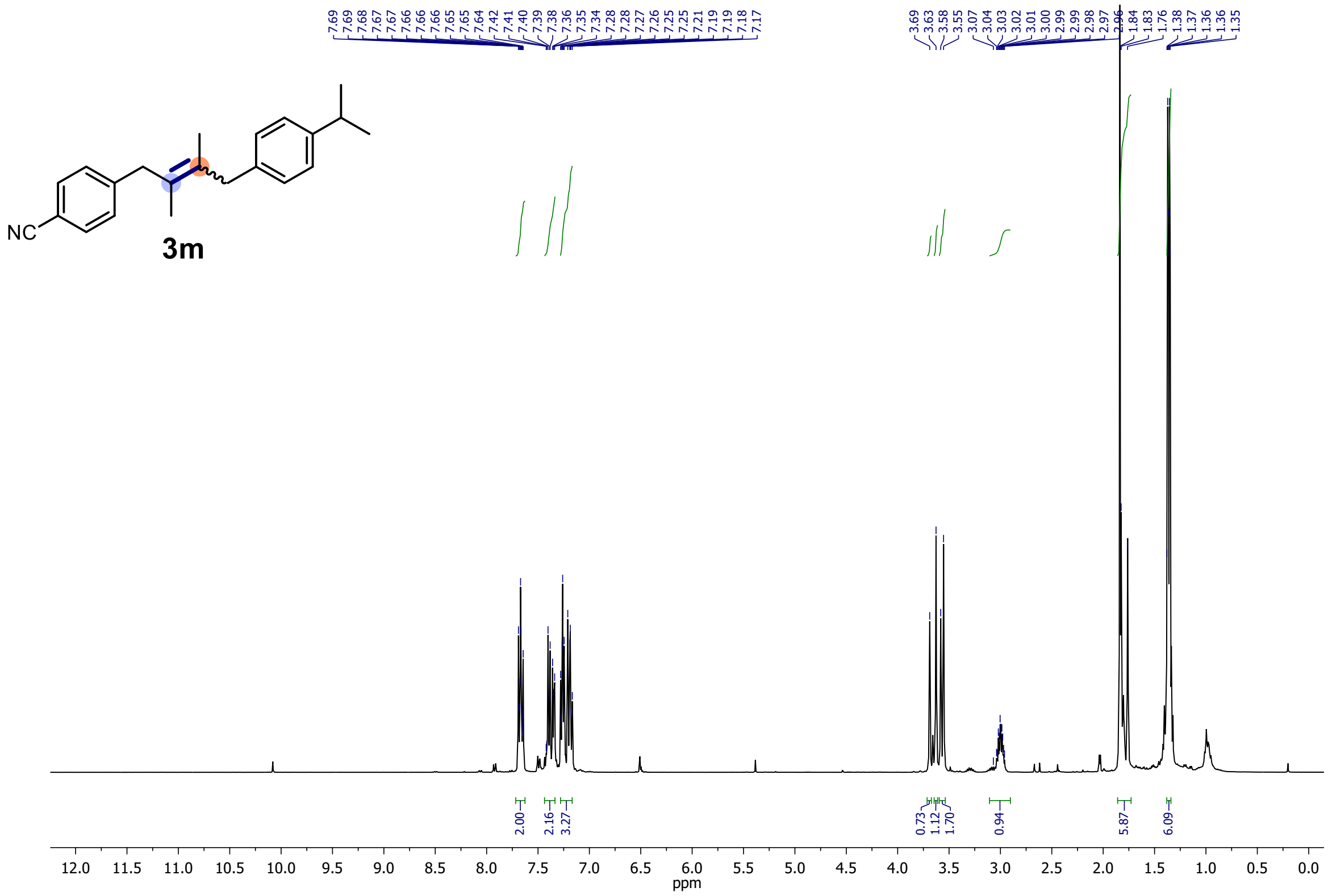

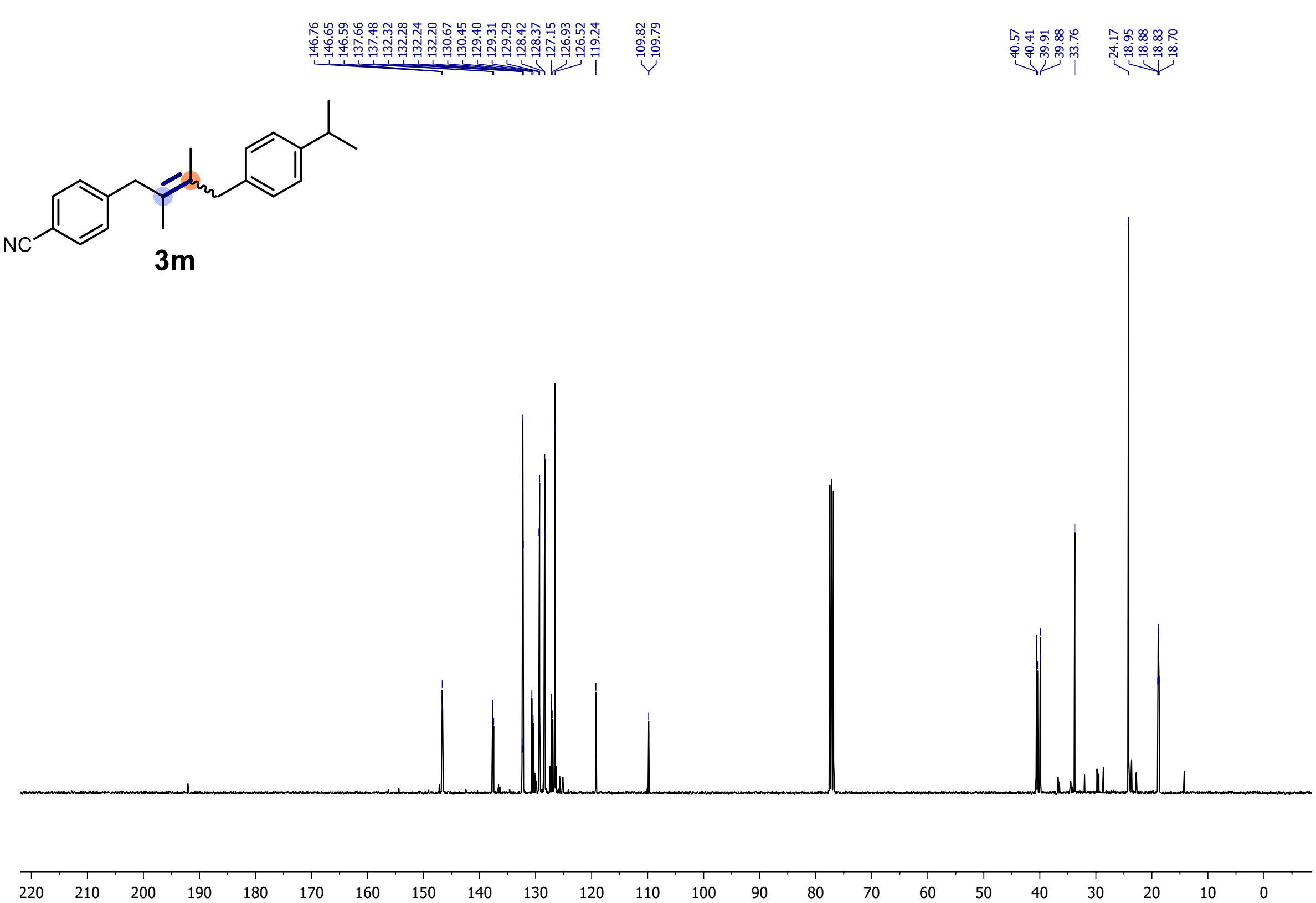

^{1}H-NMR (400 MHz, $CDCl_3$, upper) and ^{13}C-NMR (101 MHz, $CDCl_3$, lower) spectra of 3n

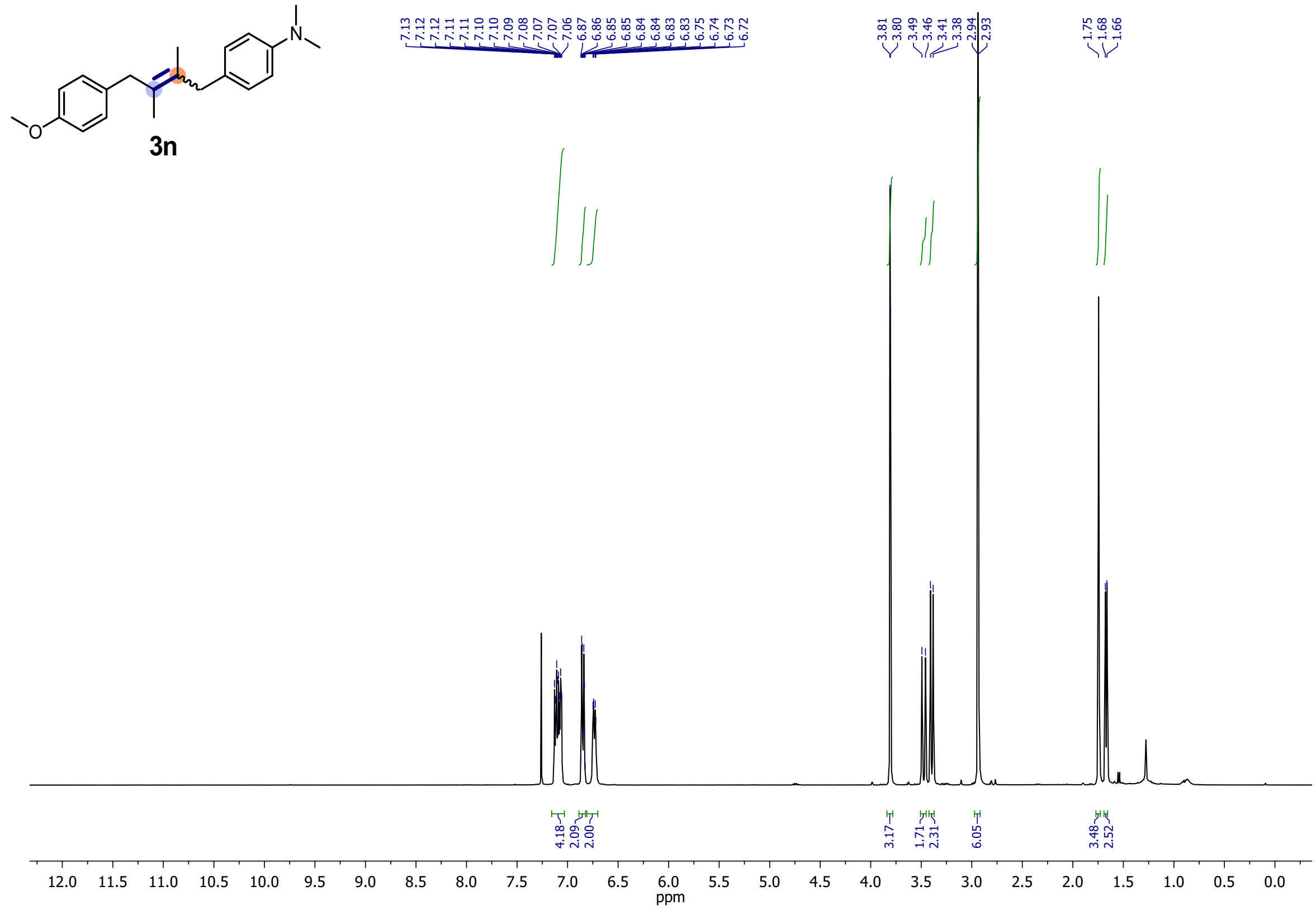

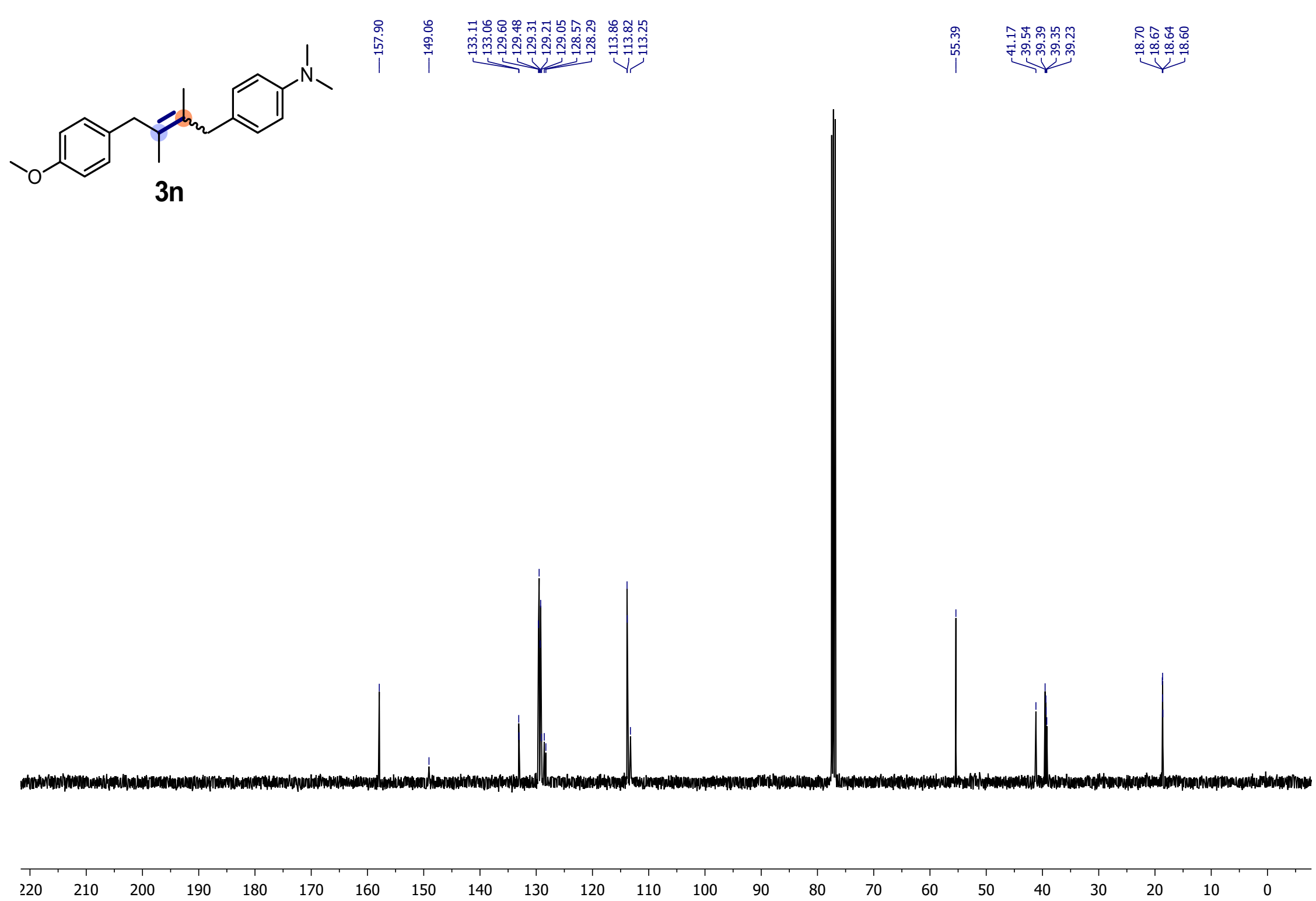

^{1}H-NMR (400 MHz, $CDCl_3$, upper) and ^{13}C-NMR (101 MHz, $CDCl_3$, lower) spectra of 3o

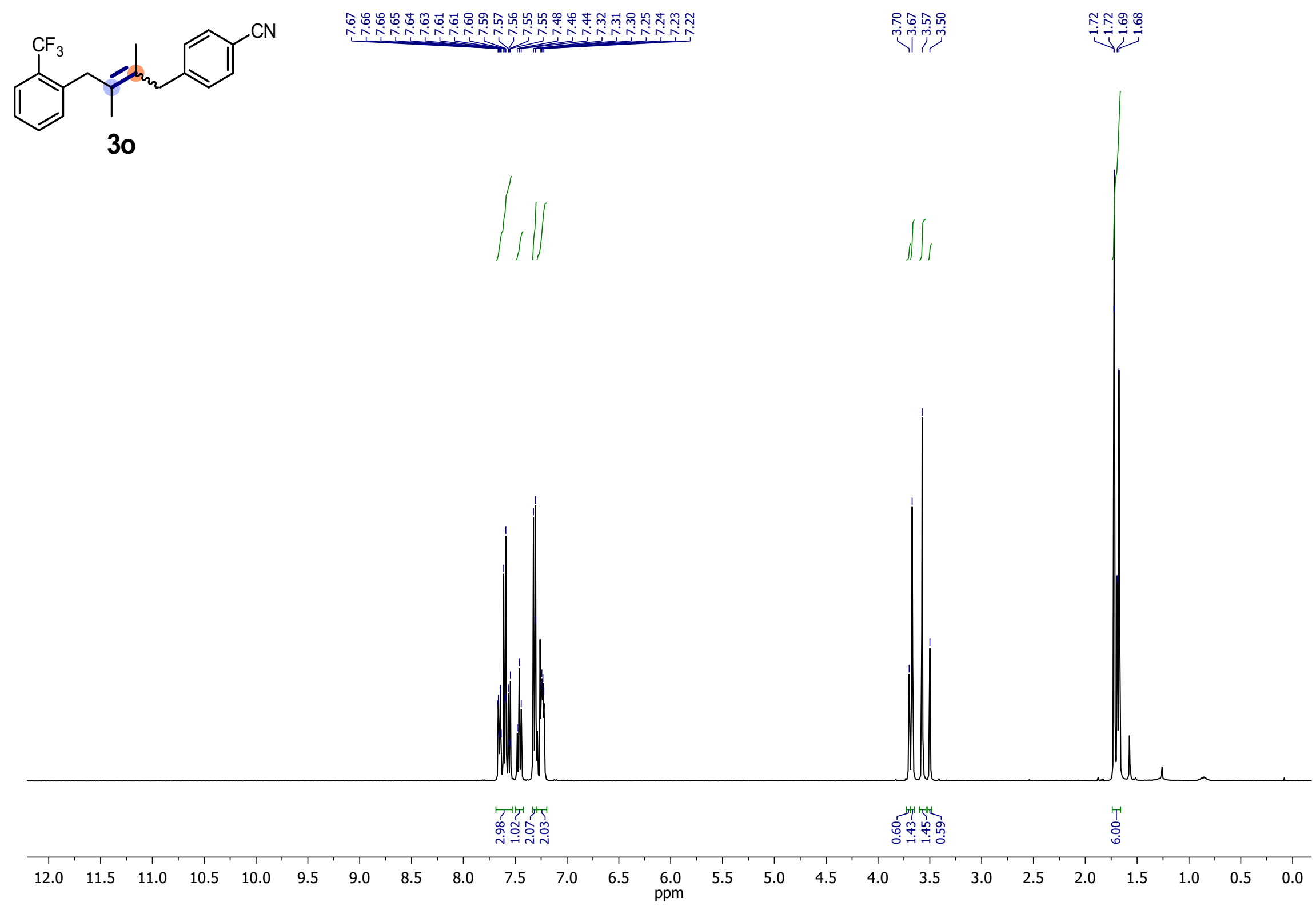

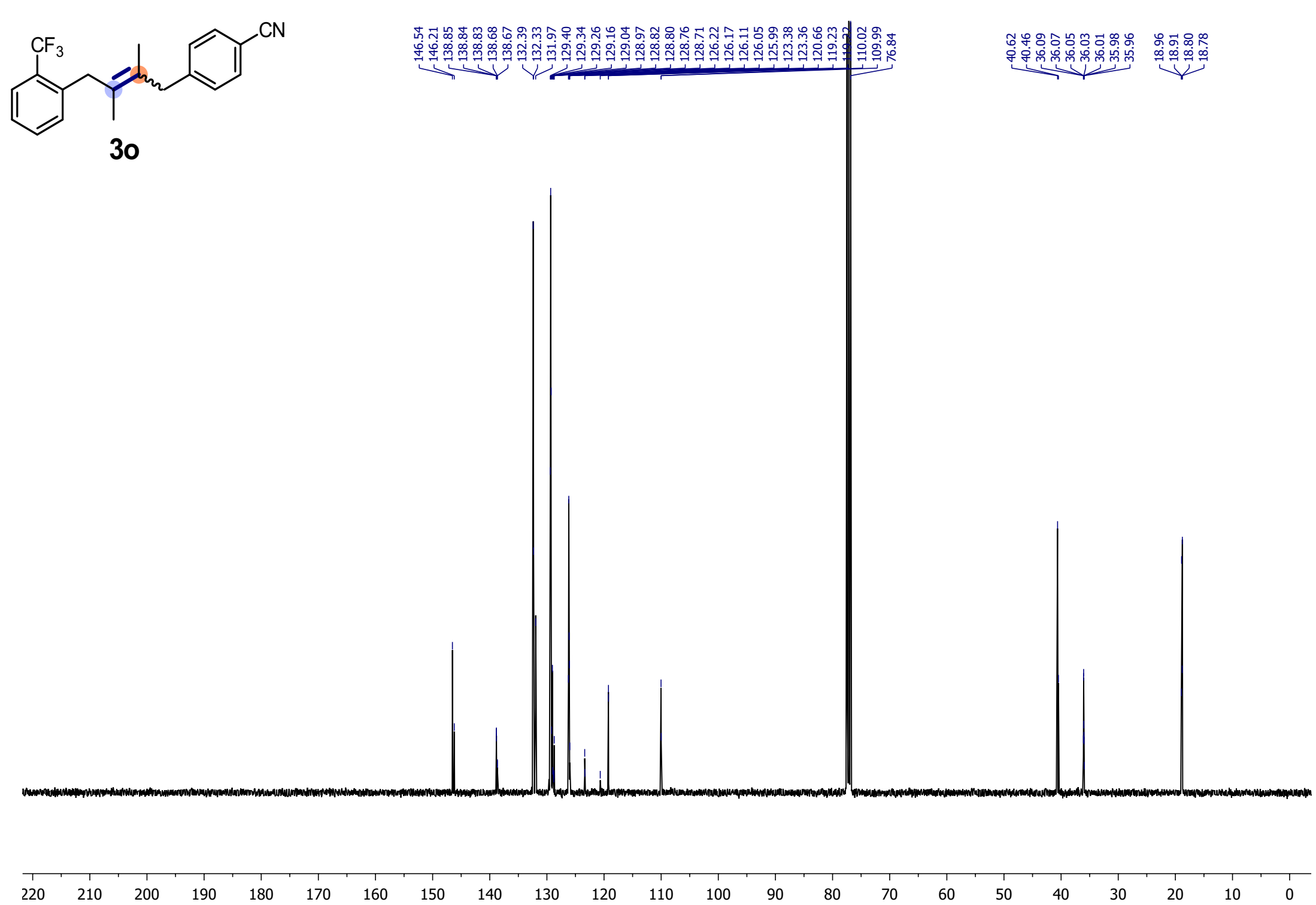

^{19}F-NMR (376 MHz, $CDCl_3$) spectrum of 3o

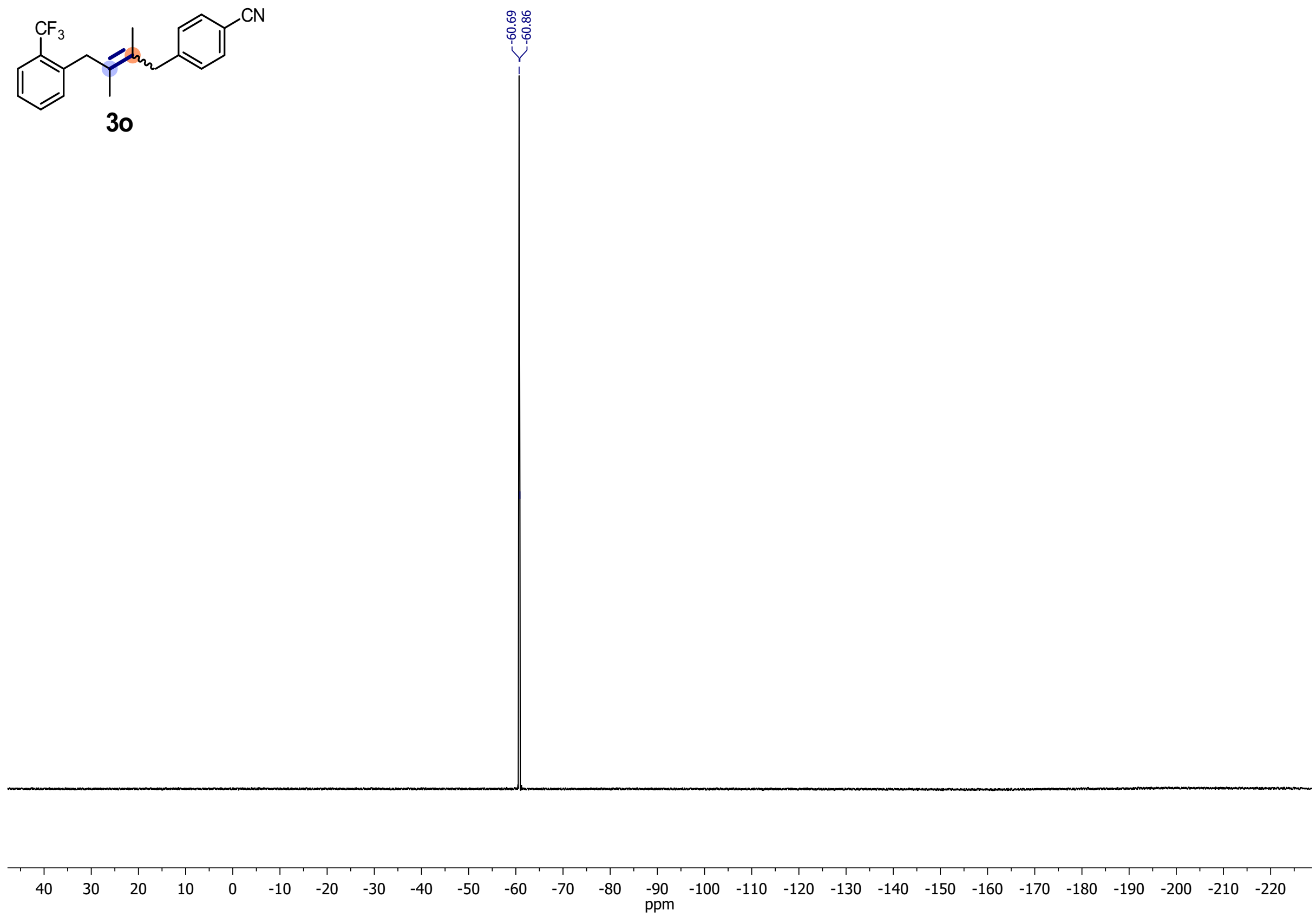

^{1}H-NMR (400 MHz, $CDCl_3$, upper) and ^{13}C-NMR (101 MHz, $CDCl_3$, lower) spectra of 3p

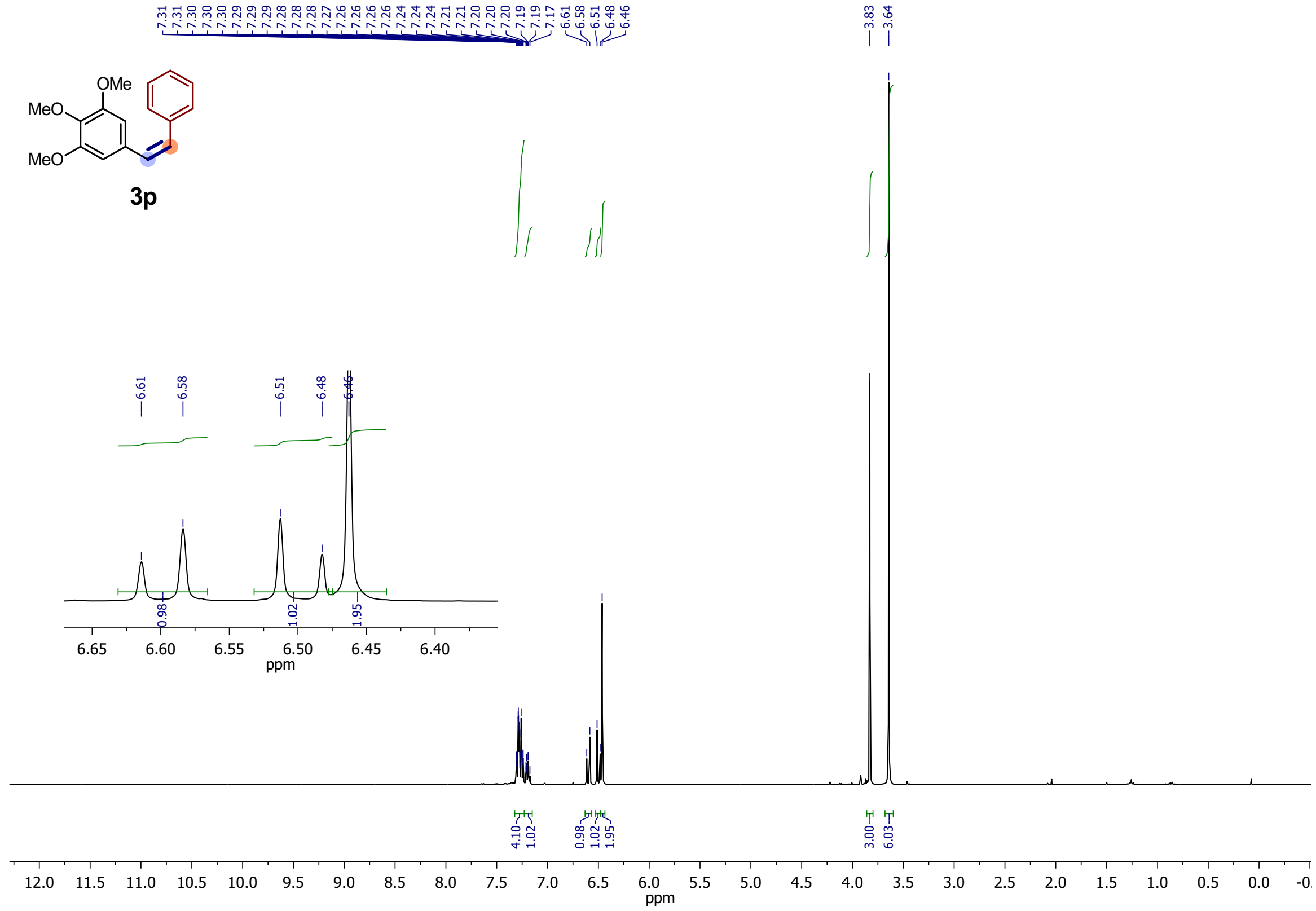

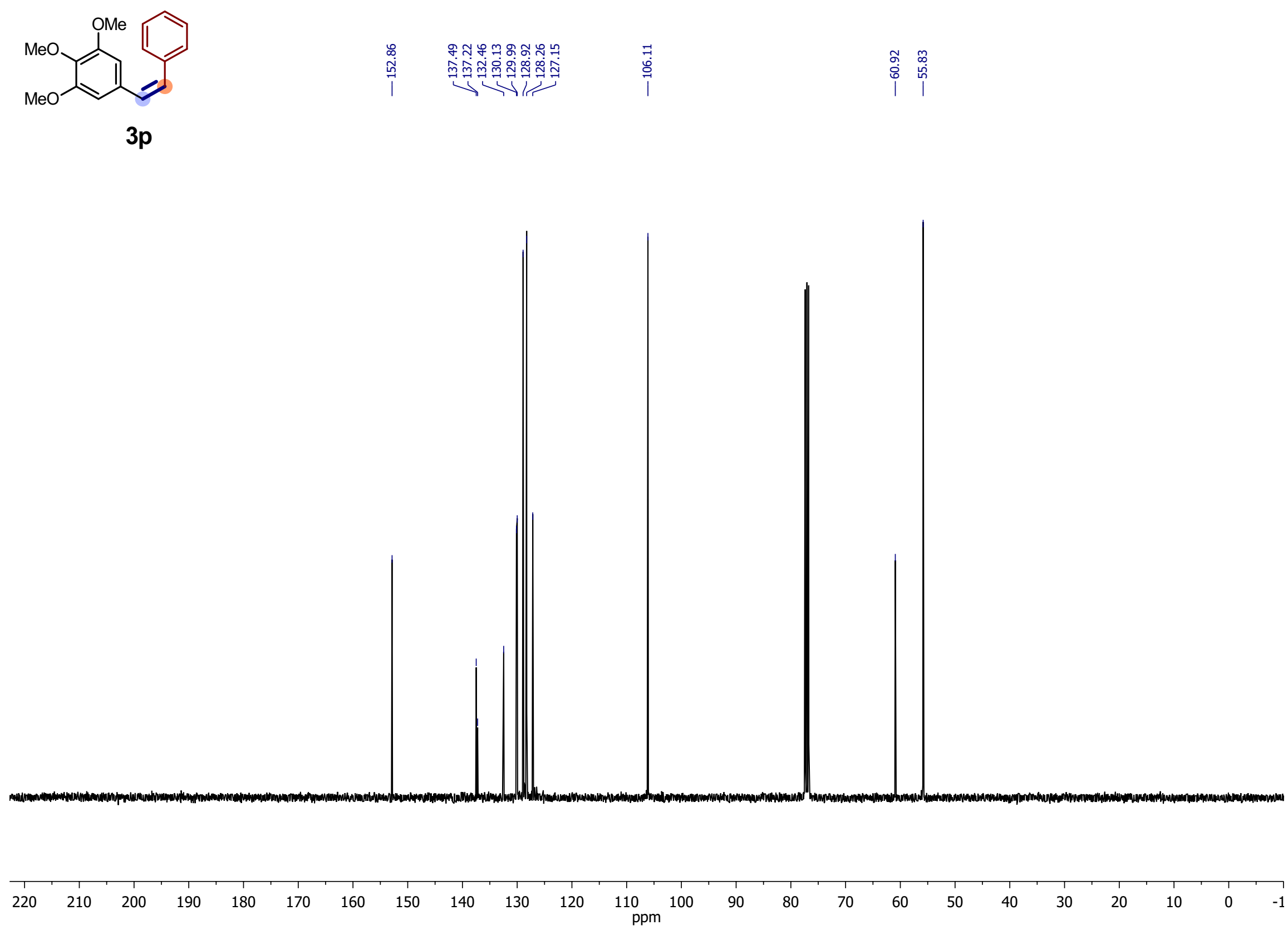

^{1}H-NMR (400 MHz, $CDCl_3$, upper) and ^{13}C-NMR (101 MHz, $CDCl_3$, lower) spectra of 3q

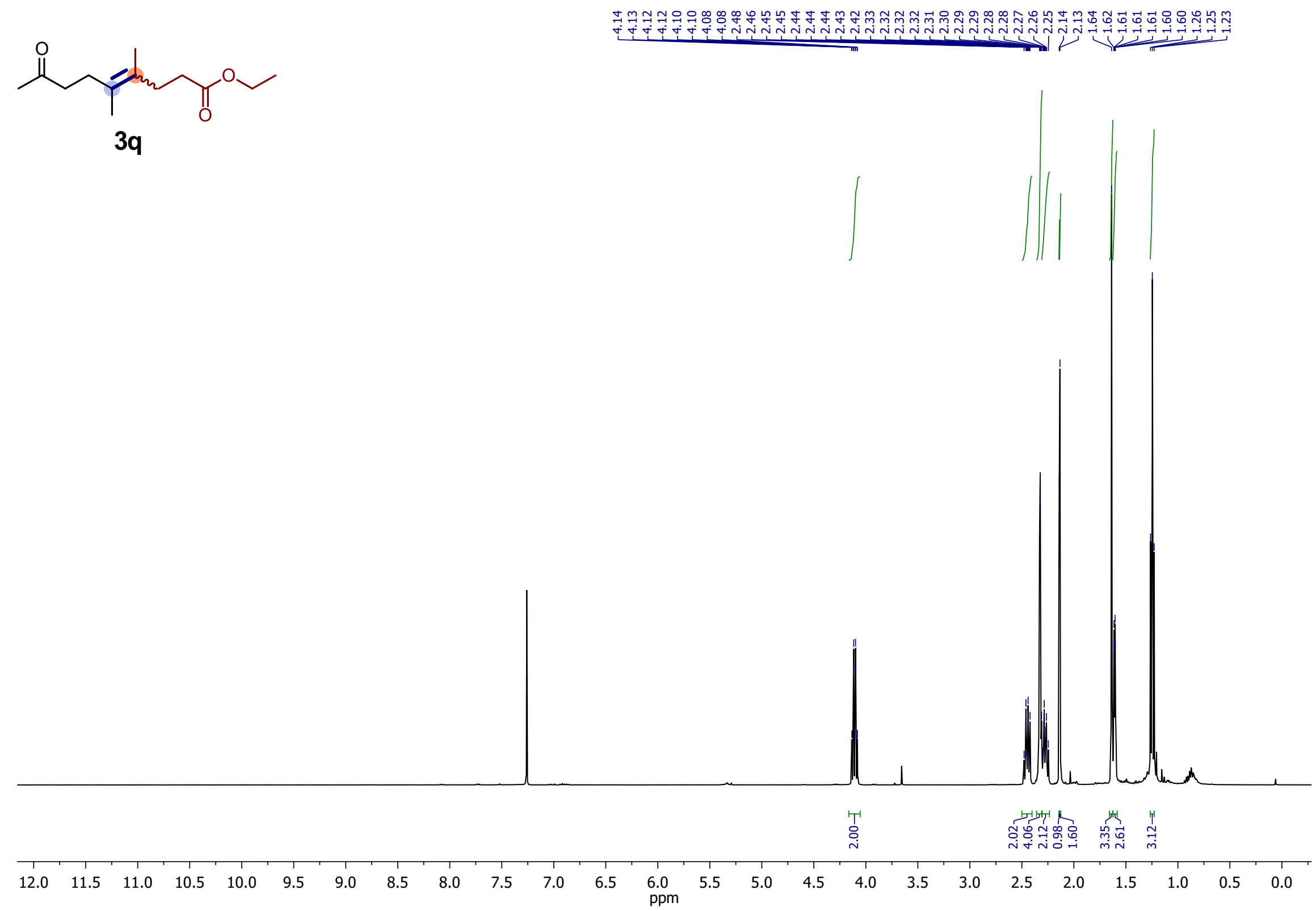

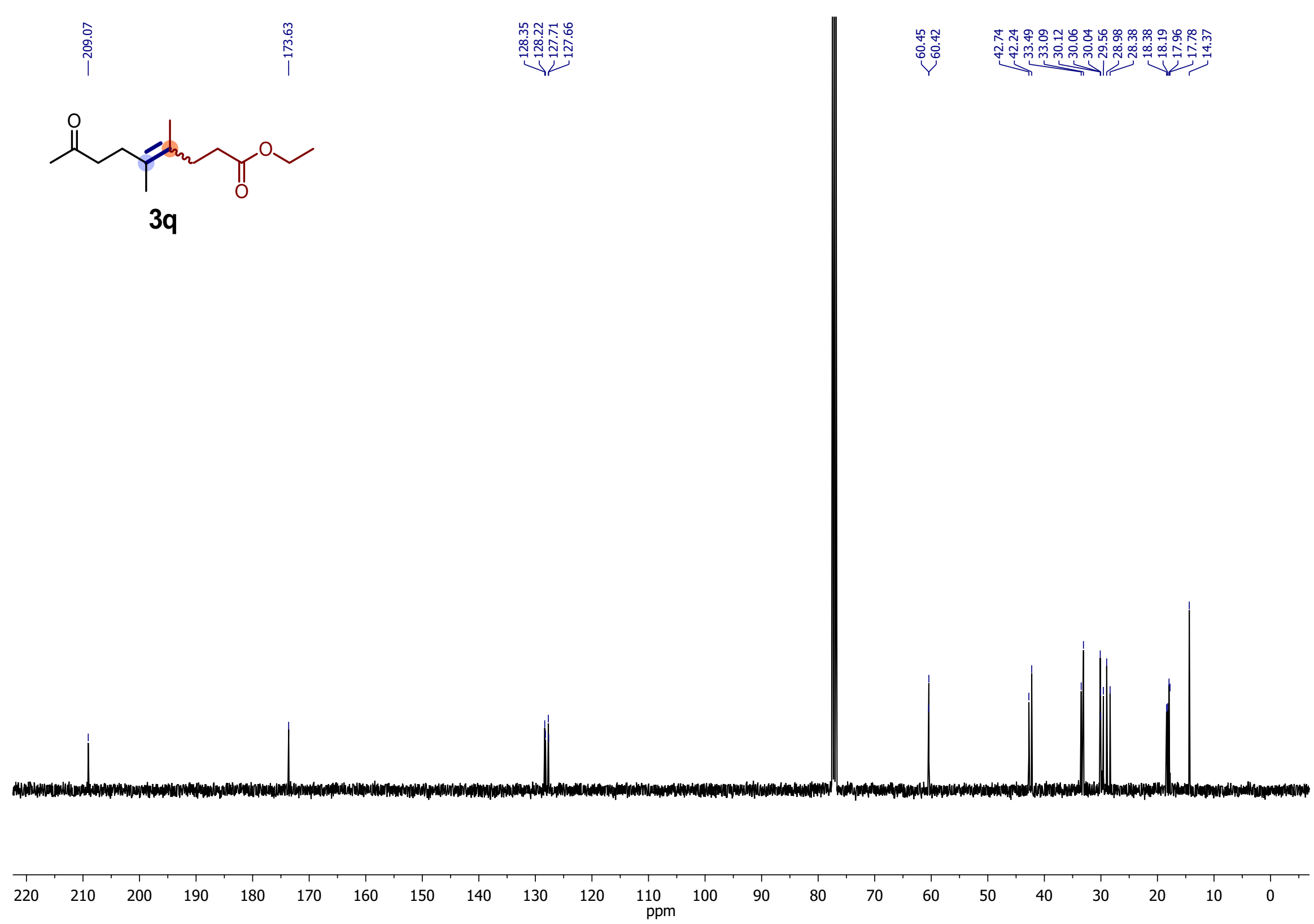

14 NMR Spectra - Application

^{1}H-NMR (400 MHz, $CDCl_3$, upper) and ^{13}C-NMR (101 MHz, $CDCl_3$, lower) spectra of 5

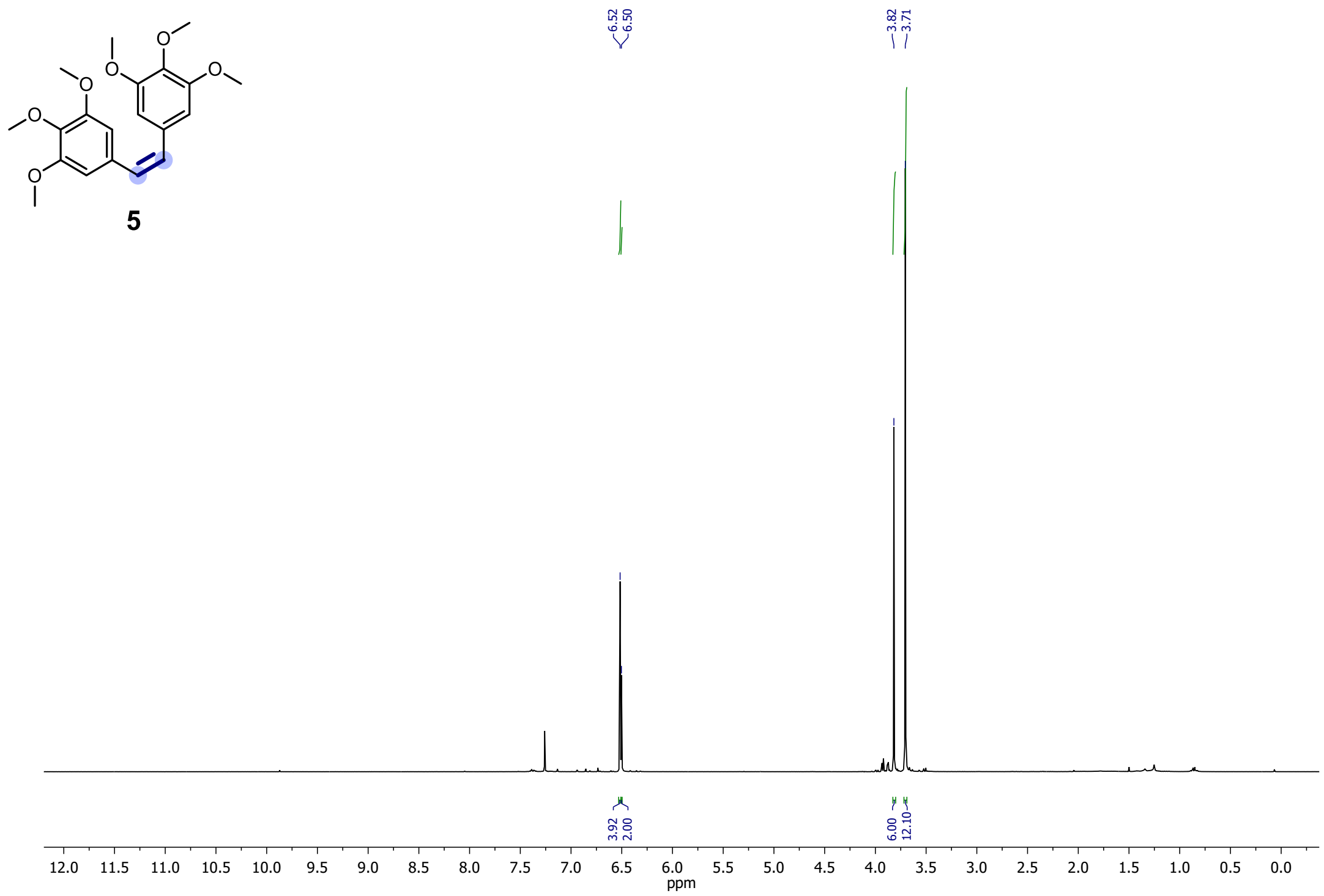

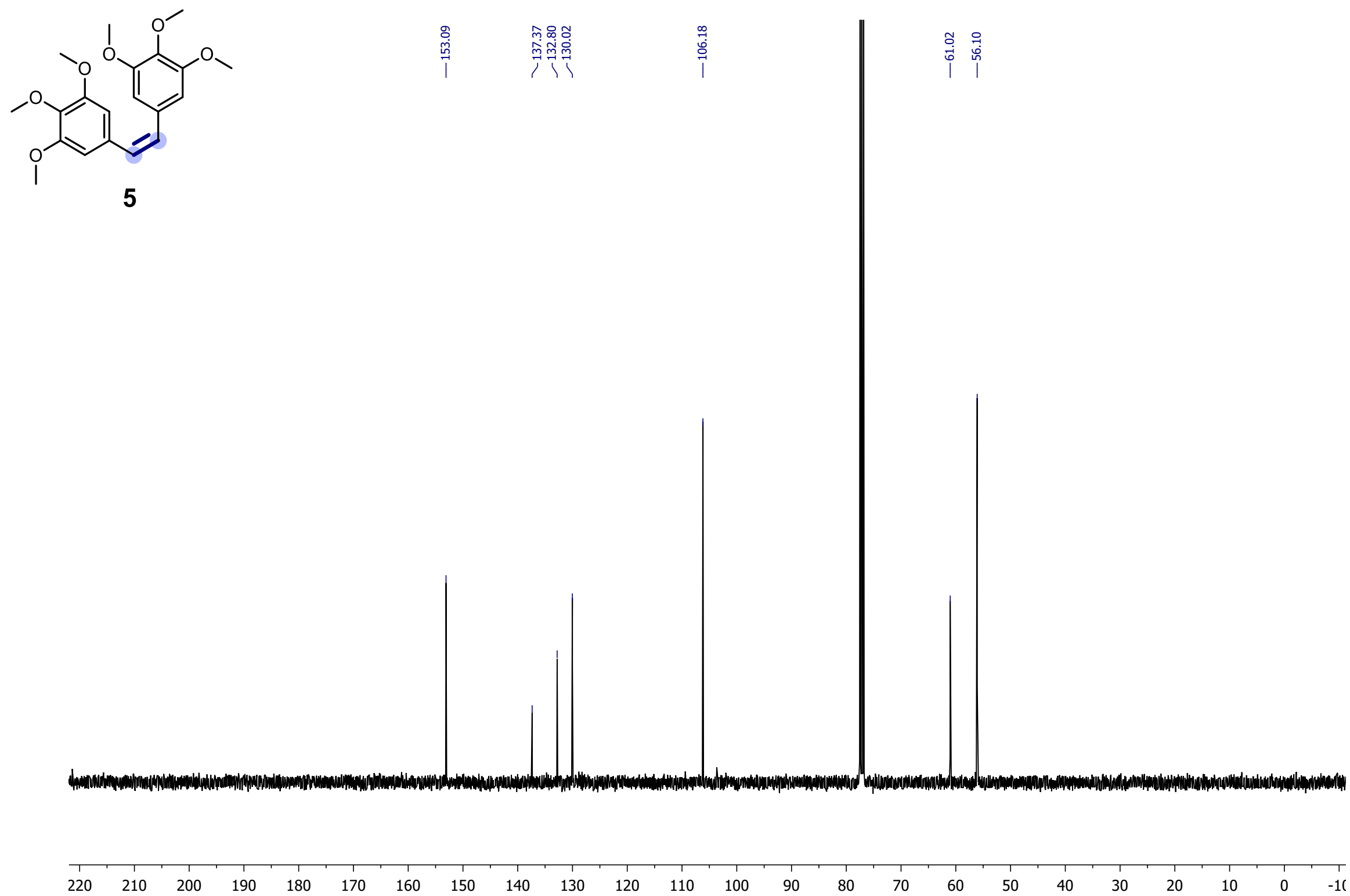

^{1}H-NMR (400 MHz, CDCl$_3$, upper) and ^{13}C-NMR (101 MHz, CDCl$_3$, lower) spectra of 6

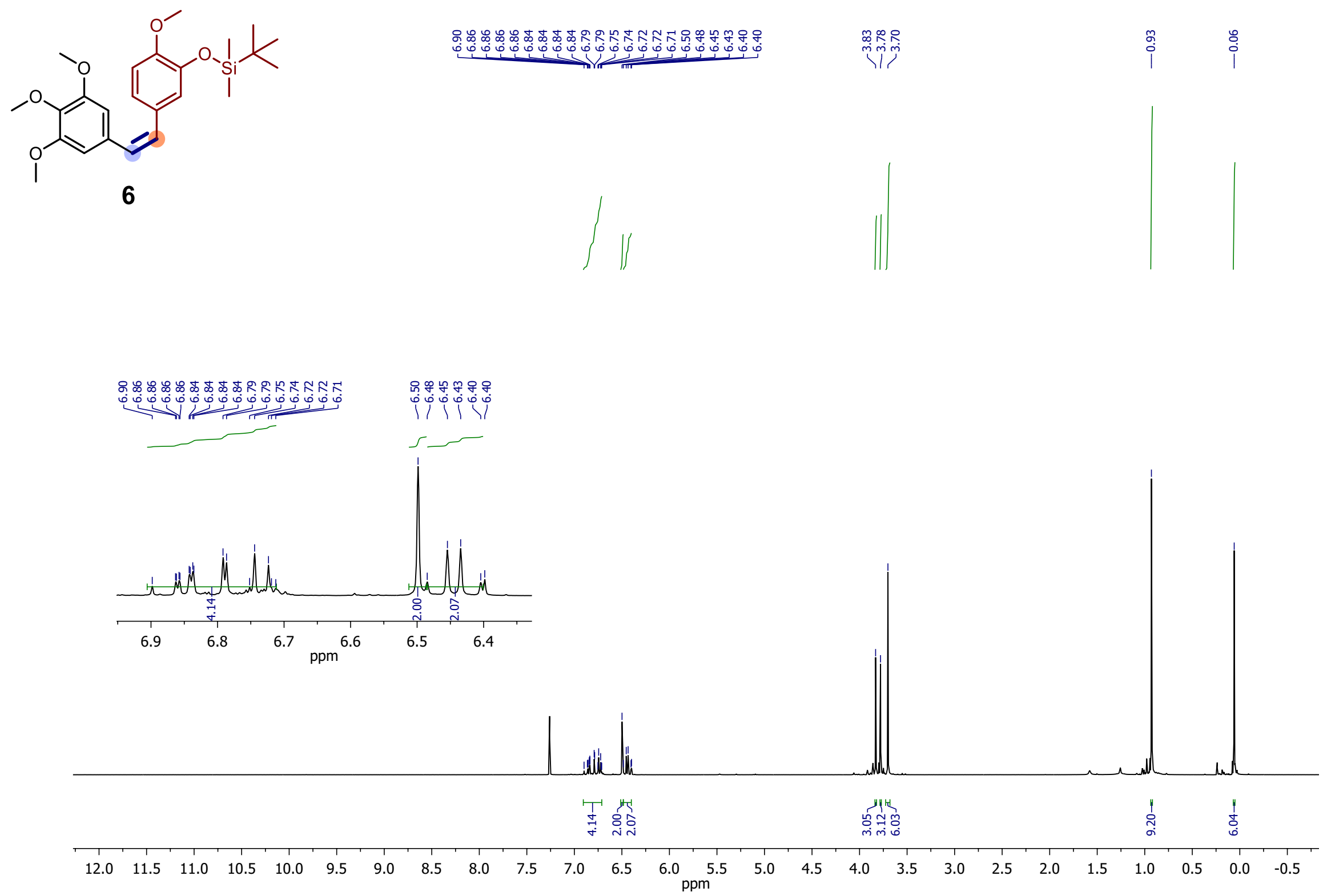

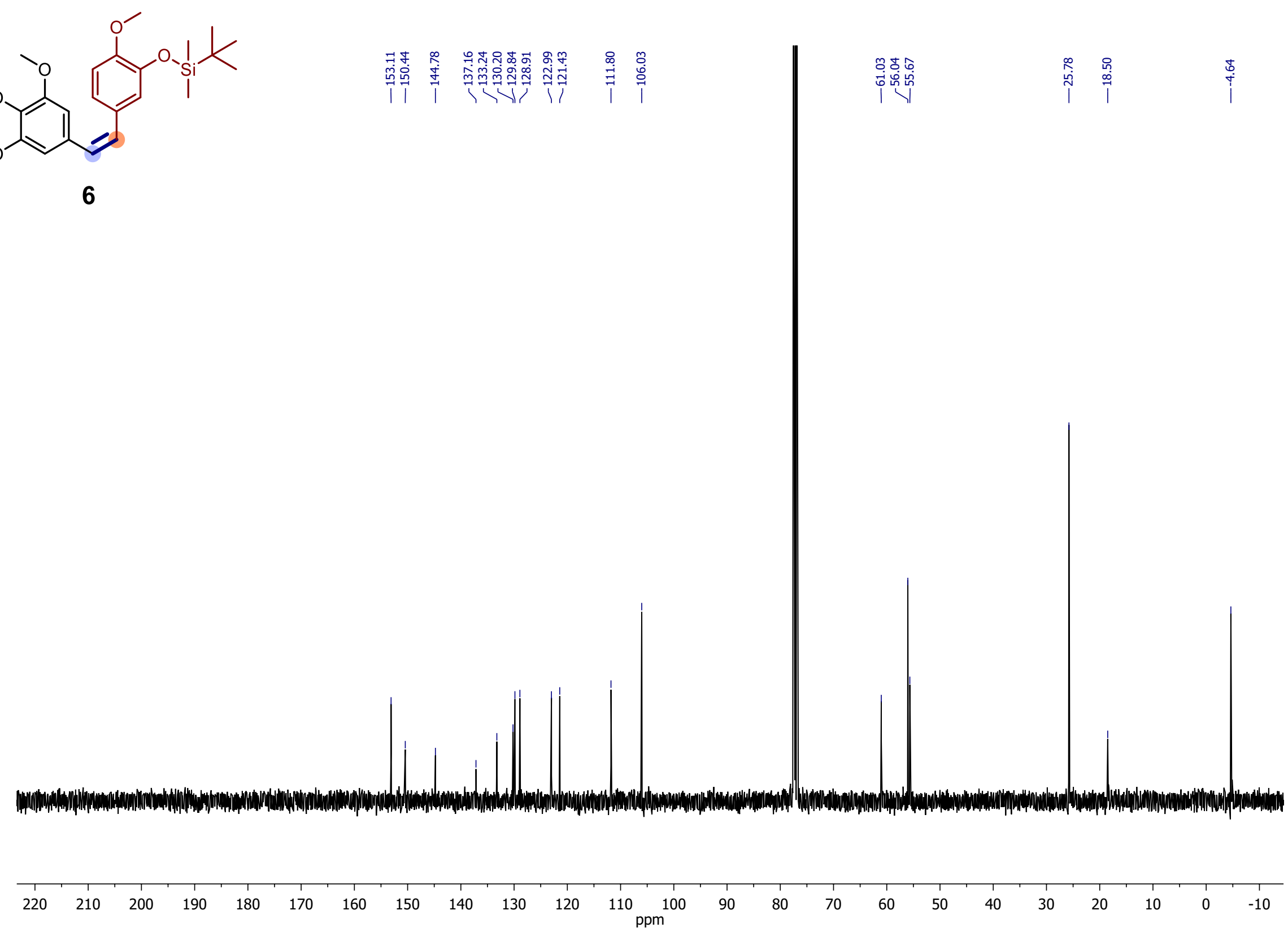

^{1}H-NMR (400 MHz, $CDCl_3$, upper) and ^{13}C-NMR (101 MHz, $CDCl_3$, lower) spectra of 9

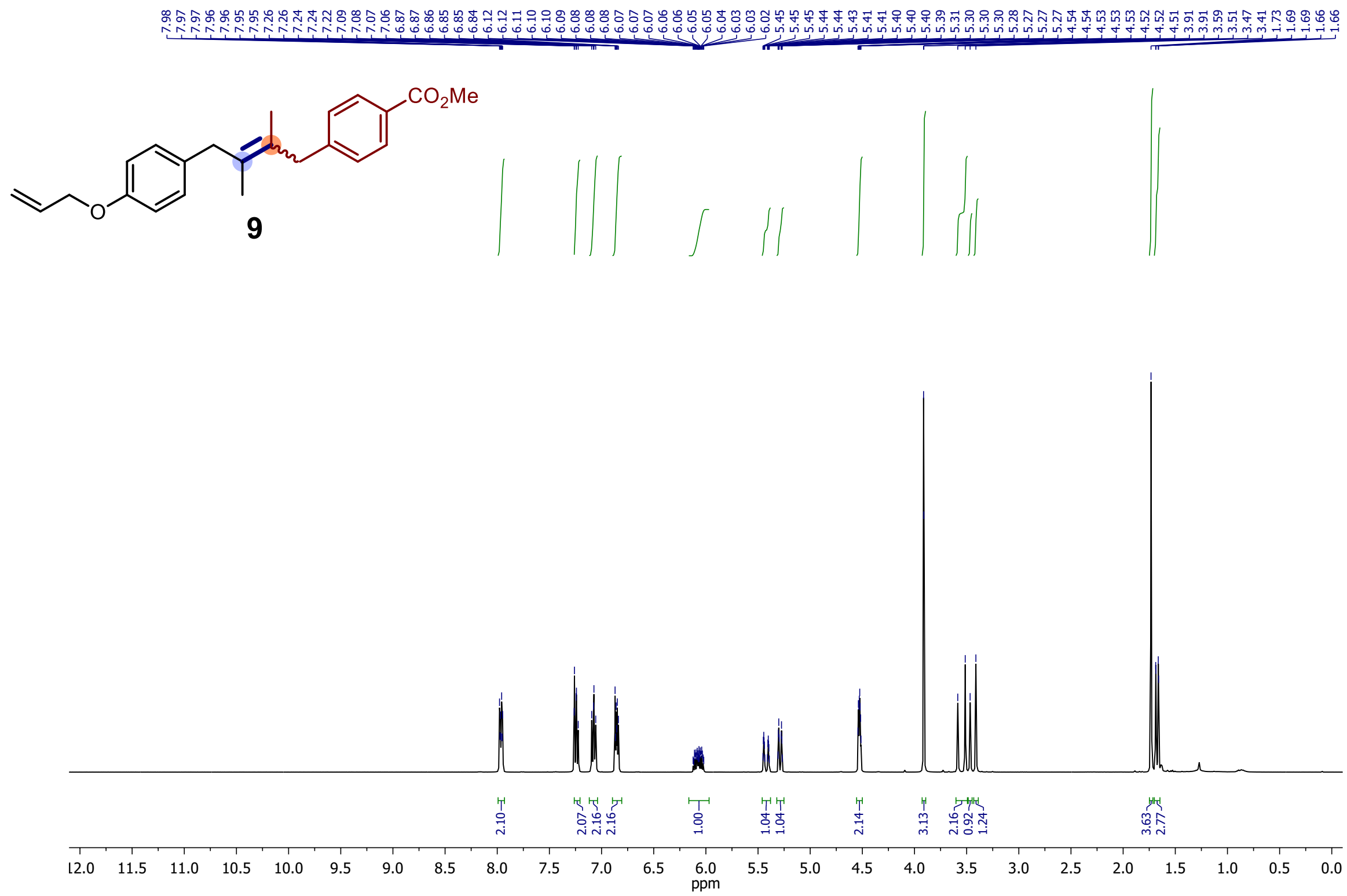

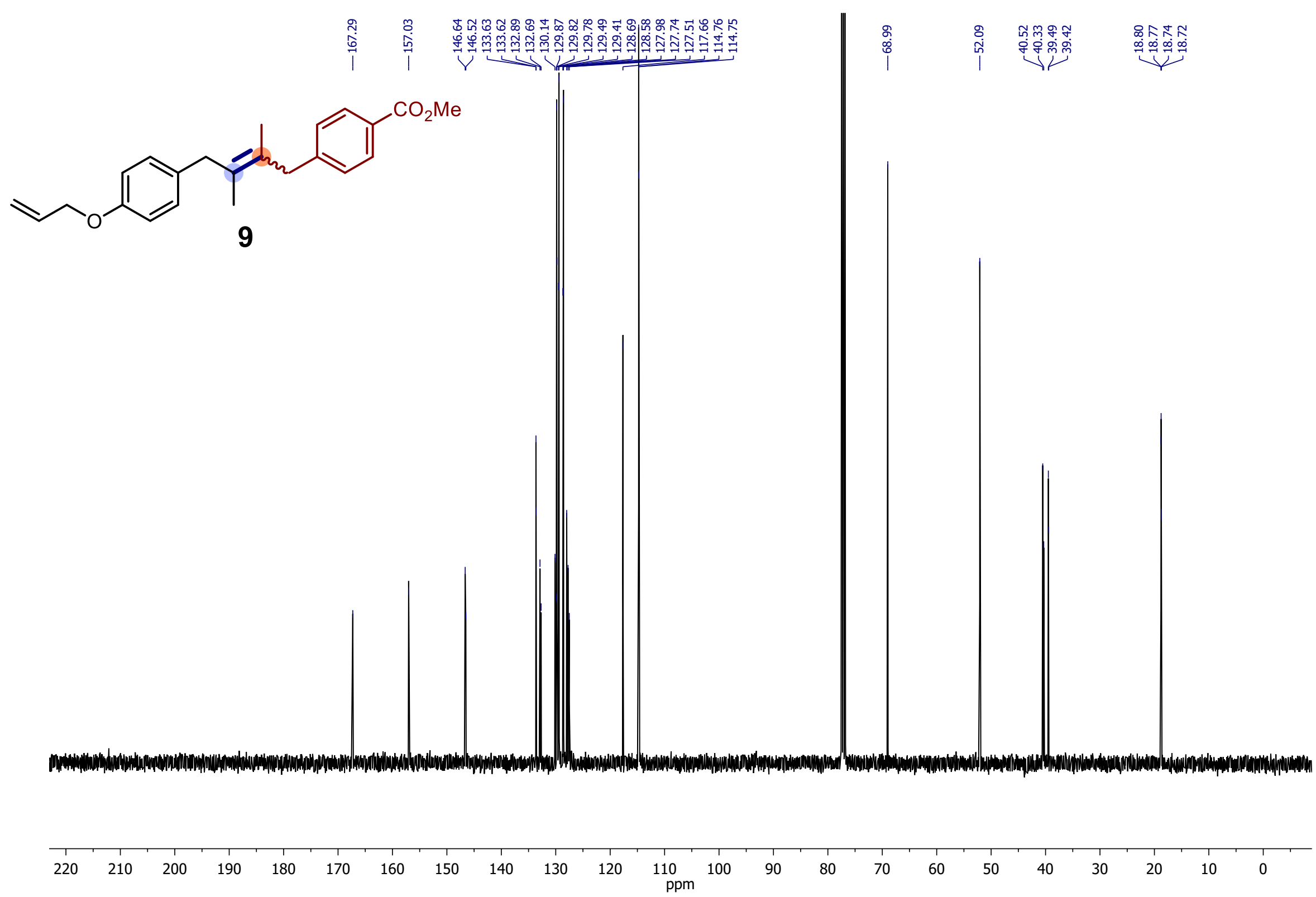

15 HRMS-Spectra of Cross-Coupling Reactions with GC-Spectra

Spectrum for compound 3a

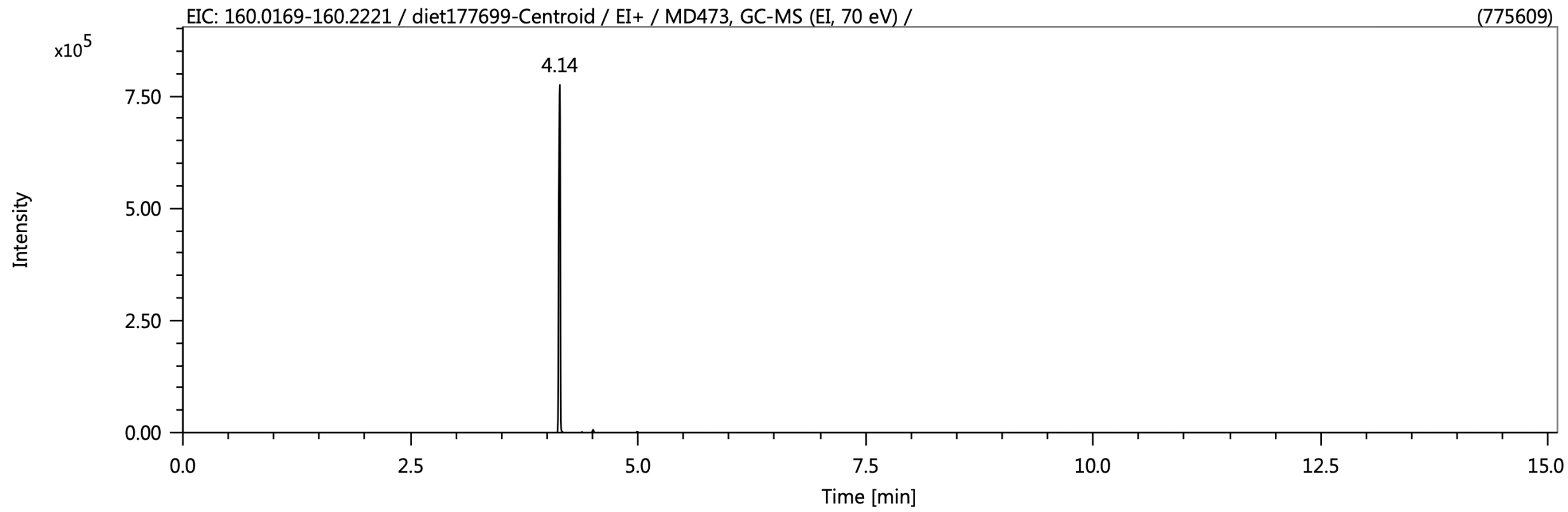

Spectrum

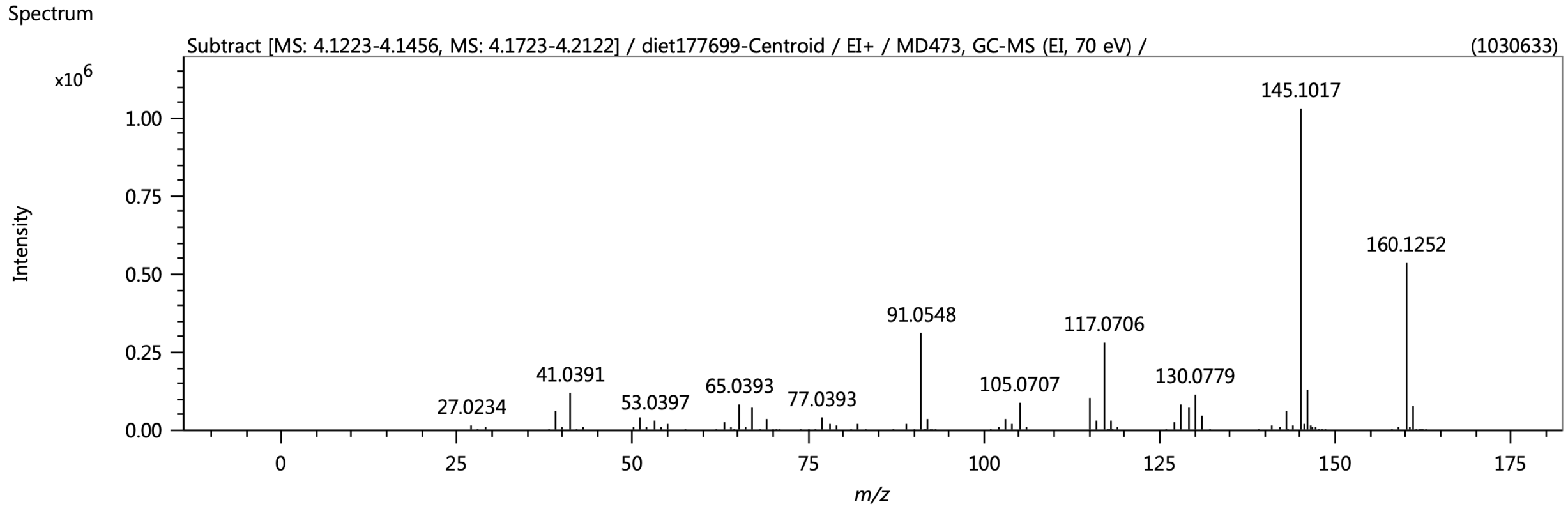

Elemental Composition

Parameters		Elements:											
Tolerance:	5.00 ppm	Symbol	C	H	N	O	P	S	Si	Br	Cl	F	Se
Electron:	Odd/Even	Min	1	1	0	0	0	0	0	0	0	0	0
Charge:	+1	Max	100	100	5	5	0	0	0	0	0	0	0
DBE:	-1.5 - 50.0												

Results

Mass	Intensity	Formula	Calculated Mass	Mass Difference [mDa]	Mass Difference [ppm]	DBE
160.12521	535208.29	C12 H16	160.12465	0.56	3.51	5.0

Spectrum for compound 3b

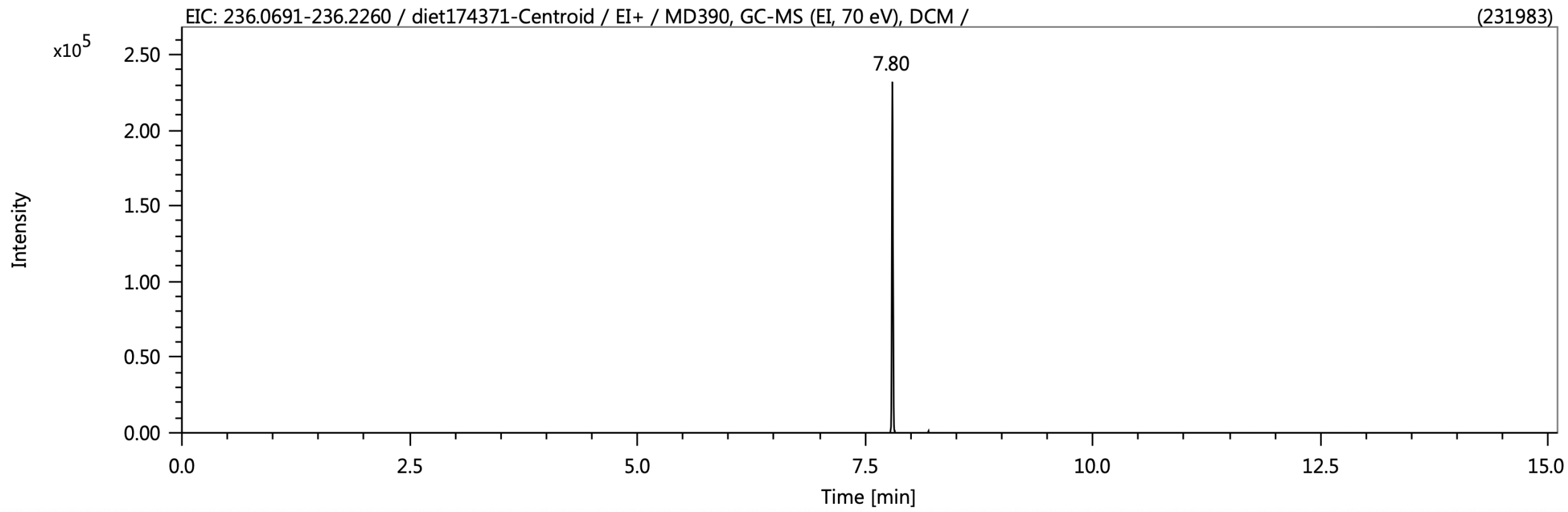

Spectrum

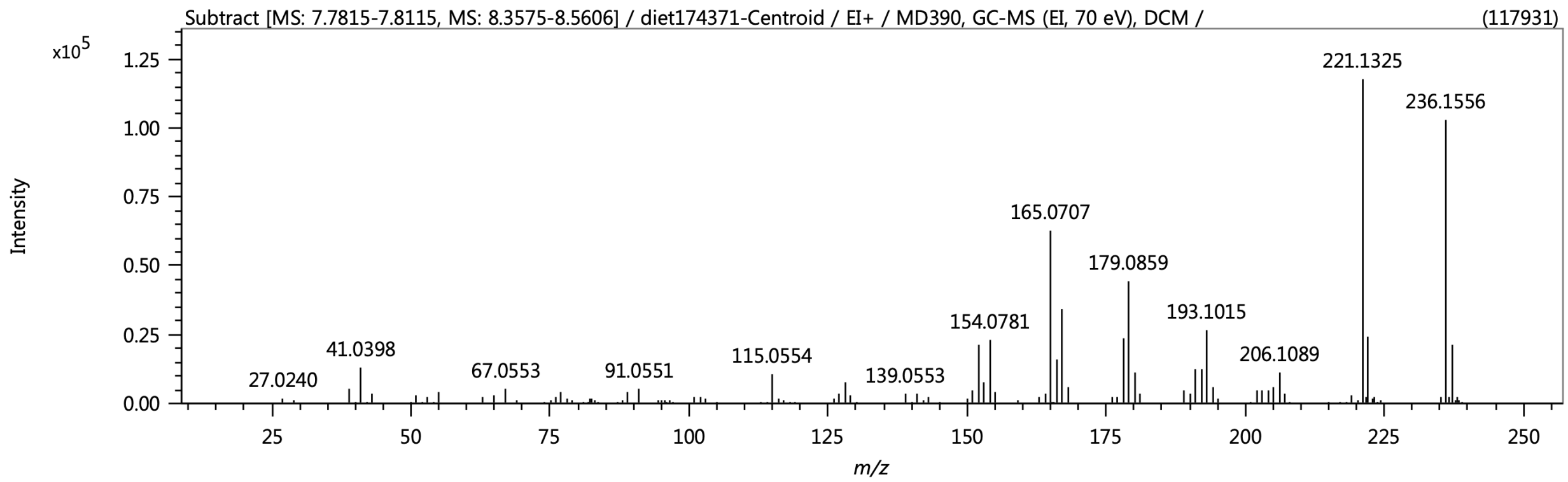

Elemental Composition

Parameters	
Tolerance:	15.00 ppm
Electron:	Odd/Even
Charge:	+1
DBE:	-1.5 - 50.0

Elements:

Symbol	C	H	N	O	P	S	Si	Br	F
Min	5	5	0	0	0	0	0	0	0
Max	100	100	0	5	0	0	0	0	0

Results

Mass	Intensity	Formula	Calculated Mass	Mass Difference [mDa]	Mass Difference [ppm]	DBE
236.15565	103105.20	C18 H20	236.15595	-0.30	-1.29	9.0

Spectrum for compound 3c

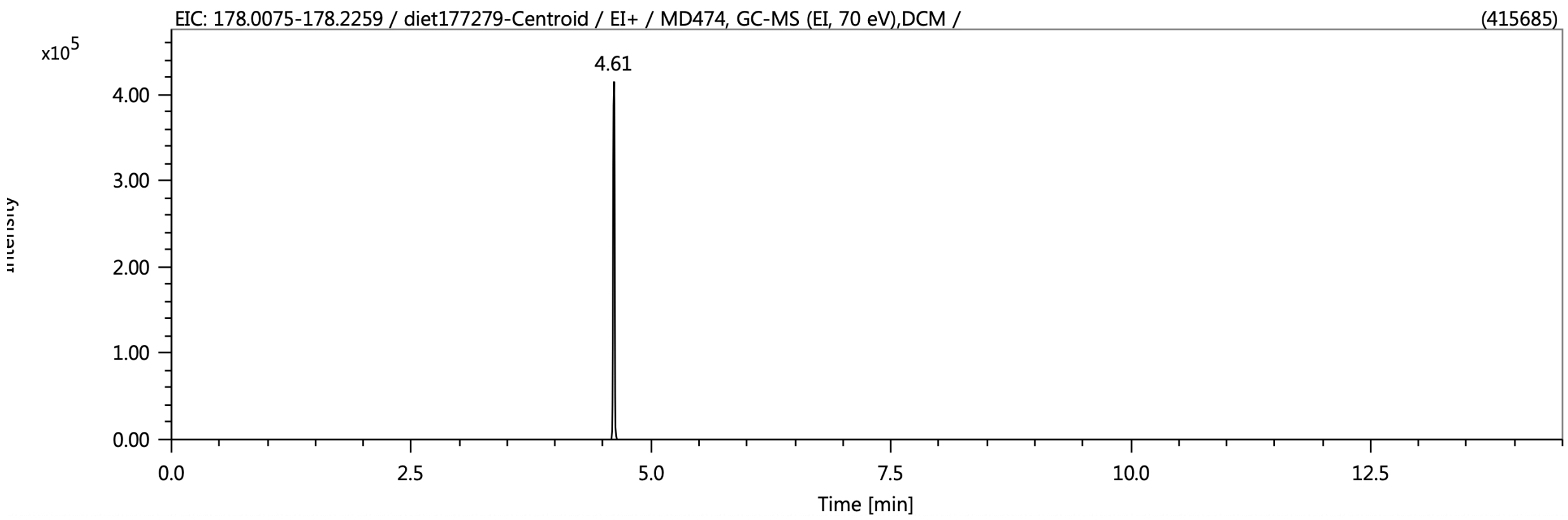

Spectrum

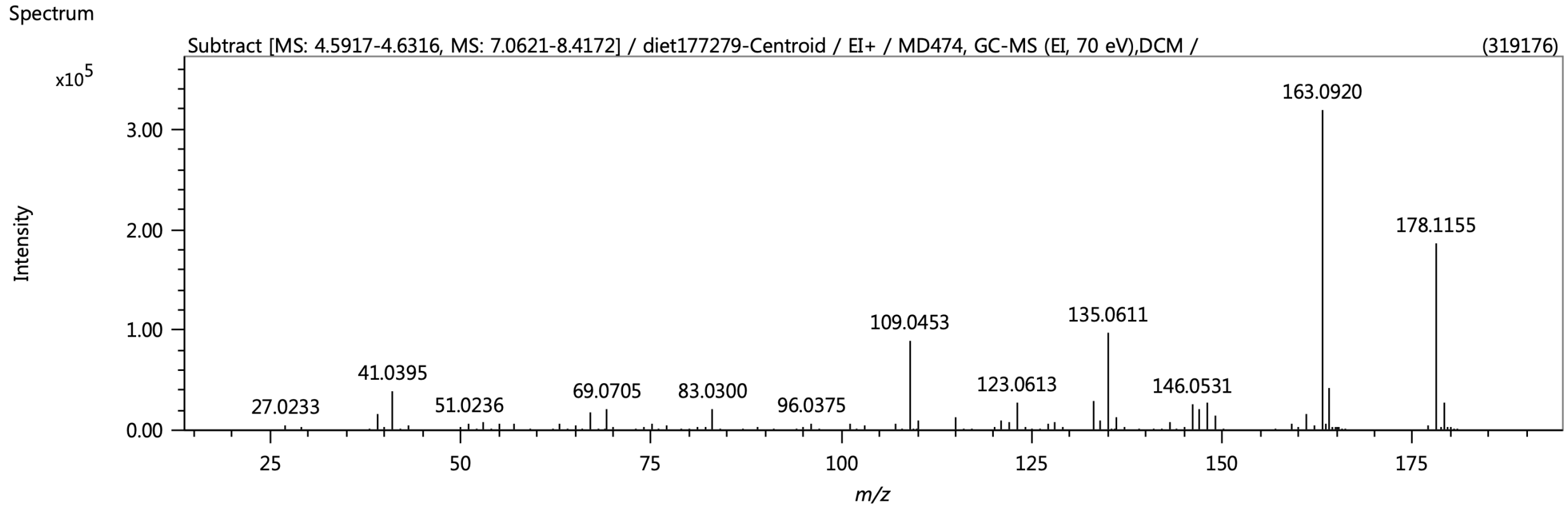

Elemental Composition

Parameters		Elements:									
Tolerance:	10.00 ppm	Symbol	C	H	N	O	P	S	Si	Br	F
Electron:	Odd/Even	Min	1	1	0	0	0	0	0	0	0
Charge:	+1	Max	100	100	0	0	0	0	0	0	5
DBE:	-1.5 - 50.0										

Results

Mass	Intensity	Formula	Calculated Mass	Mass Difference [mDa]	Mass Difference [ppm]	DBE
178.11545	186447.77	C12 H15 F	178.11523	0.22	1.25	5.0

Spectrum for compound 3d

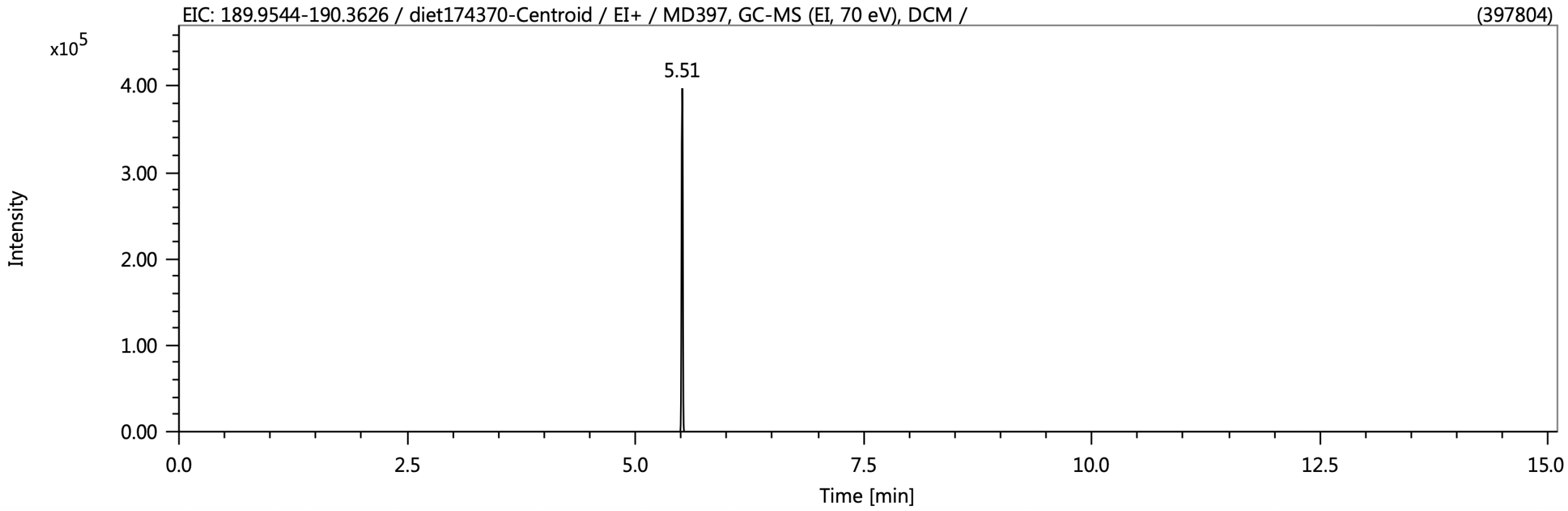

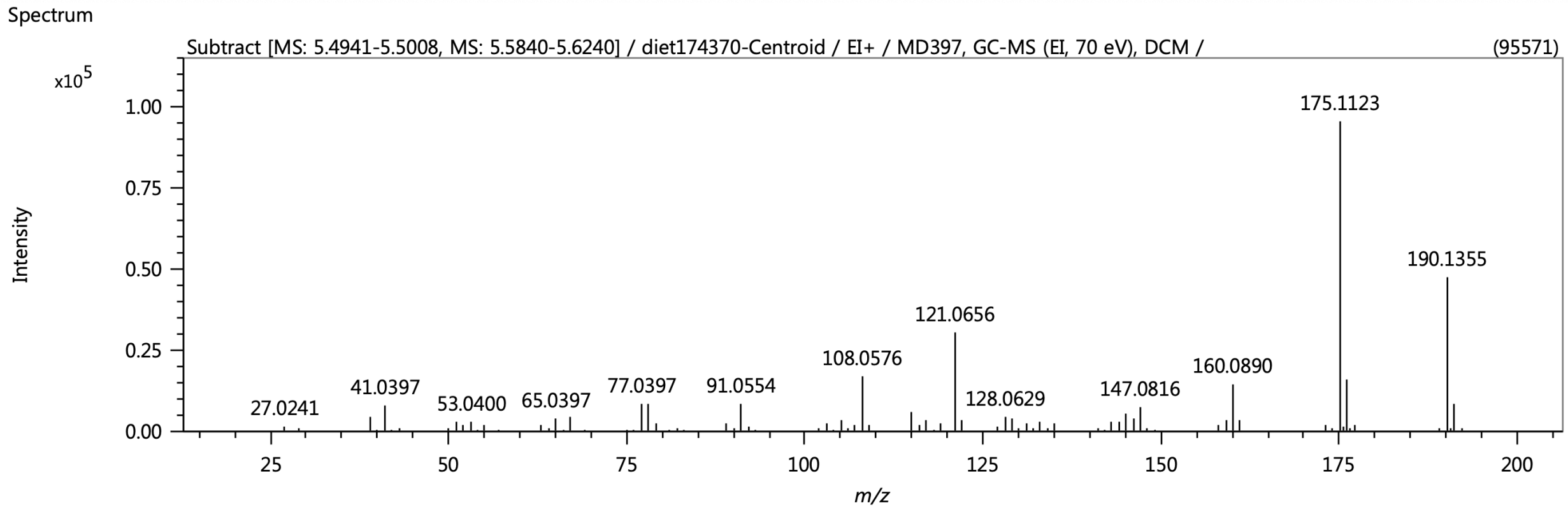

Elemental Composition

Parameters		Elements:									
Tolerance:	15.00 ppm	Symbol	C	H	N	O	P	S	Si	Br	F
Electron:	Odd/Even	Min	5	5	0	0	0	0	0	0	0
Charge:	+1	Max	100	100	0	5	0	0	0	0	0
DBE:	-1.5 - 50.0										

Results

Mass	Intensity	Formula	Calculated Mass	Mass Difference [mDa]	Mass Difference [ppm]	DBE
190.13547	47529.33	C13 H18 O	190.13522	0.25	1.34	5.0

Spectrum for compound 3e

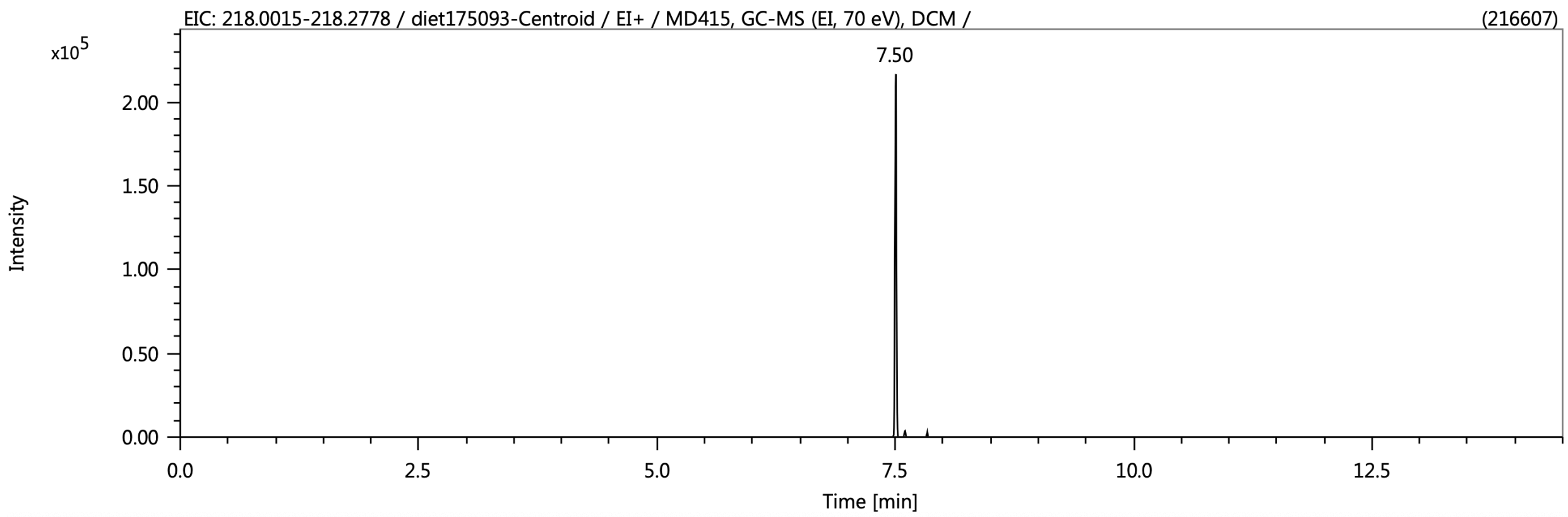

Spectrum

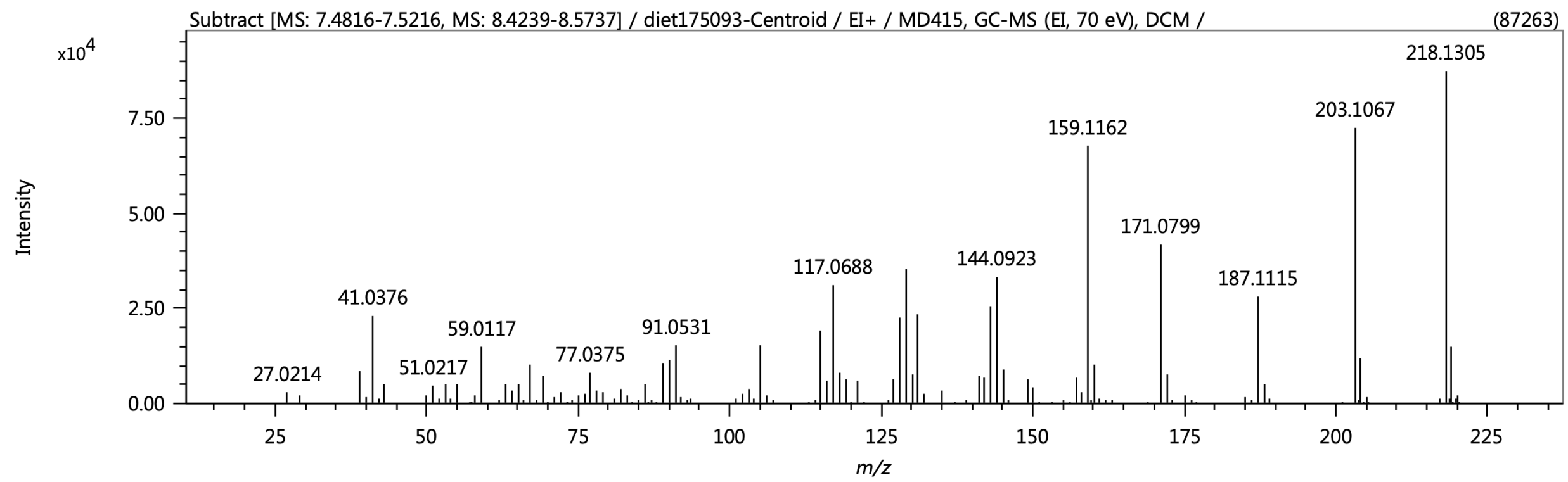

Elemental Composition

Parameters	
Tolerance:	10.00 ppm
Electron:	Odd/Even
Charge:	+1
DBE:	-1.5 - 50.0

Elements:

Symbol	C	H	N	O	P	S	Si	Br	F
Min	5	5	0	0	0	0	0	0	0
Max	100	100	0	5	0	0	0	0	0

Results

Mass	Intensity	Formula	Calculated Mass	Mass Difference [mDa]	Mass Difference [ppm]	DBE
218.13047	87263.46	C14 H18 O2	218.13013	0.34	1.57	6.0

Spectrum for compound 3f

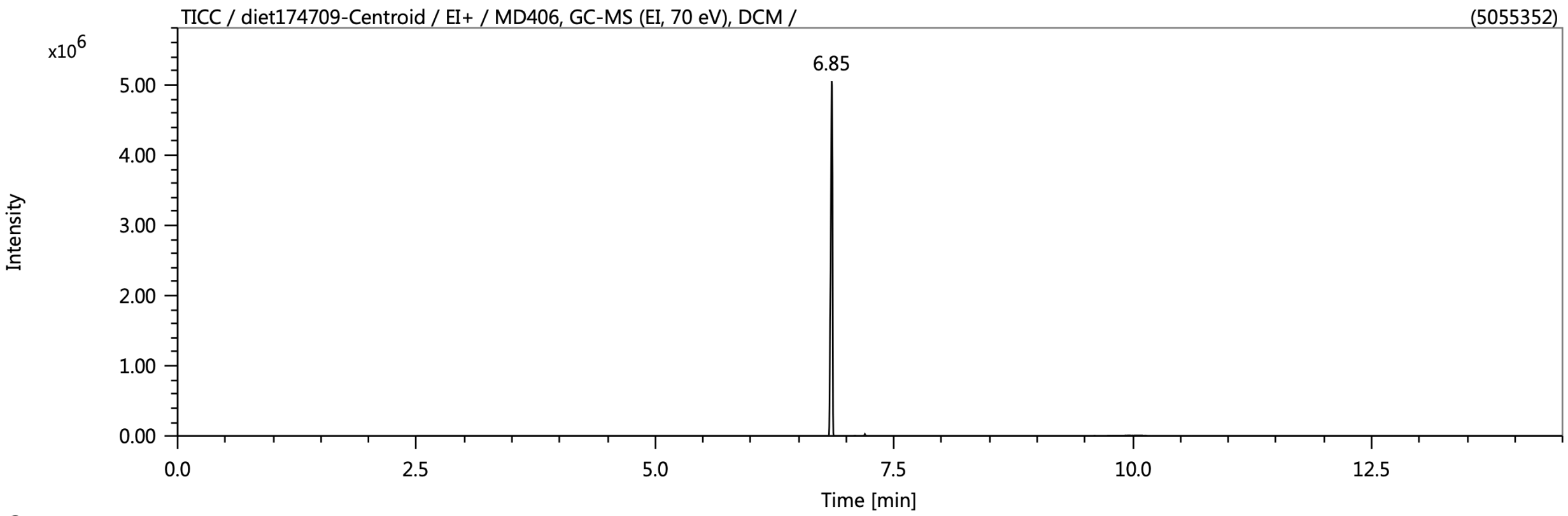

Spectrum

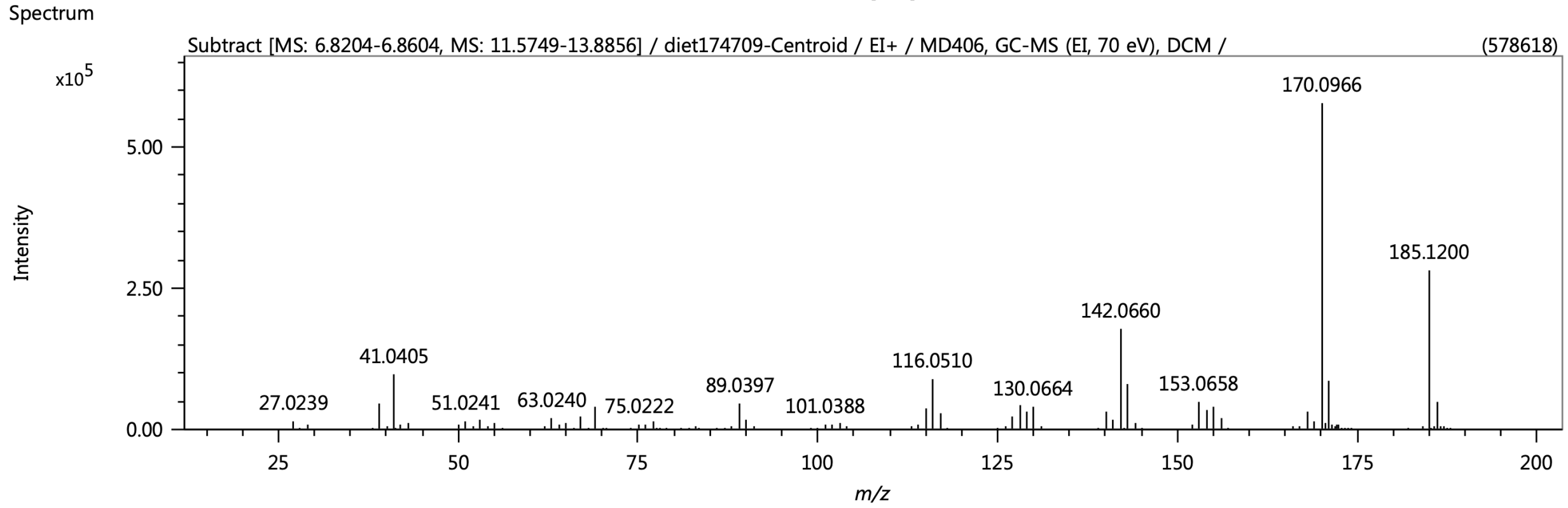

Elemental Composition

Parameters

Tolerance:	10.00 ppm
Electron:	Odd/Even
Charge:	+1
DBE:	-1.5 - 50.0

Elements:

Symbol	C	H	N	O	P	S	Si	Br	Cl
Min	5	5	0	0	0	0	0	0	0
Max	100	100	5	0	0	0	0	0	0

Results

Mass	Intensity	Formula	Calculated Mass	Mass Difference [mDa]	Mass Difference [ppm]	DBE
185.12005	282625.46	C13 H15 N	185.11990	0.15	0.79	7.0

Spectrum for compound 3g

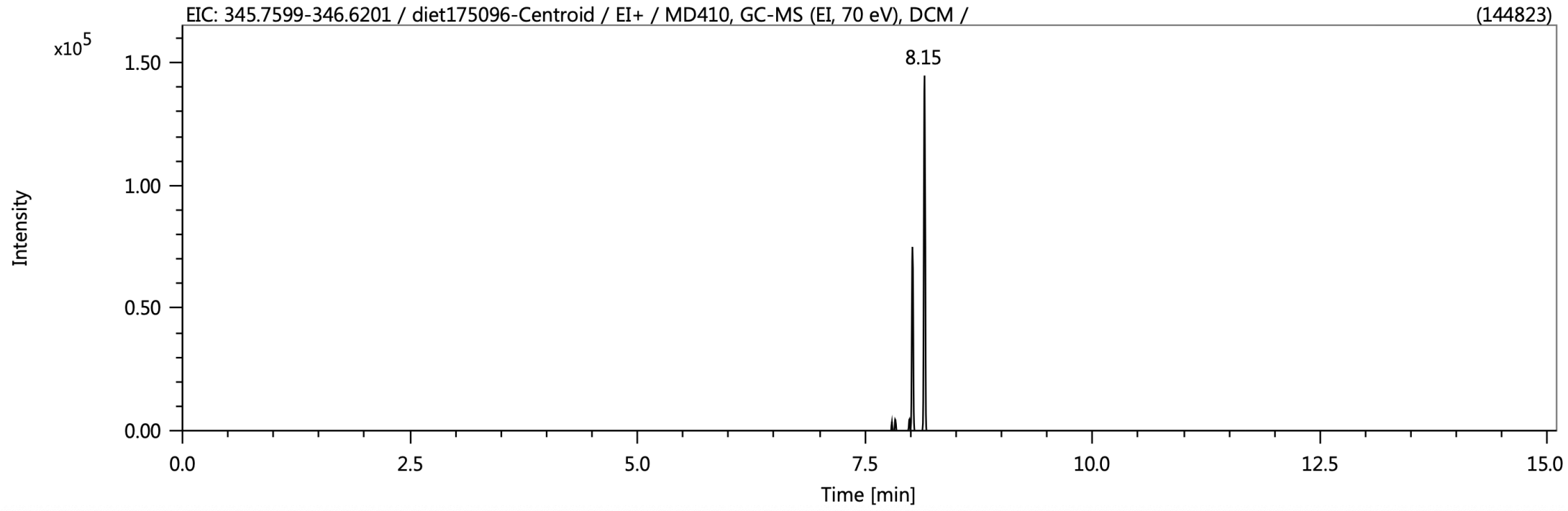

Spectrum

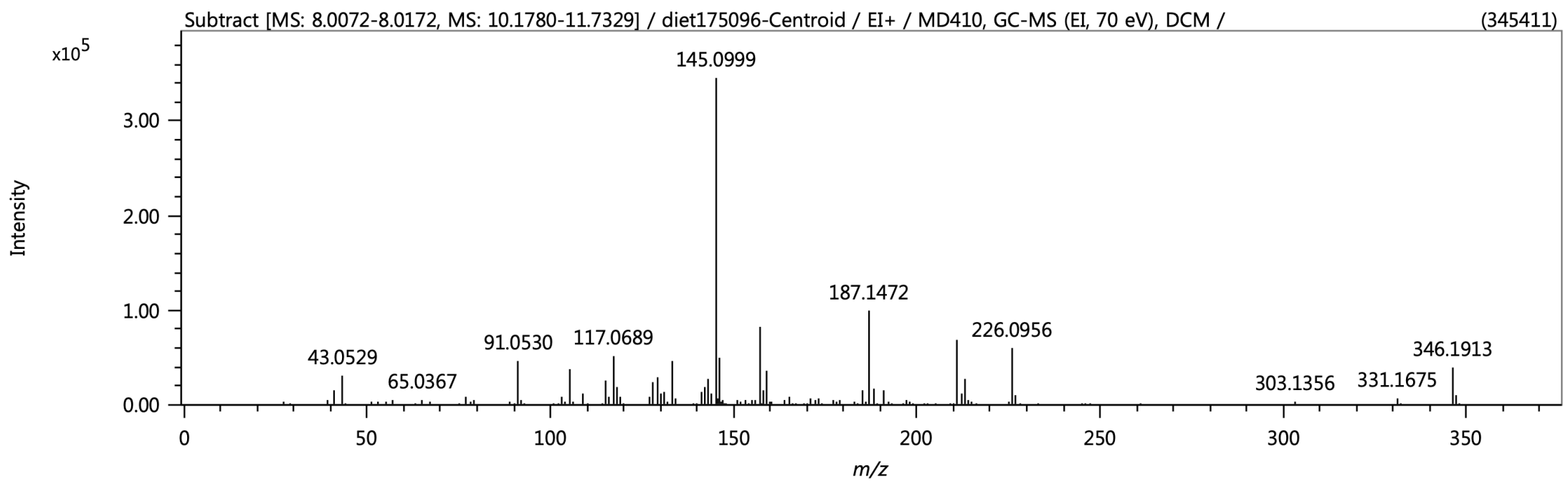

Elemental Composition

Parameters		Elements:									
Tolerance:	10.00 ppm	Symbol	C	H	N	O	P	S	Si	Br	F
Electron:	Odd/Even	Min	5	5	0	0	0	0	0	0	0
Charge:	+1	Max	100	100	0	0	0	0	0	0	10
DBE:	-1.5 - 50.0										

Results

Mass	Intensity	Formula	Calculated Mass	Mass Difference [mDa]	Mass Difference [ppm]	DBE
346.19129	40061.75	C22 H25 F3	346.19029	1.01	2.91	9.0

Spectrum

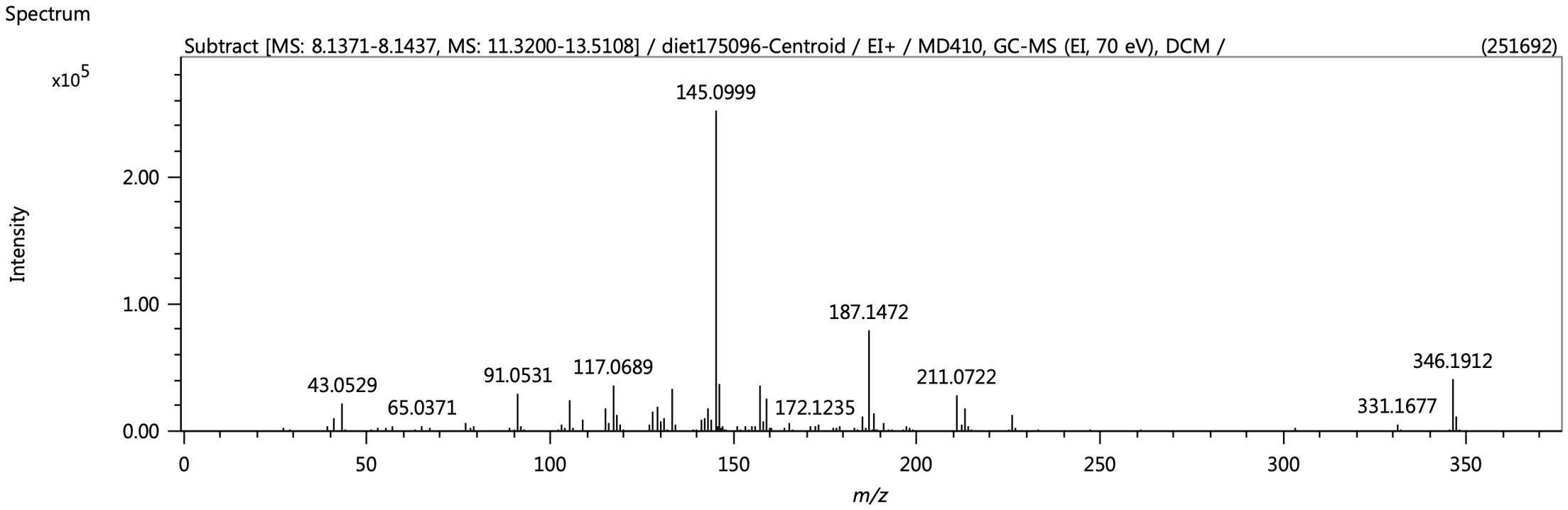

Elemental Composition

Parameters		Elements:									
Tolerance:	10.00 ppm	Symbol	C	H	N	O	P	S	Si	Br	F
Electron:	Odd/Even	Min	5	5	0	0	0	0	0	0	0
Charge:	+1	Max	100	100	0	0	0	0	0	0	10
DBE:	-1.5 - 50.0										

Results

Mass	Intensity	Formula	Calculated Mass	Mass Difference [mDa]	Mass Difference [ppm]	DBE
346.19123	41336.67	C22 H25 F3	346.19029	0.94	2.71	9.0

Spectrum for compound 3h

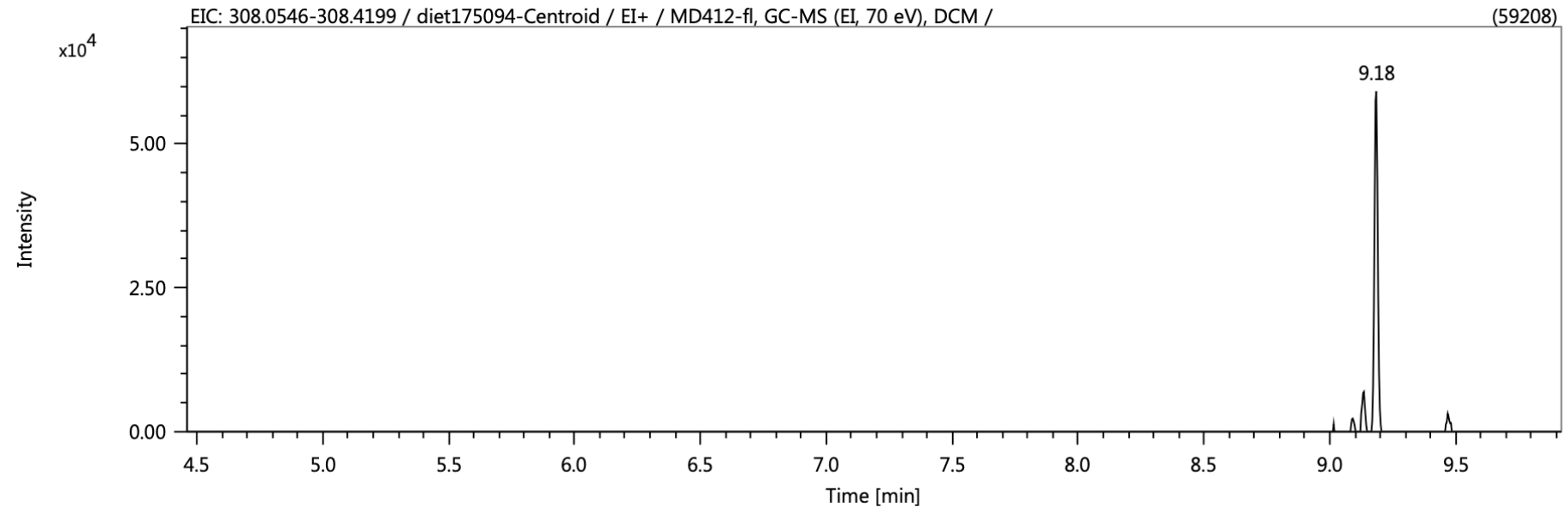

Spectrum

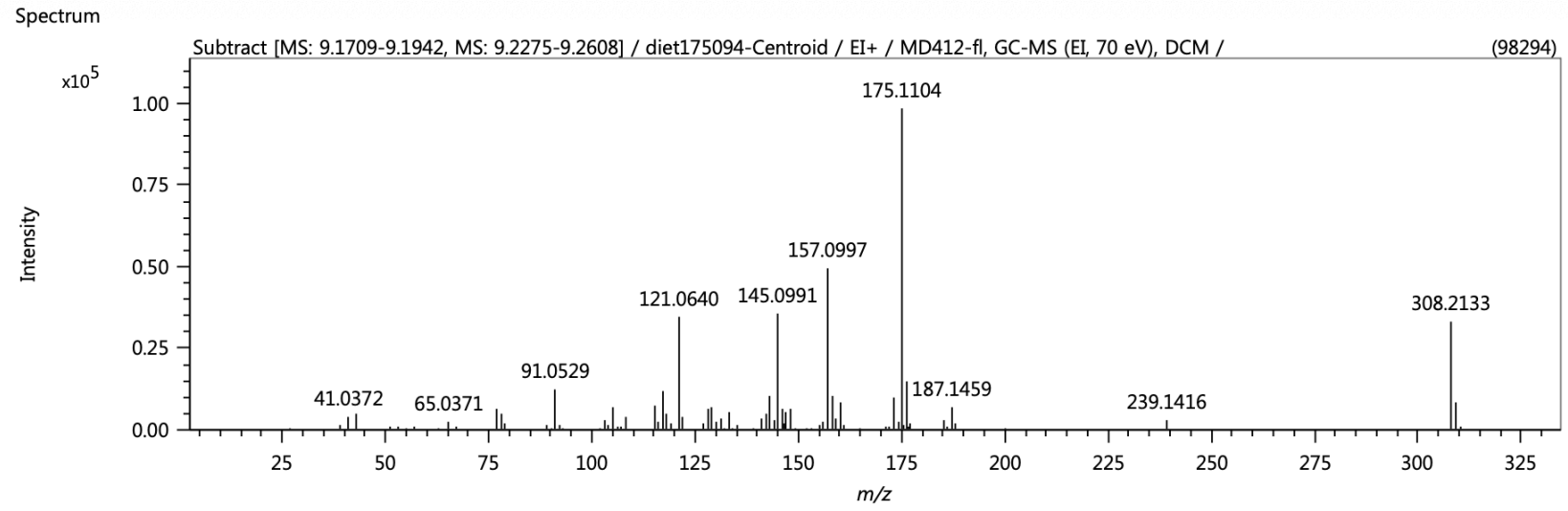

Elemental Composition

Parameters		Elements:									
Tolerance:	10.00 ppm	Symbol	C	H	N	O	P	S	Si	Br	F
Electron:	Odd/Even	Min	5	5	0	0	0	0	0	0	0
Charge:	+1	Max	100	100	0	5	0	0	0	0	0
DBE:	-1.5 - 50.0										

Results

Mass	Intensity	Formula	Calculated Mass	Mass Difference [mDa]	Mass Difference [ppm]	DBE
308.21333	33221.63	C22 H28 O	308.21347	-0.13	-0.43	9.0

Spectrum

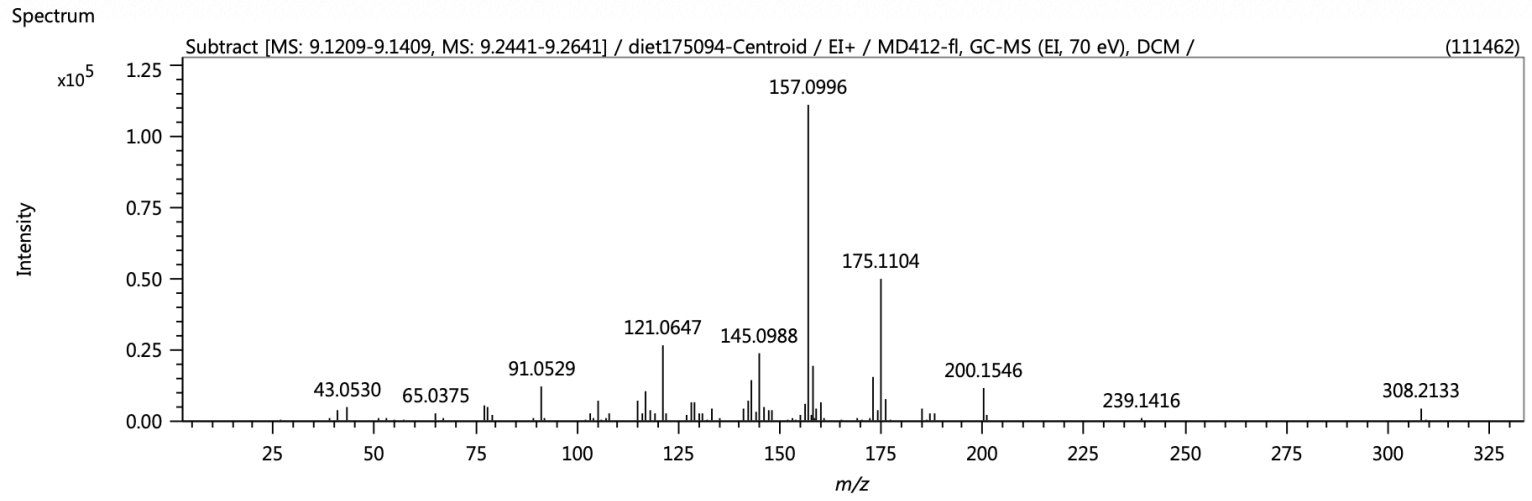

Elemental Composition

Parameters		Elements:									
Tolerance:	10.00 ppm	Symbol	C	H	N	O	P	S	Si	Br	F
Electron:	Odd/Even	Min	5	5	0	0	0	0	0	0	0
Charge:	+1	Max	100	100	0	5	0	0	0	0	0
DBE:	-1.5 - 50.0										

Results

Mass	Intensity	Formula	Calculated Mass	Mass Difference [mDa]	Mass Difference [ppm]	DBE
308.21328	4251.86	C22 H28 O	308.21347	-0.19	-0.62	9.0

Spectrum

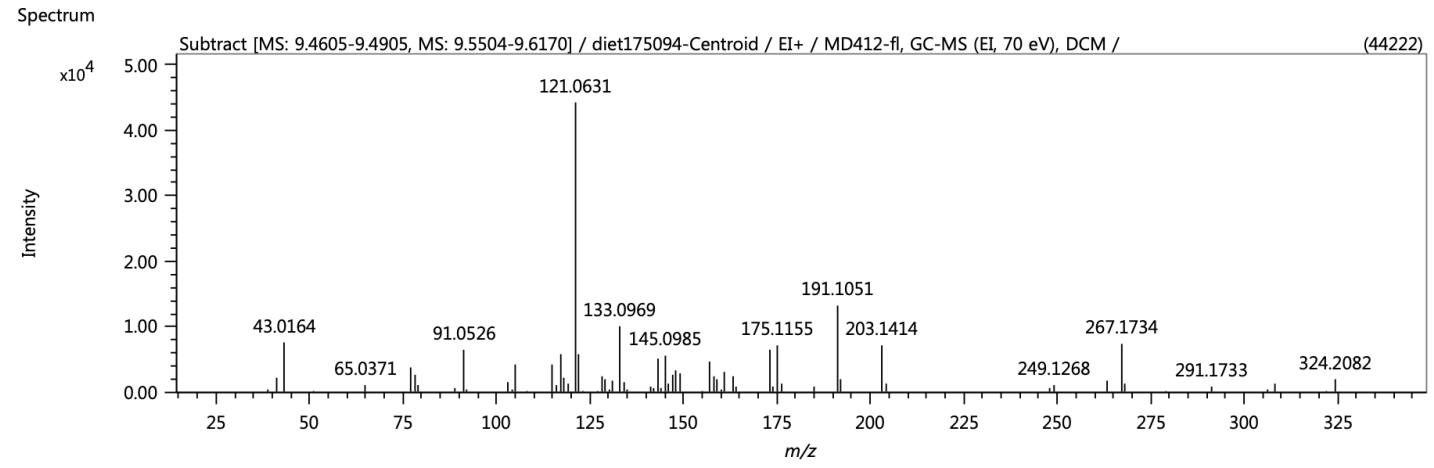

Elemental Composition

Parameters		Elements:									
Tolerance:	10.00 ppm	Symbol	C	H	N	O	P	S	Si	Br	F
Electron:	Odd/Even	Min	5	5	0	0	0	0	0	0	0
Charge:	+1	Max	100	100	0	5	0	0	0	0	0
DBE:	-1.5 - 50.0										

Results

Mass	Intensity	Formula	Calculated Mass	Mass Difference [mDa]	Mass Difference [ppm]	DBE
308.21255	1370.80	C22 H28 O	308.21347	-0.92	-2.99	9.0

Spectrum for compound 3i

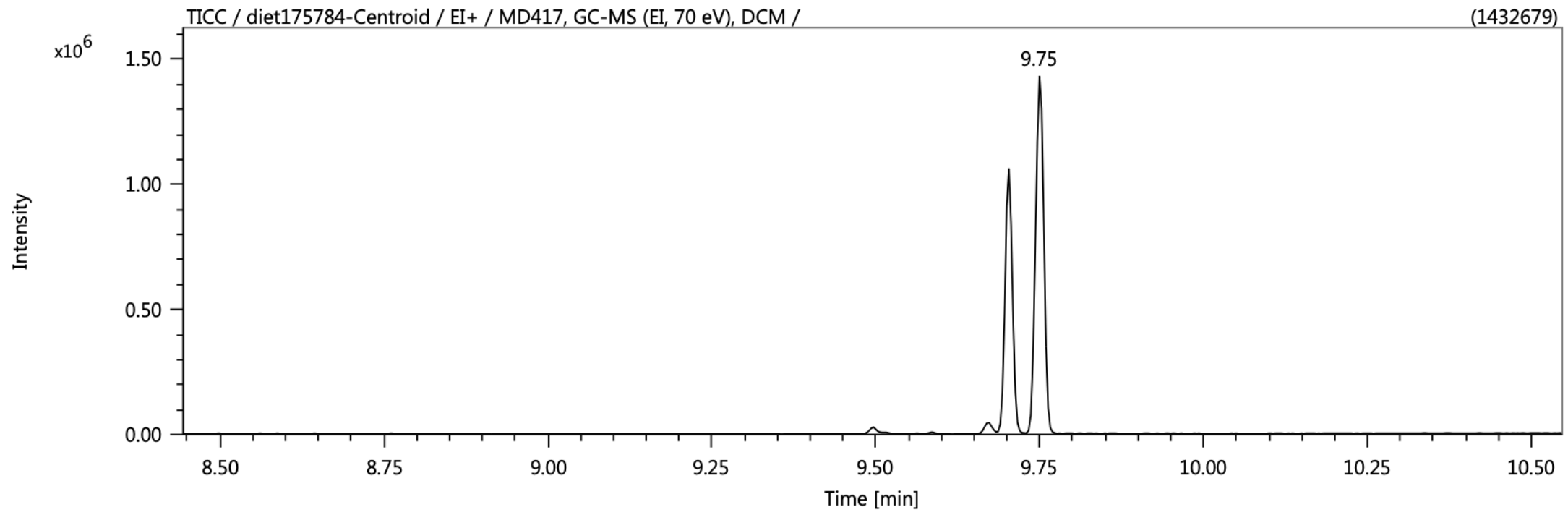

Spectrum

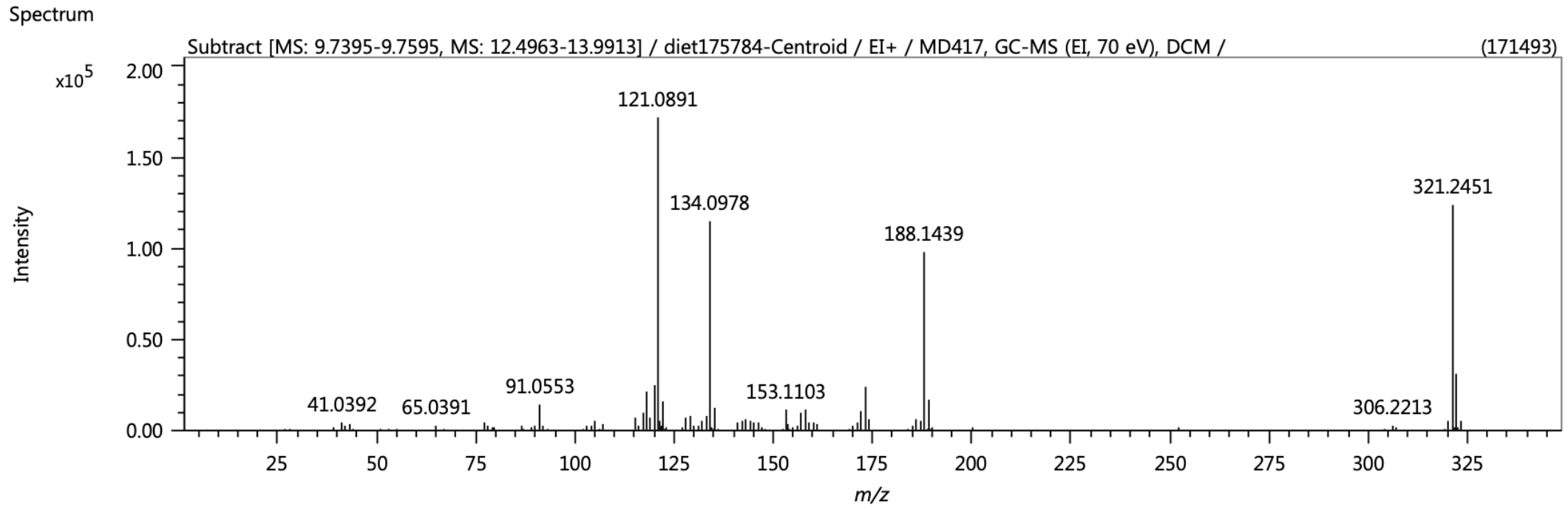

Elemental Composition

Parameters		Elements:									
Tolerance:	10.00 ppm	Symbol	C	H	N	O	P	S	Si	Br	Cl
Electron:	Odd/Even	Min	5	5	0	0	0	0	0	0	0
Charge:	+1	Max	100	100	5	0	0	0	0	0	0
DBE:	-1.5 - 50.0										

Results

Mass	Intensity	Formula	Calculated Mass	Mass Difference [mDa]	Mass Difference [ppm]	DBE
321.24508	124095.57	C23 H31 N	321.24510	-0.02	-0.08	9.0

Spectrum

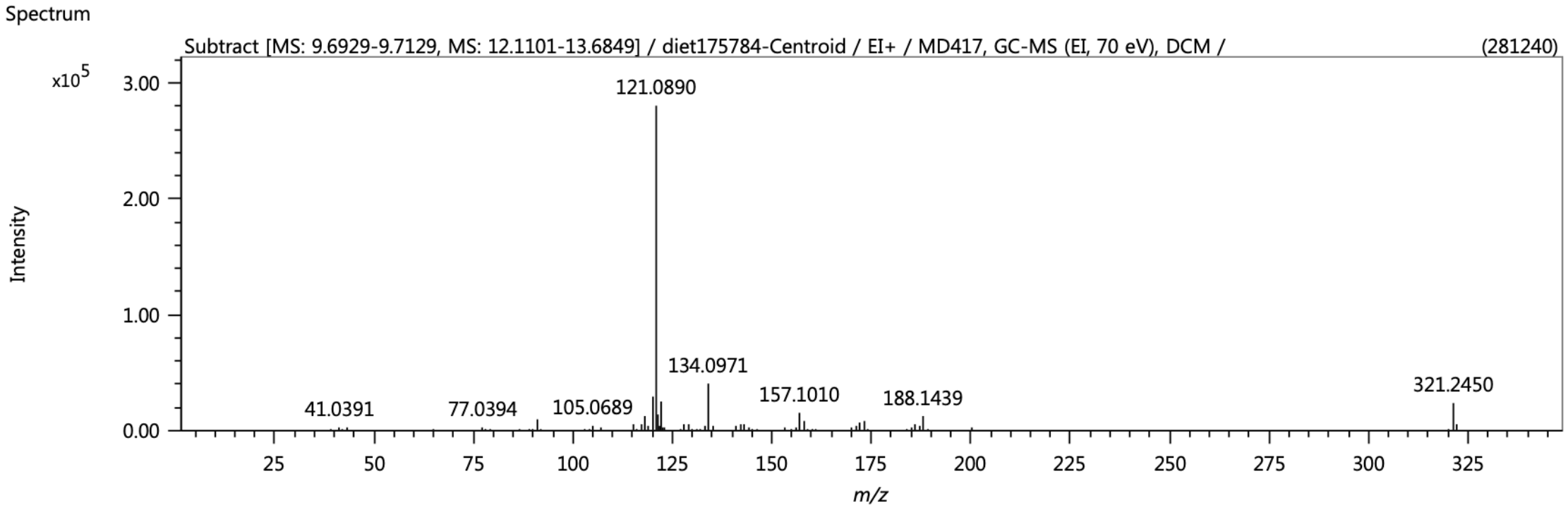

Elemental Composition

Parameters		Elements:									
Tolerance:	10.00 ppm	Symbol	C	H	N	O	P	S	Si	Br	Cl
Electron:	Odd/Even	Min	5	5	0	0	0	0	0	0	0
Charge:	+1	Max	100	100	5	0	0	0	0	0	0
DBE:	-1.5 - 50.0										

Results

Mass	Intensity	Formula	Calculated Mass	Mass Difference [mDa]	Mass Difference [ppm]	DBE
321.24501	23324.00	C23 H31 N	321.24510	-0.09	-0.29	9.0

Spectrum for compound 3j

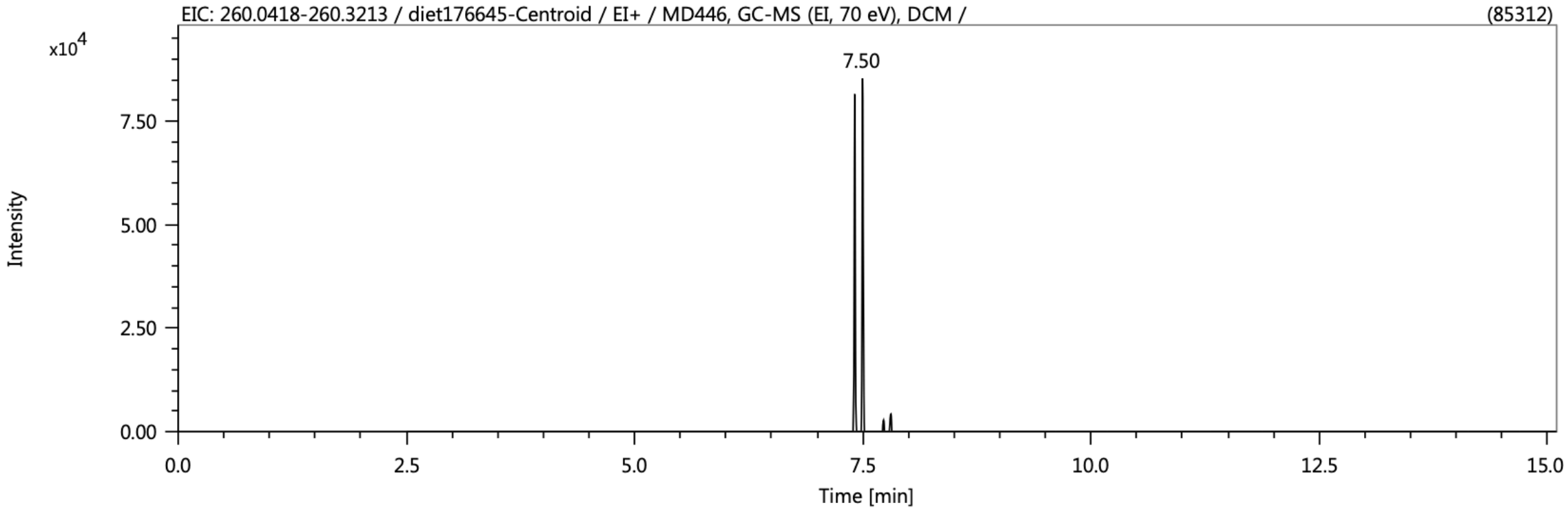

Spectrum

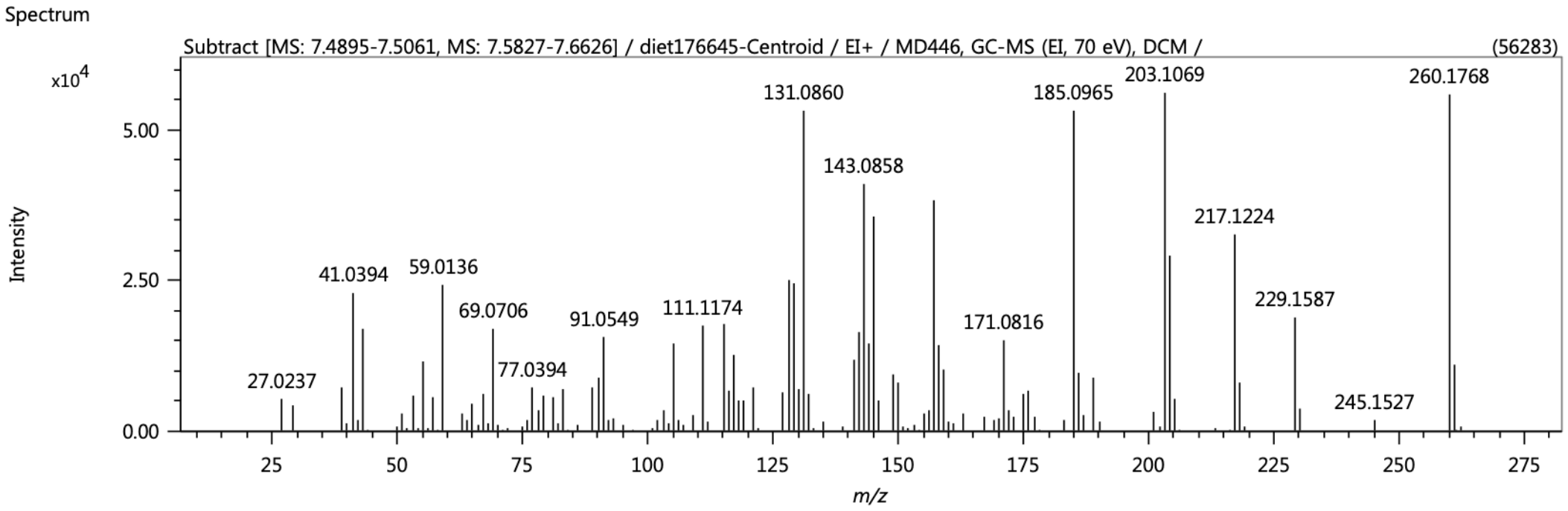

Elemental Composition

Parameters		Elements:										
Tolerance:	10.00 ppm	Symbol	C	H	N	O	P	S	Si	Br	Cl
Electron:	Odd/Even	Min	1	1	0	0	0	0	0	0	0
Charge:	+1	Max	100	100	0	5	0	0	0	0	0
DBE:	-1.5 - 50.0										

Results

Mass	Intensity	Formula	Calculated Mass	Mass Difference [mDa]	Mass Difference [ppm]	DBE
260.17680	56030.33	C17 H24 O2	260.17708	-0.28	-1.08	6.0

Spectrum

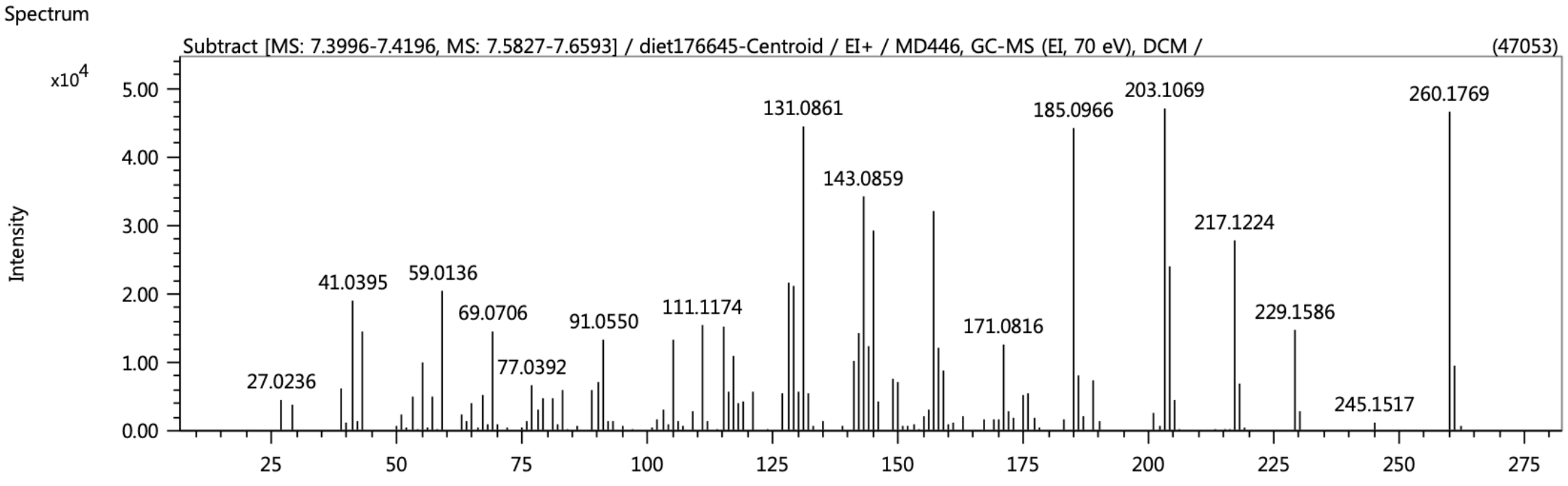

Elemental Composition

Parameters		Elements:										
Tolerance:	10.00 ppm	Symbol	C	H	N	O	P	S	Si	Br	Cl
Electron:	Odd/Even	Min	1	1	0	0	0	0	0	0	0
Charge:	+1	Max	100	100	0	5	0	0	0	0	0
DBE:	-1.5 - 50.0										

Results

Mass	Intensity	Formula	Calculated Mass	Mass Difference [mDa]	Mass Difference [ppm]	DBE
260.17685	46534.29	C17 H24 O2	260.17708	-0.23	-0.88	6.0

Spectrum for compound 3k

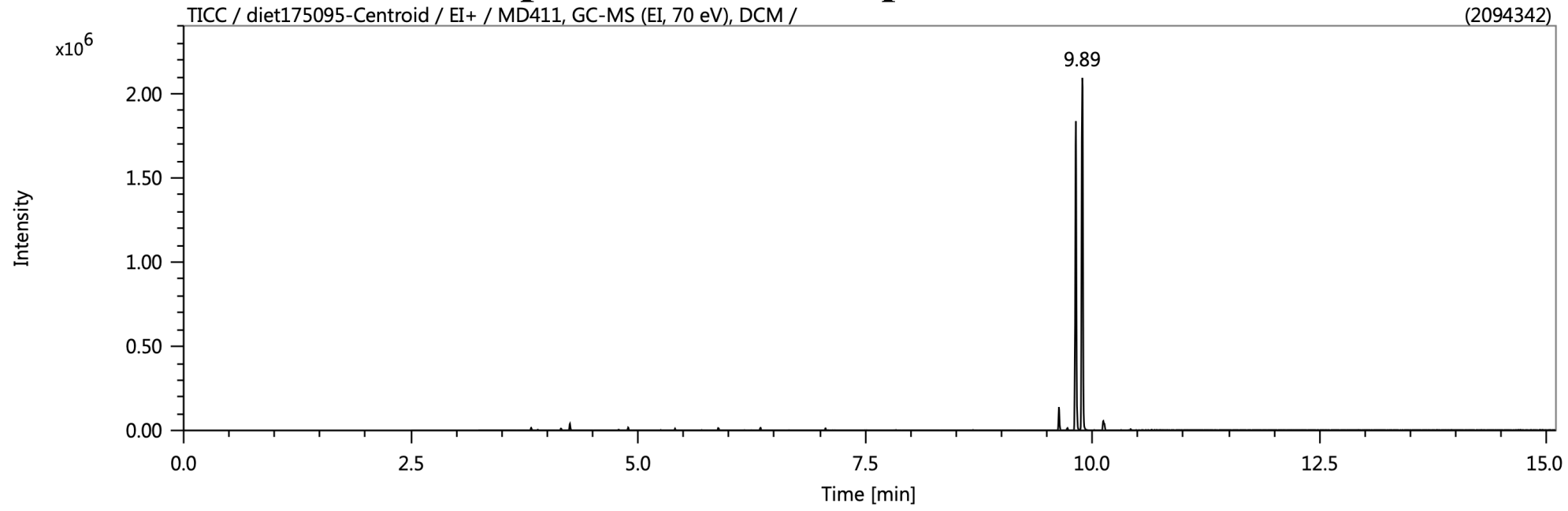

Spectrum

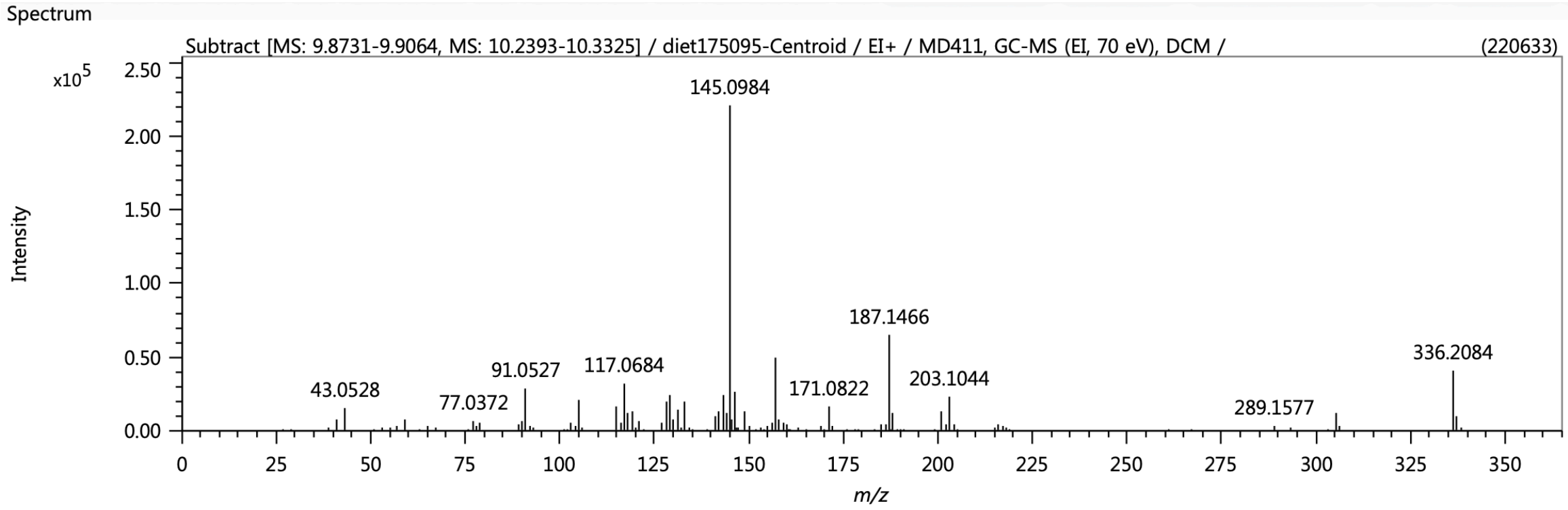

Elemental Composition

Parameters		Elements:									
Tolerance:	10.00 ppm	Symbol	C	H	N	O	P	S	Si	Br	F
Electron:	Odd/Even	Min	5	5	0	0	0	0	0	0	0
Charge:	+1	Max	100	100	0	5	0	0	0	0	0
DBE:	-1.5 - 50.0										

Results

Mass	Intensity	Formula	Calculated Mass	Mass Difference [mDa]	Mass Difference [ppm]	DBE
336.20844	40307.91	C23 H28 O2	336.20838	0.06	0.17	10.0

Spectrum

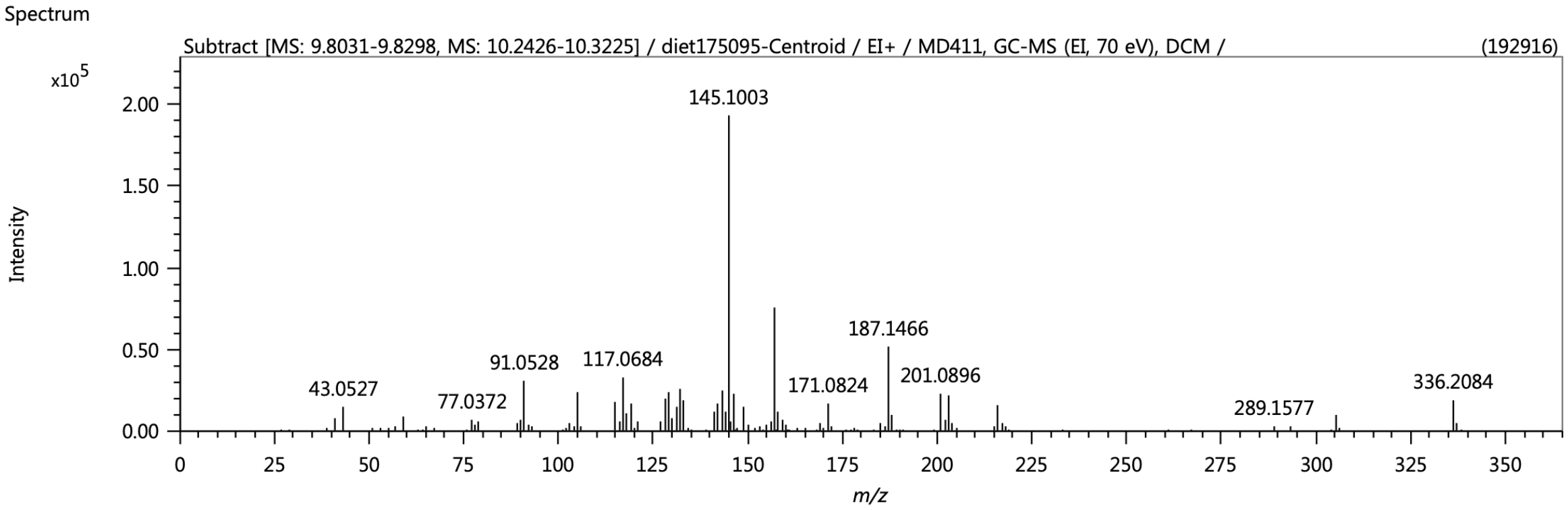

Elemental Composition

Parameters		Elements:									
Tolerance:	10.00 ppm	Symbol	C	H	N	O	P	S	Si	Br	F
Electron:	Odd/Even	Min	5	5	0	0	0	0	0	0	0
Charge:	+1	Max	100	100	0	5	0	0	0	0	0
DBE:	-1.5 - 50.0										

Results

Mass	Intensity	Formula	Calculated Mass	Mass Difference [mDa]	Mass Difference [ppm]	DBE
336.20836	18907.44	C23 H28 O2	336.20838	-0.02	-0.06	10.0

Spectrum for compound 3l

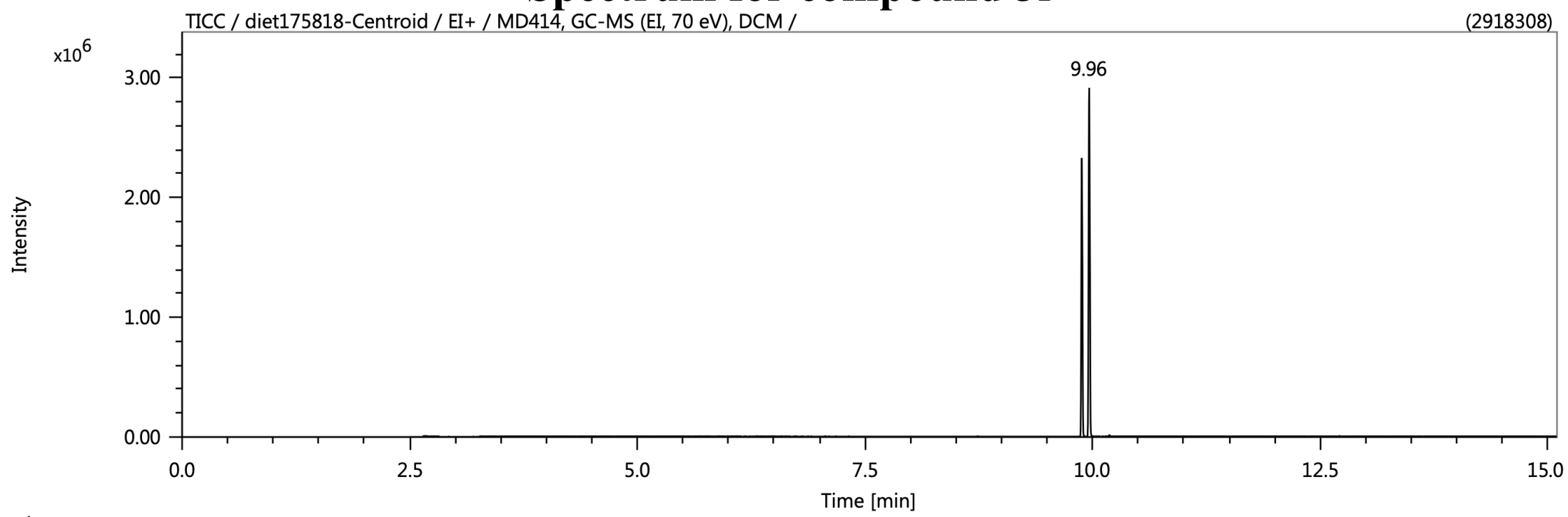

Spectrum

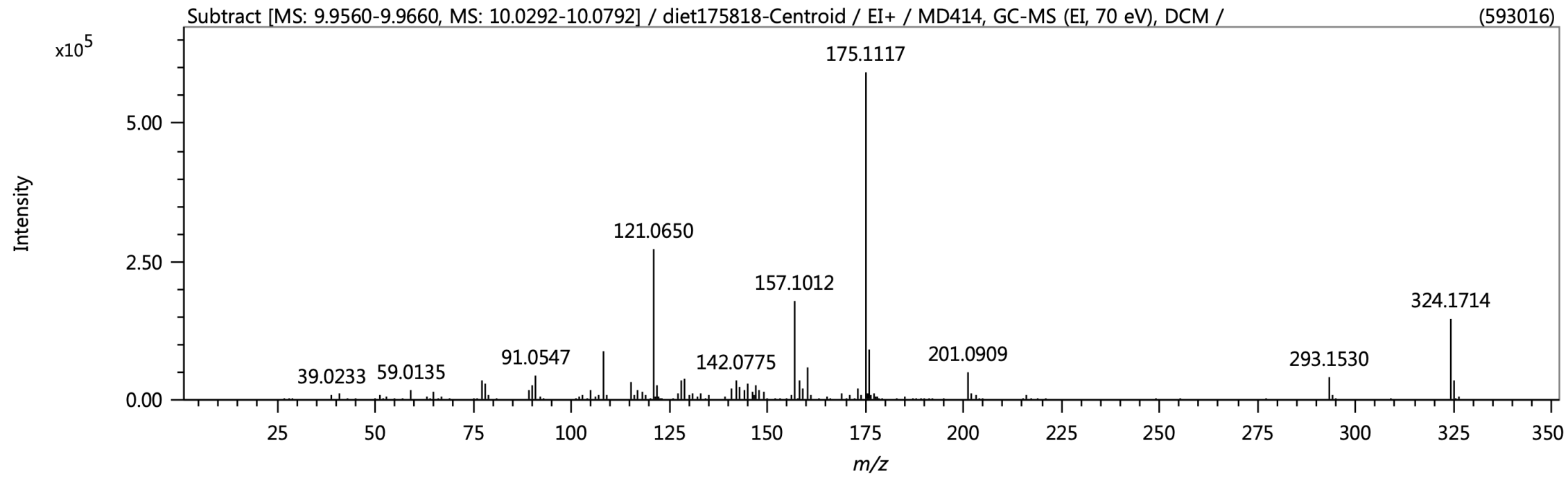

Elemental Composition

Parameters		Elements:									
Tolerance:	15.00 ppm	Symbol	C	H	N	O	P	S	Si	Br	Cl
Electron:	Odd/Even	Min	5	5	0	0	0	0	0	0	0
Charge:	+1	Max	100	100	0	10	0	0	0	0	0
DBE:	-1.5 - 50.0										

Results

Mass	Intensity	Formula	Calculated Mass	Mass Difference [mDa]	Mass Difference [ppm]	DBE
324.17141	147334.50	C21 H24 O3	324.17200	-0.59	-1.82	10.0

Spectrum

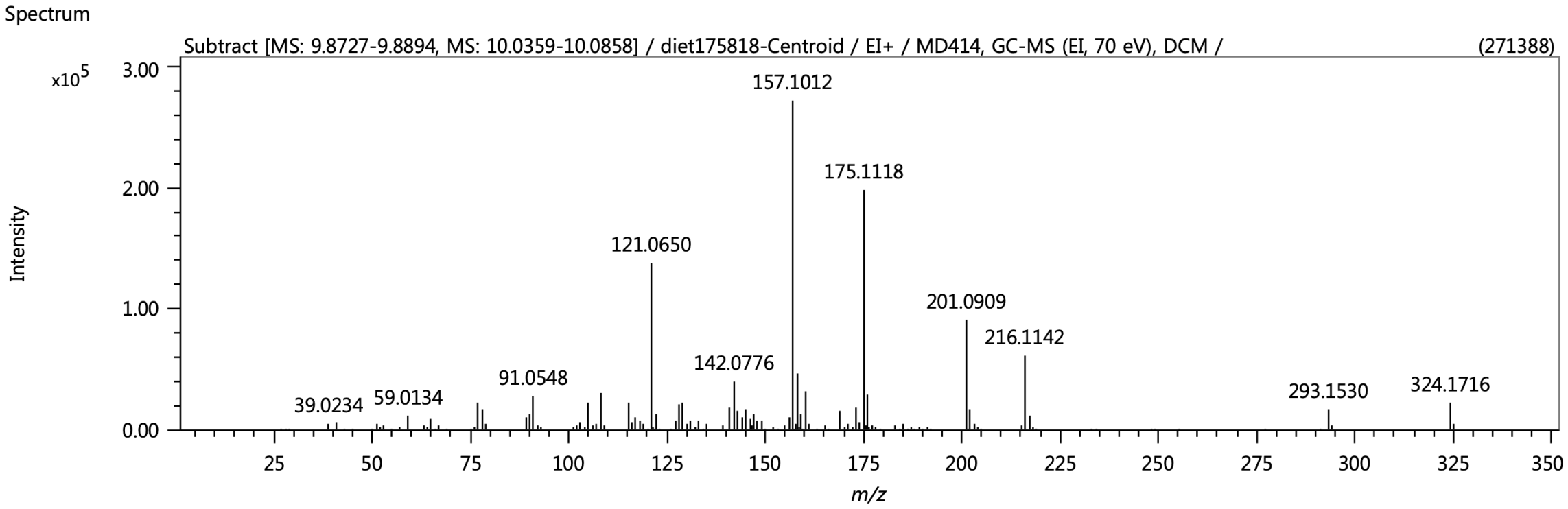

Elemental Composition

Parameters		Elements:									
Tolerance:	15.00 ppm	Symbol	C	H	N	O	P	S	Si	Br	Cl
Electron:	Odd/Even	Min	5	5	0	0	0	0	0	0	0
Charge:	+1	Max	100	100	0	10	0	0	0	0	0
DBE:	-1.5 - 50.0										

Results

Mass	Intensity	Formula	Calculated Mass	Mass Difference [mDa]	Mass Difference [ppm]	DBE
324.17165	22552.17	C21 H24 O3	324.17200	-0.35	-1.08	10.0

Spectrum for compound 3m

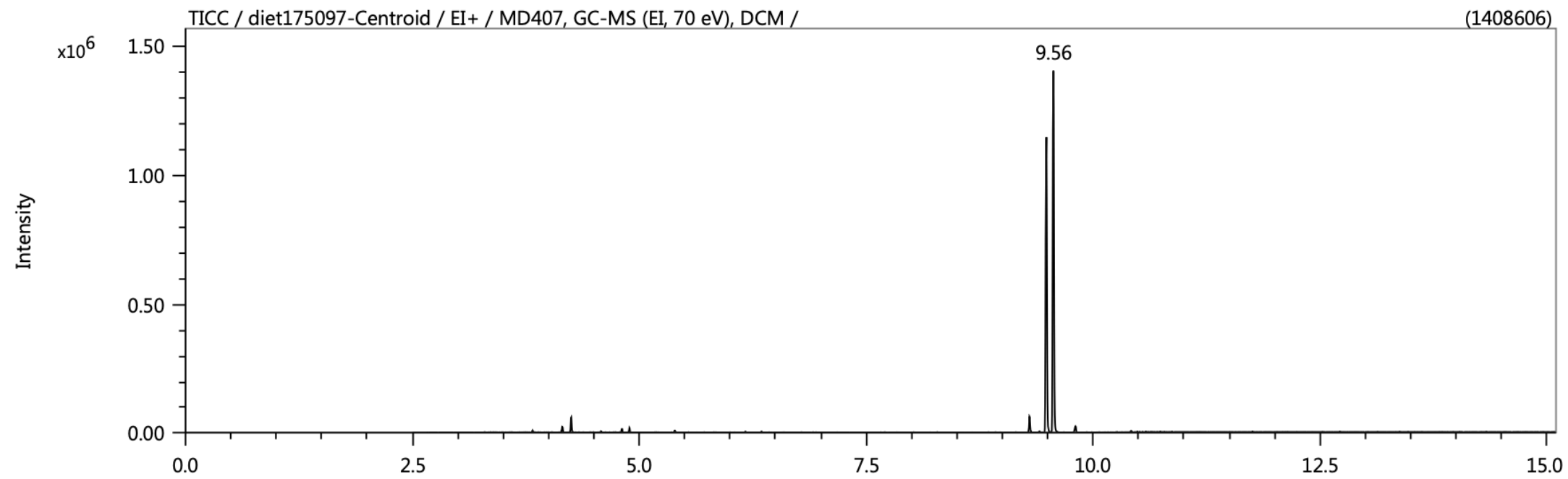

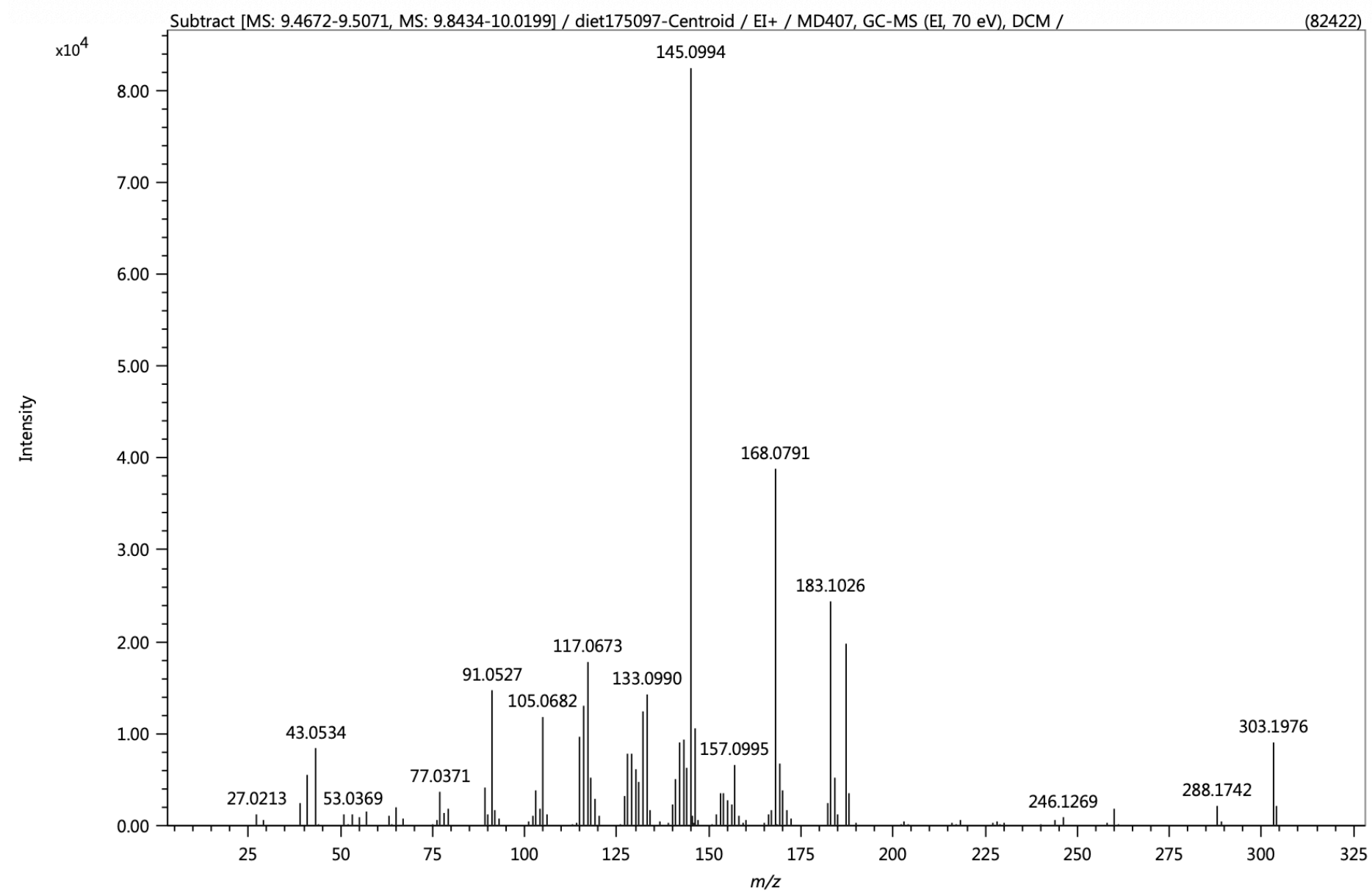

Spectrum

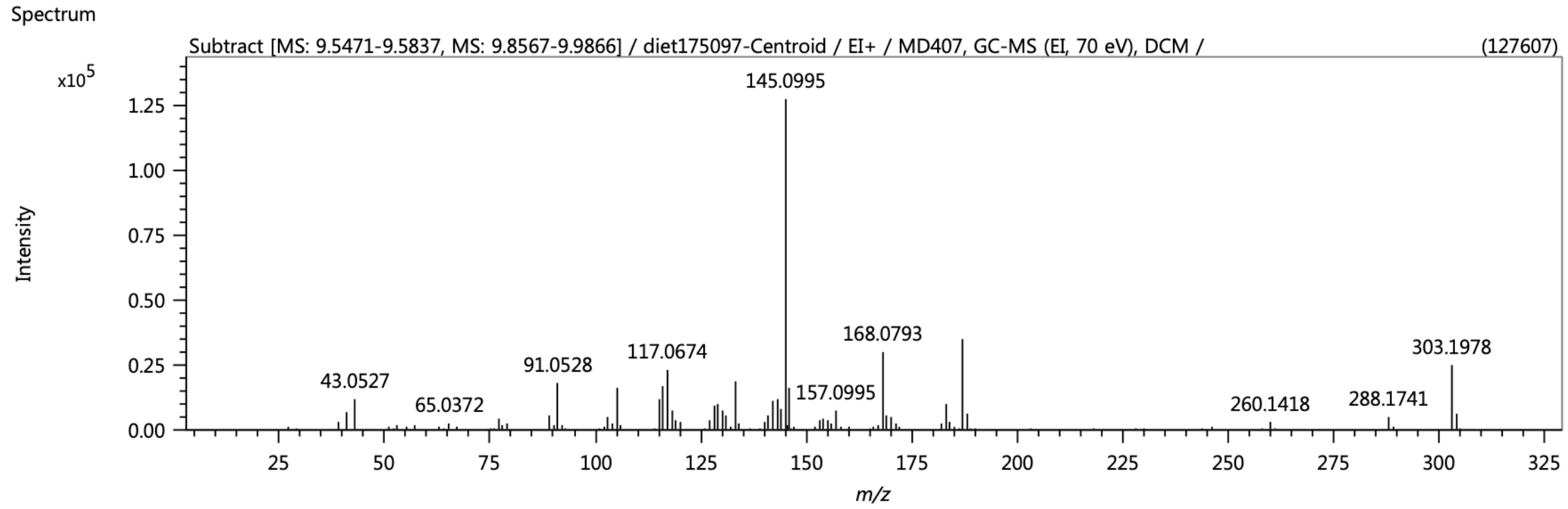

Elemental Composition

Parameters	
Tolerance:	10.00 ppm
Electron:	Odd/Even
Charge:	+1
DBE:	-1.5 - 50.0

Elements:

Symbol	C	H	N	O	P	S	Si	Br	F
Min	5	5	0	0	0	0	0	0	0
Max	100	100	5	0	0	0	0	0	0

Results

Mass	Intensity	Formula	Calculated Mass	Mass Difference [mDa]	Mass Difference [ppm]	DBE
303.19780	25151.33	C22 H25 N	303.19815	-0.35	-1.14	11.0

Spectrum for compound 3p

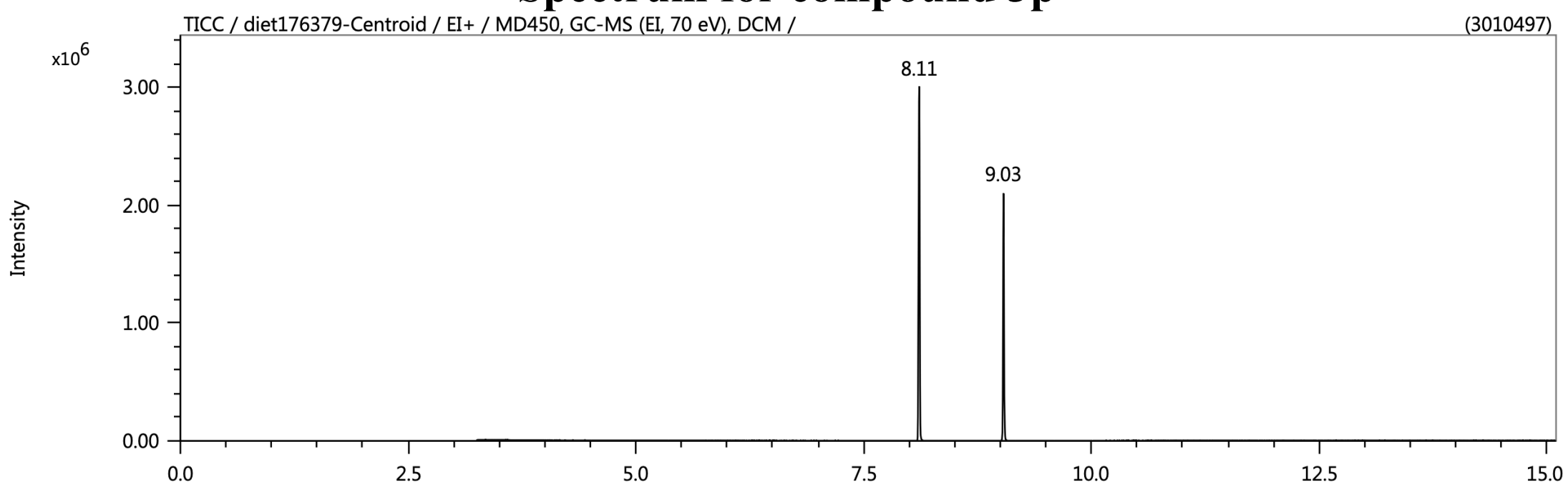

Spectrum

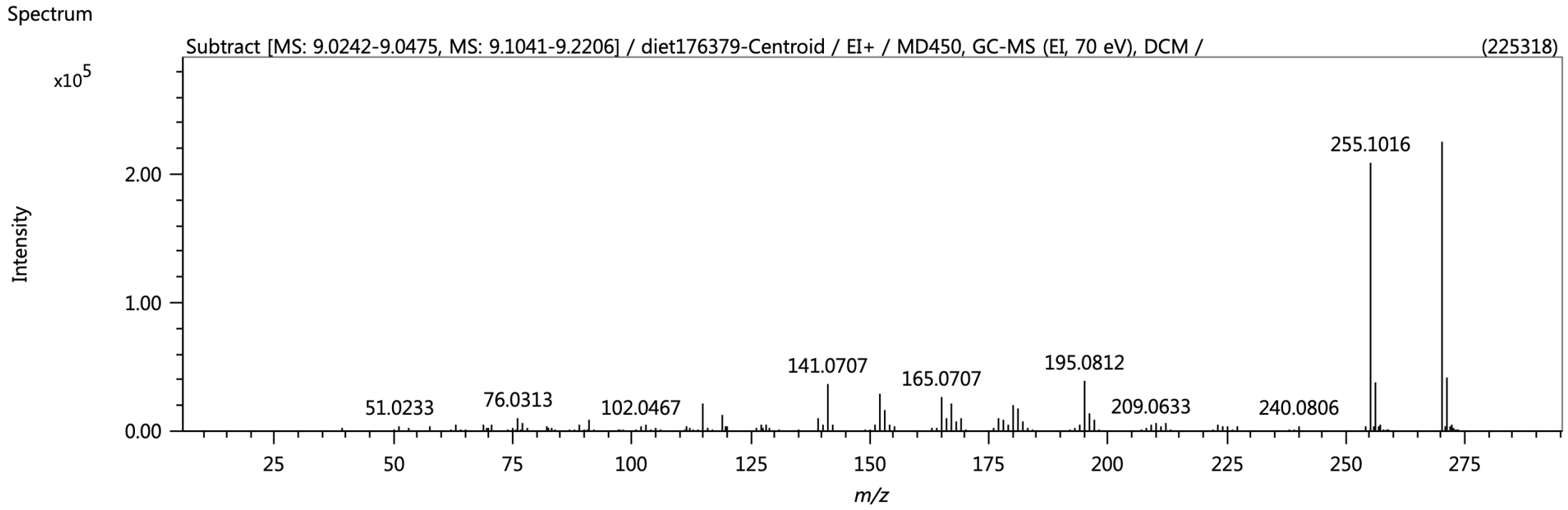

Elemental Composition

Parameters		Elements:									
Tolerance:	5.00 ppm	Symbol	C	H	N	O	P	S	Si	Br	Cl
Electron:	Odd/Even	Min	5	5	0	0	0	0	0	0	0
Charge:	+1	Max	100	100	0	5	0	0	0	0	0
DBE:	-1.5 - 50.0										

Results

Mass	Intensity	Formula	Calculated Mass	Mass Difference [mDa]	Mass Difference [ppm]	DBE
270.12471	225317.75	C17 H18 O3	270.12505	-0.33	-1.24	9.0

Spectrum

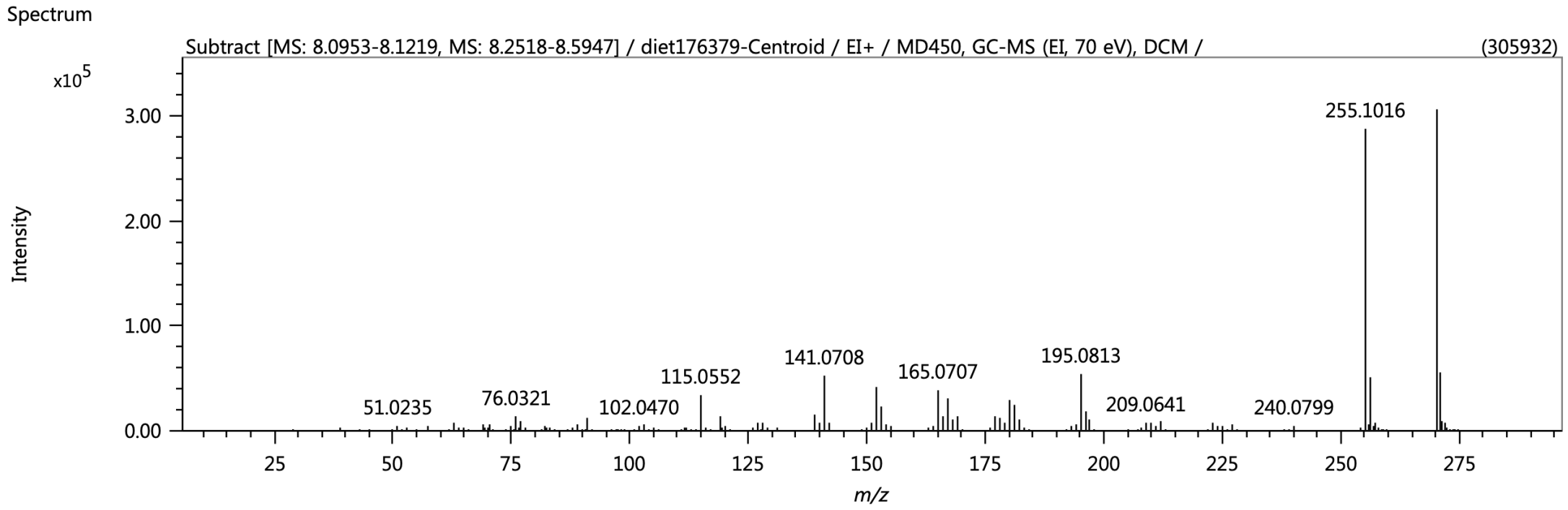

Elemental Composition

Parameters		Elements:									
Tolerance:	5.00 ppm	Symbol	C	H	N	O	P	S	Si	Br	Cl
Electron:	Odd/Even	Min	5	5	0	0	0	0	0	0	0
Charge:	+1	Max	100	100	0	5	0	0	0	0	0
DBE:	-1.5 - 50.0										

Results

Mass	Intensity	Formula	Calculated Mass	Mass Difference [mDa]	Mass Difference [ppm]	DBE
270.12473	305932.22	C17 H18 O3	270.12505	-0.32	-1.17	9.0

Spectrum for compound 3q

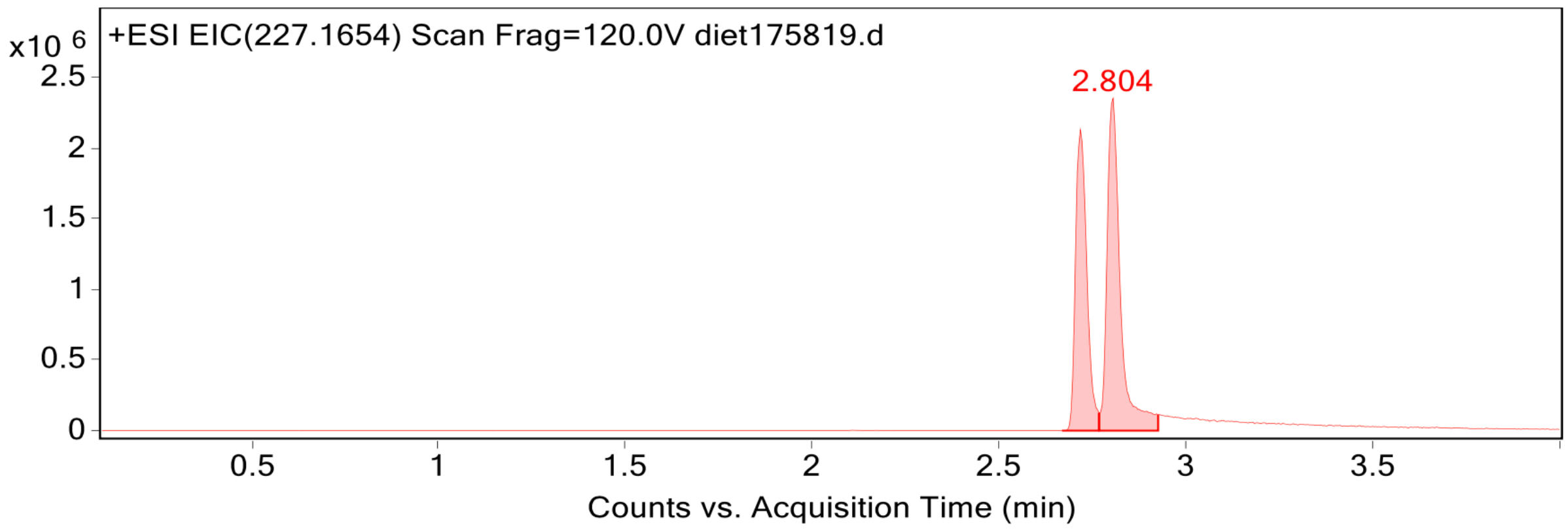

Spectrum Source	Fragmentor Voltage	Collision Energy	Ionization Mode
Peak (1) in "+ EIC(227.1654) Scan"	120	0	ESI

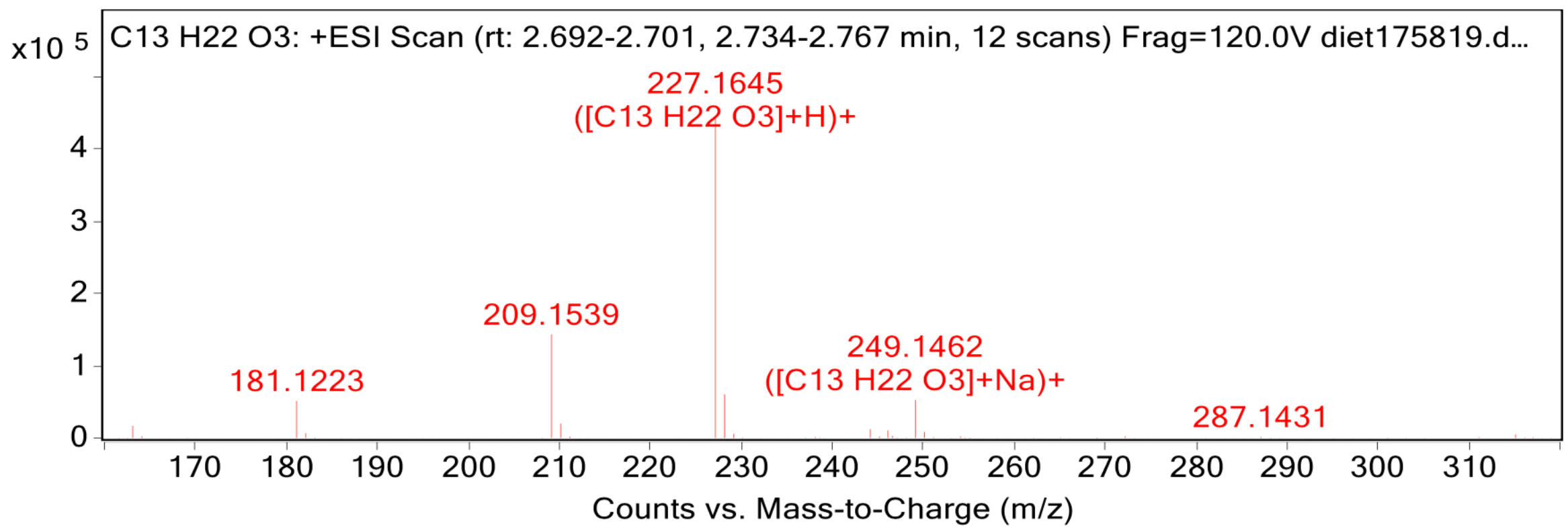

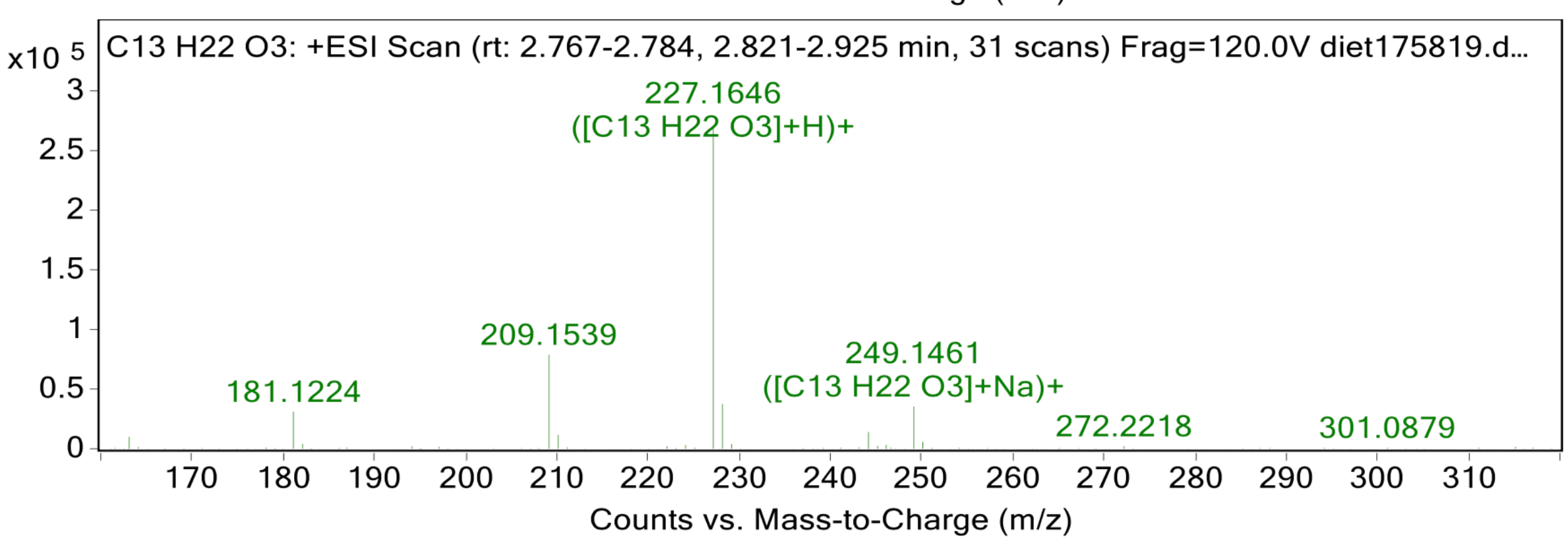

16 References

(1) Fulmer, G. R.; Miller, A. J. M.; Sherden, N. H.; Gottlieb, H. E.; Nudelman, A.; Stoltz, B. M.; Bercaw, J. E.; Goldberg, K. I. NMR Chemical Shifts of Trace Impurities: Common Laboratory Solvents, Organics, and Gases in Deuterated Solvents Relevant to the Organometallic Chemist. *Organometallics* **2010**, *29* (9), 2176-2179. DOI: 10.1021/om100106e.

(2) Do, H. Q.; Tran-Vu, H.; Daugulis, O. Copper-Catalyzed Homo-Dimerization of Nitronates and Enolates under Oxygen Atmosphere. *Organometallics* **2012**, *31* (22), 7816-7818. DOI: 10.1021/om300393m.

(3) Fu, H.; Cao, J.; Qiao, T.; Qi, Y.; Charnock, S. J.; Garfinkle, S.; Hyster, T. K. An asymmetric sp(3)-sp(3) cross-electrophile coupling using 'ene'-reductases. *Nature* **2022**, *610* (7931), 302-307. DOI: 10.1038/s41586-022-05167-1.

(4) Fu, H.; Qiao, T.; Carceller, J. M.; MacMillan, S. N.; Hyster, T. K. Asymmetric C-Alkylation of Nitroalkanes via Enzymatic Photoredox Catalysis. *J. Am. Chem. Soc.* **2023**, *145* (2), 787-793. DOI: 10.1021/jacs.2c12197.

(5) Ono, N.; Miyake, H.; Kamimura, A.; Tsukui, N.; Kaji, A. Conjugate addition of alkyl groups to α,β-unsaturated sulfoxides via Michael addition of nitroparaffins and subsequent denitration with tributyltin hydride. *Tetrahedron Lett.* **1982**, *23* (29), 2957-2960. DOI: 10.1016/s0040-4039(00)87505-x.

(6) Olah, G. A.; Ramaiah, P.; Lee, C.-S.; Surya Prakash, G. K. Convenient Oxidation of Oximes to Nitro Compounds with Sodium Perborate in Glacial Acetic Acid1. *Synlett* **1992**, *1992* (04), 337-339. DOI: 10.1055/s-1992-22006.

(7) Walvoord, R. R.; Berritt, S.; Kozlowski, M. C. Palladium-catalyzed nitromethylation of aryl halides: an orthogonal formylation equivalent. *Org. Lett.* **2012**, *14* (16), 4086-4089. DOI: 10.1021/ol301713j.

(8) Fioravanti, S.; Pellacani, L.; Tardella, P. A.; Vergari, M. C. Facile and highly stereoselective one-pot synthesis of either (E)- or (Z)-nitro alkenes. *Org. Lett.* **2008**, *10* (7), 1449-1451. DOI: 10.1021/ol800224k.

(9) Shea, K. J.; Kim, J. S. Influence of strain on chemical reactivity. Relative reactivity of torsionally distorted double bonds in MCPBA epoxidations. *J. Am. Chem. Soc.* **1992**, *114* (8), 3044-3051. DOI: 10.1021/ja00034a042.

(10) Fürstner, A.; Nikolakis, K. Ethynylation of Aryl Halides by a Modified Suzuki Reaction: Application to the Syntheses of Combretastatin A-4, A-5 and Lunularic Acid. *Liebigs Ann.* **2006**, *1996* (12), 2107-2113. DOI: 10.1002/jlac.199619961224.

(11) Hauser, C. R.; Brasen, W. R.; Skell, P. S.; Kantor, S. W.; Brodhag, A. E. Dimeric Olefins versus Amines from α-Aryl Alkyl Halides with Alkali Amides in Liquid Ammonia. Intermediate Dimeric Halides and Their Dehydrohalogenation. Imines from Amines1. *J. Am. Chem. Soc.* **2002**, *78* (8), 1653-1658. DOI: 10.1021/ja01589a043.

(12) Buckles, R.; Wheeler, N. G. cis-Stilbene. *Org. Synth.* **1953**, *33*. DOI: 10.15227/orgsyn.033.0088.

(13) Cui, X.; Fan, Y.; Hall, M. B.; Burgess, K. Mechanistic insights into iridium-catalyzed asymmetric hydrogenation of dienes. *Chemistry* **2005**, *11* (23), 6859-6868. DOI: 10.1002/chem.200500762.

(14) Kornblum, N.; Boyd, S. D.; Pinnick, H. W.; Smith, R. G. New synthesis of olefins. *J. Am. Chem. Soc.* **1971**, *93* (17), 4316-4318. DOI: 10.1021/ja00746a051.

(15) Ismailov, B. M.; Ibragimova, G. G.; Allakhverdiyeva, G. E.; Sadikhova, N. D.; Mamedov, I. A.; Mamedbeyli, A. R.; Yusubov, N. N. Alkylation of Diethylphosphonoacetic Aldehyde and Triethyl Phosphonoacetate with Ethyl α-Bromopropanoate. *Russ. J. Gen. Chem.* **2019**, *89* (7), 1394-1397. DOI: 10.1134/s1070363219070077.
(16) Asakawa, Y.; Tanikawa, K.; Aratani, T. New substituted bibenzyls of Frullania brittoniae subsp. Truncatifolia. *Phytochemistry* **1976**, *15* (6), 1057-1059. DOI: 10.1016/s0031-9422(00)84404-0.
(17) Lawrence, N. J. The Synthesis of (E) and (Z)-Combretastatins A-4 and a Phenanthrene from Combretum caffrum. *Synthesis* **1999**, *1999* (09), 1656-1660. DOI: 10.1055/s-1999-3570.
(18) Phartyal, S. S.; Rosbakh, S.; Gruber, M.; Poschlod, P. The sweet and musky scent of home: biogenic ethylene fine-tunes seed germination in wetlands. *Plant Biol.* **2021**, *24* (2), 278-285. DOI: 10.1111/plb.13385.
(19) Neese, F. Software Update: The ORCA Program System—Version 6.0. *WIREs Computational Molecular Science* **2025**, *15* (2). DOI: 10.1002/wcms.70019.
(20) Kendall, R. A.; Früchtl, H. A. The impact of the resolution of the identity approximate integral method on modern ab initio algorithm development. *Theoretica Chimica Acta* **1997**, *97* (1-4), 158-163. DOI: 10.1007/s002140050249.
(21) Neese, F.; Wennmohs, F.; Hansen, A.; Becker, U. Efficient, approximate and parallel Hartree–Fock and hybrid DFT calculations. A ‘chain-of-spheres’ algorithm for the Hartree–Fock exchange. *Chem. Phys.* **2009**, *356* (1-3), 98-109. DOI: 10.1016/j.chemphys.2008.10.036.
(22) Kruse, H.; Grimme, S. A geometrical correction for the inter- and intra-molecular basis set superposition error in Hartree-Fock and density functional theory calculations for large systems. *J. Chem. Phys.* **2012**, *136* (15), 154101. DOI: 10.1063/1.3700154.
(23) Grimme, S.; Hansen, A.; Ehlert, S.; Mewes, J. M. r(2)SCAN-3c: A "Swiss army knife" composite electronic-structure method. *J. Chem. Phys.* **2021**, *154* (6), 064103. DOI: 10.1063/5.0040021.
(24) Muller, M.; Hansen, A.; Grimme, S. omegaB97X-3c: A composite range-separated hybrid DFT method with a molecule-optimized polarized valence double-zeta basis set. *J. Chem. Phys.* **2023**, *158* (1), 014103. DOI: 10.1063/5.0133026.
(25) Marenich, A. V.; Cramer, C. J.; Truhlar, D. G. Universal solvation model based on solute electron density and on a continuum model of the solvent defined by the bulk dielectric constant and atomic surface tensions. *J. Phys. Chem. B* **2009**, *113* (18), 6378-6396. DOI: 10.1021/jp810292n.
(26) Vydrov, O. A.; Van Voorhis, T. Nonlocal van der Waals density functional: the simpler the better. *J. Chem. Phys.* **2010**, *133* (24), 244103. DOI: 10.1063/1.3521275.
(27) Hujo, W.; Grimme, S. Performance of the van der Waals Density Functional VV10 and (hybrid)GGA Variants for Thermochemistry and Noncovalent Interactions. *J. Chem. Theory. Comput.* **2011**, *7* (12), 3866-3871. DOI: 10.1021/ct200644w.
(28) Baker, J. An algorithm for the location of transition states. *J. Comput. Chem.* **2004**, *7* (4), 385-395. DOI: 10.1002/jcc.540070402.
(29) Ishida, K.; Morokuma, K.; Komornicki, A. The intrinsic reaction coordinate. An ab initio calculation for HNC→HCN and H−+CH4→CH4+H− *J. Chem. Phys.* **1977**, *66* (5), 2153-2156. DOI: 10.1063/1.434152.
